# Supplementary material for: One-pot multicomponent nitro-Mannich reaction using a heterogeneous catalyst under solvent-free conditions
Source: PeerJ. 2018 Jun 27;6:e5065. doi: 10.7717/peerj.5065 (PMC6026460; doi:10.7717/peerj.5065)
Supplement: File S1 [file peerj-06-5065-s001.pdf]

IR, ^{1}H NMR, ^{13}C NMR and Mass spectra of all products

6 *N*-(2-nitro-1-phenylethyl)aniline

Table 1.1: MS data.

Compound	Formula/Mass		Parent m/z	Cone Voltage	Daughters	Collision Energy	Ion Mode
**6**	242	1	241.03	20	92.04	56	ES-
		2	242.07	30	72.98	24	ES+
		3	242.07	30	94.06	26	ES+
		4	242.07	30	86.98	24	ES+
		5	242.07	30	122.03	24	ES+

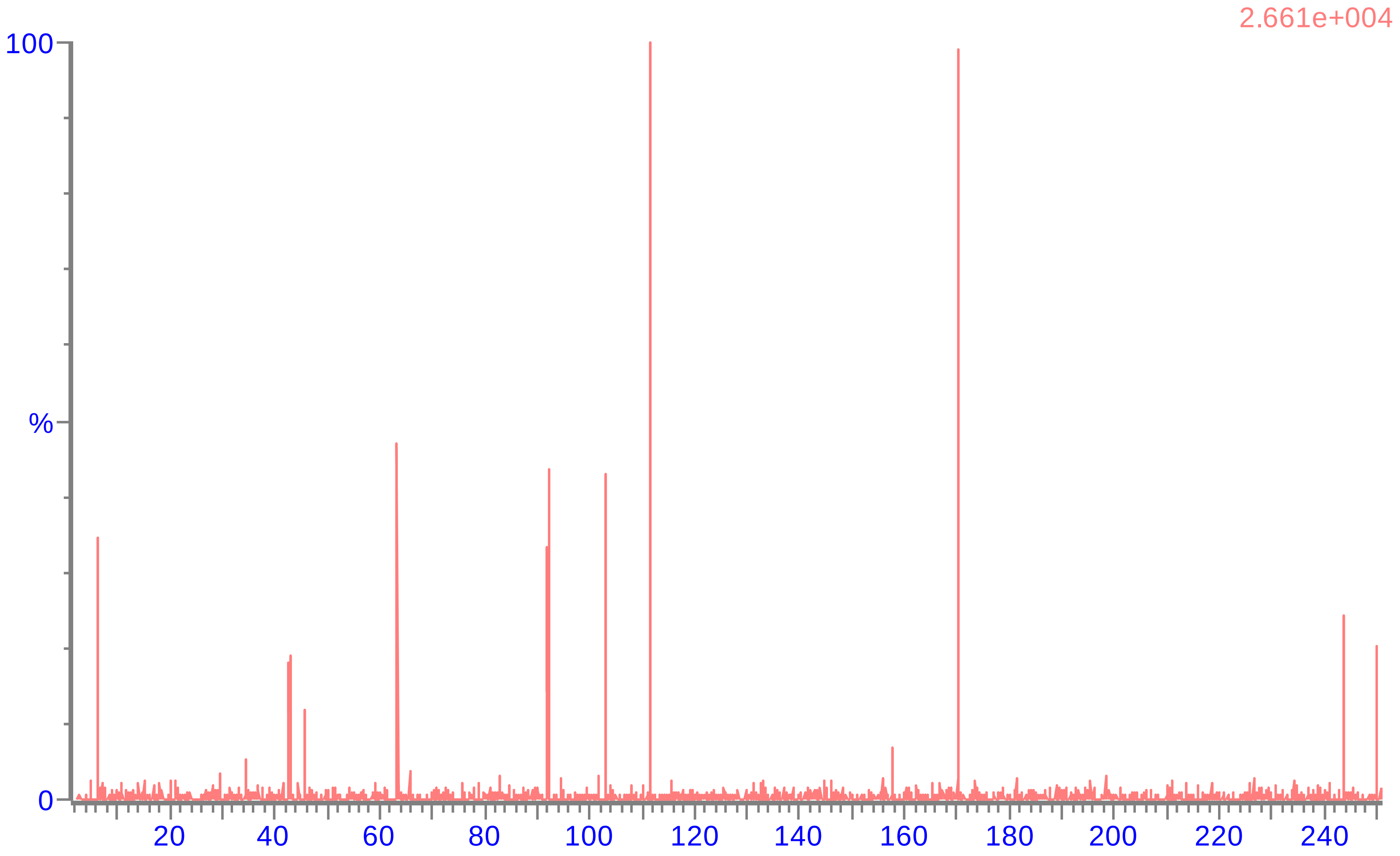

Figure 1.1: Mass spectrum for daughter fragment peak ES+, m/z 241.03 -> 92.04.

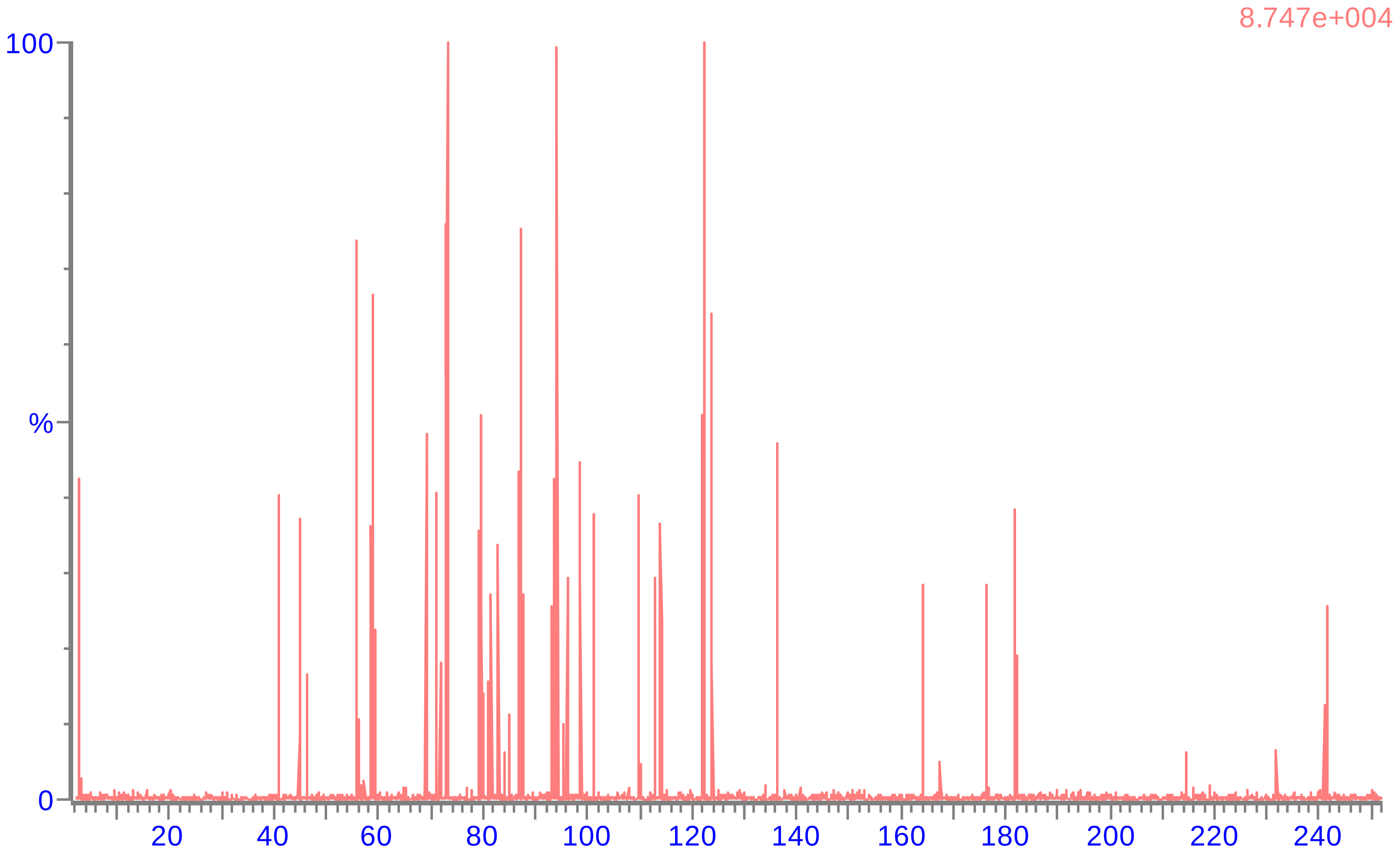

Figure 1.2: Mass spectrum for daughter fragment peak ES+, m/z 241.03 -> 72.98.

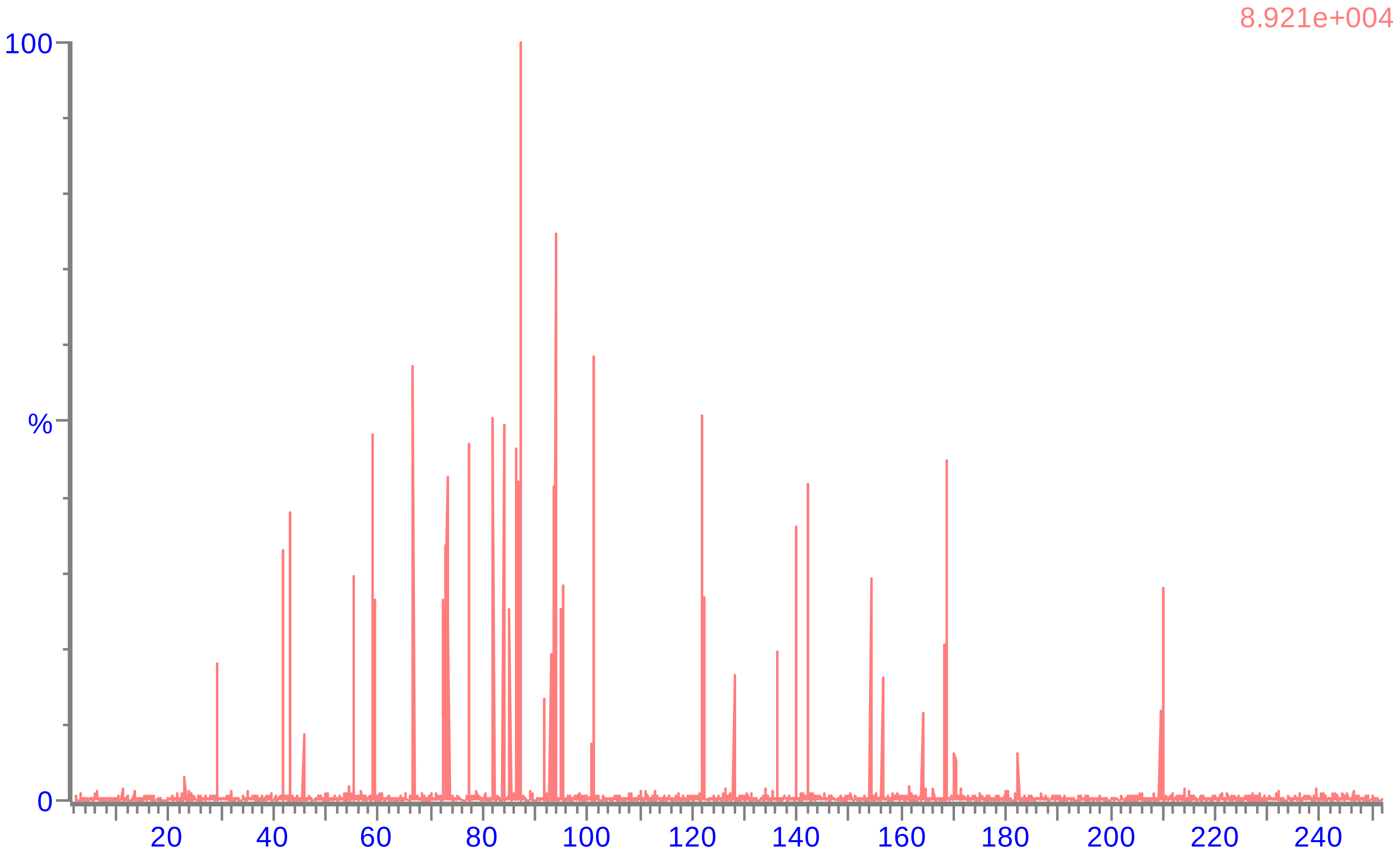

Figure 1.3: Mass spectrum for daughter fragment peak ES+, m/z 241.03 -> 94.06.

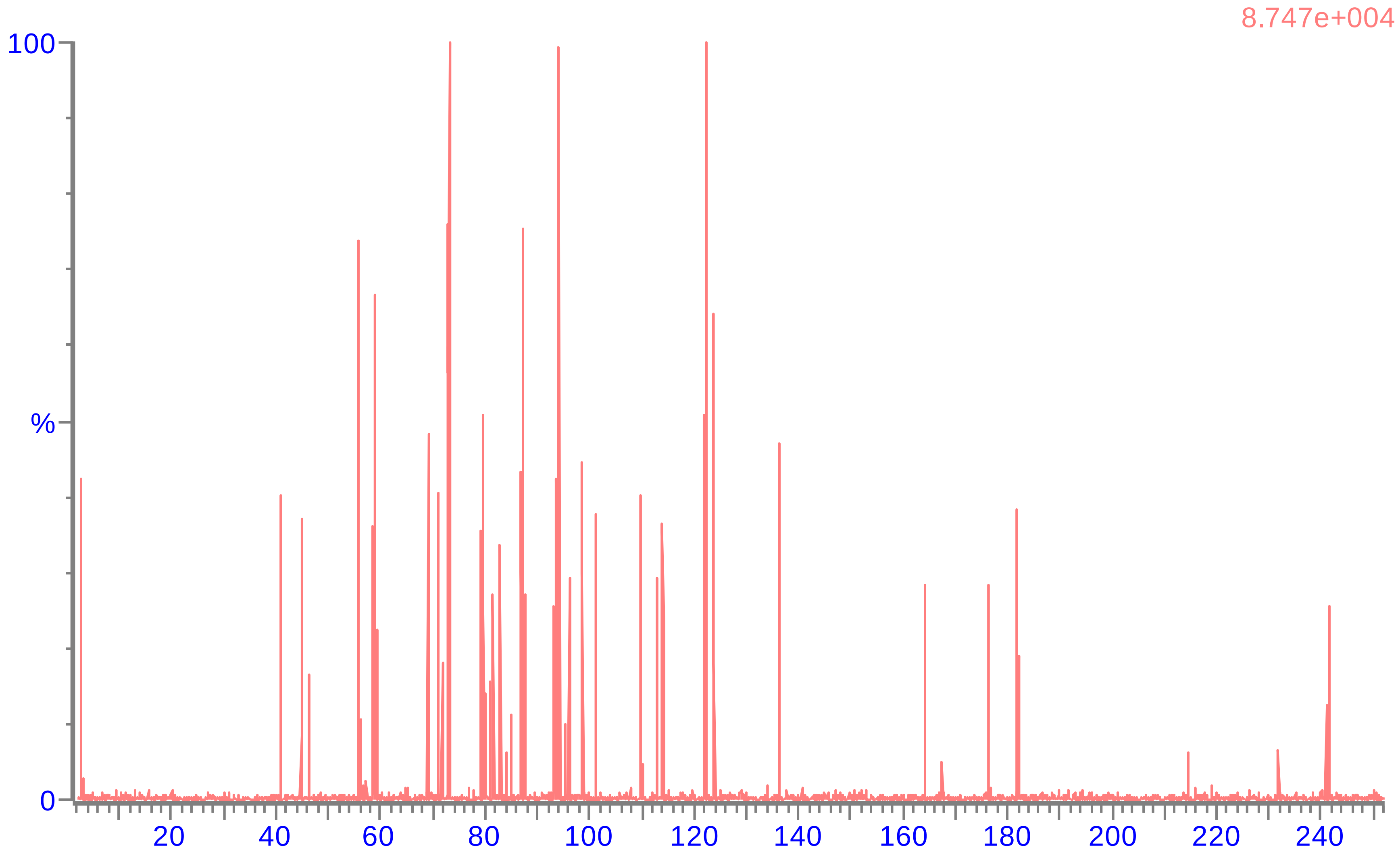

Figure 1.4: Mass spectrum for daughter fragment peak ES+, m/z 241.03 -> 86.98.

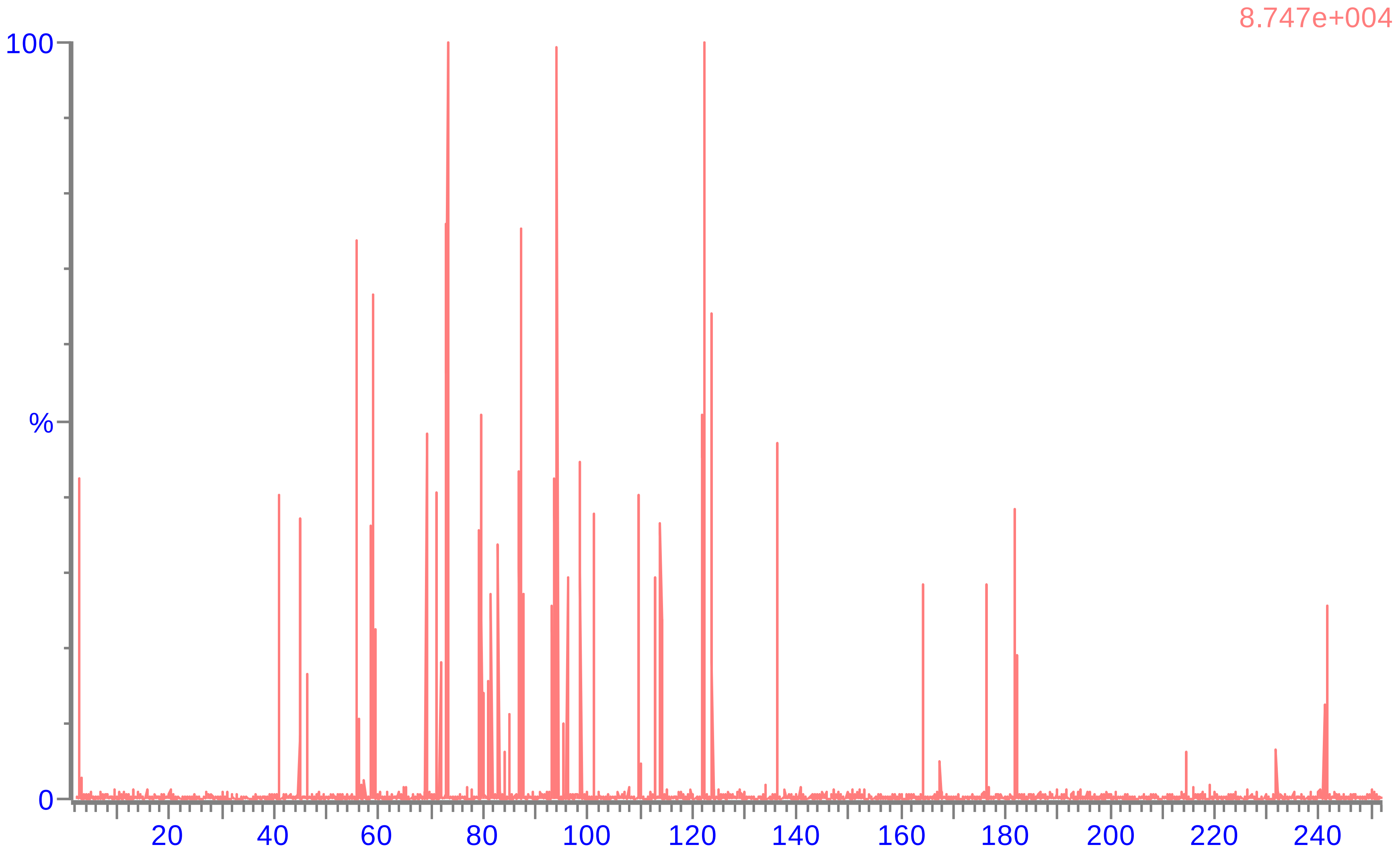

Figure 1.5: Mass spectrum for daughter fragment peak ES+, m/z 241.03 -> 122.03.

7a *N*-(2-nitro-1-(*p*-tolyl)ethyl)aniline

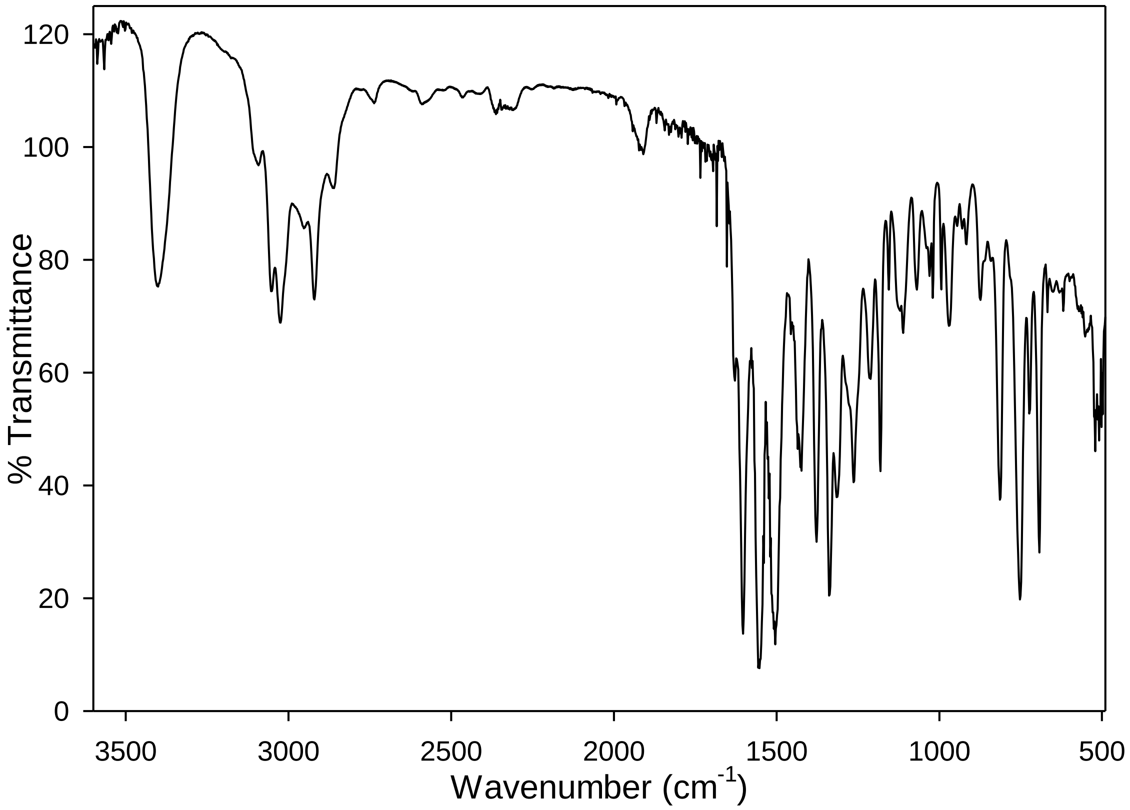

Figure 1.6: IR spectrum of **7a** *N*-(2-nitro-1-(*p*-tolyl)ethyl)aniline.

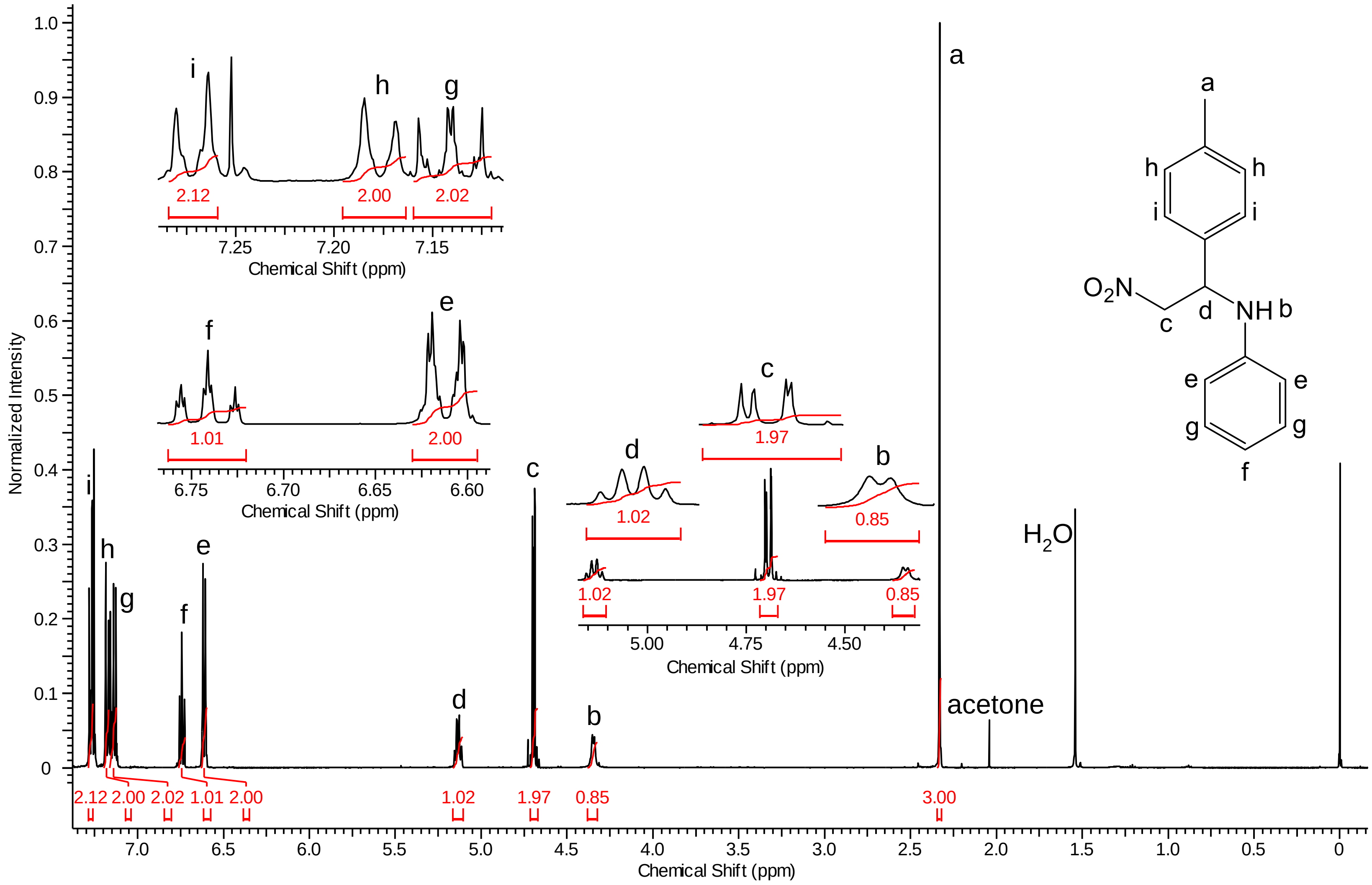

Figure 1.7: ^{1}H NMR spectrum of **7a** *N*-(2-nitro-1-(*p*-tolyl)ethyl)aniline.

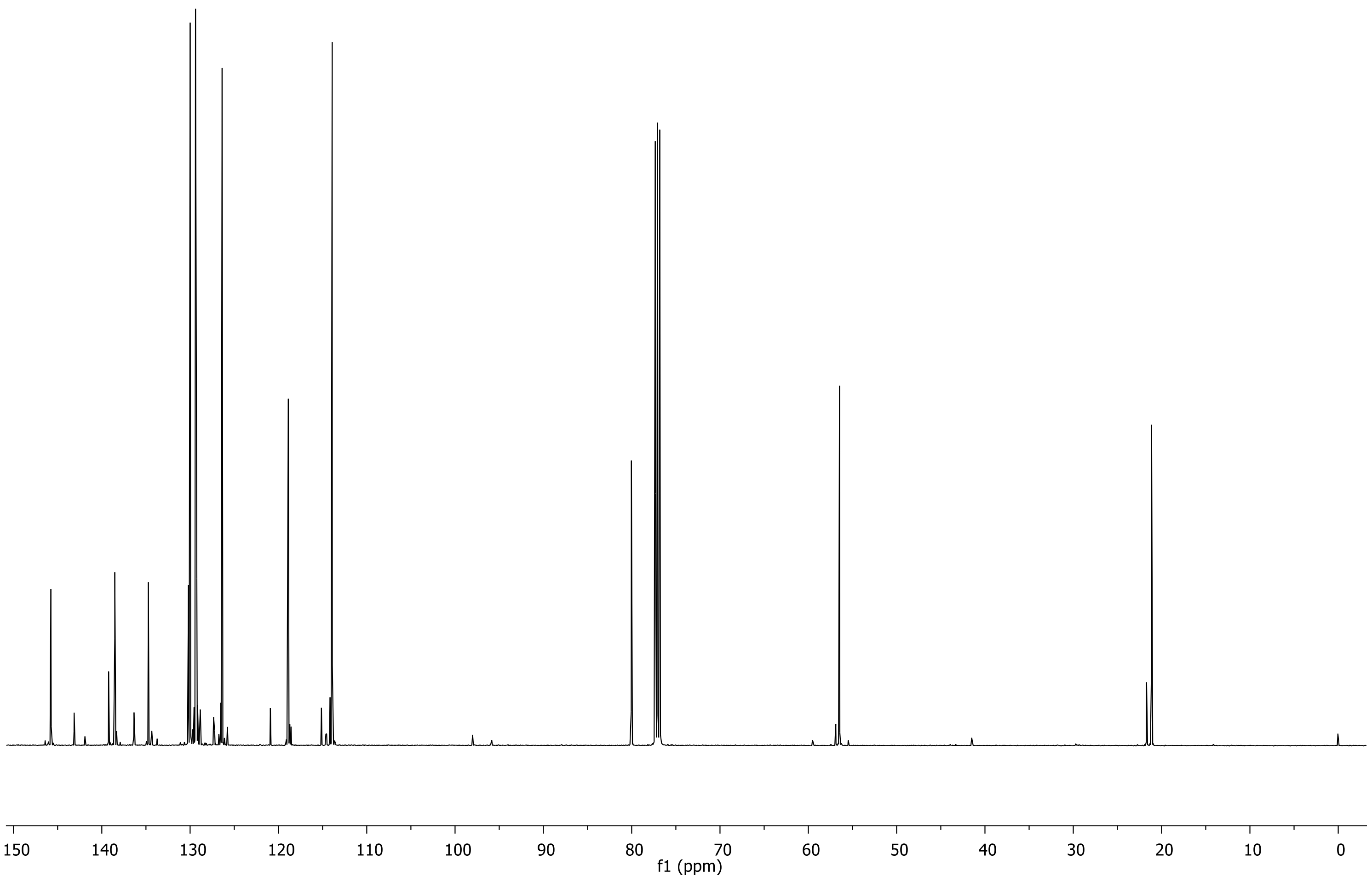

Figure 1.8: ^{13}C NMR spectrum of **7a** *N*-(2-nitro-1-(*p*-tolyl)ethyl)aniline.

Table 1.2: MS data

Compound	Formula/Mass		Parent m/z	Cone Voltage	Daughters	Collision Energy	Ion Mode
**7a**	256	1	257.10	16	118.06	18	ES+
		2	257.10	16	94.06	10	ES+
		3	257.10	16	164.05	10	ES+
		4	257.10	16	196.12	8	ES+
		5	257.10	16	91.07	52	ES+

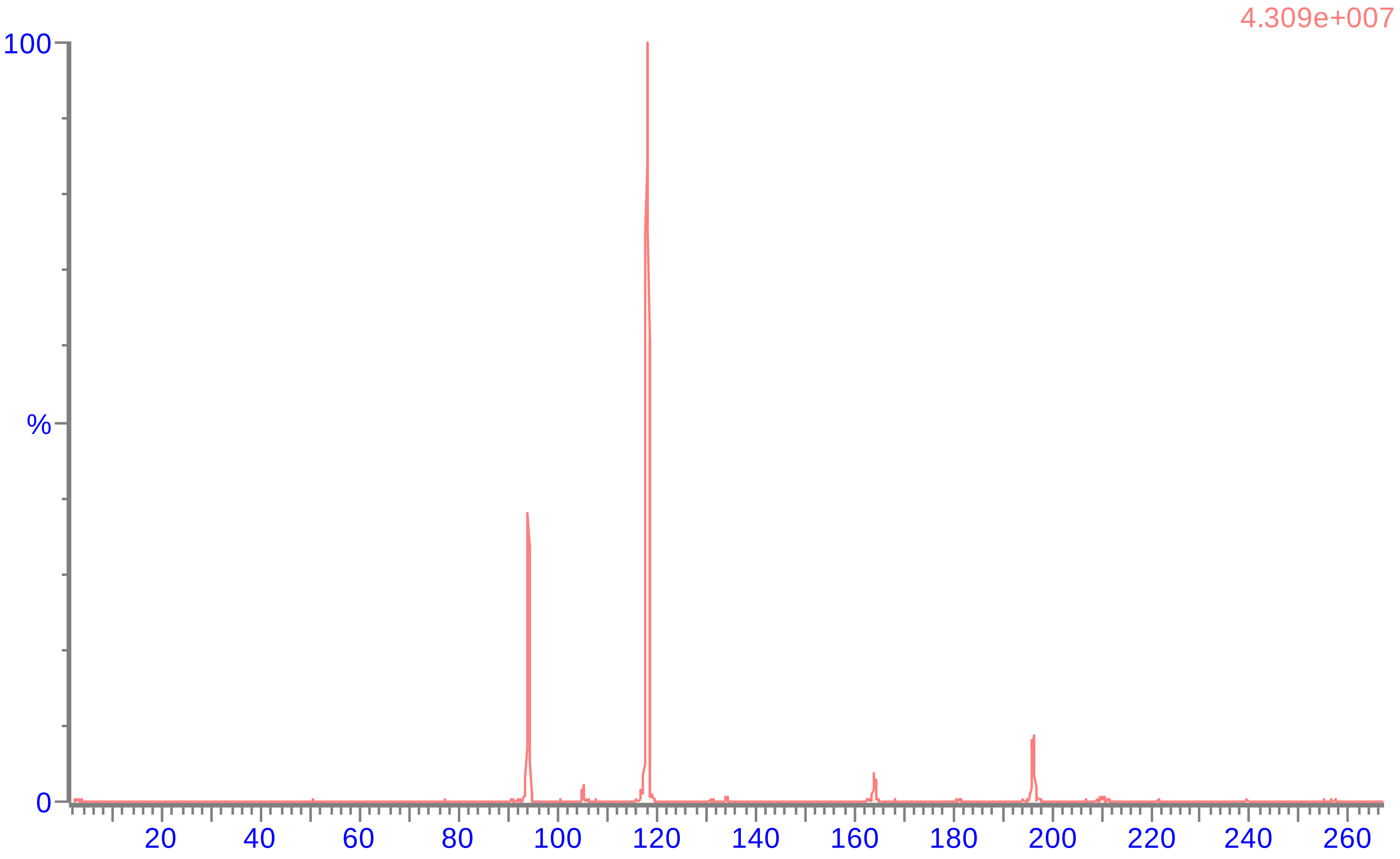

Figure 1.9: Mass spectrum for daughter fragment peak ES+, m/z 257.10 -> 118.06.

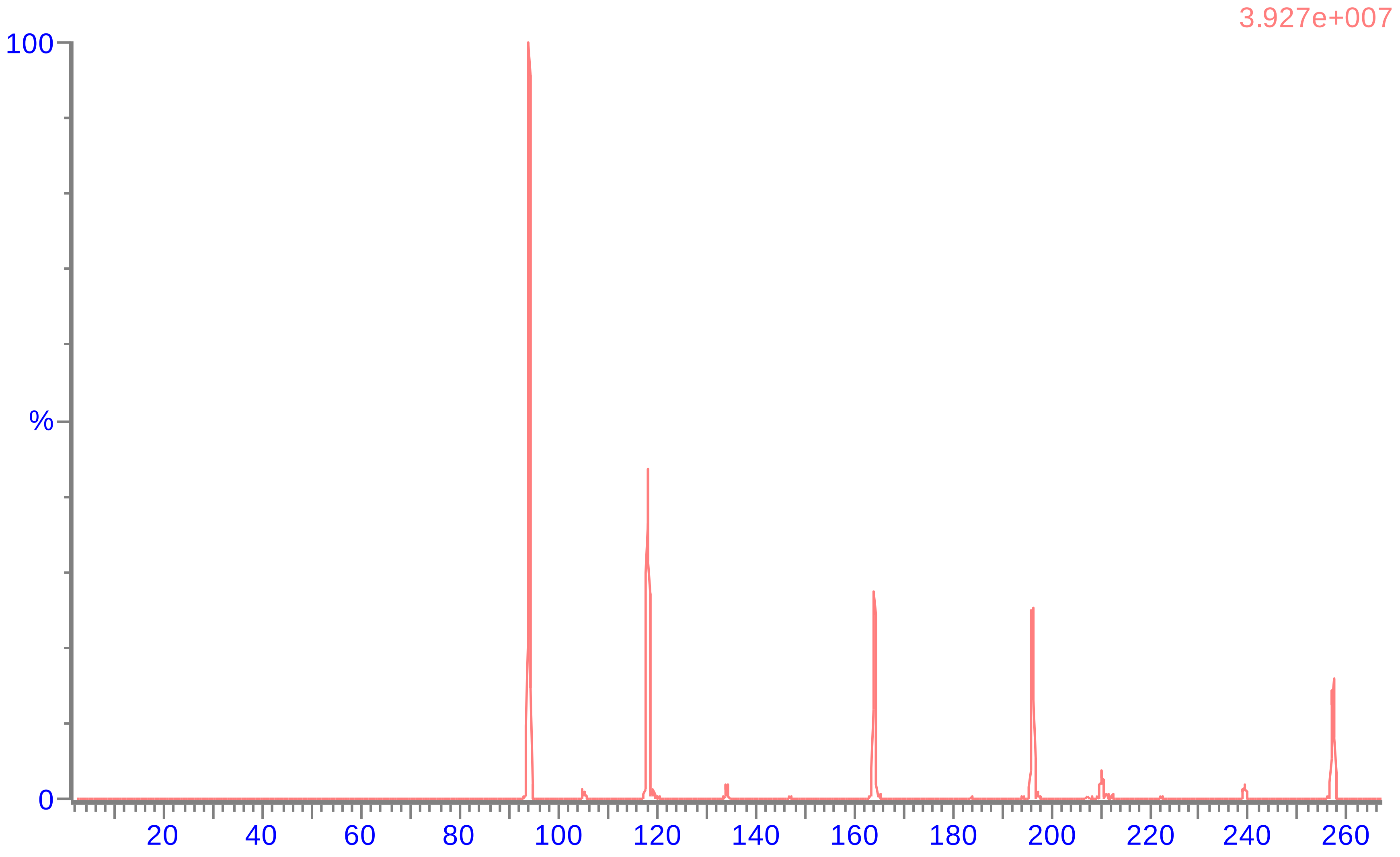

Figure 1.10: Mass spectrum for daughter fragment peak ES+, m/z 257.10 -> 94.06.

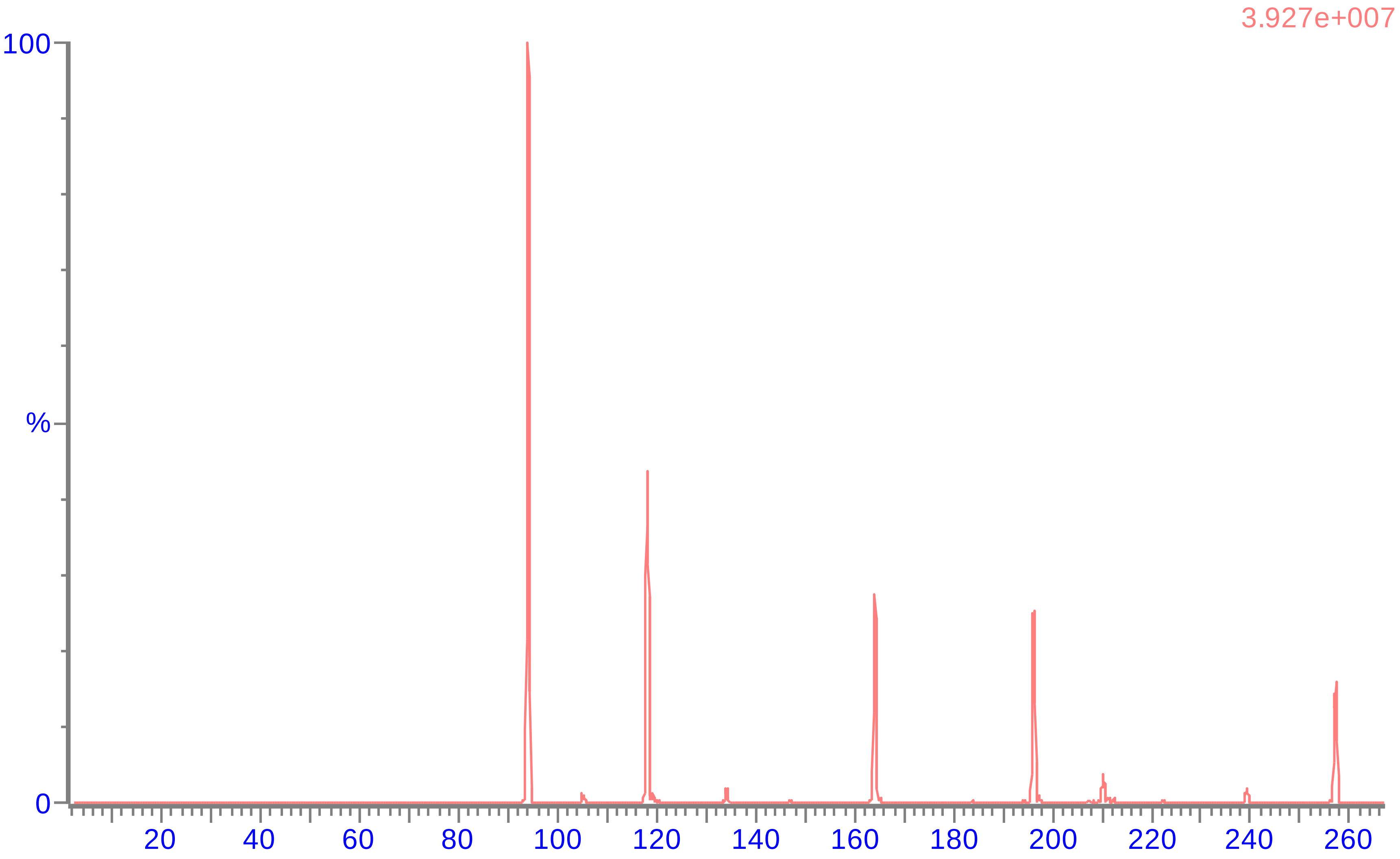

Figure 1.11: Mass spectrum for daughter fragment peak ES+, m/z 257.10 -> 164.05.

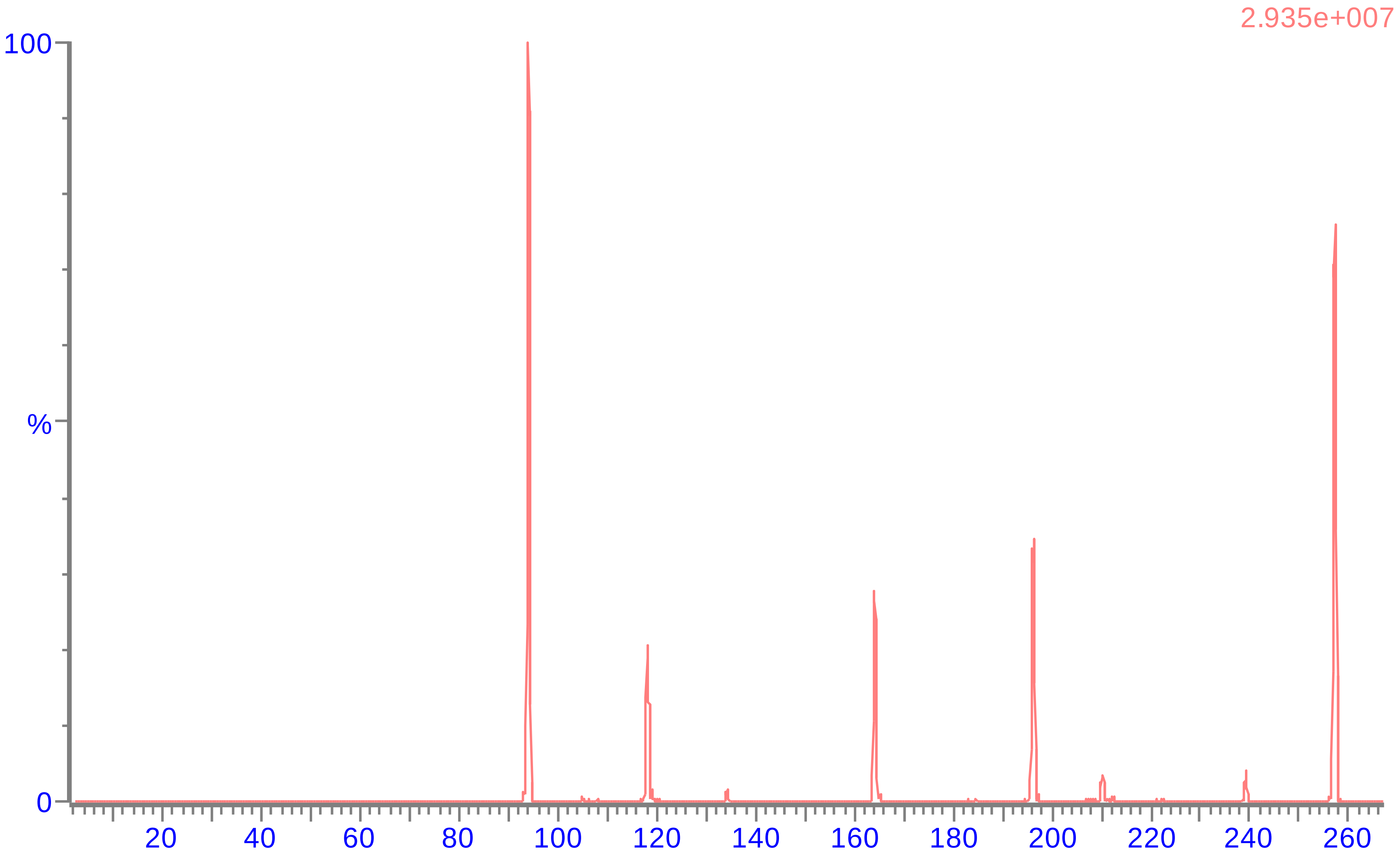

Figure 1.12: Mass spectrum for daughter fragment peak ES+, m/z 257.10 -> 196.12.

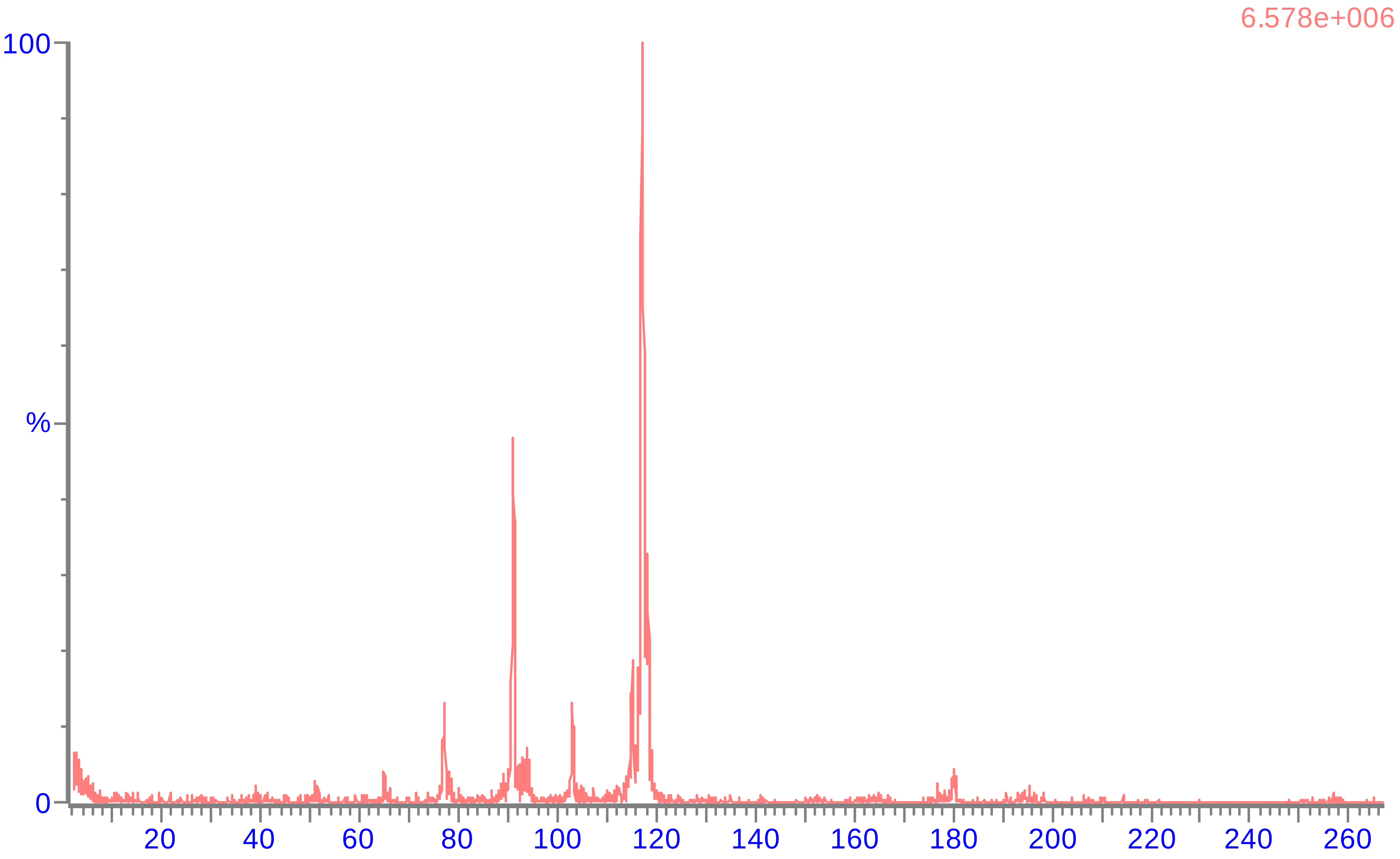

Figure 1.13: Mass spectrum for daughter fragment peak ES+, m/z 257.10 -> 91.07.

7b *N*-(1-(4-methoxyphenyl)-2-nitroethyl)aniline

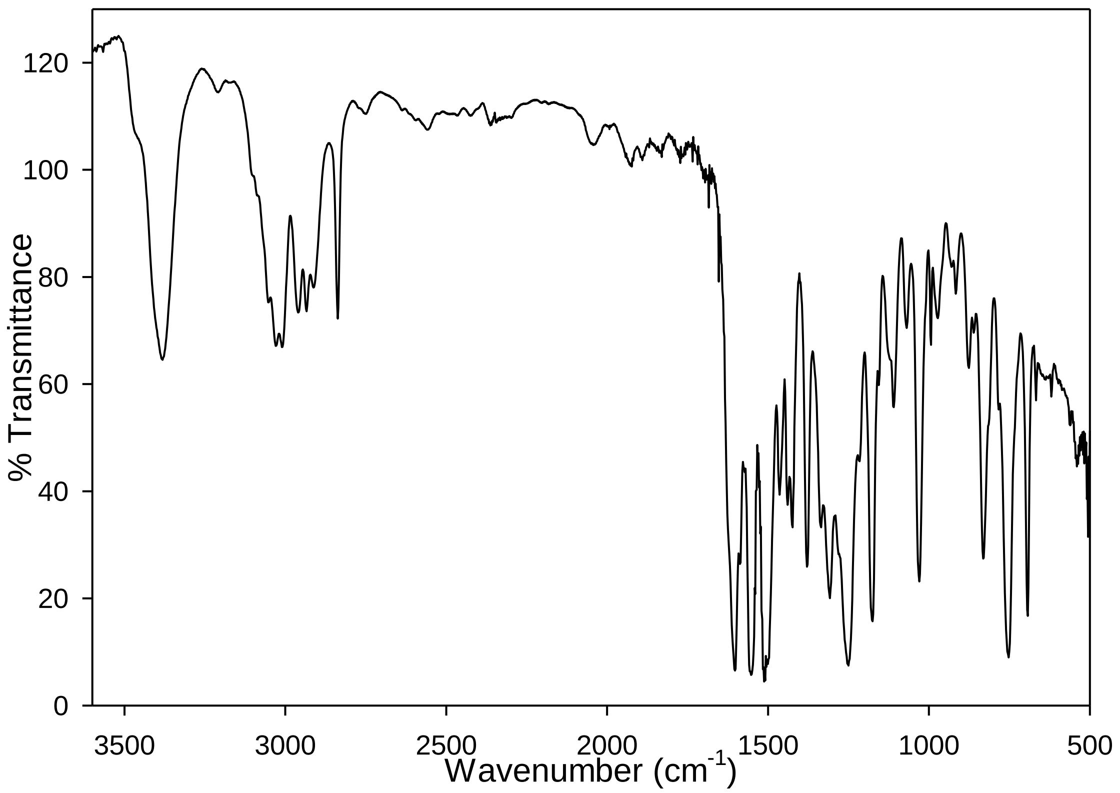

Figure 1.14: IR spectrum of **7b** *N*-(1-(4-methoxyphenyl)-2-nitroethyl)aniline.

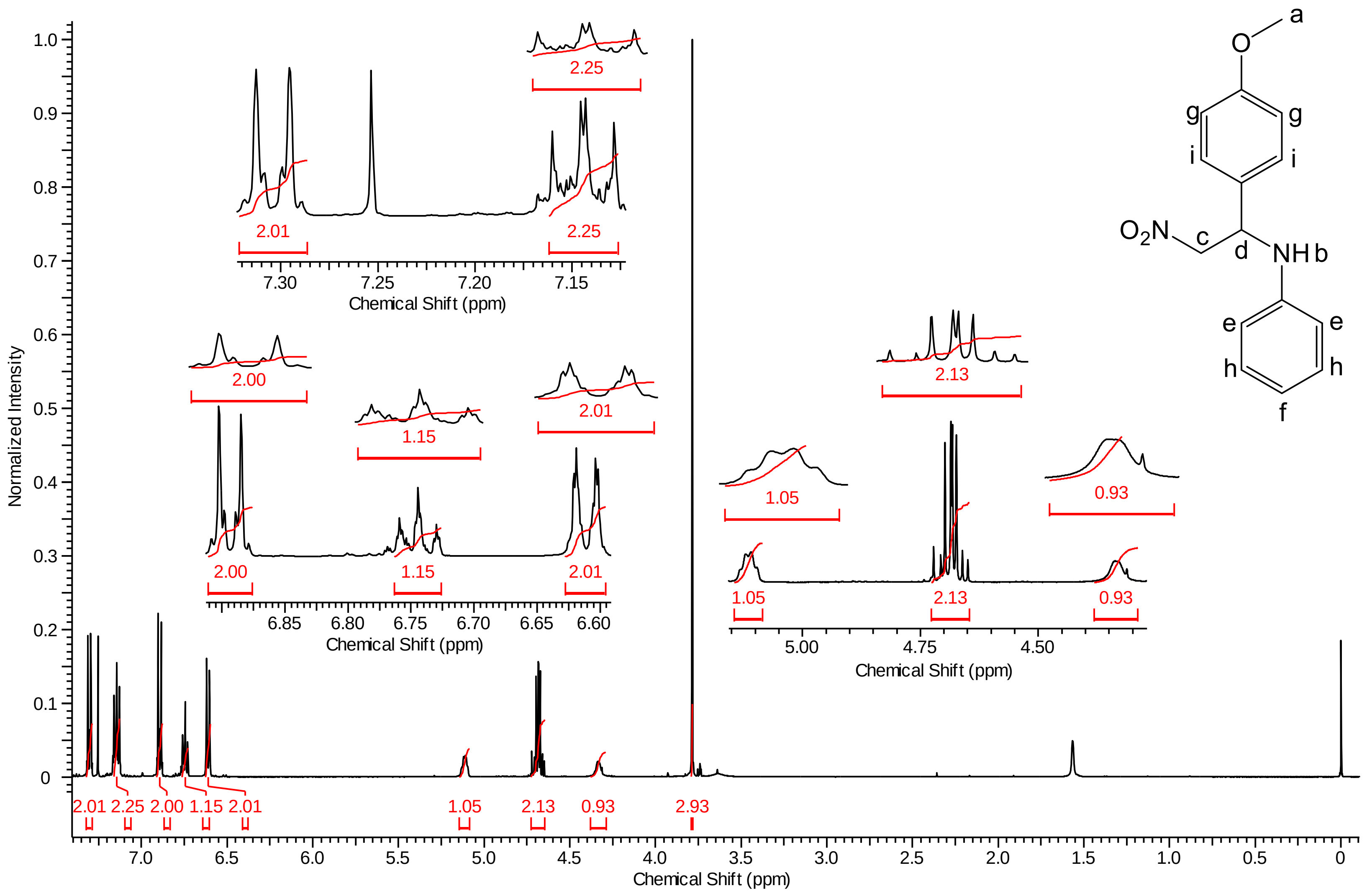

Figure 1.15: ^{1}H NMR spectrum of **7b** *N*-(1-(4-methoxyphenyl)-2-nitroethyl)aniline.

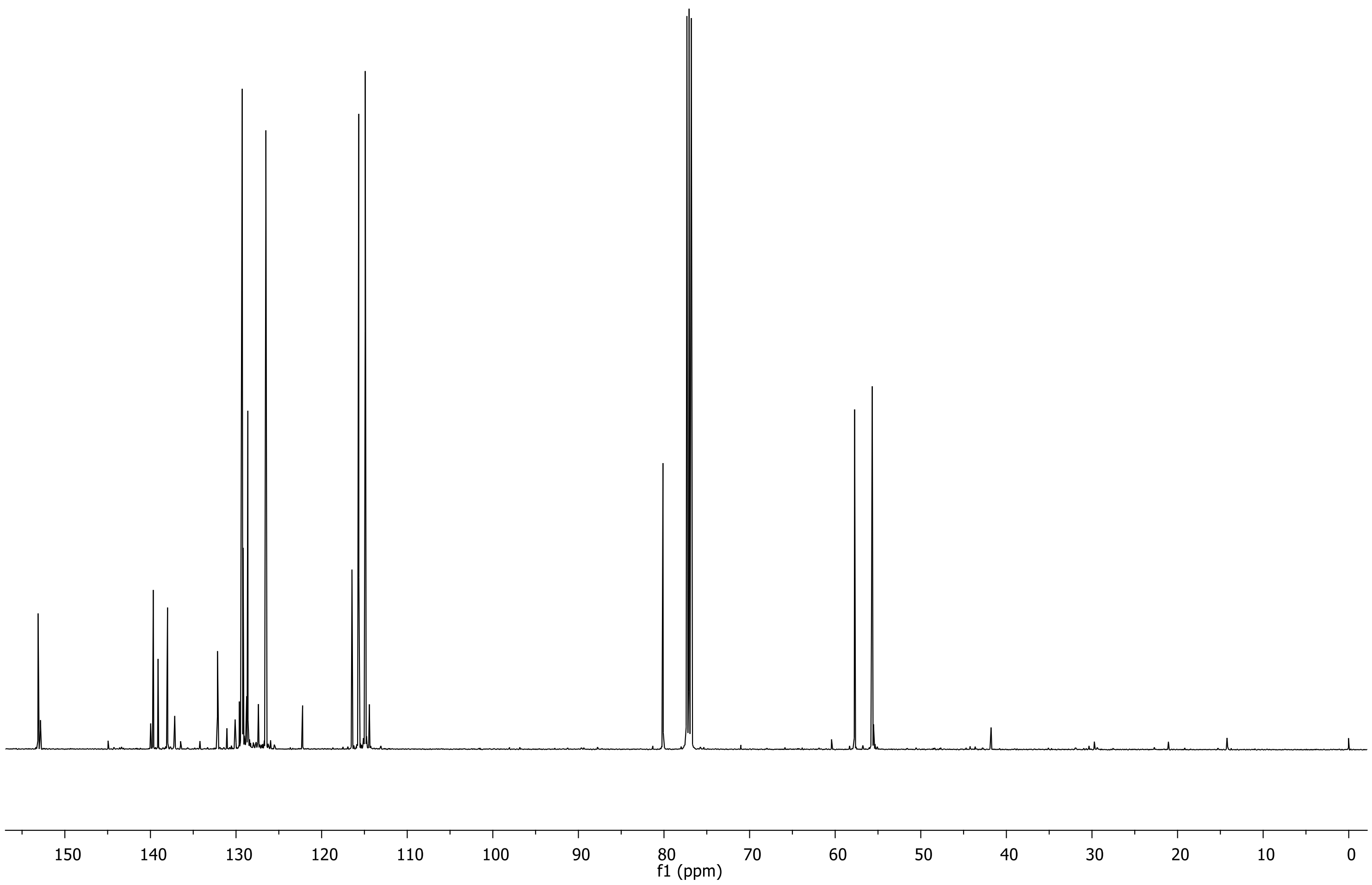

Figure 1.16: ^{13}C NMR spectrum of **7b** *N*-(1-(4-methoxyphenyl)-2-nitroethyl)aniline.

Table 1.3: MS data.

Compound	Formula/Mass		Parent m/z	Cone Voltage	Daughters	Collision Energy	Ion Mode
**7b**	272	1	273.10	22	123.05	14	ES+
		2	273.10	22	108.02	34	ES+
		3	273.10	22	212.14	12	ES+
		4	273.10	22	80.06	62	ES+
		5	273.10	22	104.06	32	ES+

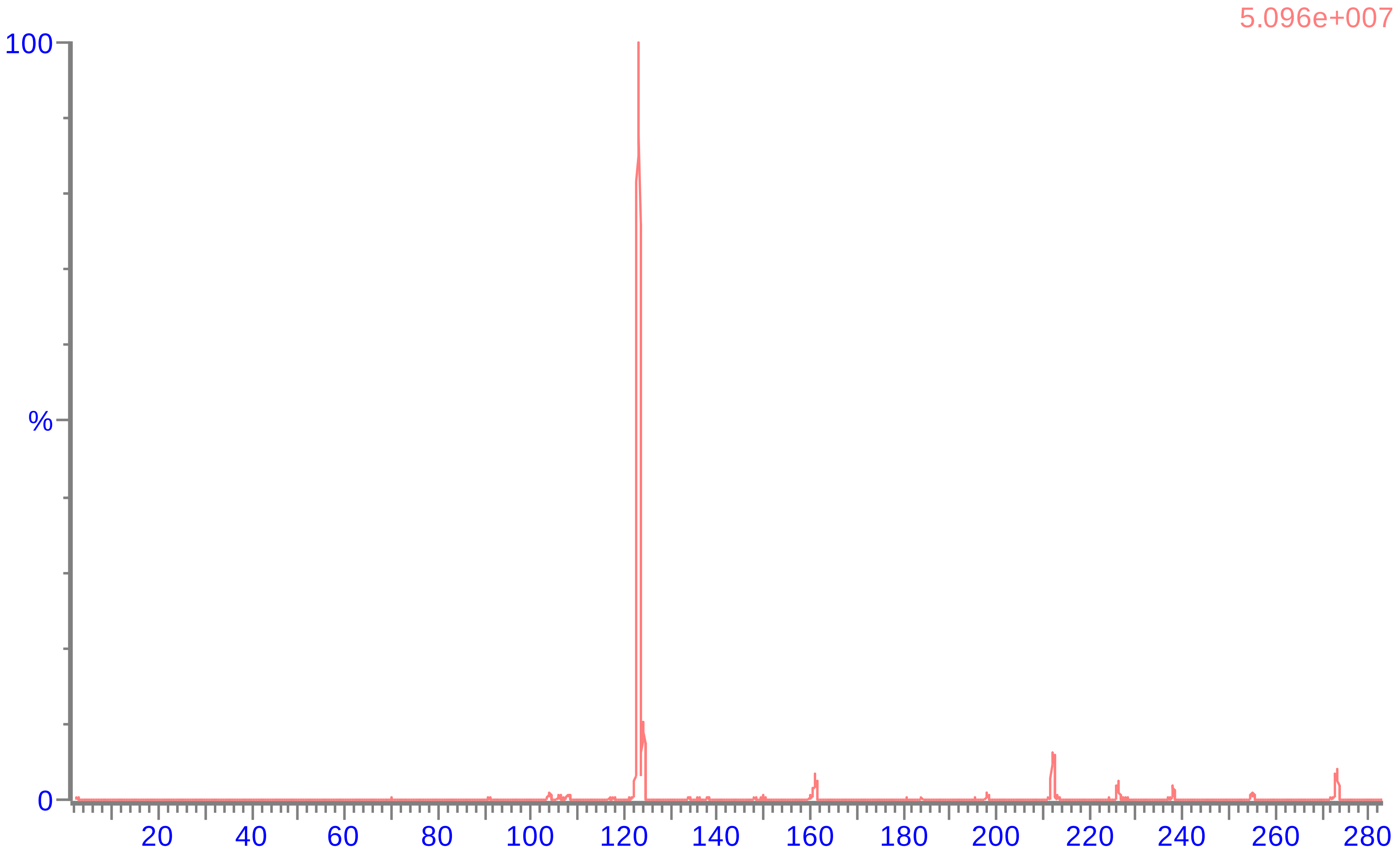

Figure 1.17: Mass spectrum for daughter fragment peak ES+, m/z 273.10 -> 123.05.

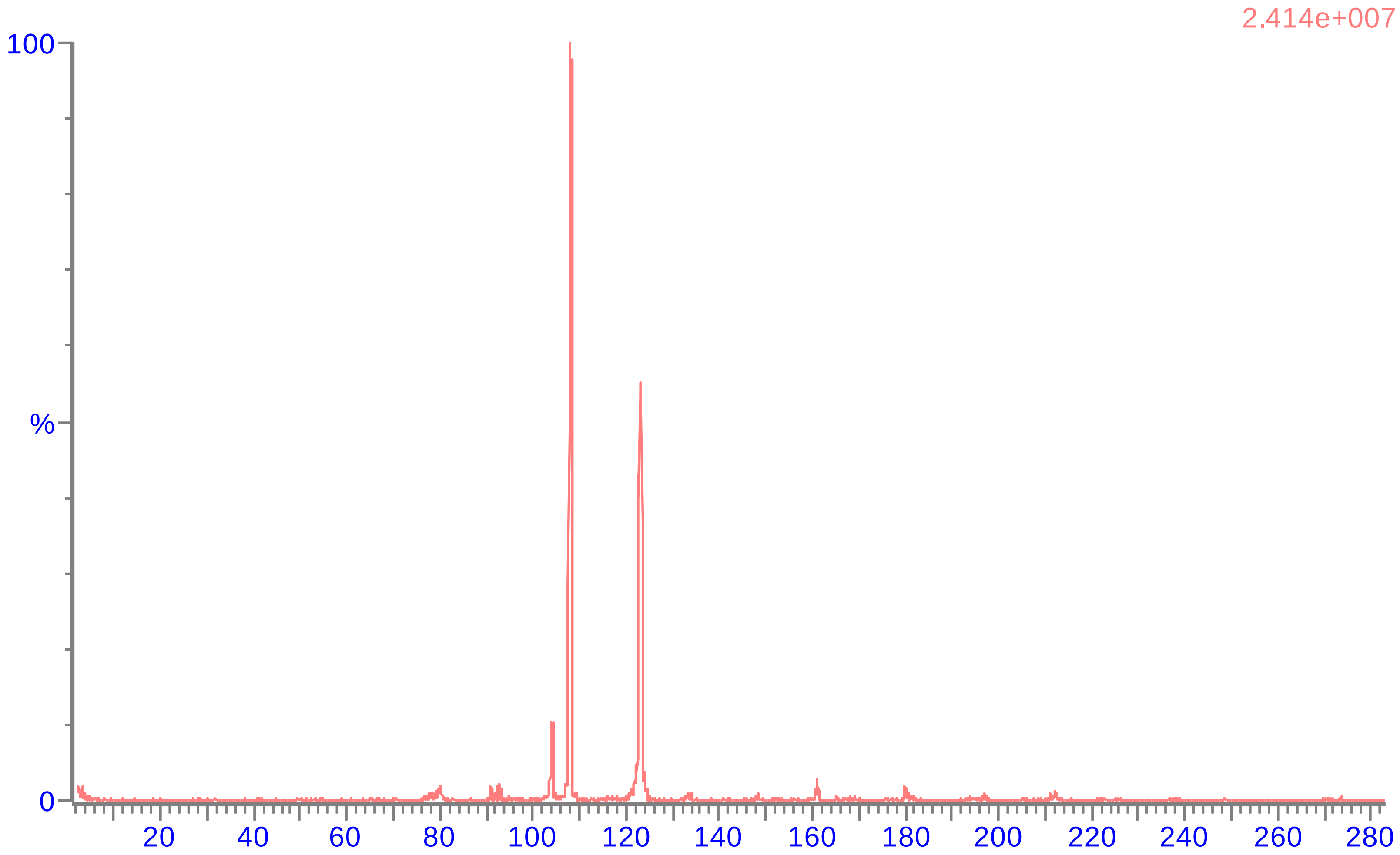

Figure 1.18: Mass spectrum for daughter fragment peak ES+, m/z 273.10 -> 108.02.

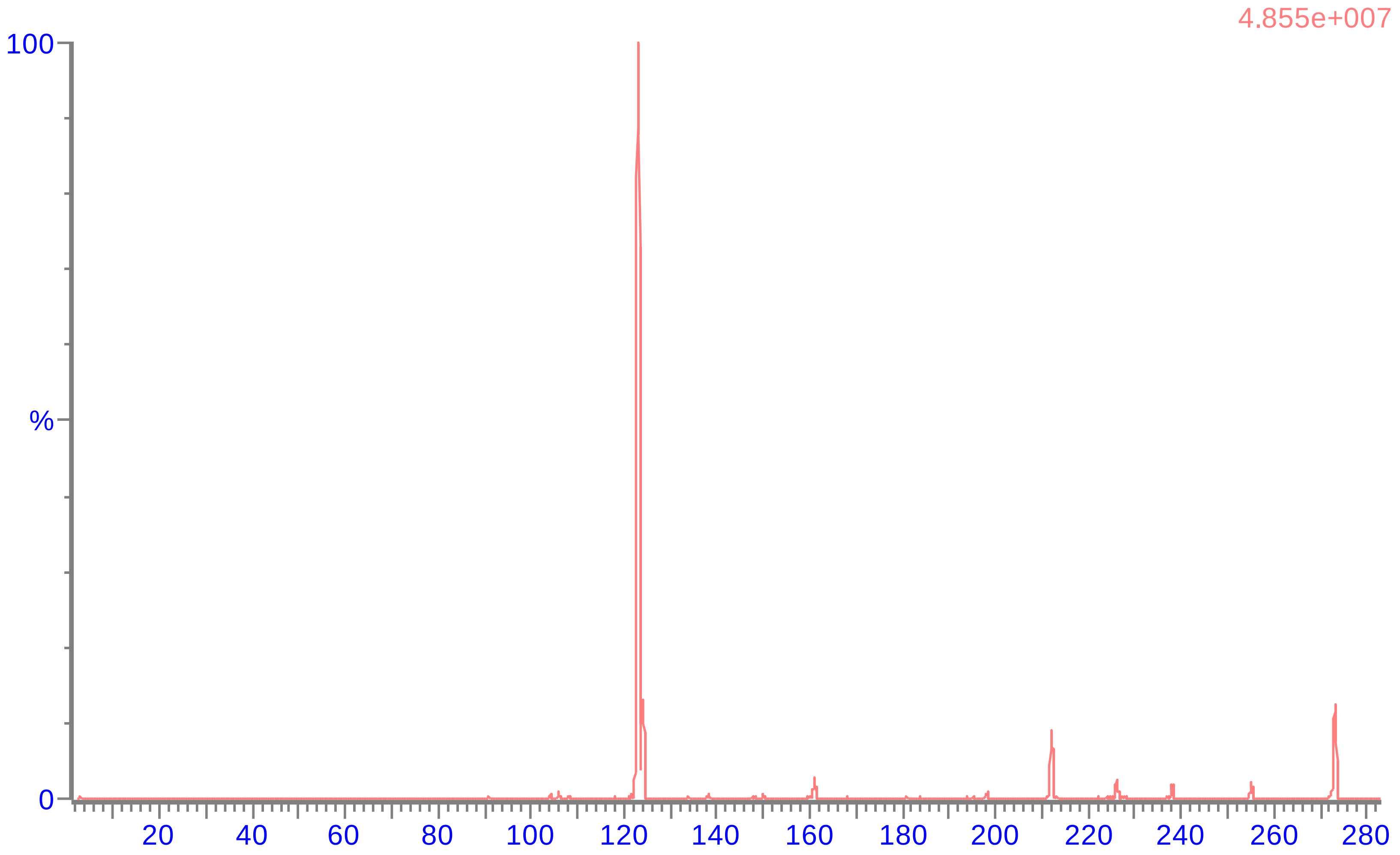

Figure 1.19: Mass spectrum for daughter fragment peak ES+, m/z 273.10 -> 212.14.

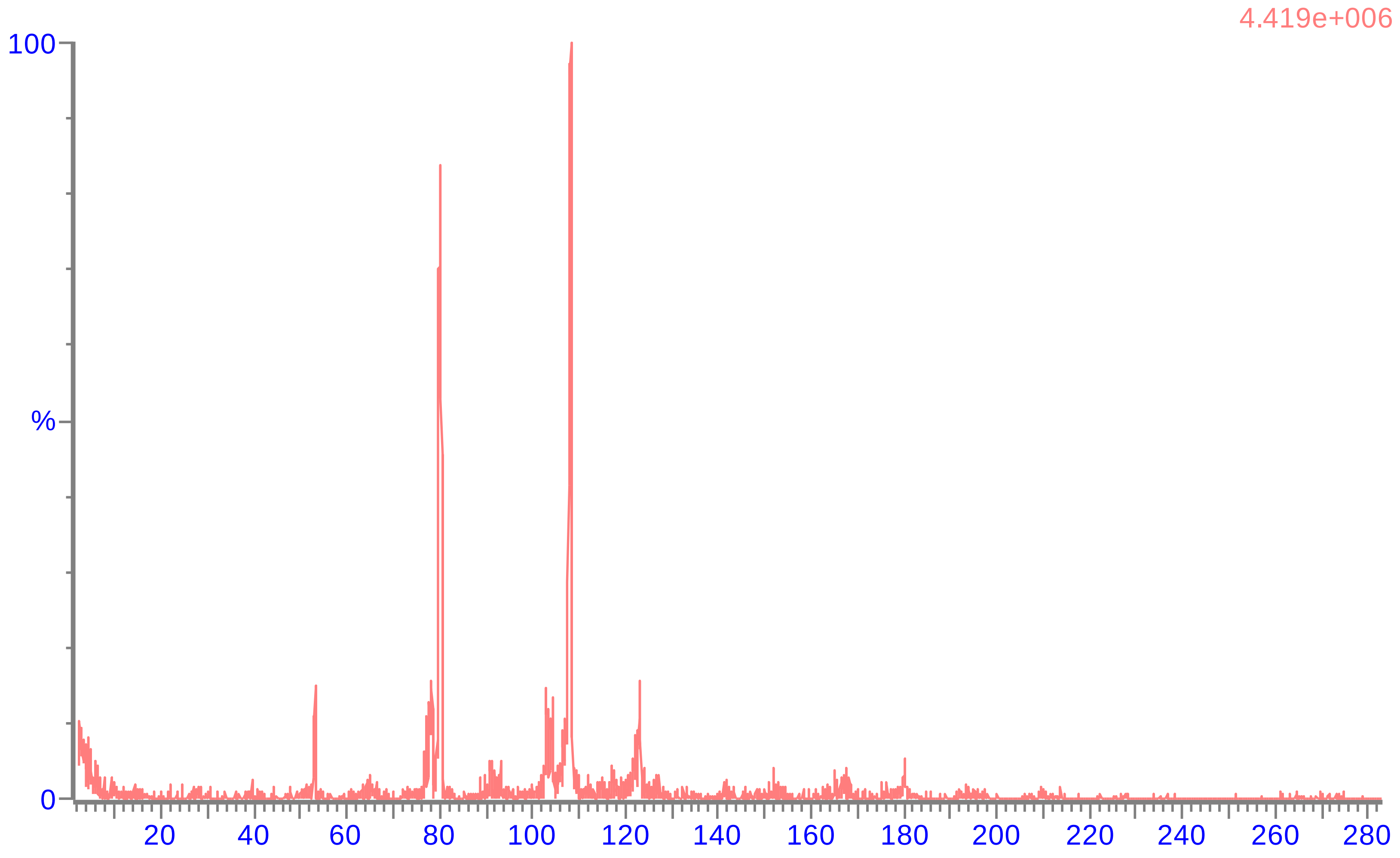

Figure 1.20: Mass spectrum for daughter fragment peak ES+, m/z 273.10 -> 80.06.

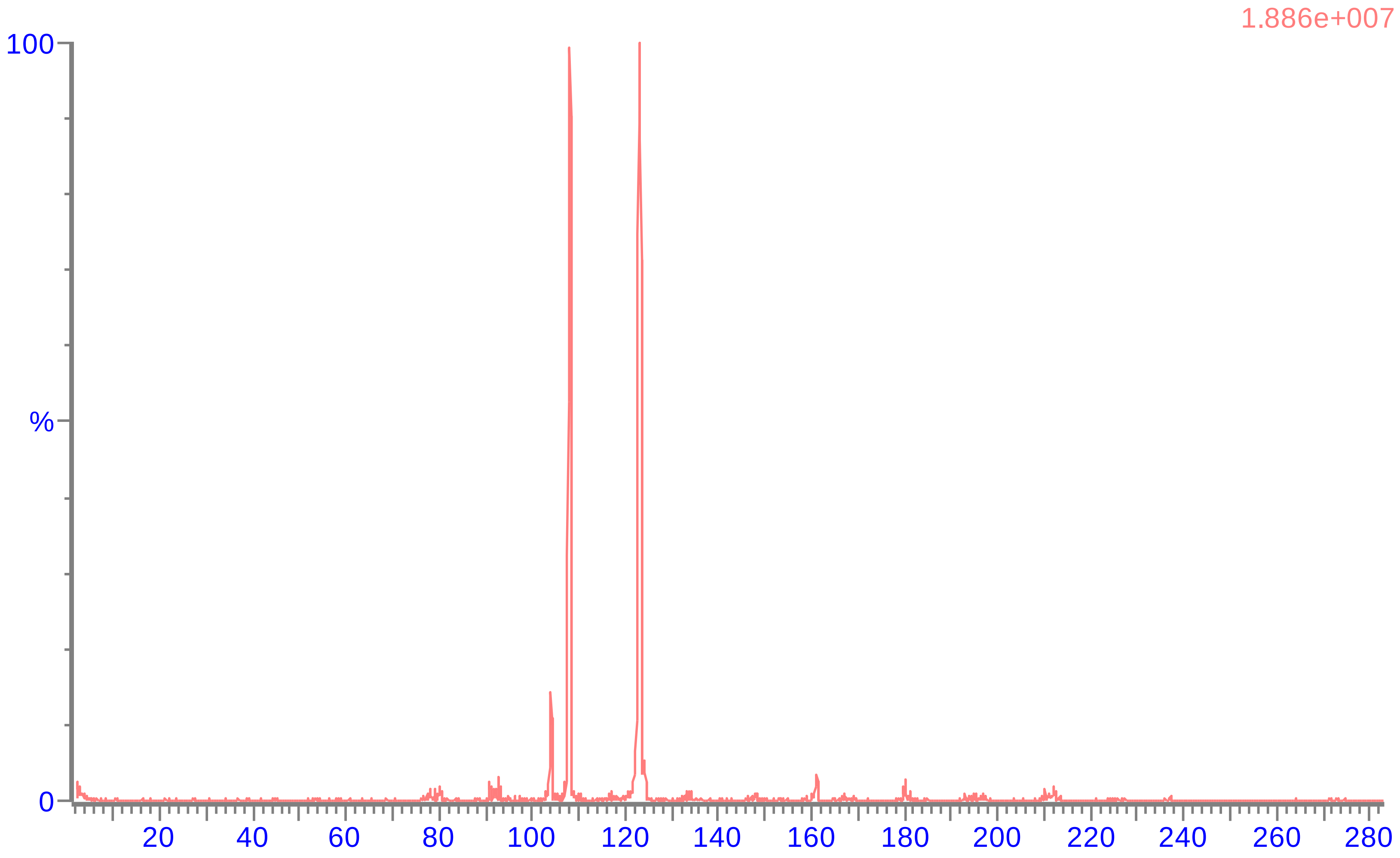

Figure 1.21: Mass spectrum for daughter fragment peak ES+, m/z 273.10 -> 104.06.

7c *N*-(1-(4-bromophenyl)-2-nitroethyl)aniline

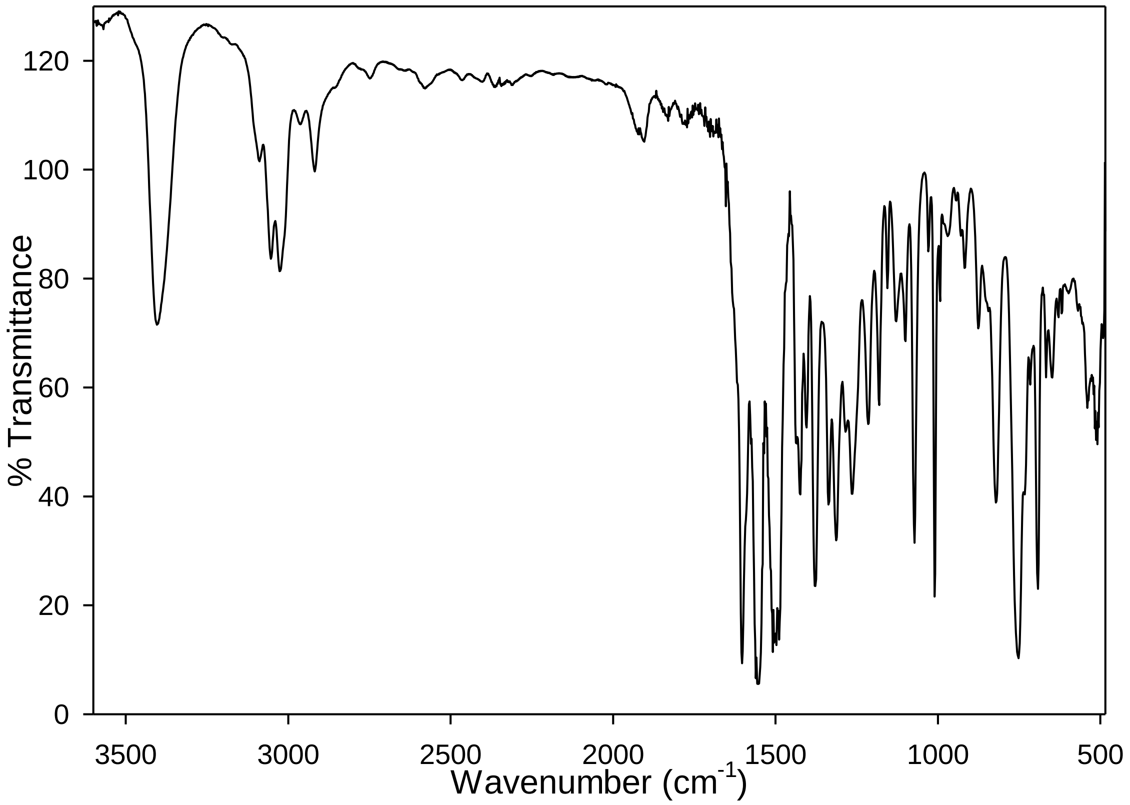

Figure19.22: IR spectrum of **7c** *N*-(1-(4-bromophenyl)-2-nitroethyl)aniline.

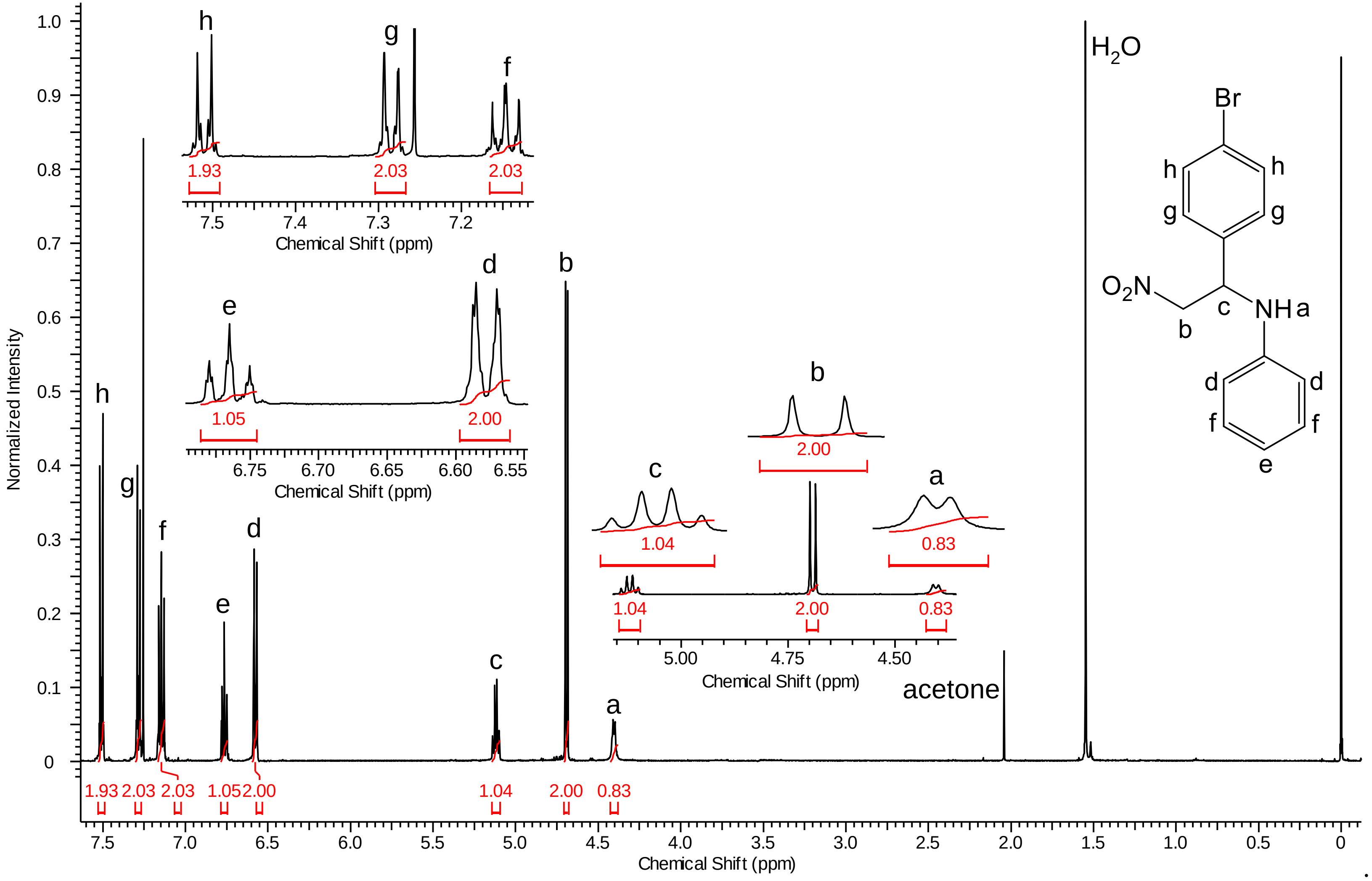

Figure 1.23: ^{1}H NMR spectrum of **7c** *N*-(1-(4-bromophenyl)-2-nitroethyl)aniline.

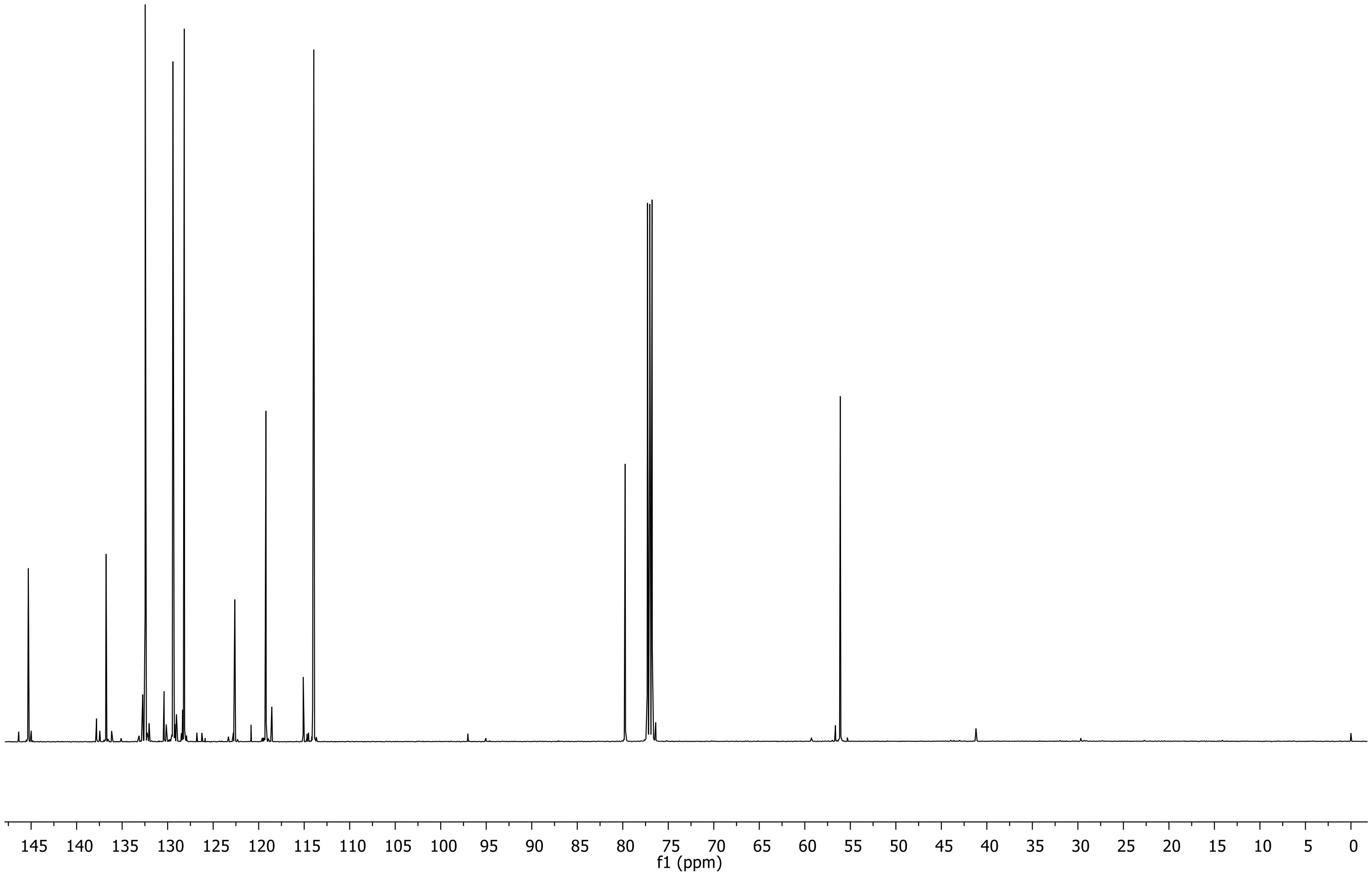

Figure 1.24: ^{13}C NMR spectrum of **7c** *N*-(1-(4-bromophenyl)-2-nitroethyl)aniline.

Table 1.4: MS data.

Compound	Formula/Mass		Parent m/z	Cone Voltage	Daughters	Collision Energy	Ion Mode
**7c**	321.5	1	323.04	18	93.98	14	ES+
		2	323.04	18	183.99	28	ES+
		3	323.04	18	262.04	16	ES+
		4	323.04	18	103.02	50	ES+
		5	323.04	18	77.01	62	ES+

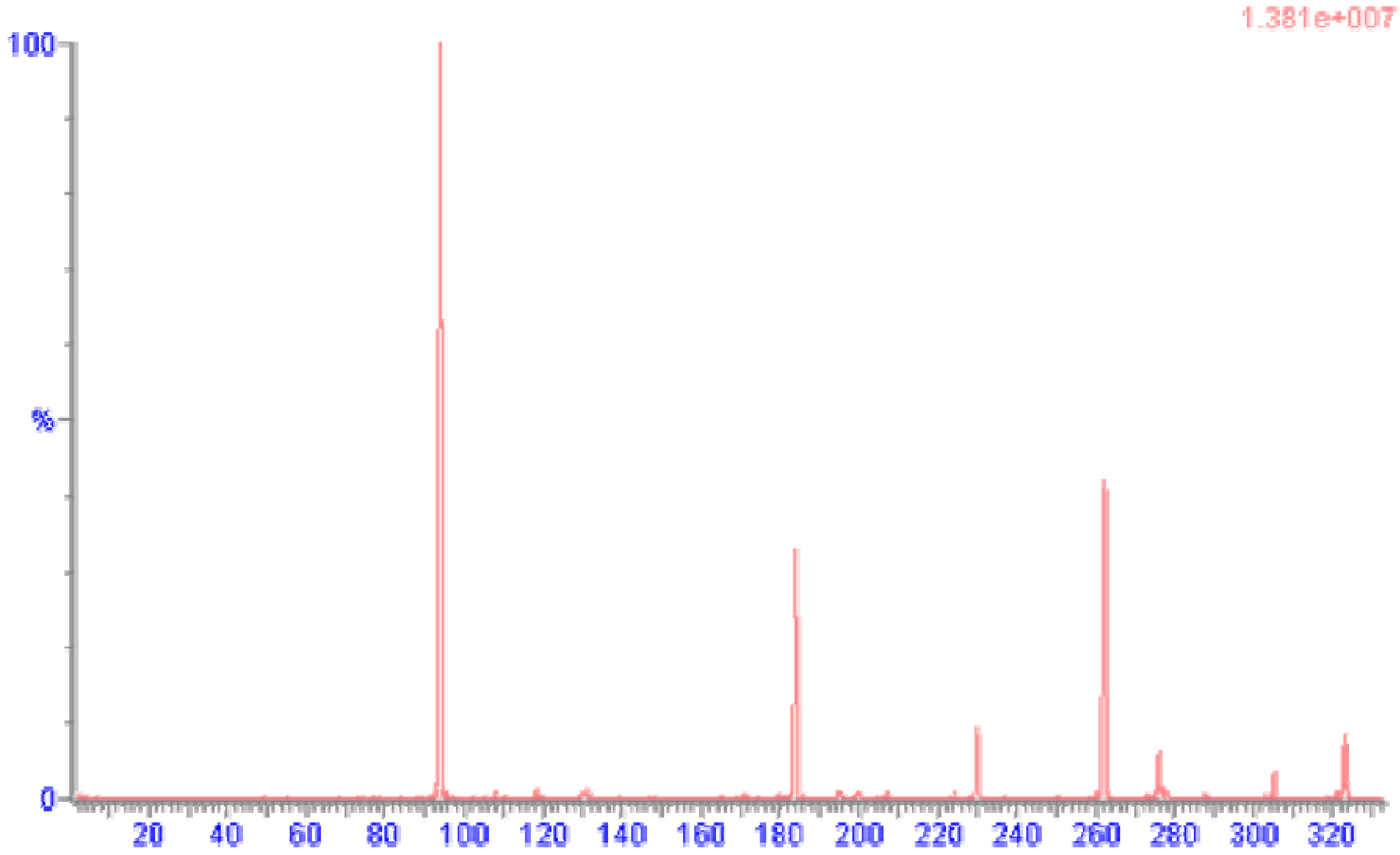

Figure 1.25: Mass spectrum for daughter fragment peak ES+, m/z 323.04 -> 93.98.

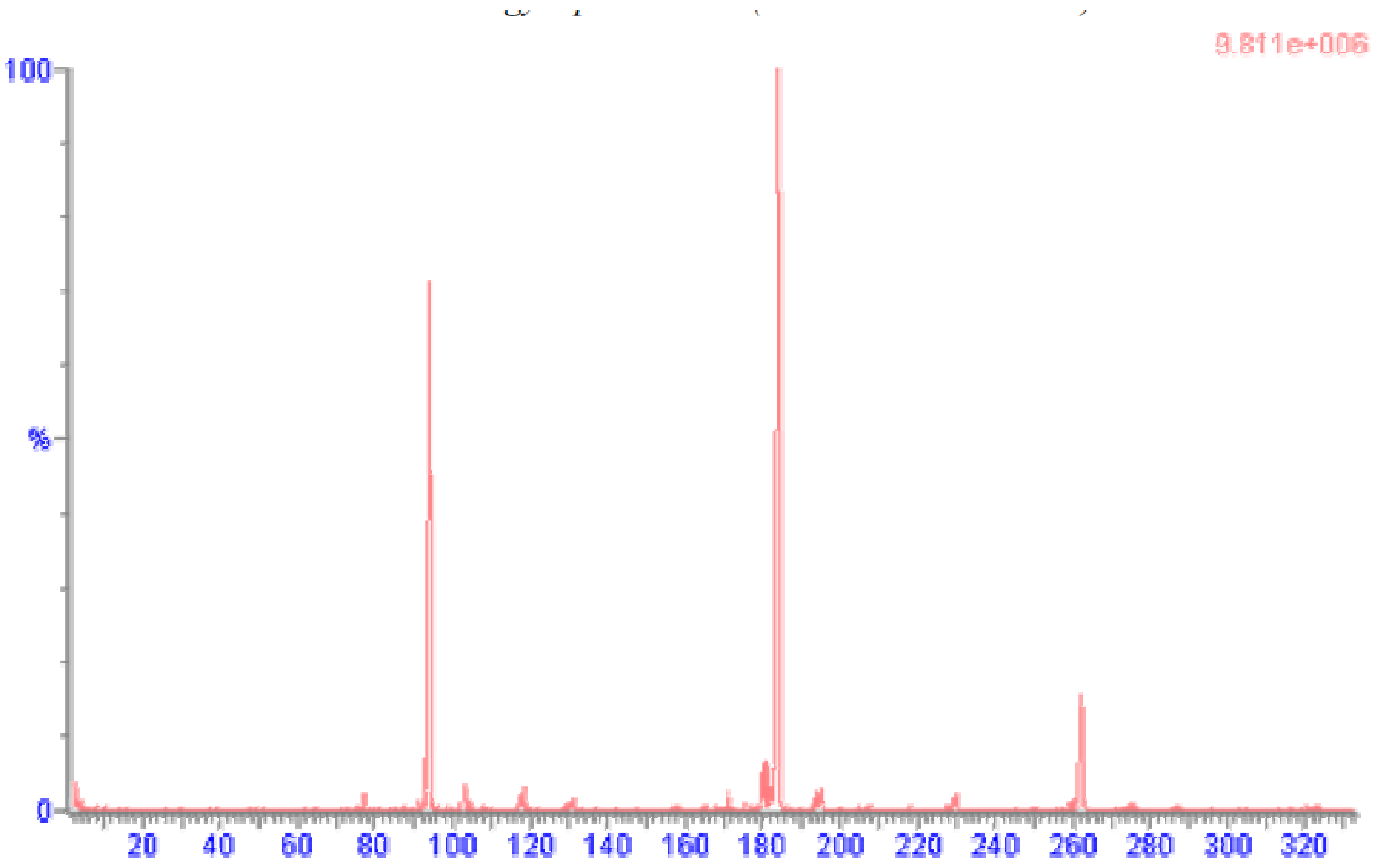

Figure 1.26: Mass spectrum for daughter fragment peak ES+, m/z 323.04 -> 183.99.

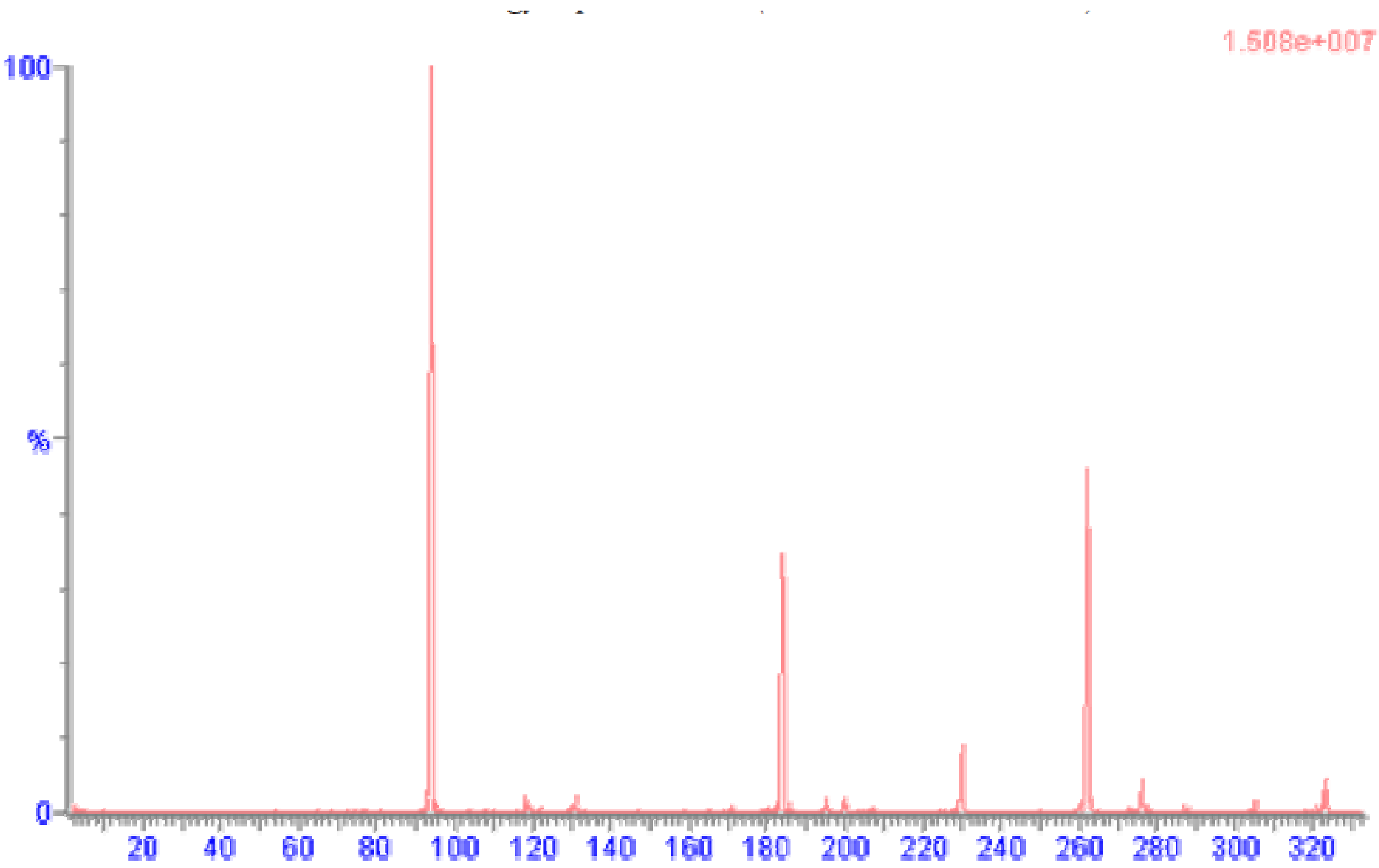

Figure 1.27: Mass spectrum for daughter fragment peak ES+, m/z 323.04 -> 262.04.

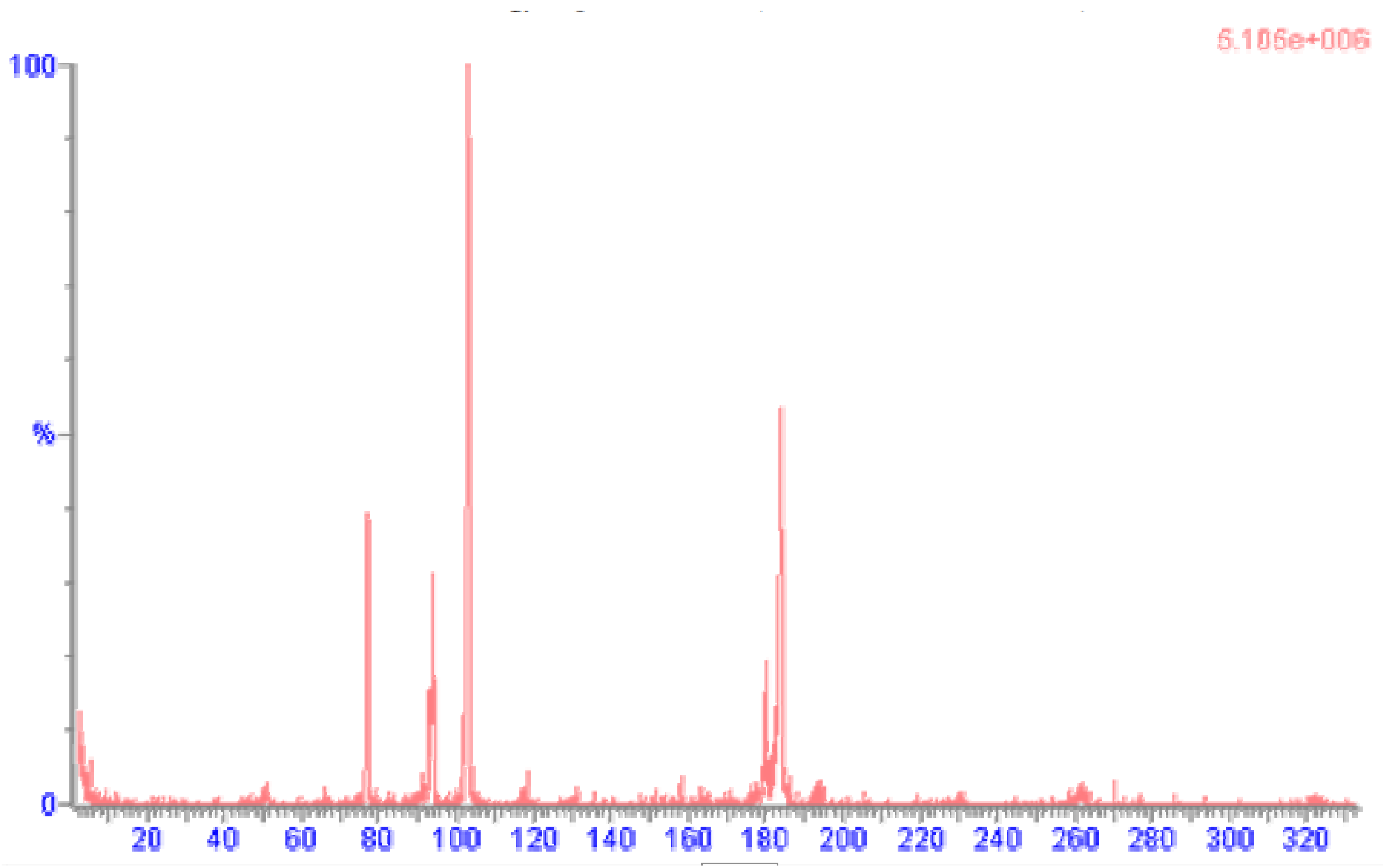

Figure 1.28: Mass spectrum for daughter fragment peak ES+, m/z 323.04 -> 103.02.

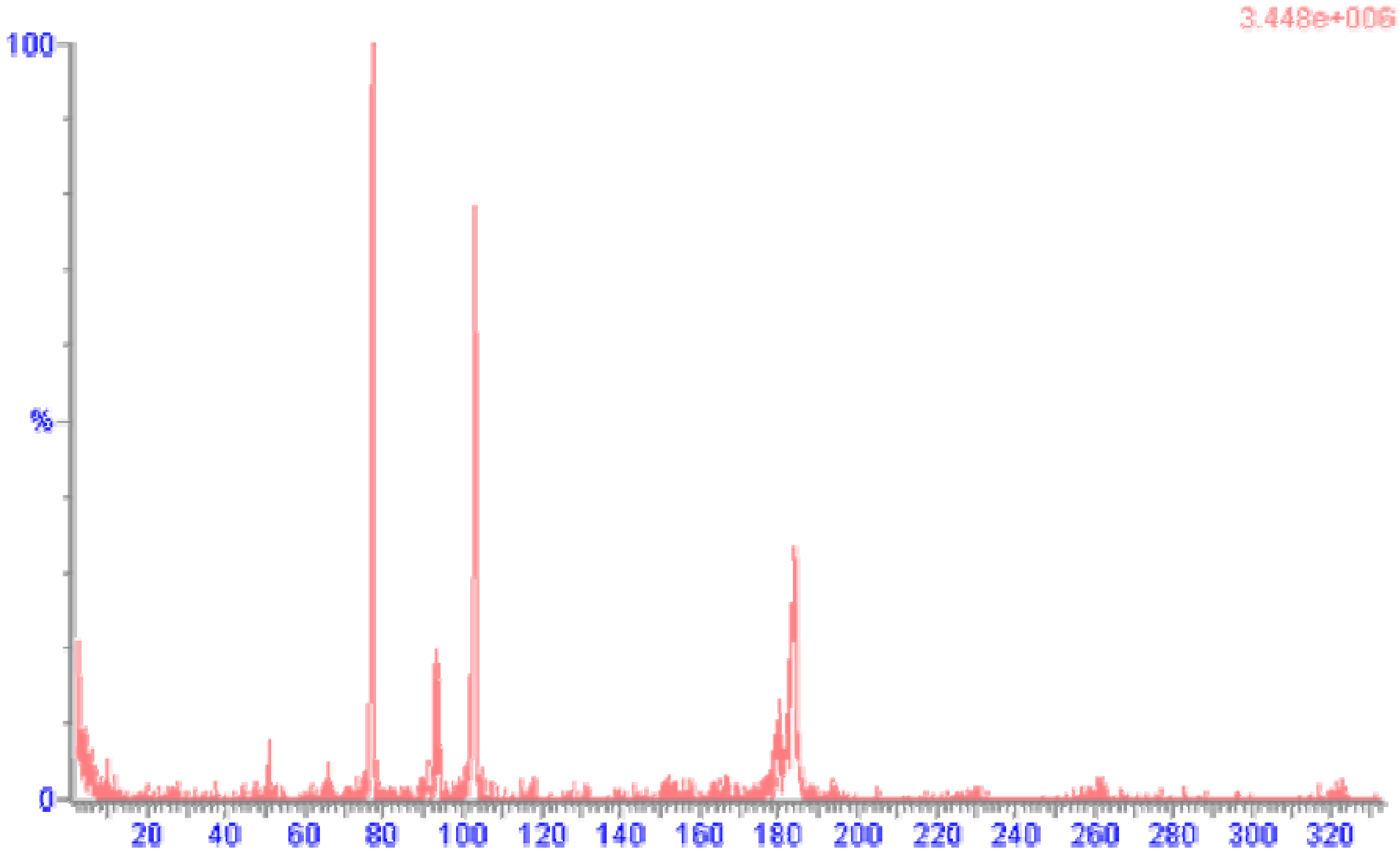

Figure 1.29: Mass spectrum for daughter fragment peak ES+, m/z 323.04 -> 77.01.

<u>122d</u> <u>*N*-(2-nitro-1-(2-nitrophenyl)ethyl)aniline</u>

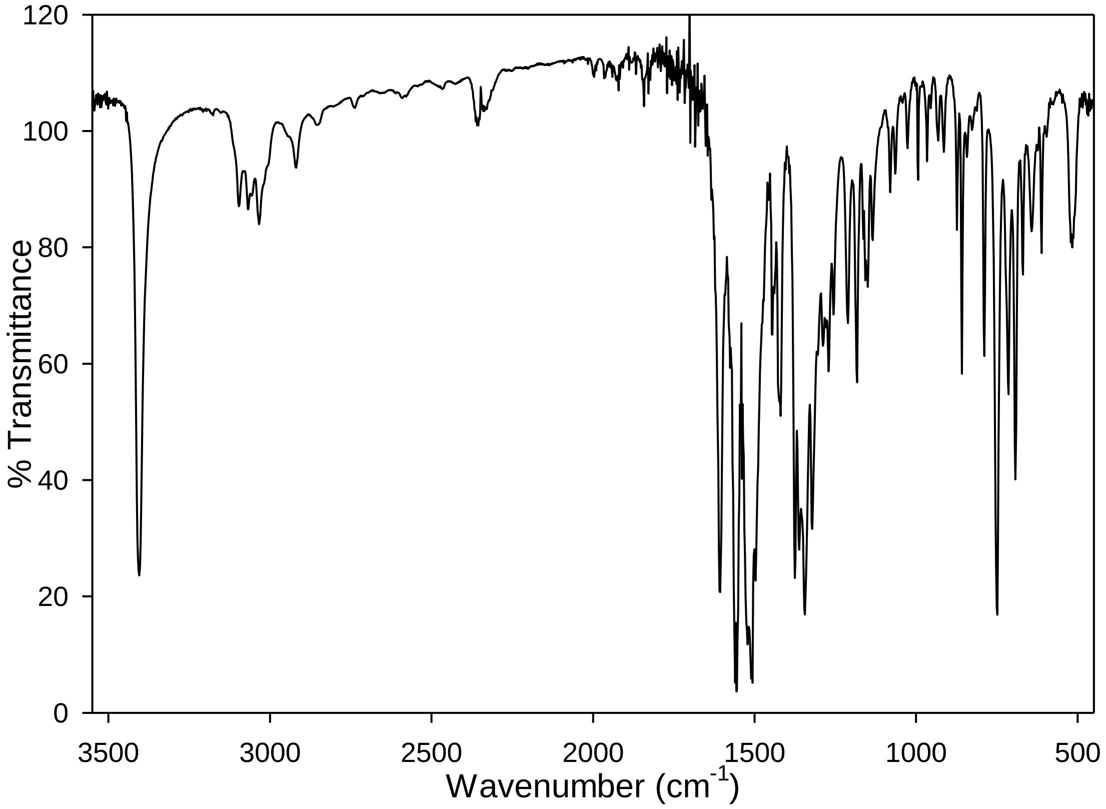

Figure 1.30: IR spectrum of **7d** *N*-(2-nitro-1-(2-nitrophenyl)ethyl)aniline.

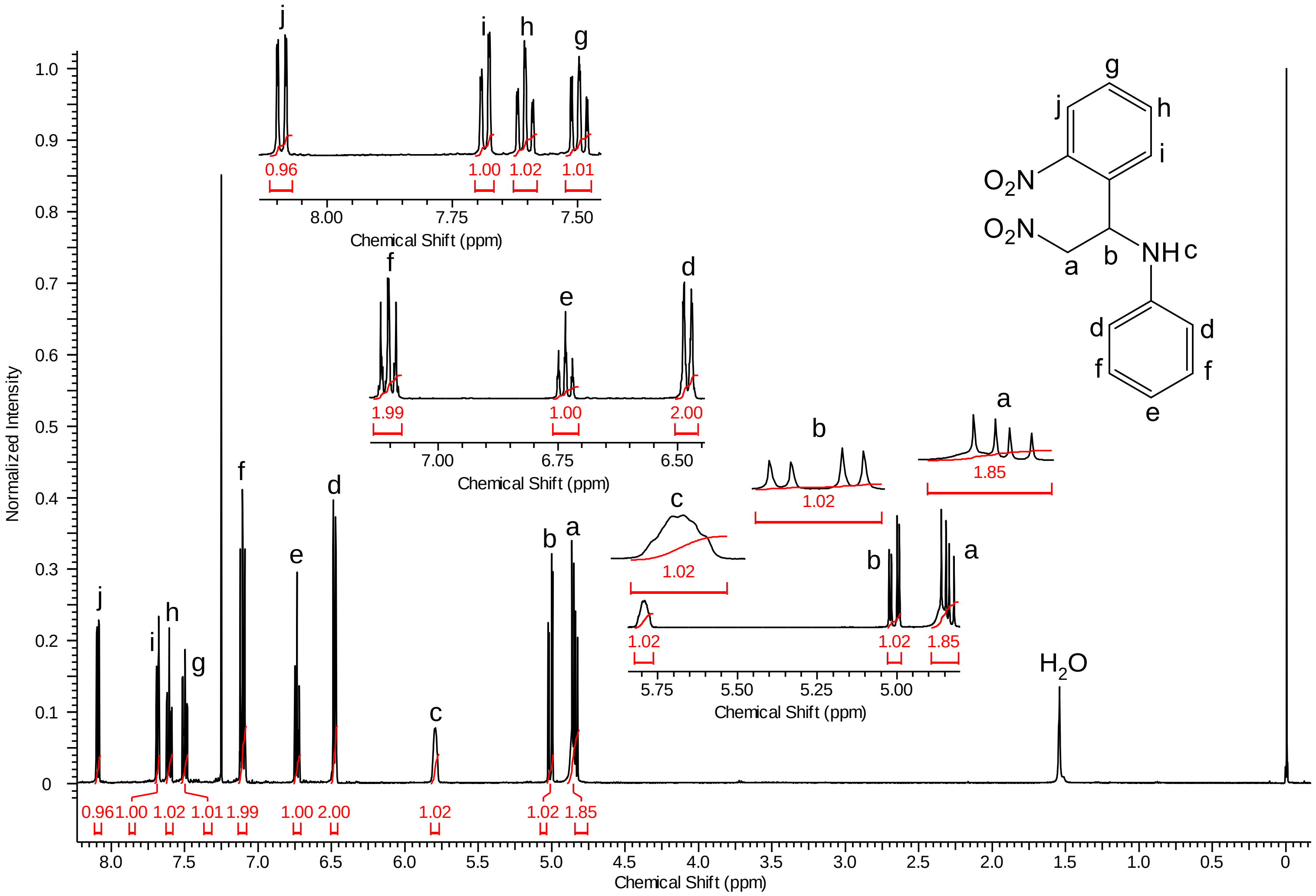

Figure 1.31: ^{1}H NMR spectrum of **7d** *N*-(2-nitro-1-(2-nitrophenyl)ethyl)aniline.

7e 3-(2-nitro-1-(phenylamino)ethyl)phenol

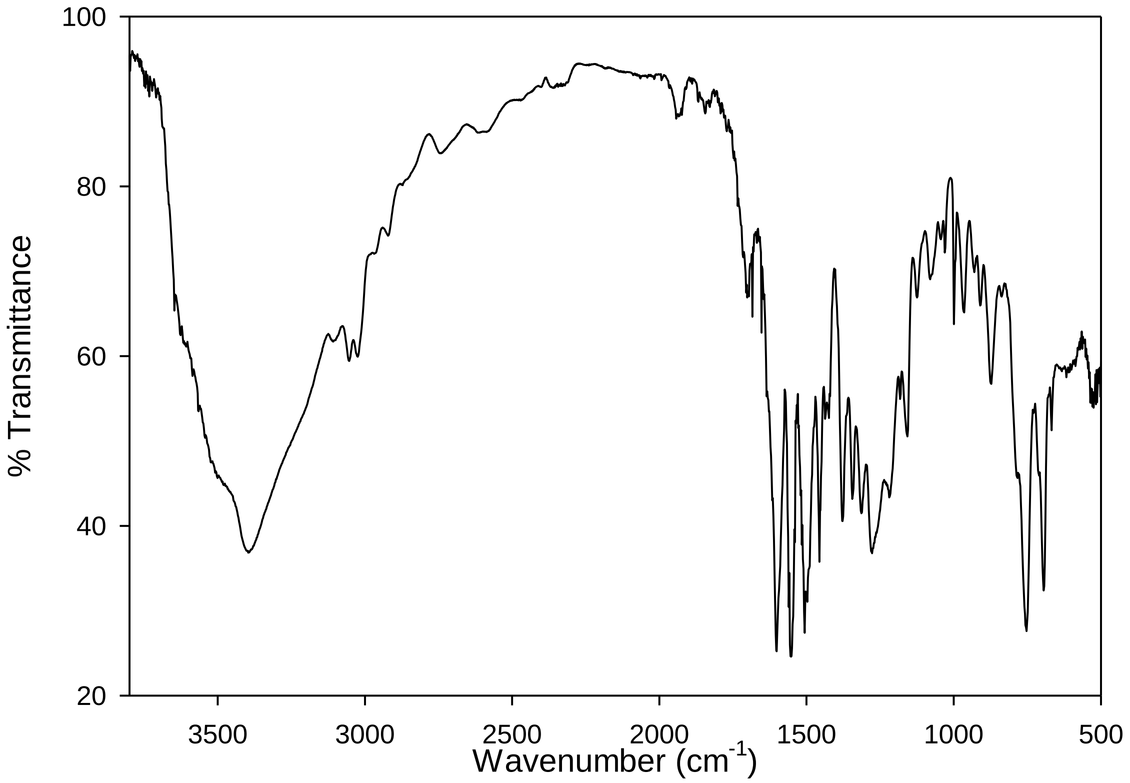

Figure 1.32: IR spectrum of **7e** 3-(2-nitro-1-(phenylamino)ethyl)phenol.

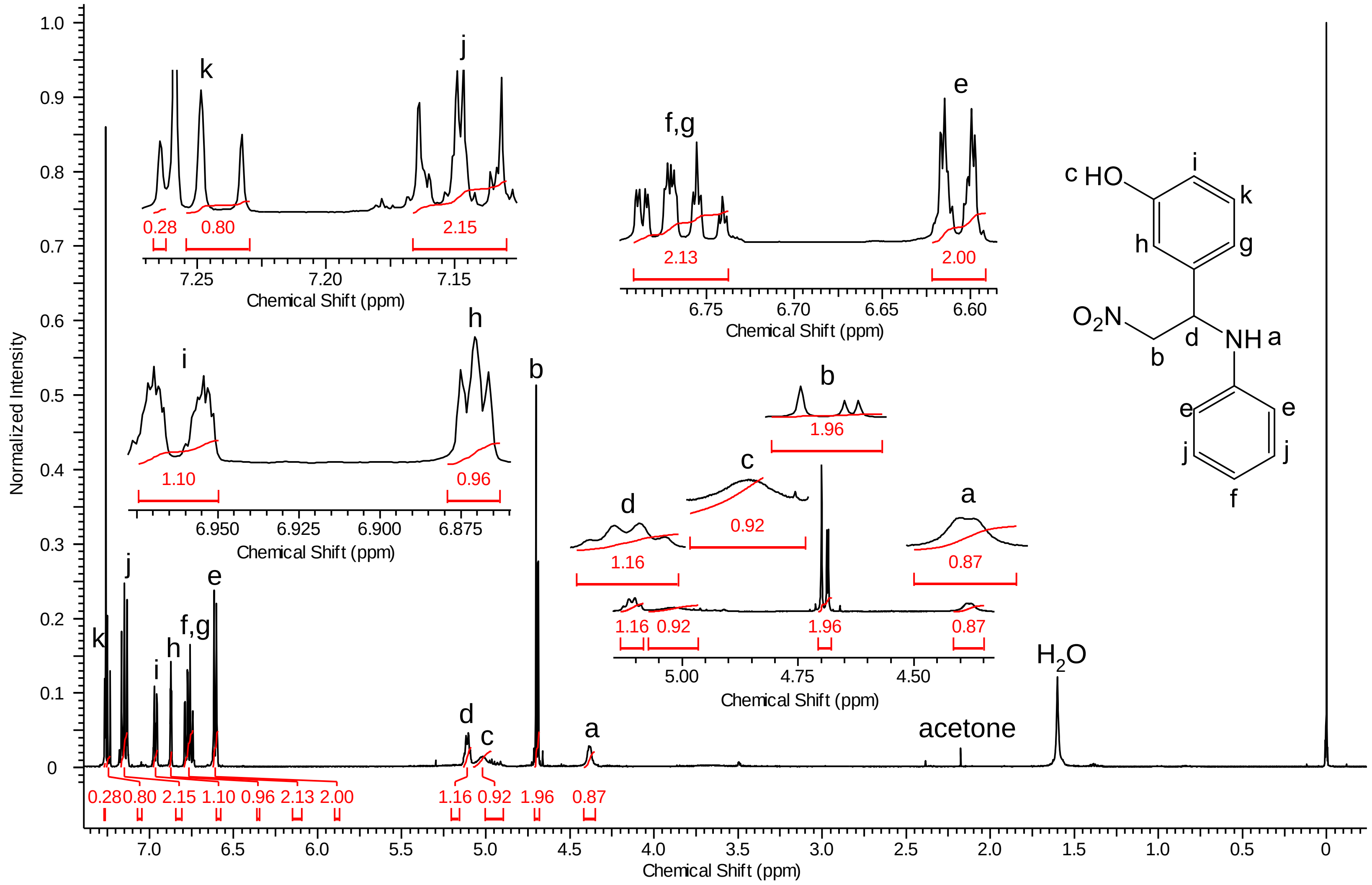

Figure 1.33: ^{1}H NMR spectrum of **7e** 3-(2-nitro-1-(phenylamino)ethyl)phenol.

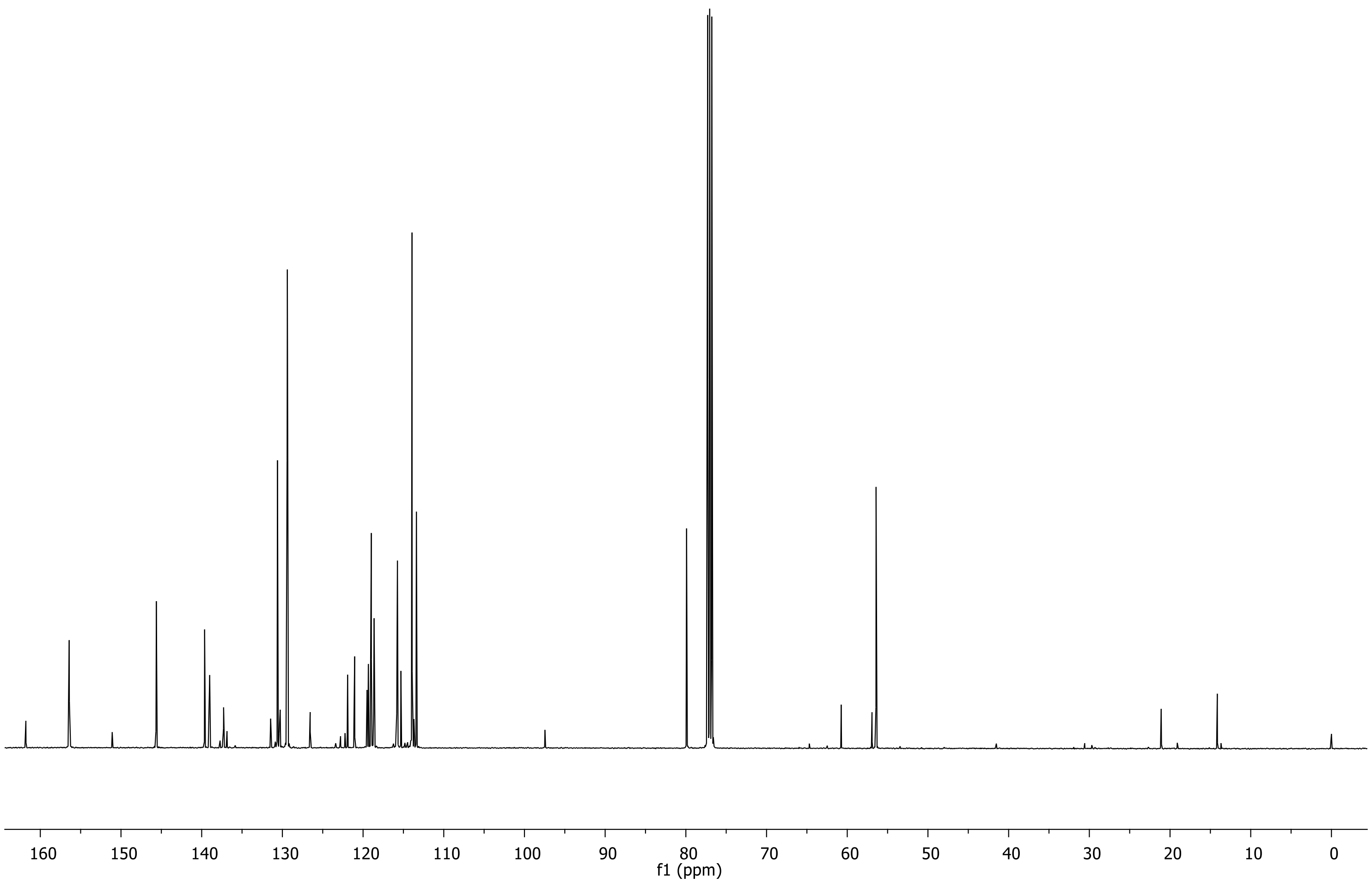

Figure 1.34: ^{13}C NMR spectrum of **7e** 3-(2-nitro-1-(phenylamino)ethyl)phenol.

Table 1.5: MS data.

**Compound**	**Formula/Mass**		**Parent m/z**	**Cone Voltage**	**Daughters**	**Collision Energy**	**Ion Mode**
**7e**	258	1	259.10	22	120.07	18	ES+
		2	259.10	22	94.05	14	ES+
		3	259.10	22	198.06	12	ES+
		4	259.10	22	212.17	12	ES+
		5	259.10	22	166.06	10	ES+

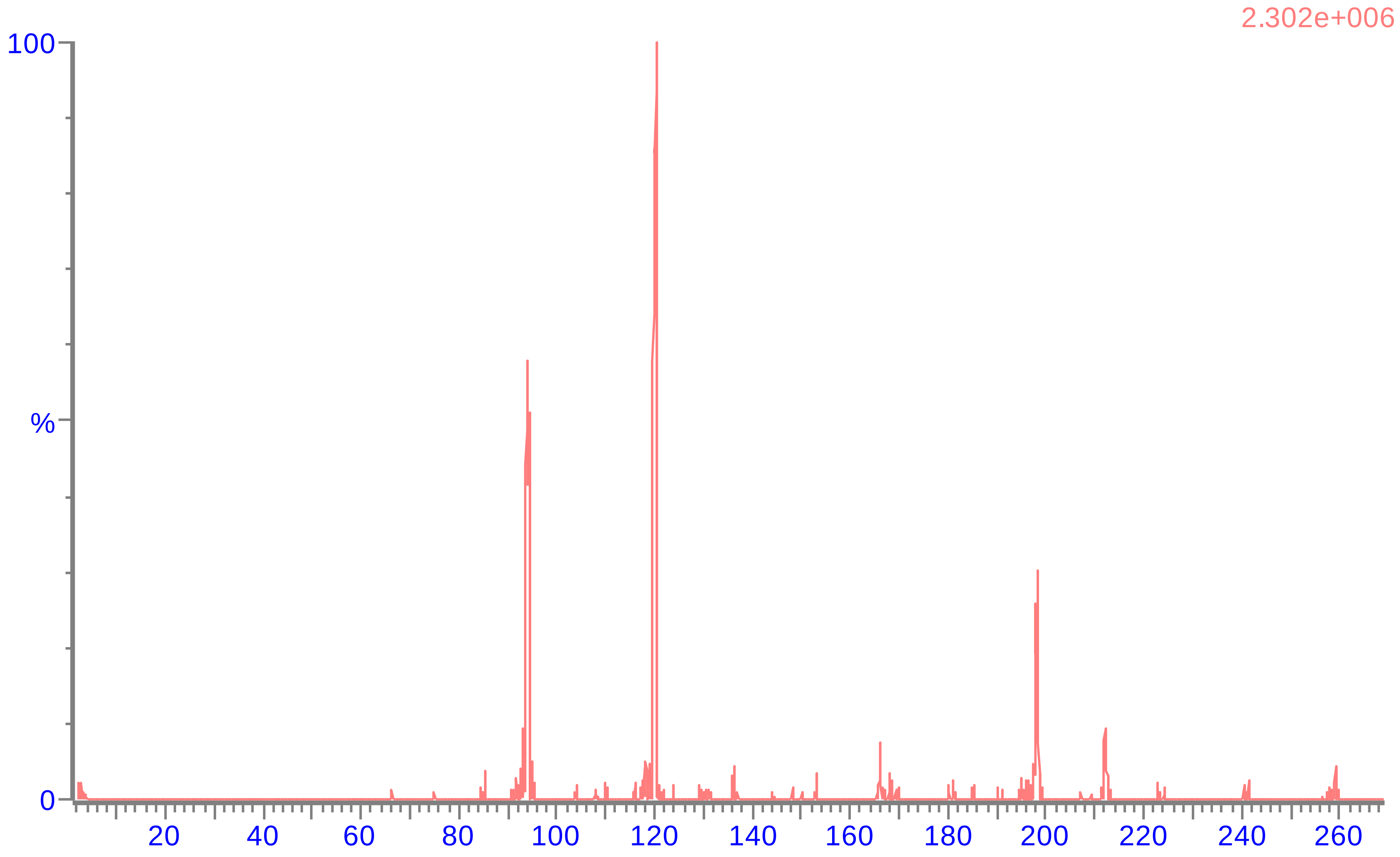

Figure 1.35: Mass spectrum for daughter fragment peak ES+, m/z 259.10 -> 120.07.

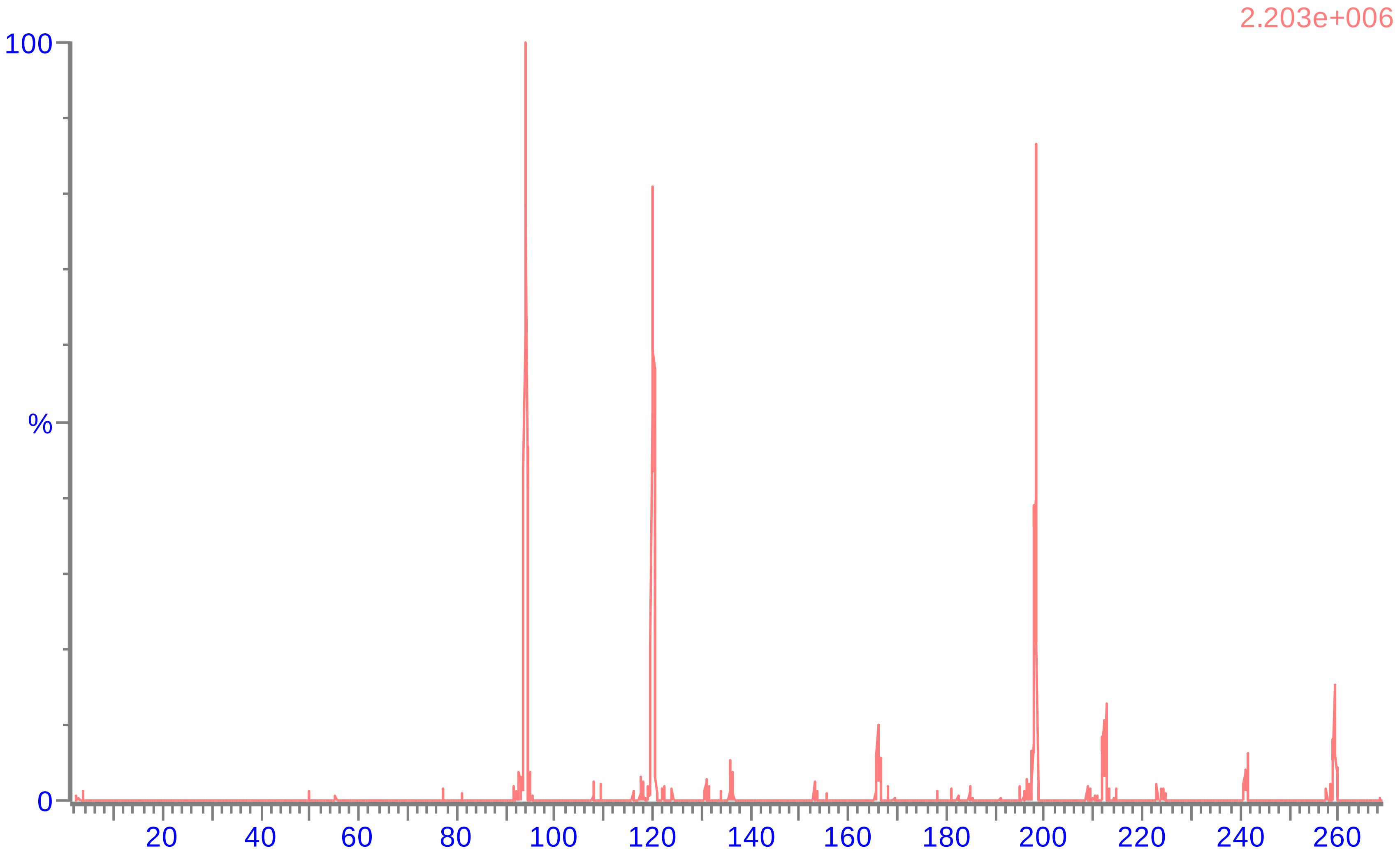

Figure 1.36: Mass spectrum for daughter fragment peak ES+, m/z 259.10 -> 94.05.

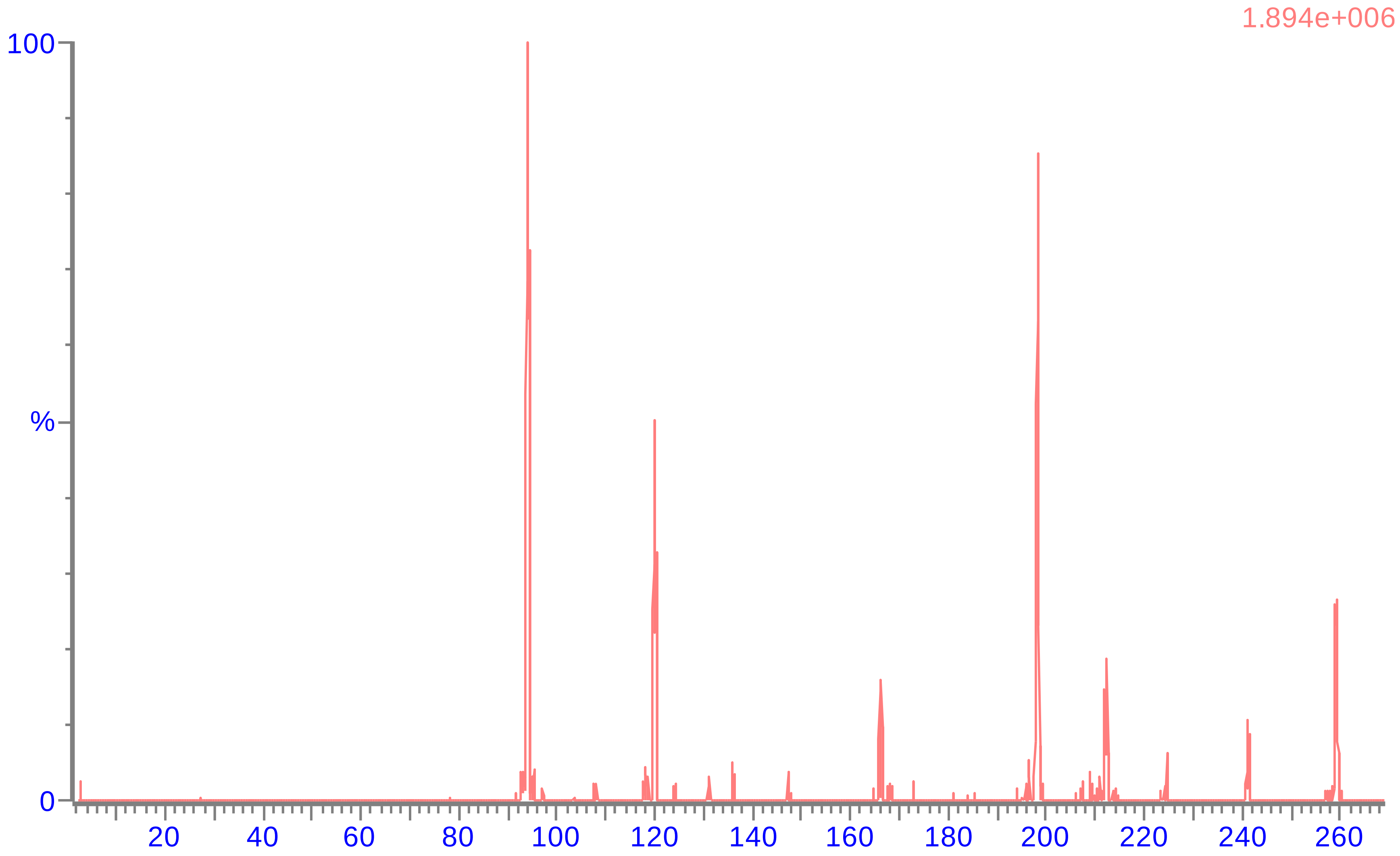

Figure 1.37: Mass spectrum for daughter fragment peak ES+, m/z 259.10 -> 198.06.

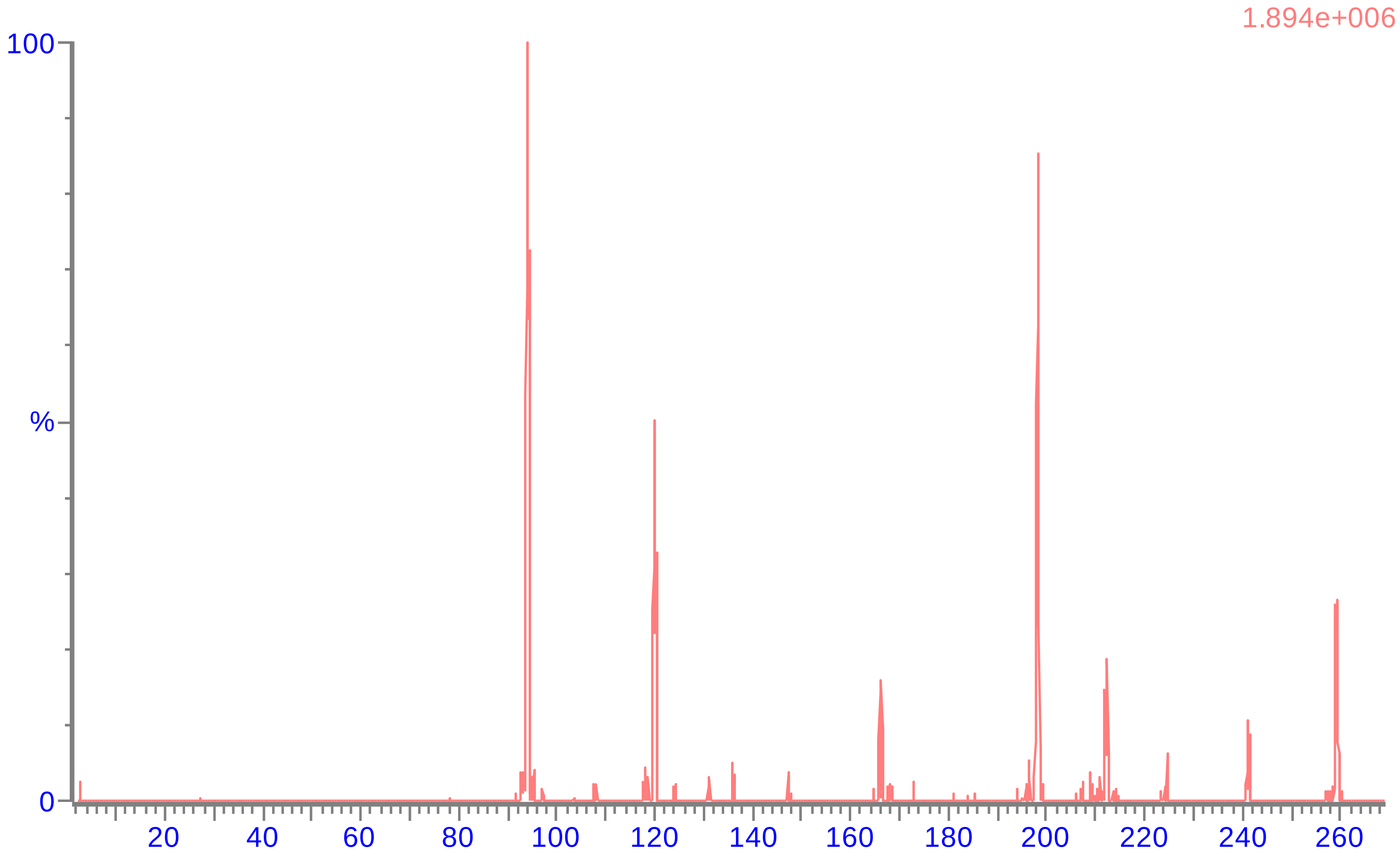

Figure 1.38: Mass spectrum for daughter fragment peak ES+, m/z 259.10 -> 212.17.

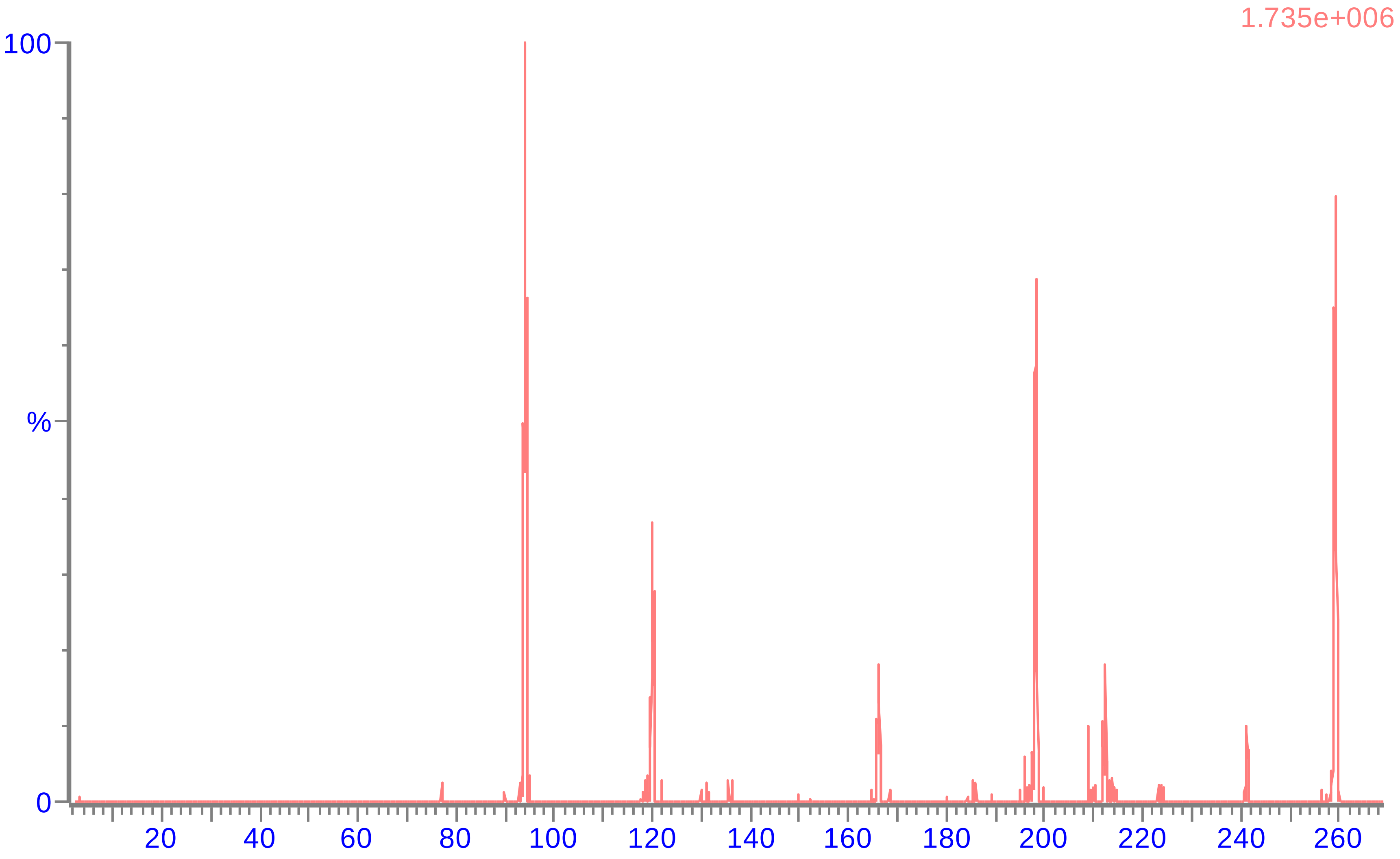

Figure 1.39: Mass spectrum for daughter fragment peak ES+, m/z 259.10 -> 166.06.

7f *N*-(1-(naphthalene-1-yl)-2-nitroethyl)aniline

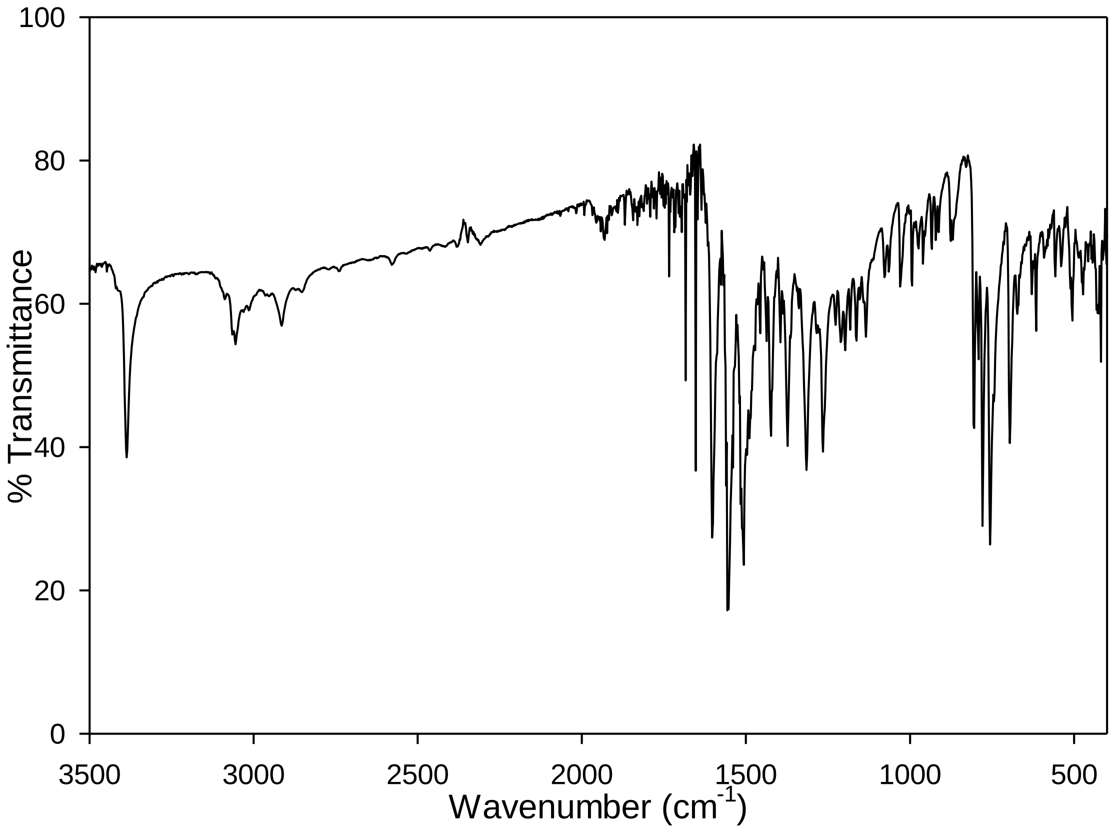

Figure 1.40: IR spectrum of **7f** *N*-(1-(naphthalene-1-yl)-2-nitroethyl)aniline.

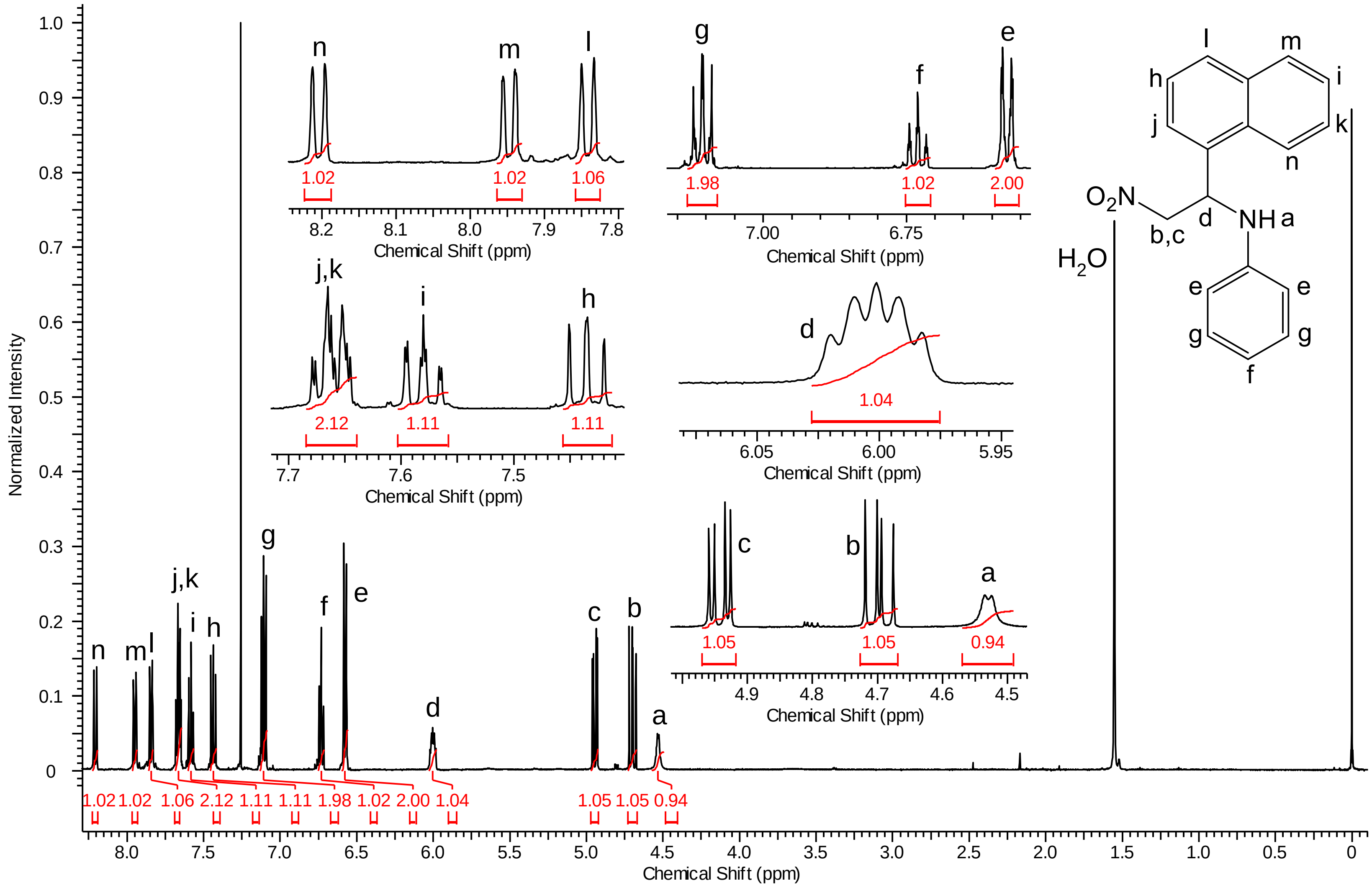

Figure 1.41: ^{1}H NMR spectrum of **7f** *N*-(1-(naphthalene-1-yl)-2-nitroethyl)aniline.

Table 1.6: MS data.

Compound	Formula/Mass		Parent m/z	Cone Voltage	Daughters	Collision Energy	Ion Mode
**7f**	292	1	293.10	16	154.02	14	ES+
		2	293.10	16	93.99	8	ES+
		3	293.10	16	200.07	8	ES+
		4	291.10	16	198.07	8	ES-
		5	293.10	16	119.03	16	ES+

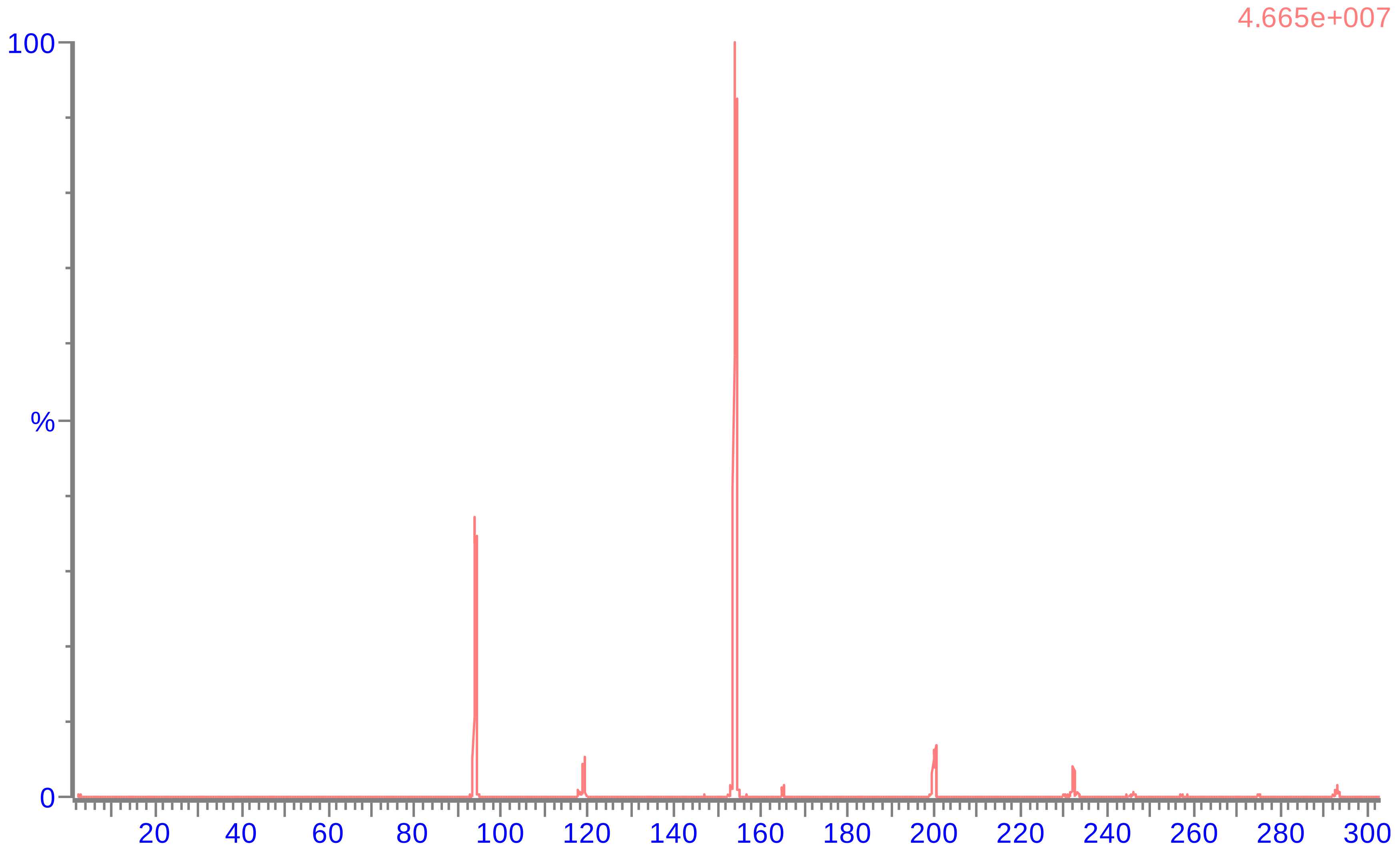

Figure 1.42: Mass spectrum for daughter fragment peak ES+, m/z 293.10 -> 154.02.

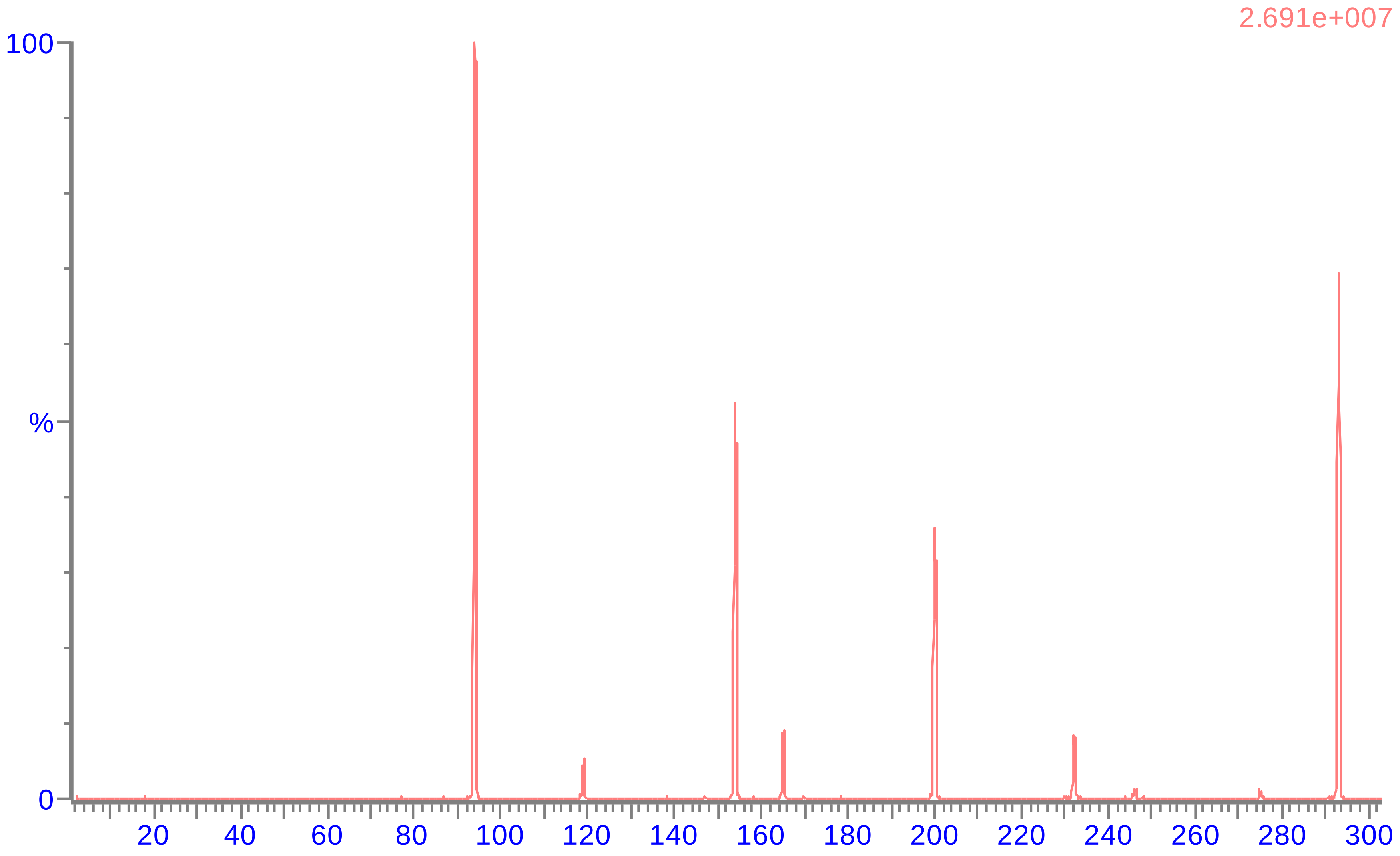

Figure 1.43: Mass spectrum for daughter fragment peak ES+, m/z 293.10 -> 93.99.

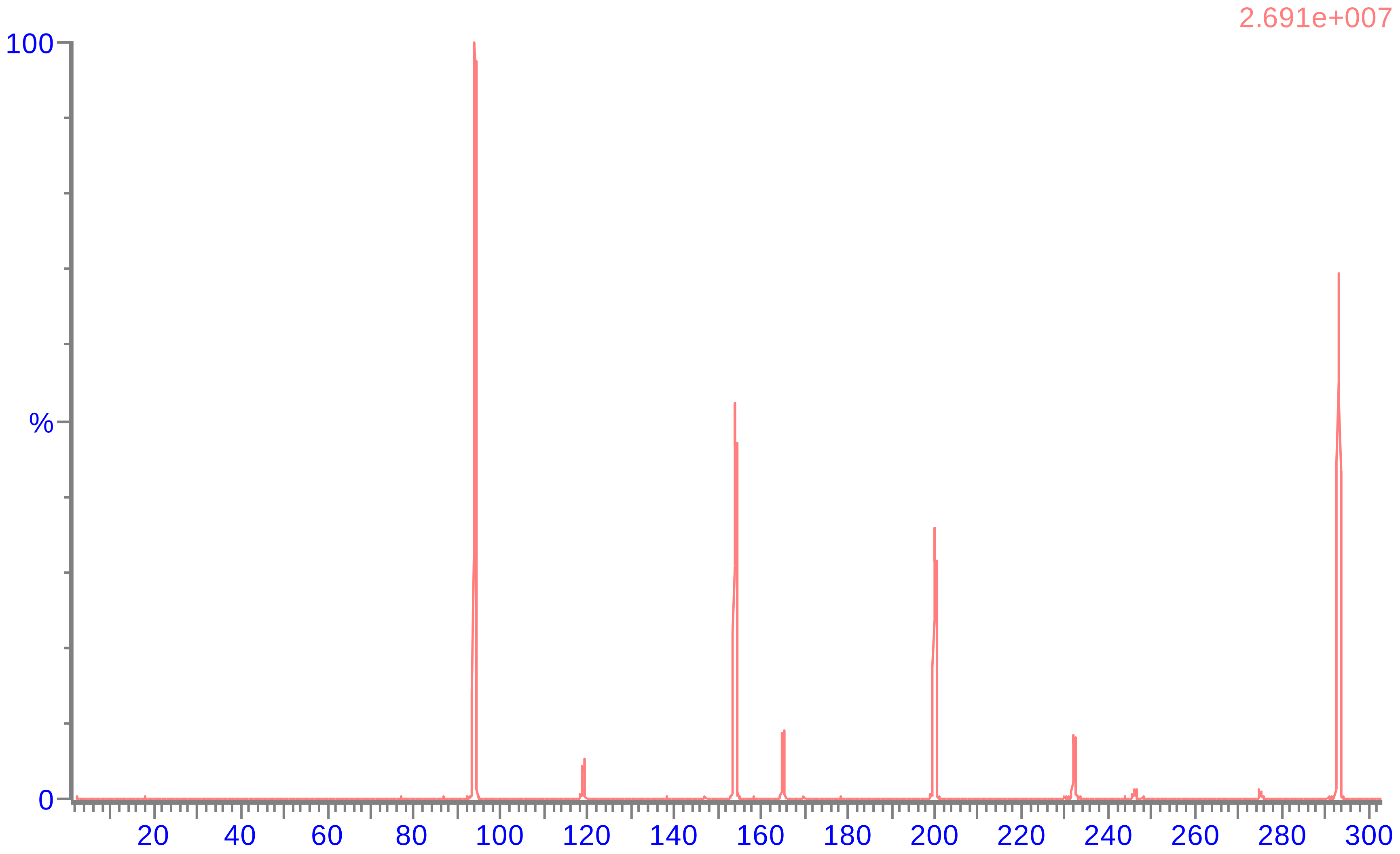

Figure 1.44: Mass spectrum for daughter fragment peak ES+, m/z 293.10 -> 200.07.

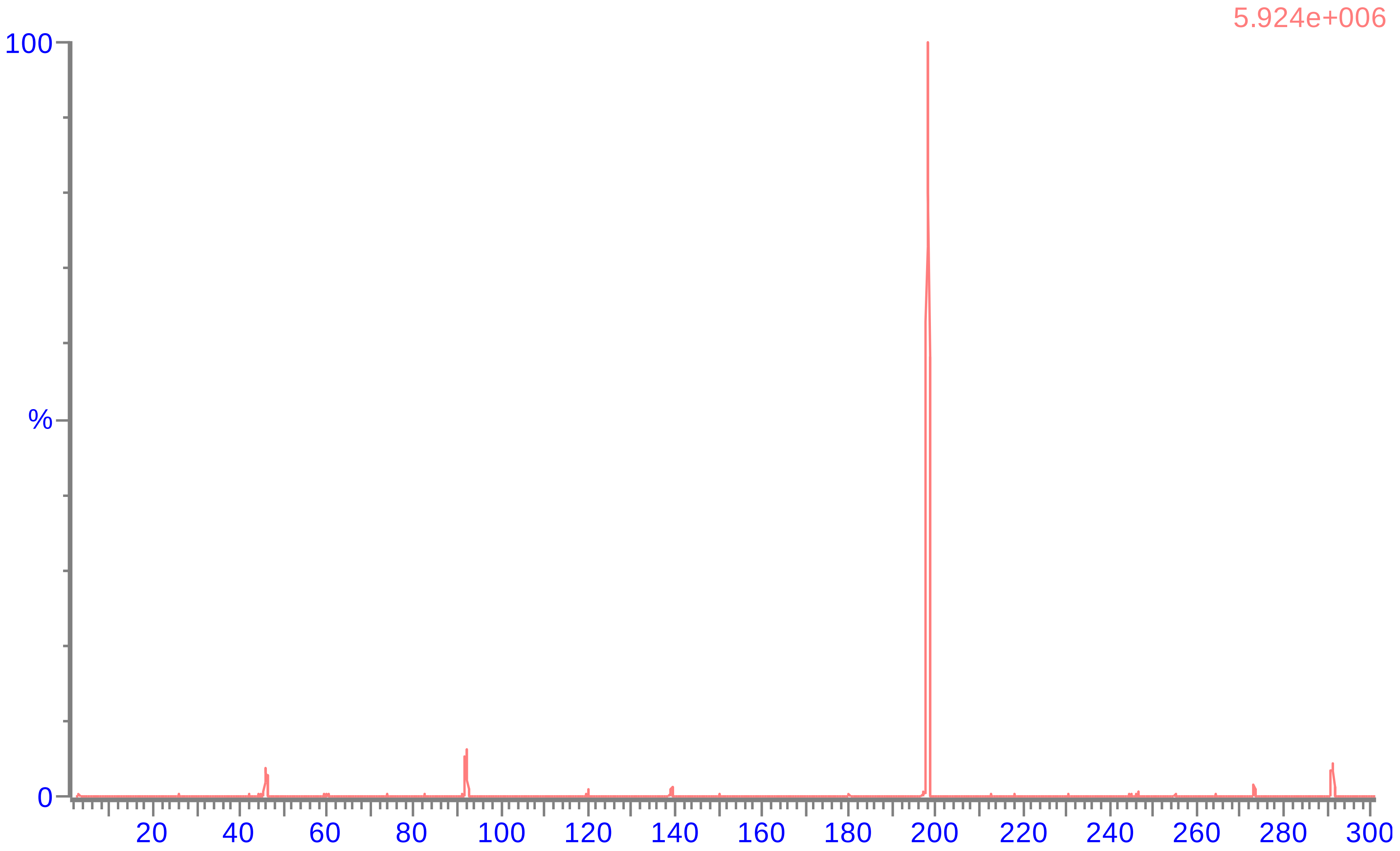

Figure 1.45: Mass spectrum for daughter fragment peak ES+, m/z 293.10 -> 198.07.

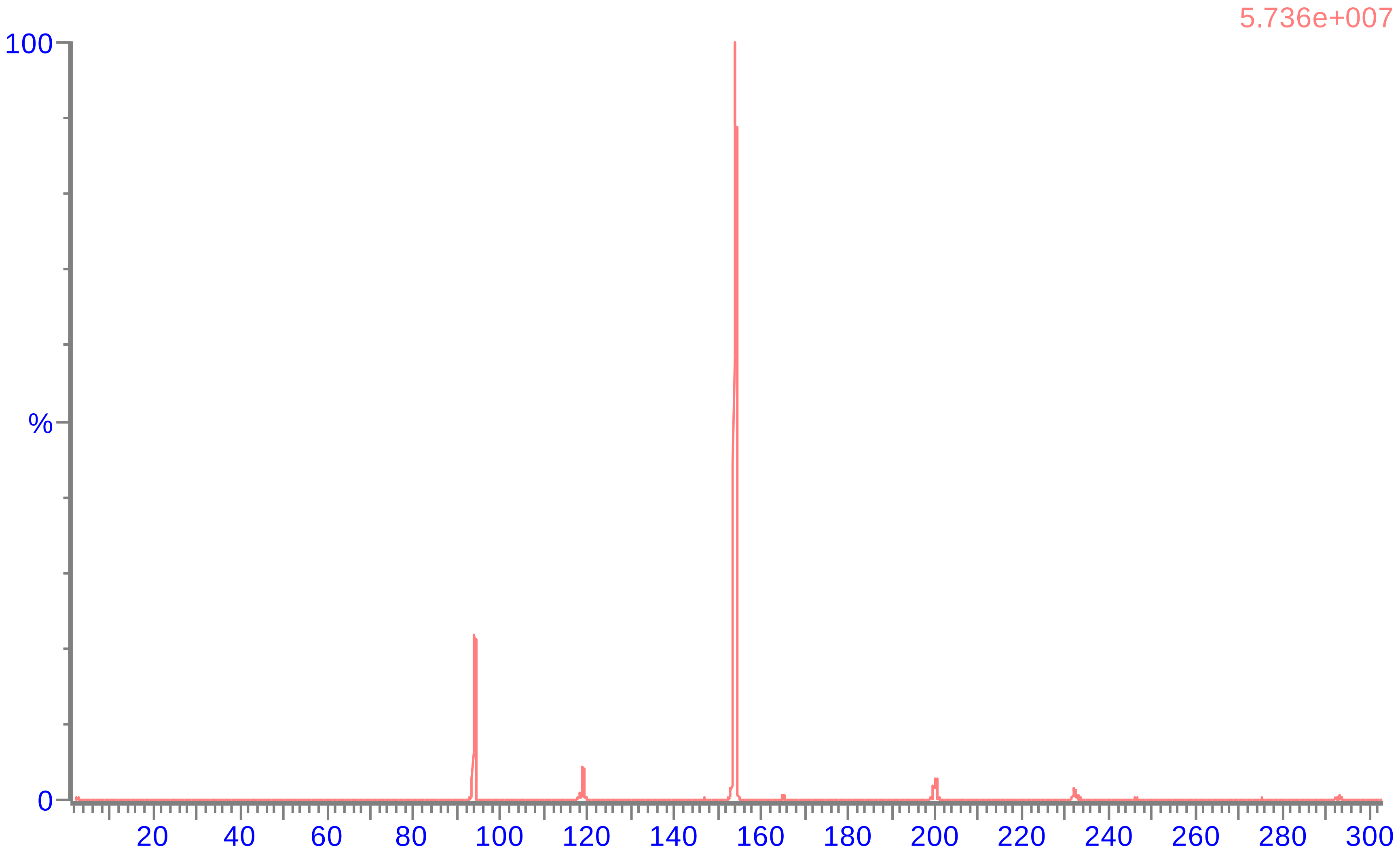

Figure 1.46: Mass spectrum for daughter fragment peak ES+, m/z 293.10 -> 119.03.

7g *N*-(1-([1,1-biphenyl]-4-yl)-2-nitroethyl)aniline

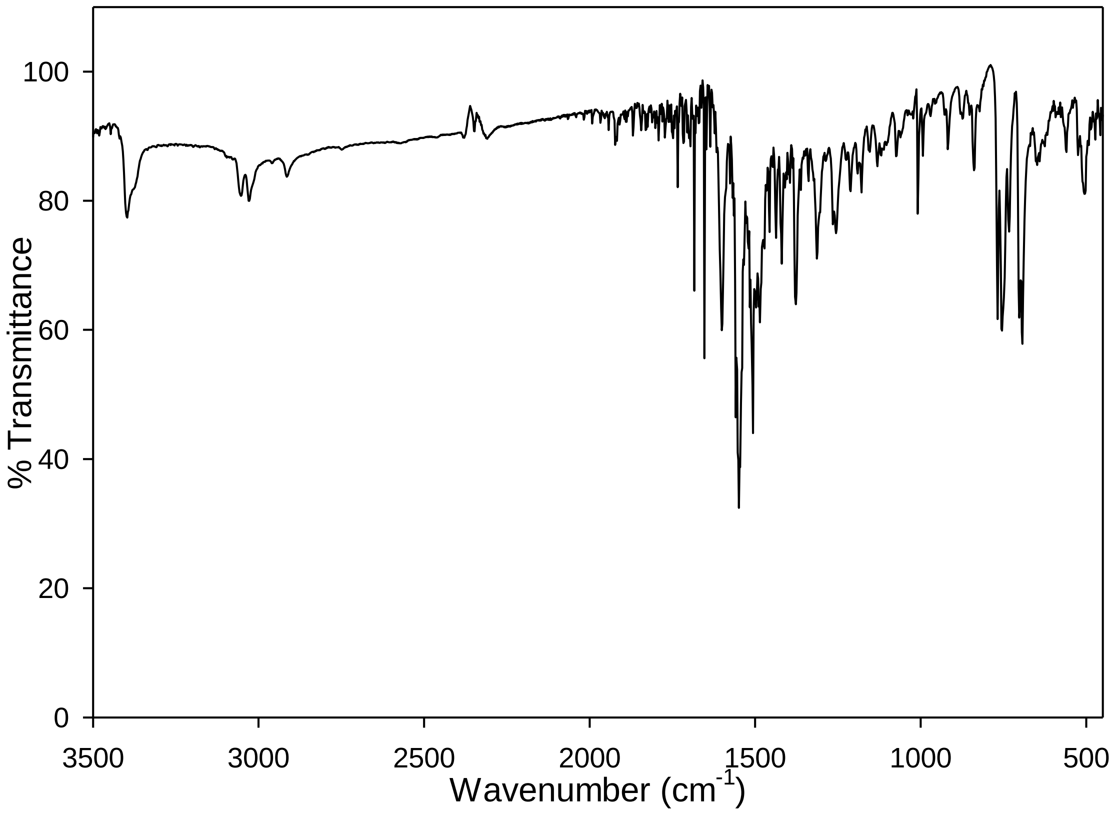

Figure 1.47: IR spectrum of **7g** *N*-(1-([1,1-biphenyl]-4-yl)-2-nitroethyl)aniline.

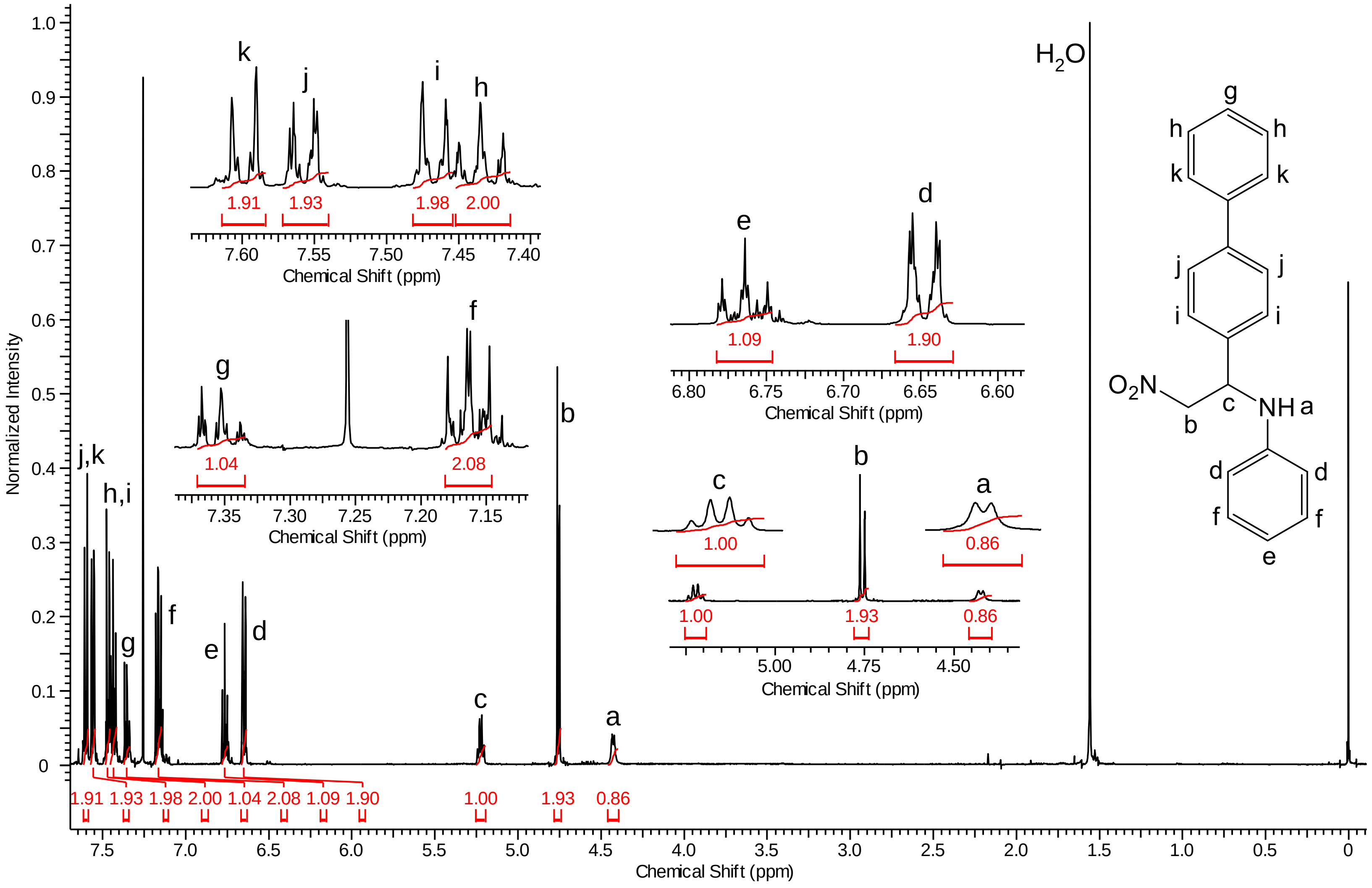

Figure 1.48: ^{1}H NMR spectrum of **7g** *N*-(1-([1,1-biphenyl]-4-yl)-2-nitroethyl)aniline.

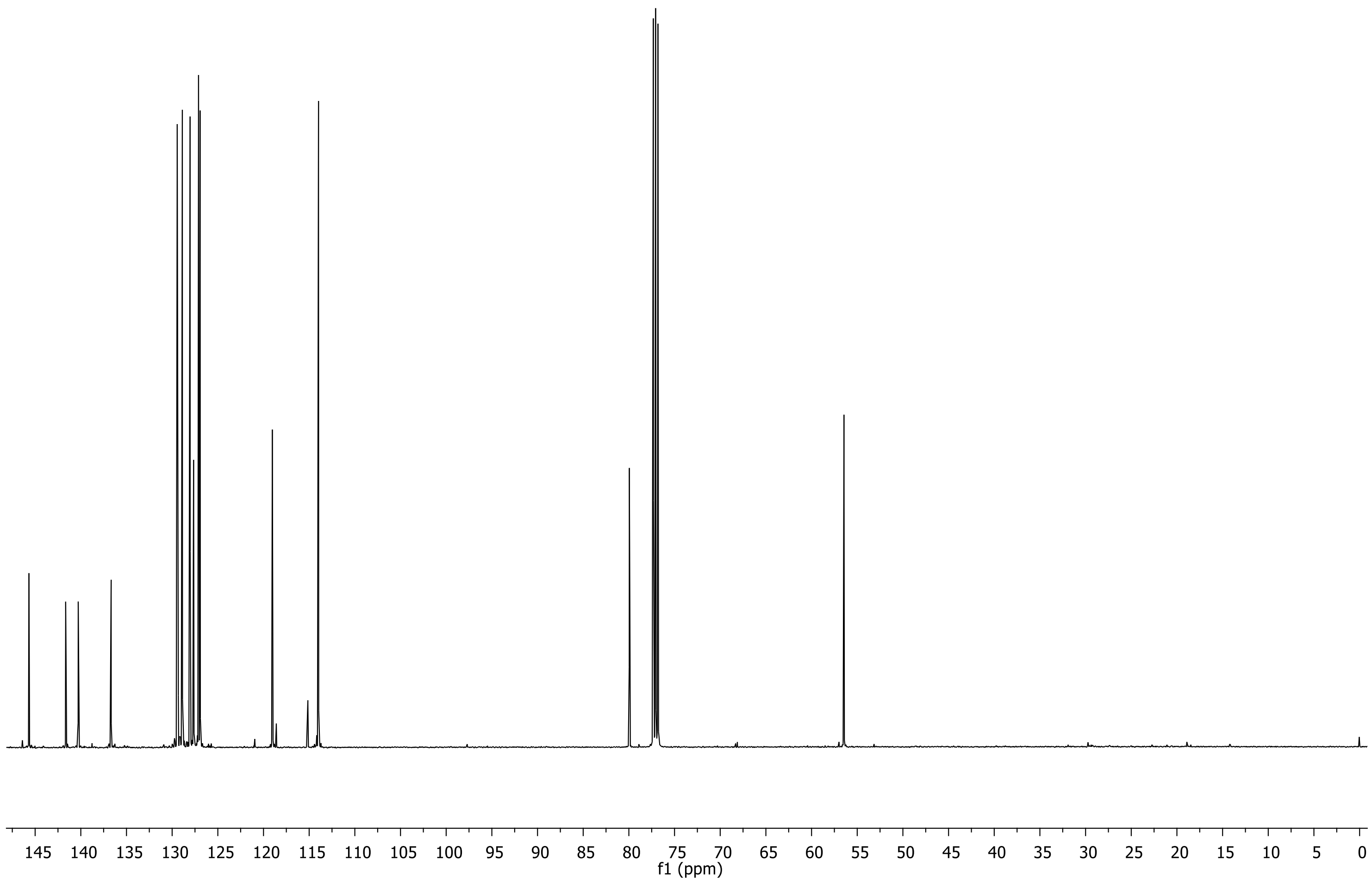

Figure 1.49: ^{13}C NMR spectrum of **7g** *N*-(1-([1,1-biphenyl]-4-yl)-2-nitroethyl)aniline.

Table 1.7 MS data.

Compound	Formula/Mass		Parent m/z	Cone Voltage	Daughters	Collision Energy	Ion Mode
**7g**	328	1	327.03	16	80.91	50	ES-

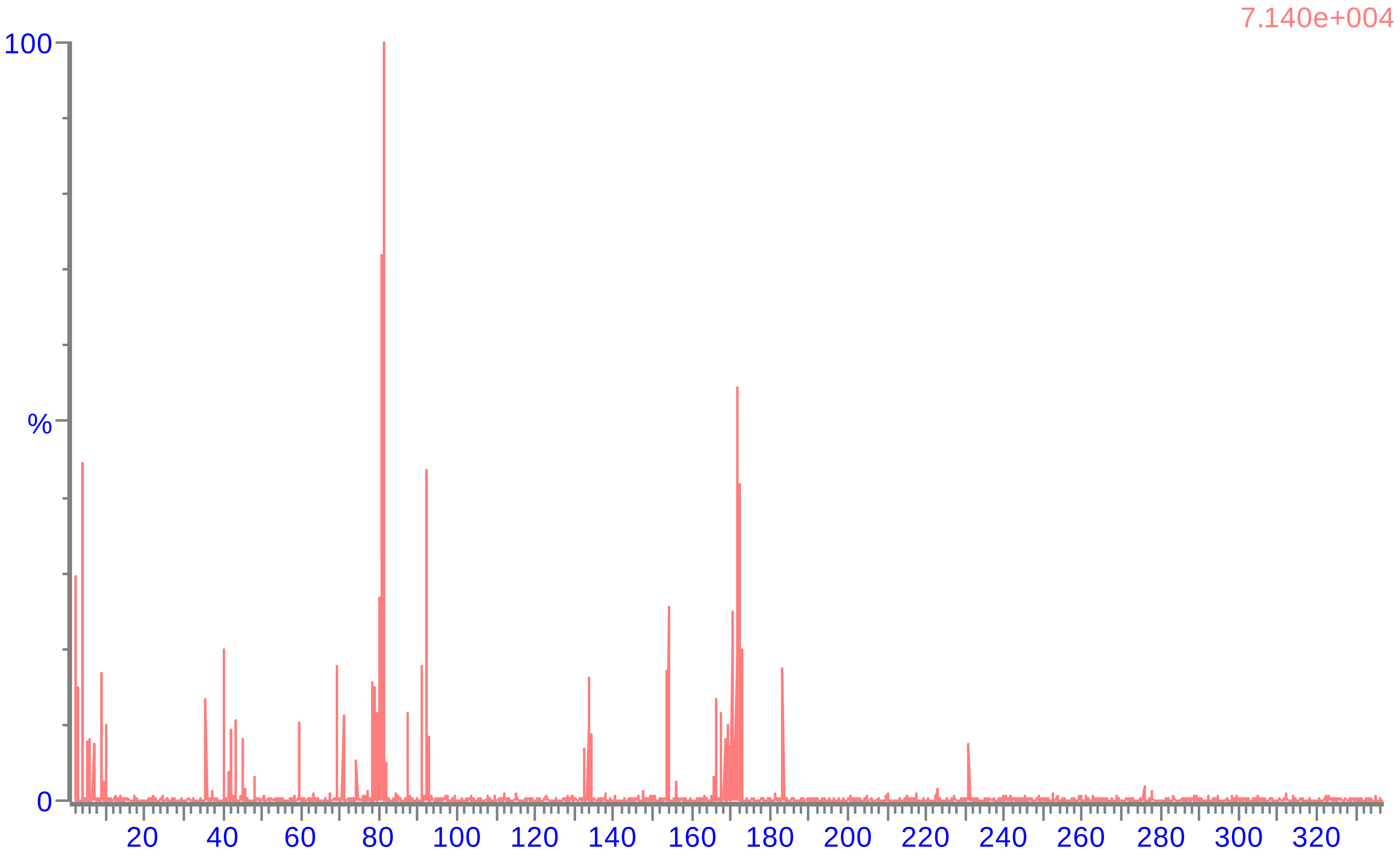

Figure 1.50: Mass spectrum for daughter fragment peak ES+, m/z 327.03 -> 80.91.

7h *N*-(1-(3,4-dichlorophenyl)-2-nitroethyl)aniline

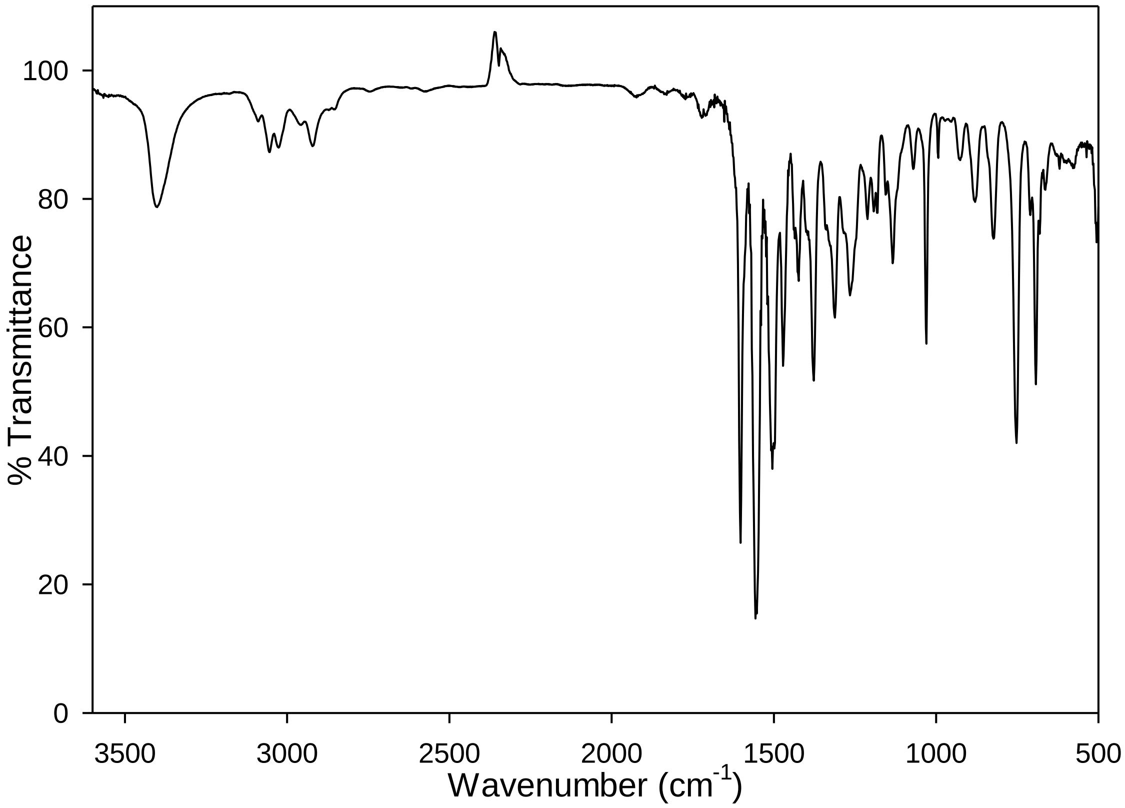

Figure 1.51: IR spectrum of **7h** *N*-(1-(3,4-dichlorophenyl)-2-nitroethyl)aniline.

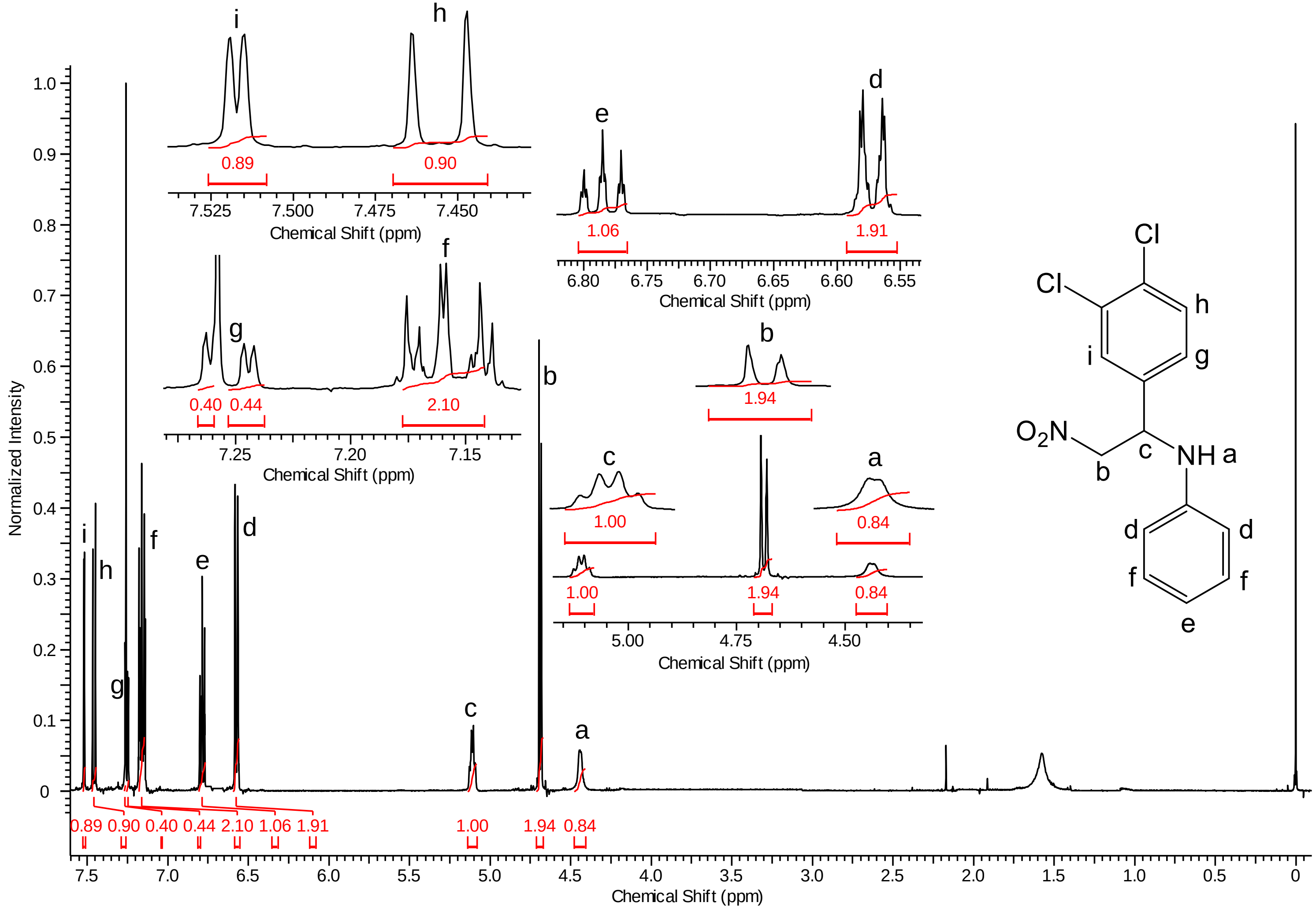

Figure 1.52: ^{1}H NMR spectrum of **7h** *N*-(1-(3,4-dichlorophenyl)-2-nitroethyl)aniline.

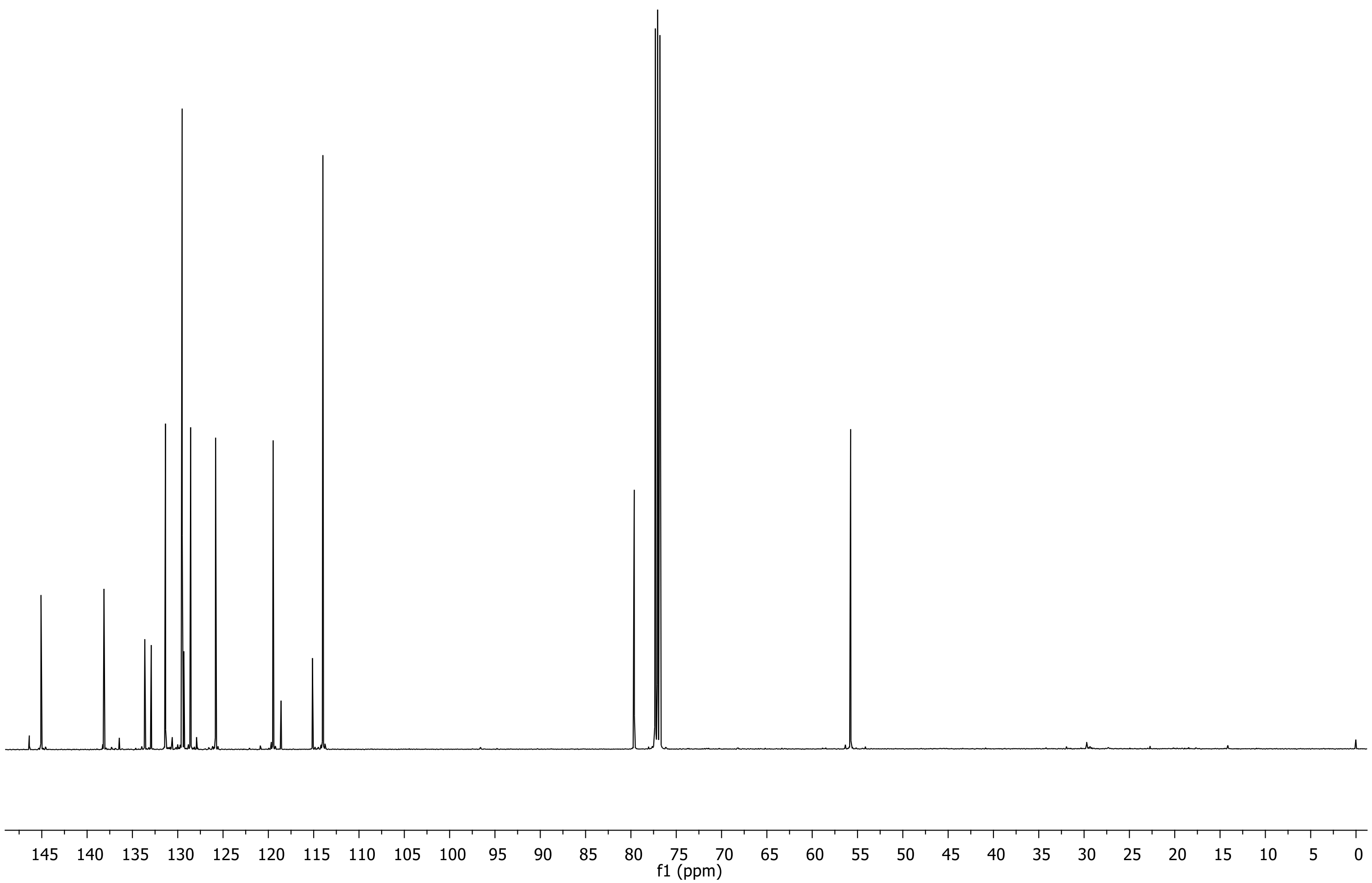

Figure 1.53: ^{13}C NMR spectrum of **7h** *N*-(1-(3,4-dichlorophenyl)-2-nitroethyl)aniline.

Table 1.8: MS data.

**Compound**	**Formula/Mass**		**Parent m/z**	**Cone Voltage**	**Daughters**	**Collision Energy**	**Ion Mode**
**7h**	312	1	312.97	16	93.98	12	ES+
		2	312.97	18	217.96	8	ES-
		3	312.97	16	252.02	12	ES+
		4	312.97	16	173.91	26	ES+
		5	312.97	16	101.73	58	ES+

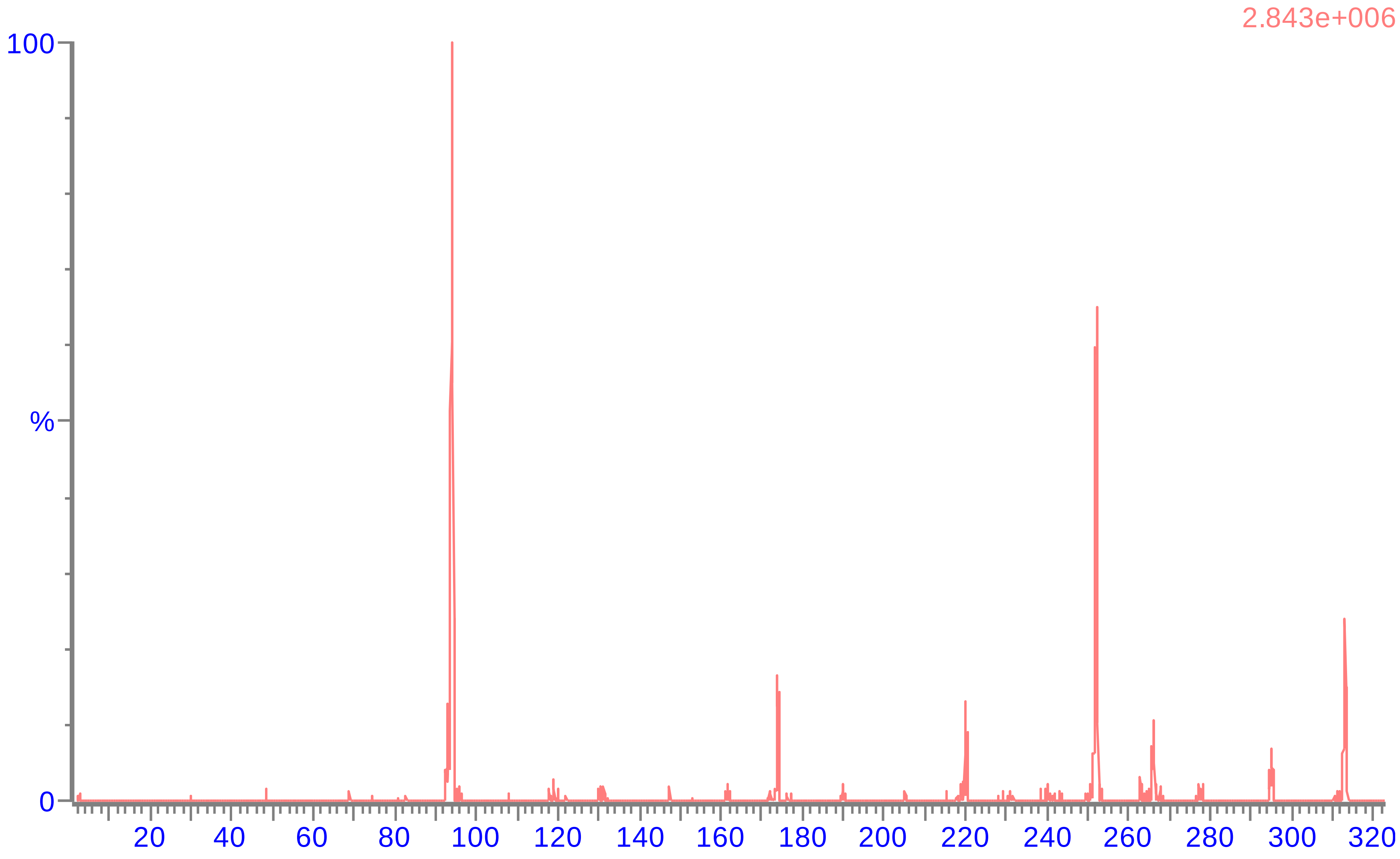

Figure 1.54: Mass spectrum for daughter fragment peak ES+, m/z 312.97 -> 93.98.

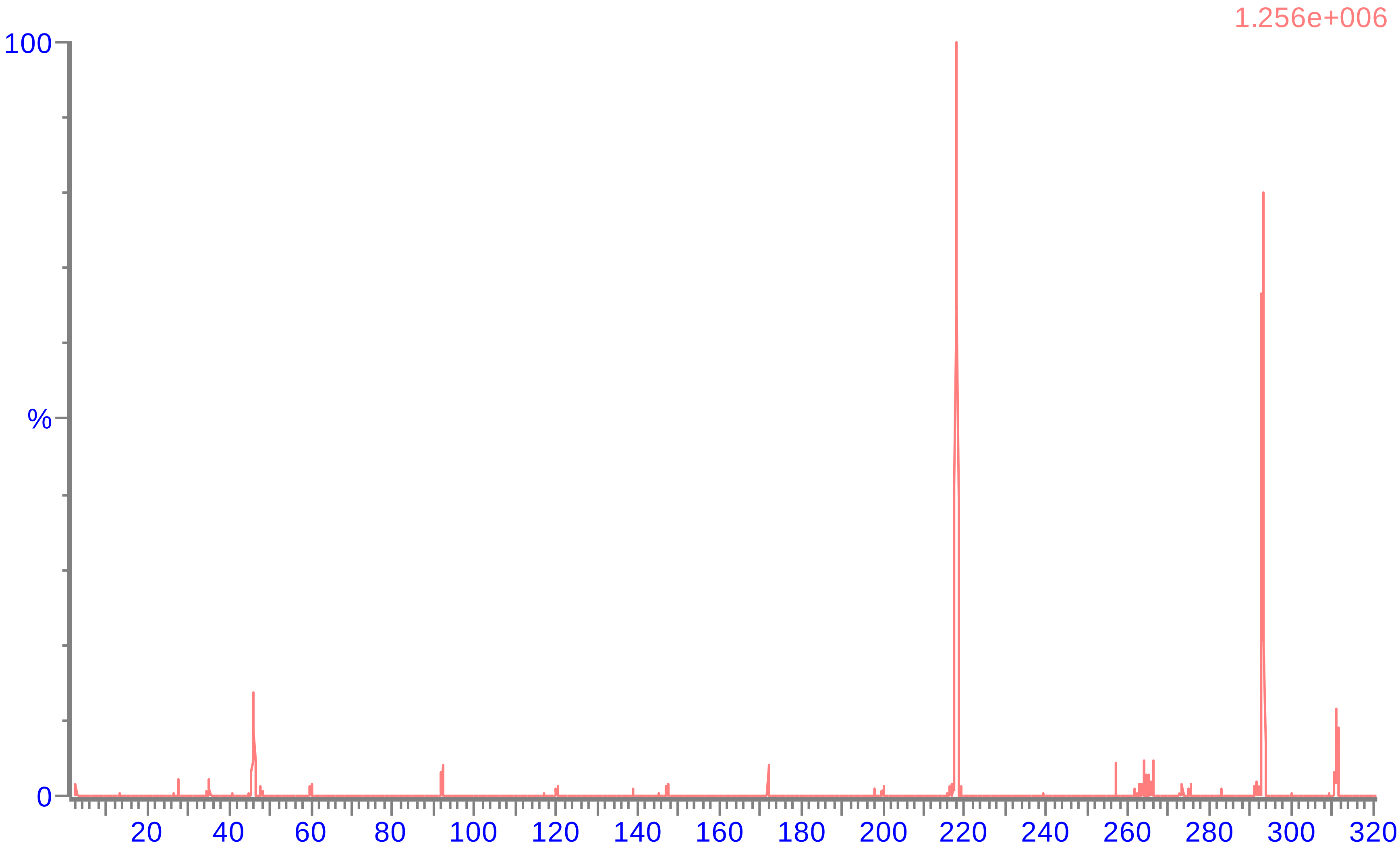

Figure 1.55: Mass spectrum for daughter fragment peak ES+, m/z 312.97 -> 217.96.

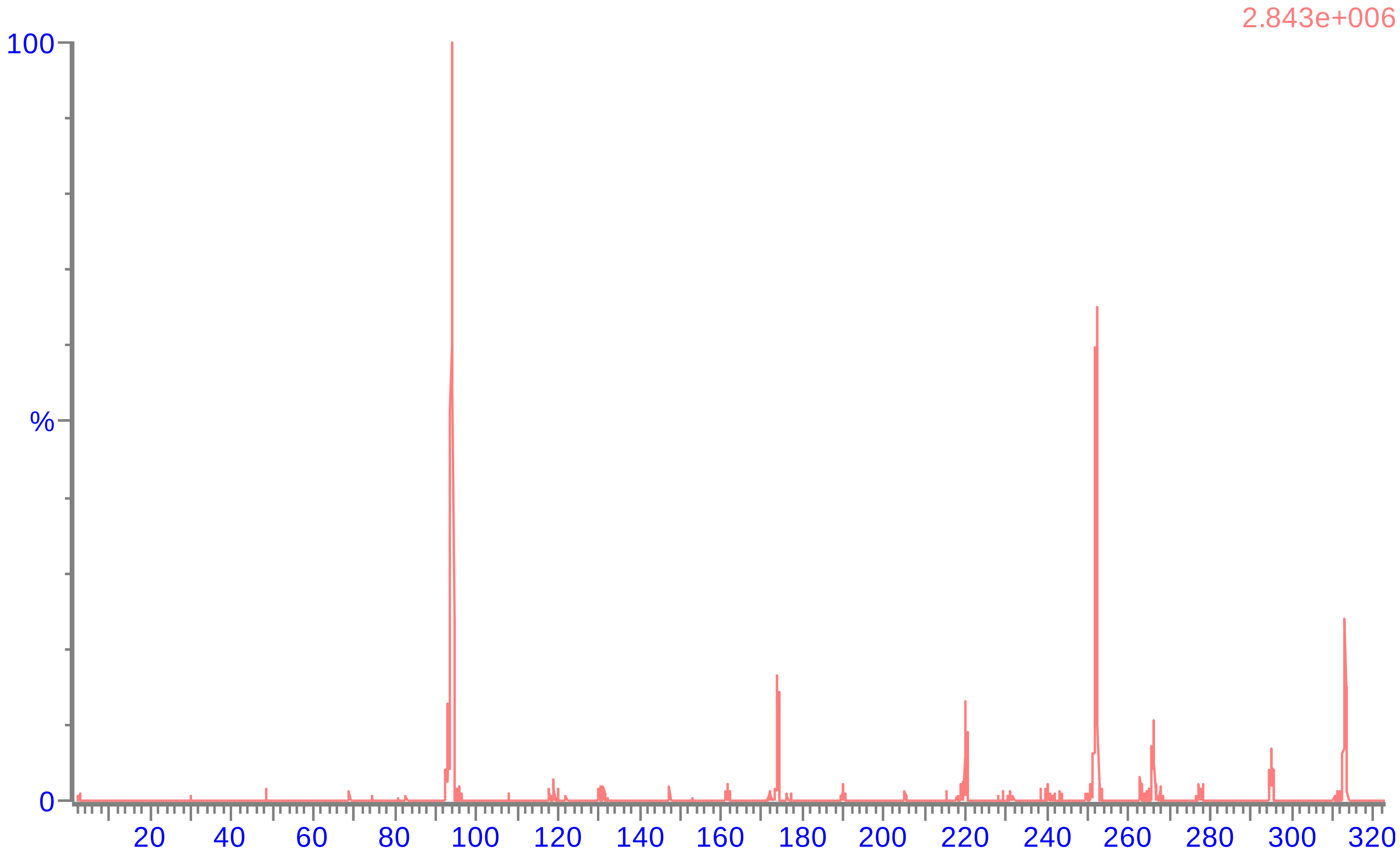

Figure 1.56: Mass spectrum for daughter fragment peak ES+, m/z 312.97 -> 252.02.

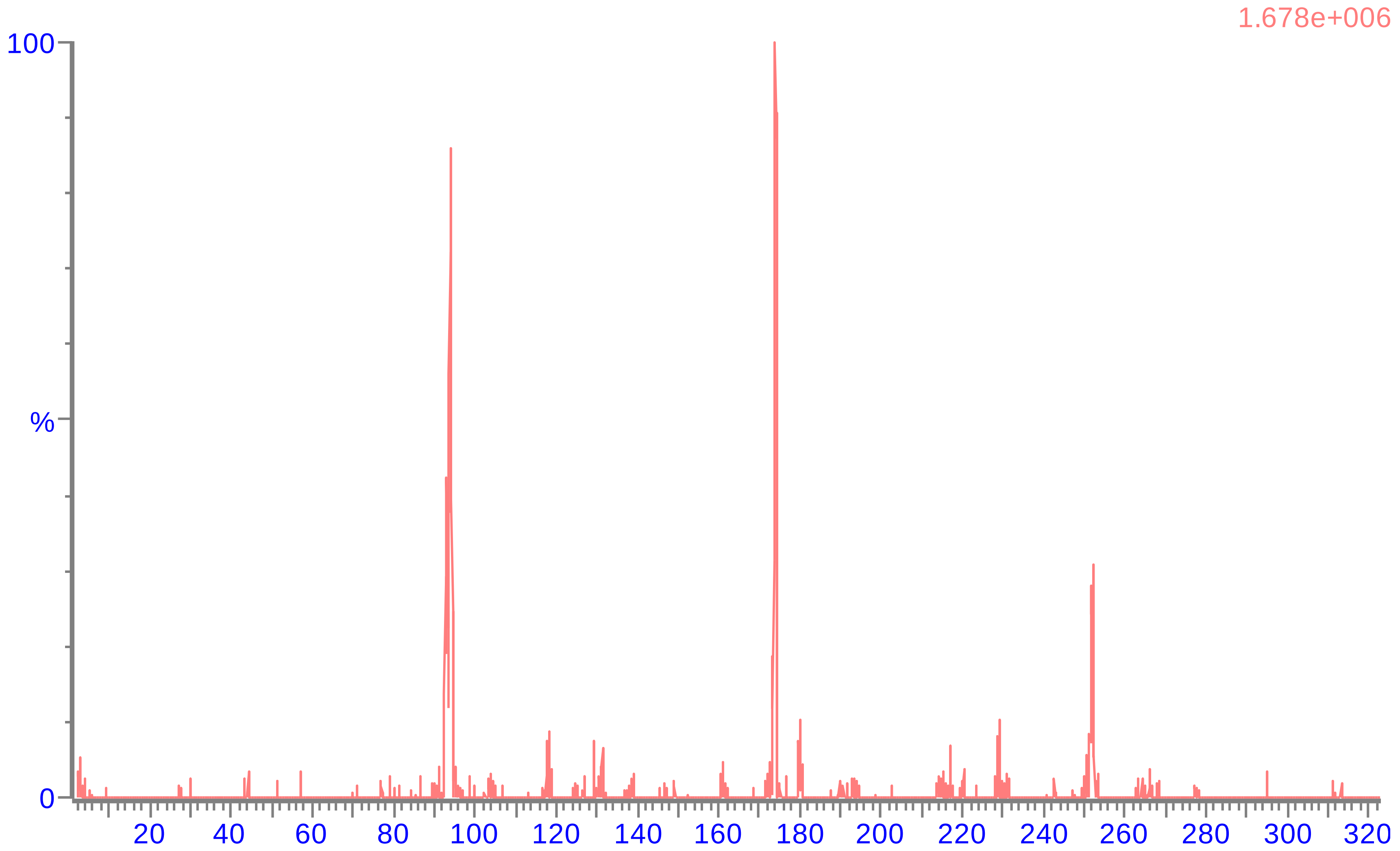

Figure 1.57: Mass spectrum for daughter fragment peak ES+, m/z 312.97 -> 173.91.

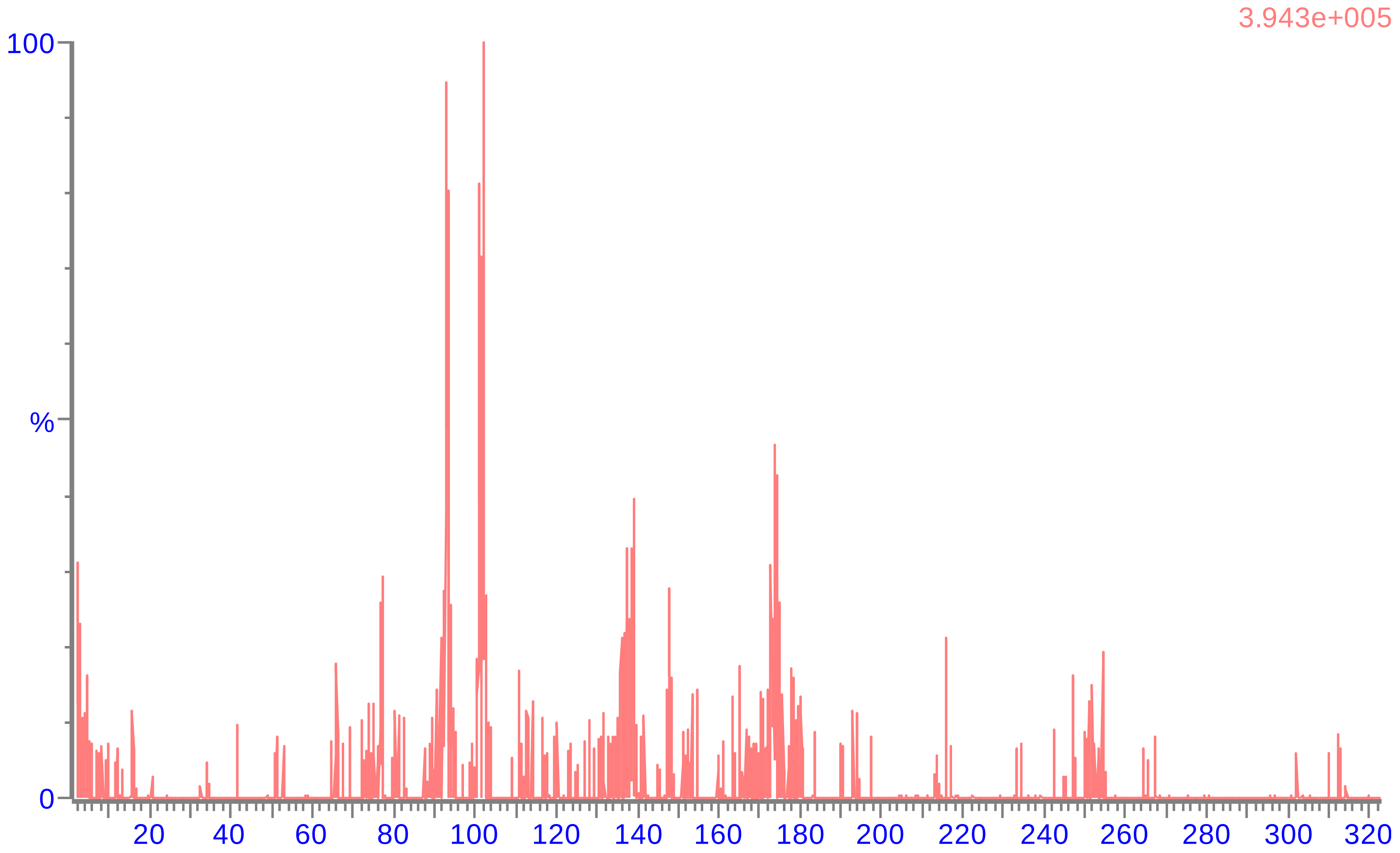

Figure 1.58: Mass spectrum for daughter fragment peak ES+, m/z 312.97 -> 101.73.

7i *N*-(1-(2,6-dichlorophenyl)-2-nitroethyl)aniline.

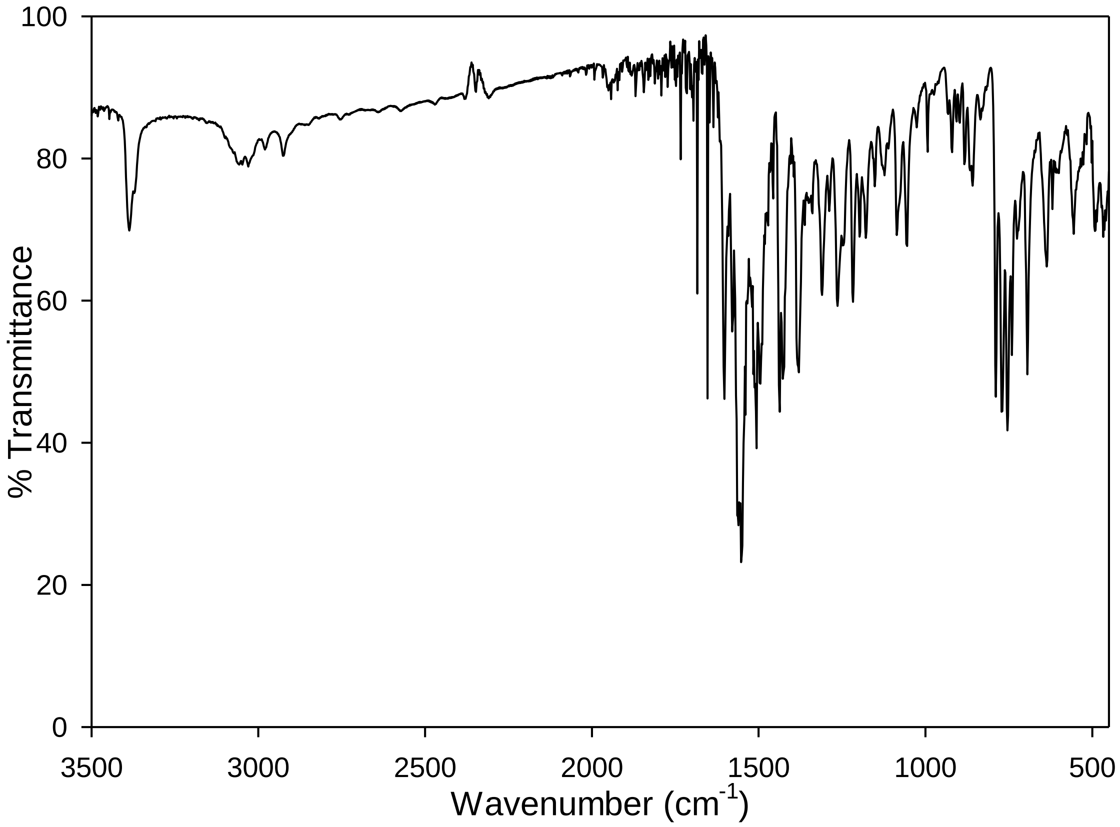

Figure 1.57: IR spectrum of **7i** *N*-(1-(2,6-dichlorophenyl)-2-nitroethyl)aniline.

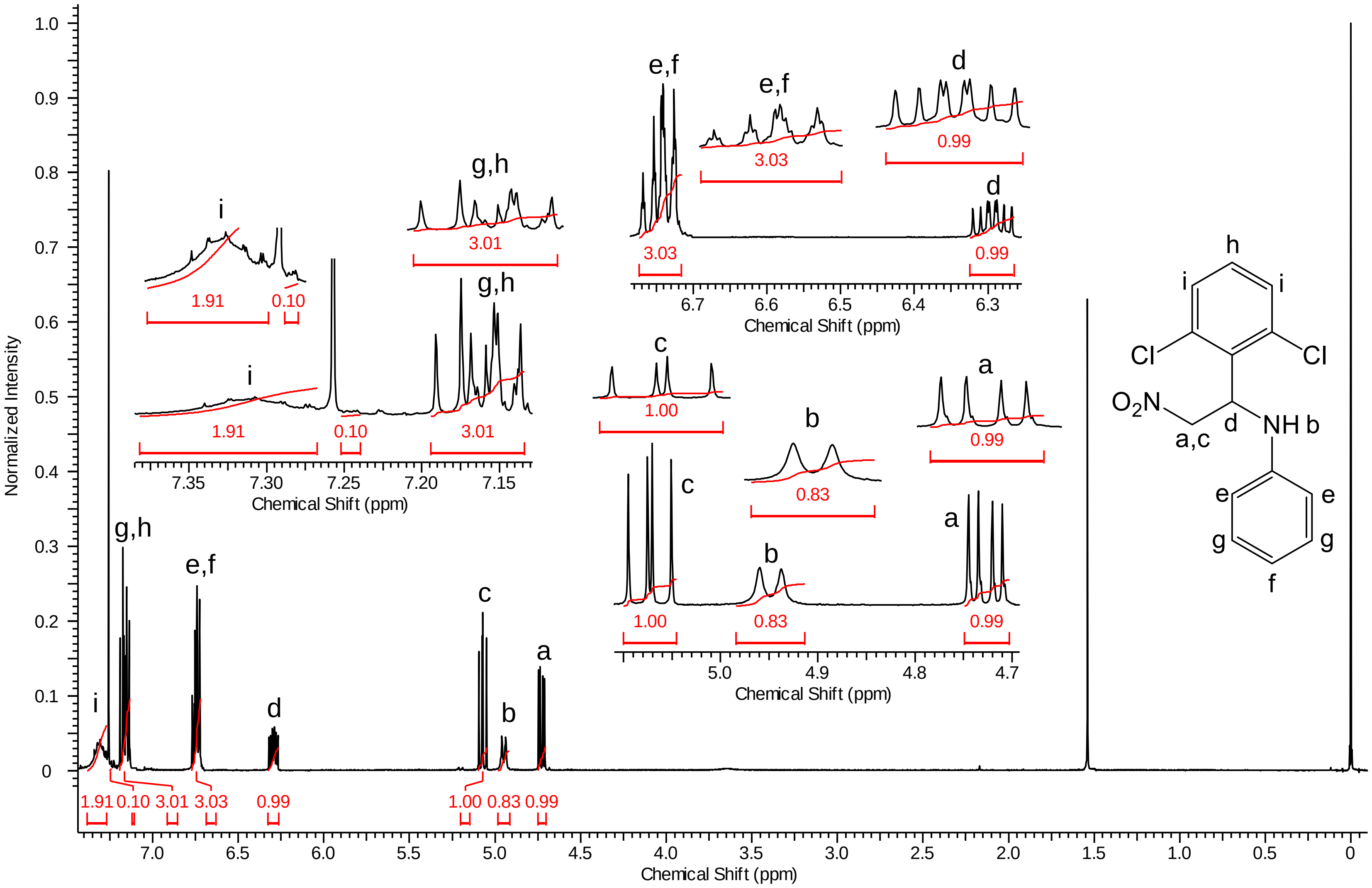

Figure 1.60: ^{1}H NMR spectrum of **7i** *N*-(1-(2,6-dichlorophenyl)-2-nitroethyl)aniline.

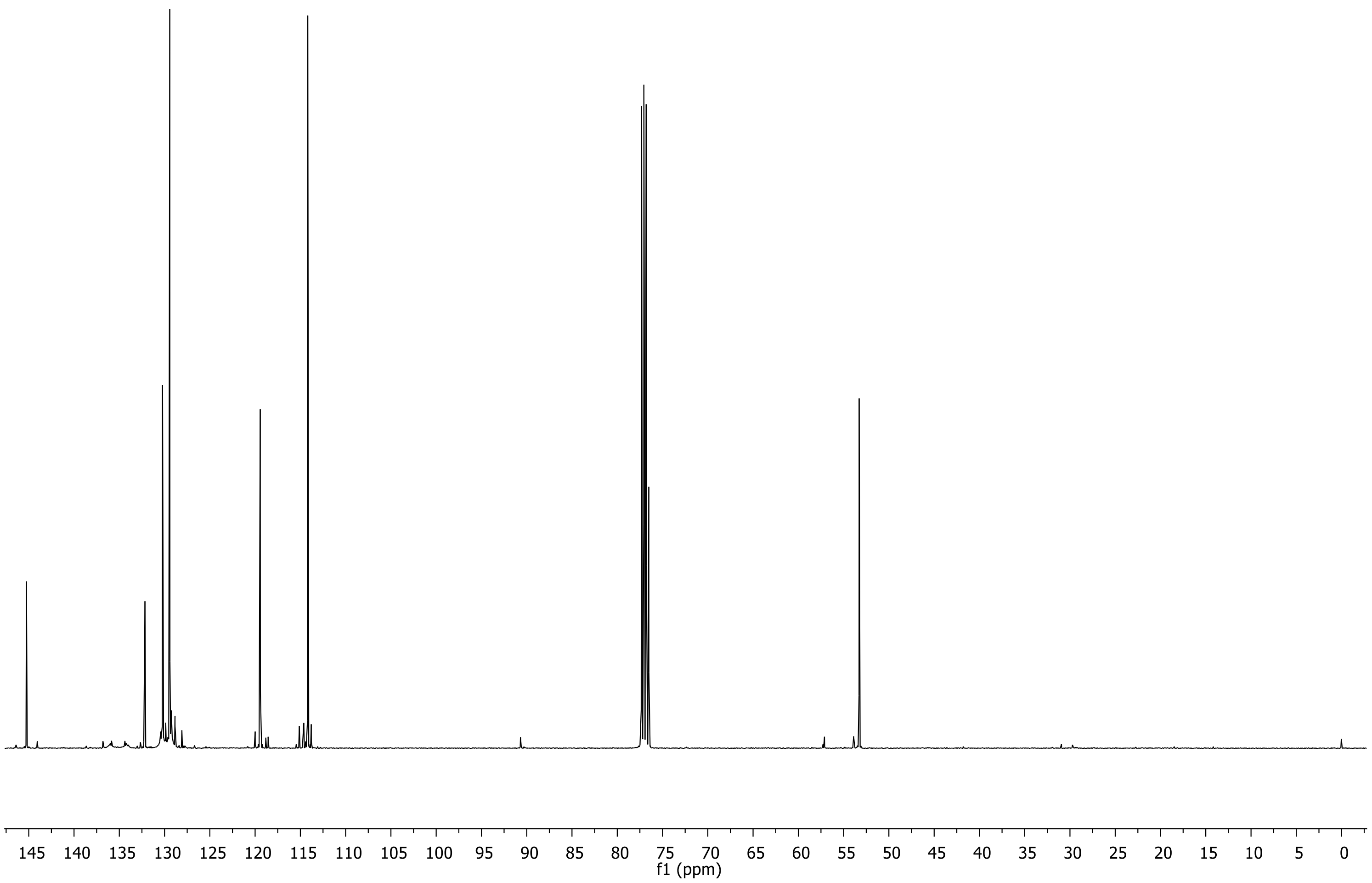

Figure 1.61: ^{13}C NMR spectrum of **7i** *N*-(1-(2,6-dichlorophenyl)-2-nitroethyl)aniline.

Table 1.9: MS data.

Compound	Formula/Mass		Parent m/z	Cone Voltage	Daughters	Collision Energy	Ion Mode
**7i**	310	1	310.97	24	93.33	22	ES+
		2	310.97	24	250.02	12	ES+
		3	310.97	24	136.98	44	ES+
		4	310.97	24	124.95	34	ES+

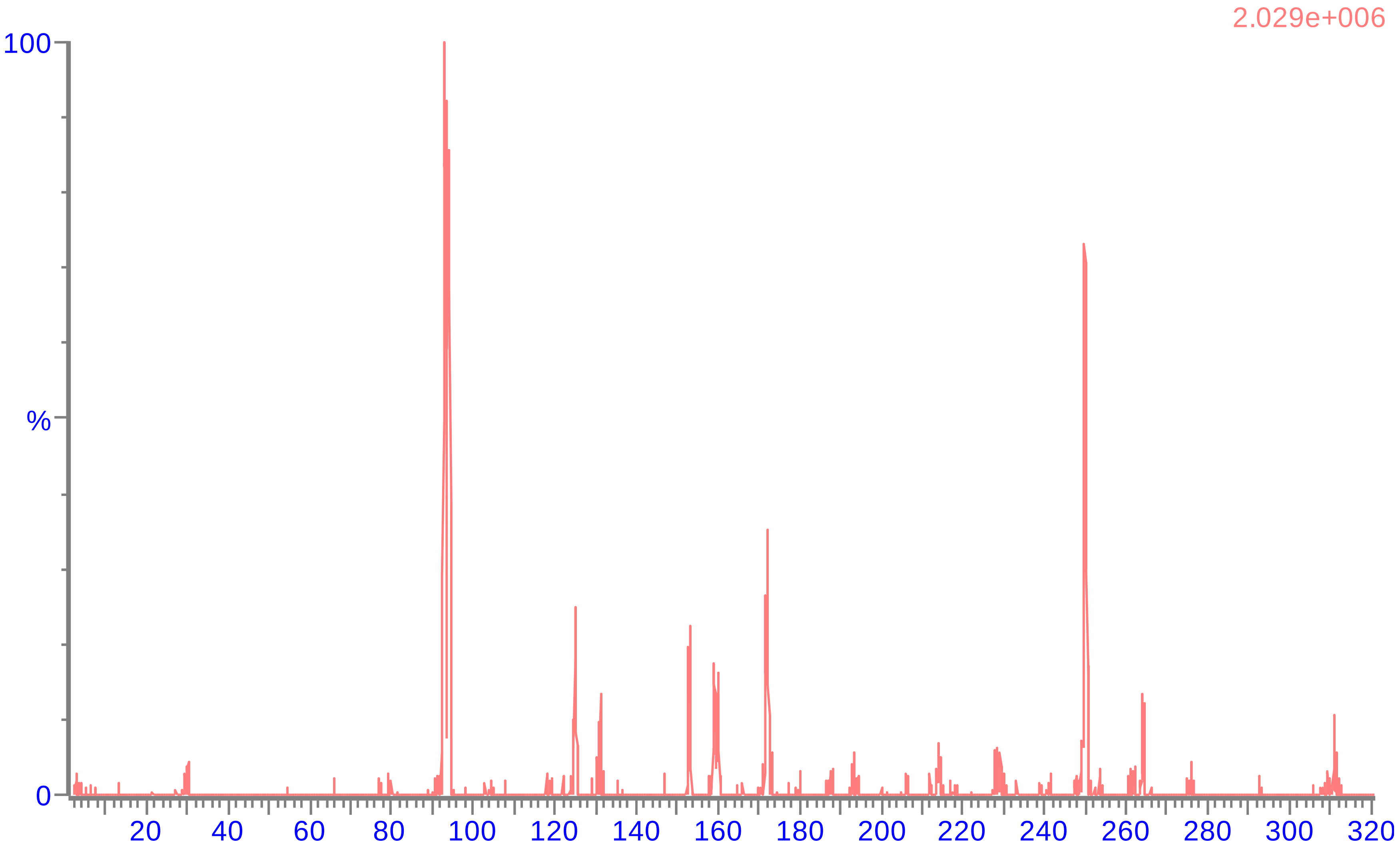

Figure 1.62: Mass spectrum for daughter fragment peak ES+, m/z 312.97 -> 93.33.

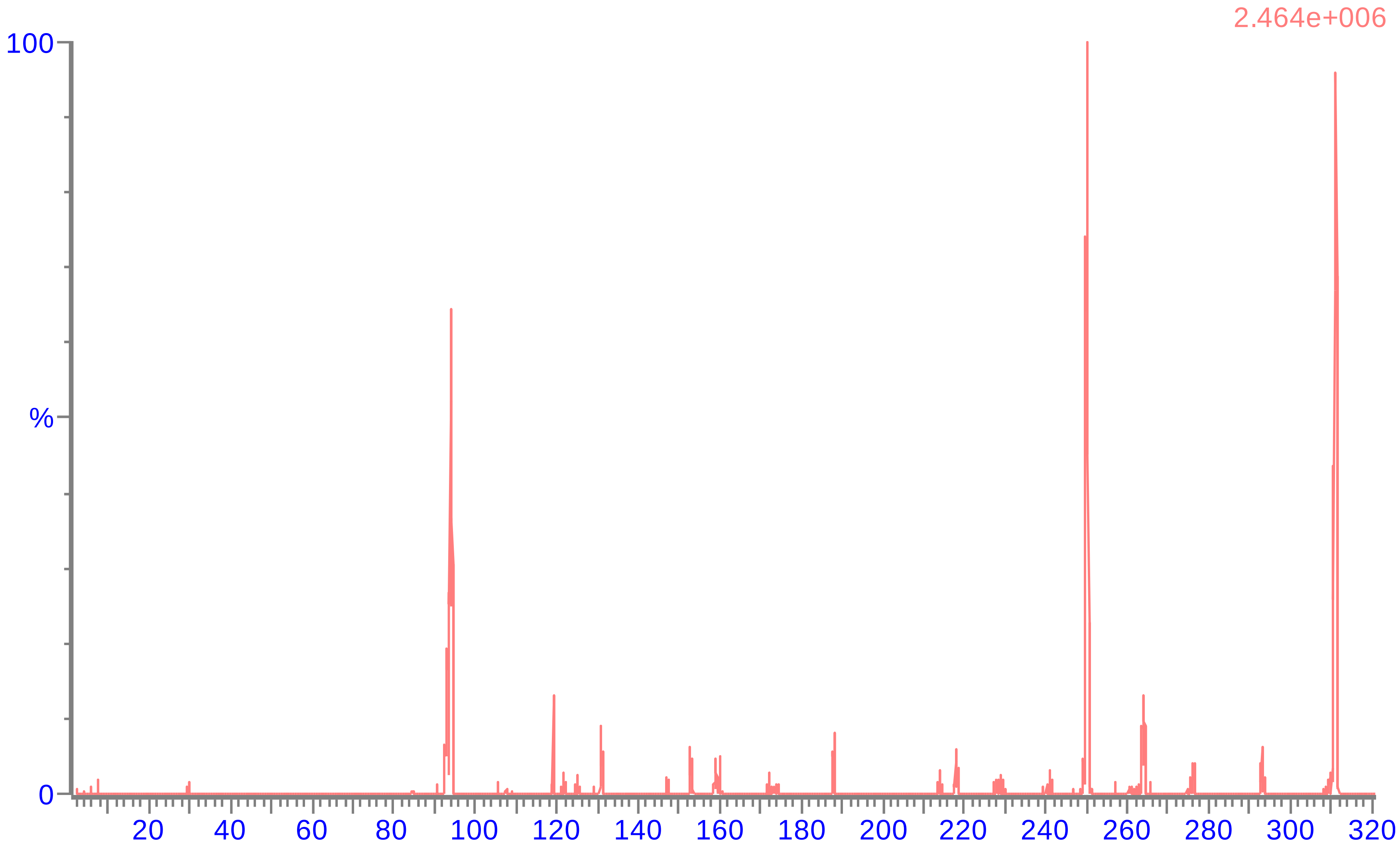

Figure 1.63: Mass spectrum for daughter fragment peak ES+, m/z 312.97 -> 250.02.

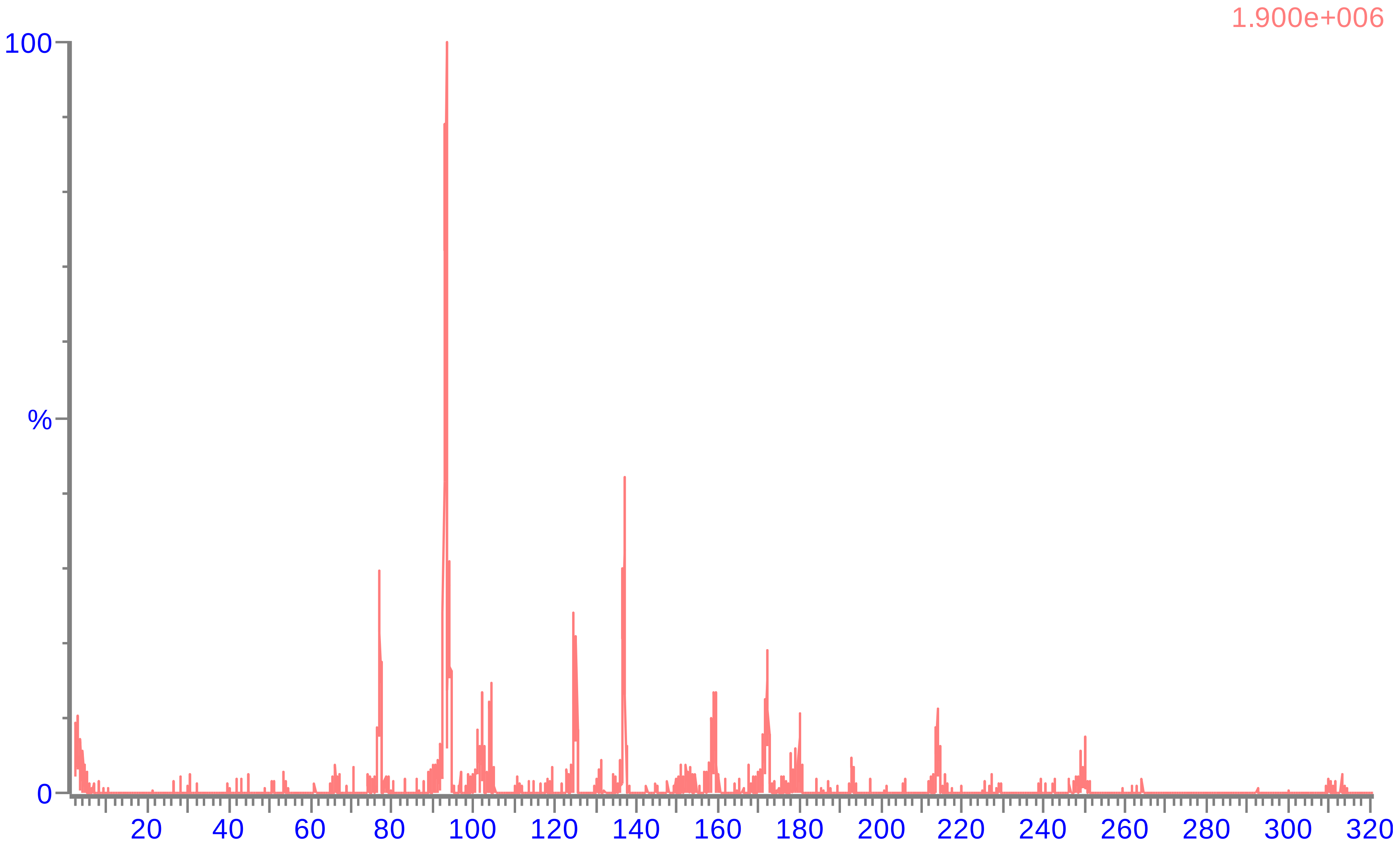

Figure 1.64: Mass spectrum for daughter fragment peak ES+, m/z 312.97 -> 136.98.

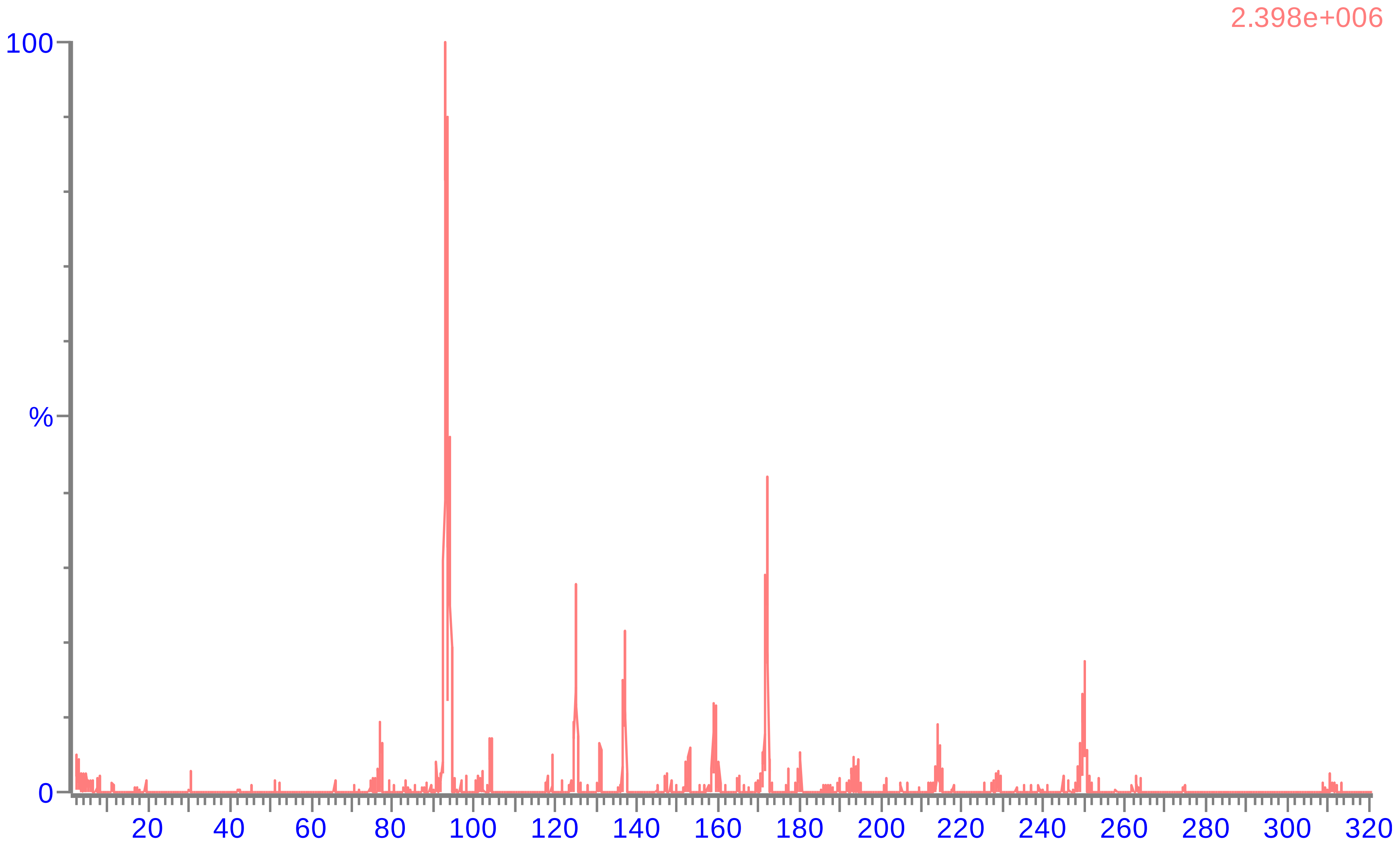

Figure 1.65: Mass spectrum for daughter fragment peak ES+, m/z 312.97 -> 124.95.

7j *N*-(1-(benzo[*d*][1,3]dioxol-5-yl)-2-nitroethyl)aniline

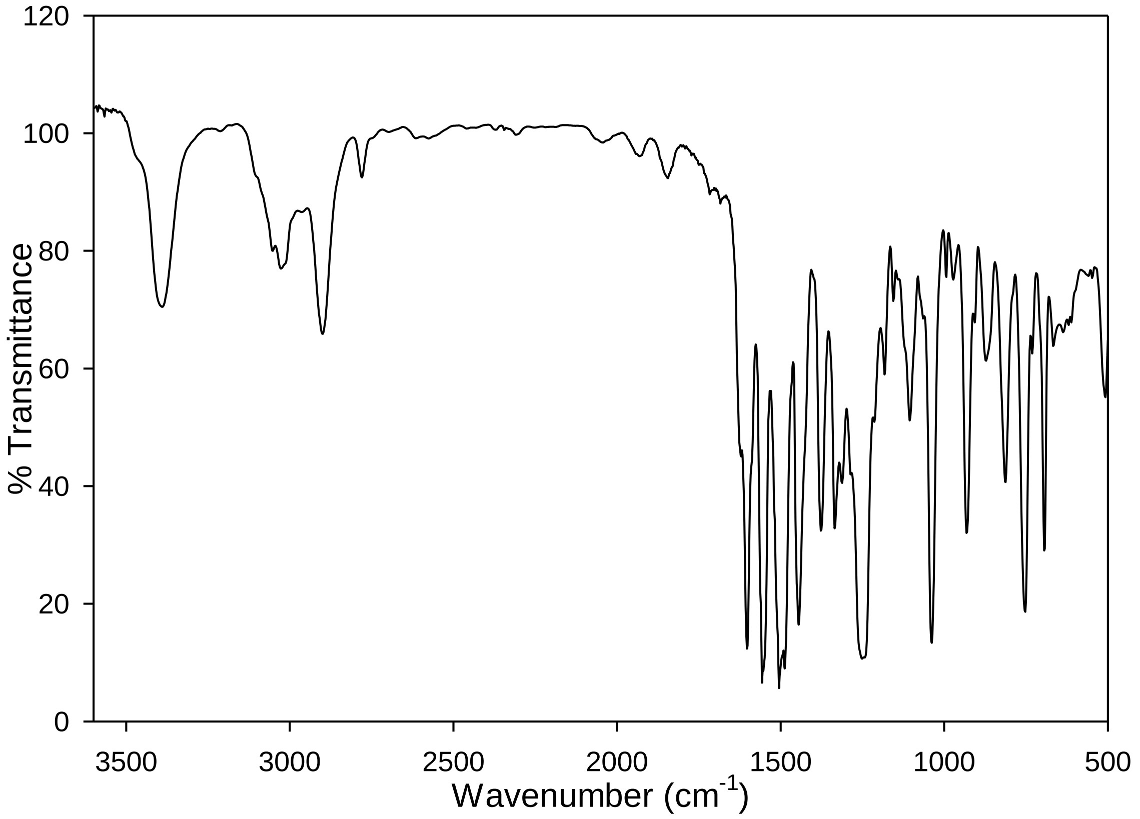

Figure 1.66: IR spectrum of **7j** *N*-(1-(benzo[*d*][1,3]dioxol-5-yl)-2-nitroethyl)aniline.

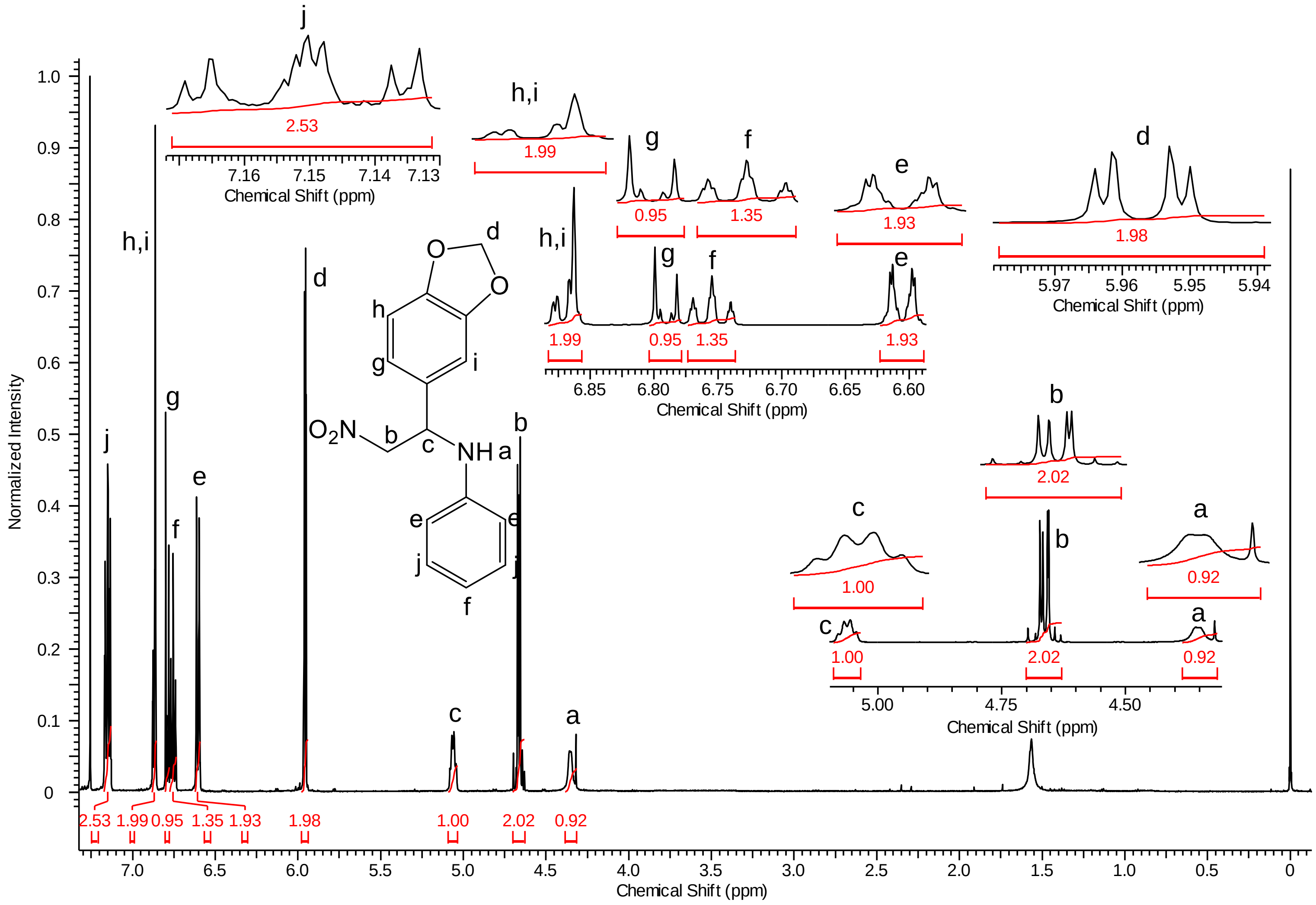

Figure 1.67: ^{1}H NMR spectrum of **7j** *N*-(1-(benzo[*d*][1,3]dioxol-5-yl)-2-nitroethyl)aniline.

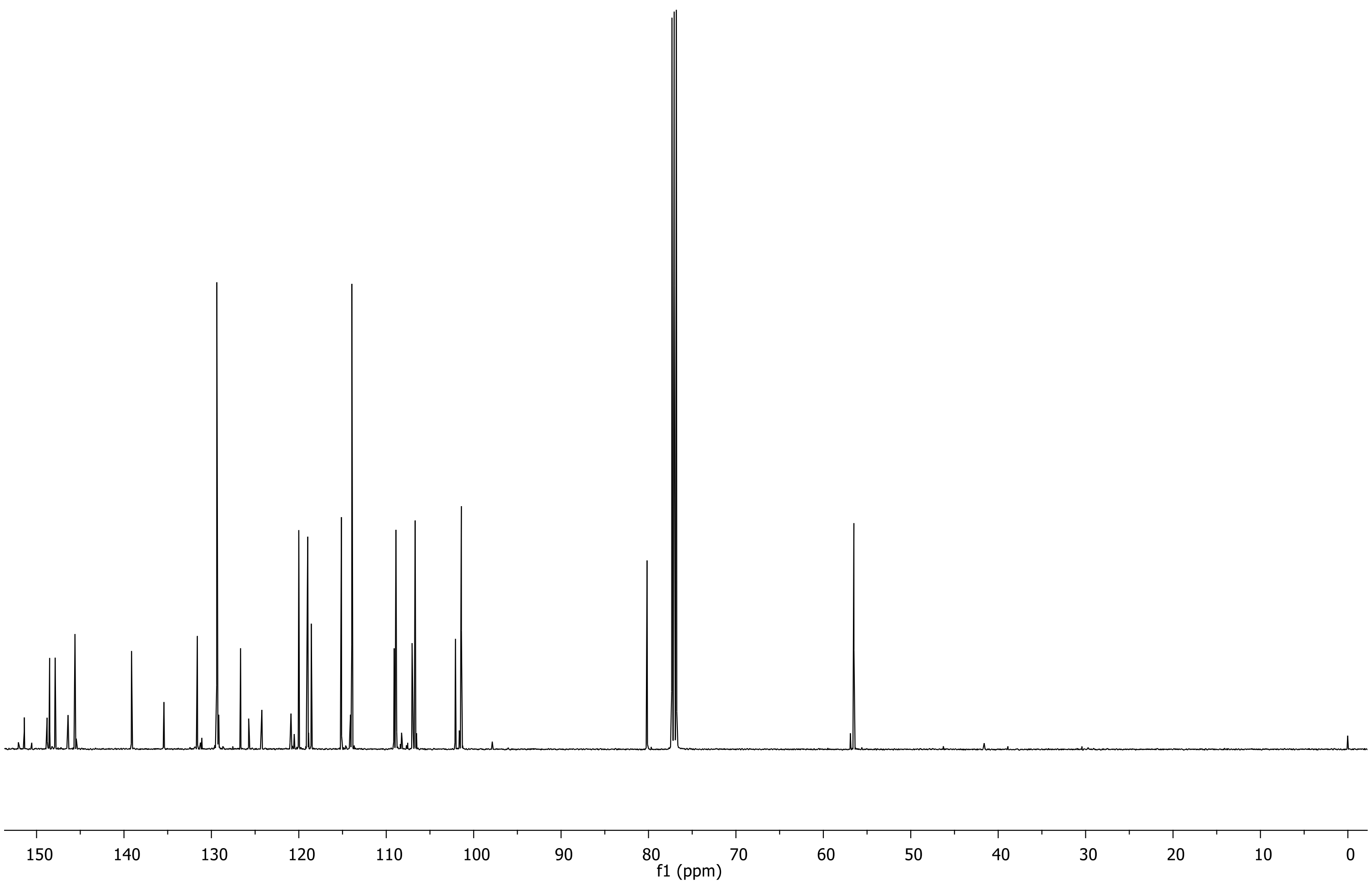

Figure 1.68: ^{13}C NMR spectrum of **7j** *N*-(1-(benzo[*d*][1,3]dioxol-5-yl)-2-nitroethyl)aniline.

Table 1.10: MS data.

Compound	Formula/Mass		Parent m/z	Cone Voltage	Daughters	Collision Energy	Ion Mode
**7j**	286	1	287.10	14	148.03	14	ES+
		2	287.10	14	94.05	6	ES+
		3	287.10	14	194.02	8	ES+
		4	287.10	14	91.64	64	ES+
		5	287.10	14	90.99	56	ES+

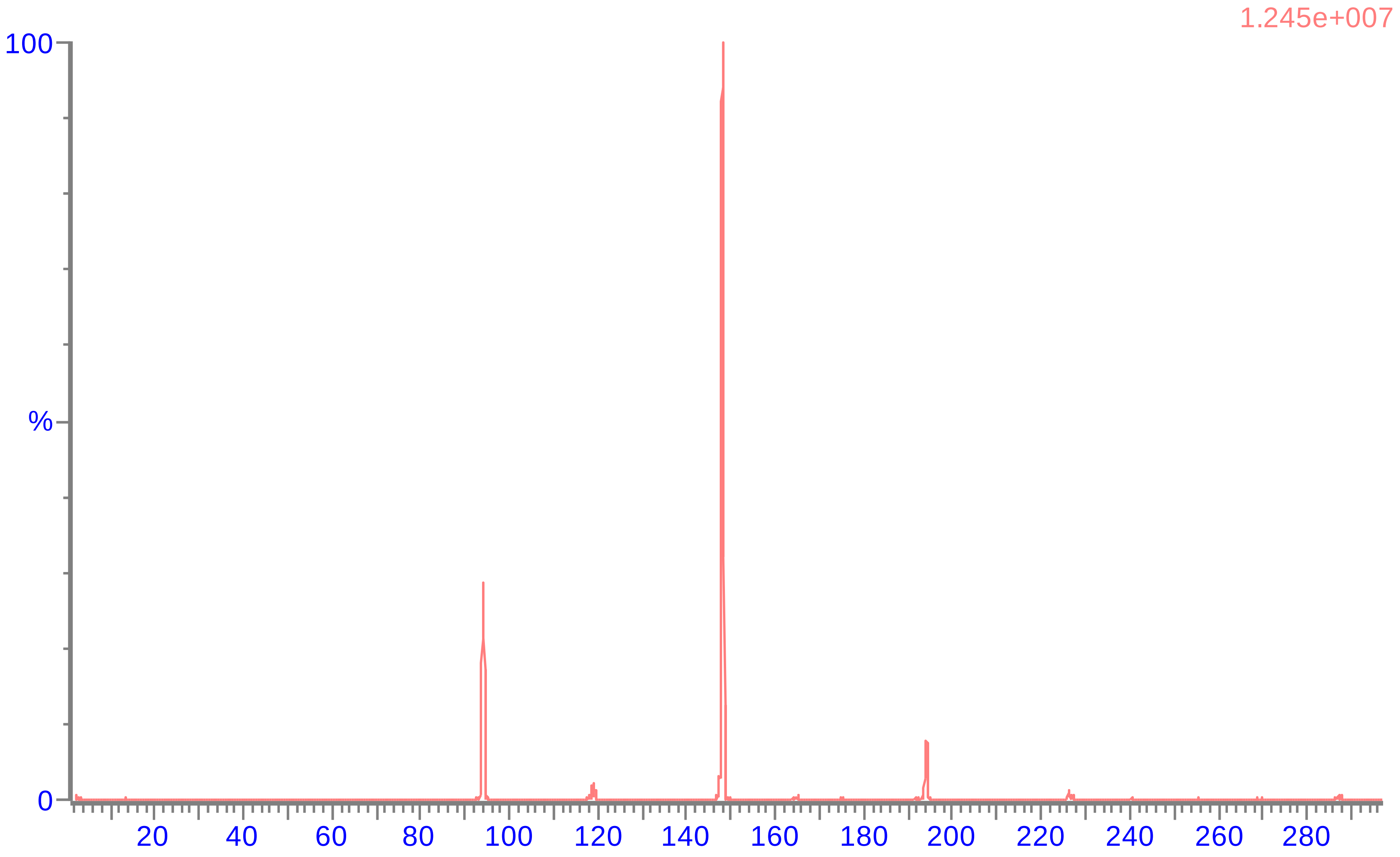

Figure 1.69: Mass spectrum for daughter fragment peak ES+, m/z 287.10 -> 148.03.

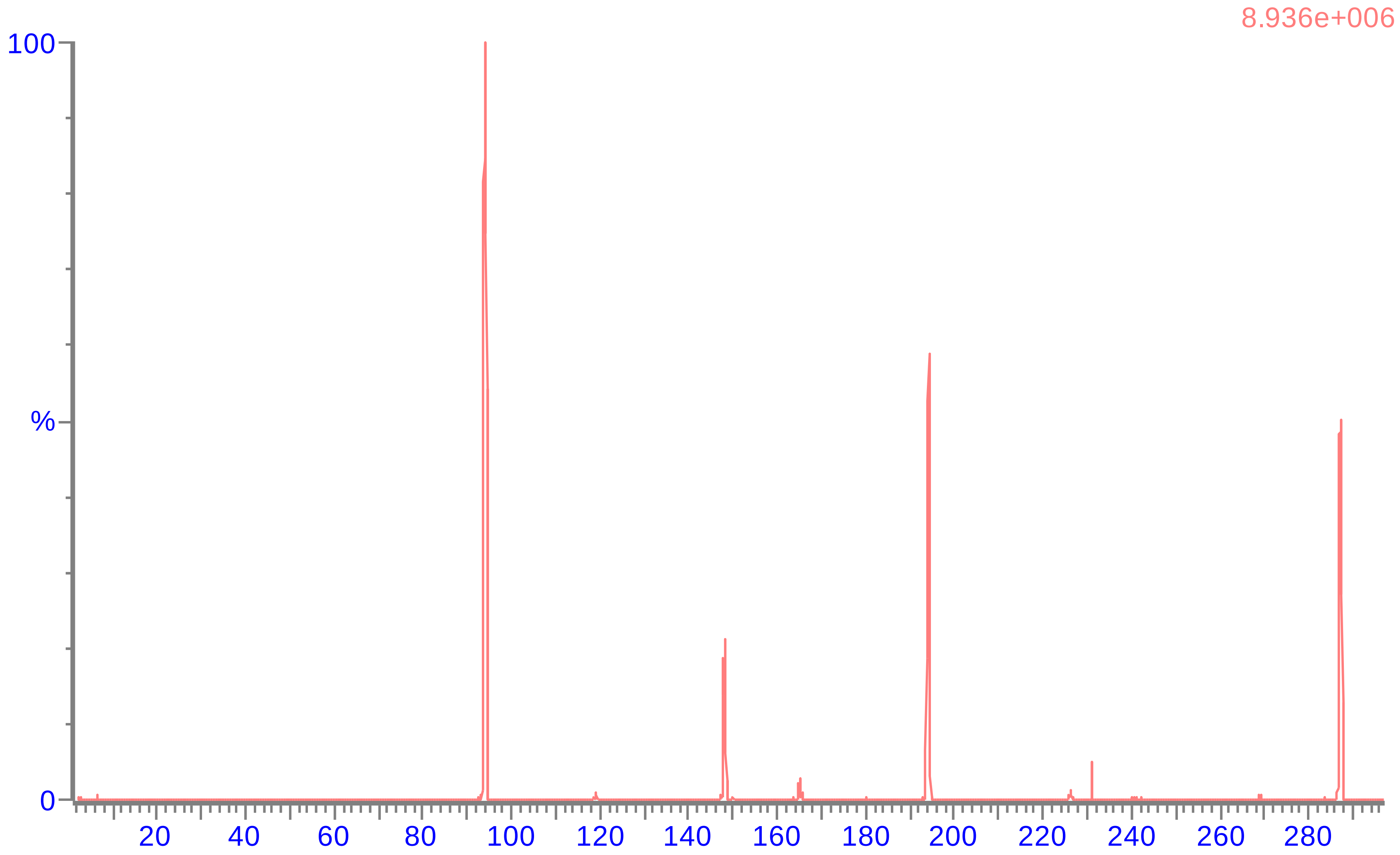

Figure18.70: Mass spectrum for daughter fragment peak ES+, m/z 287.10 -> 94.05.

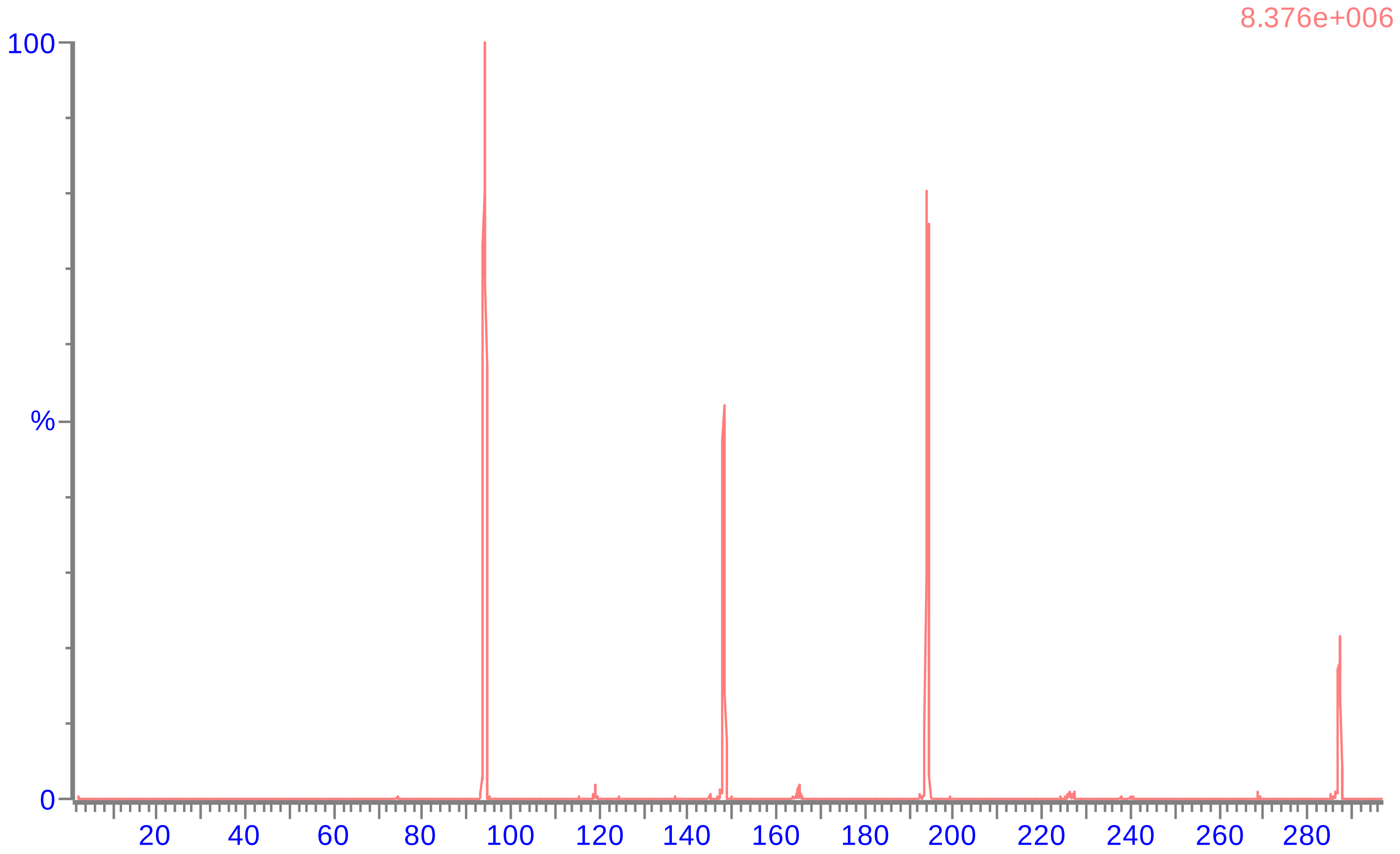

Figure 1.71: Mass spectrum for daughter fragment peak ES+, m/z 287.10 -> 194.02.

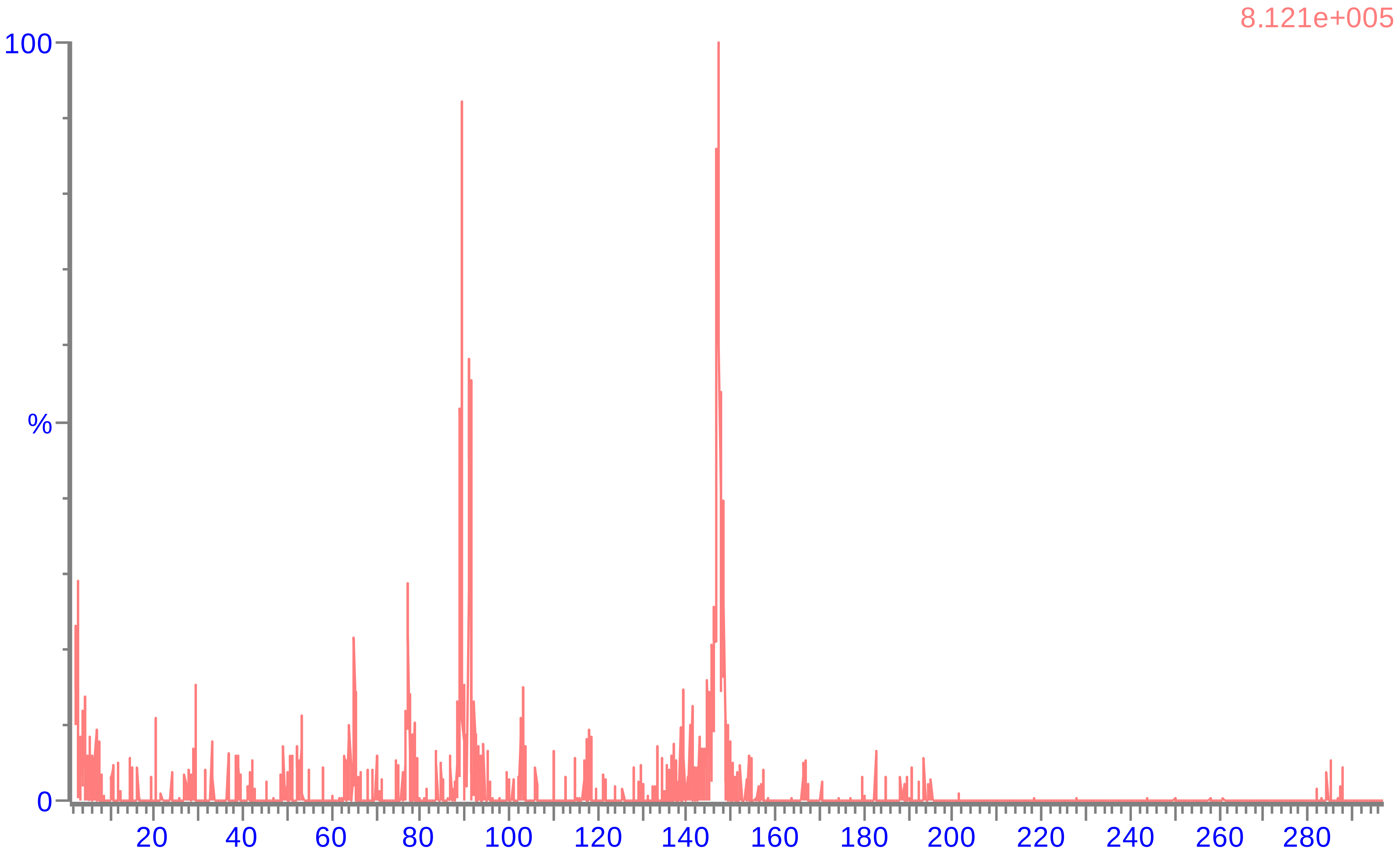

Figure1.72: Mass spectrum for daughter fragment peak ES+, m/z 287.10 -> 91.64.

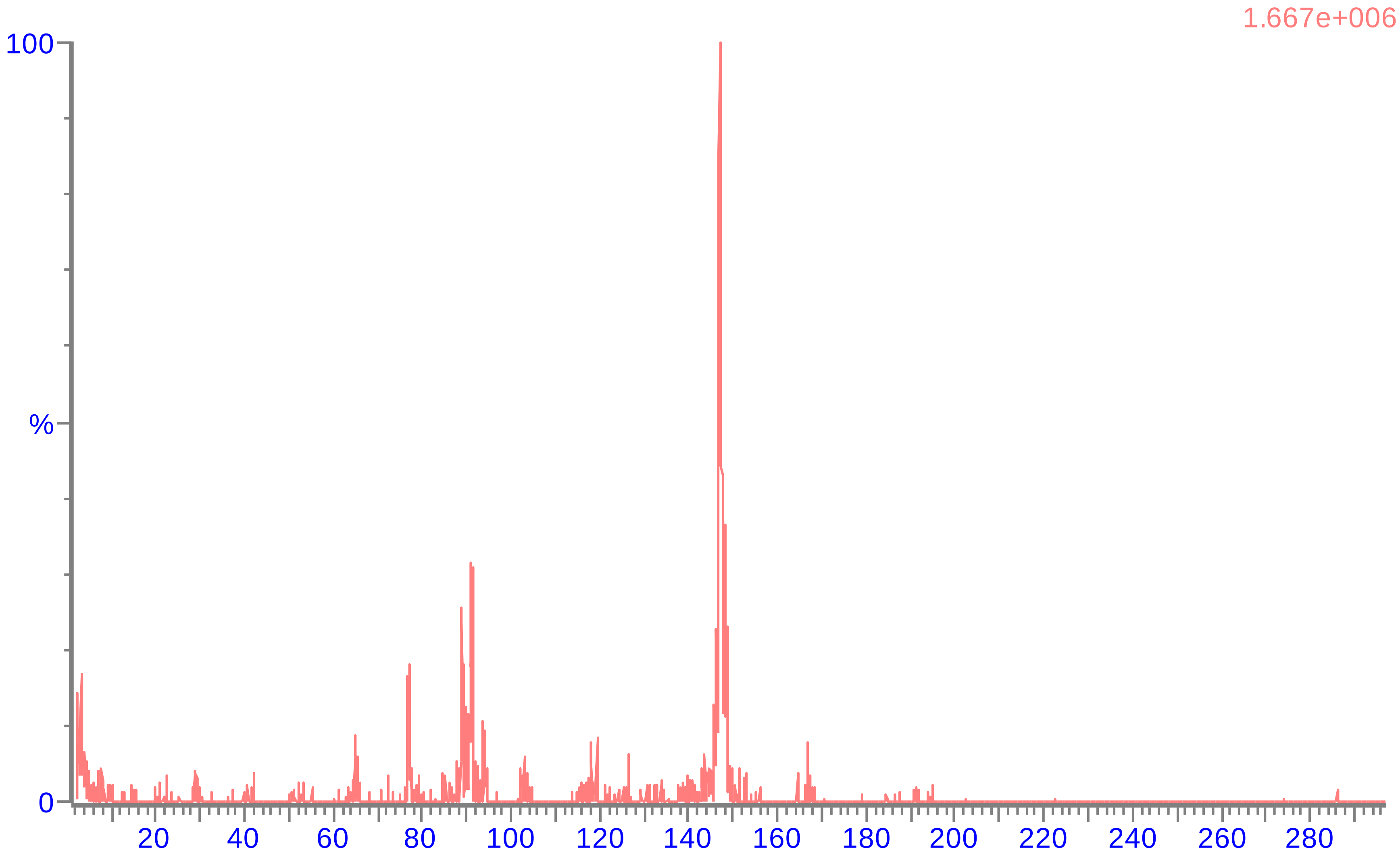

Figure 1.73: Mass spectrum for daughter fragment peak ES+, m/z 287.10 -> 90.99.

7k *N*-(1-nitropentan-2-yl)aniline.

Table 1.11: MS data.

Compound	Formula/Mass		Parent m/z	Cone Voltage	Daughters	Collision Energy	Ion Mode
**7k**	208	1	209.10	26	148.11	12	ES+
		2	209.10	26	106.02	28	ES+
		3	209.10	26	93.08	32	ES+
		4	209.10	26	118.25	36	ES+
		5	209.10	26	40.98	28	ES+

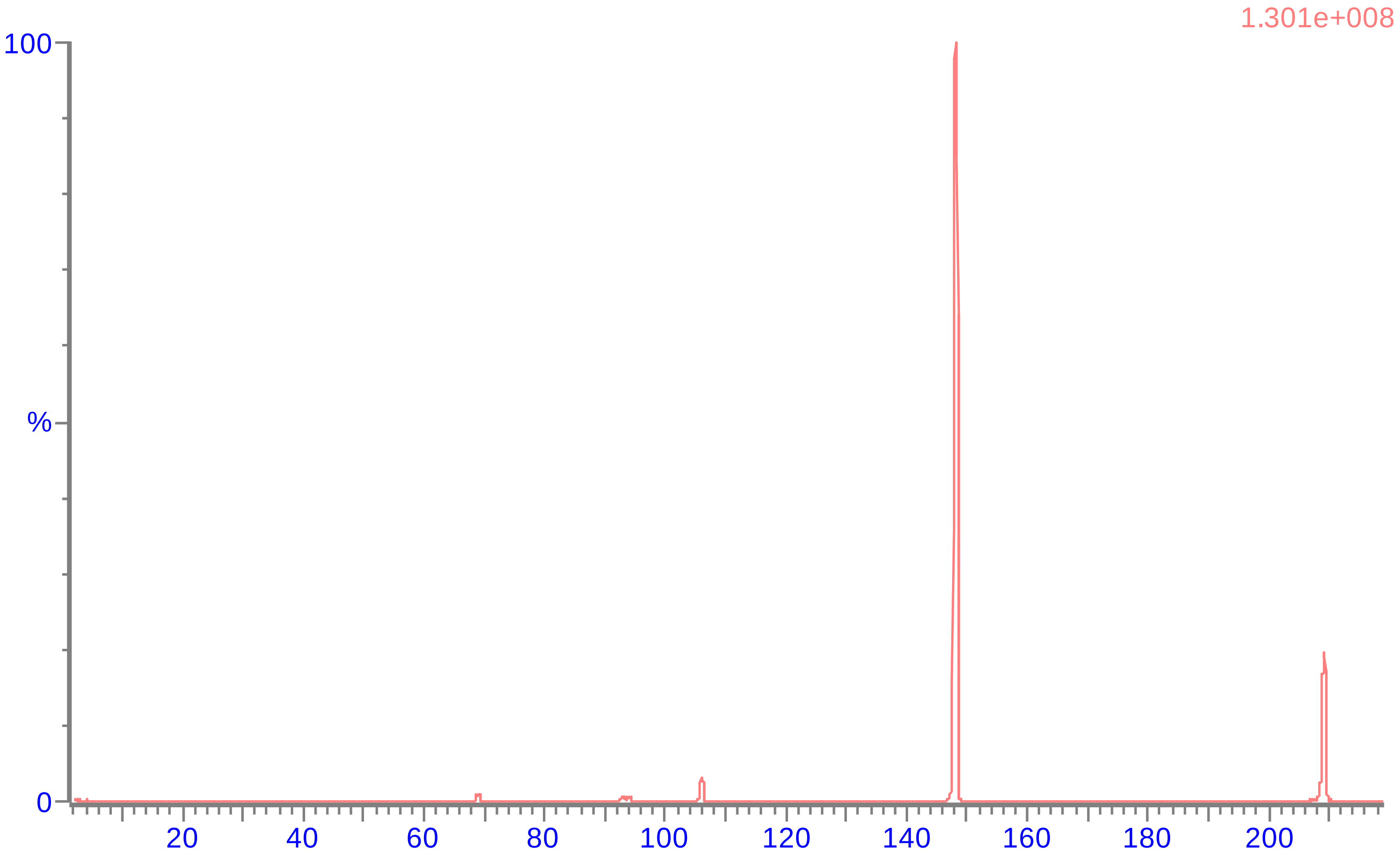

Figure 1.74: Mass spectrum for daughter fragment peak ES+, m/z 209.10 -> 148.11.

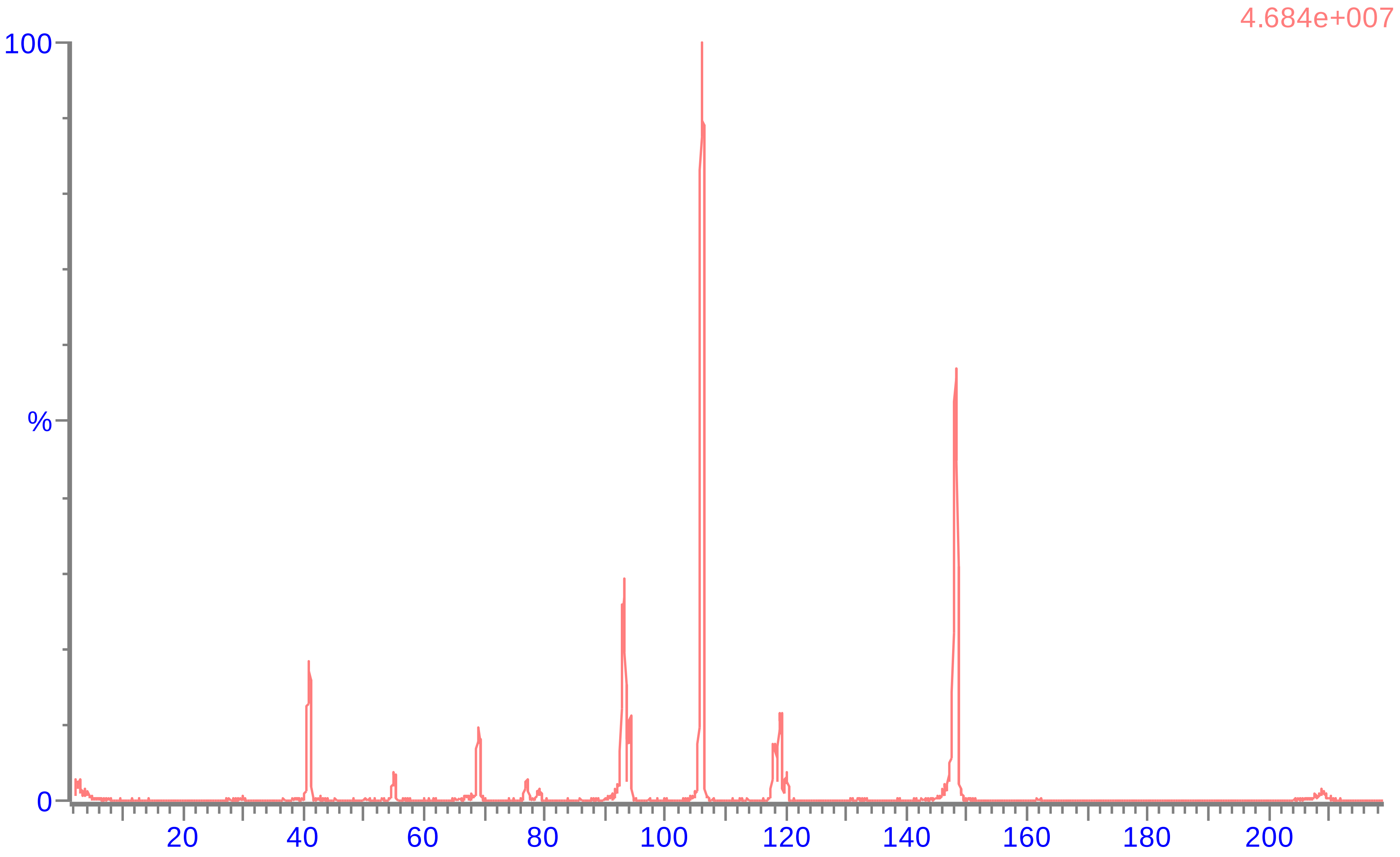

Figure 1.75: Mass spectrum for daughter fragment peak ES+, m/z 209.10 -> 106.02.

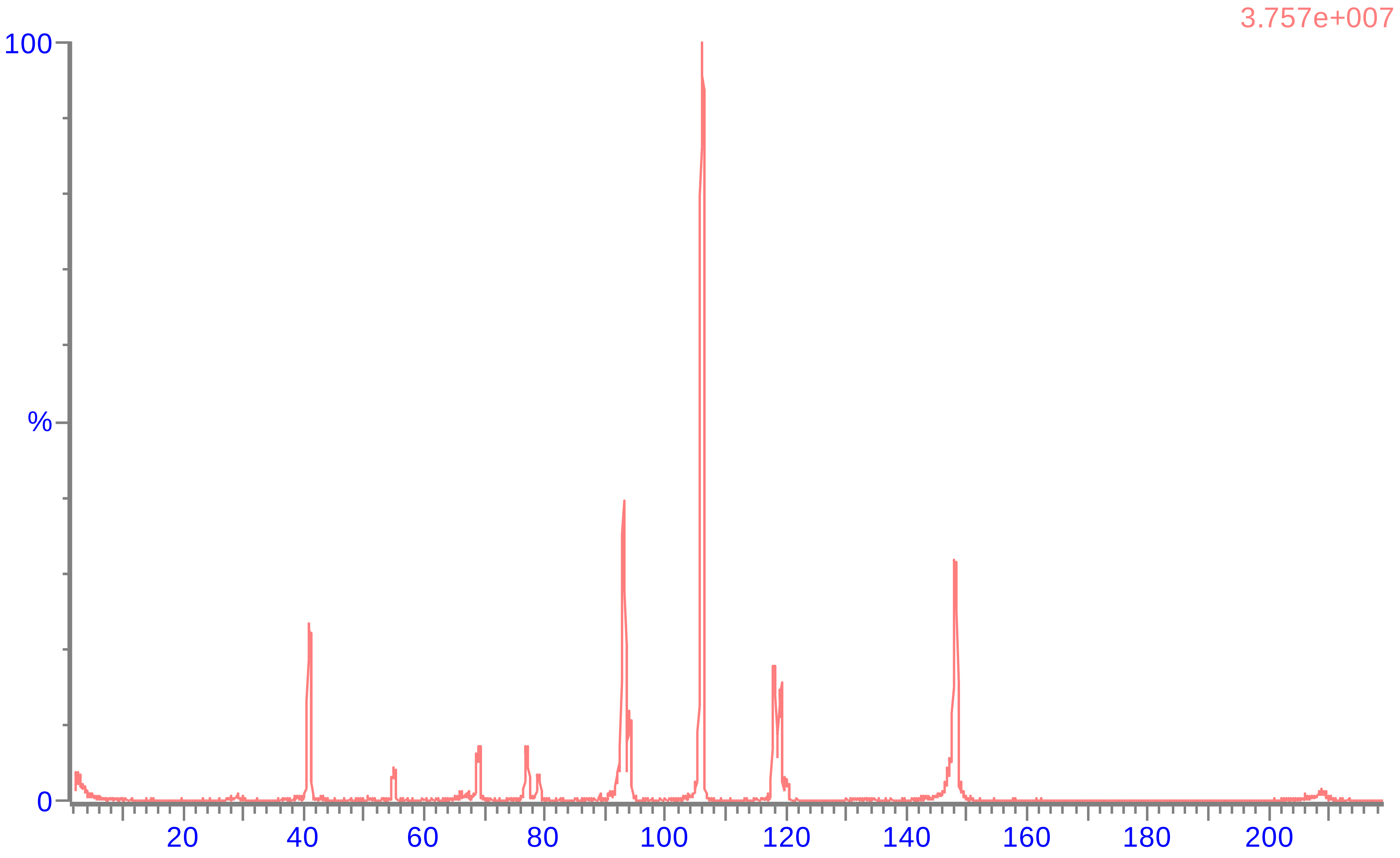

Figure 1.76: Mass spectrum for daughter fragment peak ES+, m/z 209.10 -> 93.08.

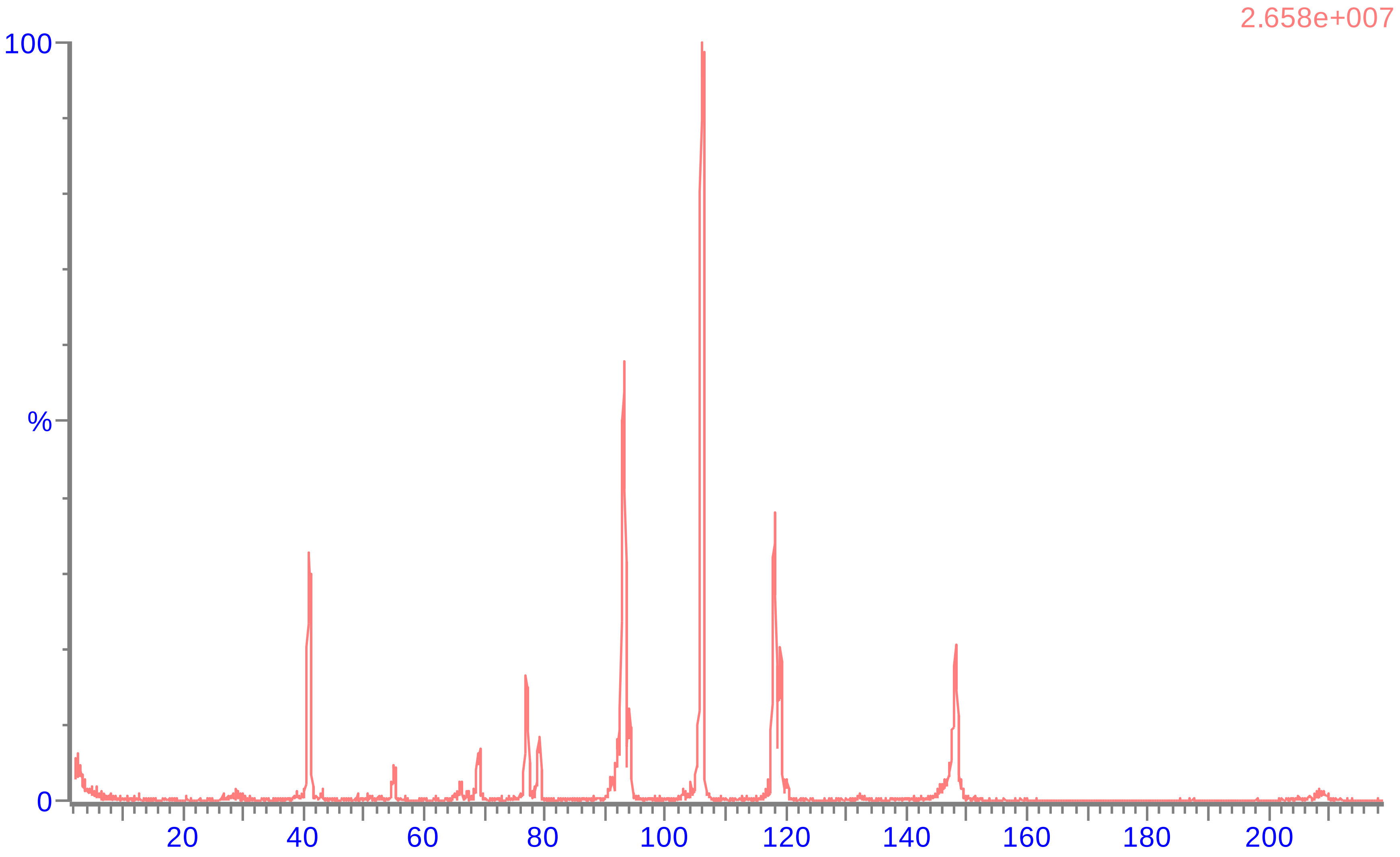

Figure 1.77: Mass spectrum for daughter fragment peak ES+, m/z 209.10 -> 118.25.

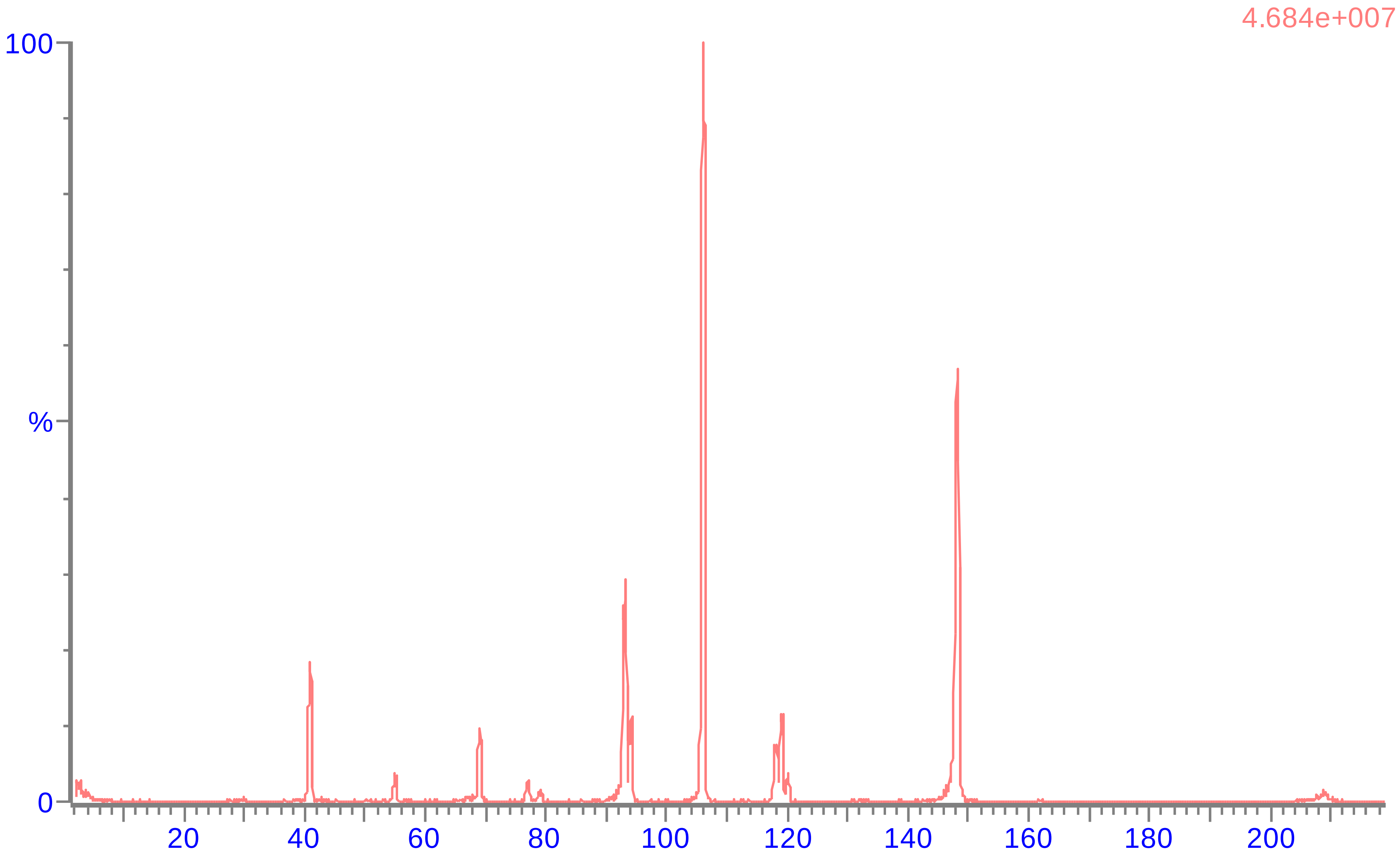

Figure 1.78: Mass spectrum for daughter fragment peak ES+, m/z 209.10 -> 40.98.

<u>**7l** *N*-(1-cyclohexyl-2-nitroethyl)aniline</u>

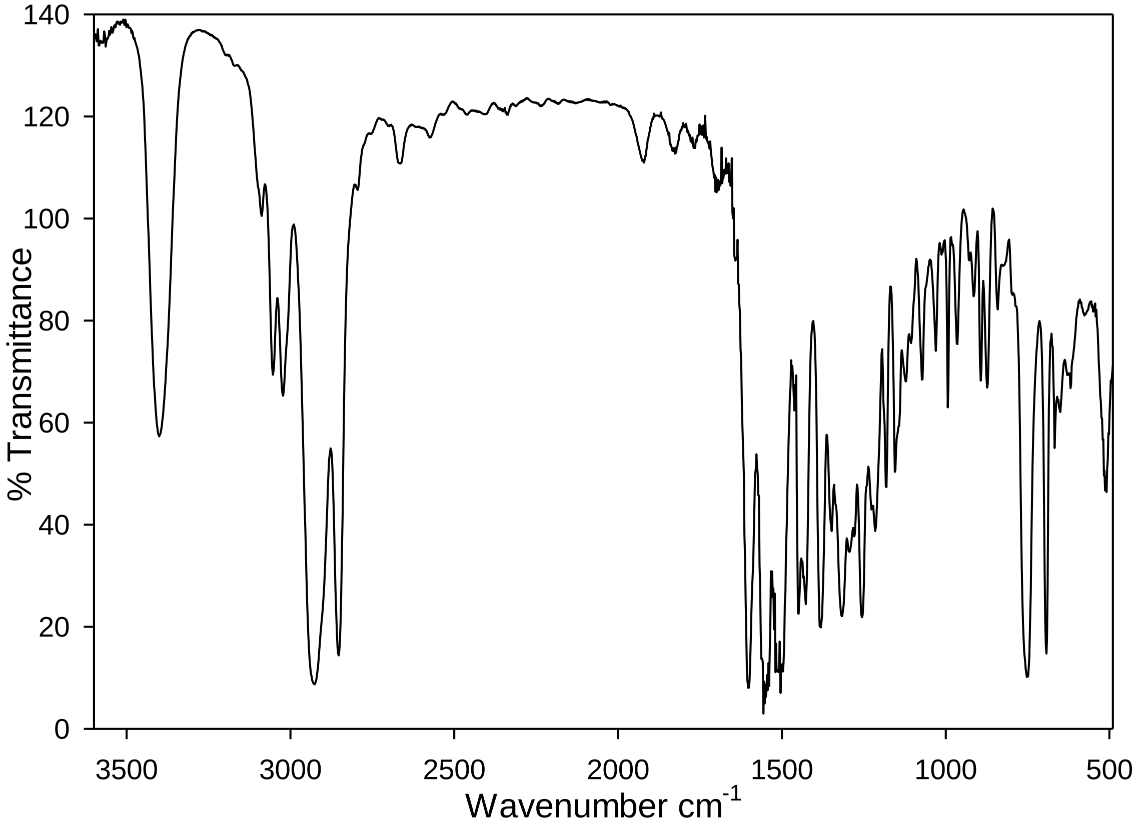

Figure 1.79: IR spectrum of **7l** *N*-(1-cyclohexyl-2-nitroethyl)aniline.

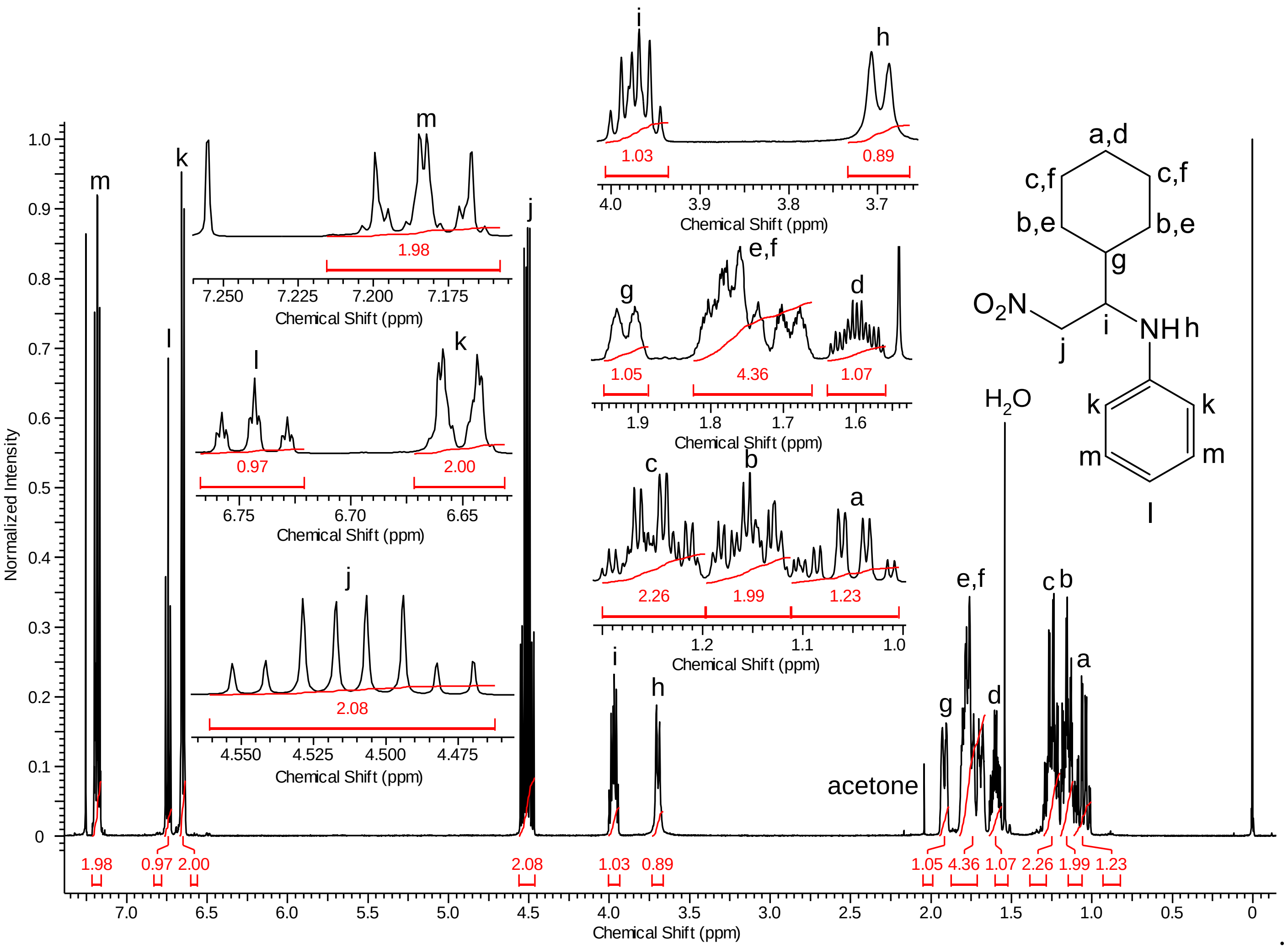

Figure 1.80: ^{1}H NMR spectrum of **7l** *N*-(1-cyclohexyl-2-nitroethyl)aniline.

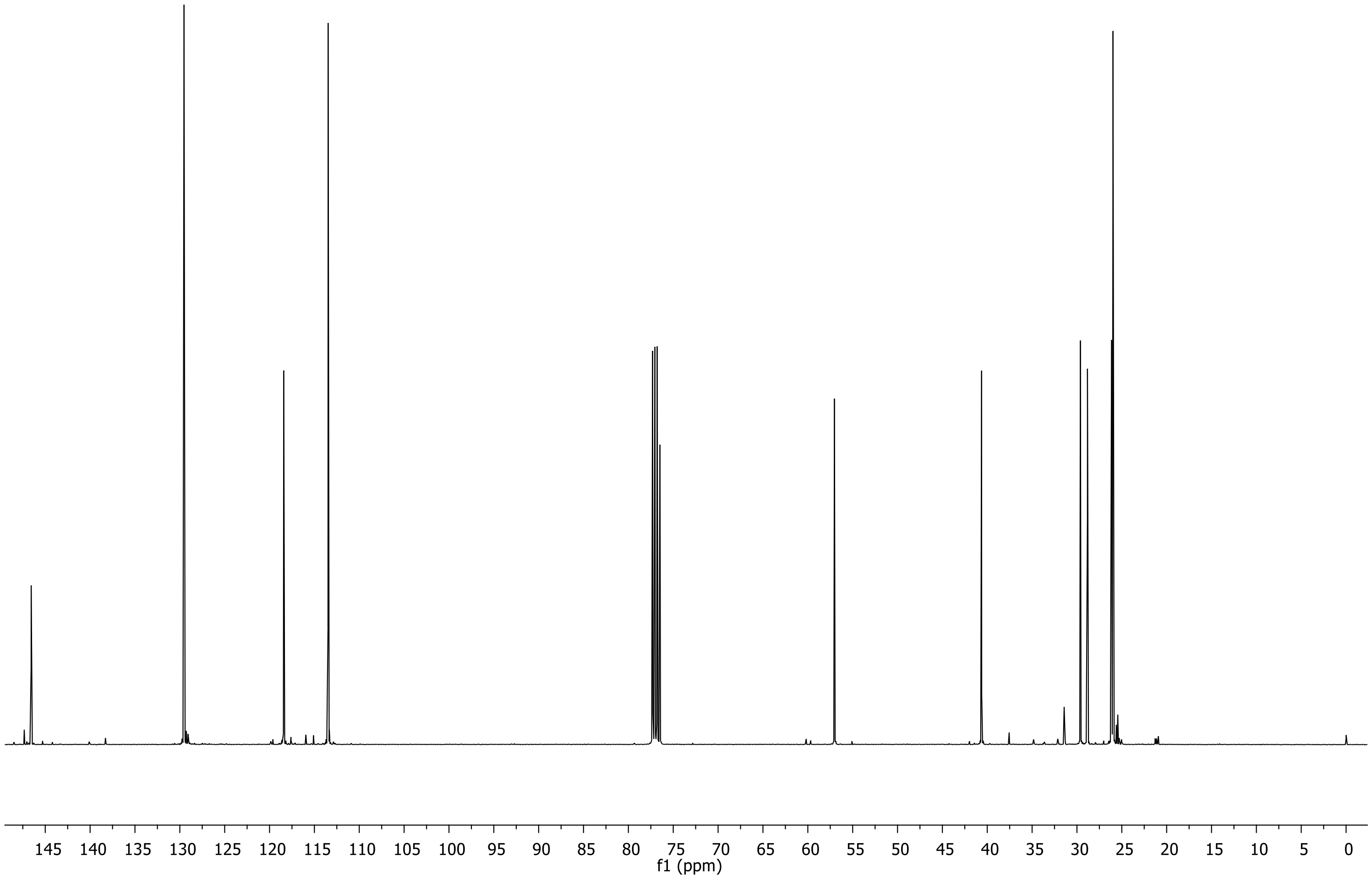

Figure 1.81: ^{13}C NMR spectrum of **7l** *N*-(1-cyclohexyl-2-nitroethyl)aniline.

Table 1.12: MS data.

Compound	Formula/Mass		Parent m/z	Cone Voltage	Daughters	Collision Energy	Ion Mode
**7l**	248.3	1	249.14	14	188.17	18	ES+
		2	249.14	14	106.02	28	ES+
		3	249.14	14	67.06	34	ES+
		4	249.14	14	94.71	28	ES+
		5	249.14	14	109.08	20	ES+

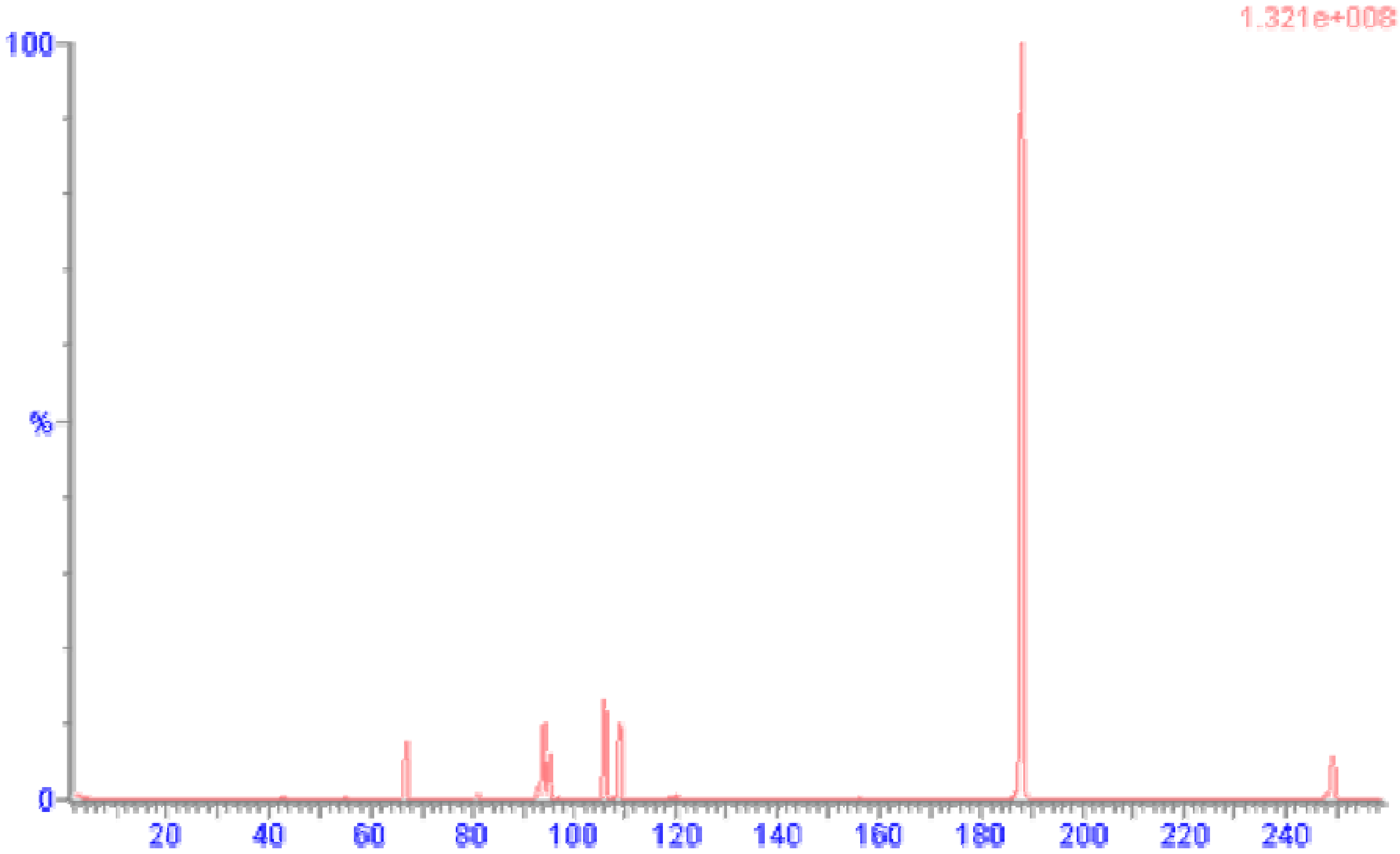

Figure 1.82: Mass spectrum for daughter fragment peak ES+, m/z 249.14 -> 188.17.

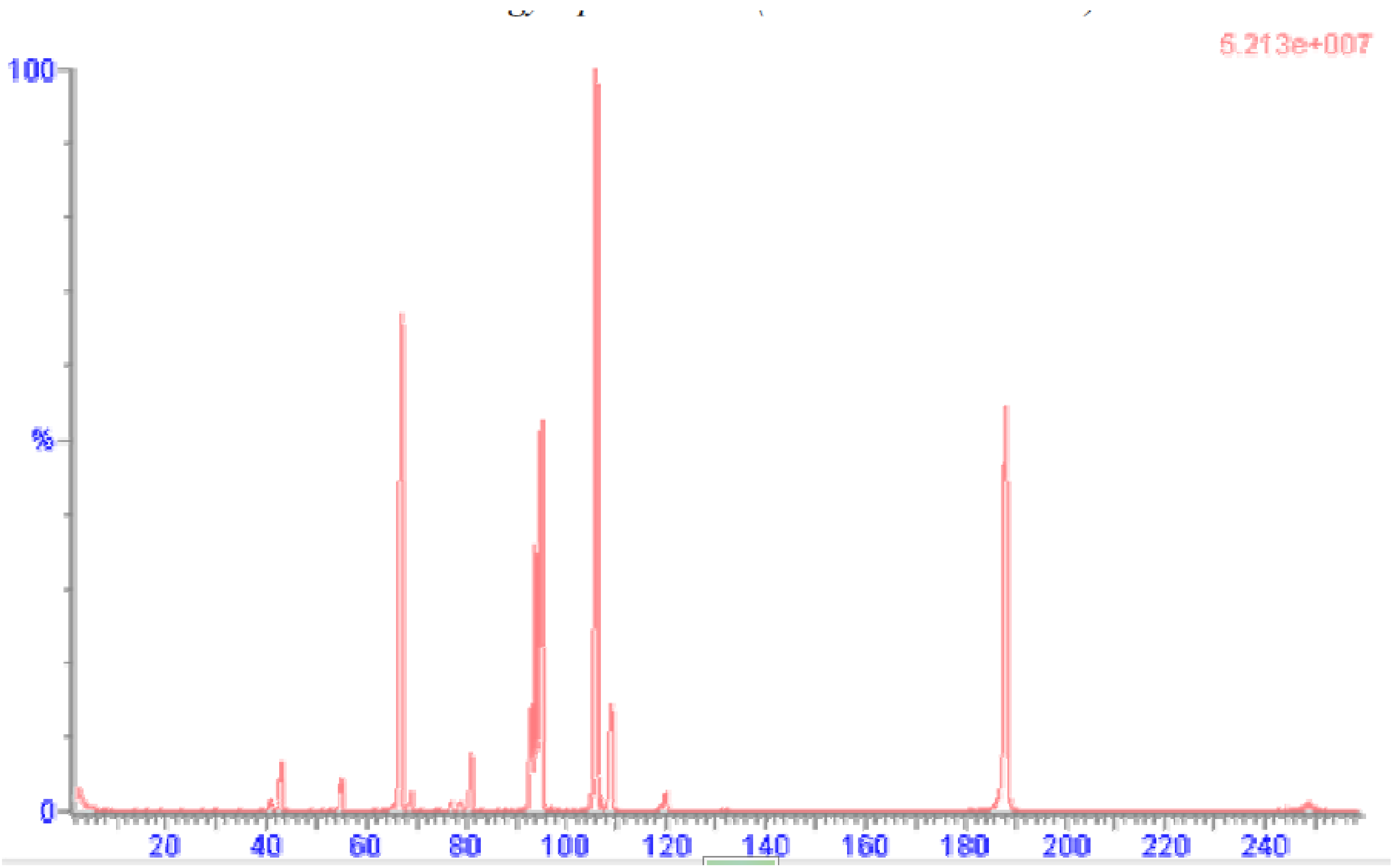

Figure 1.83: Mass spectrum for daughter fragment peak ES+, m/z 249.14 -> 106.02.

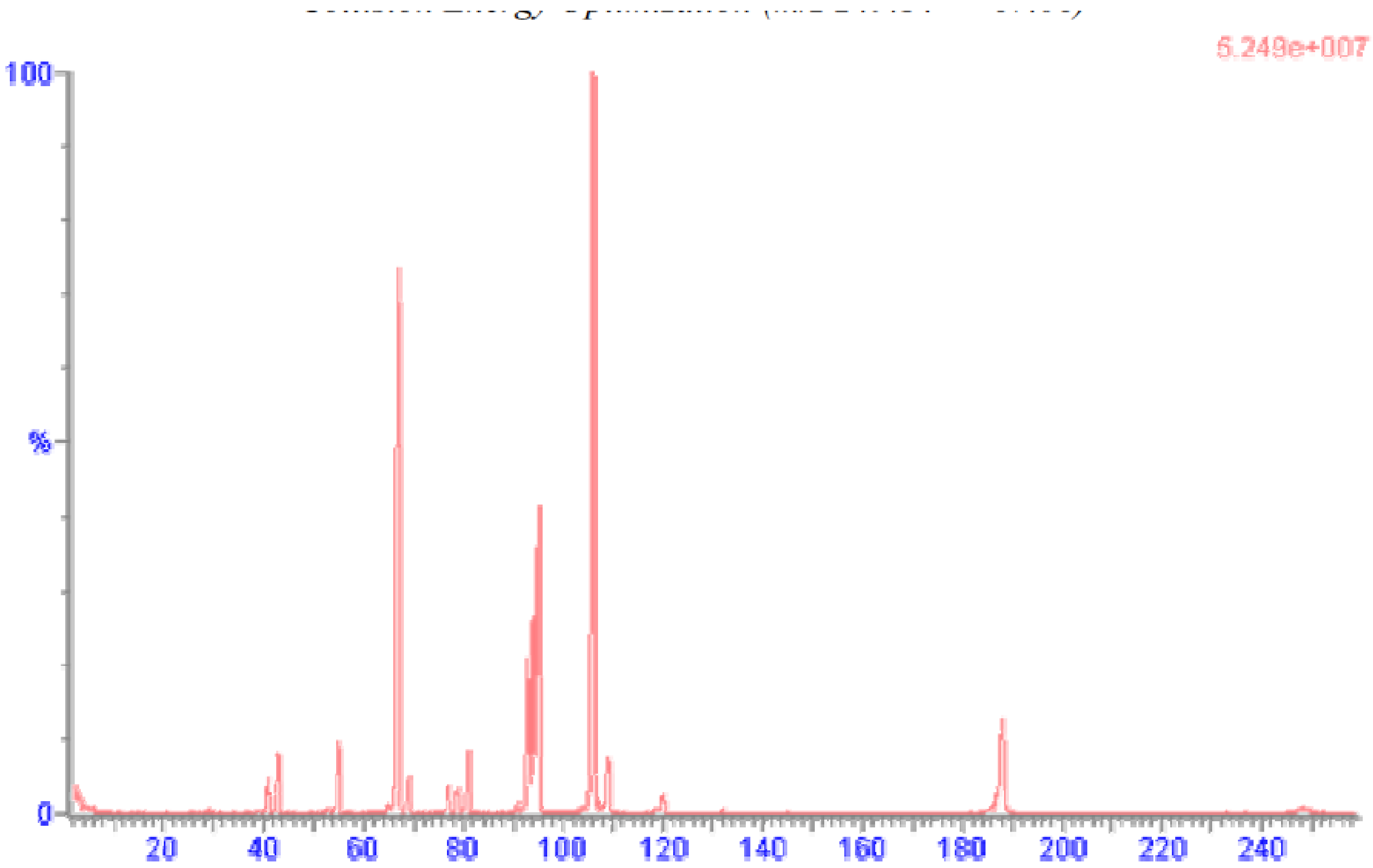

Figure 1.84: Mass spectrum for daughter fragment peak ES+, m/z 249.14 -> 67.06.

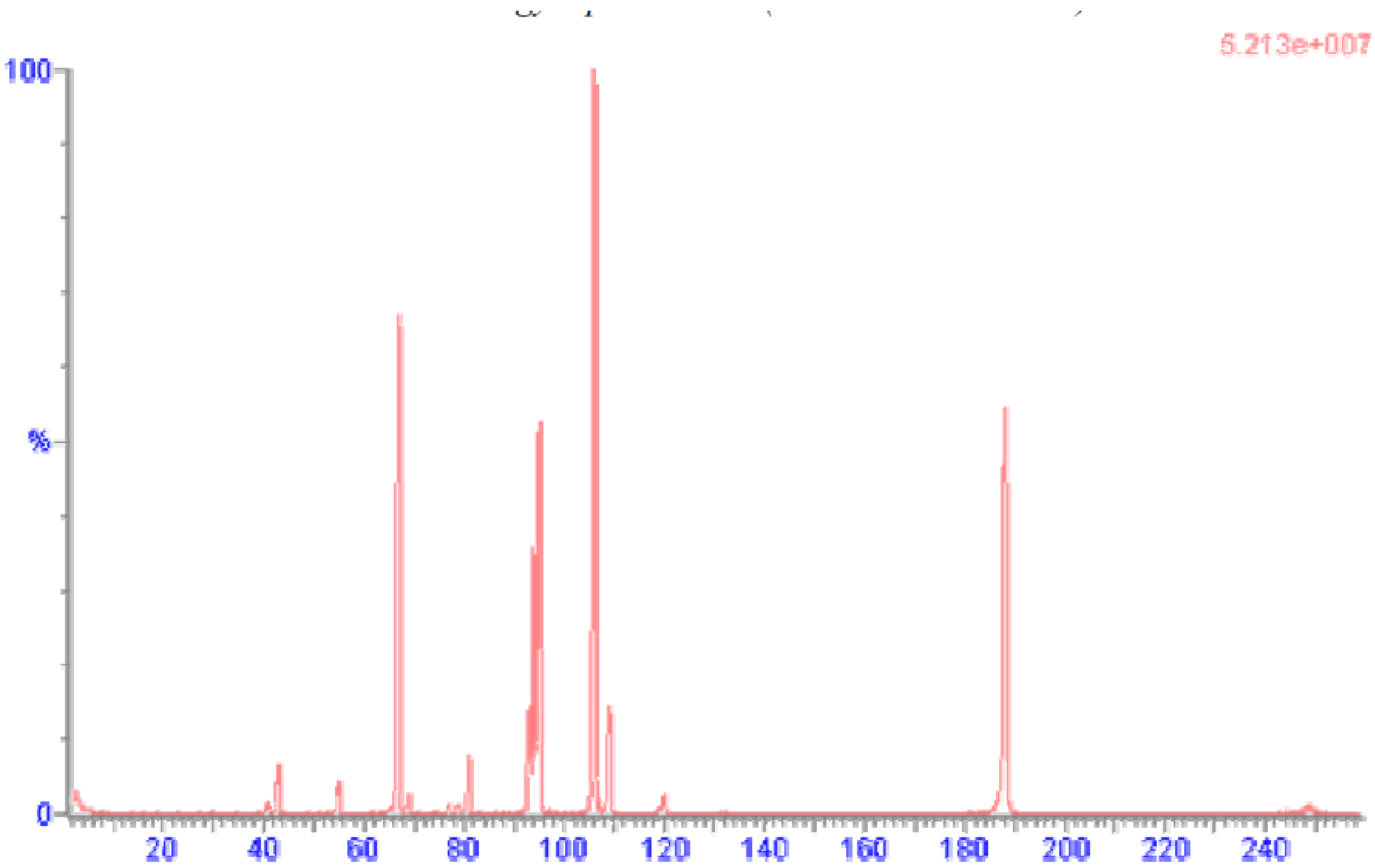

Figure 1.85: Mass spectrum for daughter fragment peak ES+, m/z 249.14 -> 94.71.

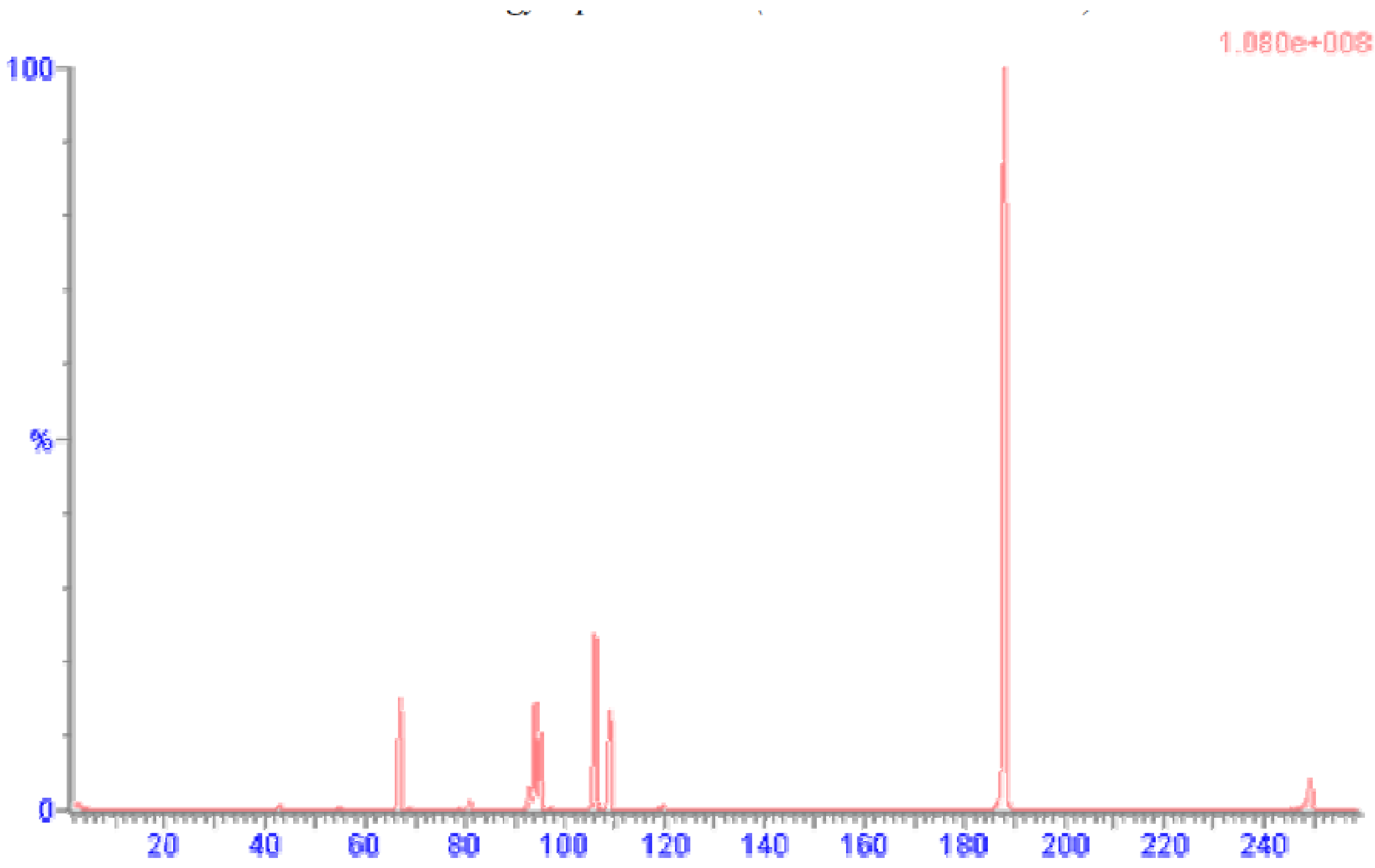

Figure 1.86: Mass spectrum for daughter fragment peak ES+, m/z 249.14 -> 109.08.

7m ethyl 3-nitro-2-(phenylamino)propanoate

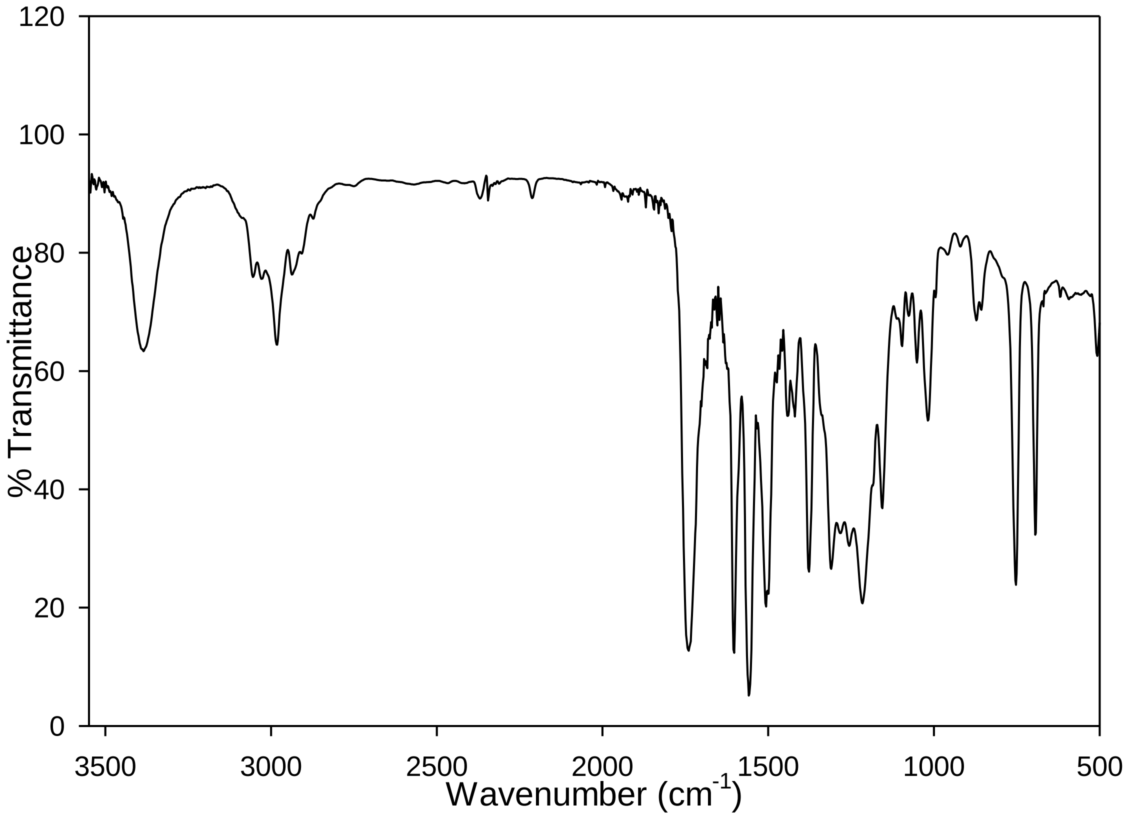

Figure 1.87: IR spectrum of **7m** ethyl 3-nitro-2-(phenylamino)propanoate.

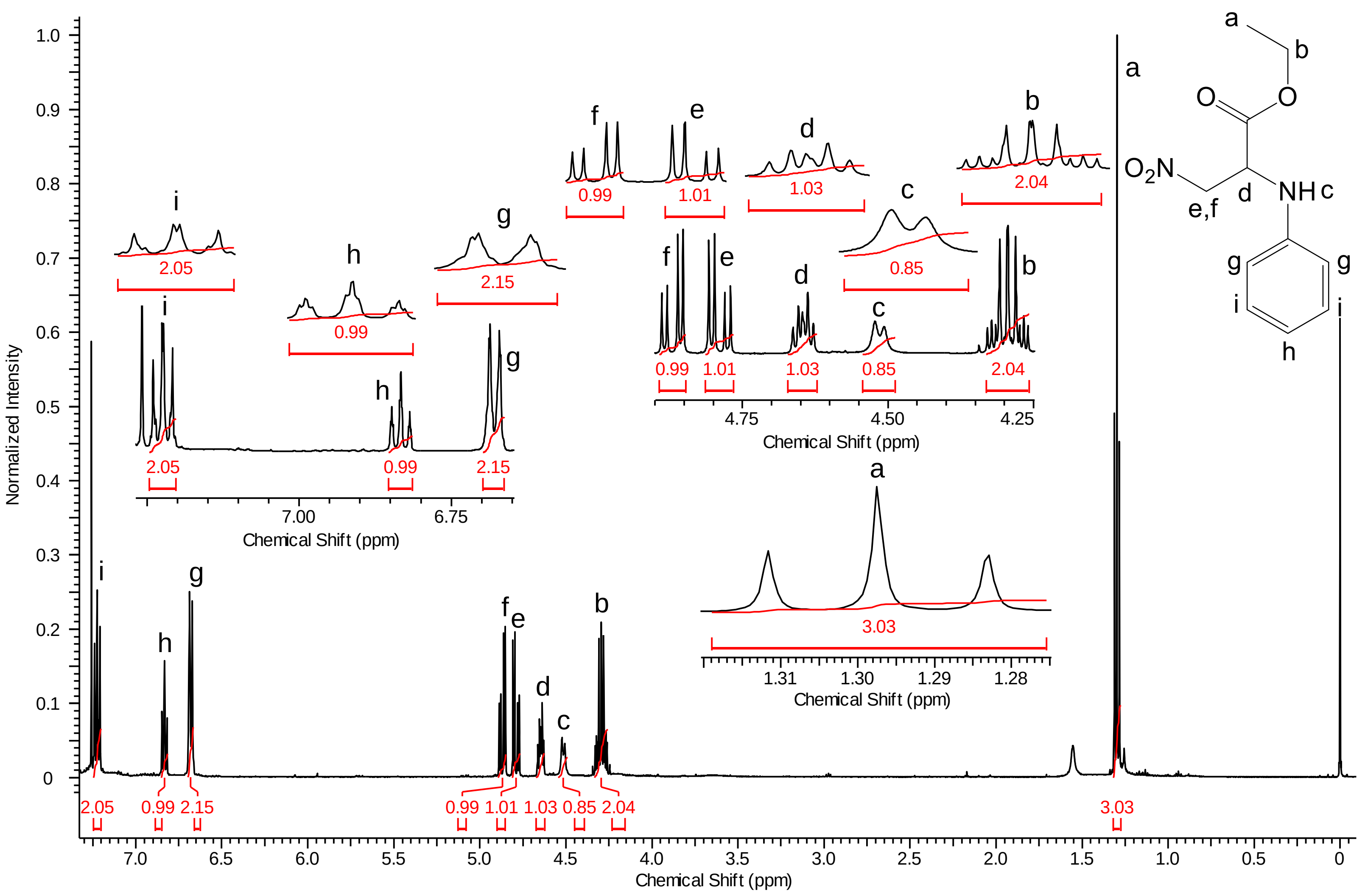

Figure 1.88: ^{1}H NMR spectrum of **7m** ethyl 3-nitro-2-(phenylamino)propanoate.

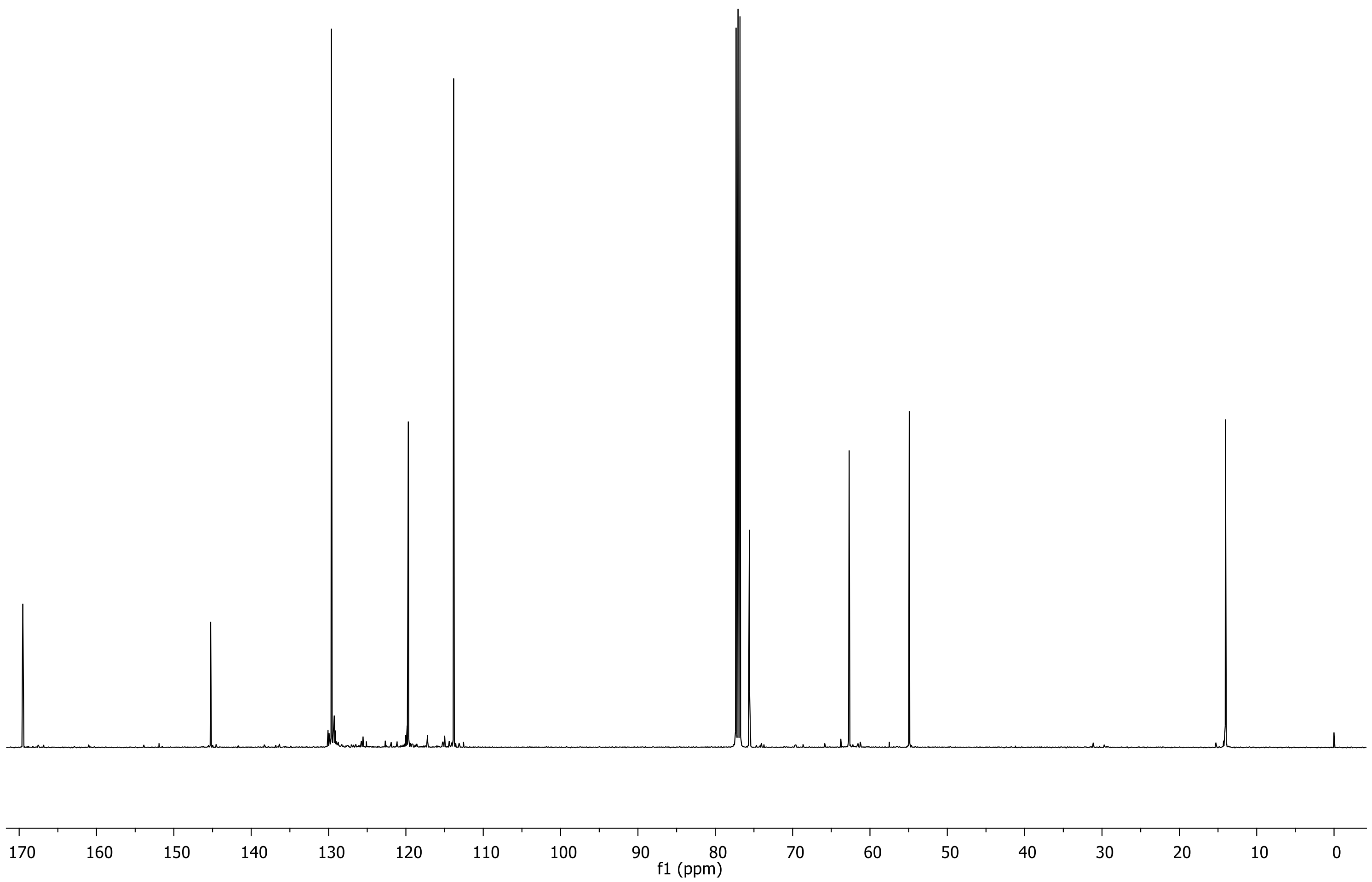

Figure 1.89: ^{13}C NMR spectrum of **7m** ethyl 3-nitro-2-(phenylamino)propanoate.

Table 1.13: MS data.

Compound	Formula/Mass		Parent m/z	Cone Voltage	Daughters	Collision Energy	Ion Mode
**7m**	238	1	239.10	20	178.11	10	ES+
		2	239.10	20	104.02	26	ES+
		3	239.10	20	77.02	40	ES+
		4	239.10	20	93.02	38	ES+
		5	239.10	20	150.07	18	ES+

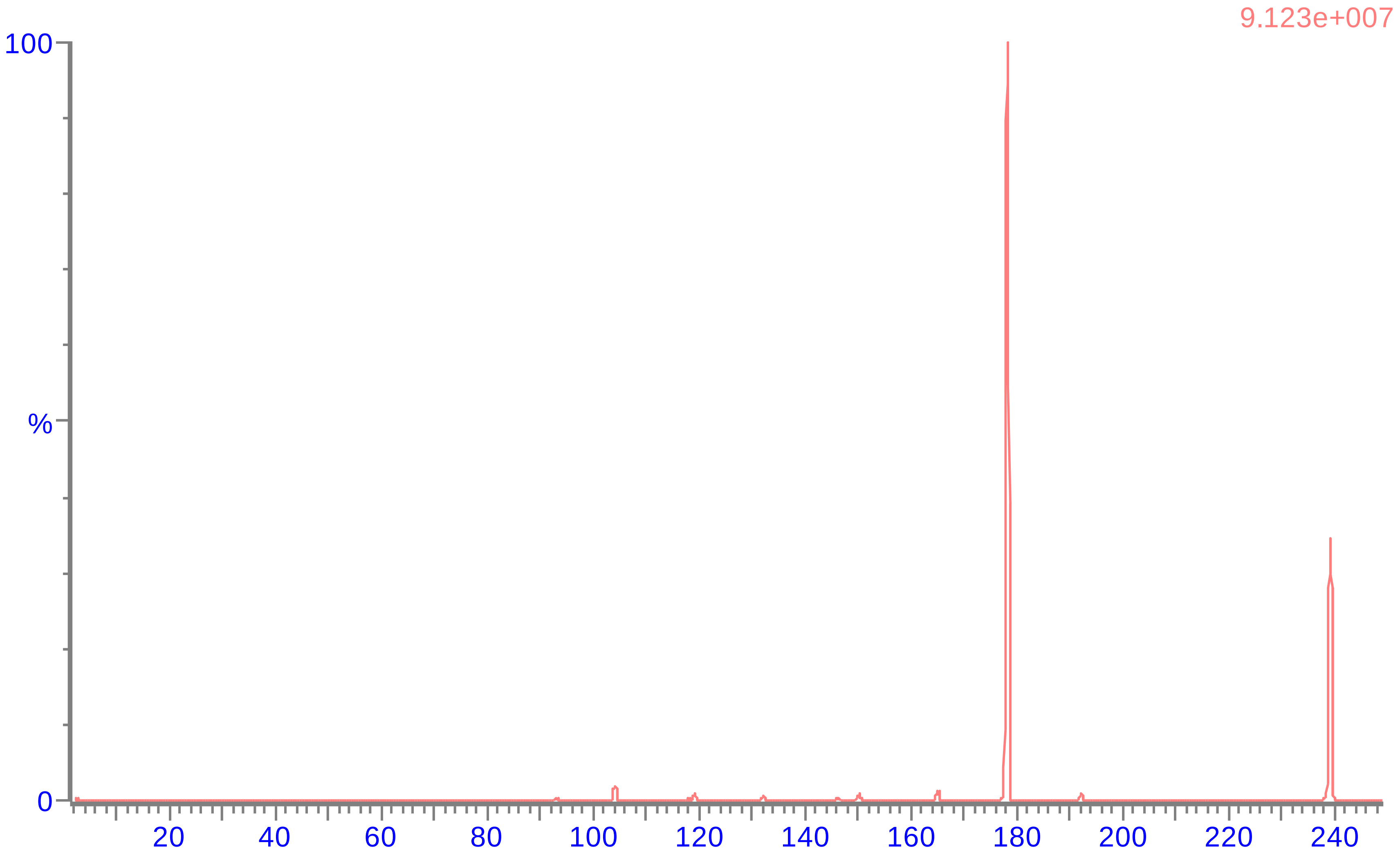

Figure 1.90: Mass spectrum for daughter fragment peak ES+, m/z 239.10 -> 178.11.

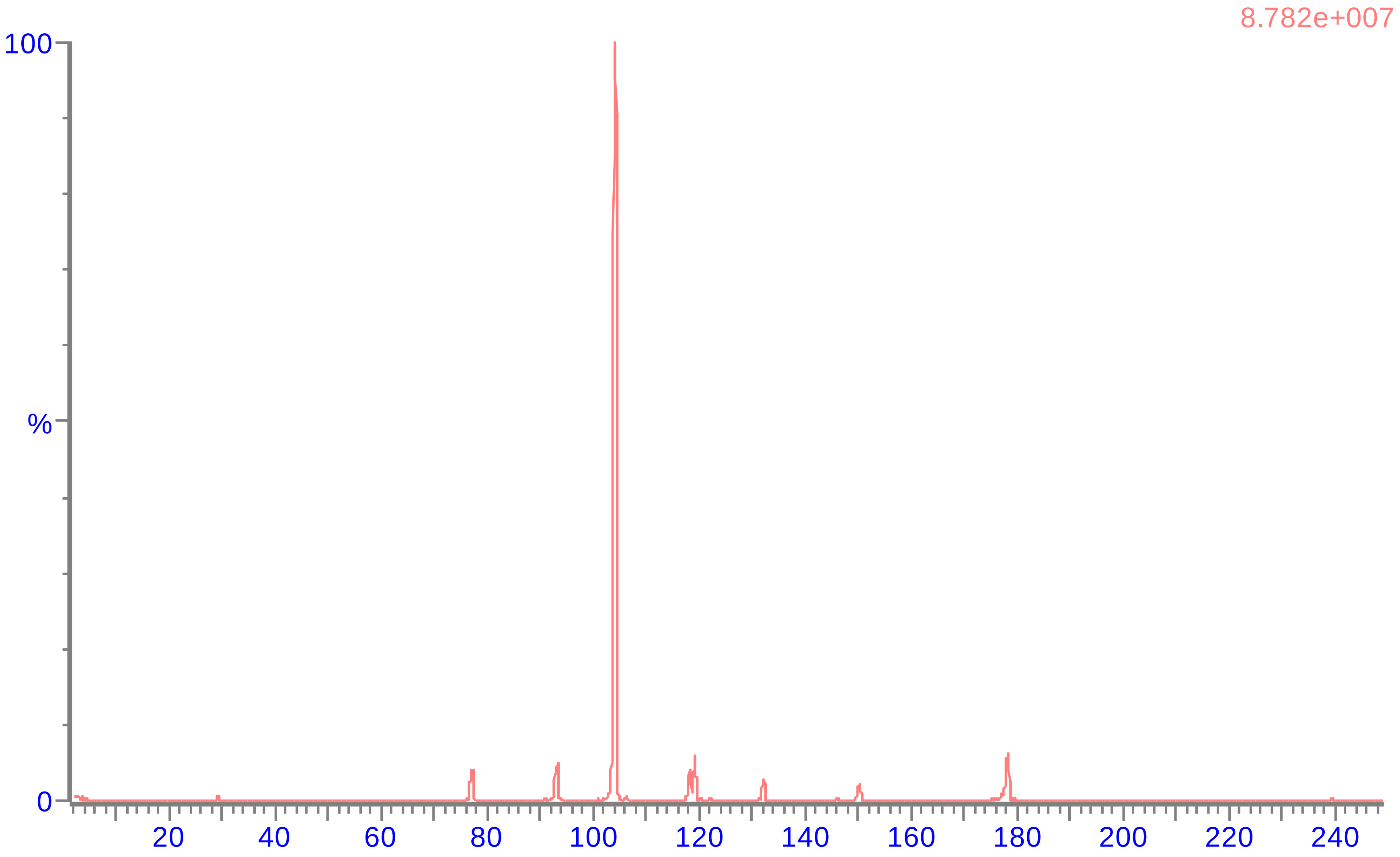

Figure 1.91: Mass spectrum for daughter fragment peak ES+, m/z 239.10 -> 104.02.

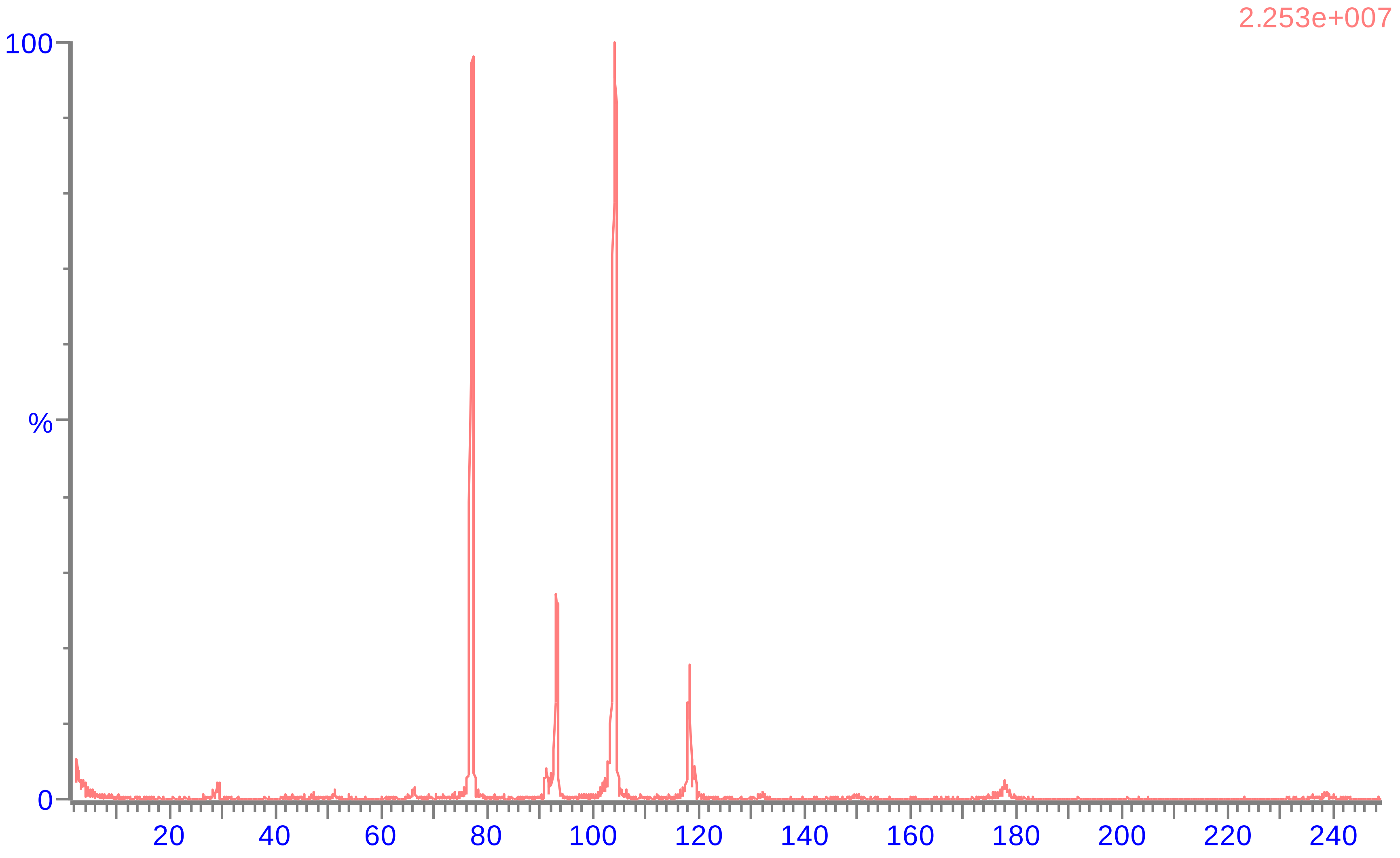

Figure 1.92: Mass spectrum for daughter fragment peak ES+, m/z 239.10 -> 77.02.

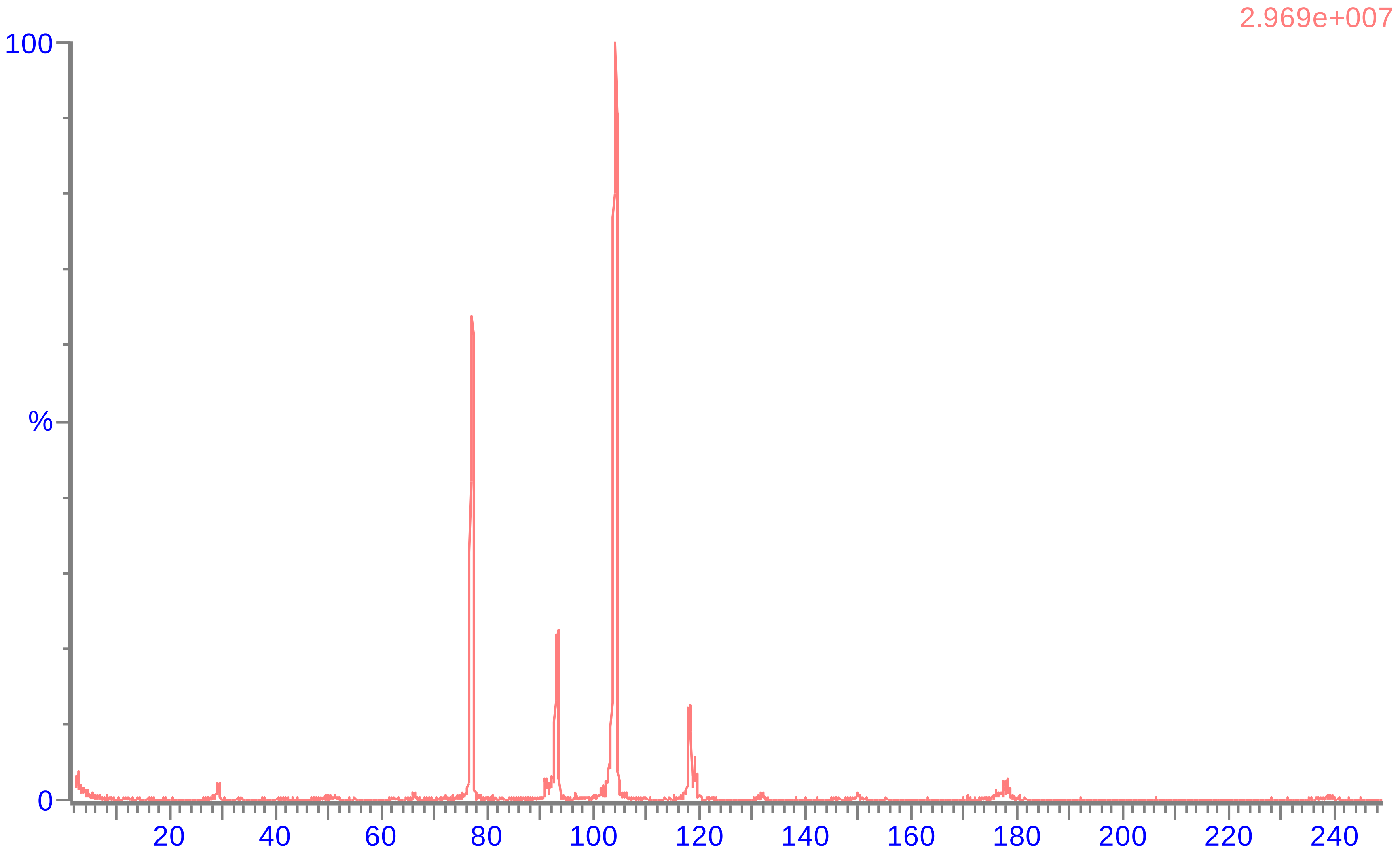

Figure 1.93: Mass spectrum for daughter fragment peak ES+, m/z 239.10 -> 93.02.

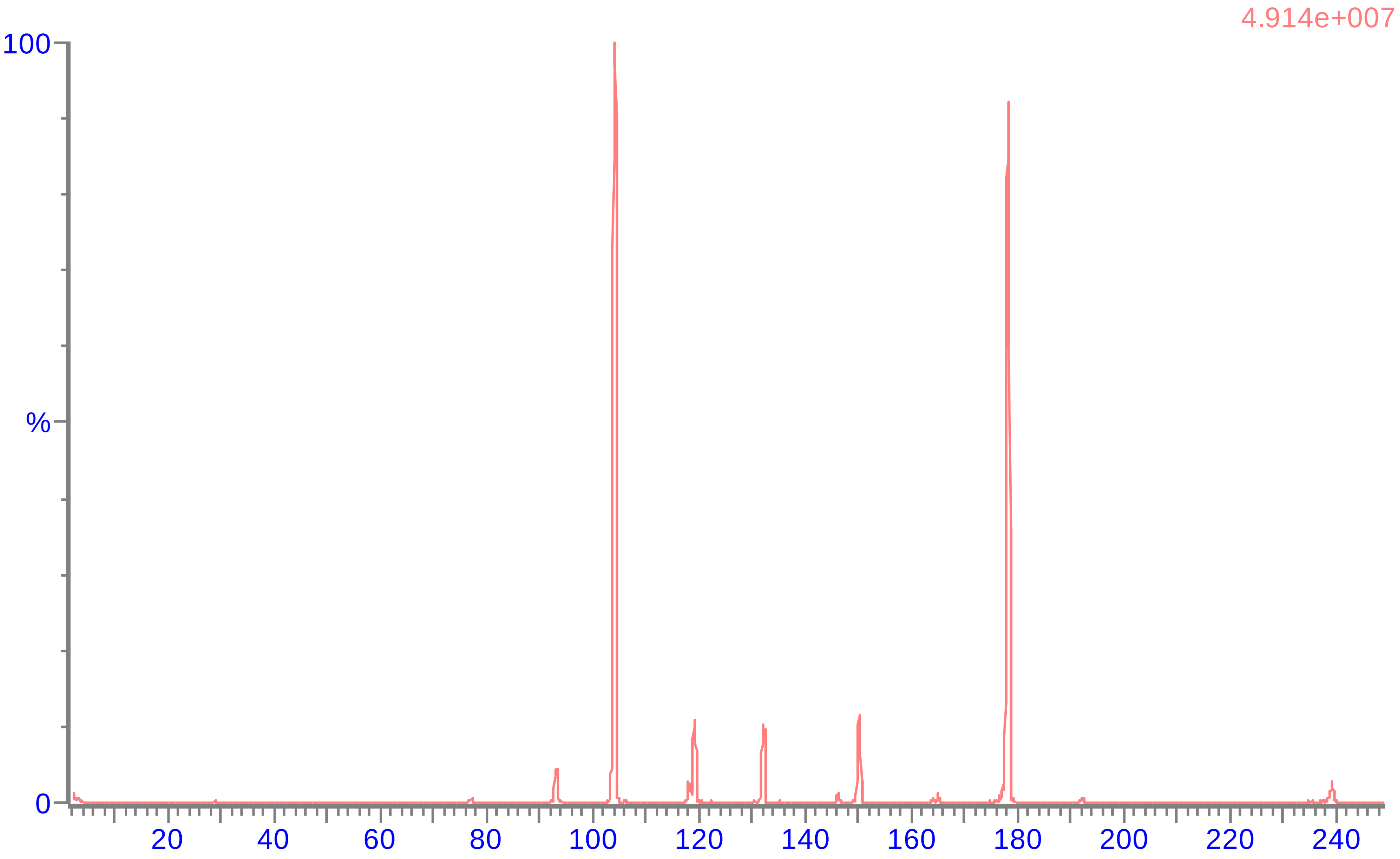

Figure 1.94: Mass spectrum for daughter fragment peak ES+, m/z 239.10 -> 150.07.

8a 4-methoxy-*N*-(2-nitro-1-phenylethyl)aniline

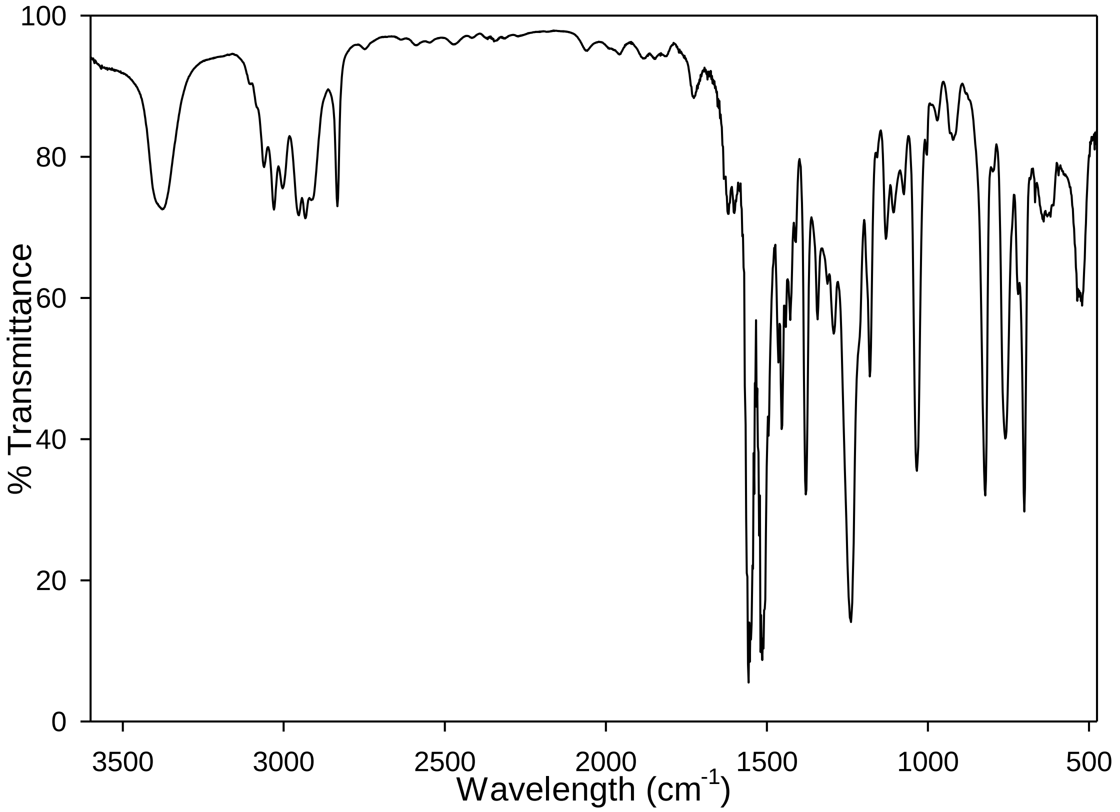

Figure 1.95: IR spectrum of **8a** 4-methoxy-*N*-(2-nitro-1-phenylethyl)aniline.

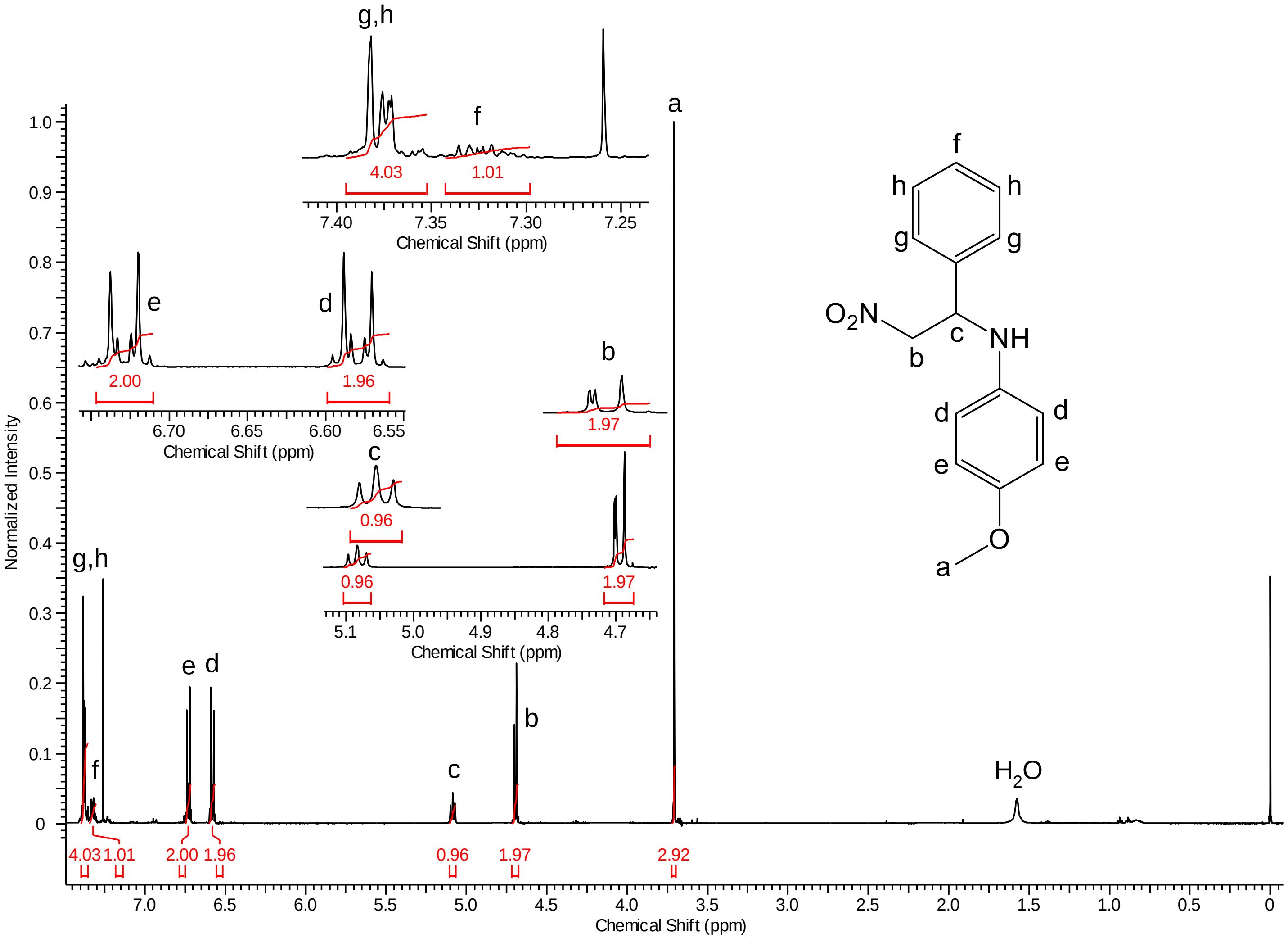

Figure 1.96: ^{1}H NMR spectrum of **8a** 4-methoxy-*N*-(2-nitro-1-phenylethyl)aniline.

8b 4-methyl-*N*-(2-nitro-1-phenylethyl)aniline

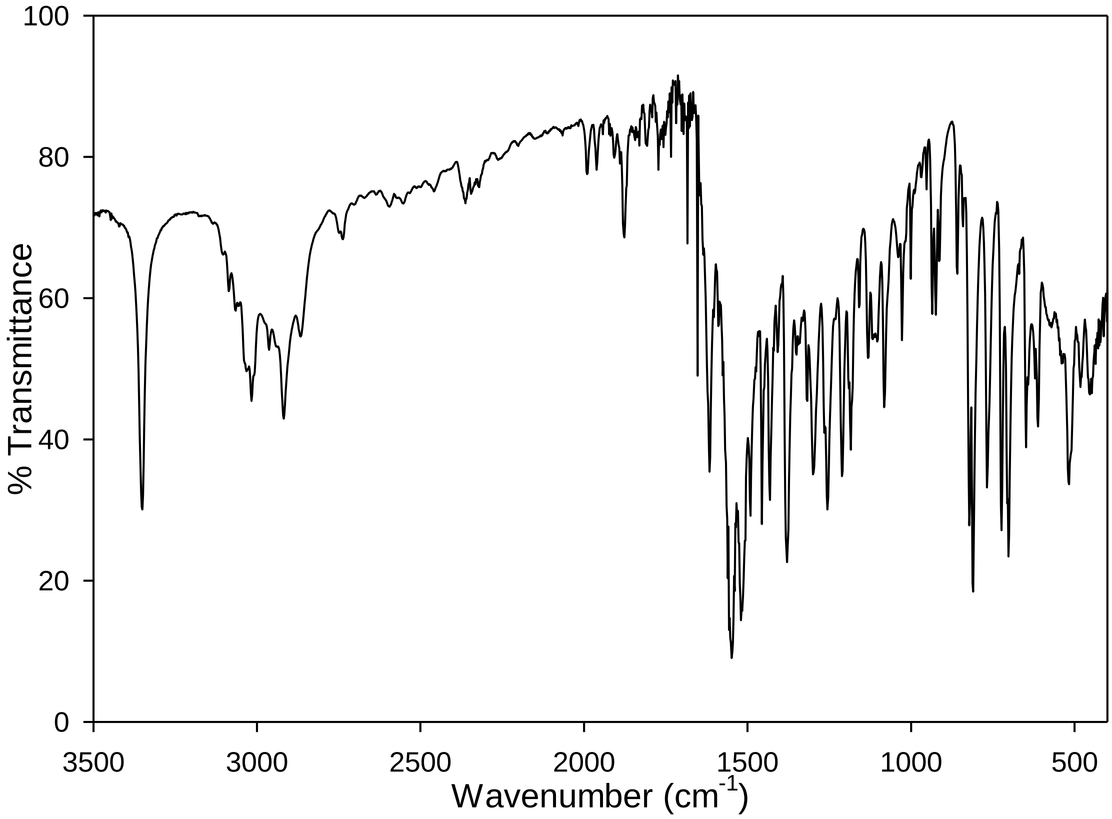

Figure 1.97: IR spectrum of **8b** 4-methyl-*N*-(2-nitro-1-phenylethyl)aniline.

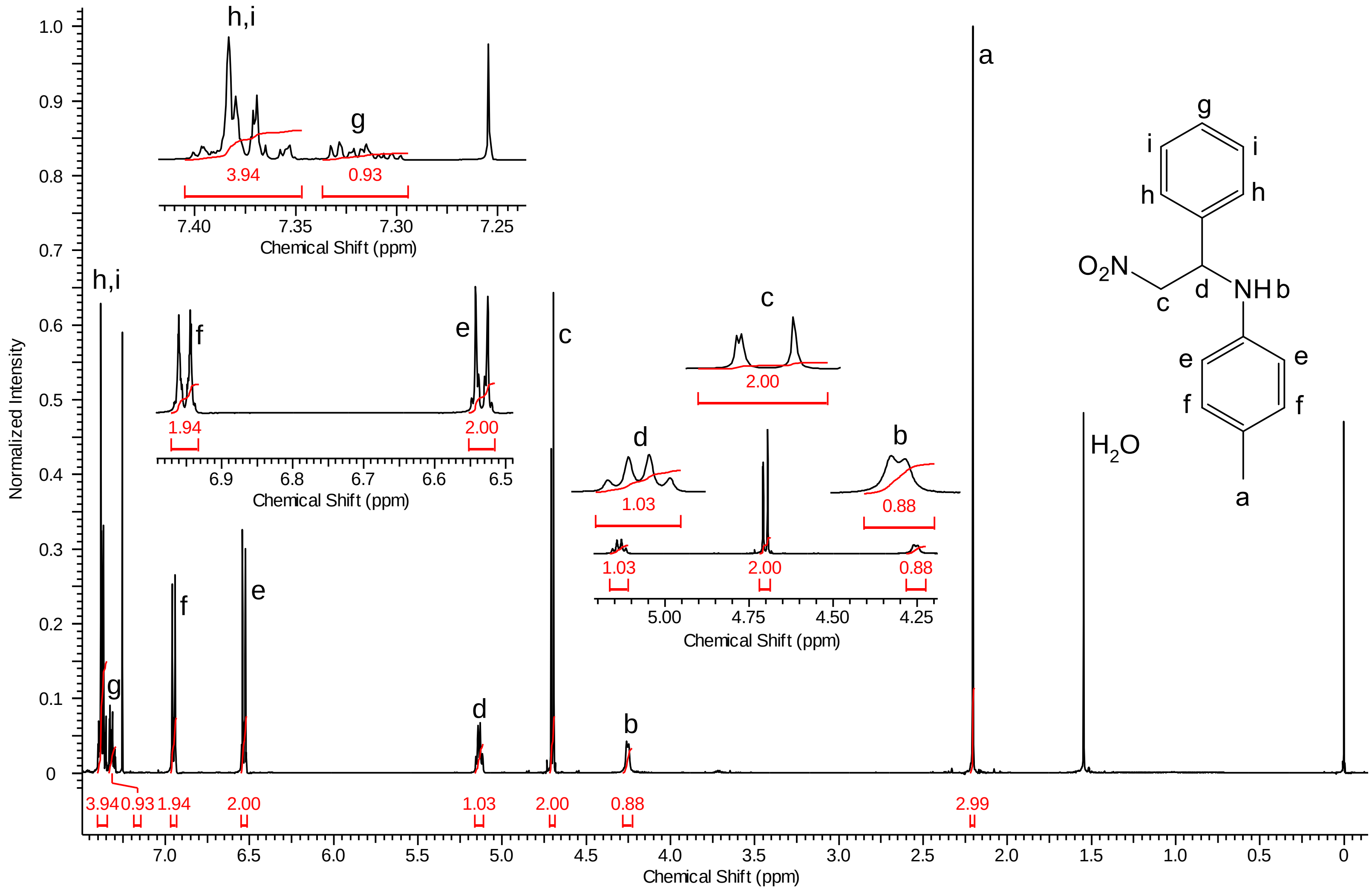

Figure 1.98: ^{1}H NMR spectrum of **8b** 4-methyl-*N*-(2-nitro-1-phenylethyl)aniline.

8c 3-methyl-*N*-(2-nitro-1-phenylethyl)aniline

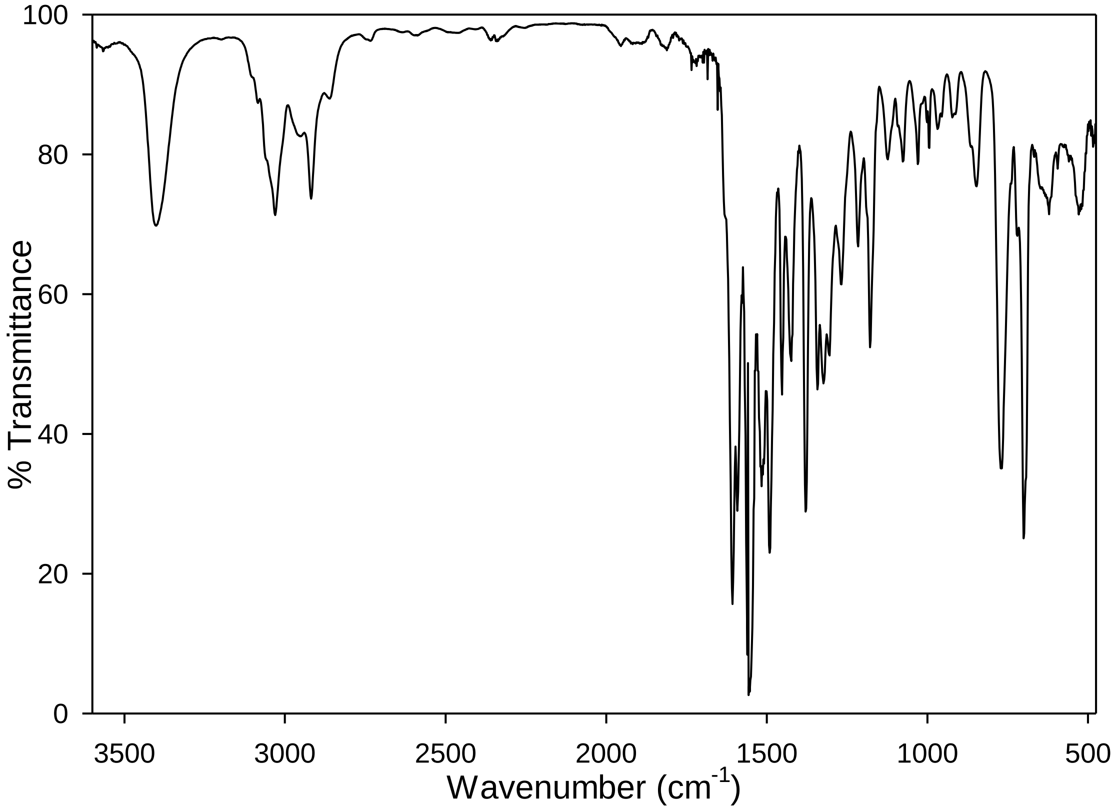

Figure 1.99: IR spectrum of **8c** 3-methyl-*N*-(2-nitro-1-phenylethyl)aniline.

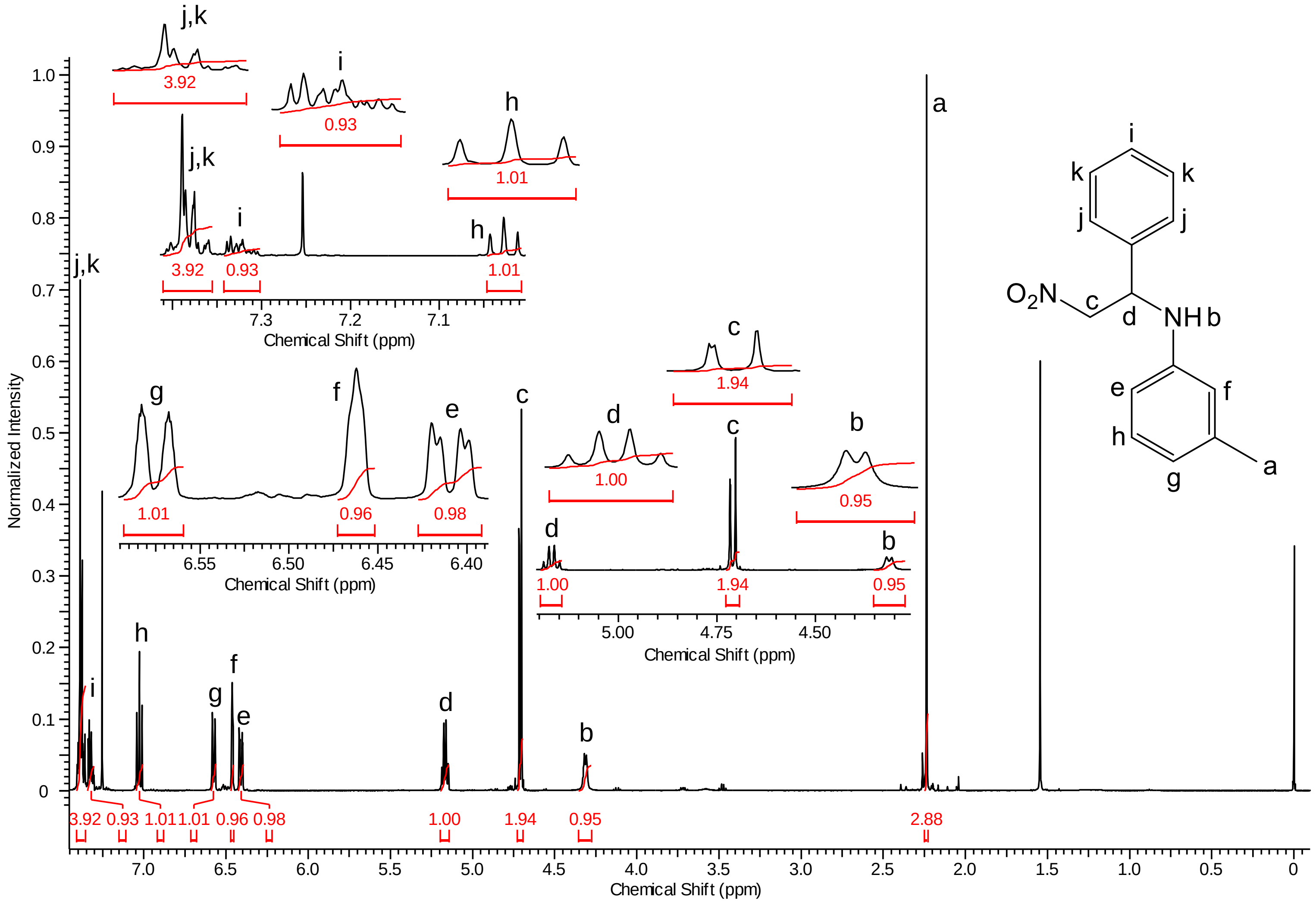

Figure 1.100: ^{1}H NMR spectrum of **8c** 3-methyl-*N*-(2-nitro-1-phenylethyl)aniline.

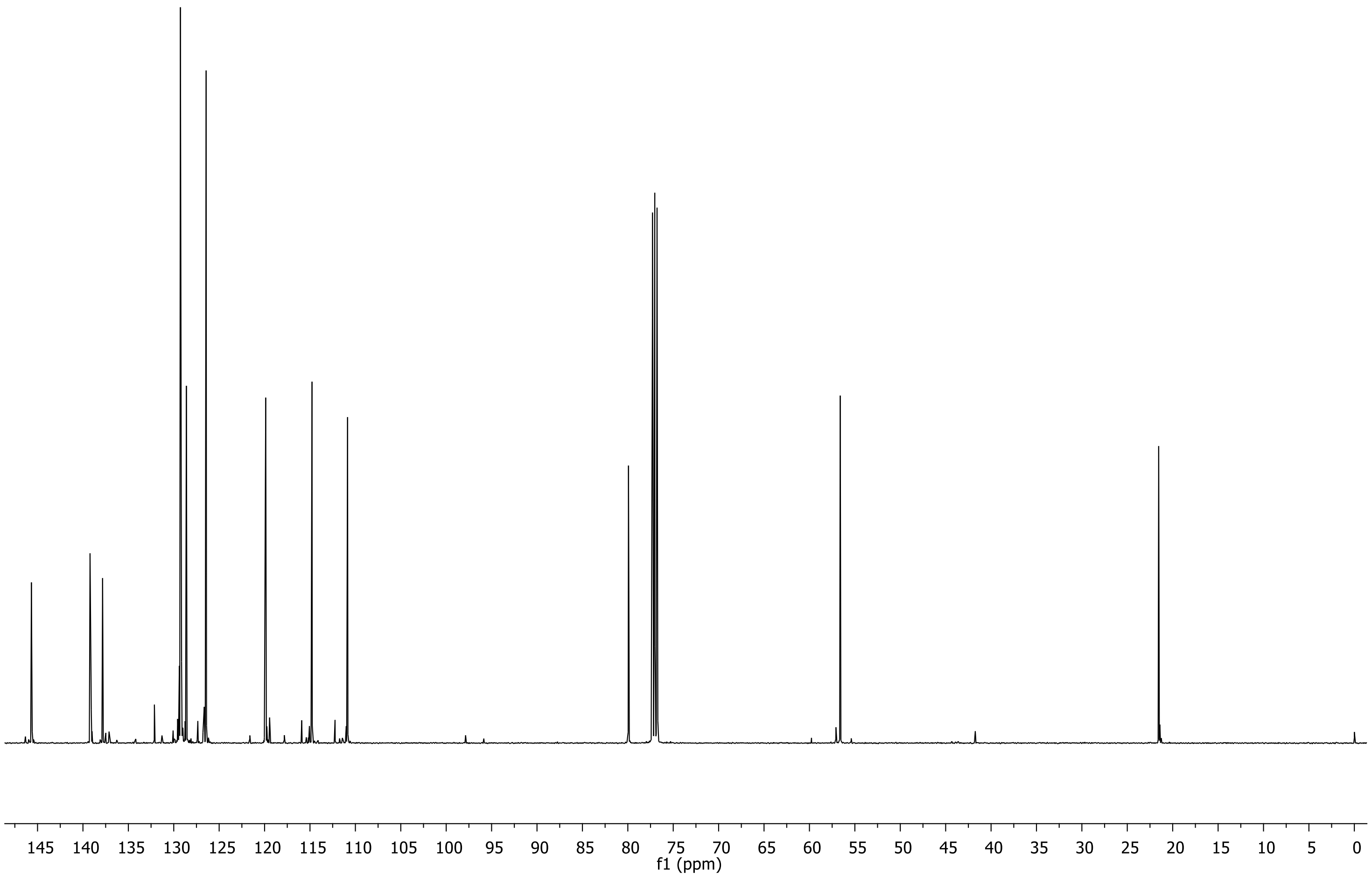

Figure 1.101: ^{13}C NMR spectrum of **8c** 3-methyl-*N*-(2-nitro-1-phenylethyl)aniline.

Table 1.14: MS data.

Compound	Formula/Mass		Parent m/z	Cone Voltage	Daughters	Collision Energy	Ion Mode
**8c**	256.3	1	257.08	20	107.98	14	ES+
		2	257.08	20	104.07	18	ES+
		3	257.08	20	196.11	12	ES+
		4	257.08	20	149.99	12	ES+
		5	257.08	20	78.05	50	ES+

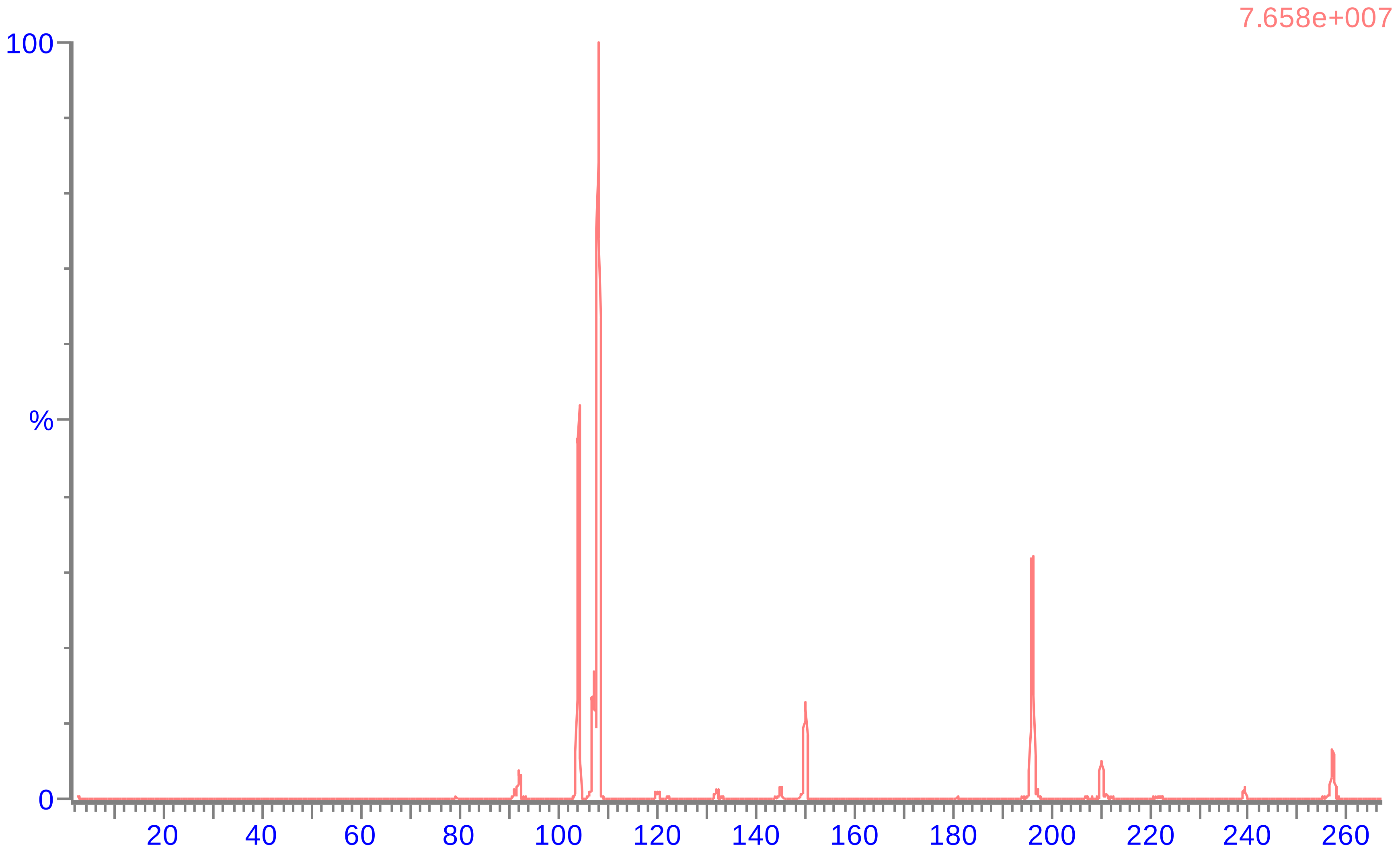

Figure 1.102: Mass spectrum for daughter fragment peak ES+, m/z 257.08 -> 107.98.

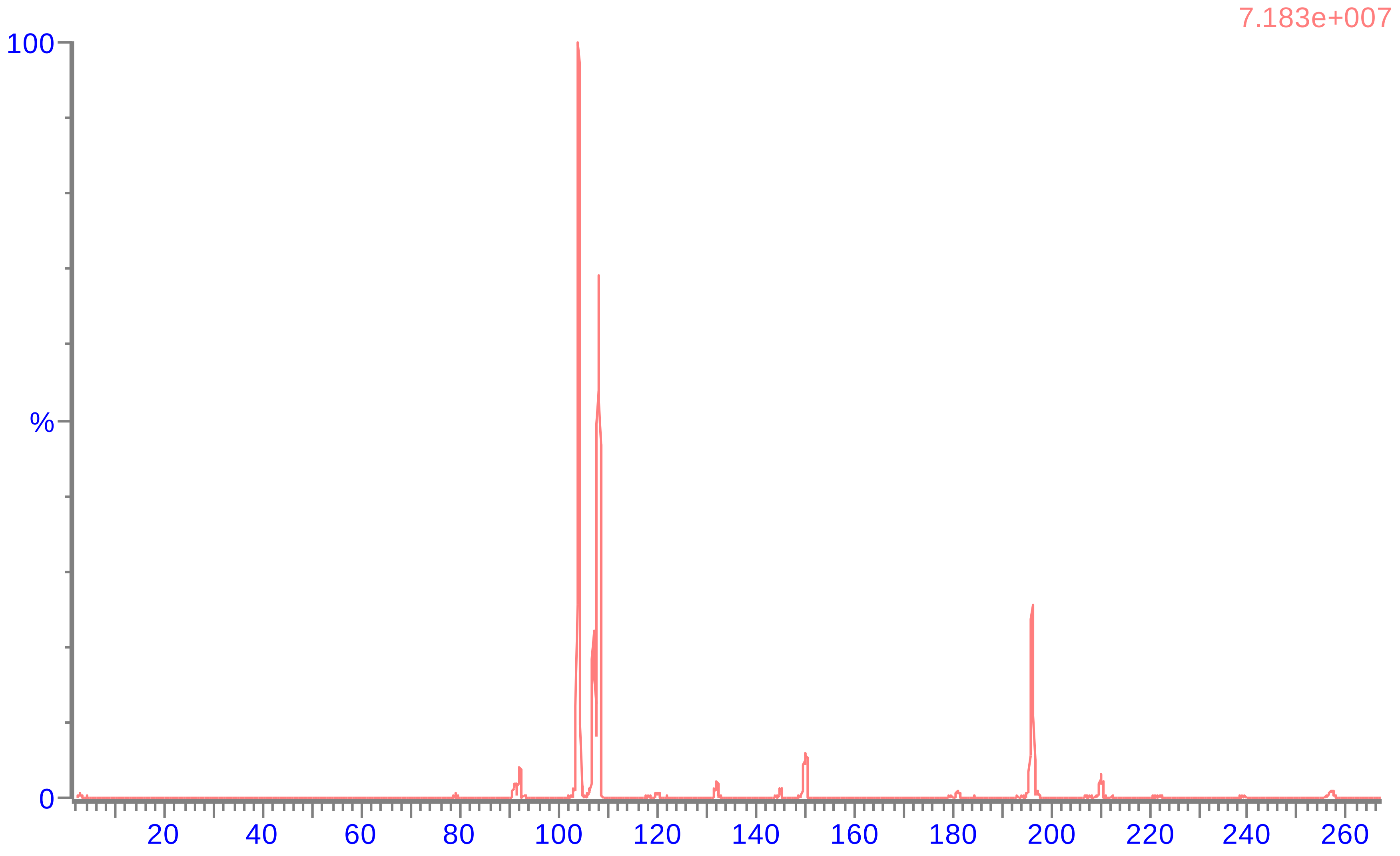

Figure 1.103: Mass spectrum for daughter fragment peak ES+, m/z 257.08 -> 104.07.

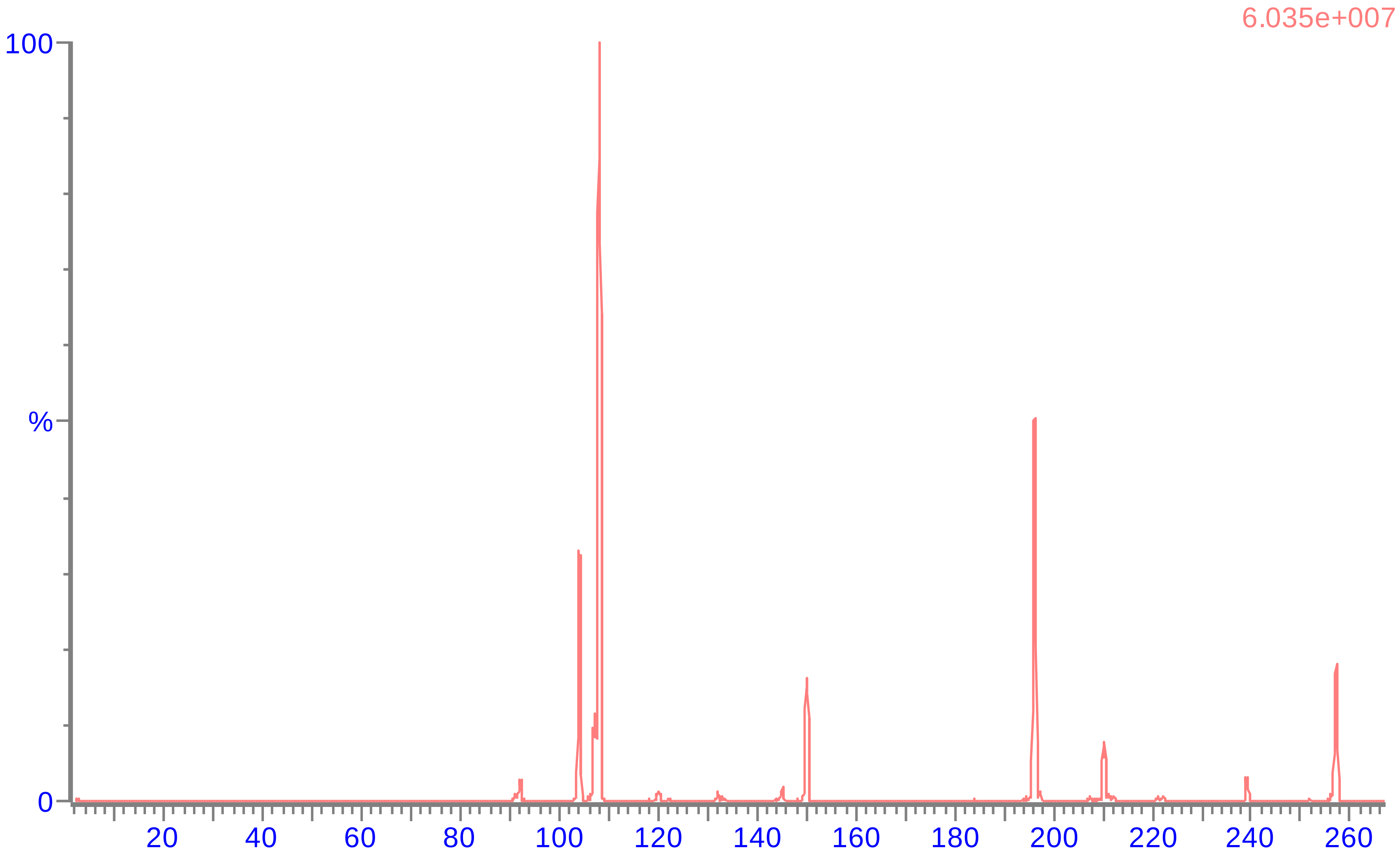

Figure 1.104: Mass spectrum for daughter fragment peak ES+, m/z 257.08 -> 196.11.

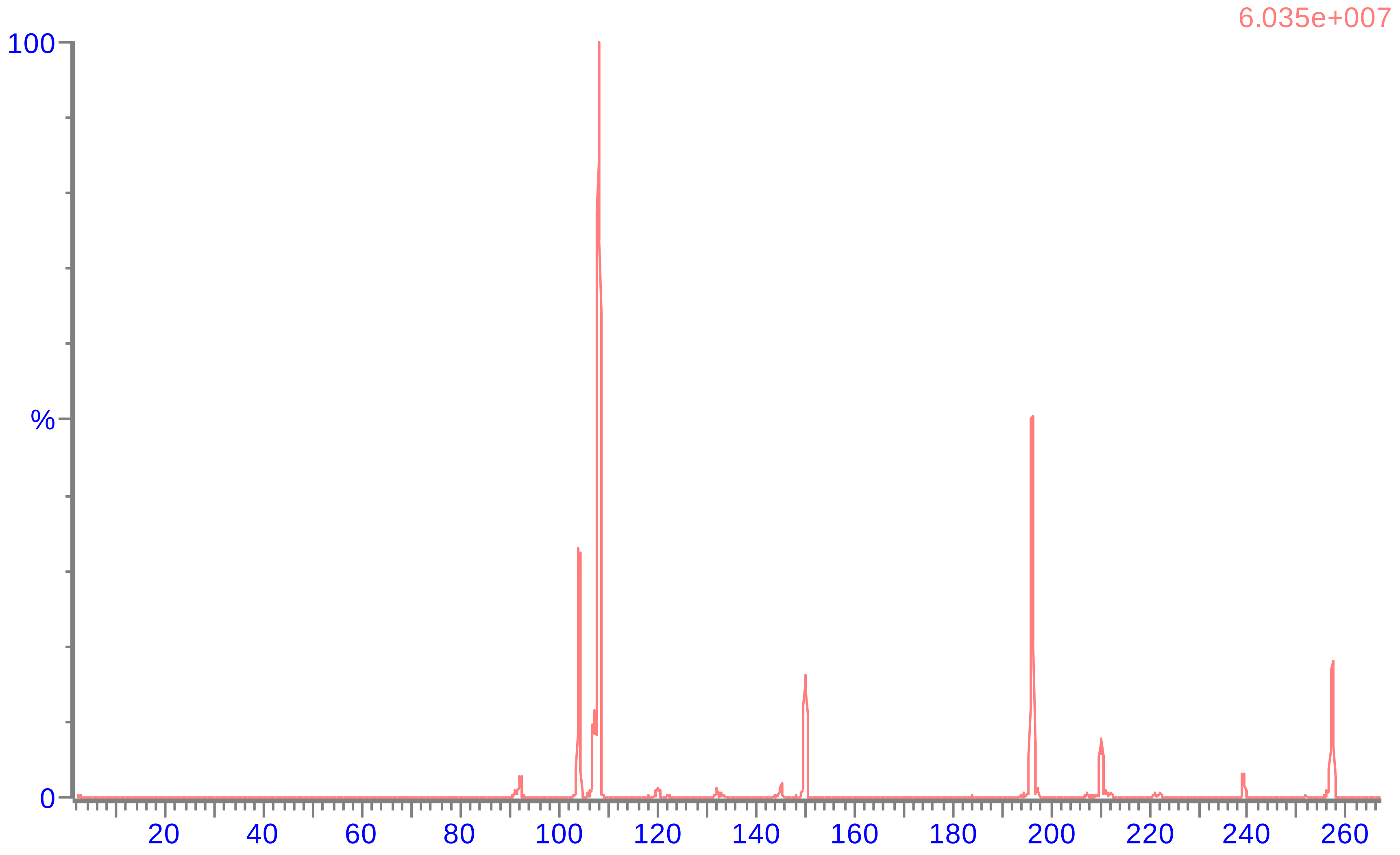

Figure 1.105: Mass spectrum for daughter fragment peak ES+, m/z 257.08 -> 149.99.

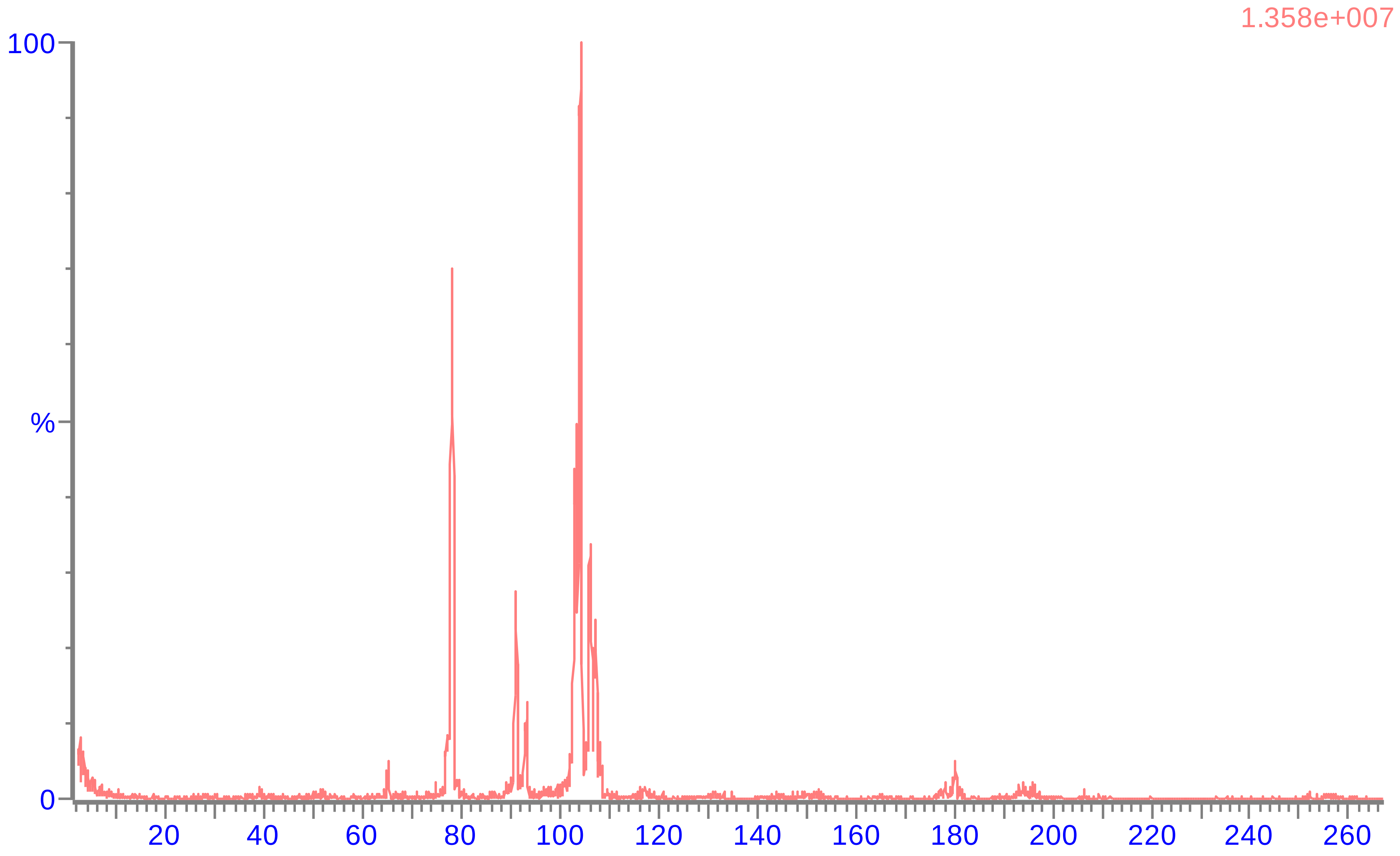

Figure 1.106: Mass spectrum for daughter fragment peak ES+, m/z 257.08 -> 78.05.

8d 2-methyl-*N*-(2-nitro-1-phenylethyl)aniline

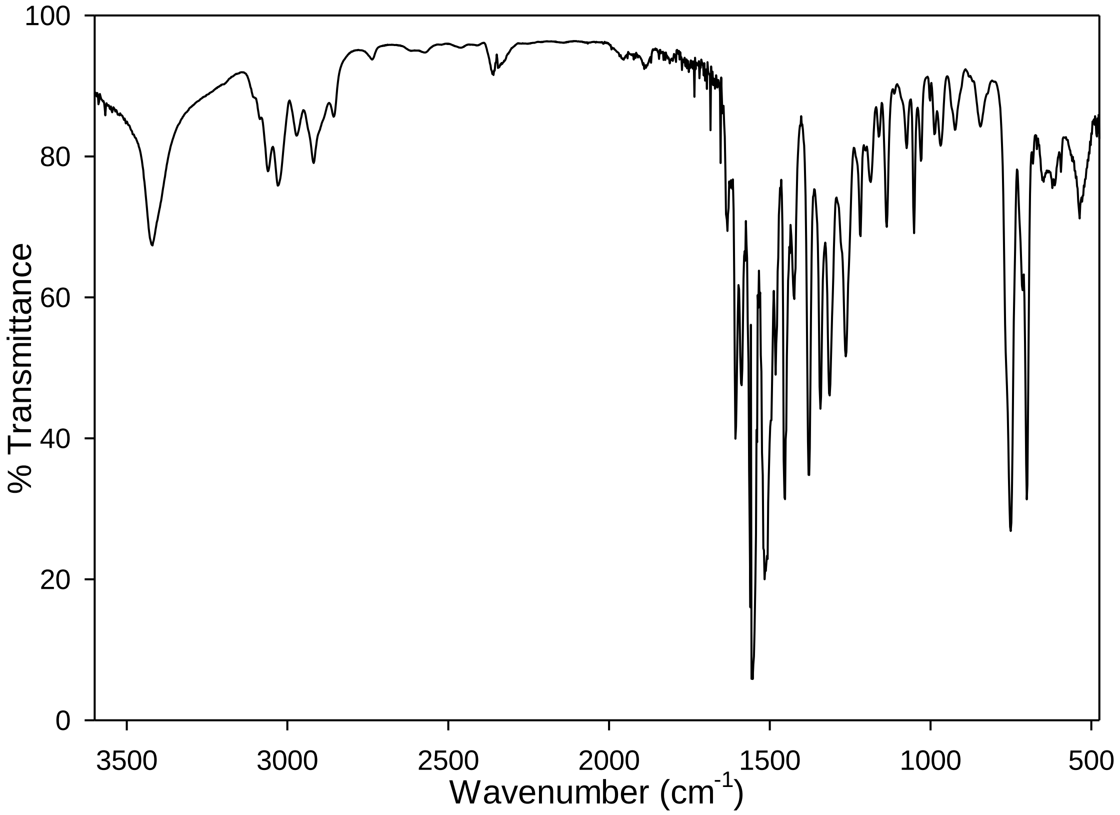

Figure 1.107: IR spectrum of **8d** 2-methyl-*N*-(2-nitro-1-phenylethyl)aniline

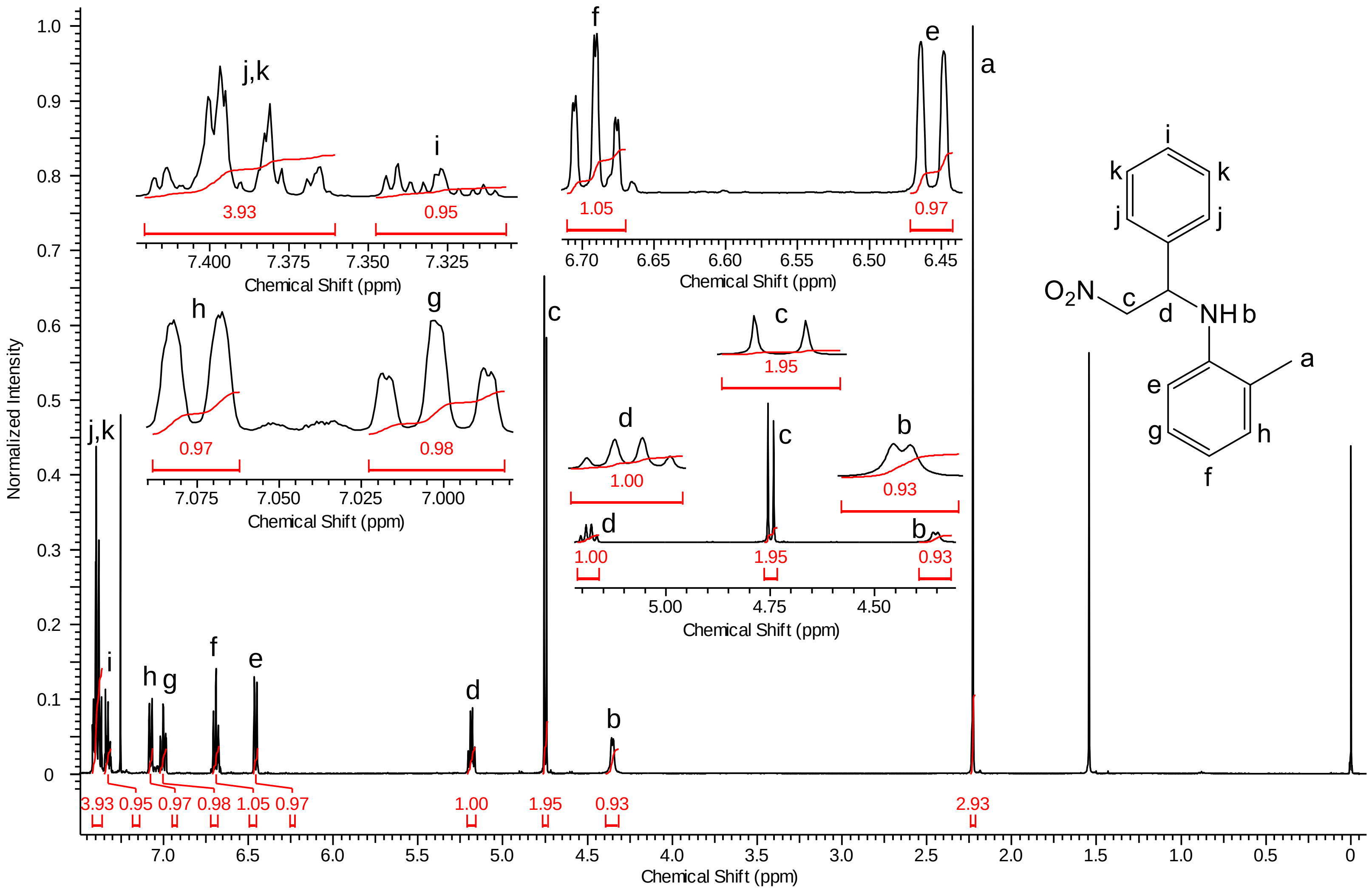

Figure 1.108: ^{1}H NMR spectrum of **8d** 2-methyl-*N*-(2-nitro-1-phenylethyl)aniline.

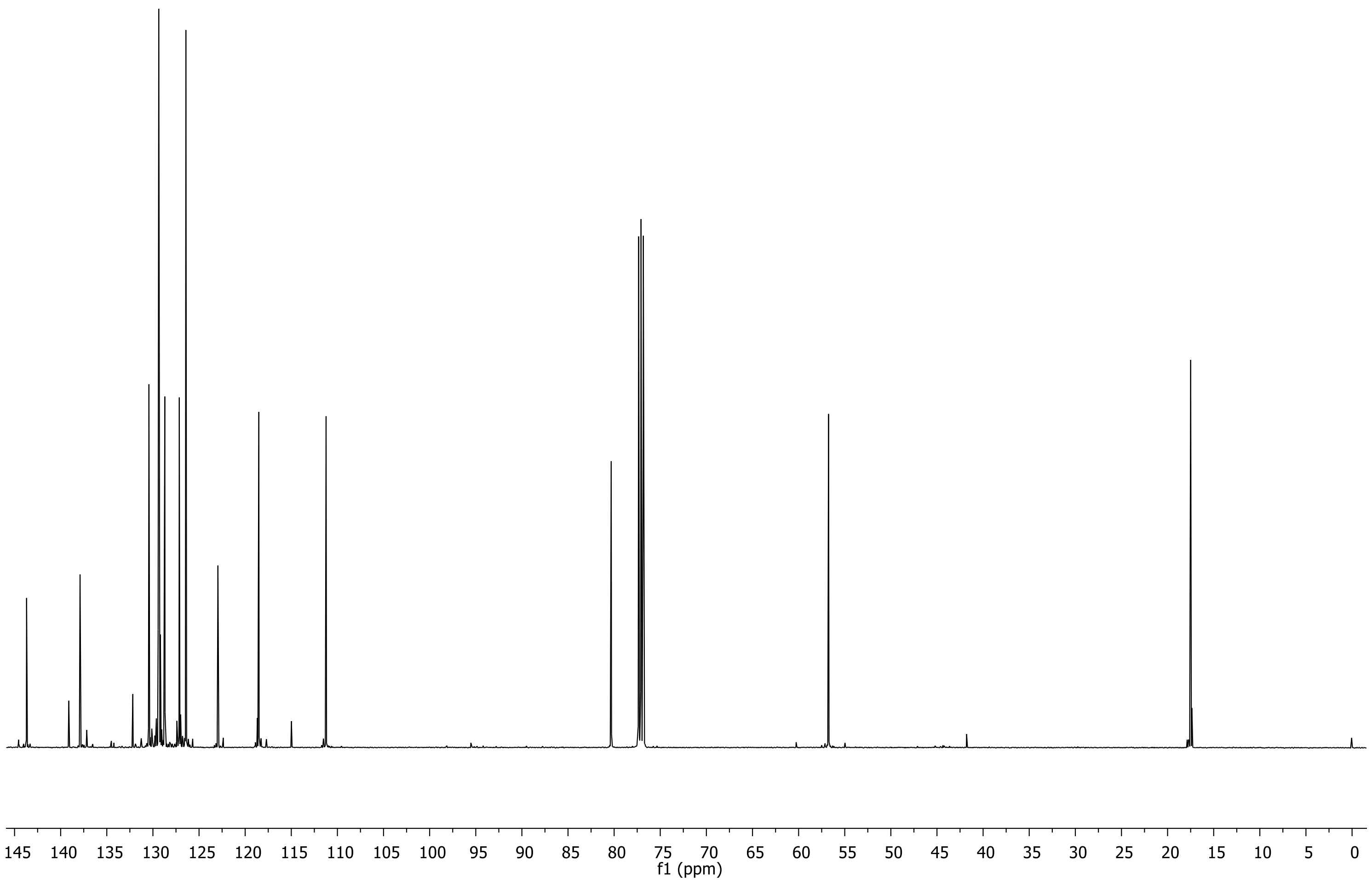

Figure 1.109: ^{13}C NMR spectrum of **8d** 2-methyl-*N*-(2-nitro-1-phenylethyl)aniline.

Table 1.15: MS data.

Compound	Formula/Mass		Parent m/z	Cone Voltage	Daughters	Collision Energy	Ion Mode
		1	257.08	20	104.07	22	ES+
		2	257.08	20	196.11	10	ES+
**8d**	256.3	3	257.08	20	107.98	14	ES+
		4	257.08	20	149.99	12	ES+
		5	257.08	20	77.99	50	ES+

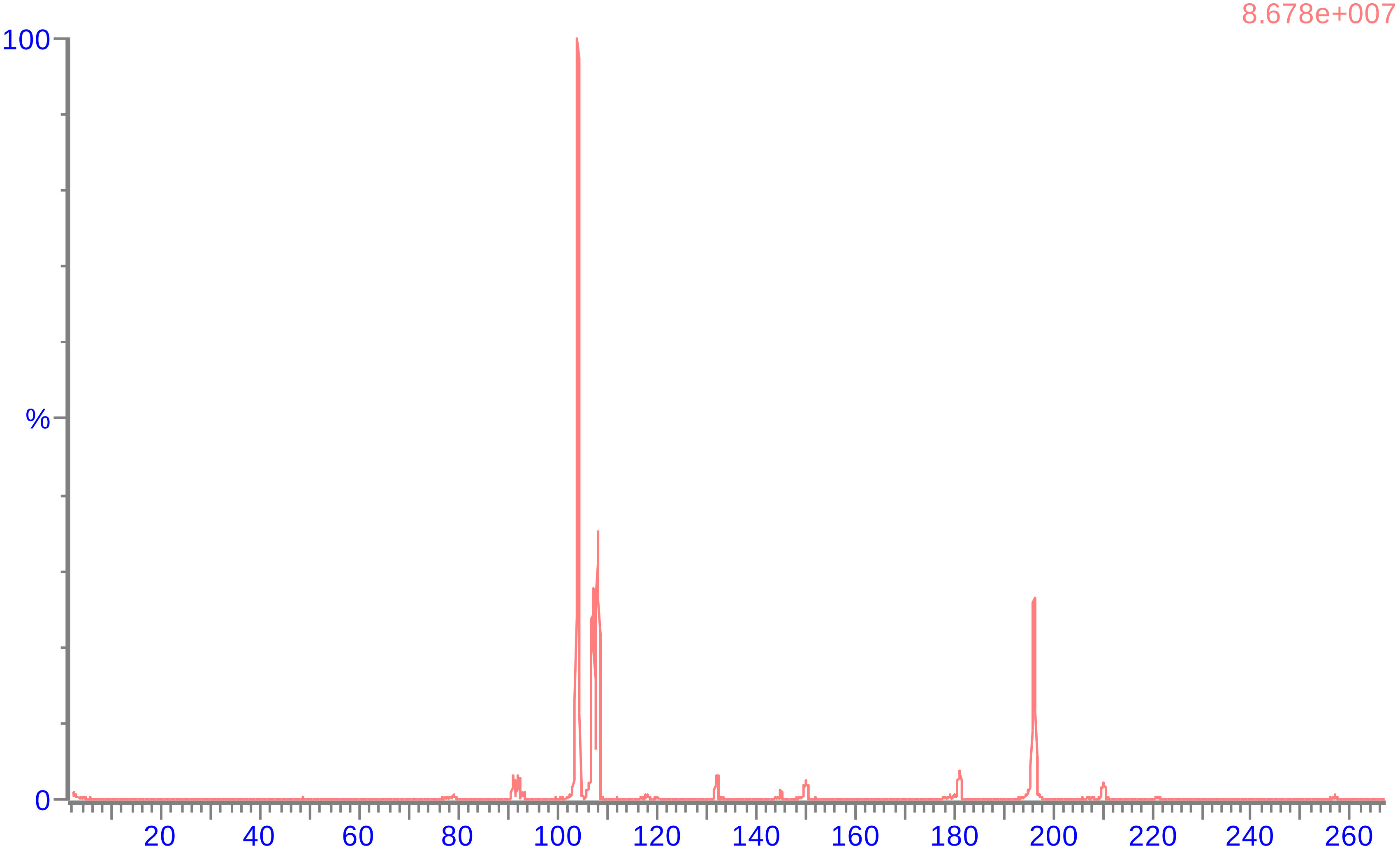

Figure 1.110: Mass spectrum for daughter fragment peak ES+, m/z 257.08 -> 104.07.

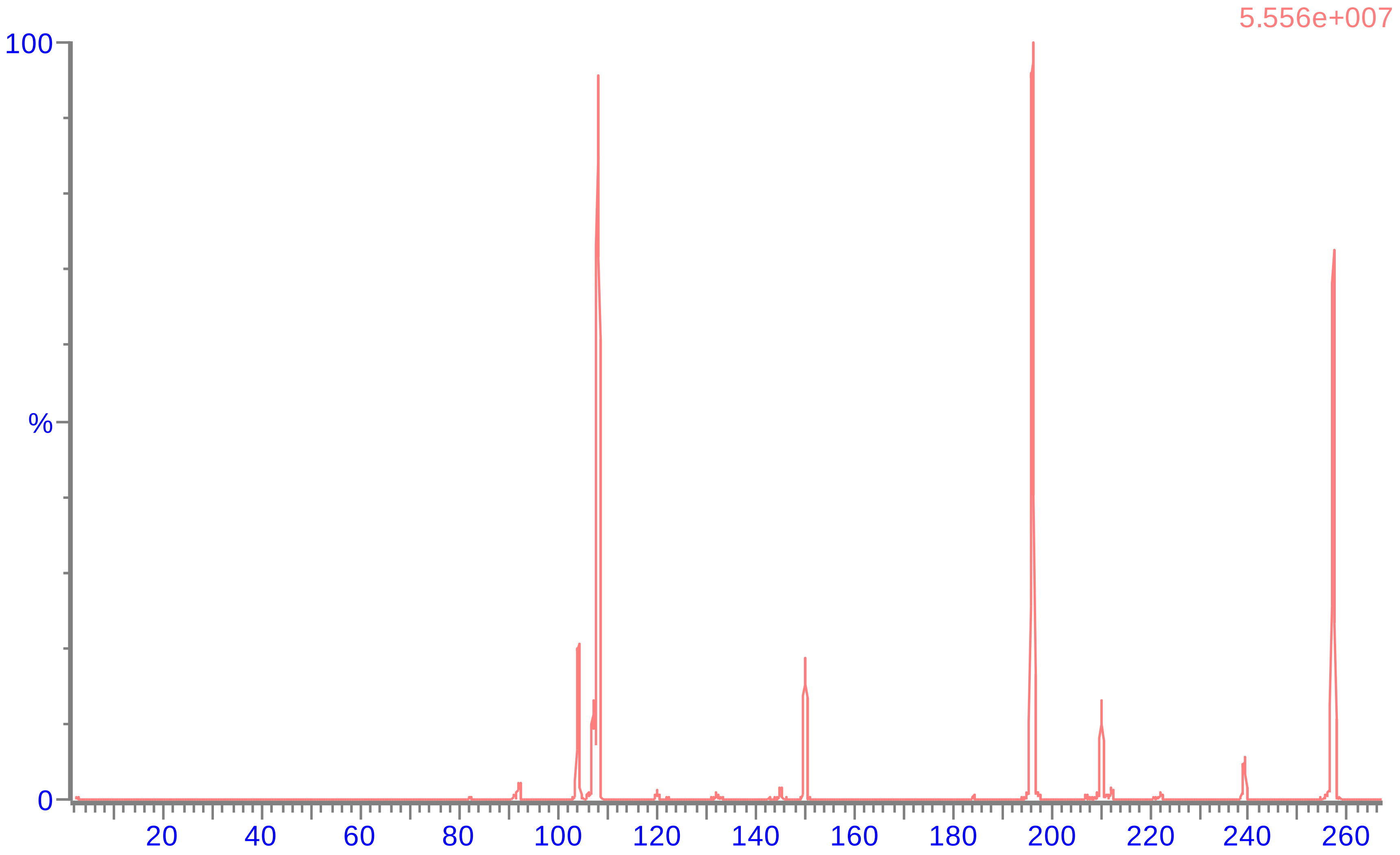

Figure 1.111: Mass spectrum for daughter fragment peak ES+, m/z 257.08 -> 196.11.

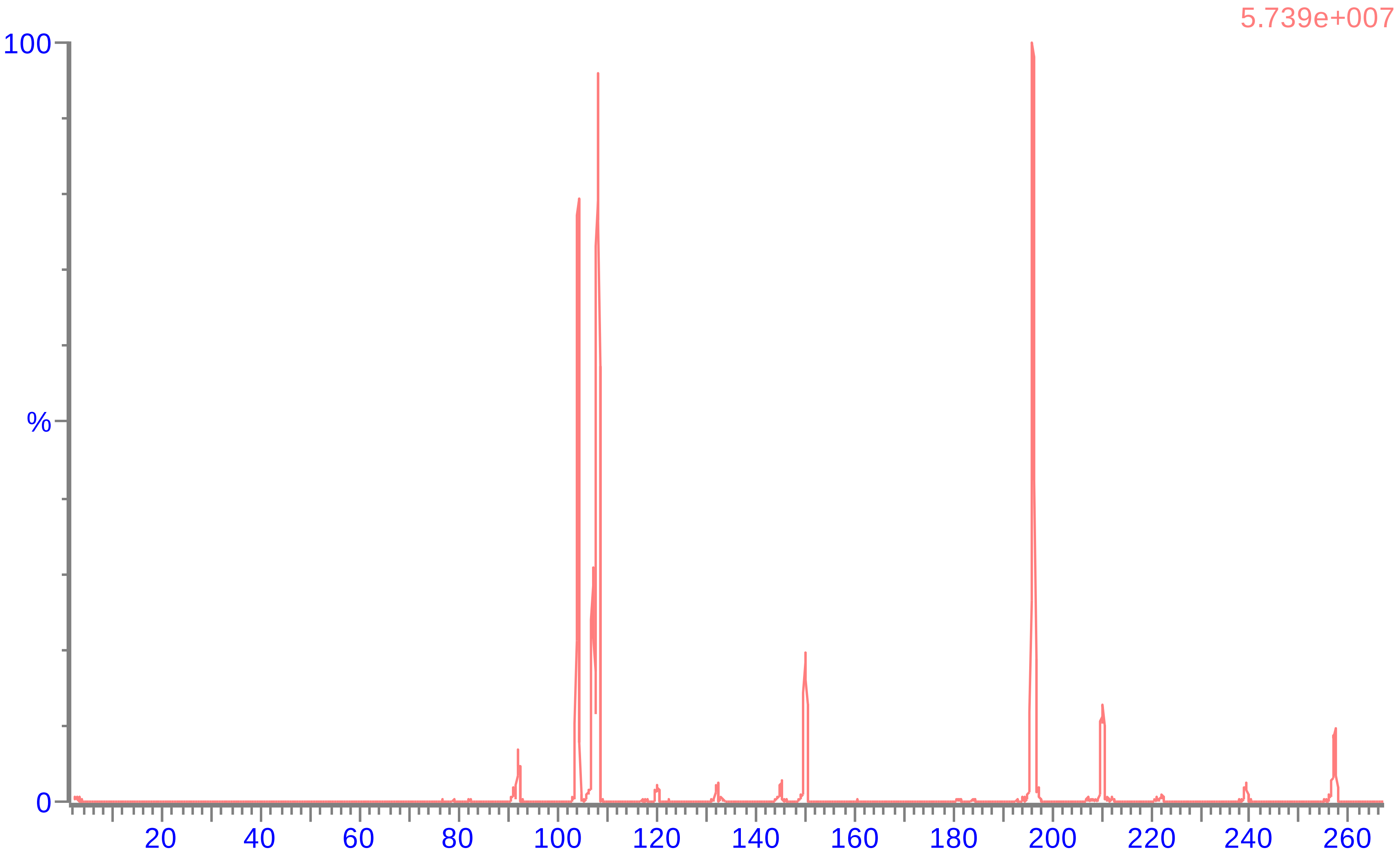

Figure 1.112: Mass spectrum for daughter fragment peak ES+, m/z 257.08 -> 107.98.

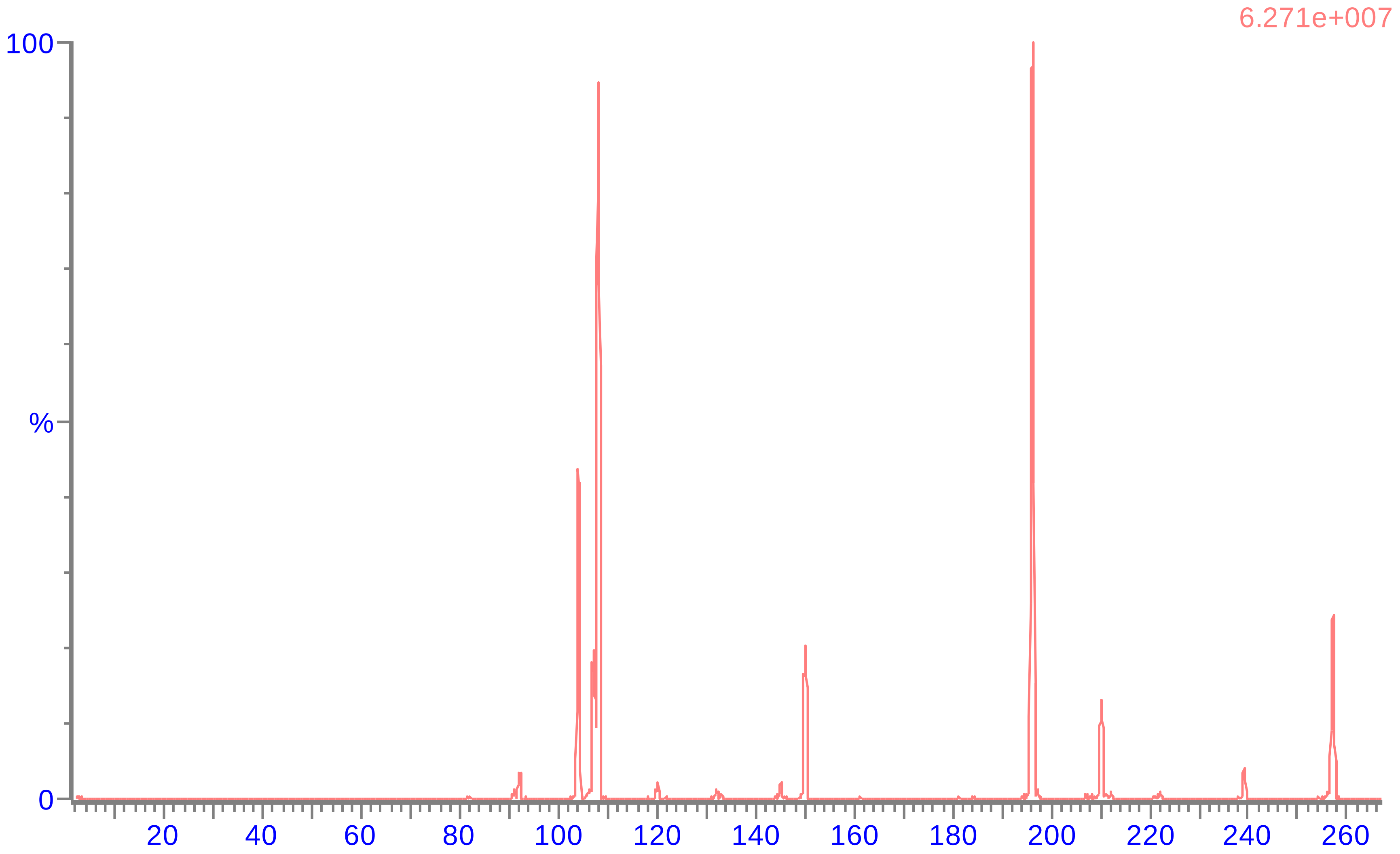

Figure 1.113: Mass spectrum for daughter fragment peak ES+, m/z 257.08 -> 149.99.

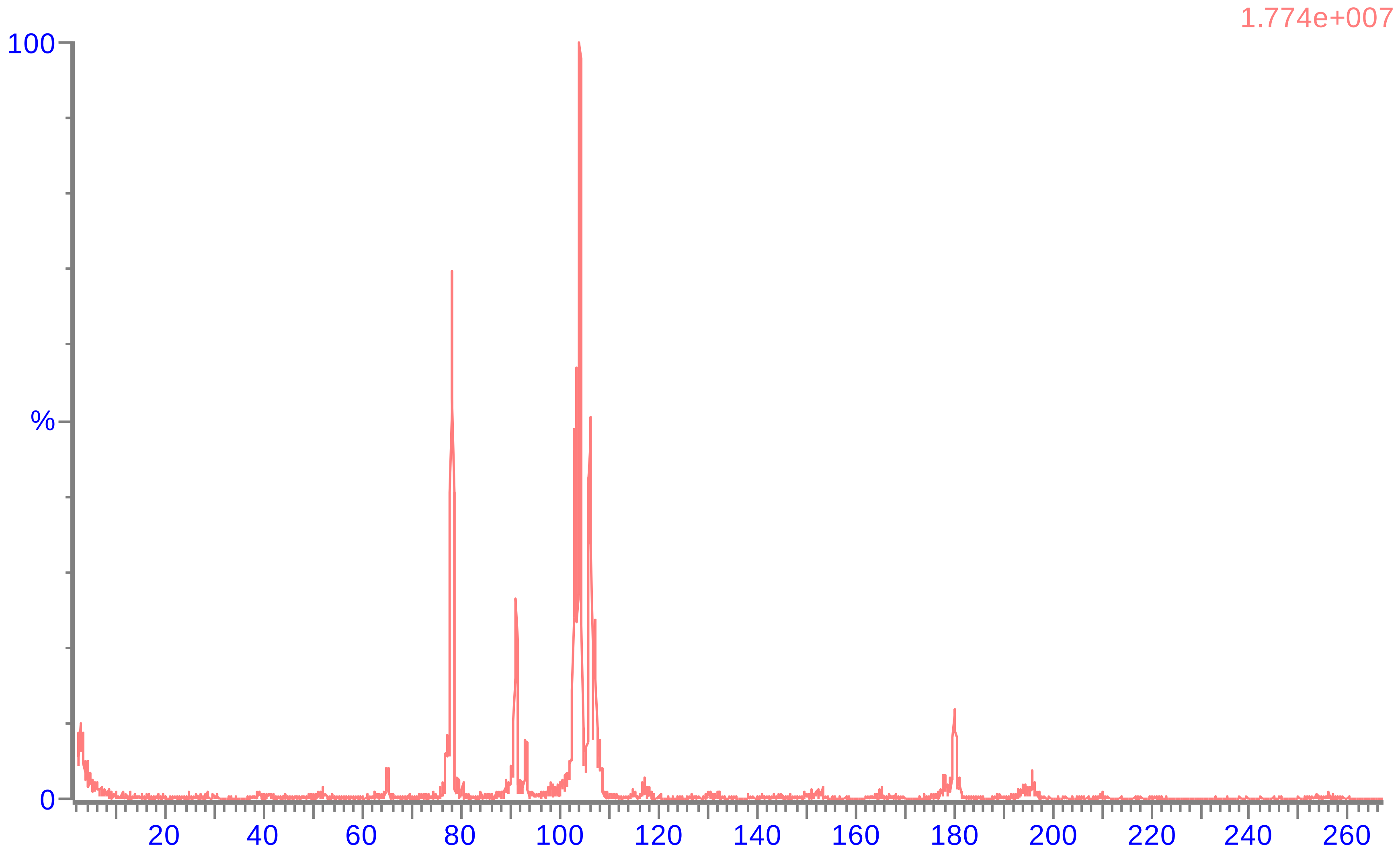

Figure 1.114: Mass spectrum for daughter fragment peak ES+, m/z 257.08 -> 77.99.

8e 4-ethyl-*N*-(2-nitro-1-phenylethyl)aniline

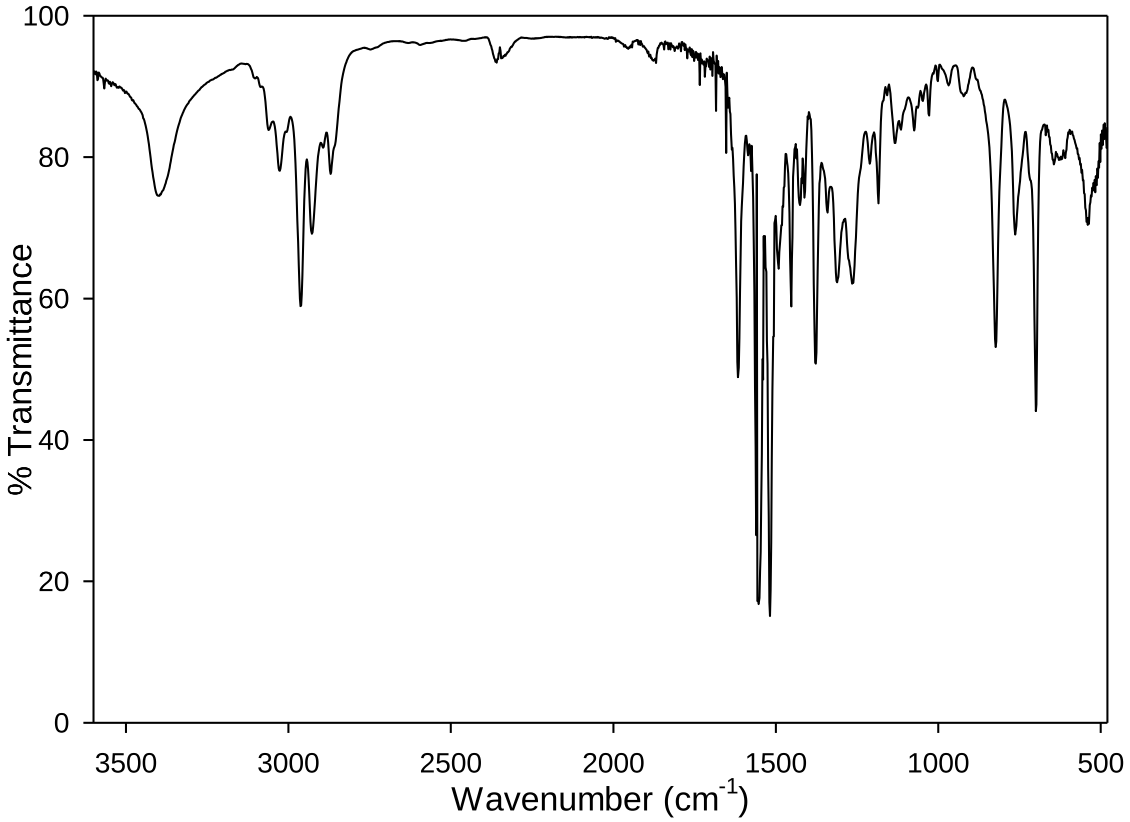

Figure 1.115: IR spectrum of **8e** 4-ethyl-*N*-(2-nitro-1-phenylethyl)aniline.

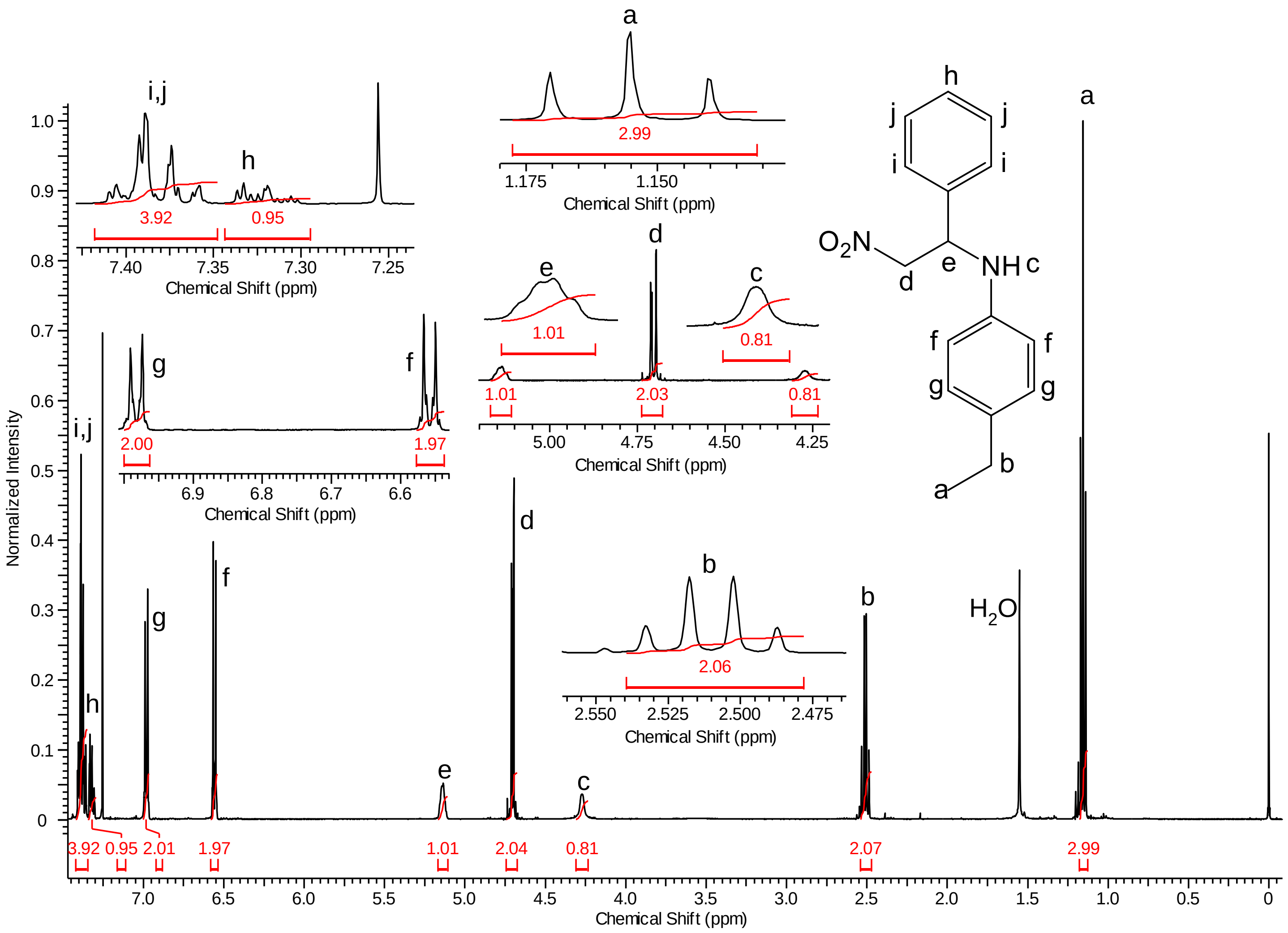

Figure 1.116: ^{1}H NMR spectrum of **8e** 4-ethyl-*N*-(2-nitro-1-phenylethyl)aniline.

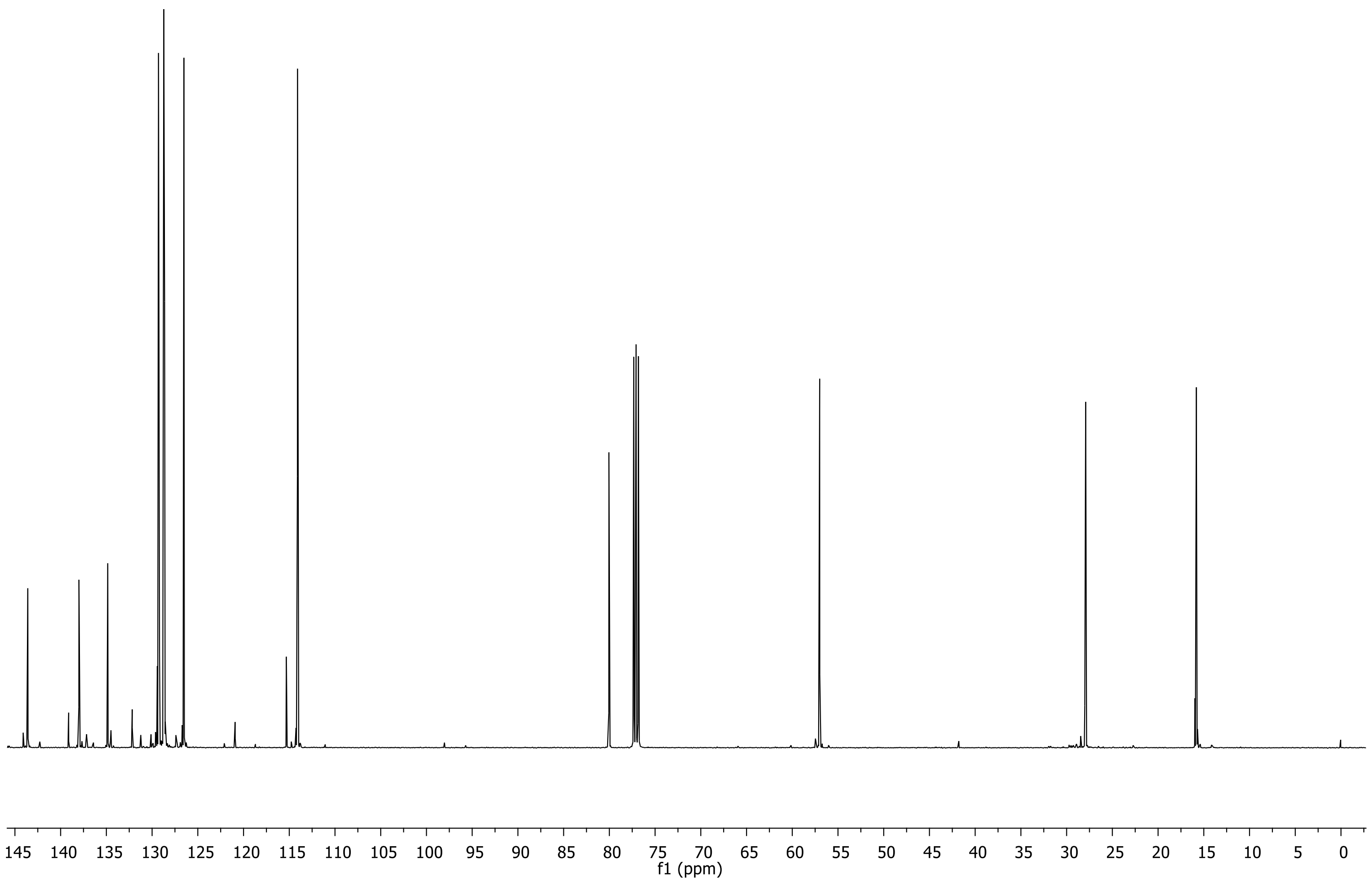

Figure 1.117: ^{13}C NMR spectrum of **8e** 4-ethyl-*N*-(2-nitro-1-phenylethyl)aniline.

Table 1.16: MS data.

Compound	Formula/Mass		Parent m/z	Cone Voltage	Daughters	Collision Energy	Ion Mode
**8e**	270.3	1	271.08	20	121.37	16	ES+
		2	271.08	20	103.69	32	ES+
		3	271.08	20	104.01	26	ES+
		4	271.08	20	210.11	10	ES+
		5	271.08	20	78.06	50	ES+

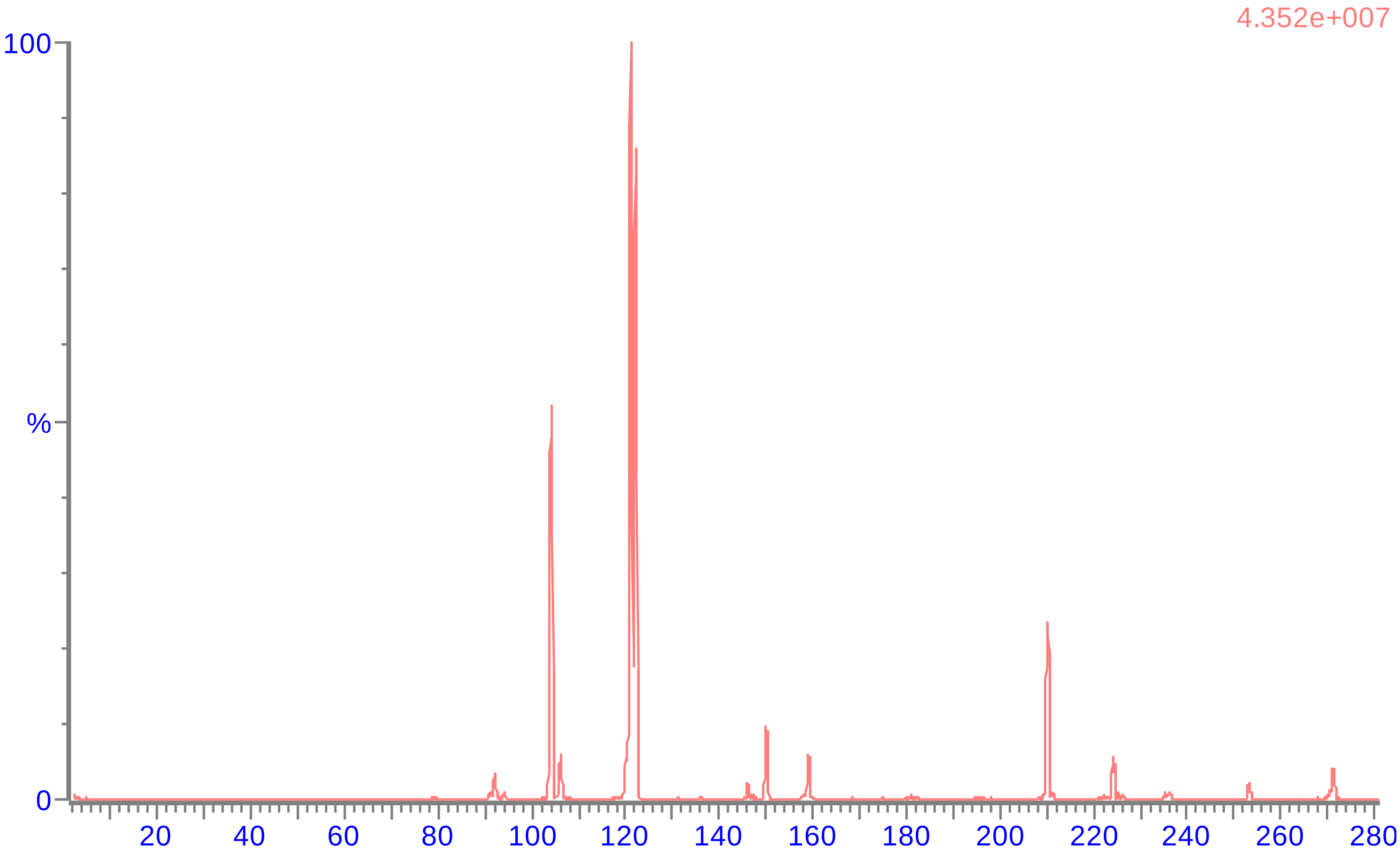

Figure 1.118: Mass spectrum for daughter fragment peak ES+, m/z 271.08 -> 121.37.

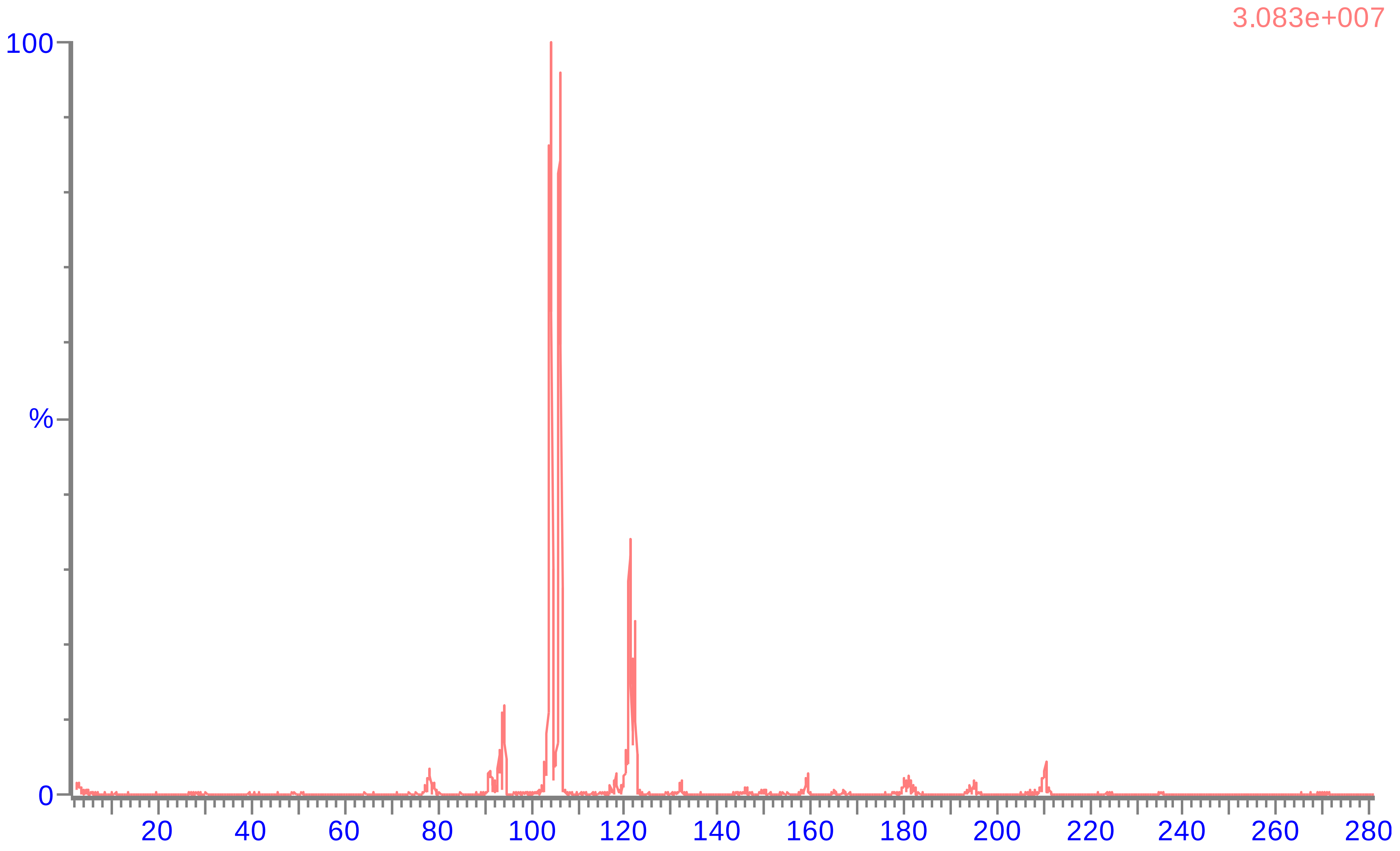

Figure 1.119: Mass spectrum for daughter fragment peak ES+, m/z 271.08 -> 103.69.

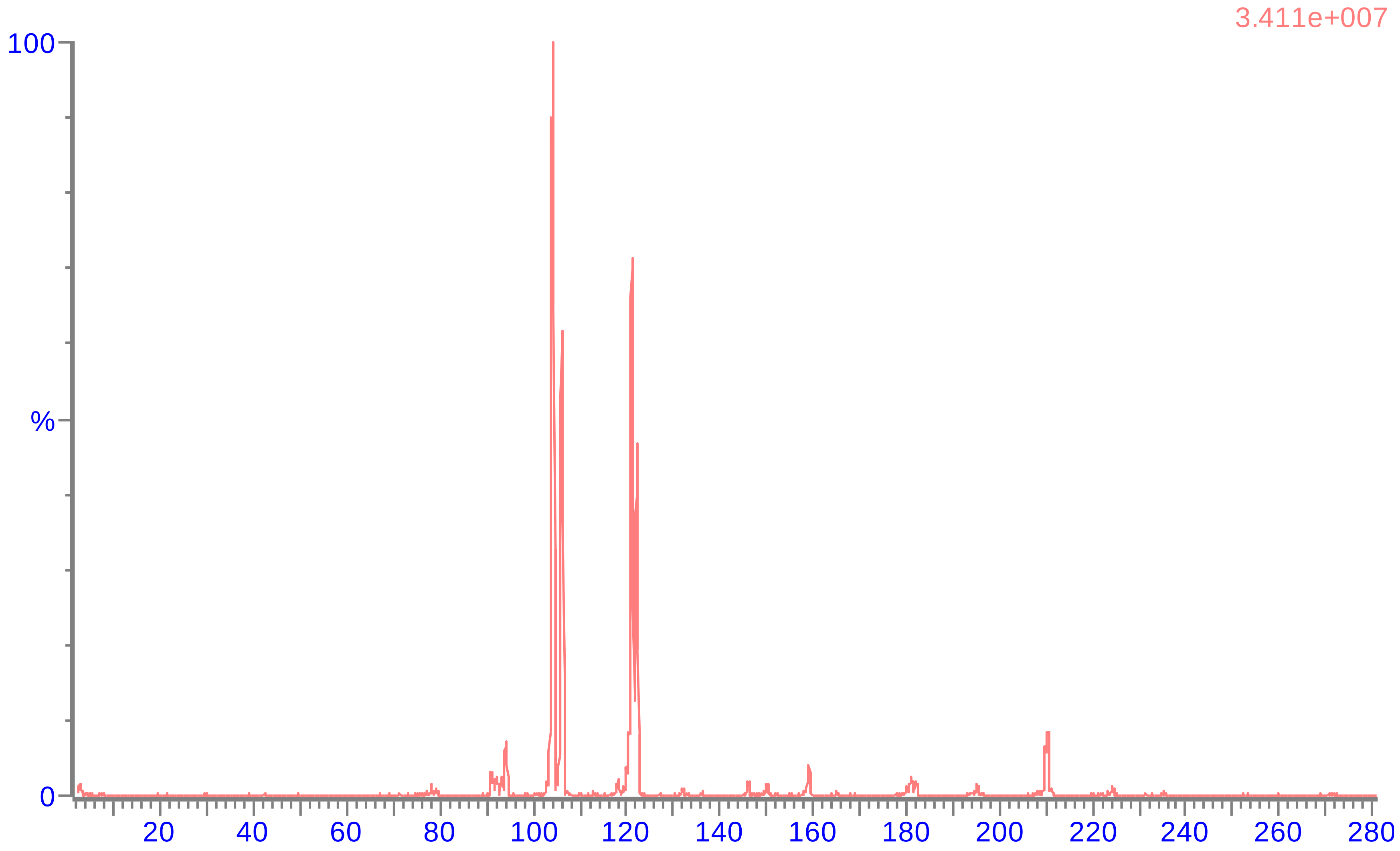

Figure 1.120: Mass spectrum for daughter fragment peak ES+, m/z 271.08 -> 104.01.

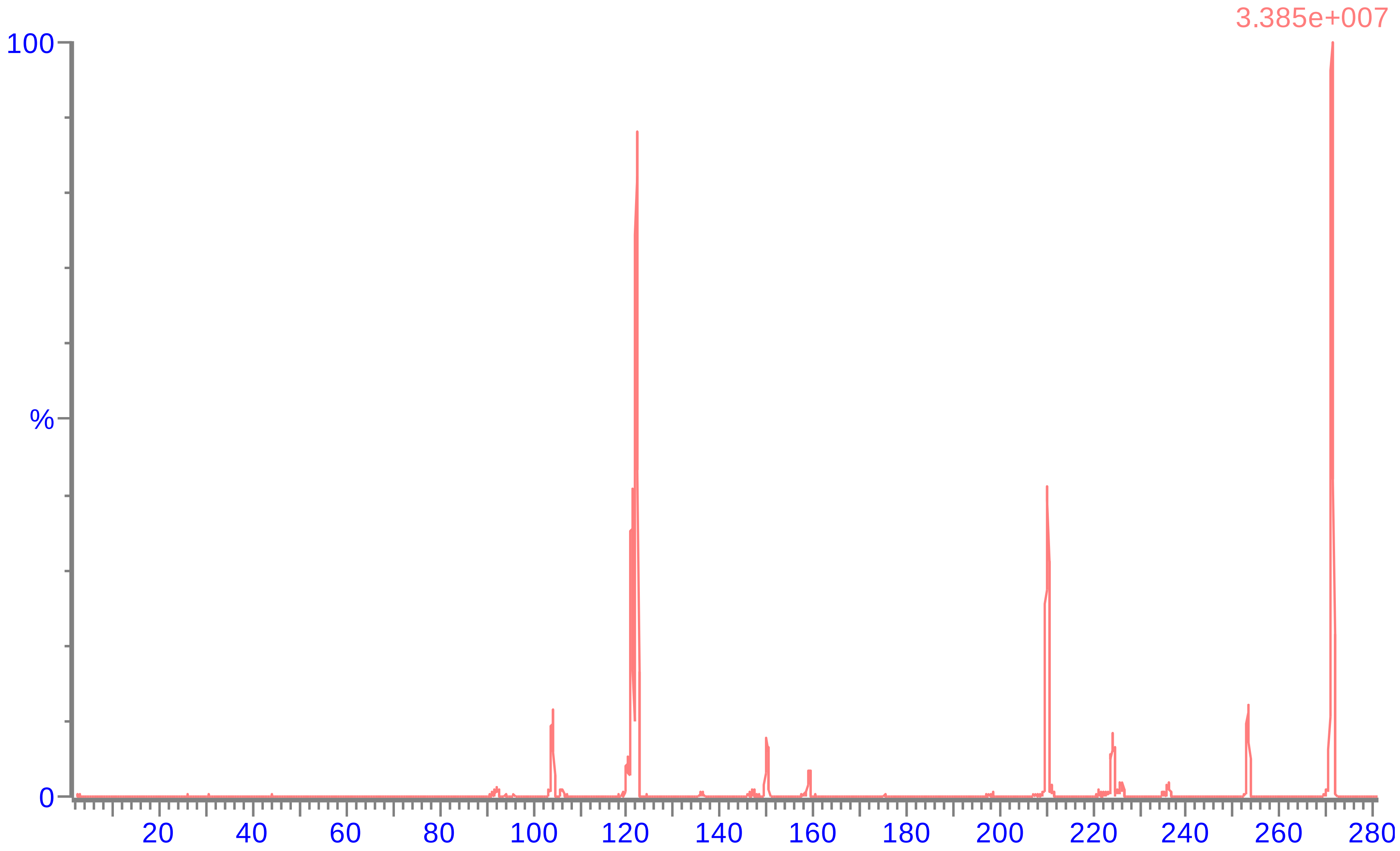

Figure 1.121: Mass spectrum for daughter fragment peak ES+, m/z 271.08 -> 210.11.

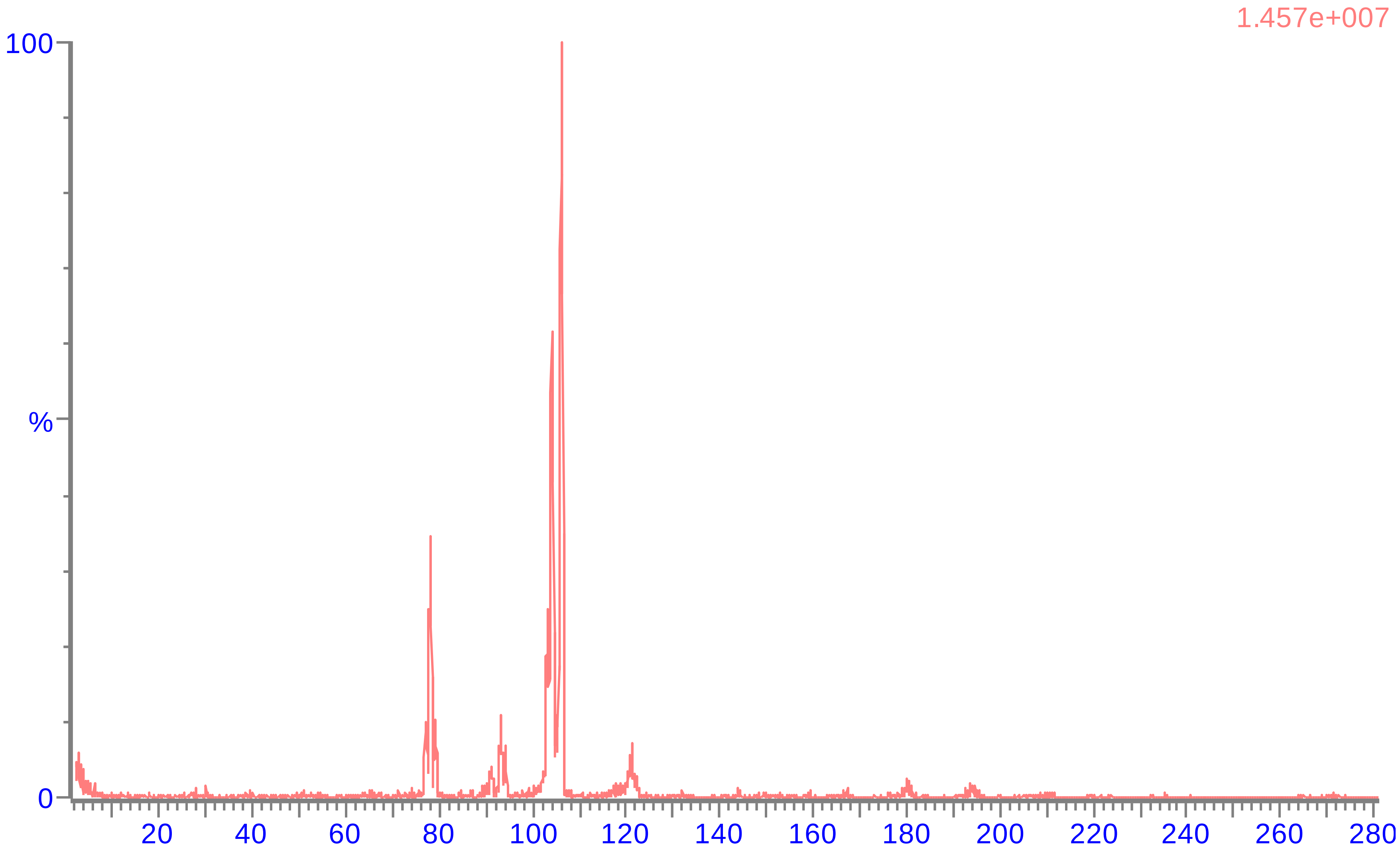

Figure 1.122: Mass spectrum for daughter fragment peak ES+, m/z 271.08 -> 78.06.

8f 4-bromo-*N*-(2-nitro-1-phenylethyl)aniline

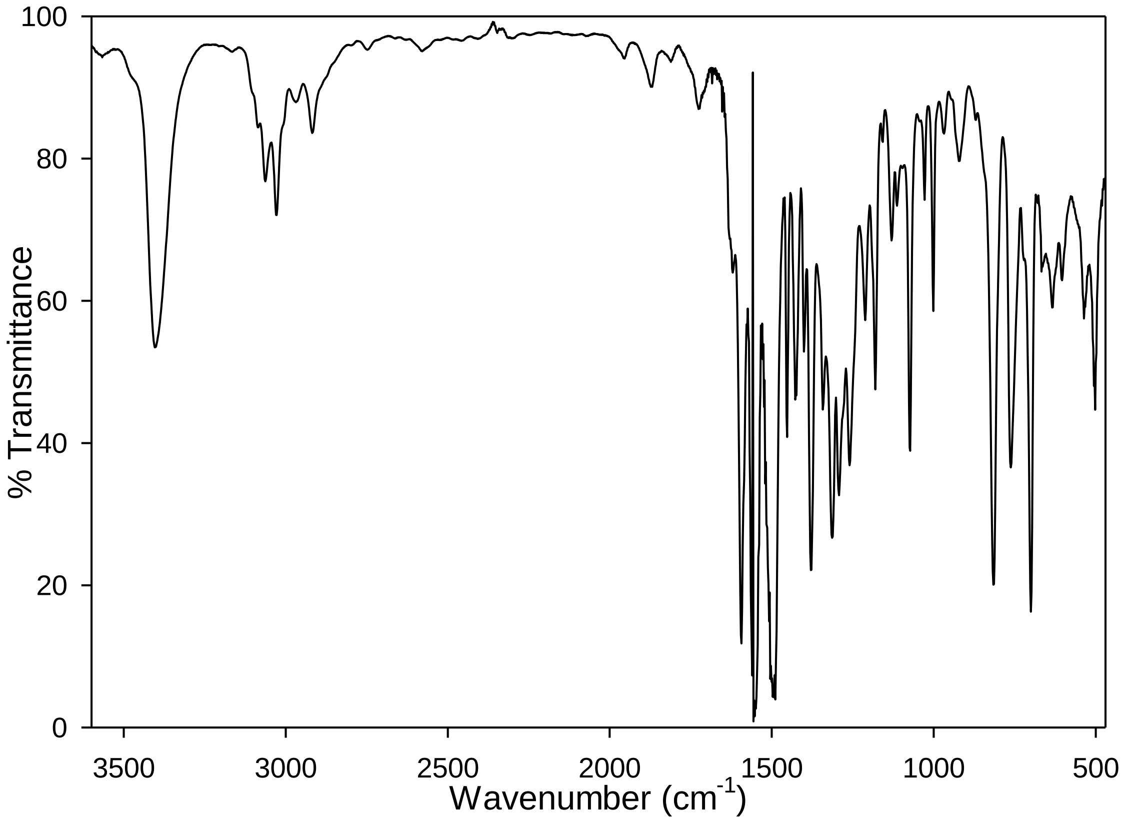

Figure 1.123: IR spectrum of **8f** 4-bromo-*N*-(2-nitro-1-phenylethyl)aniline.

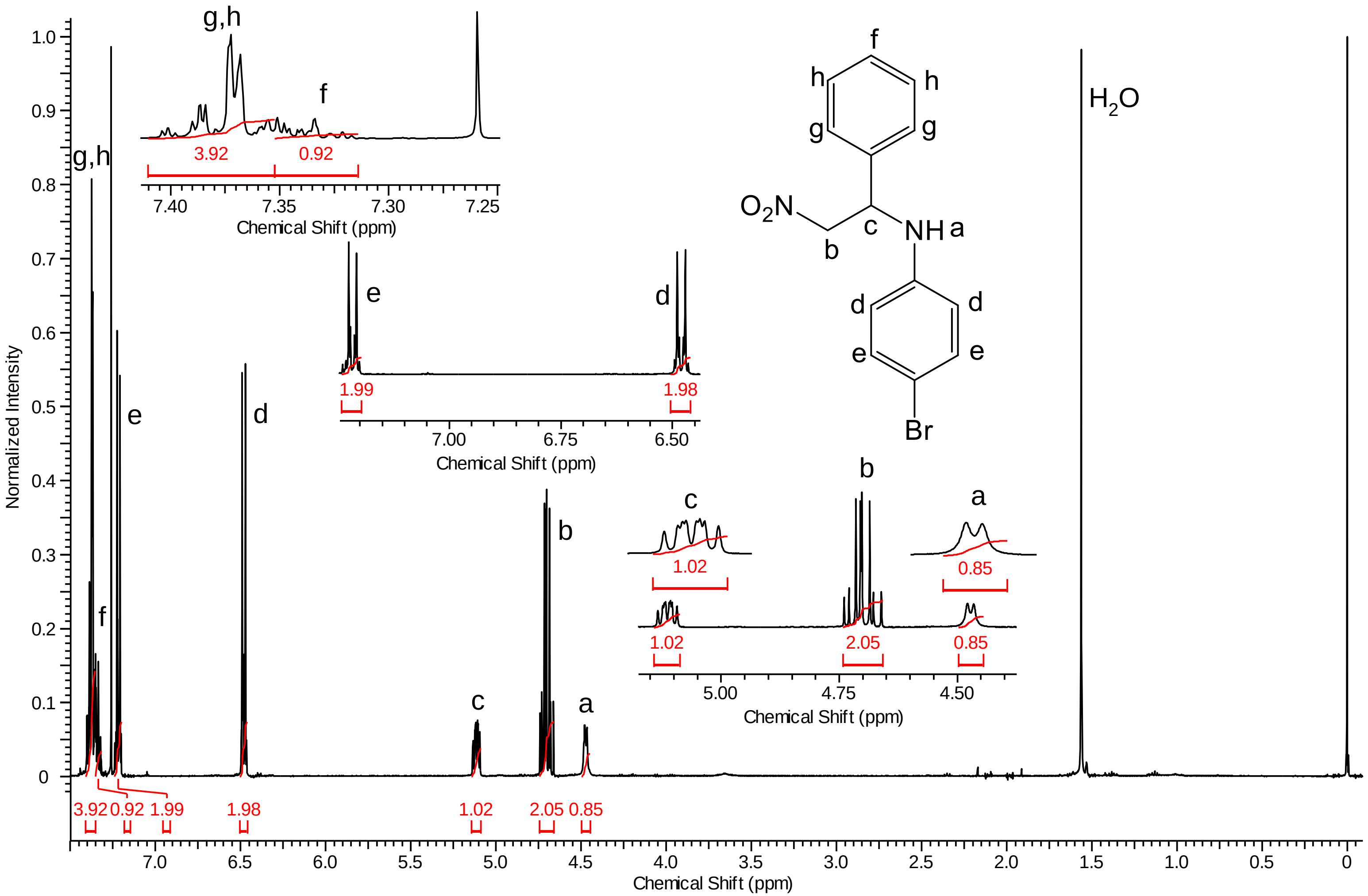

Figure 1.124: ^{1}H NMR spectrum of **8f** 4-bromo-*N*-(2-nitro-1-phenylethyl)aniline.

Table 1.17: MS data.

Compound	Formula/Mass		Parent m/z	Cone Voltage	Daughters	Collision Energy	Ion Mode
**8f**	320	1	321.03	22	104.06	22	ES+
		2	321.03	22	171.96	10	ES+
		3	321.03	22	150.05	14	ES+
		4	321.03	22	77.98	60	ES+
		5	321.03	22	260.03	12	ES+

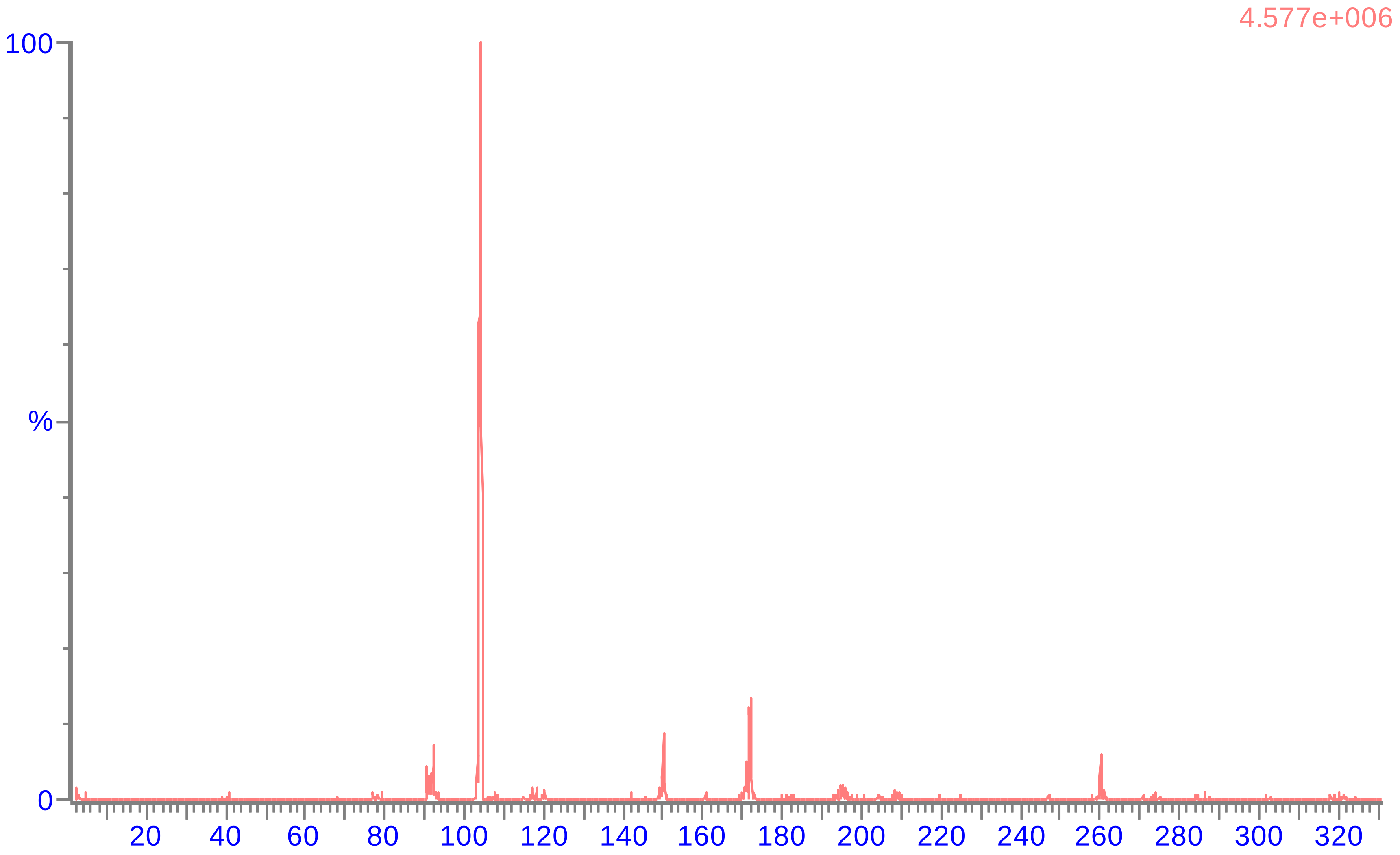

Figure 1.125: Mass spectrum for daughter fragment peak ES+, m/z 321.03 -> 104.06.

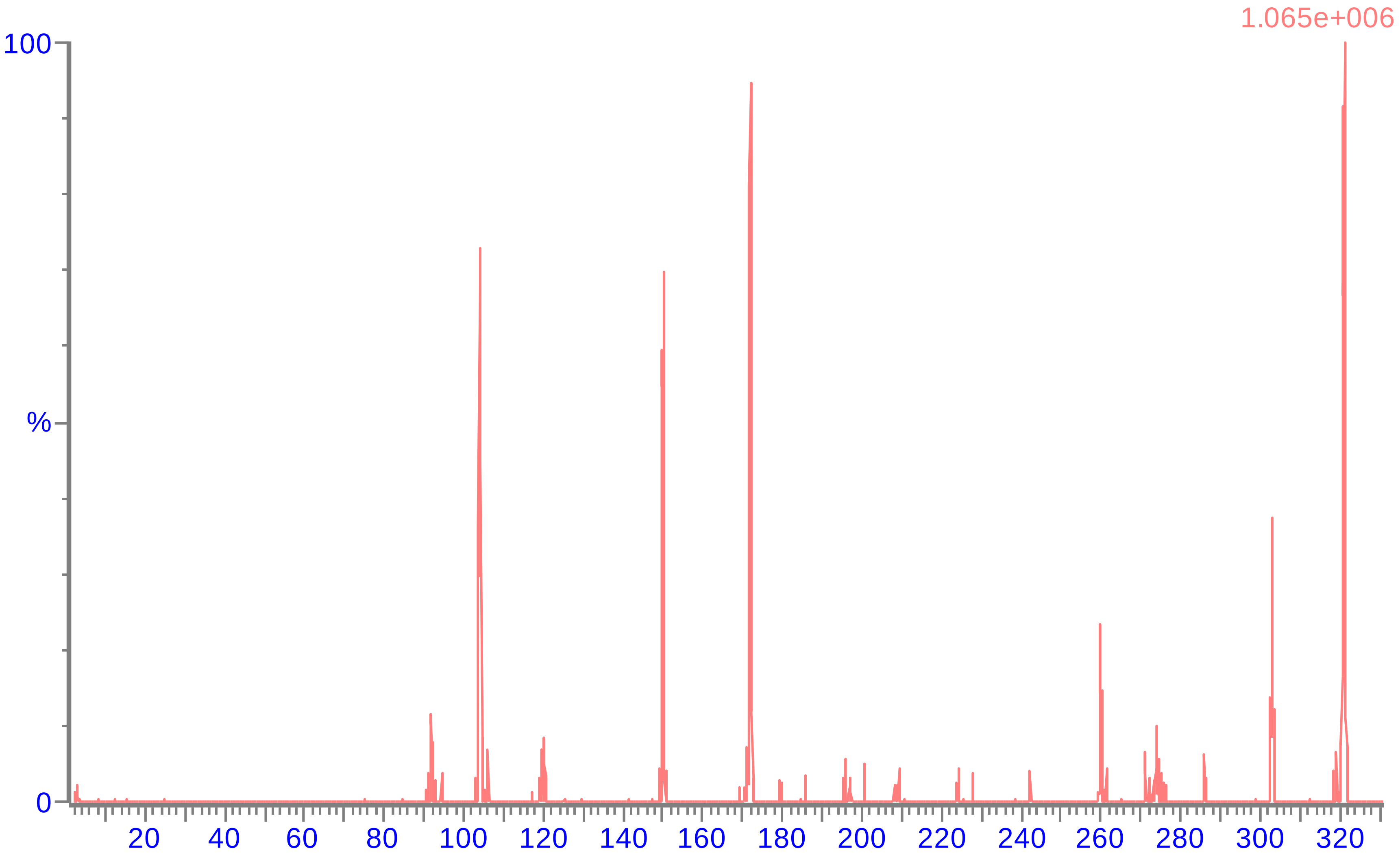

Figure 1.126: Mass spectrum for daughter fragment peak ES+, m/z 321.03 -> 171.96.

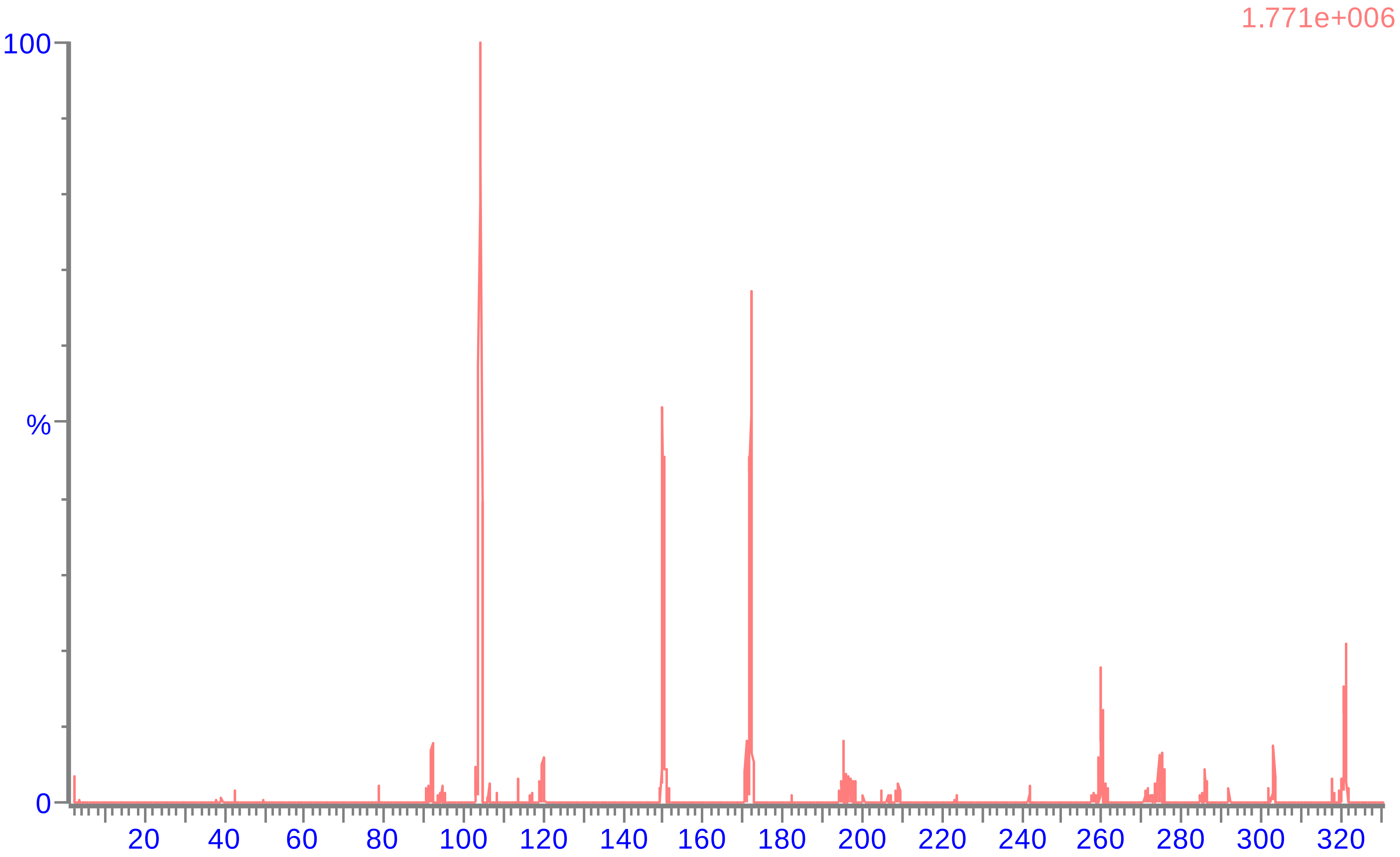

Figure 1.127: Mass spectrum for daughter fragment peak ES+, m/z 321.03 -> 150.05.

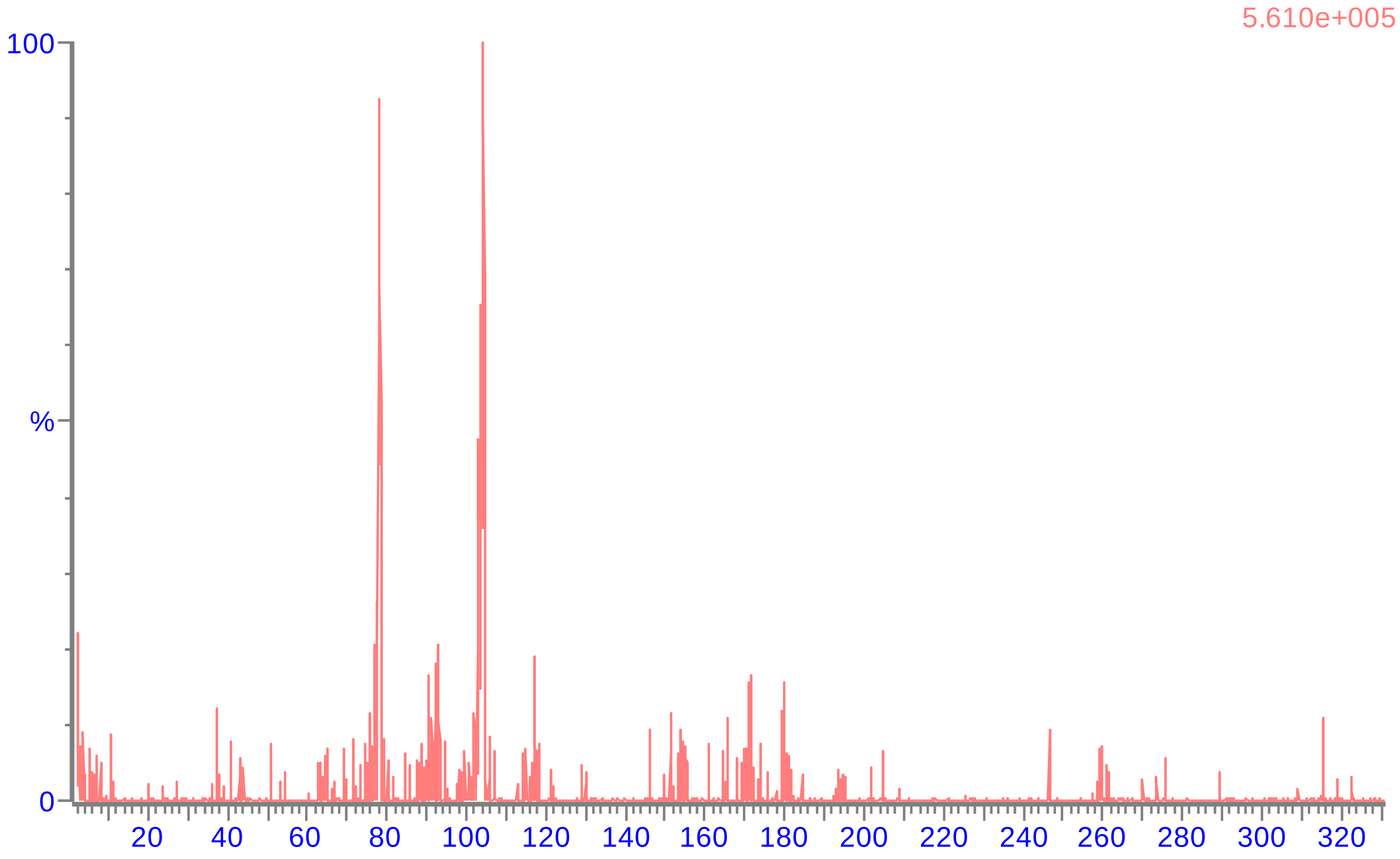

Figure 1.128: Mass spectrum for daughter fragment peak ES+, m/z 321.03 -> 77.98.

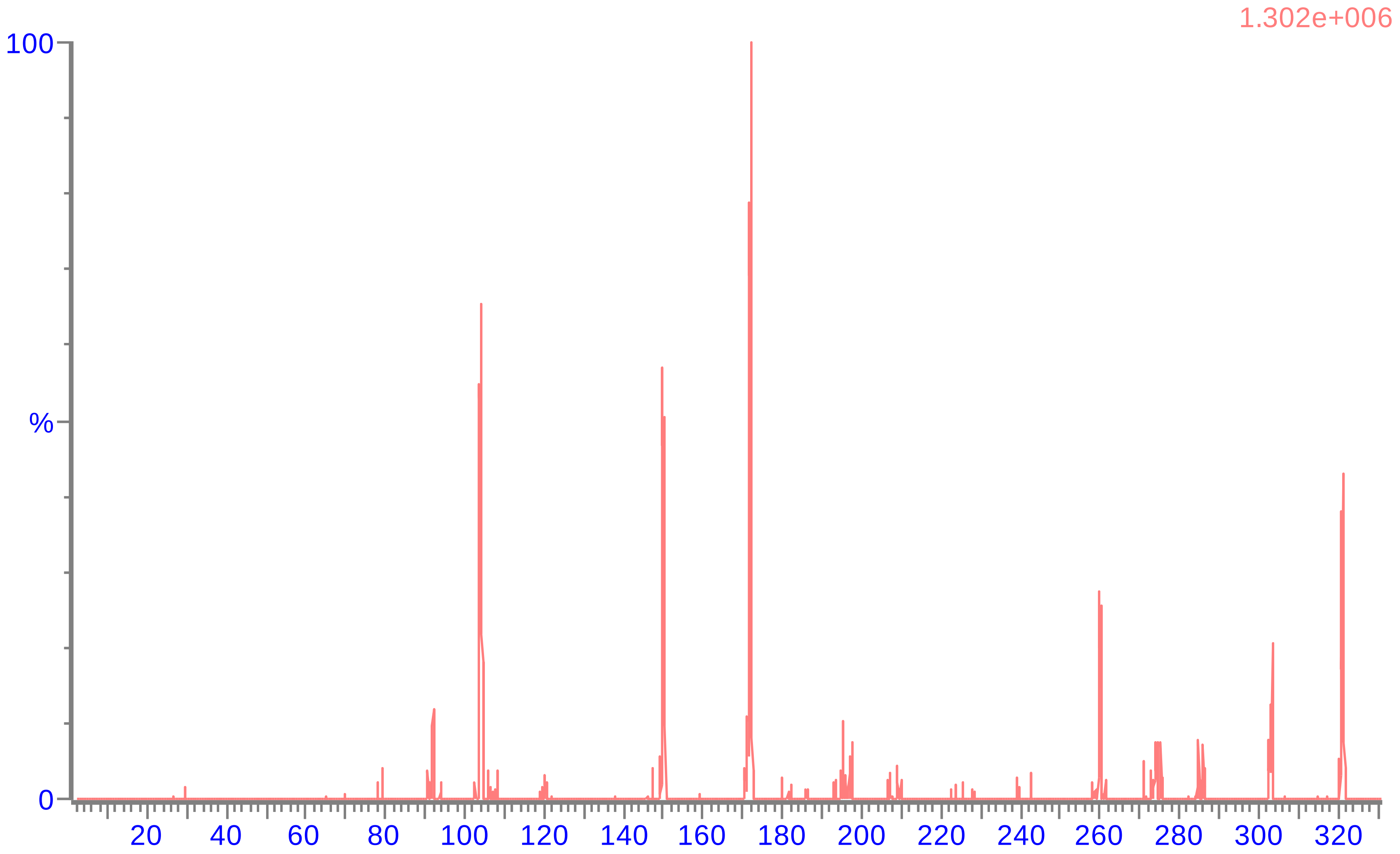

Figure 1.129: Mass spectrum for daughter fragment peak ES+, m/z 321.03 -> 260.03.

8g 4-chloro-*N*-(2-nitro-1-phenylethyl)aniline

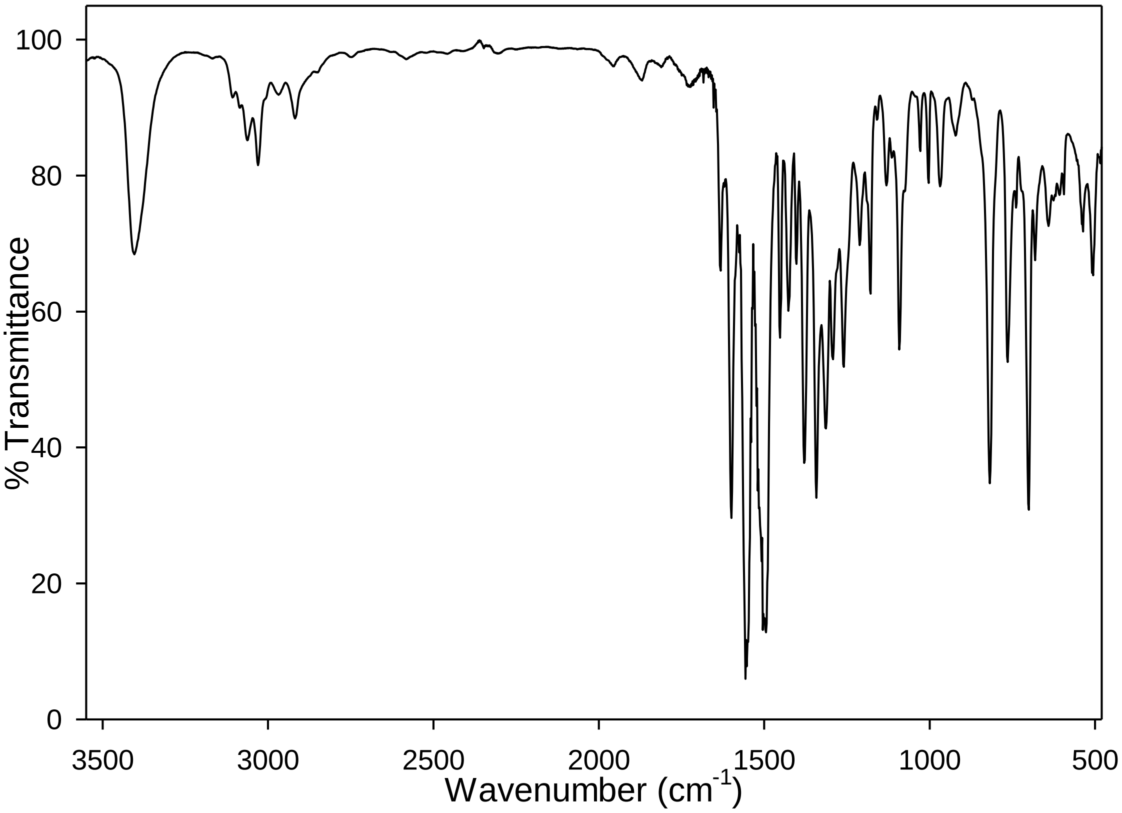

Figure 1.130: IR spectrum of **8g** 4-chloro-*N*-(2-nitro-1-phenylethyl)aniline.

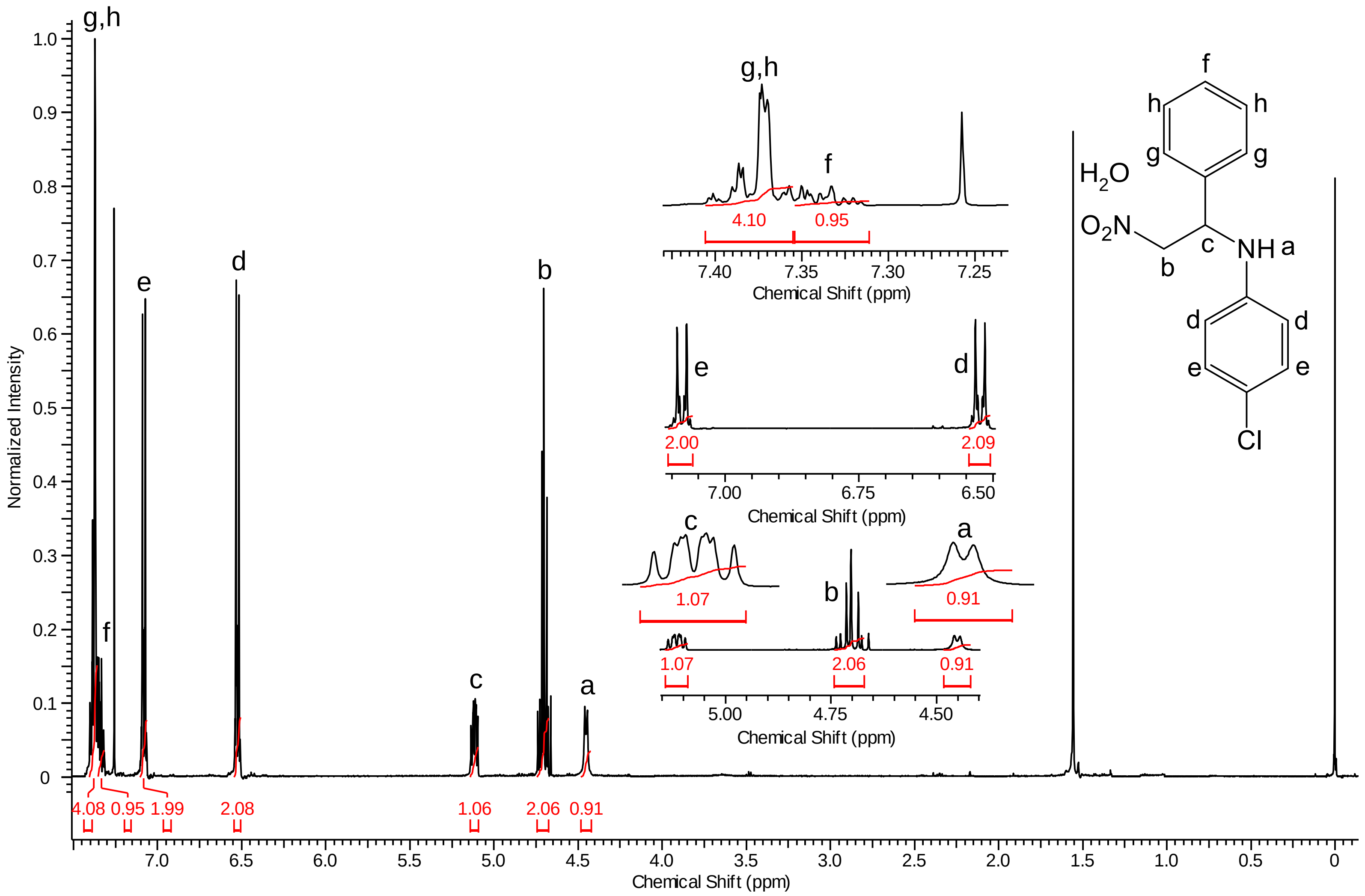

Figure 1.131: ^{1}H NMR spectrum of **8g** 4-chloro-*N*-(2-nitro-1-phenylethyl)aniline.

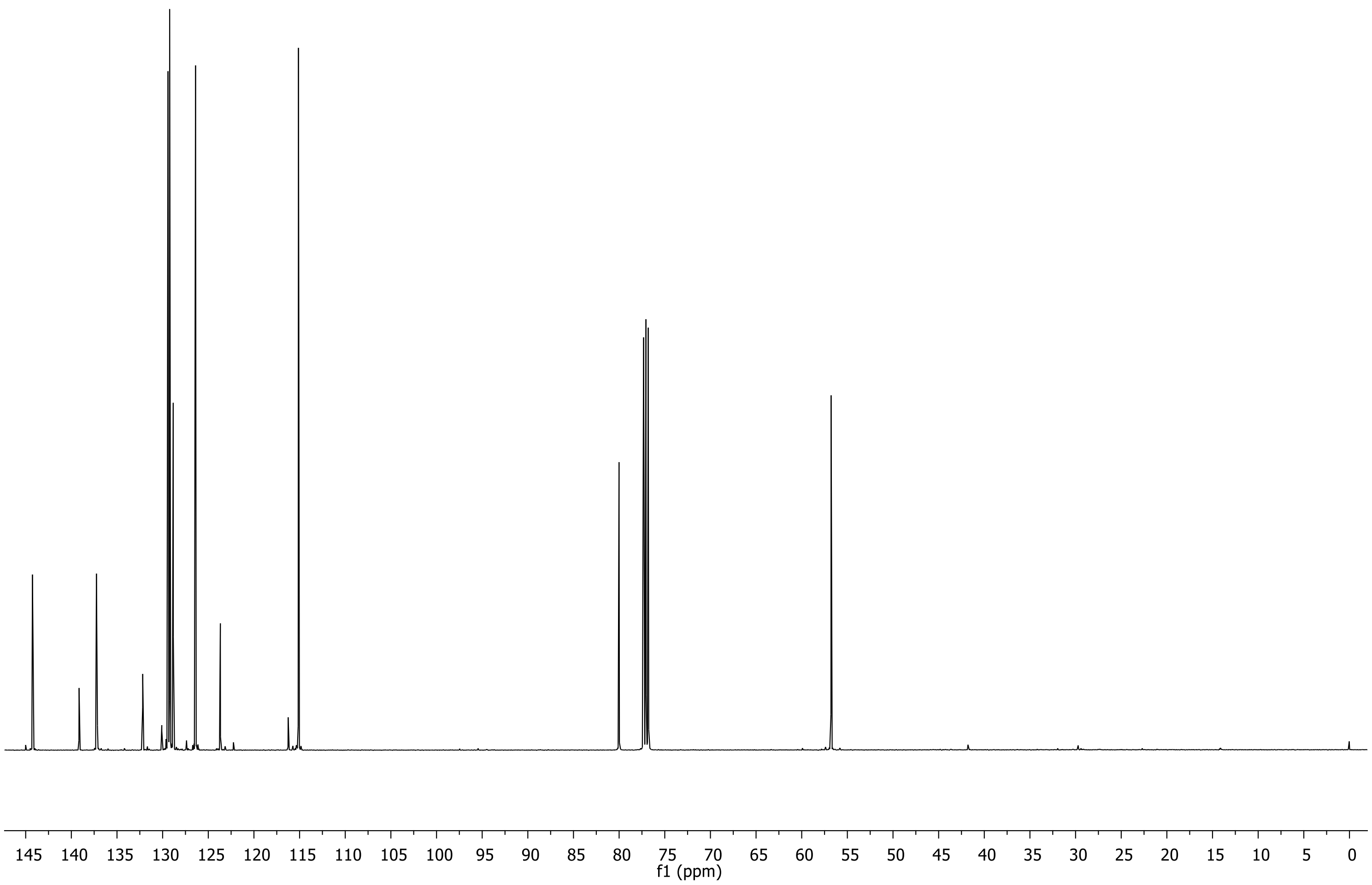

Figure 1.132: ^{1}H NMR spectrum of **8g** 4-chloro-*N*-(2-nitro-1-phenylethyl)aniline.

Table 1.18: MS data.

Compound	Formula/Mass		Parent m/z	Cone Voltage	Daughters	Collision Energy	Ion Mode
**8g**	276.8	1	277.07	24	128.02	12	ES+
		2	277.07	24	216.05	10	ES+

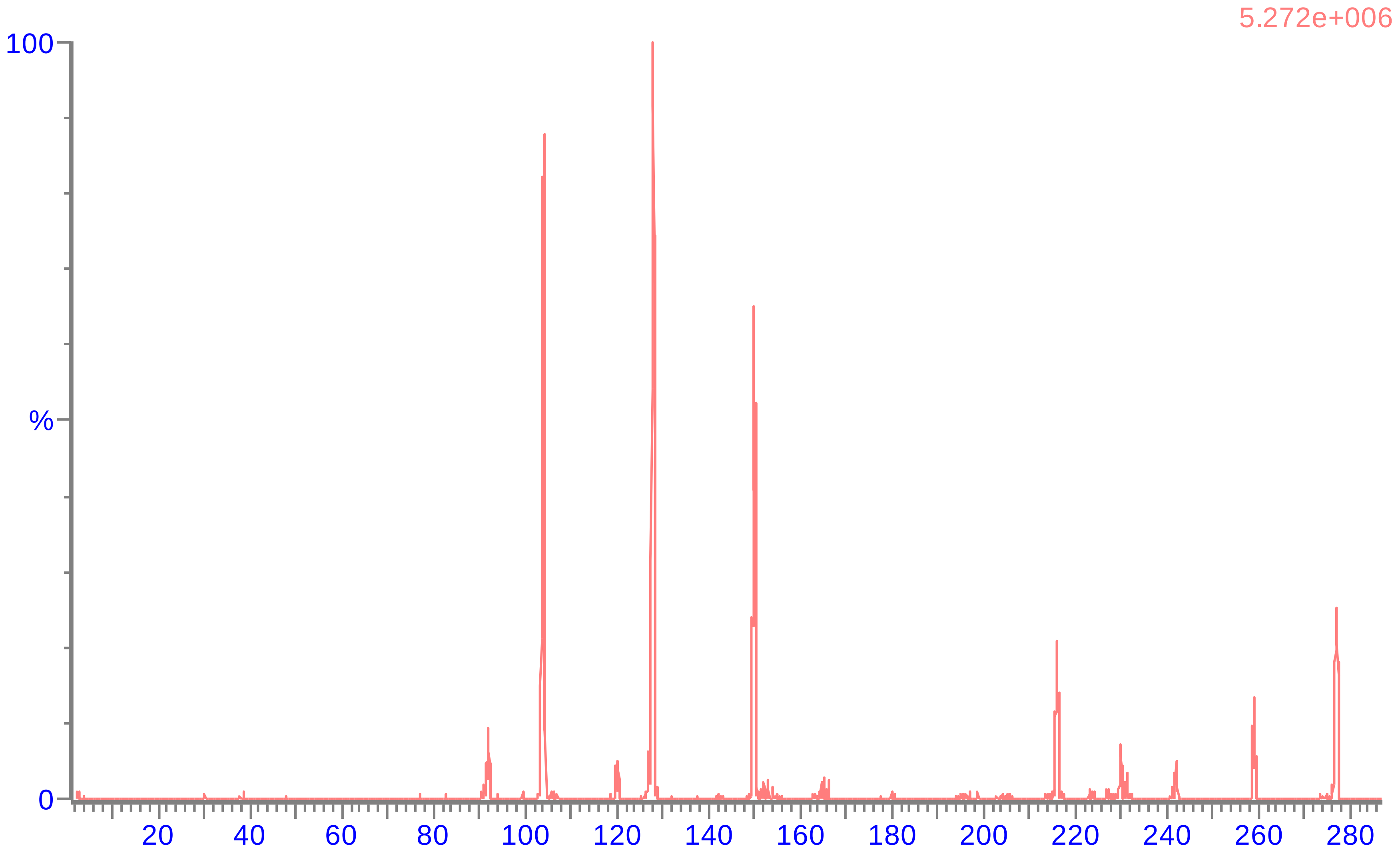

Figure 1.133: Mass spectrum for daughter fragment peak ES+, m/z 277.07 -> 128.02.

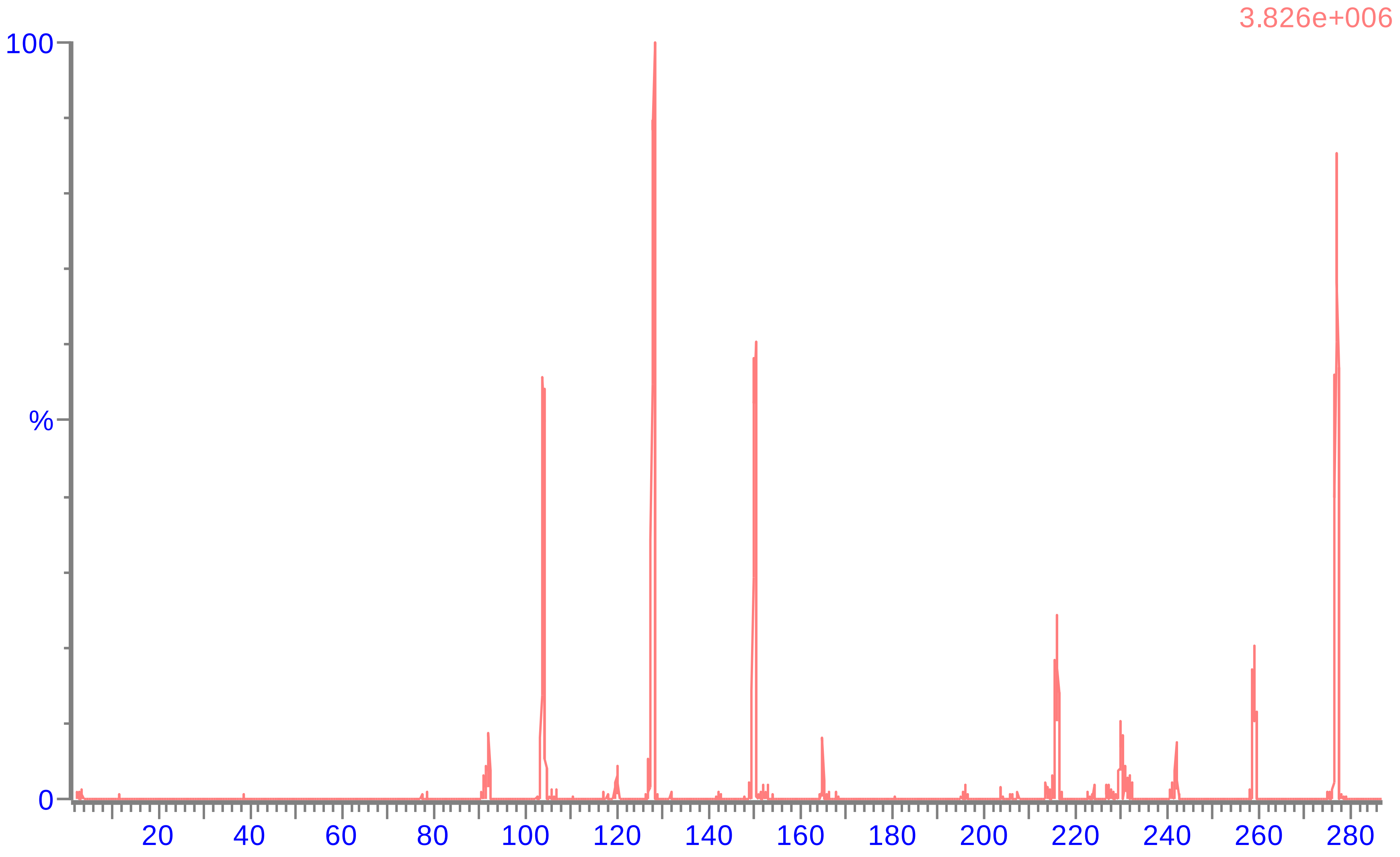

Figure 1.134: Mass spectrum for daughter fragment peak ES+, m/z 277.07 -> 216.05.

8h 1-(4-((2-nitro-1-phenylethyl)amino)phenyl)ethanone

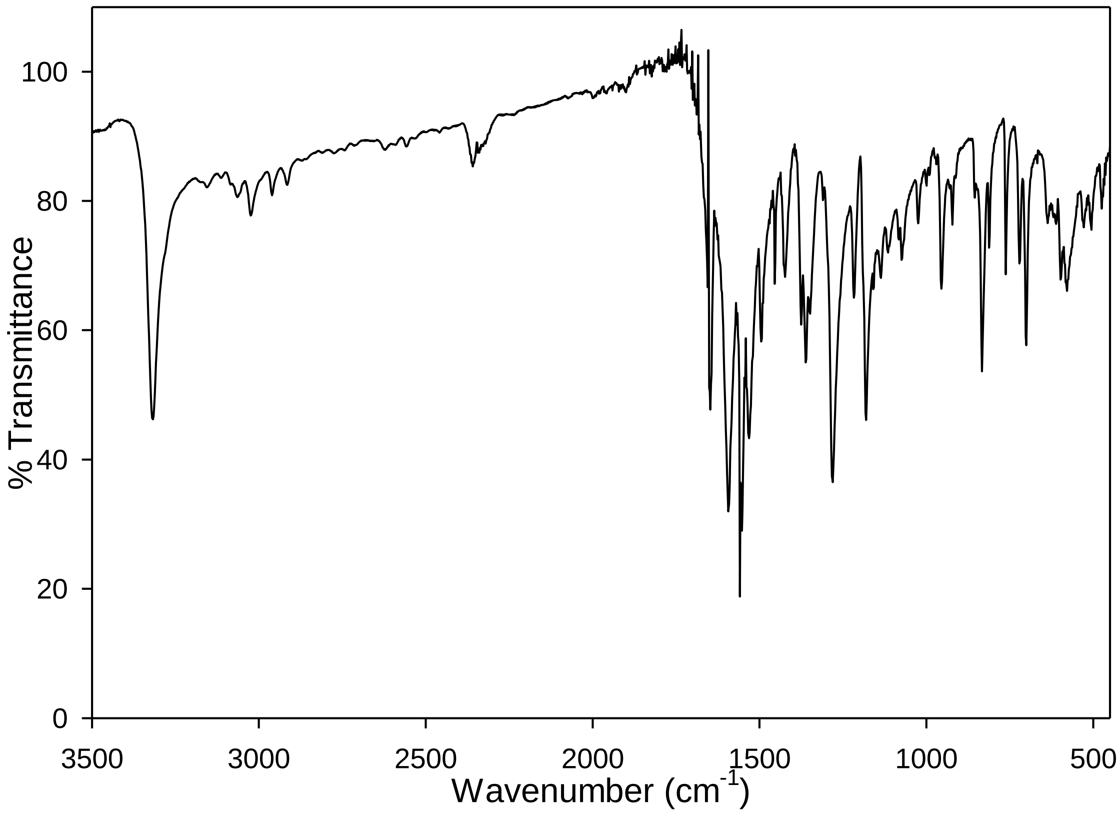

Figure 1.135: IR spectrum of **8h** 1-(4-((2-nitro-1-phenylethyl)amino)phenyl)ethanone.

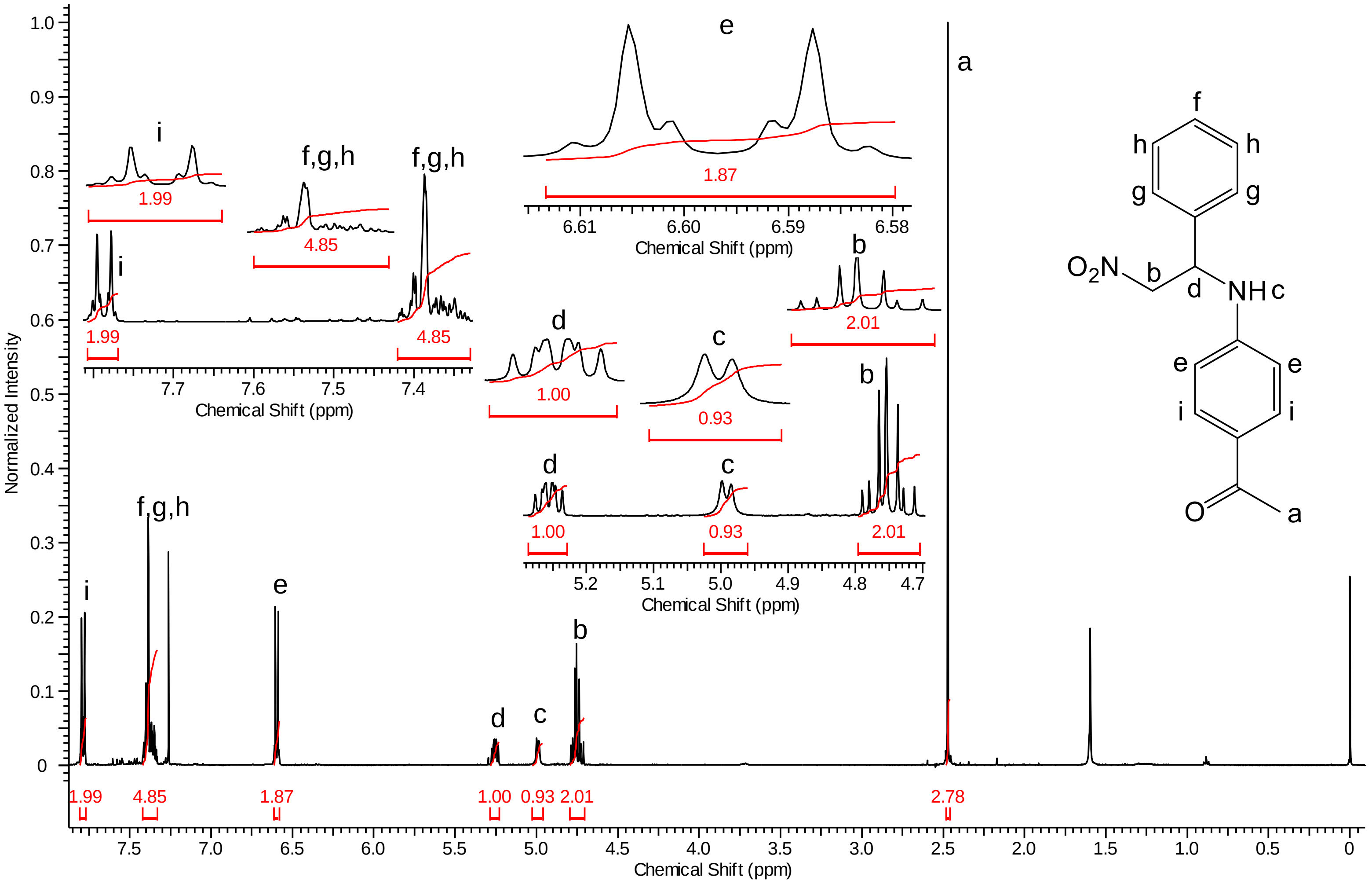

Figure 1.136: ^{1}H NMR spectrum of **8h** 1-(4-((2-nitro-1-phenylethyl)amino)phenyl)ethanone.

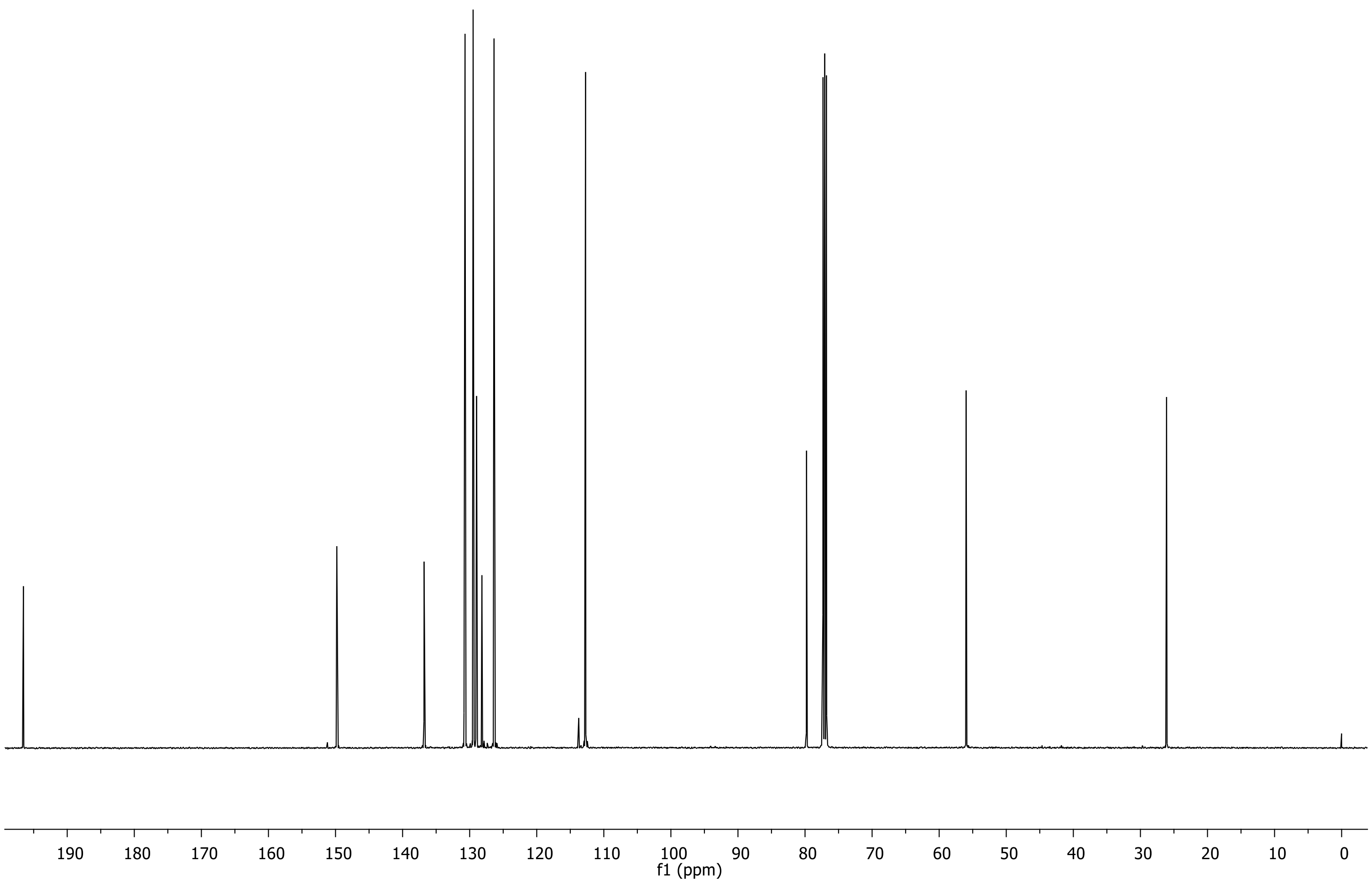

Figure 1.137: ^{13}C NMR spectrum of **8h** 1-(4-((2-nitro-1-phenylethyl)amino)phenyl)ethanone.

Table 1.19: MS data.

Compound	Formula/Mass		Parent m/z	Cone Voltage	Daughters	Collision Energy	Ion Mode
**8h**	284	1	285.10	34	135.09	22	ES+
		2	285.10	34	224.14	16	ES+
		3	285.10	34	42.99	48	ES+
		4	285.10	34	93.08	40	ES+
		5	285.10	34	104.07	32	ES+

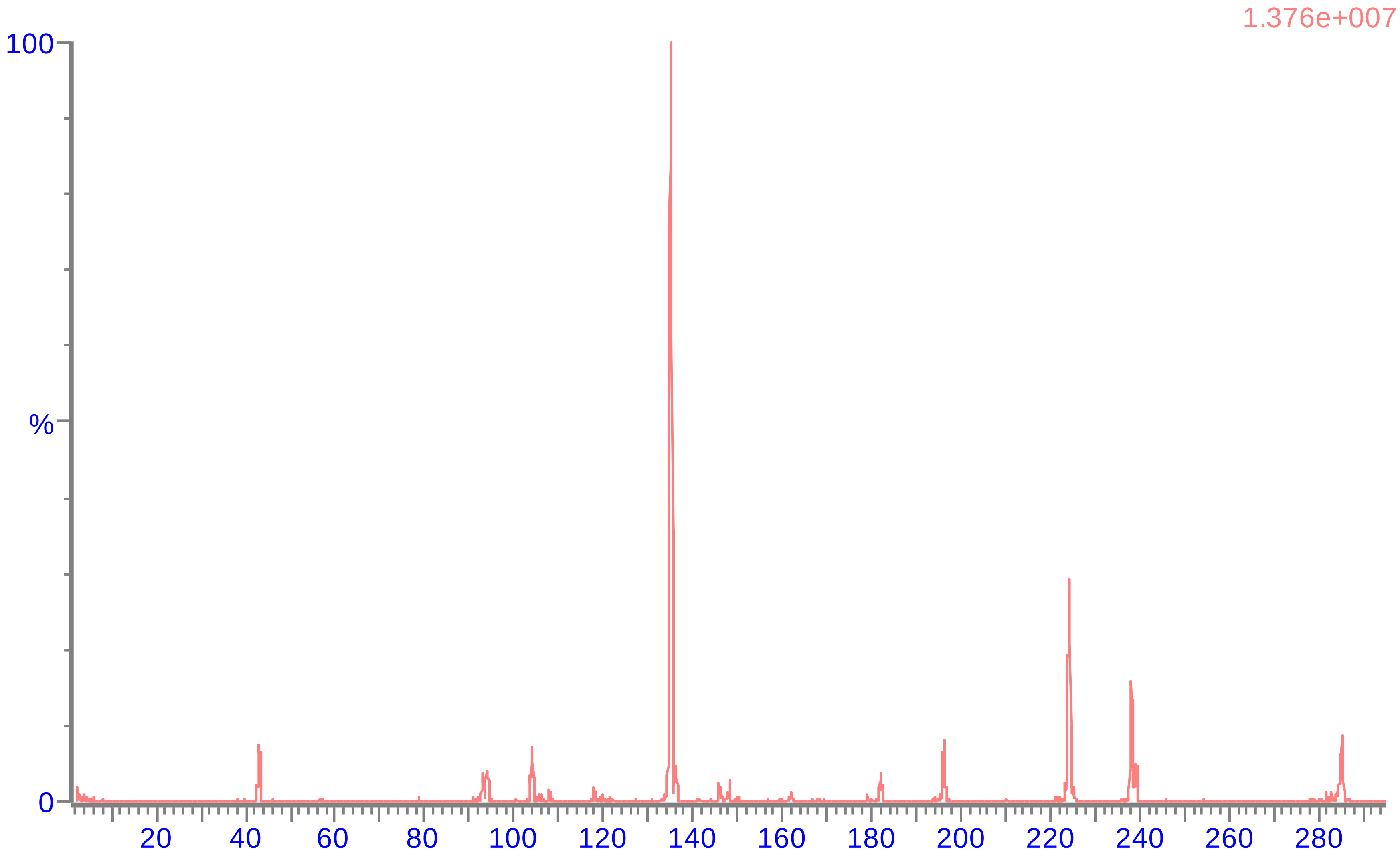

Figure 1.138: Mass spectrum for daughter fragment peak ES+, m/z 285.10 -> 135.09.

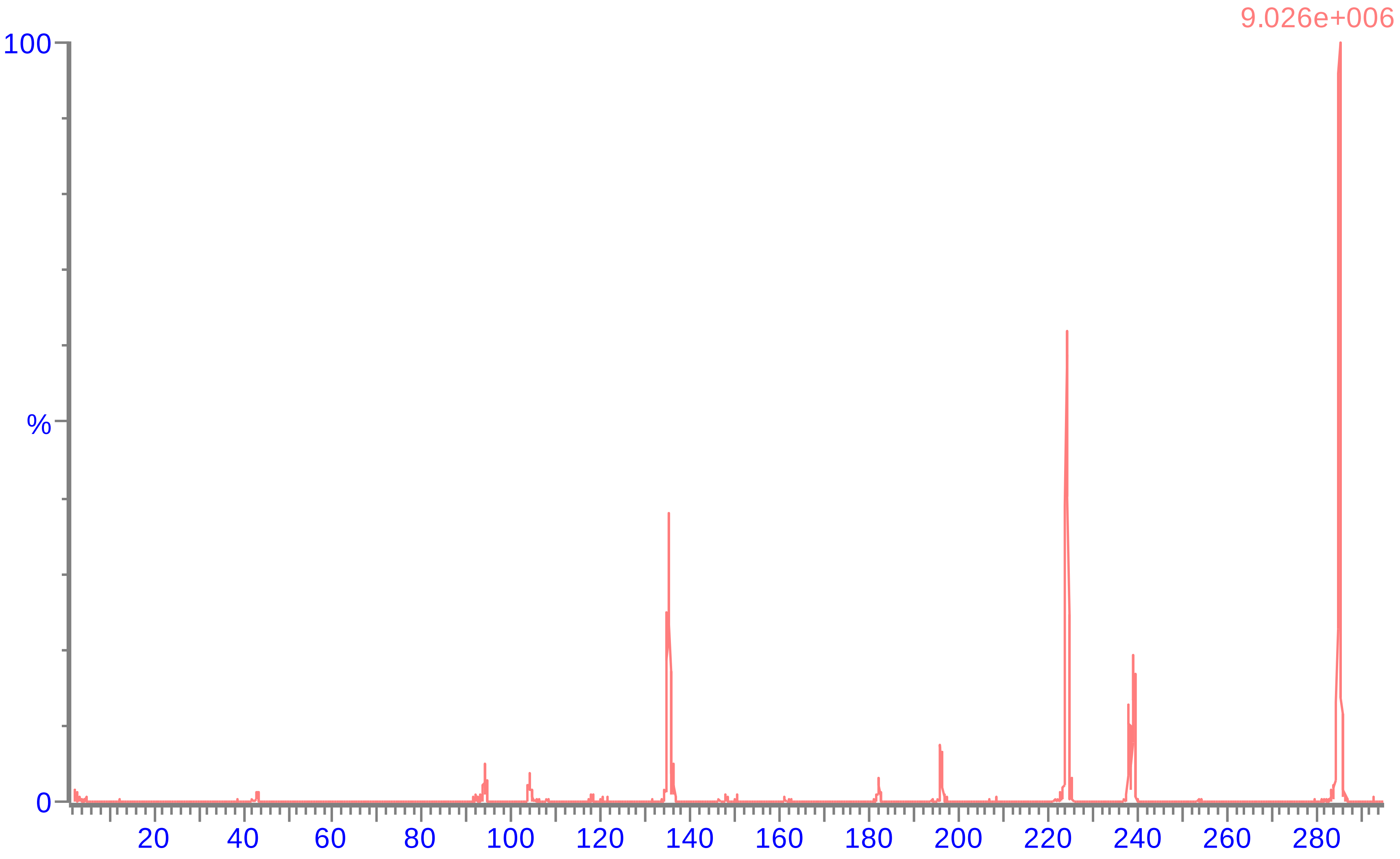

Figure 1.139: Mass spectrum for daughter fragment peak ES+, m/z 285.10 -> 224.14.

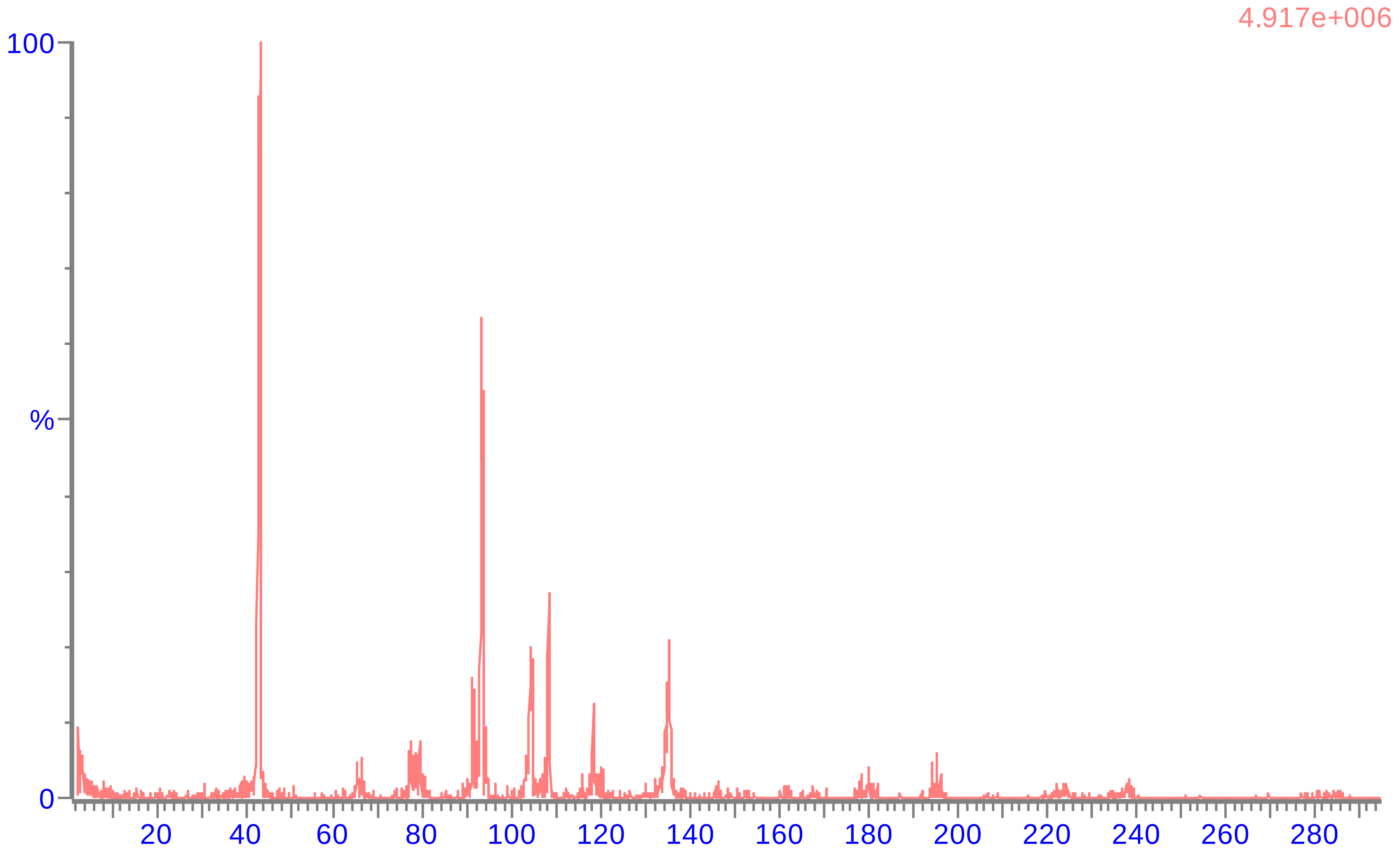

Figure 1.140: Mass spectrum for daughter fragment peak ES+, m/z 285.10 -> 42.99.

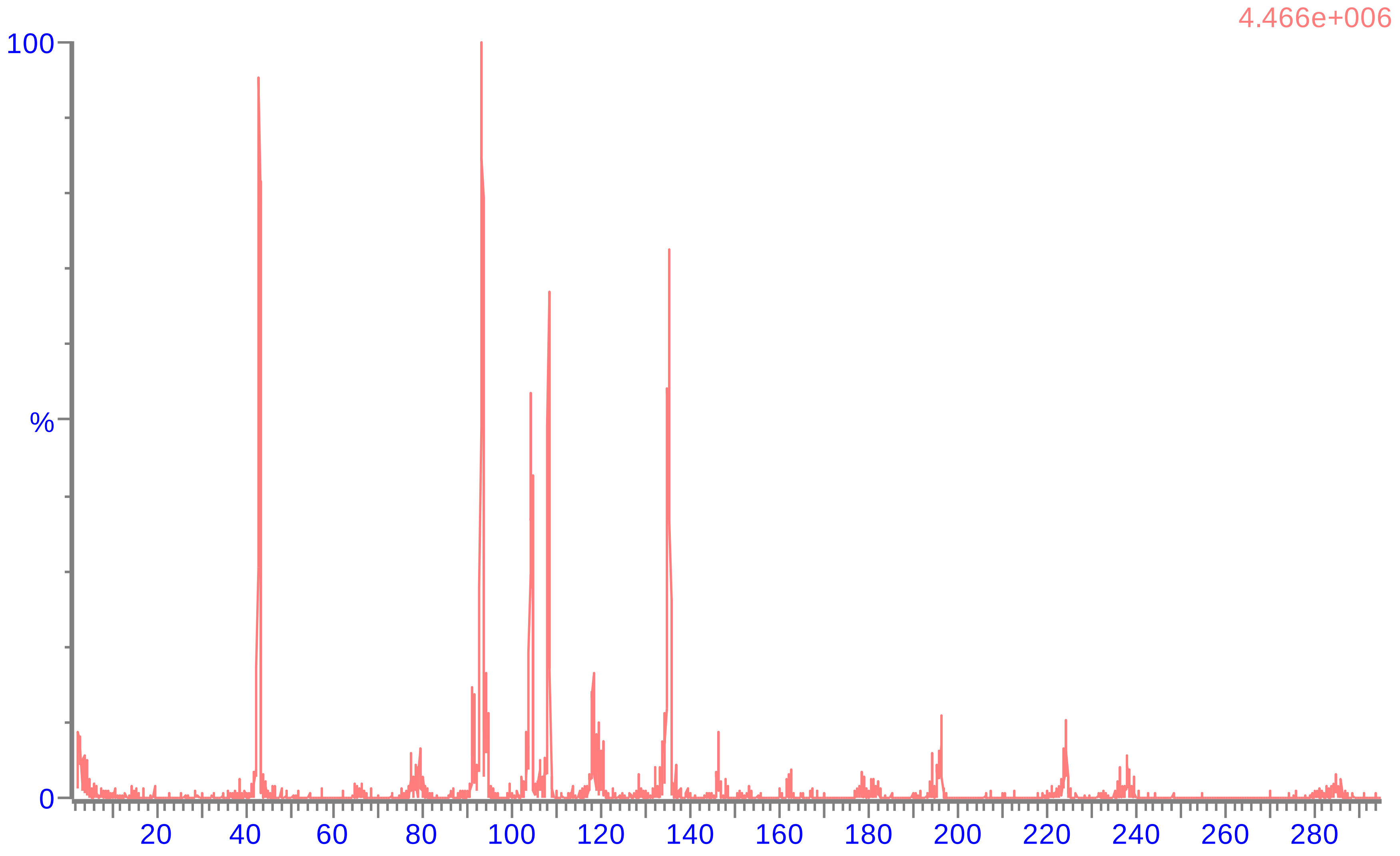

Figure 1.141: Mass spectrum for daughter fragment peak ES+, m/z 285.10 -> 93.08.

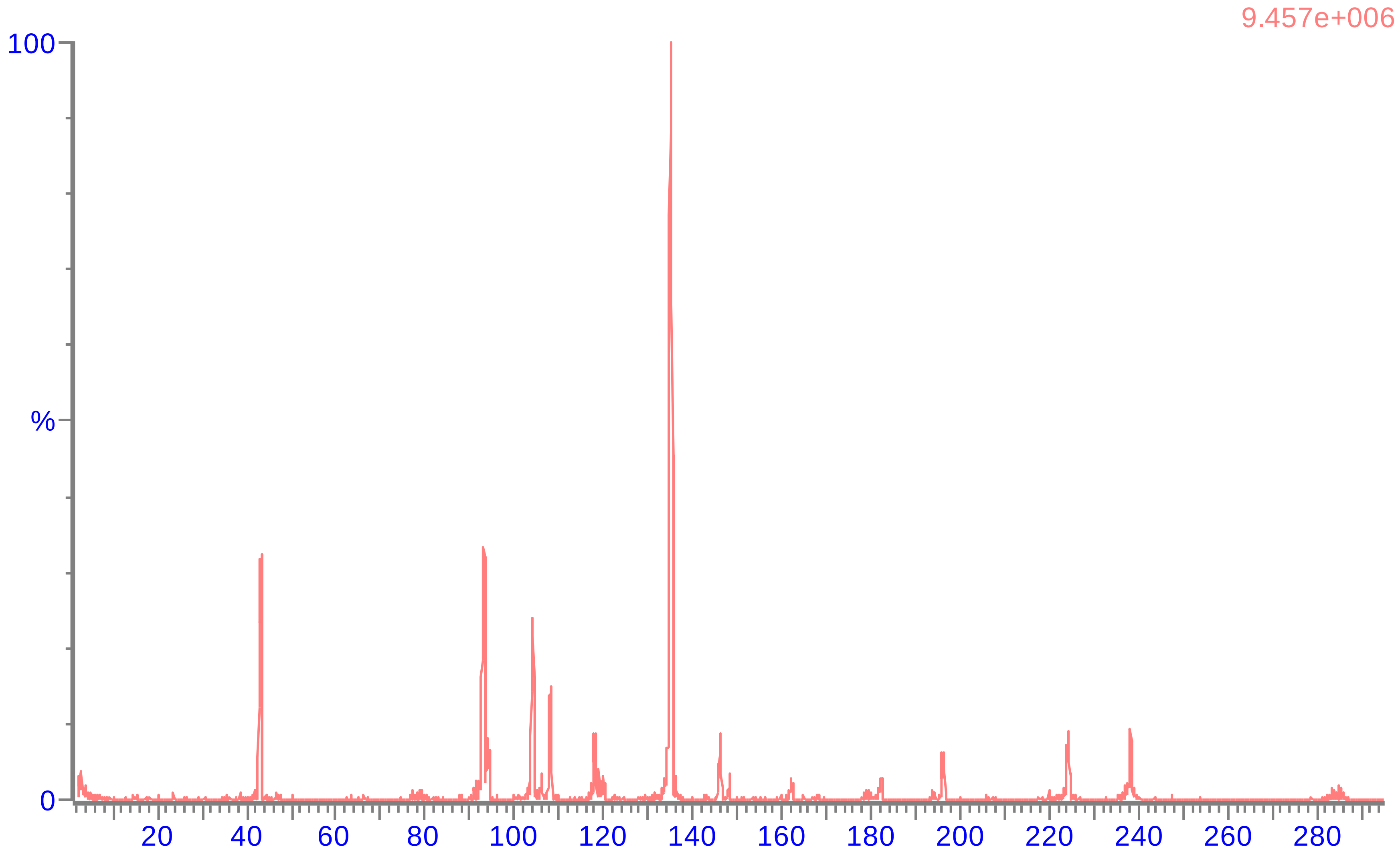

Figure 1.142: Mass spectrum for daughter fragment peak ES+, m/z 285.10 -> 104.07.

8i 4-((2-nitro-1-phenylethyl)amino)benzonitrile

Table 1.20: MS data.

Compound	Formula/Mass		Parent m/z	Cone Voltage	Daughters	Collision Energy	Ion Mode
**8i**	267	1	268.10	38	118.10	26	ES+
		2	268.10	38	221.12	24	ES+
		3	268.10	38	91.05	46	ES+
		4	268.10	38	143.08	36	ES+

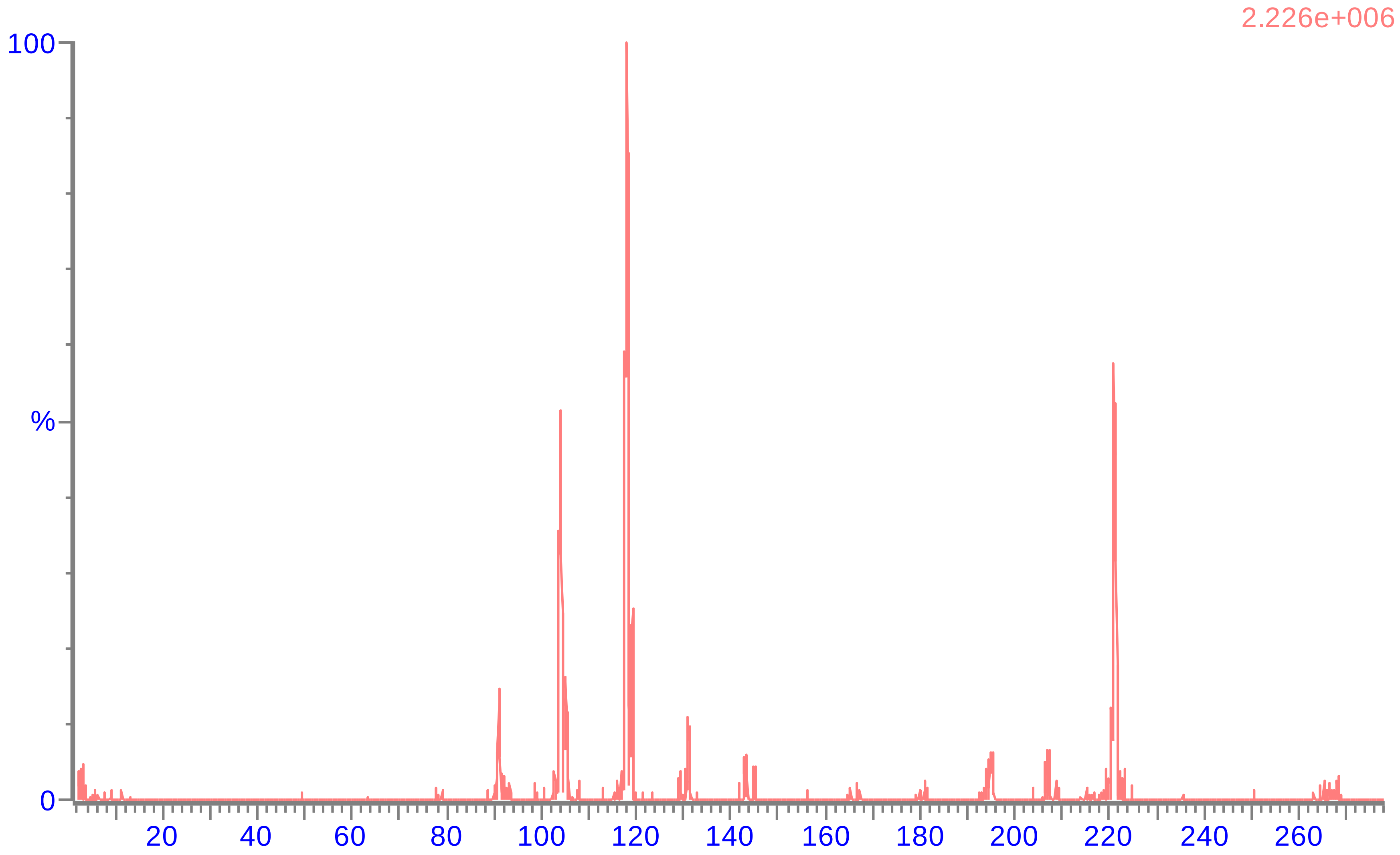

Figure 1.143: Mass spectrum for daughter fragment peak ES+, m/z 268.10 -> 118.10.

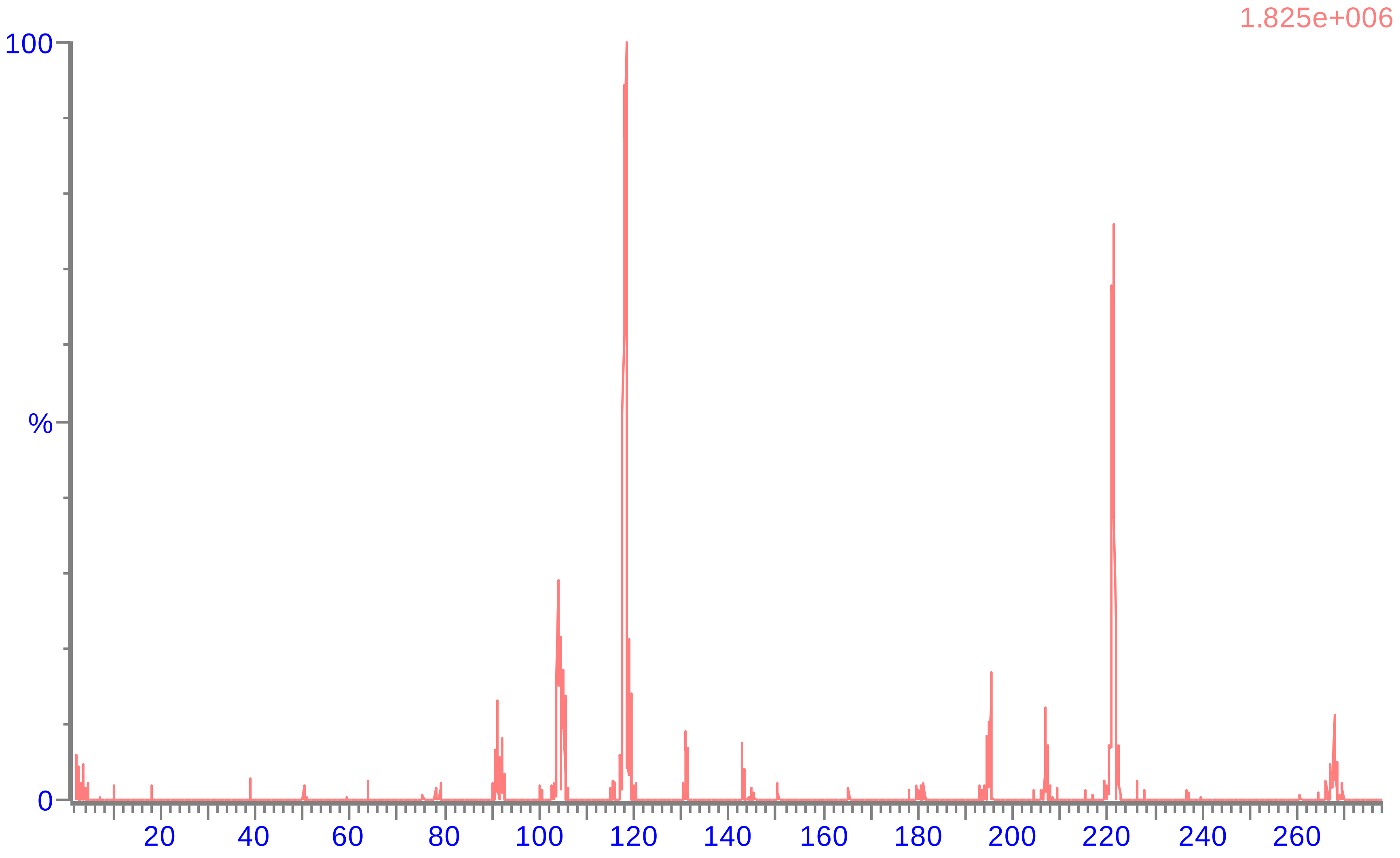

Figure 1.144: Mass spectrum for daughter fragment peak ES+, m/z 268.10 -> 221.12.

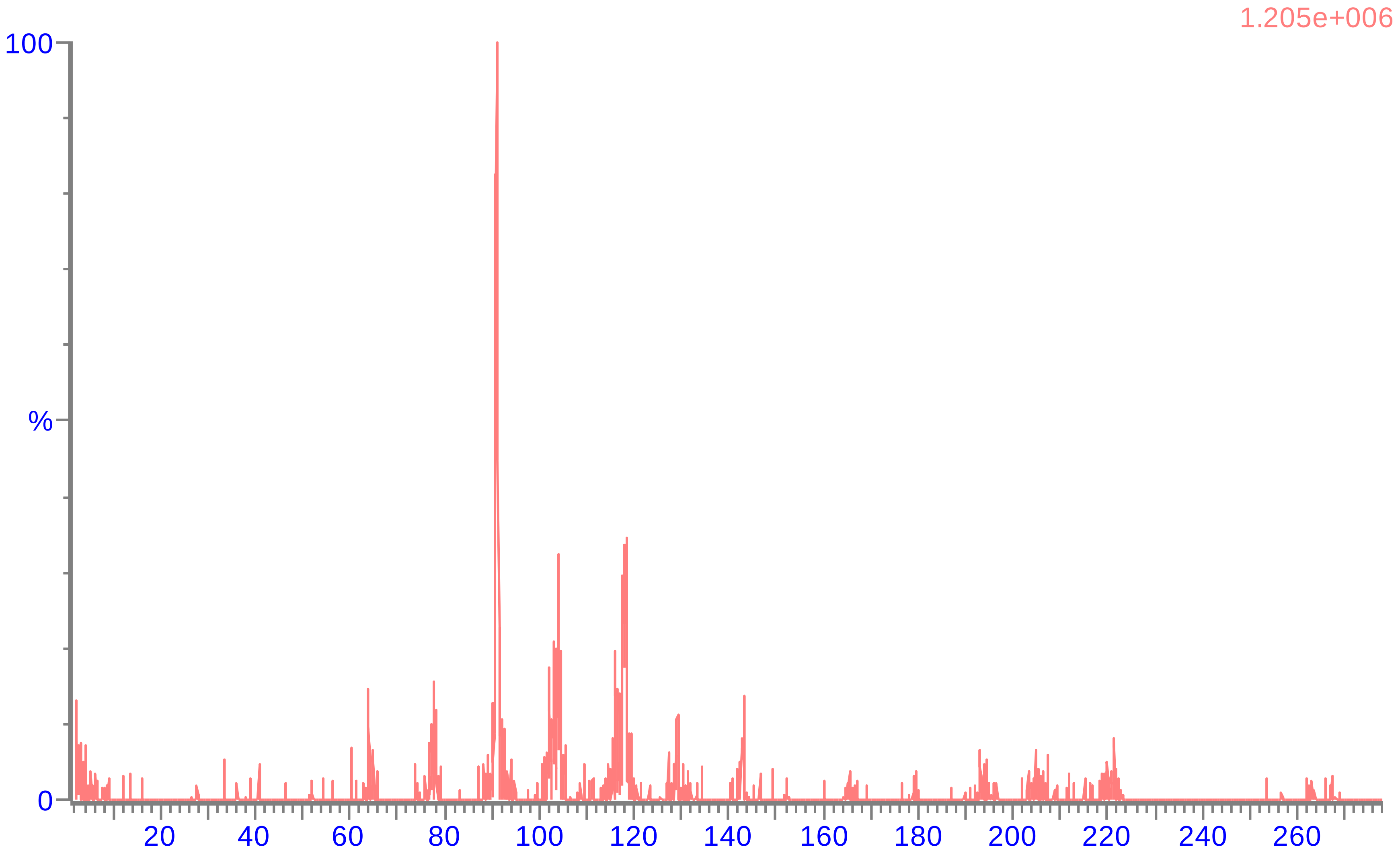

Figure 1.145: Mass spectrum for daughter fragment peak ES+, m/z 268.10 -> 91.05.

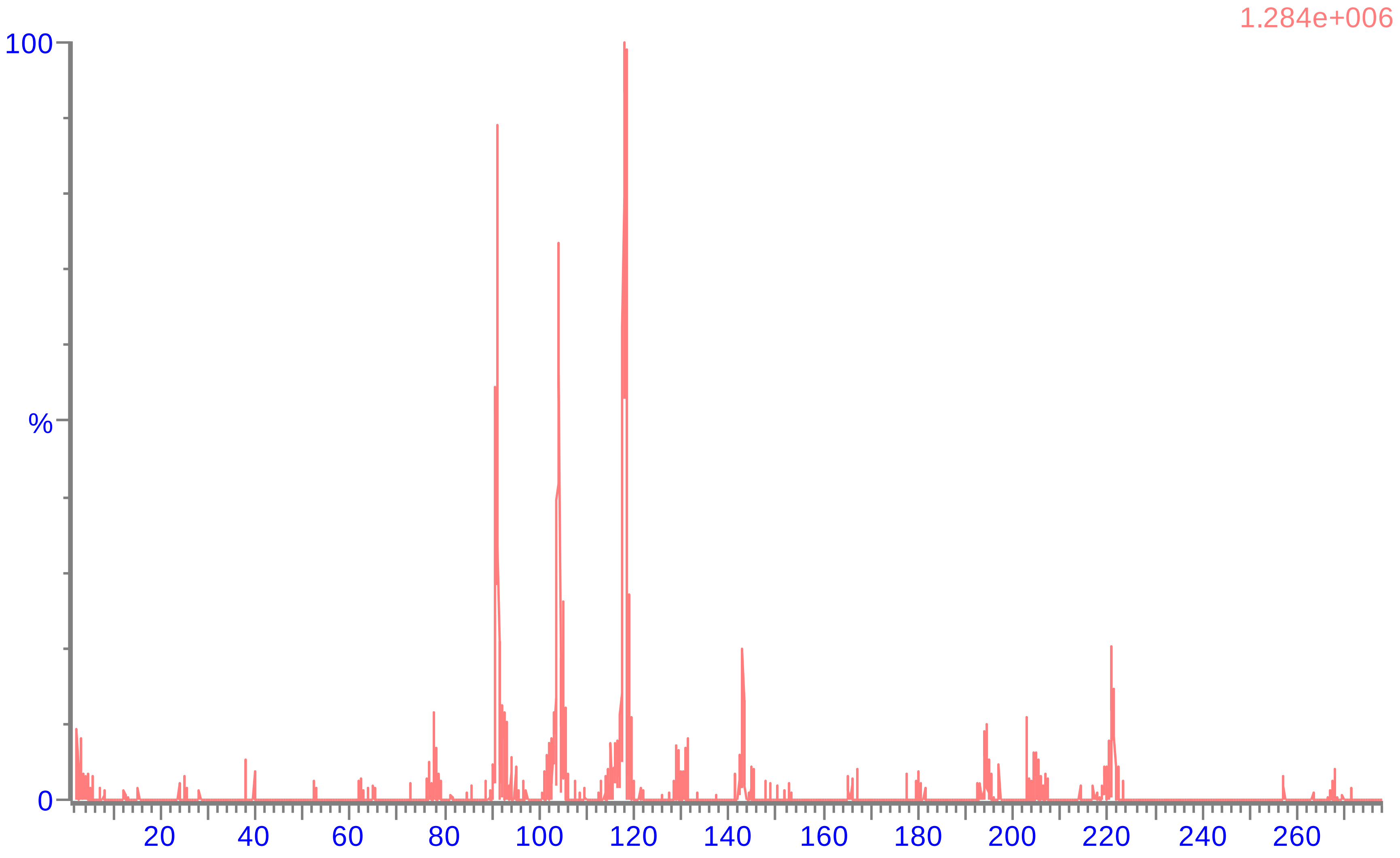

Figure 1.146: Mass spectrum for daughter fragment peak ES+, m/z 268.10 -> 143.08.

8j 3-nitro-*N*-(2-nitro-1-phenylethyl)aniline

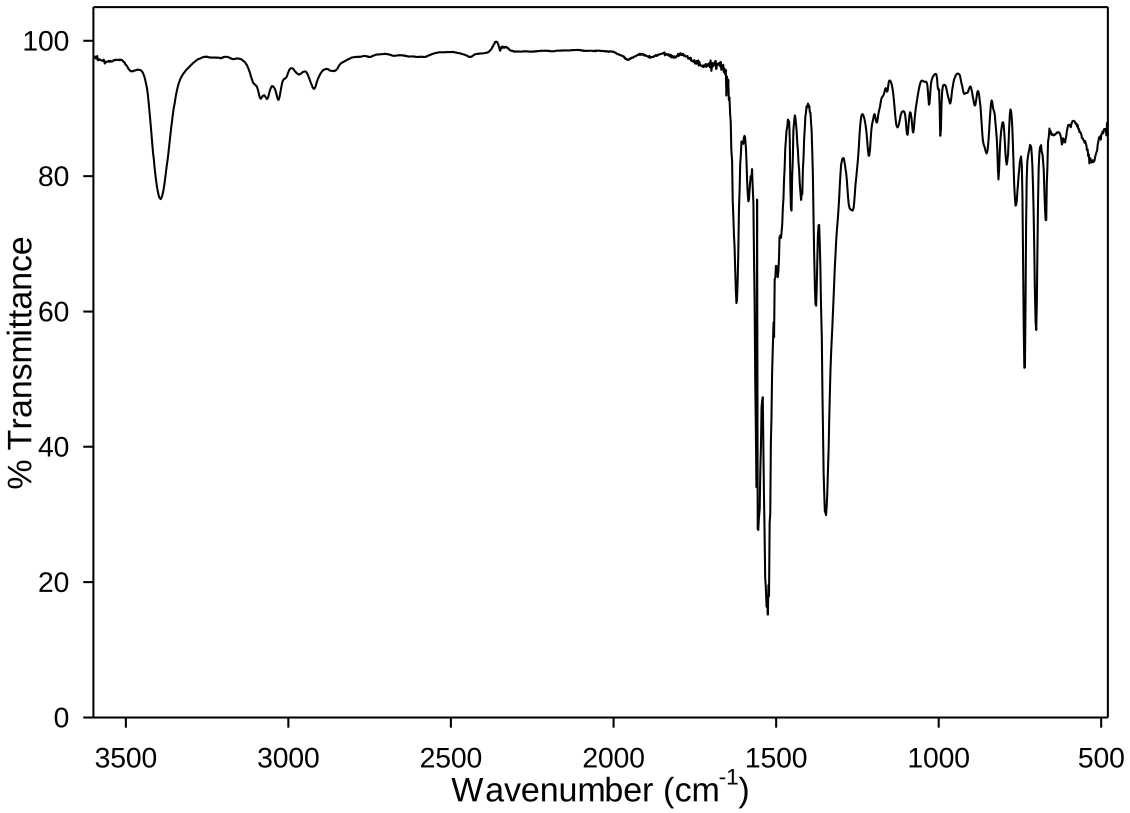

Figure 1.147: IR spectrum of **8j** 3-nitro-*N*-(2-nitro-1-phenylethyl)aniline.

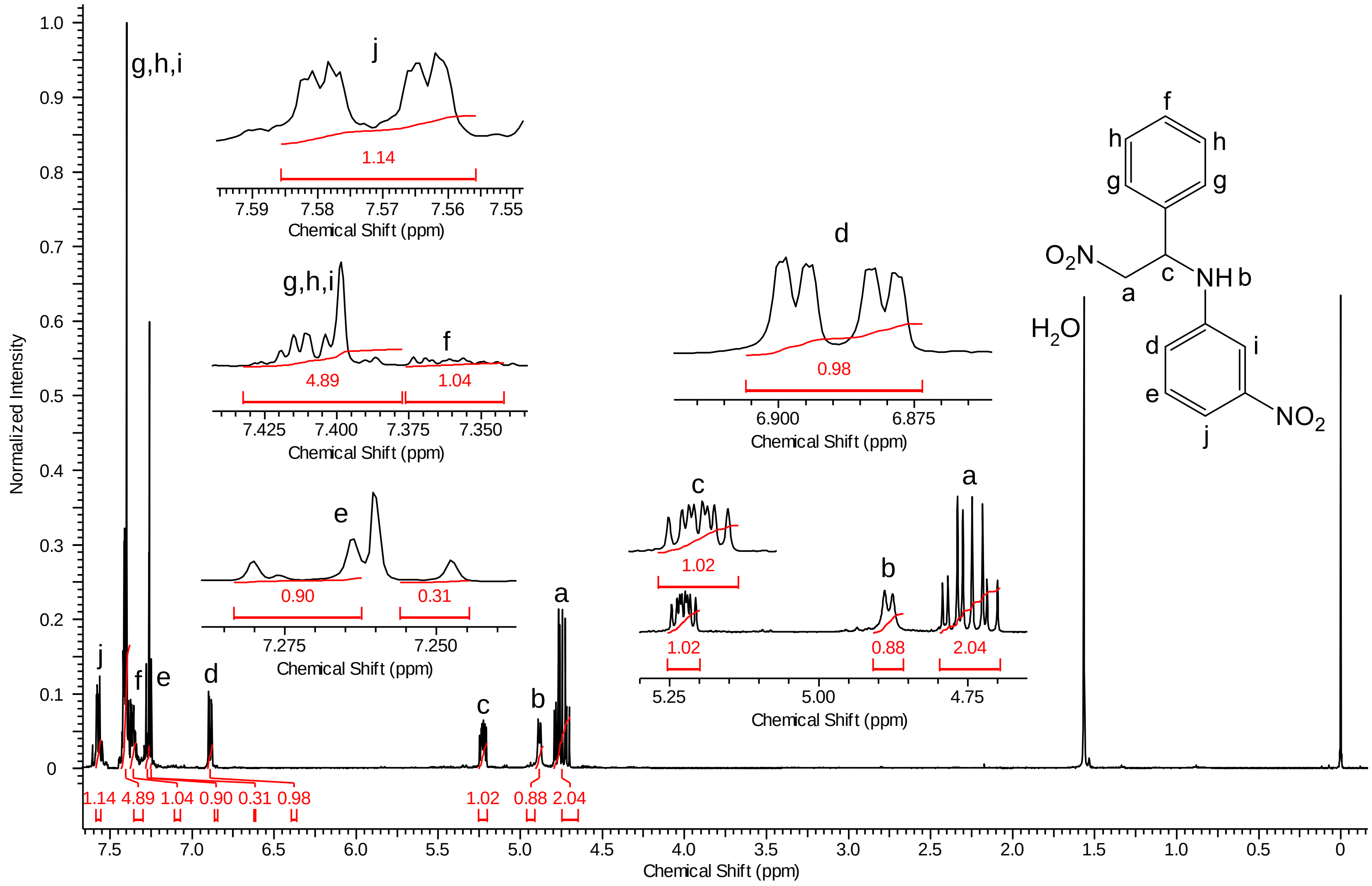

Figure 1.148: ^{1}H NMR spectrum of **8j** 3-nitro-*N*-(2-nitro-1-phenylethyl)aniline.

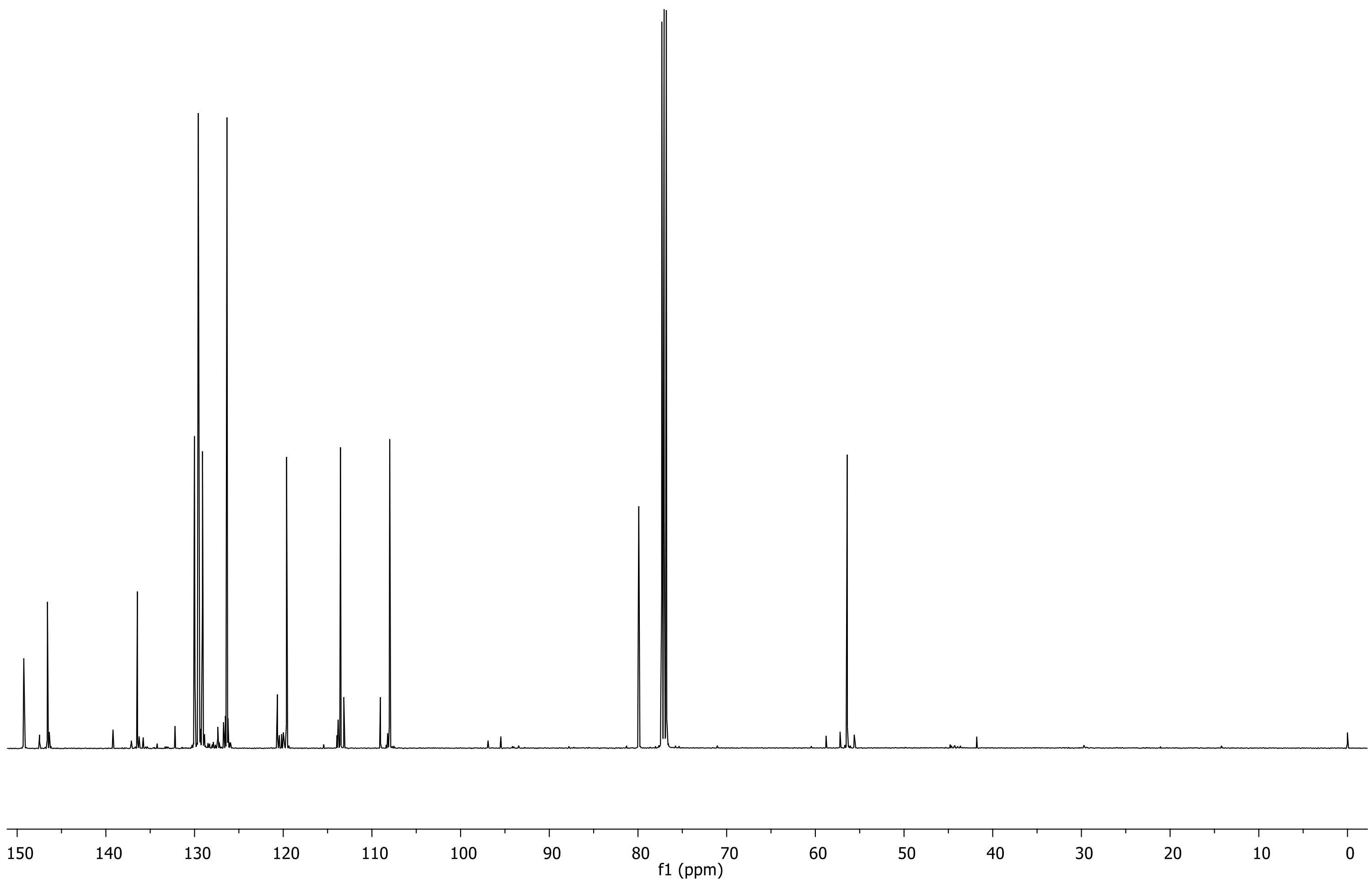

Figure 1.149: ^{13}C NMR spectrum of **8j** 3-nitro-*N*-(2-nitro-1-phenylethyl)aniline.

Table 1.21: MS data.

Compound	Formula/Mass		Parent m/z	Cone Voltage	Daughters	Collision Energy	Ion Mode
**8j**	287.3	1	288.08	18	104.06	28	ES+
		2	288.08	18	150.05	8	ES+
		3	288.08	18	138.99	8	ES+
		4	288.08	18	77.99	48	ES+
		5	288.08	18	91.97	14	ES+

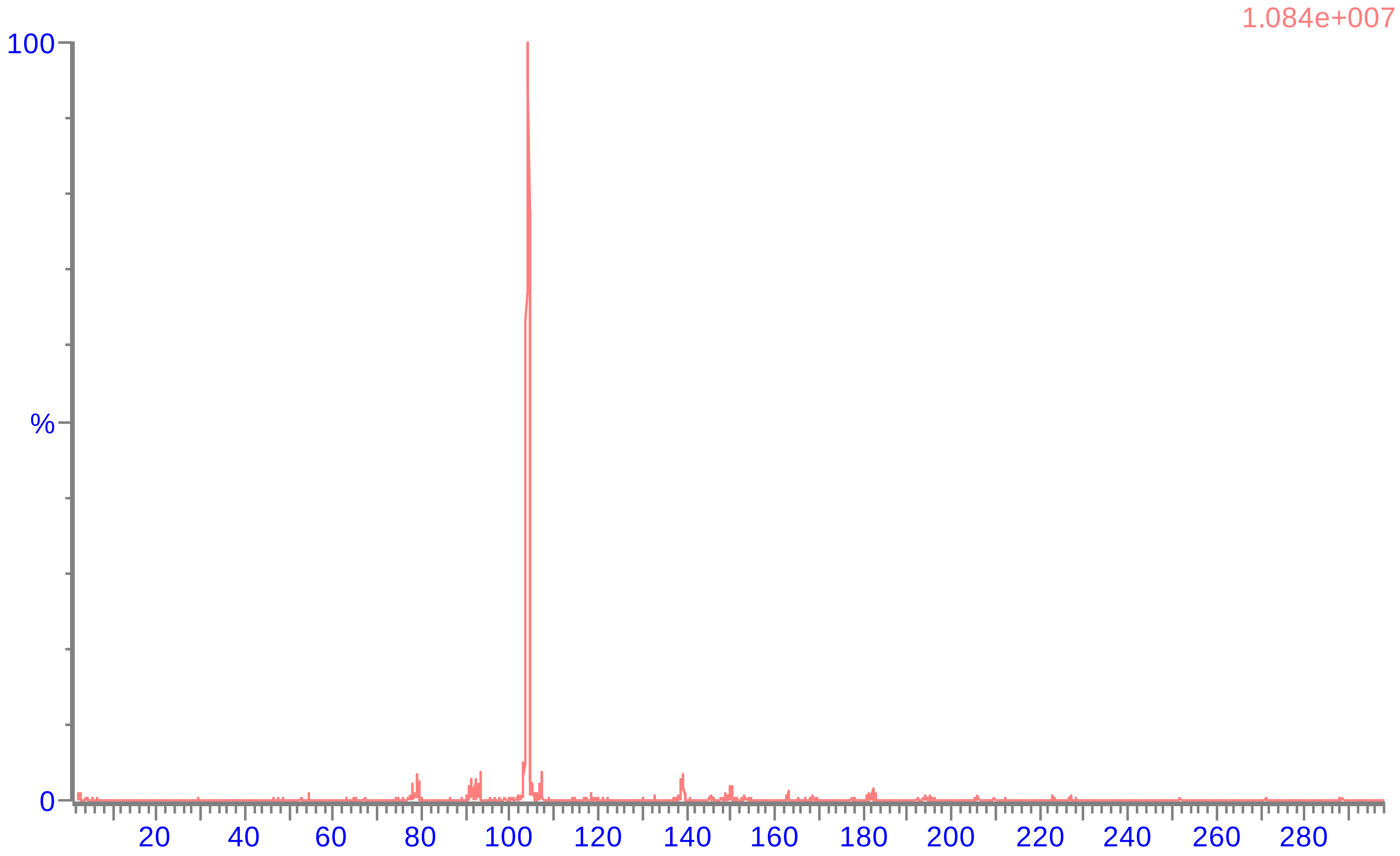

Figure 1.150: Mass spectrum for daughter fragment peak ES+, m/z 288.08 -> 104.06.

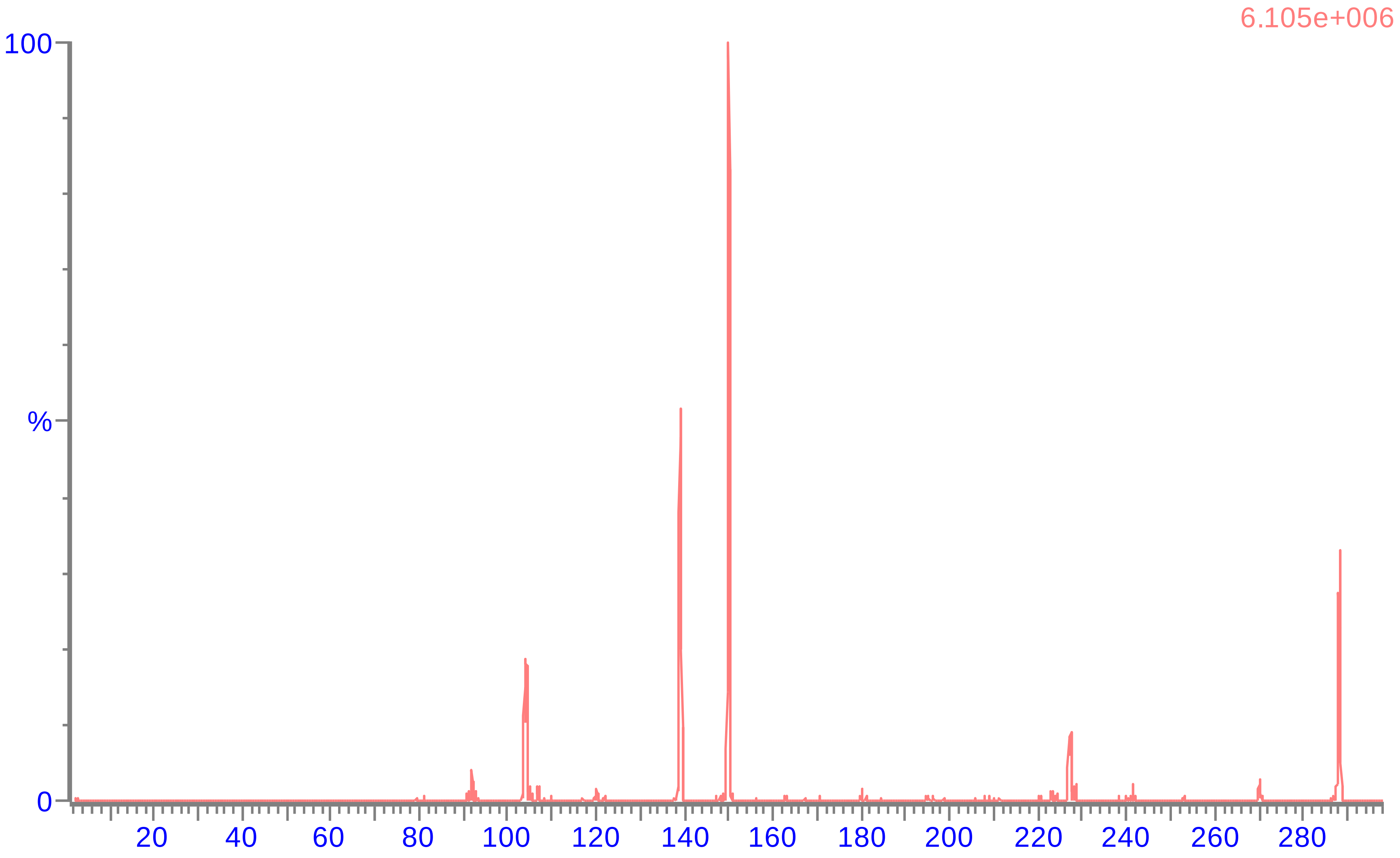

Figure 1.151: Mass spectrum for daughter fragment peak ES+, m/z 288.08 -> 150.05.

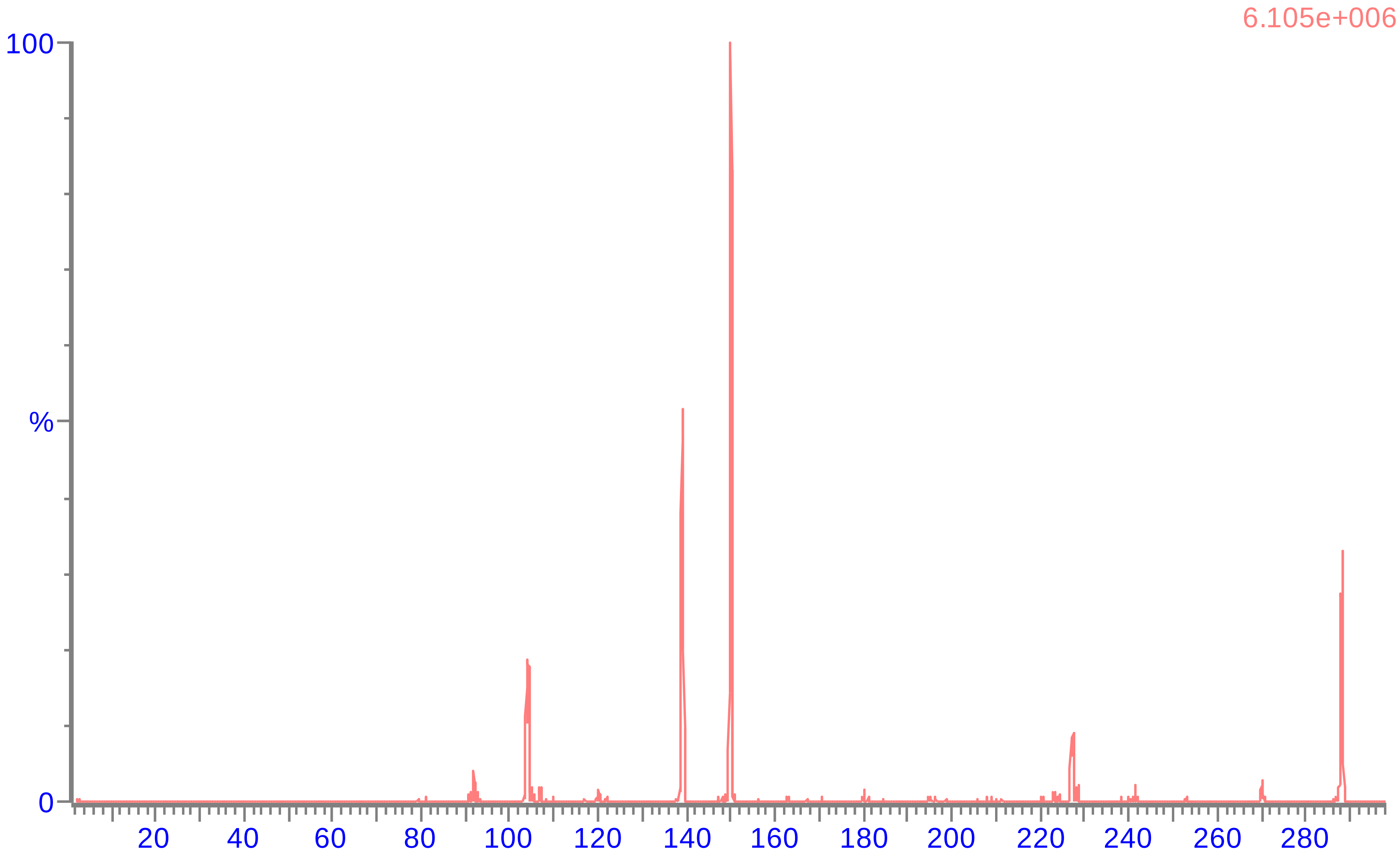

Figure 1.152: Mass spectrum for daughter fragment peak ES+, m/z 288.08 -> 138.99.

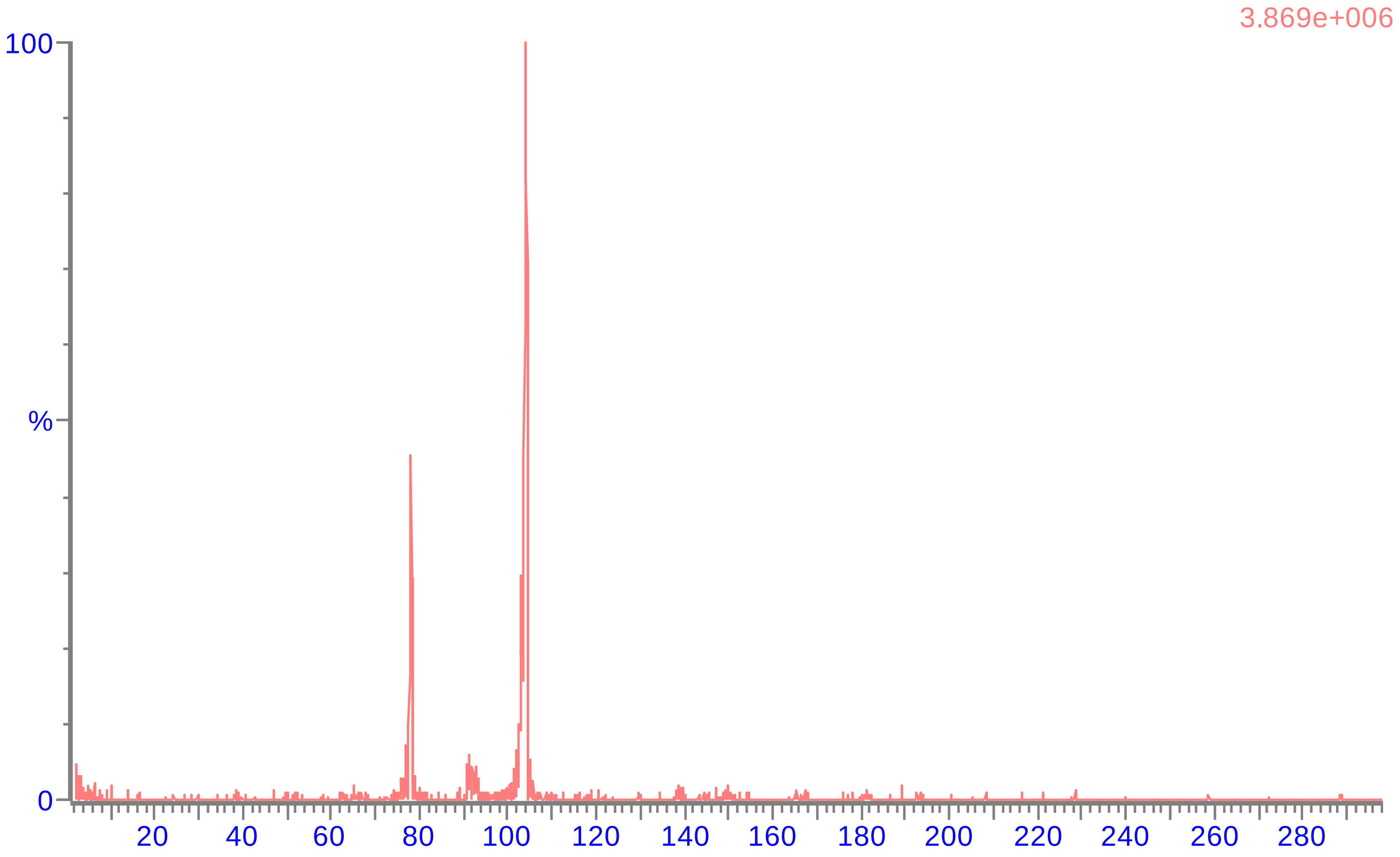

Figure 1.153: Mass spectrum for daughter fragment peak ES+, m/z 288.08 -> 77.99.

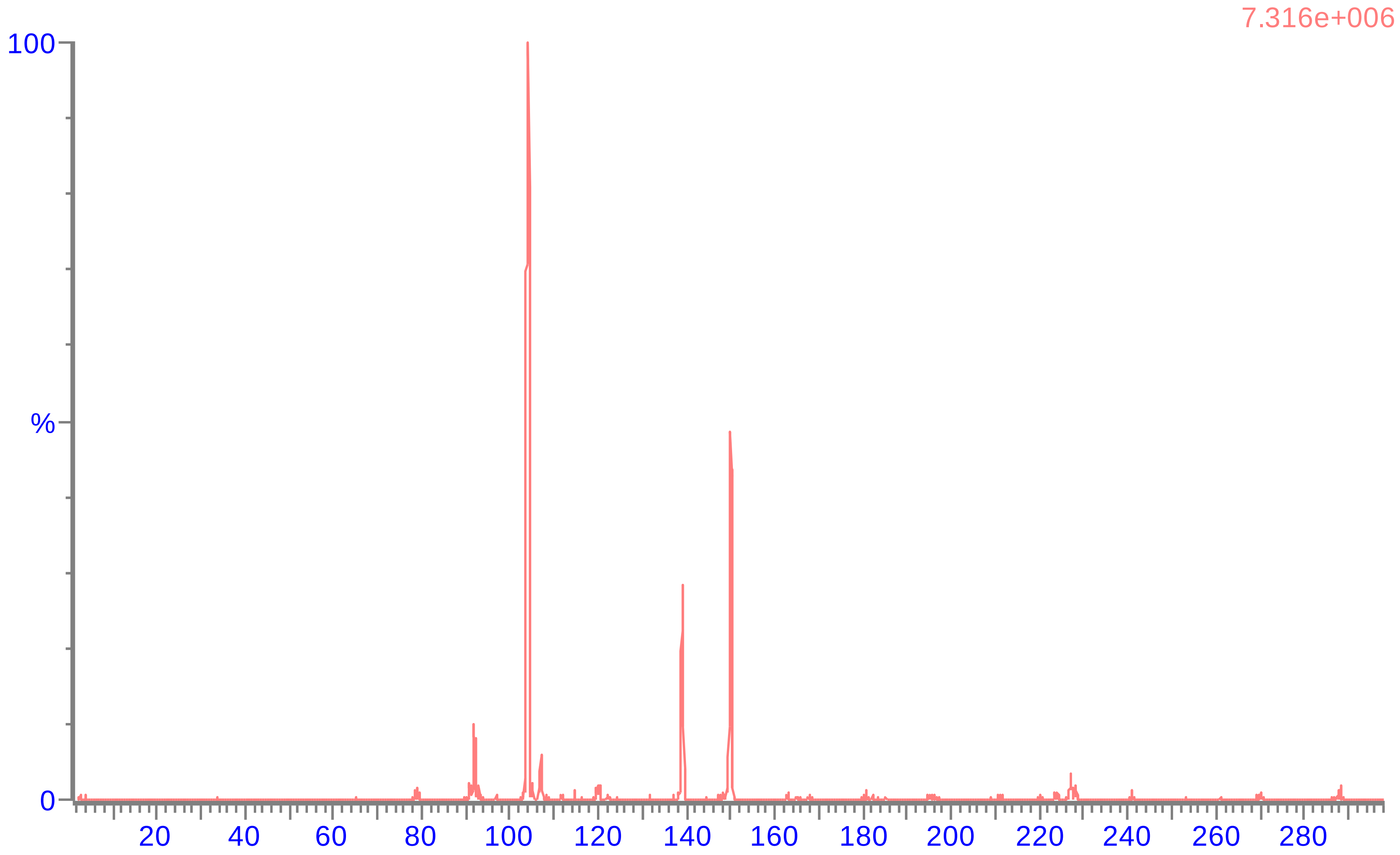

Figure 1.154: Mass spectrum for daughter fragment peak ES+, m/z 288.08 -> 91.97.

8k 2,5-dichloro-*N*-(2-nitro-1-phenylethyl)aniline

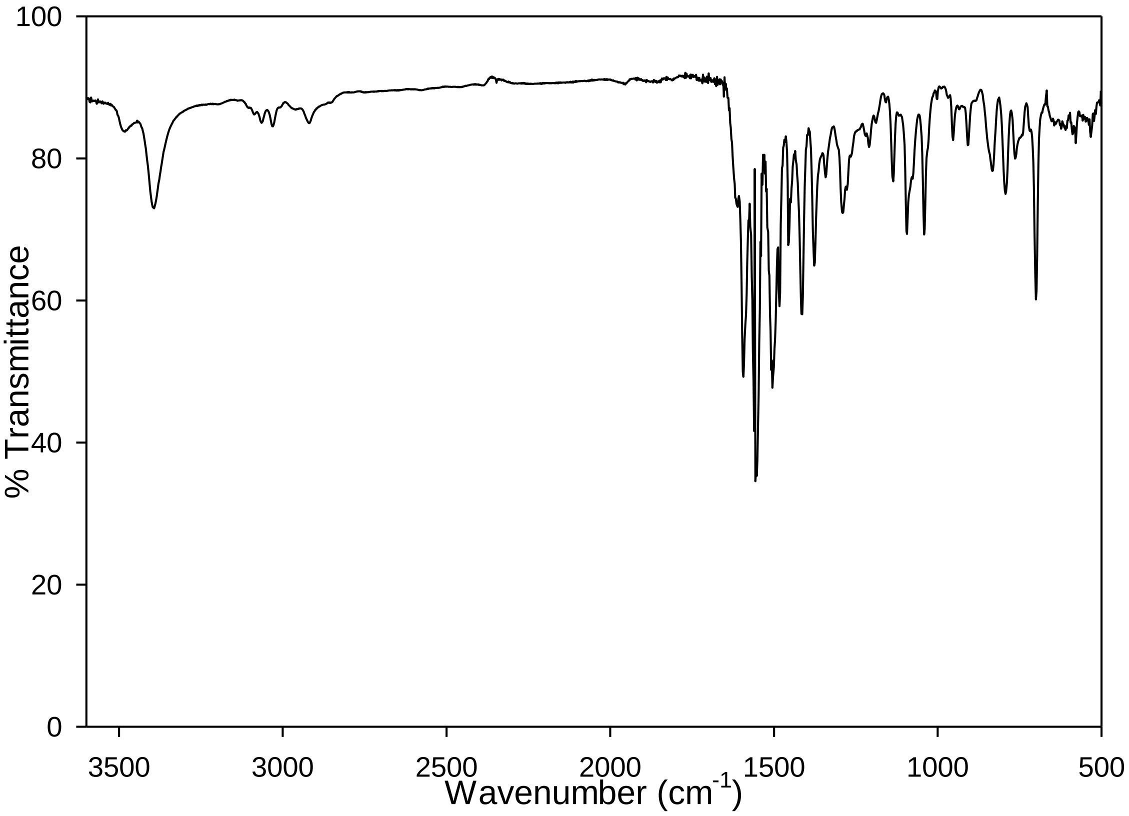

Figure 1.155: IR spectrum of **8k** 2,5-dichloro-*N*-(2-nitro-1-phenylethyl)aniline.

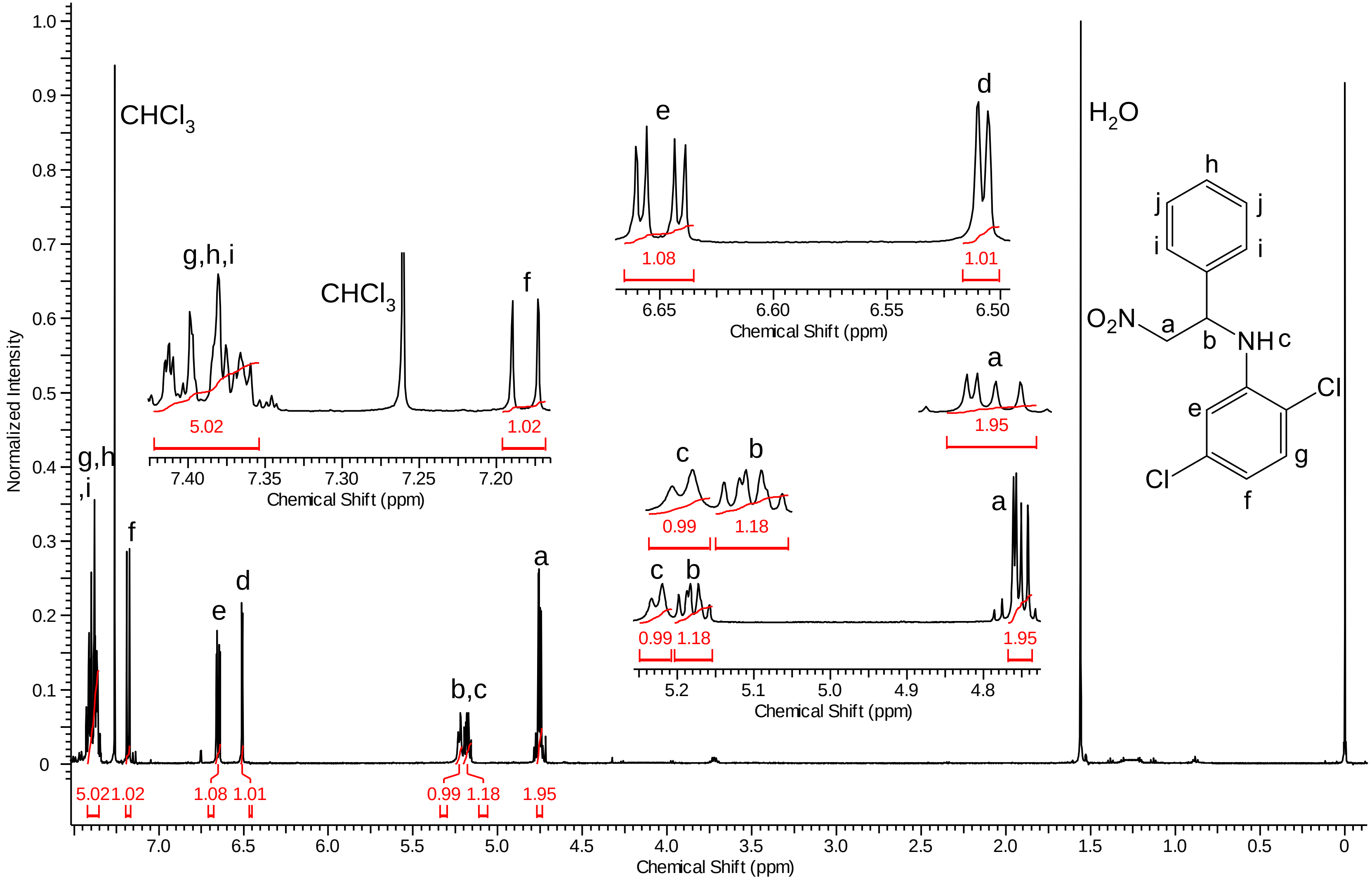

Figure 1.156: ^{1}H NMR spectrum of **8k** 2,5-dichloro-*N*-(2-nitro-1-phenylethyl)aniline.

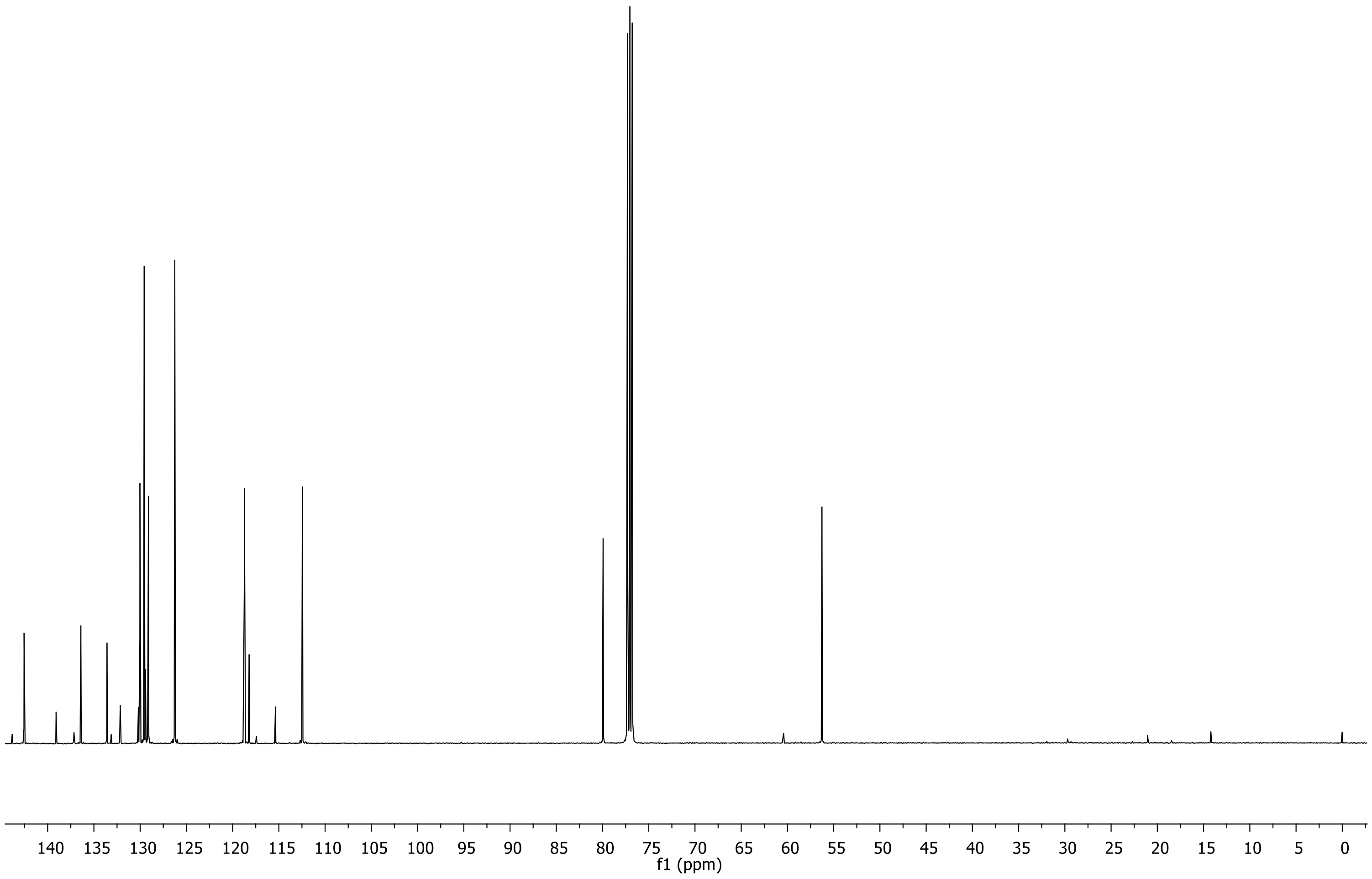

Figure 1.157: ^{13}C NMR spectrum of **8k** 2,5-dichloro-*N*-(2-nitro-1-phenylethyl)aniline.

Table 1.22: MS data.

Compound	Formula/Mass		Parent m/z	Cone Voltage	Daughters	Collision Energy	Ion Mode
**8k**	312	1	312.97	16	93.98	12	ES+
		2	312.97	18	217.96	8	ES-
		3	312.97	16	252.02	12	ES+
		4	312.97	16	173.91	26	ES+
		5	312.97	16	101.73	58	ES+

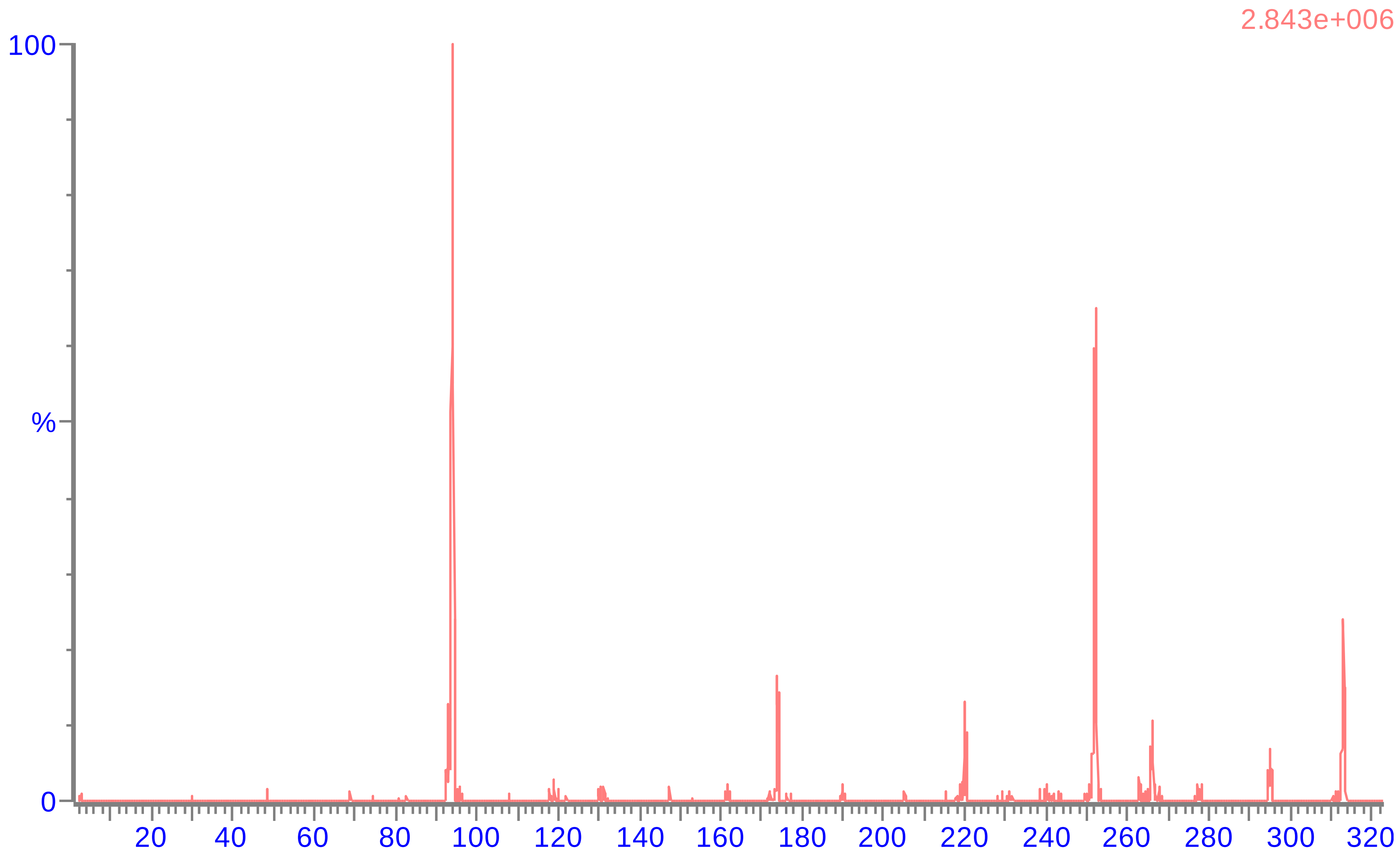

Figure 1.158: Mass spectrum for daughter fragment peak ES+, m/z 312.97 -> 93.98.

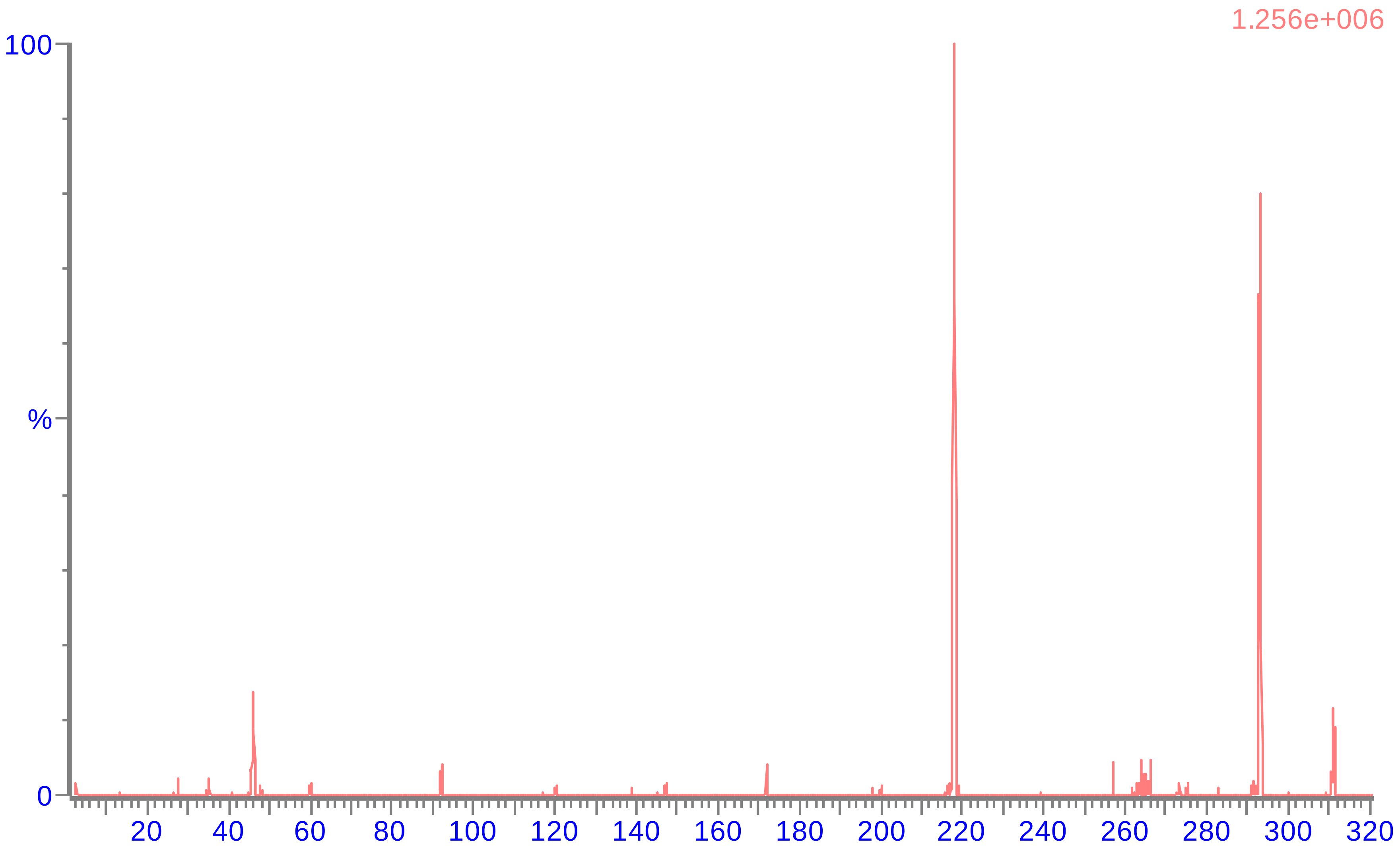

Figure 1.159: Mass spectrum for daughter fragment peak ES+, m/z 312.97 -> 217.96.

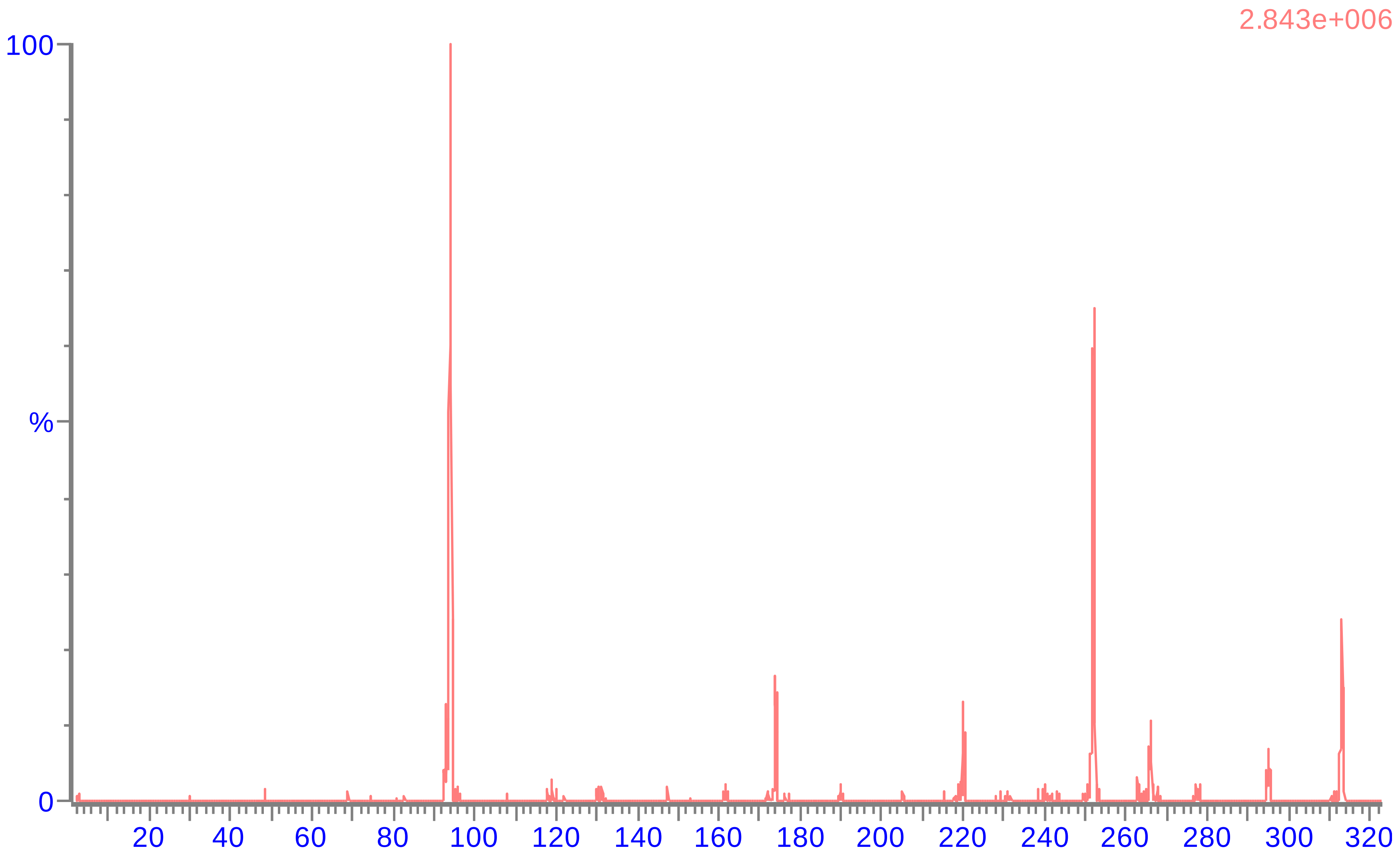

Figure 1.160: Mass spectrum for daughter fragment peak ES+, m/z 312.97 -> 252.02.

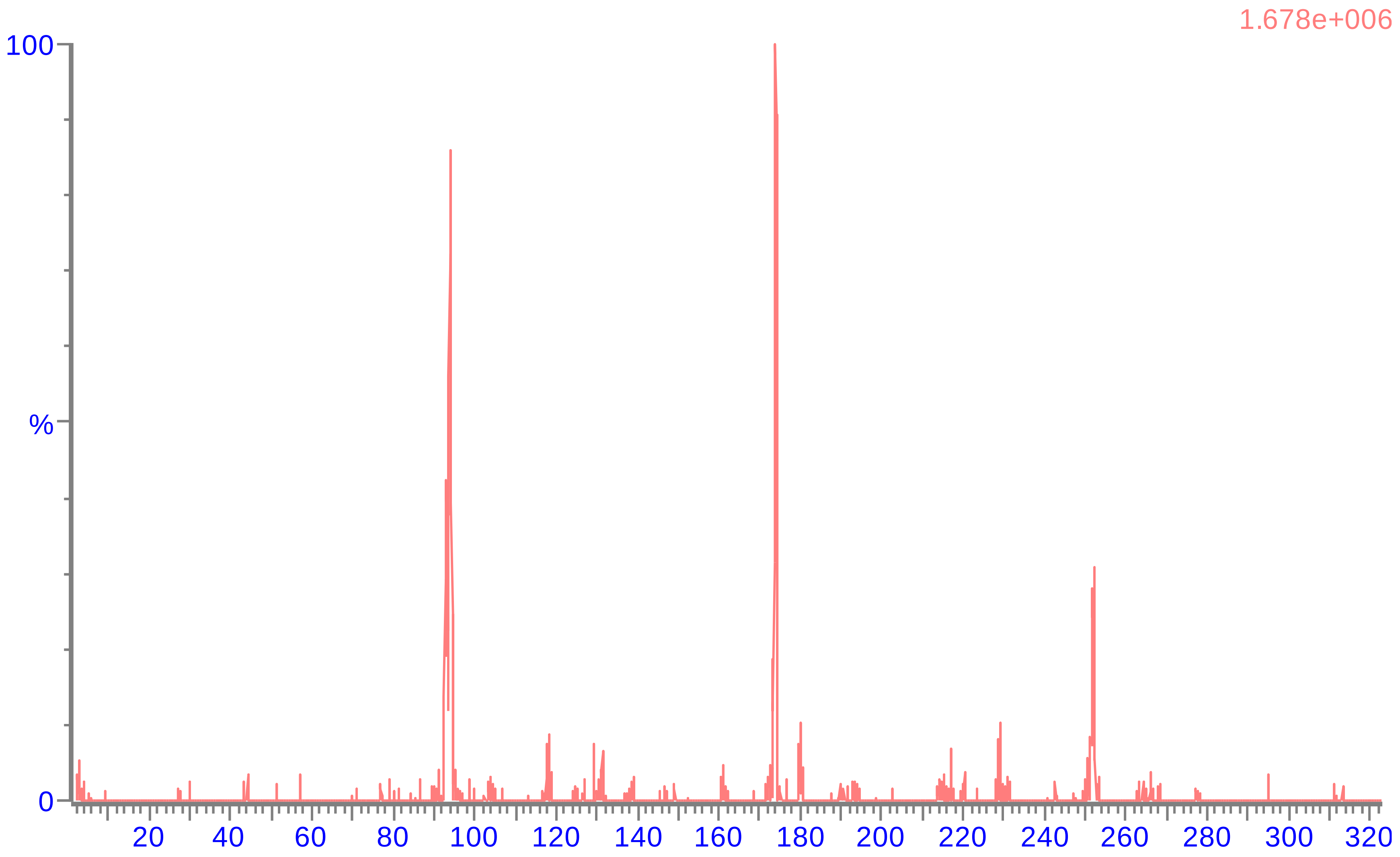

Figure 1.161: Mass spectrum for daughter fragment peak ES+, m/z 312.97 -> 173.91.

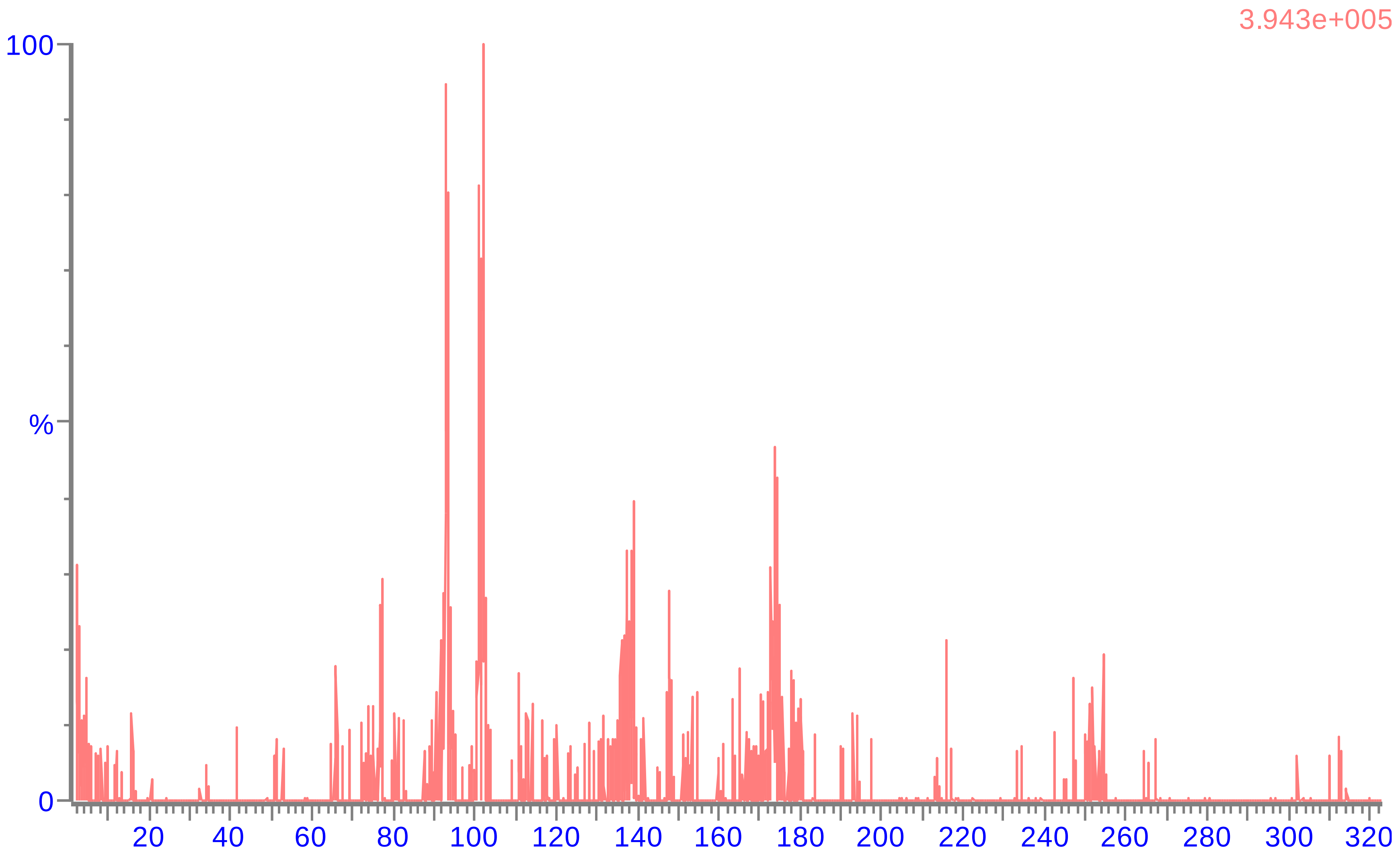

Figure 1.162: Mass spectrum for daughter fragment peak ES+, m/z 312.97 -> 101.73.

8l (*E*)-*N*-(2-nitro-1-phenylethyl)-4-(phenyldiazenyl)aniline

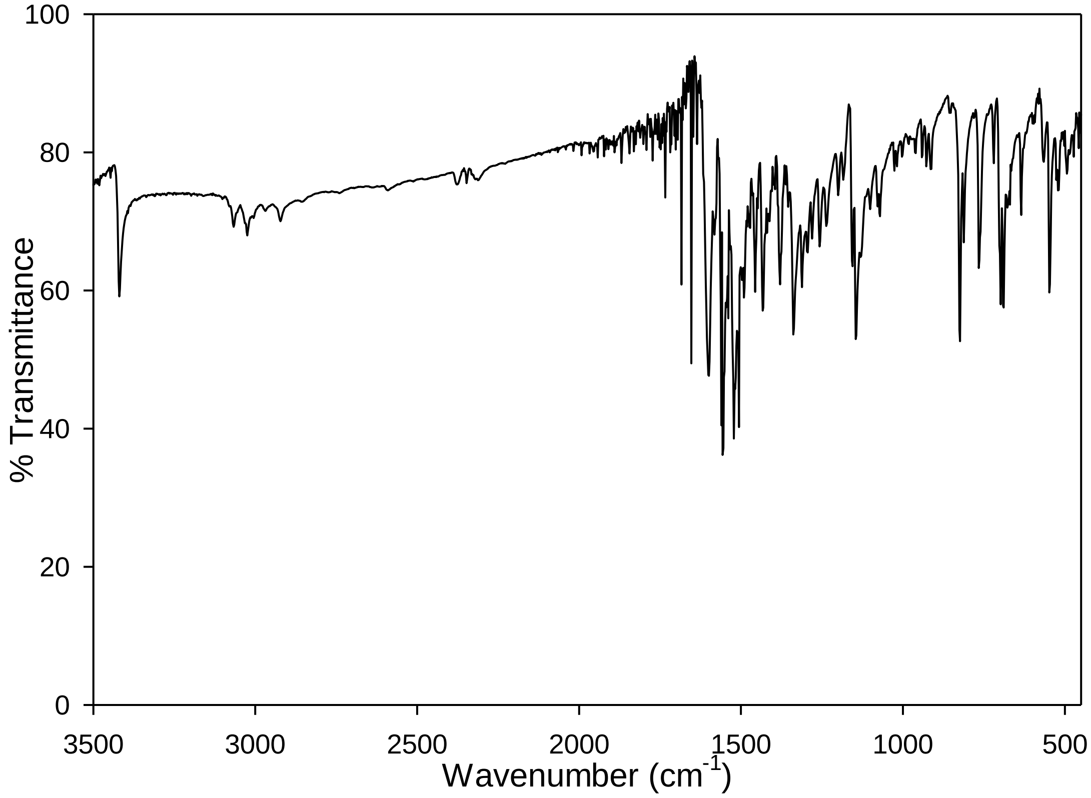

Figure 1.163: IR spectrum of **8l** (*E*)-*N*-(2-nitro-1-phenylethyl)-4-(phenyldiazenyl)aniline.

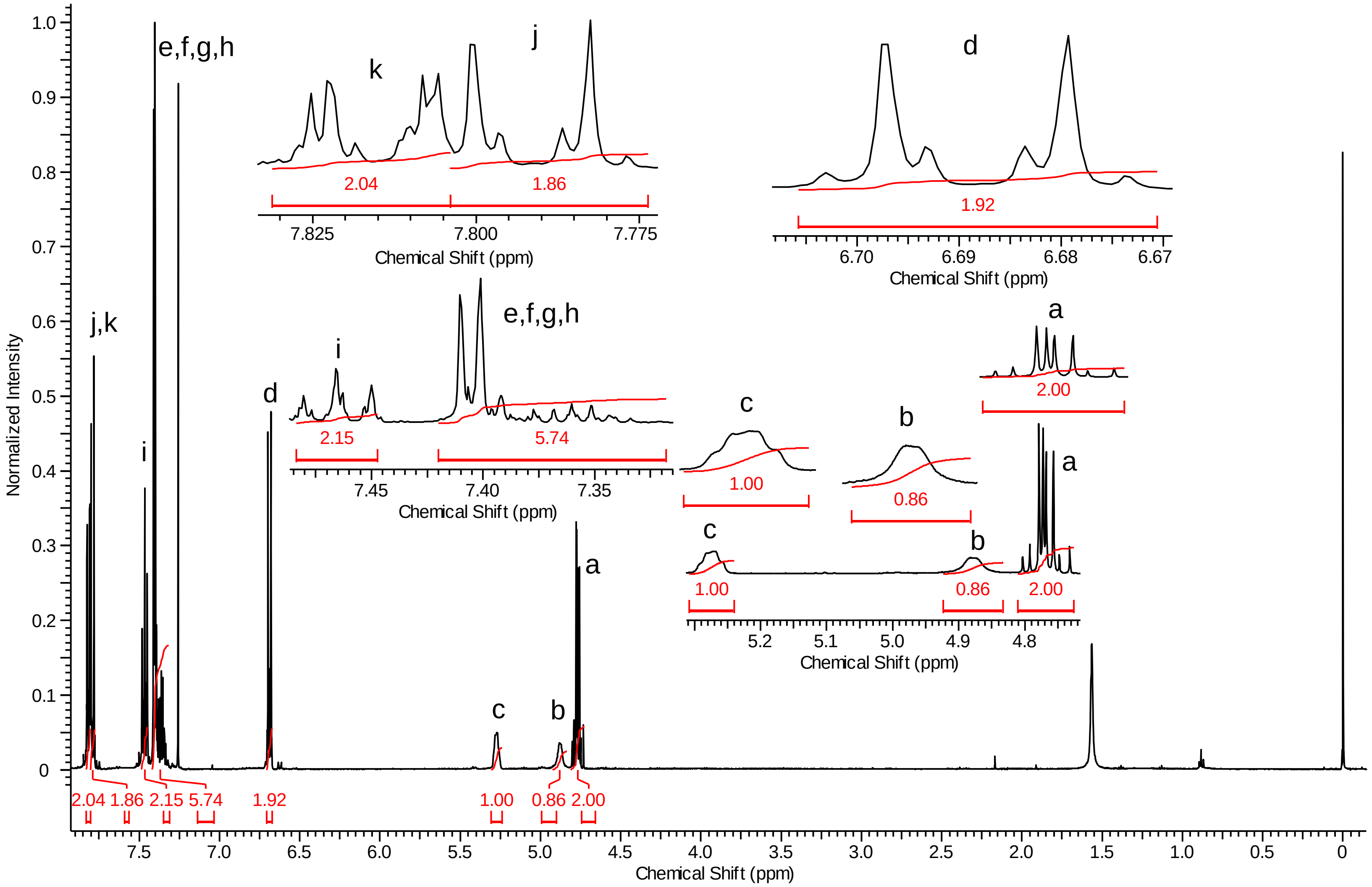

Figure 1.164: ^{1}H NMR spectrum of **8l** (*E*)-*N*-(2-nitro-1-phenylethyl)-4-(phenyldiazenyl)aniline.

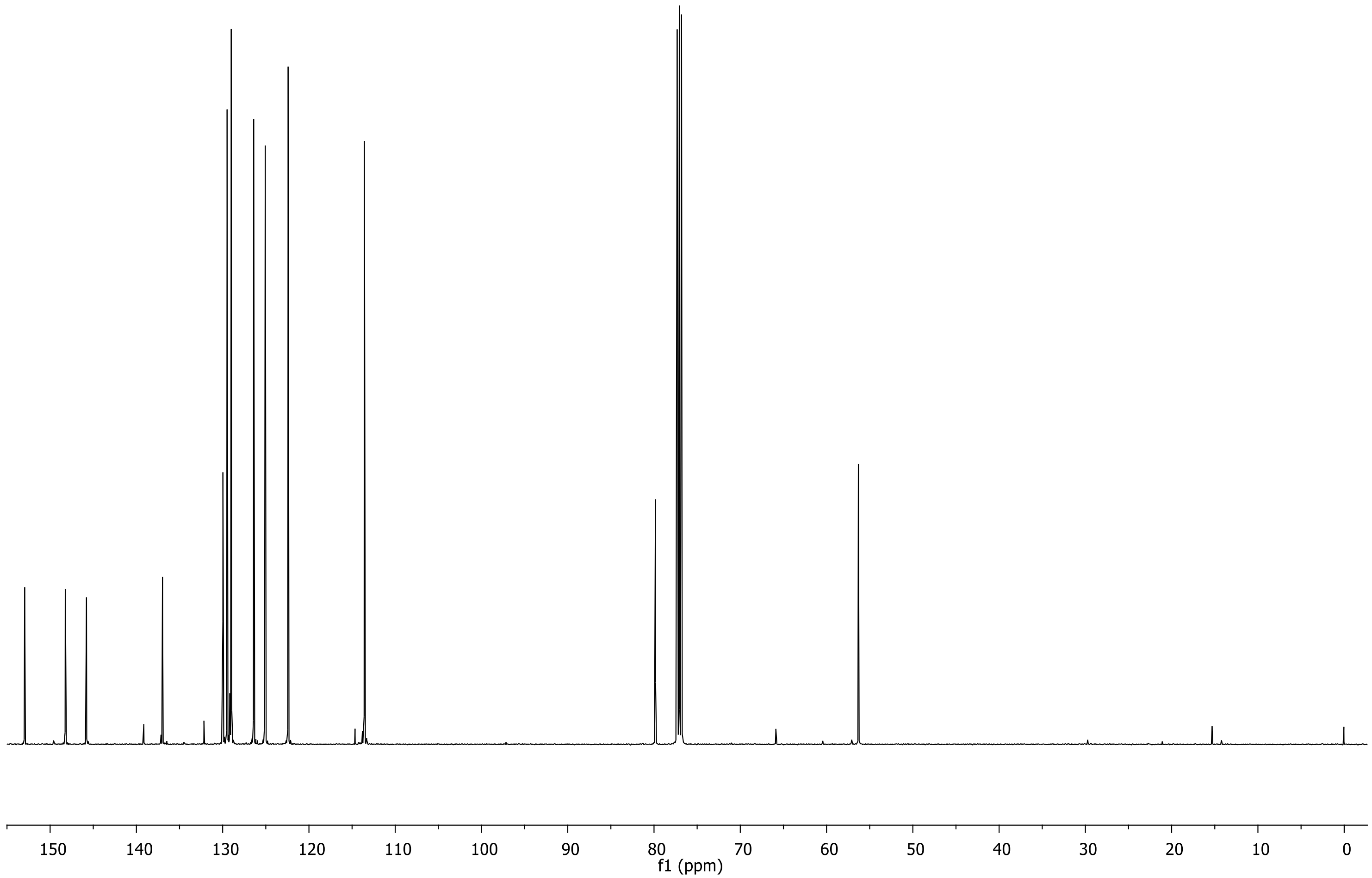

Figure 1.165: ^{13}C NMR spectrum of **8l** (*E*)-*N*-(2-nitro-1-phenylethyl)-4-(phenyldiazenyl)aniline.

Table 1.23: MS data

Compound	Formula/Mass		Parent m/z	Cone Voltage	Daughters	Collision Energy	Ion Mode
**8l**	346	1	347.16	40	197.07	26	ES+
		2	347.16	40	77.07	44	ES+
		3	347.16	40	91.97	54	ES+
		4	347.16	40	65.04	64	ES+
		5	347.16	40	104.13	32	ES+

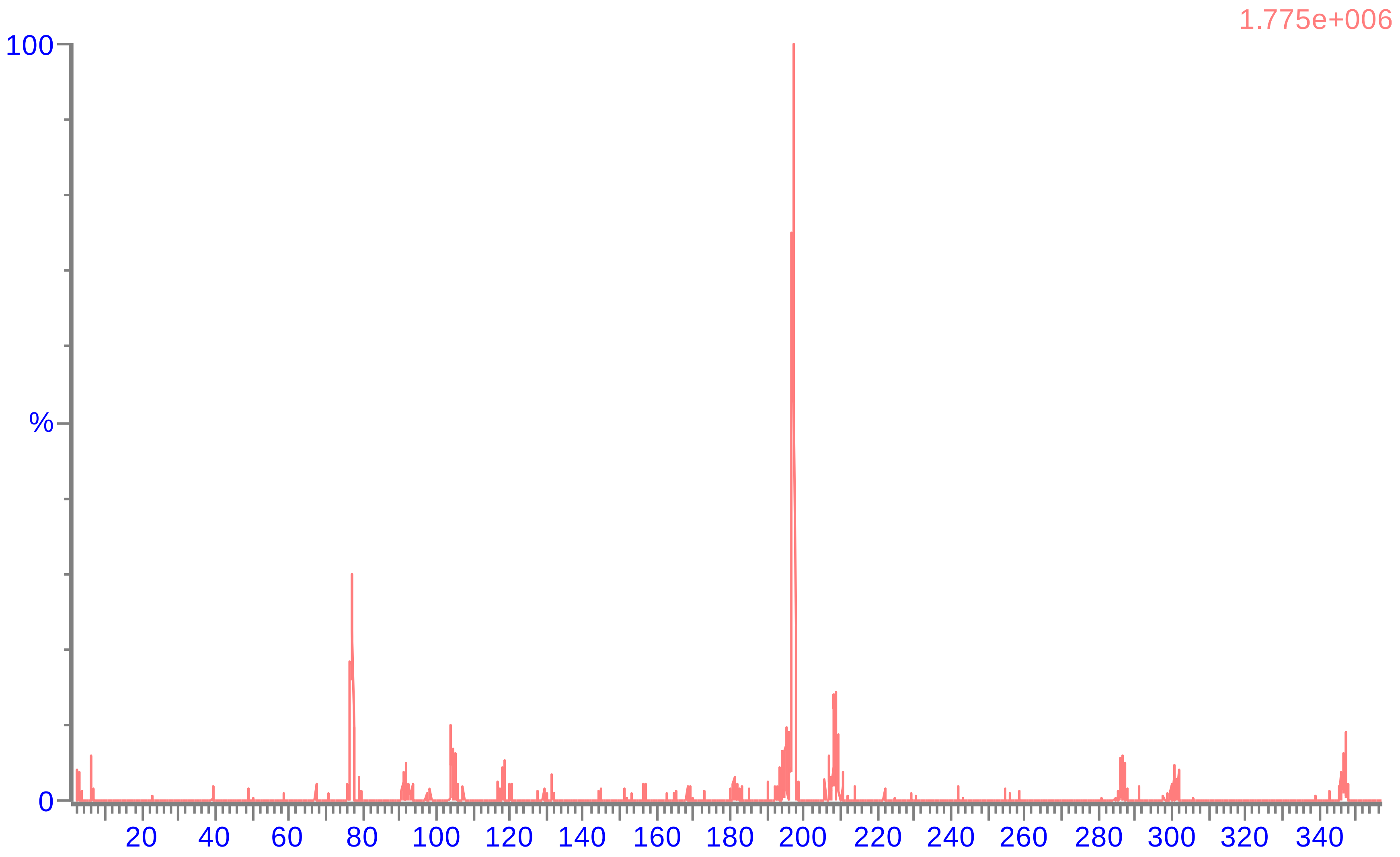

Figure 1.166: Mass spectrum for daughter fragment peak ES+, m/z 347.16 -> 197.07.

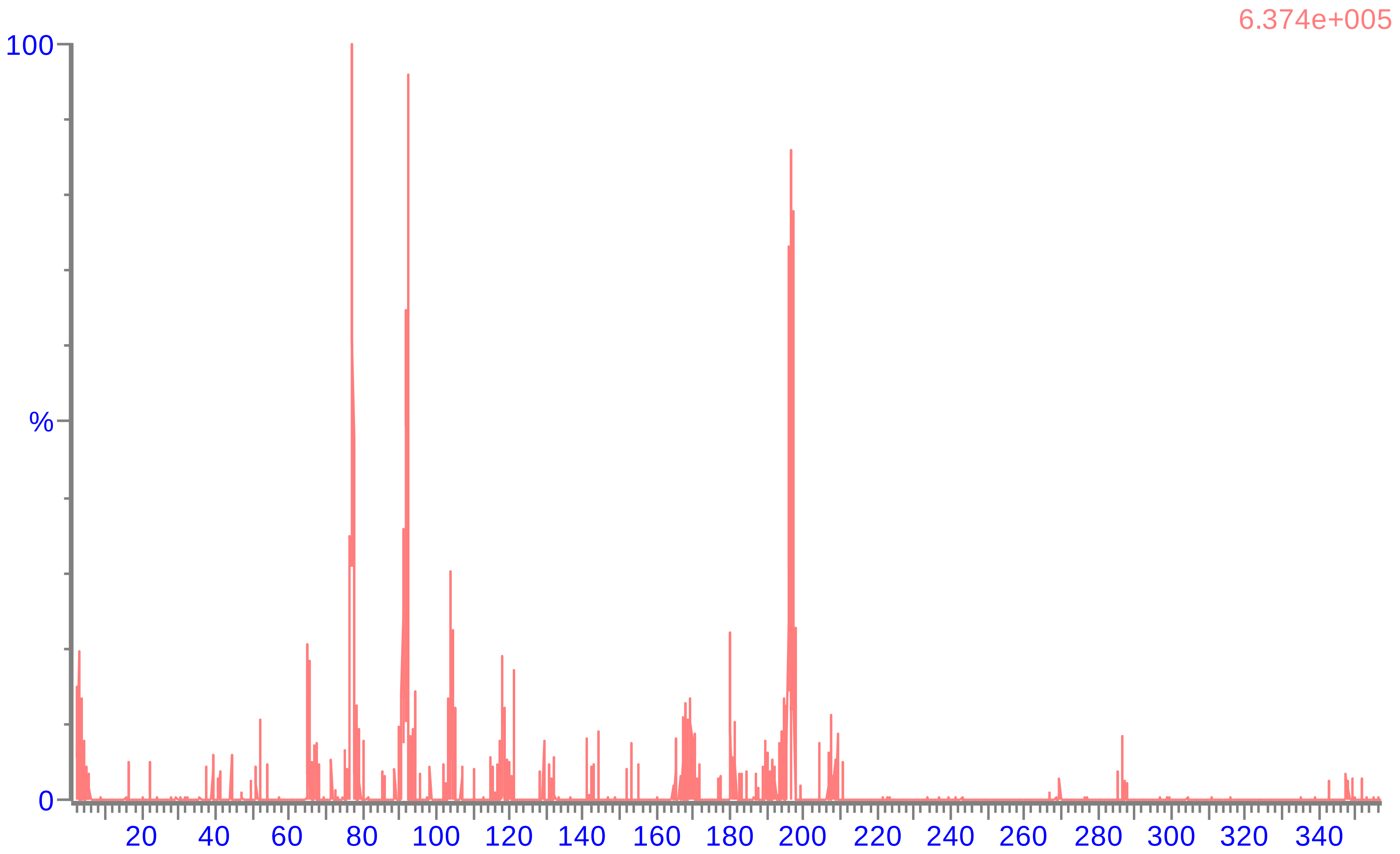

Figure 1.167: Mass spectrum for daughter fragment peak ES+, m/z 347.16 -> 77.07.

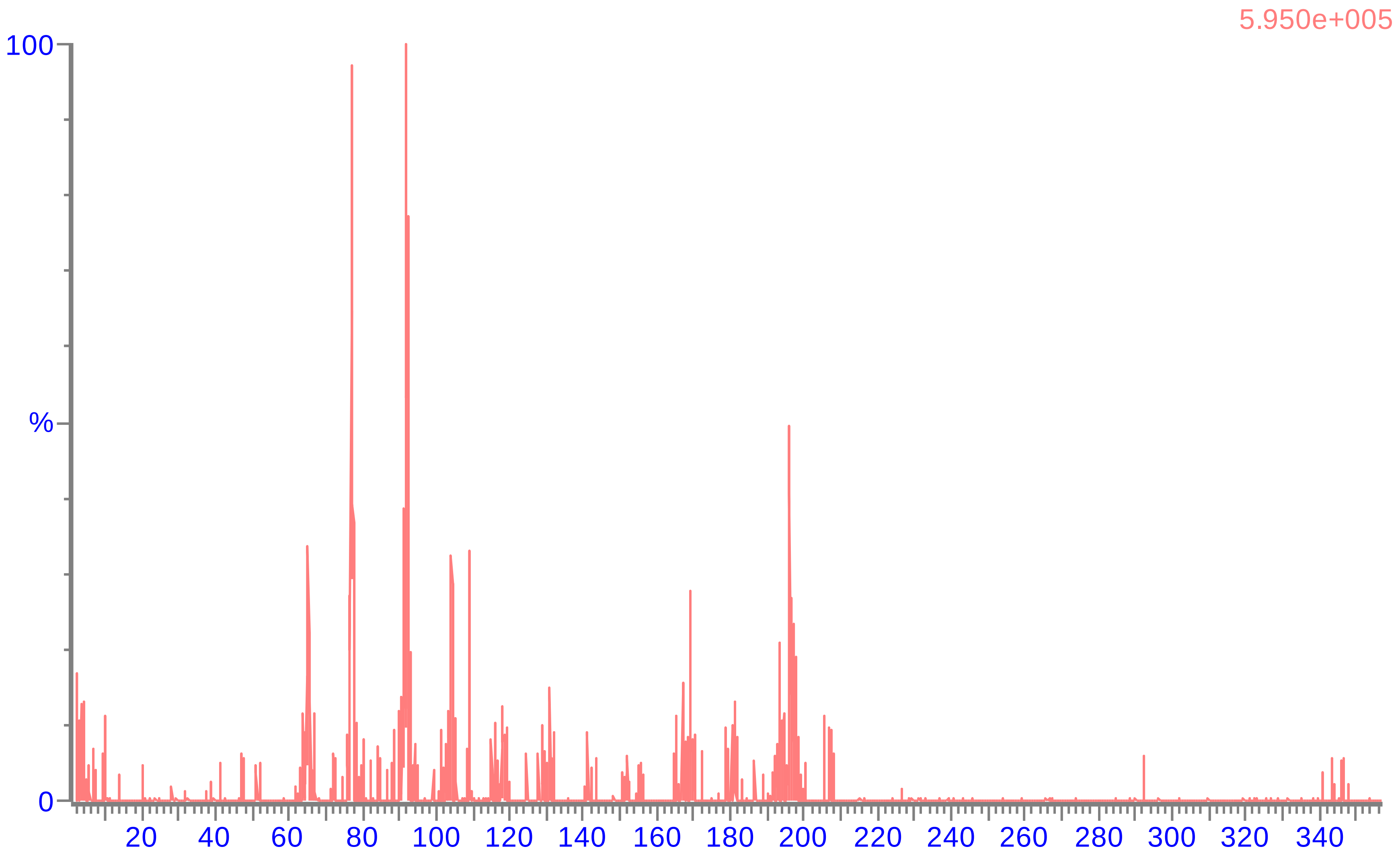

Figure 1.168: Mass spectrum for daughter fragment peak ES+, m/z 347.16 -> 91.97.

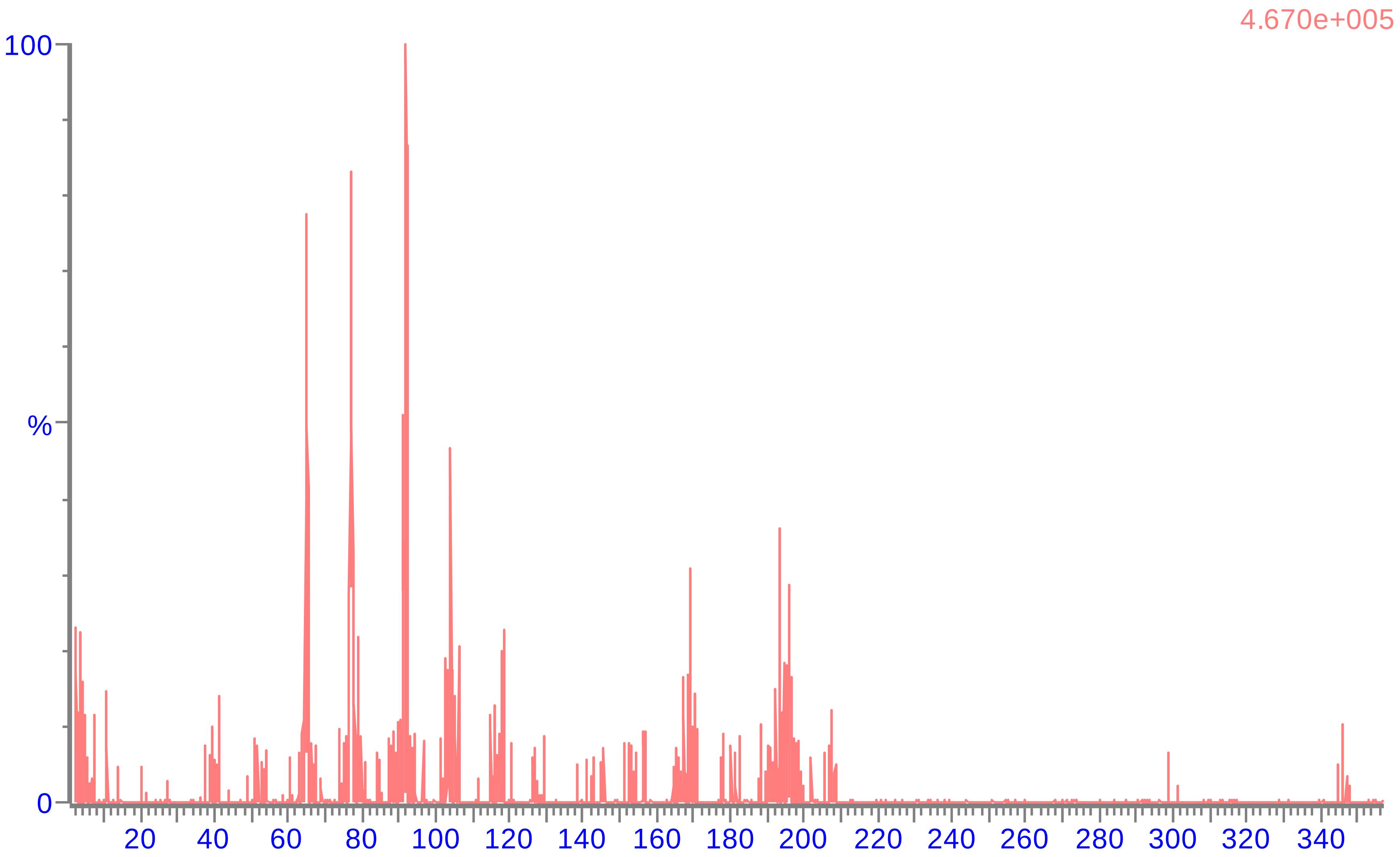

Figure 1.169: Mass spectrum for daughter fragment peak ES+, m/z 347.16 -> 65.04.

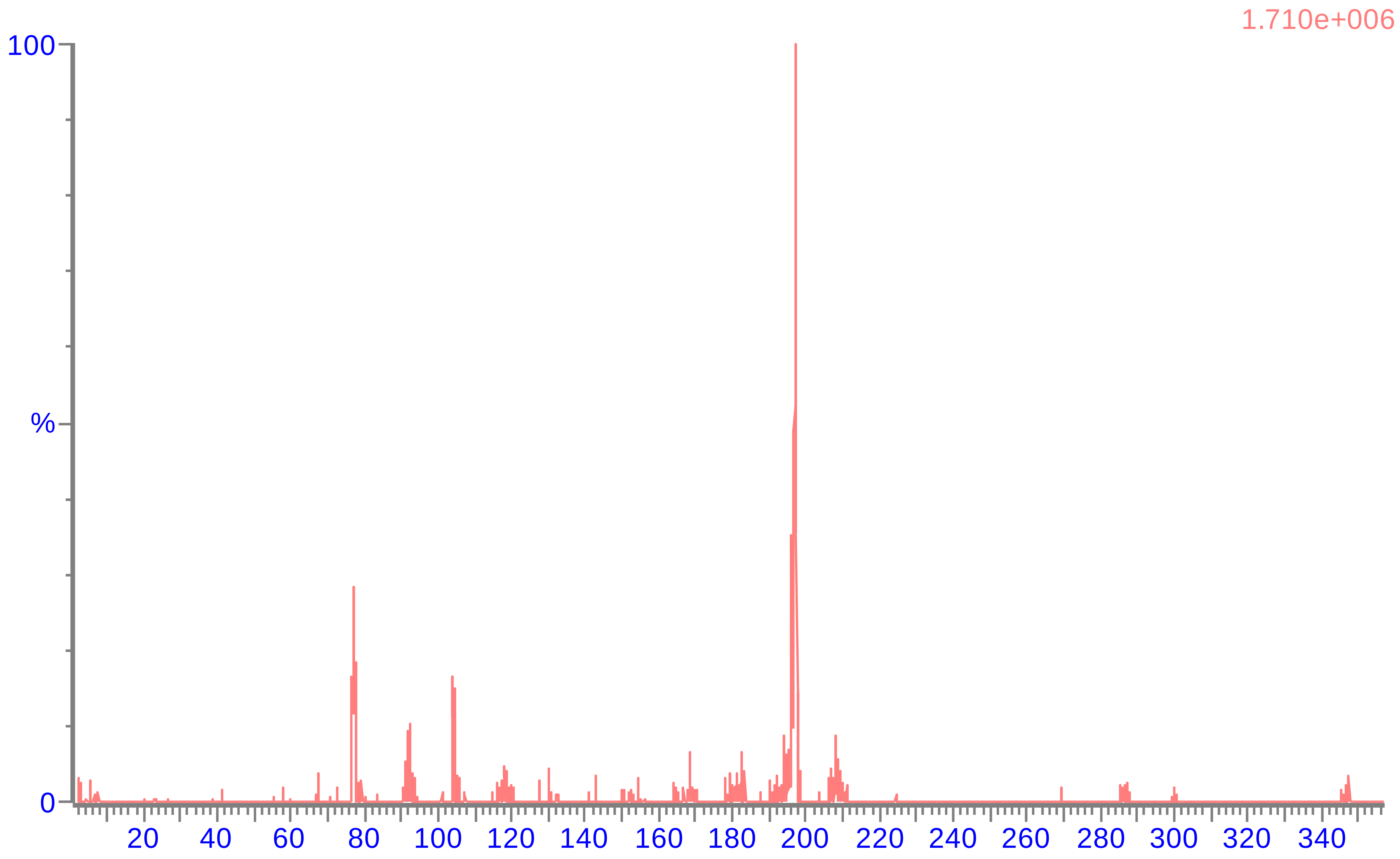

Figure 1.170: Mass spectrum for daughter fragment peak ES+, m/z 347.16 -> 104.13.

9a *N*-(2-nitro-1-phenylpropyl)aniline.

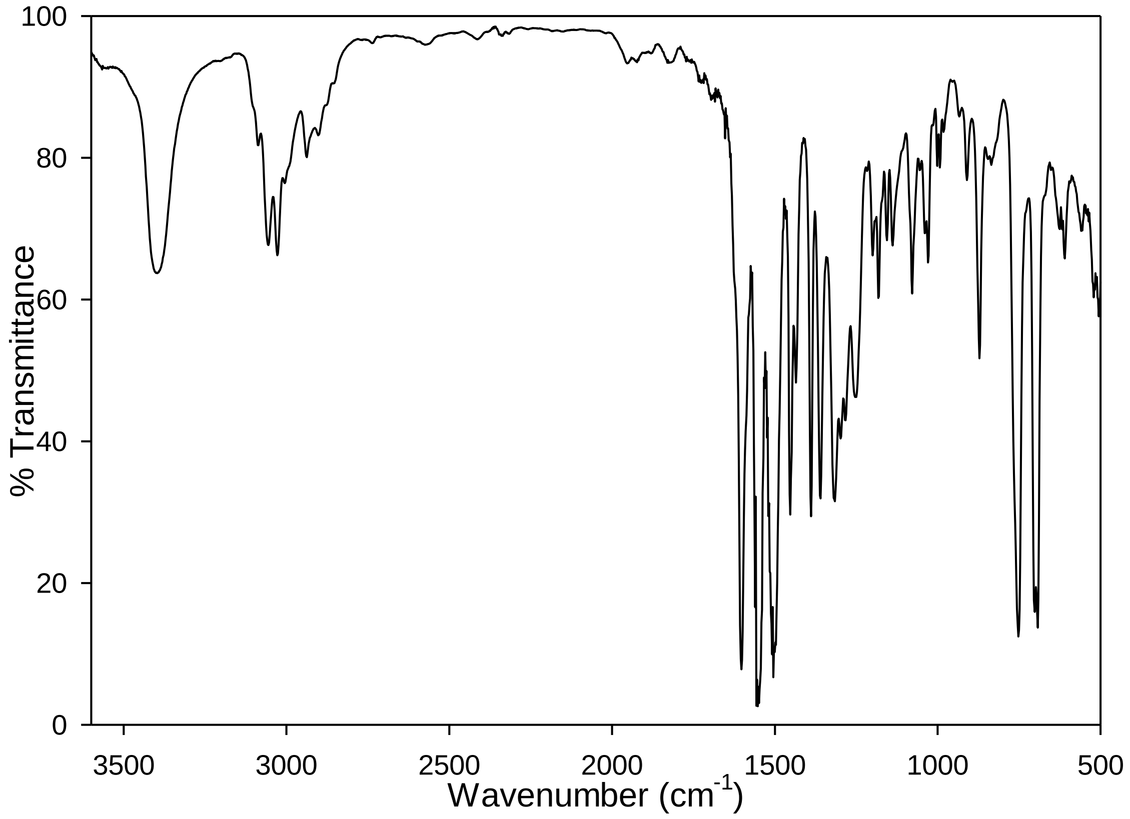

Figure 1.171: IR spectrum of **9a** *N*-(2-nitro-1-phenylpropyl)aniline.

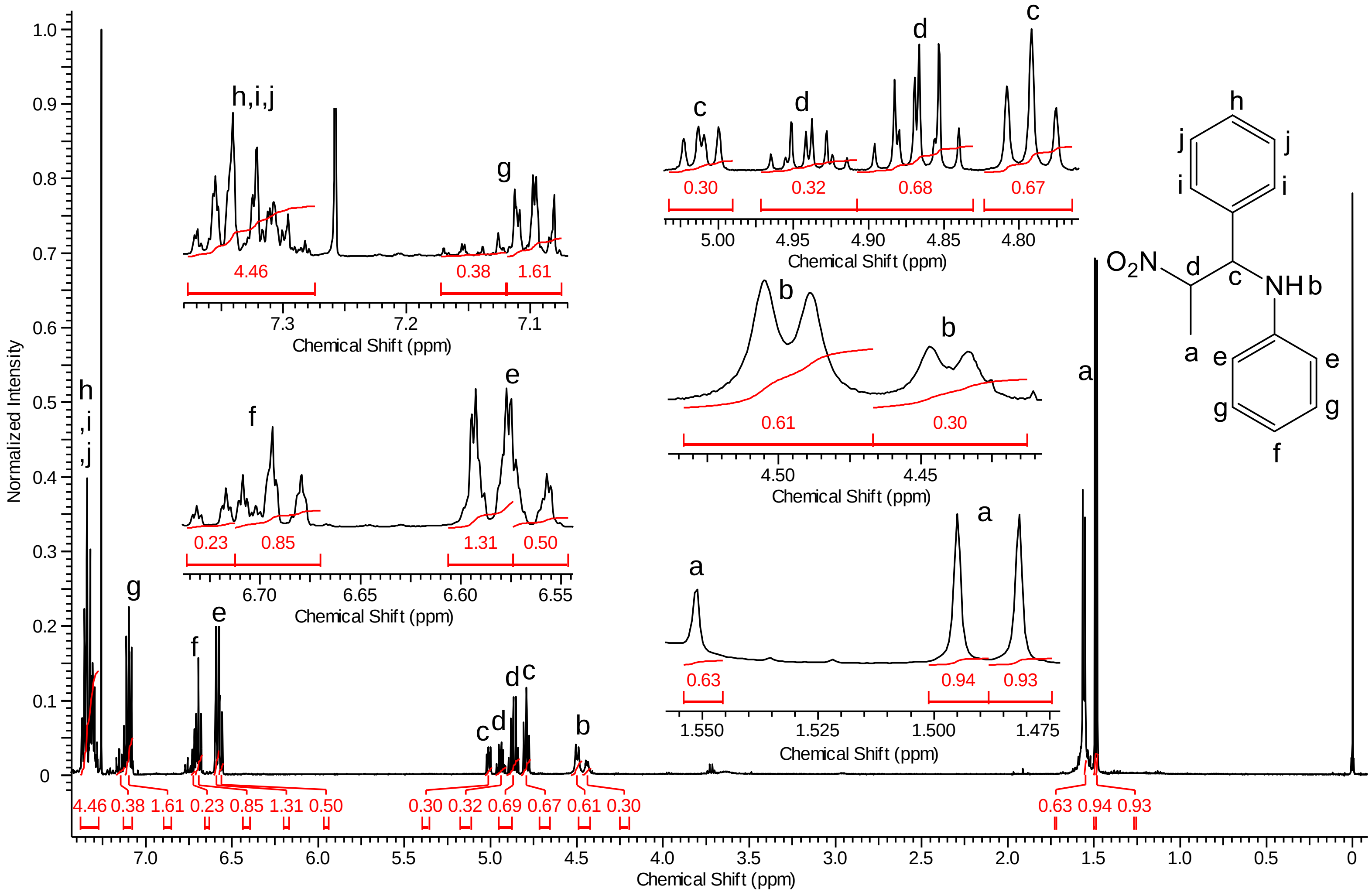

Figure 1.172: ^{1}H NMR spectrum of **9a** *N*-(2-nitro-1-phenylpropyl)aniline (two pairs of diastereomers).

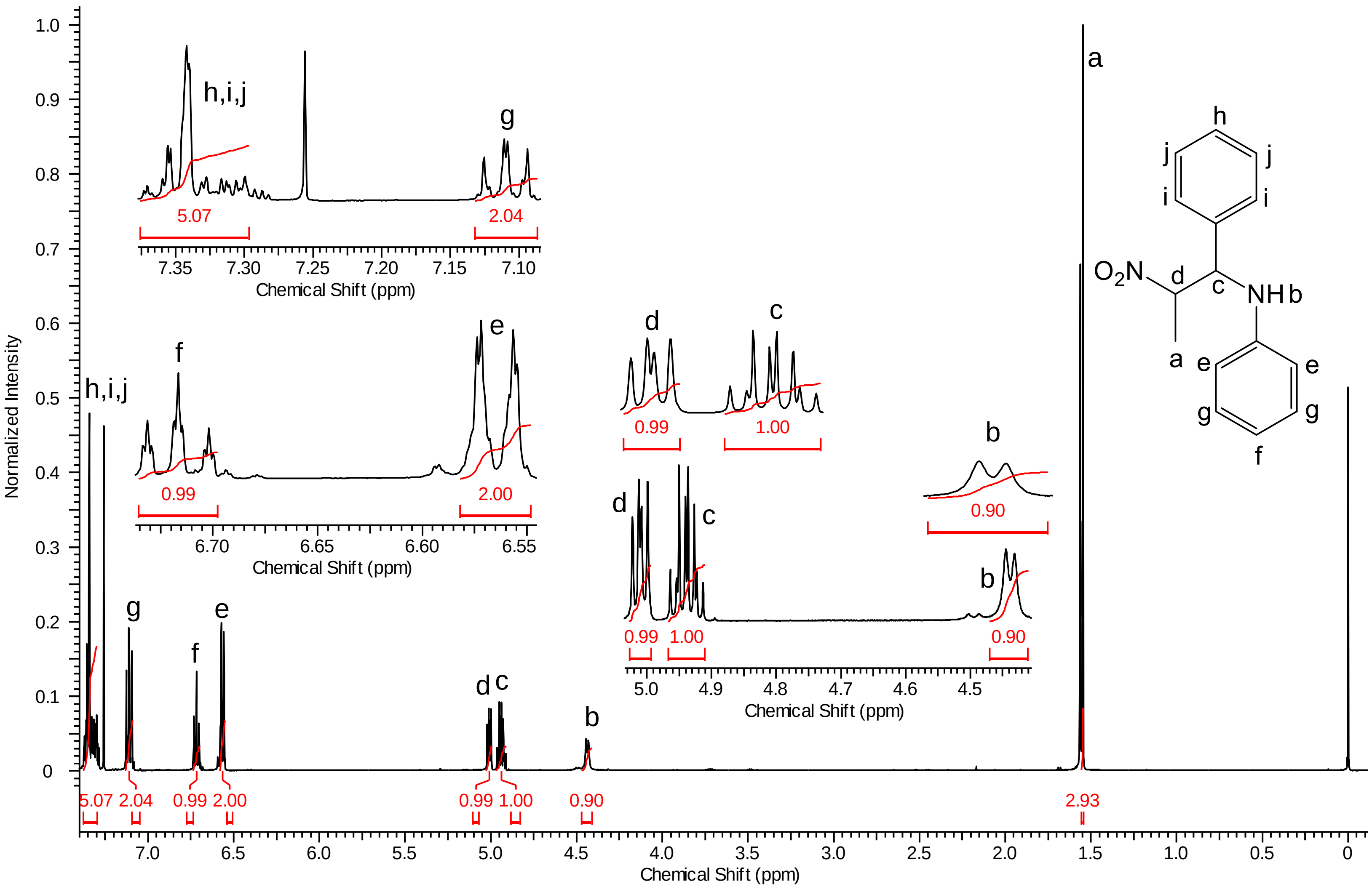

Figure 1.173: ^{1}H NMR spectrum of **9a** *N*-(2-nitro-1-phenylpropyl)aniline (one pair of diastereomers).

9b *N*-(2-nitro-1-phenylbutyl)aniline

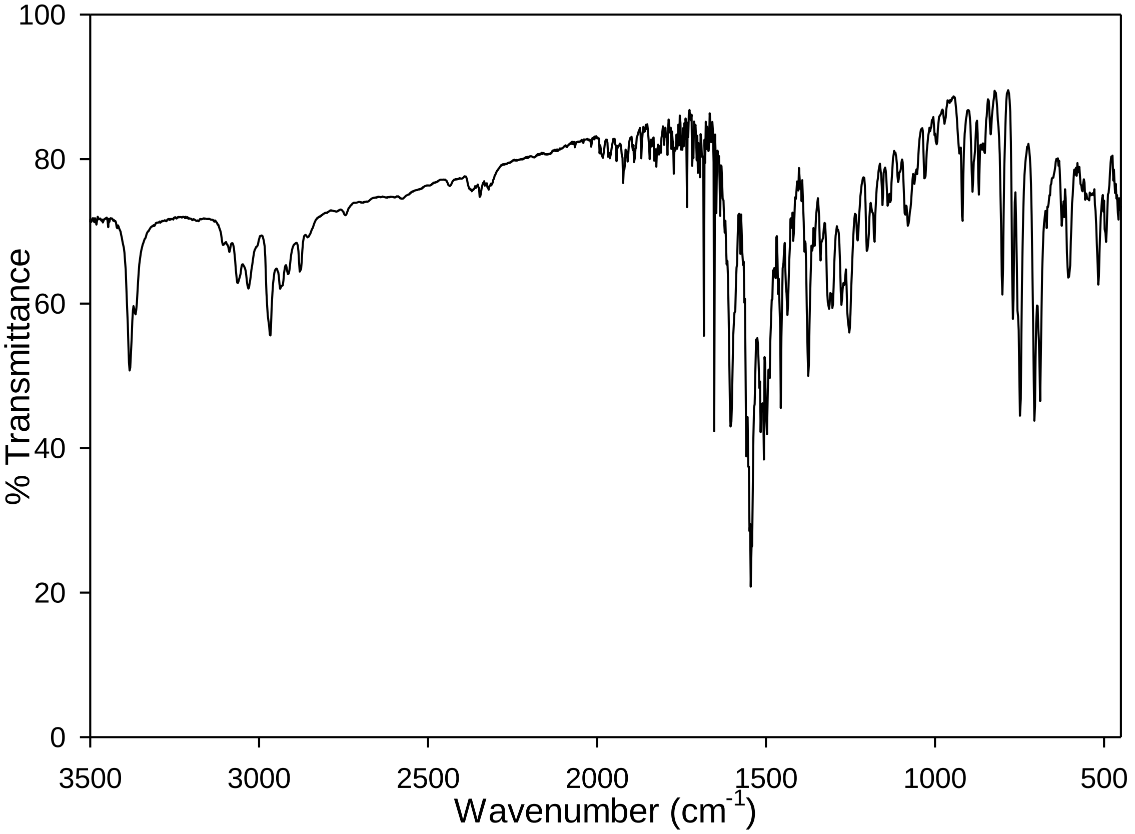

Figure 1.174: IR spectrum of **9b** *N*-(2-nitro-1-phenylbutyl)aniline.

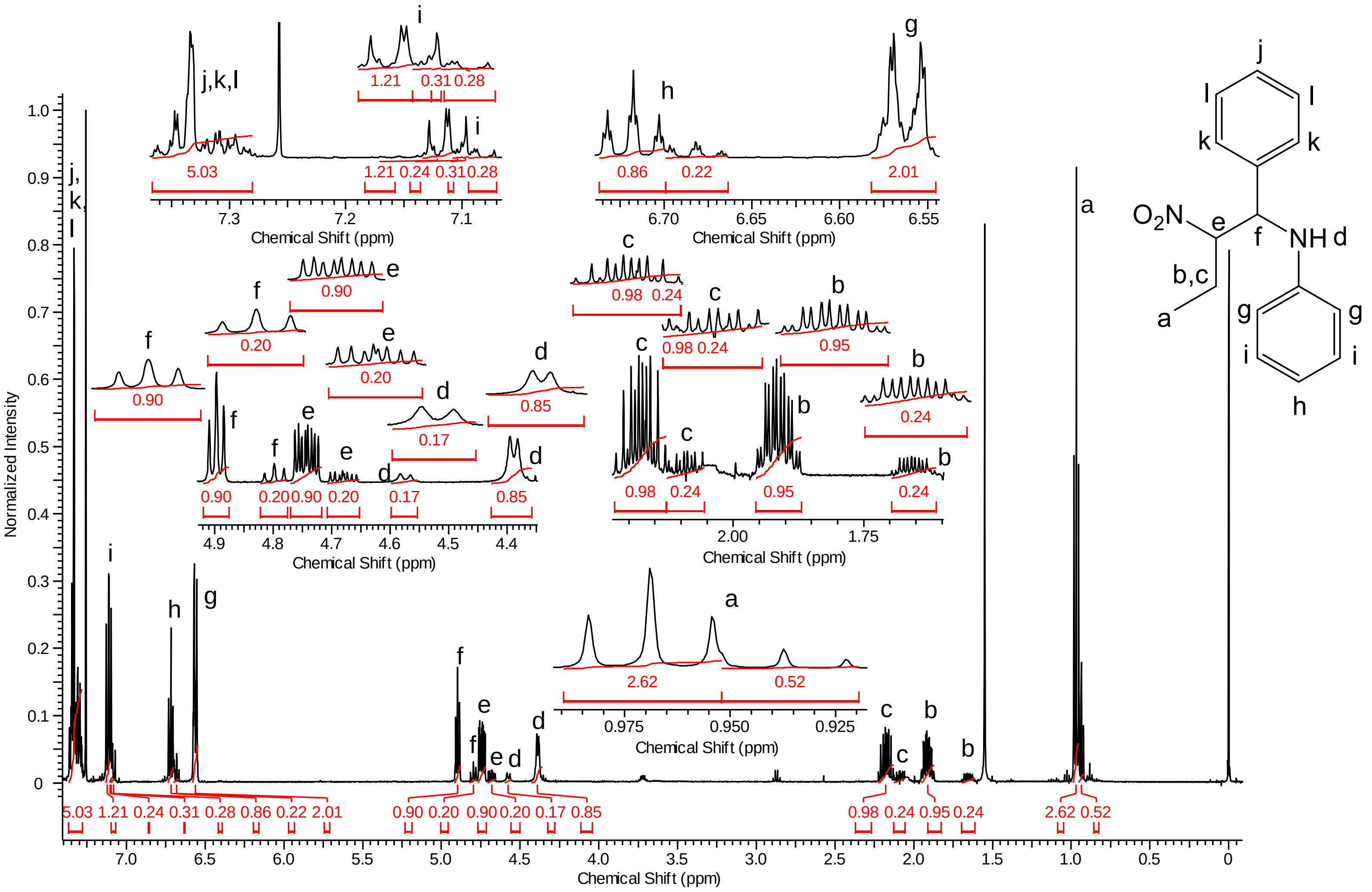

Figure 1.175: ^{1}H NMR spectrum of **9b** *N*-(2-nitro-1-phenylbutyl)aniline (two pairs of diastereomers).

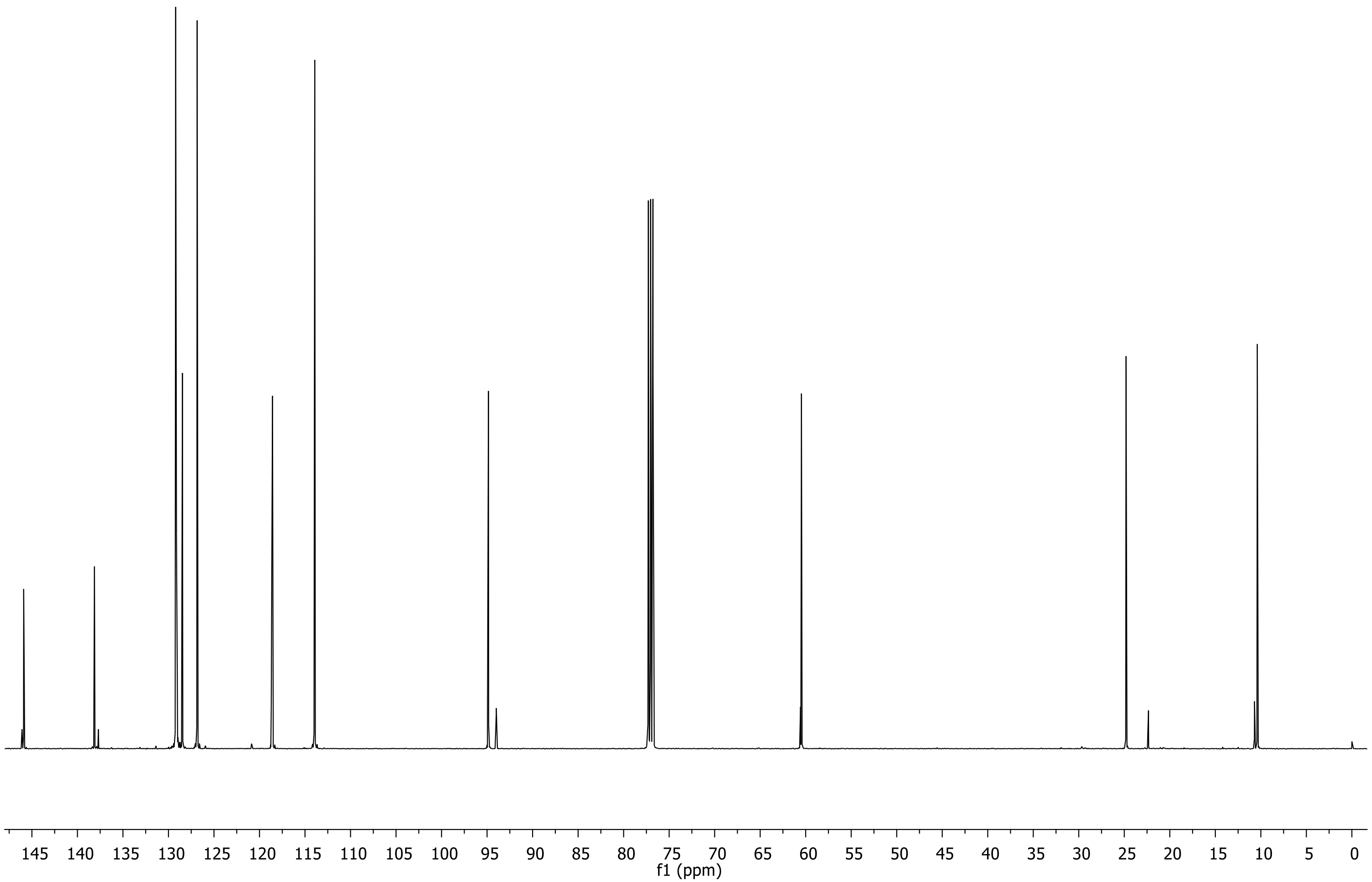

Figure 1.176: ^{13}C NMR spectrum of **9b** *N*-(2-nitro-1-phenylbutyl)aniline (two pairs of diastereomers).

Table 1.24: MS data.

Compound	Formula/Mass		Parent m/z	Cone Voltage	Daughters	Collision Energy	Ion Mode
**9b**	270	1	271.16	24	148.03	16	ES+
		2	271.16	24	77.07	56	ES+
		3	271.16	24	56.19	22	ES+

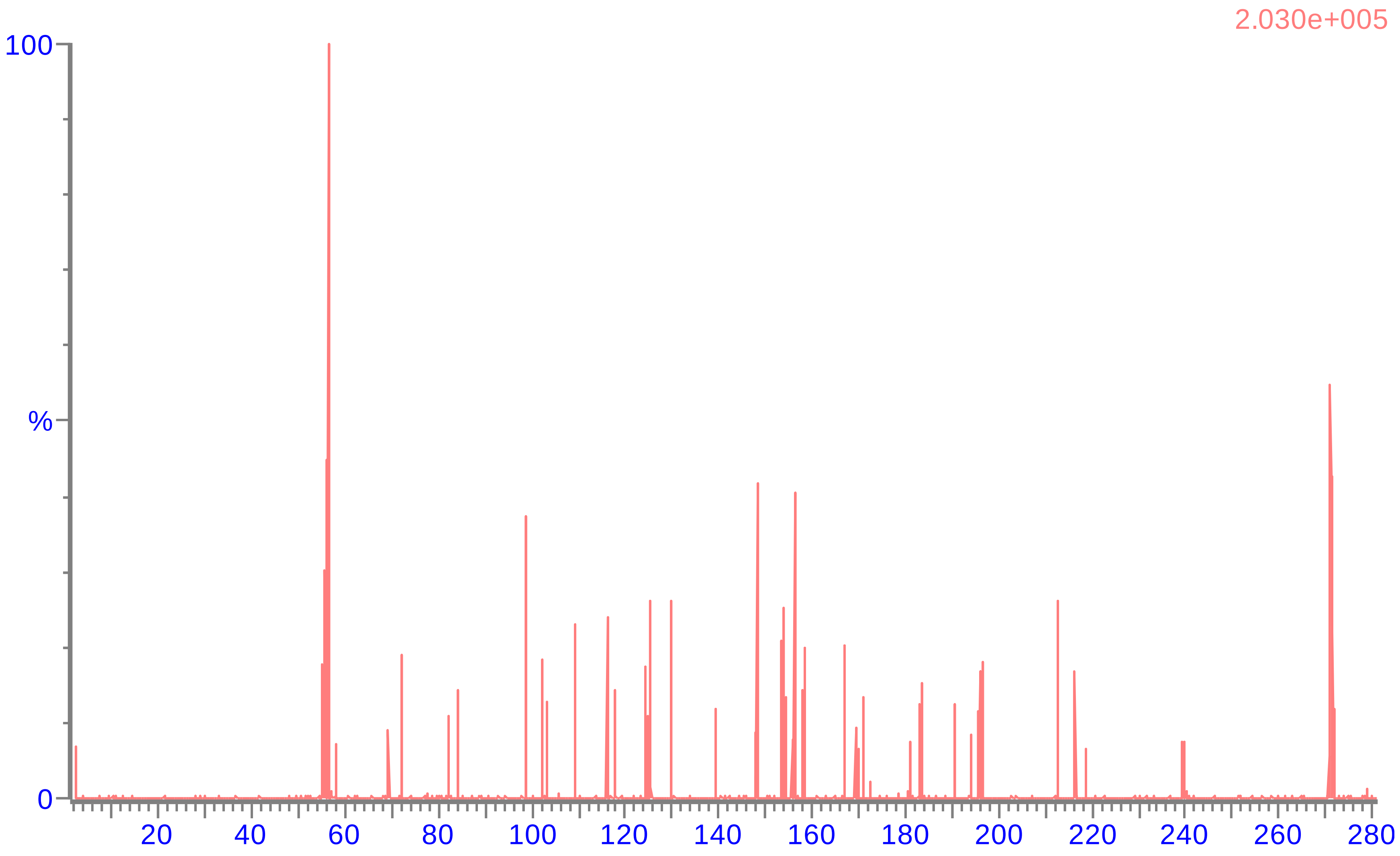

Figure 1.177: Mass spectrum for daughter fragment peak ES+, m/z 276.16 -> 148.03.

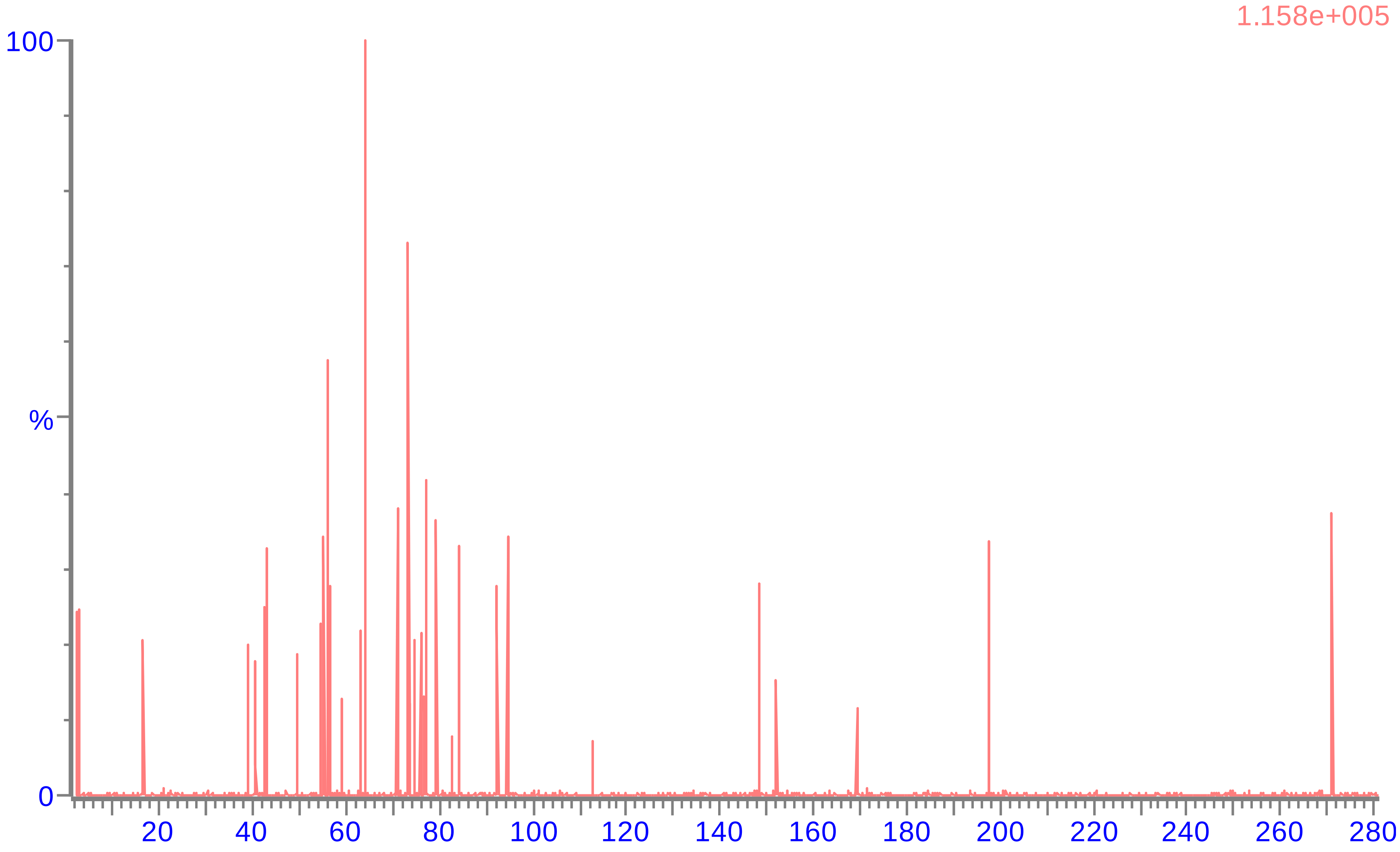

Figure 1.178: Mass spectrum for daughter fragment peak ES+, m/z 276.16 -> 77.07.

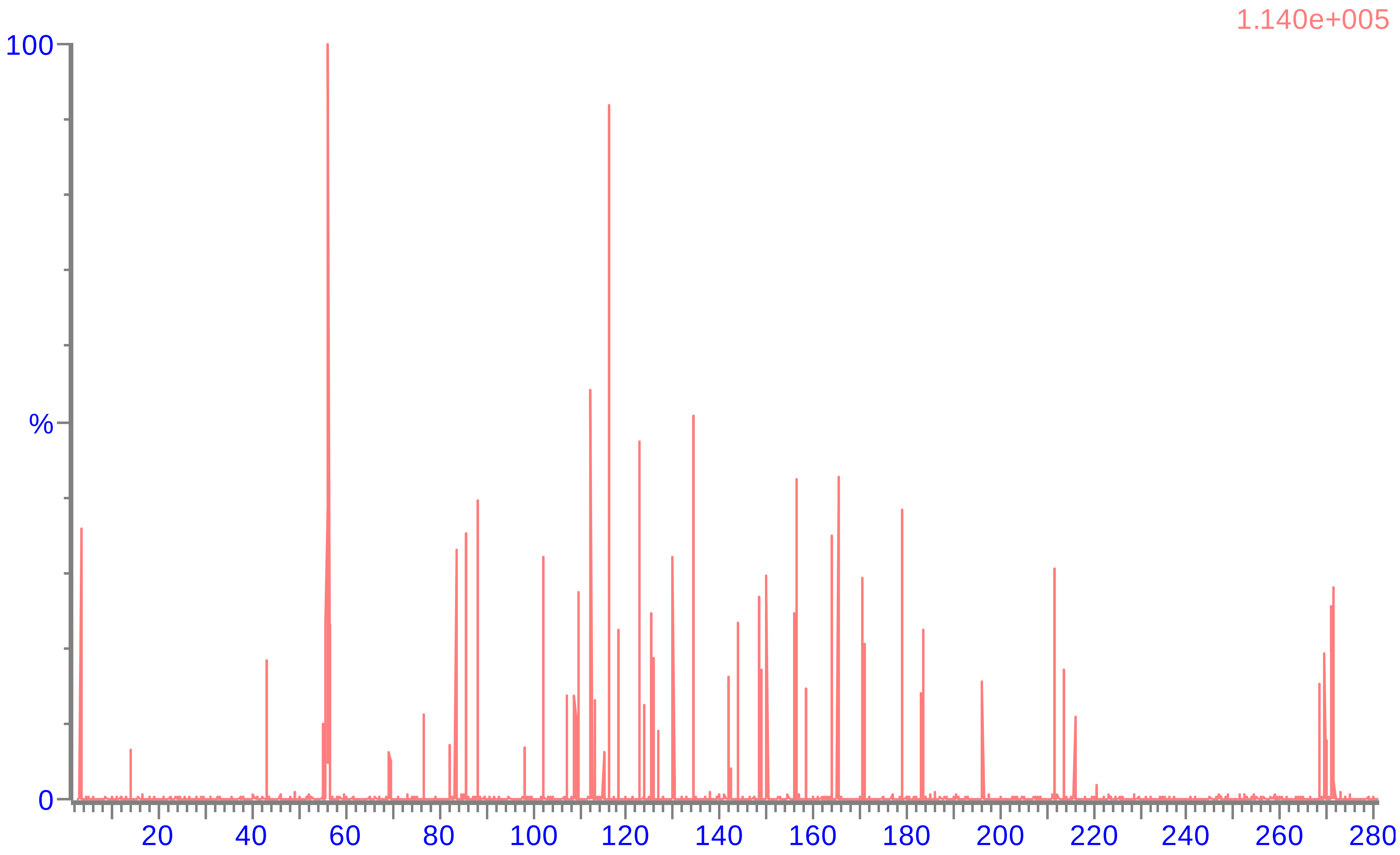

Figure 1.179: Mass spectrum for daughter fragment peak ES+, m/z 276.16 -> 56.19.

4a 3-nitro-*N*-(2-nitro-1-(*p*-tolyl)ethyl)aniline

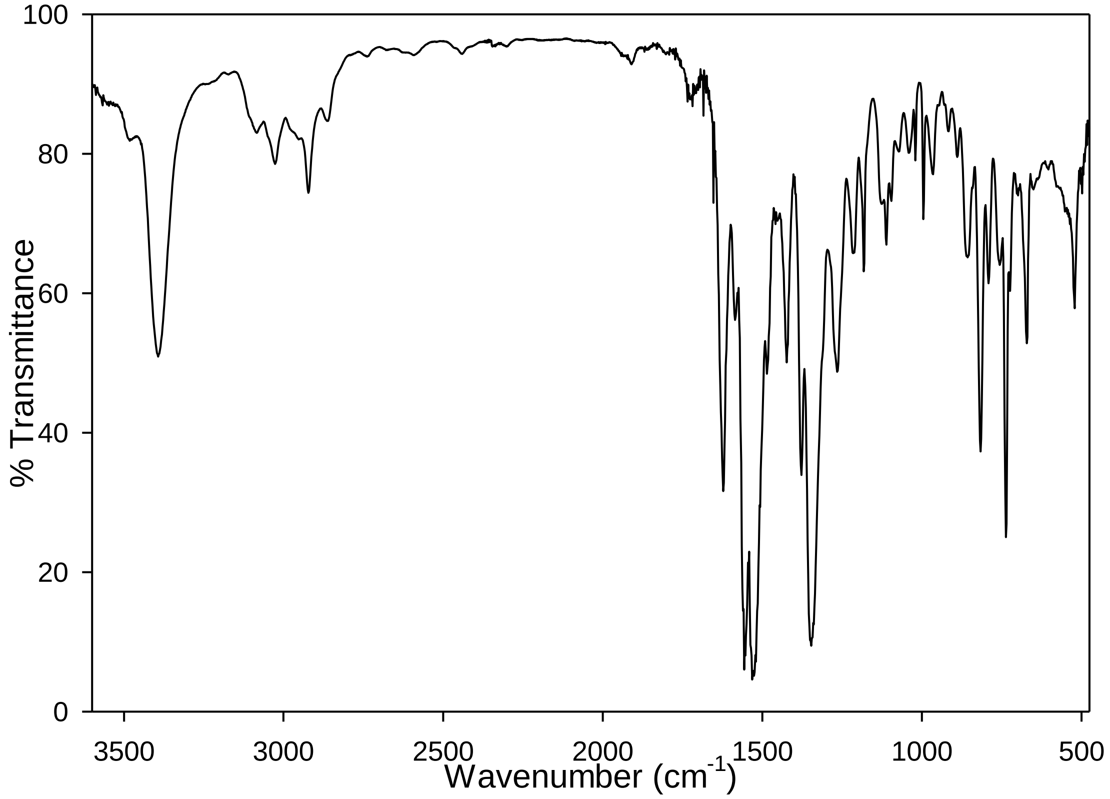

Figure 1.180: IR spectrum of **4a** 3-nitro-*N*-(2-nitro-1-(*p*-tolyl)ethyl)aniline.

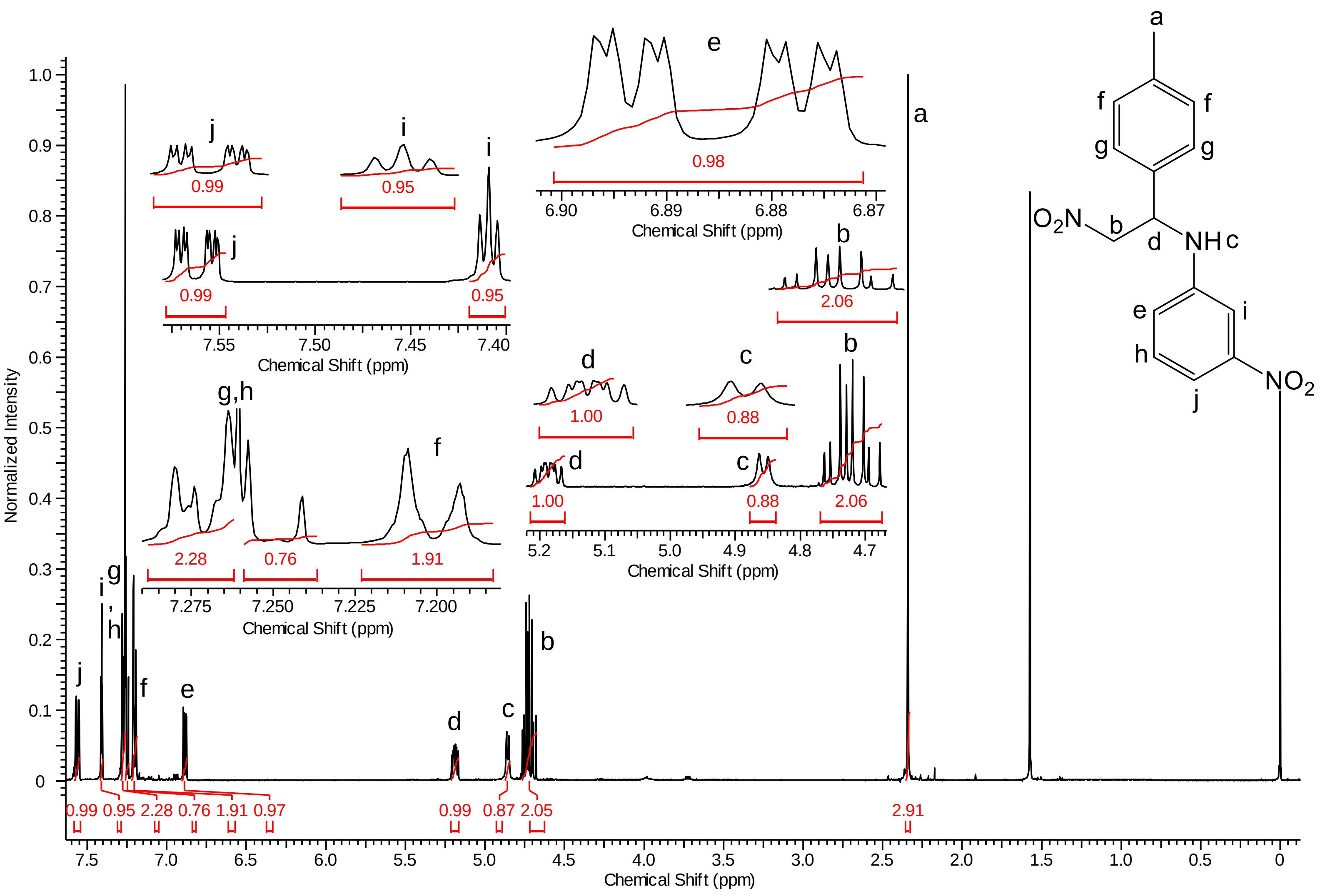

Figure 1.181: ^{1}H NMR spectrum of **4a** 3-nitro-*N*-(2-nitro-1-(*p*-tolyl)ethyl)aniline.

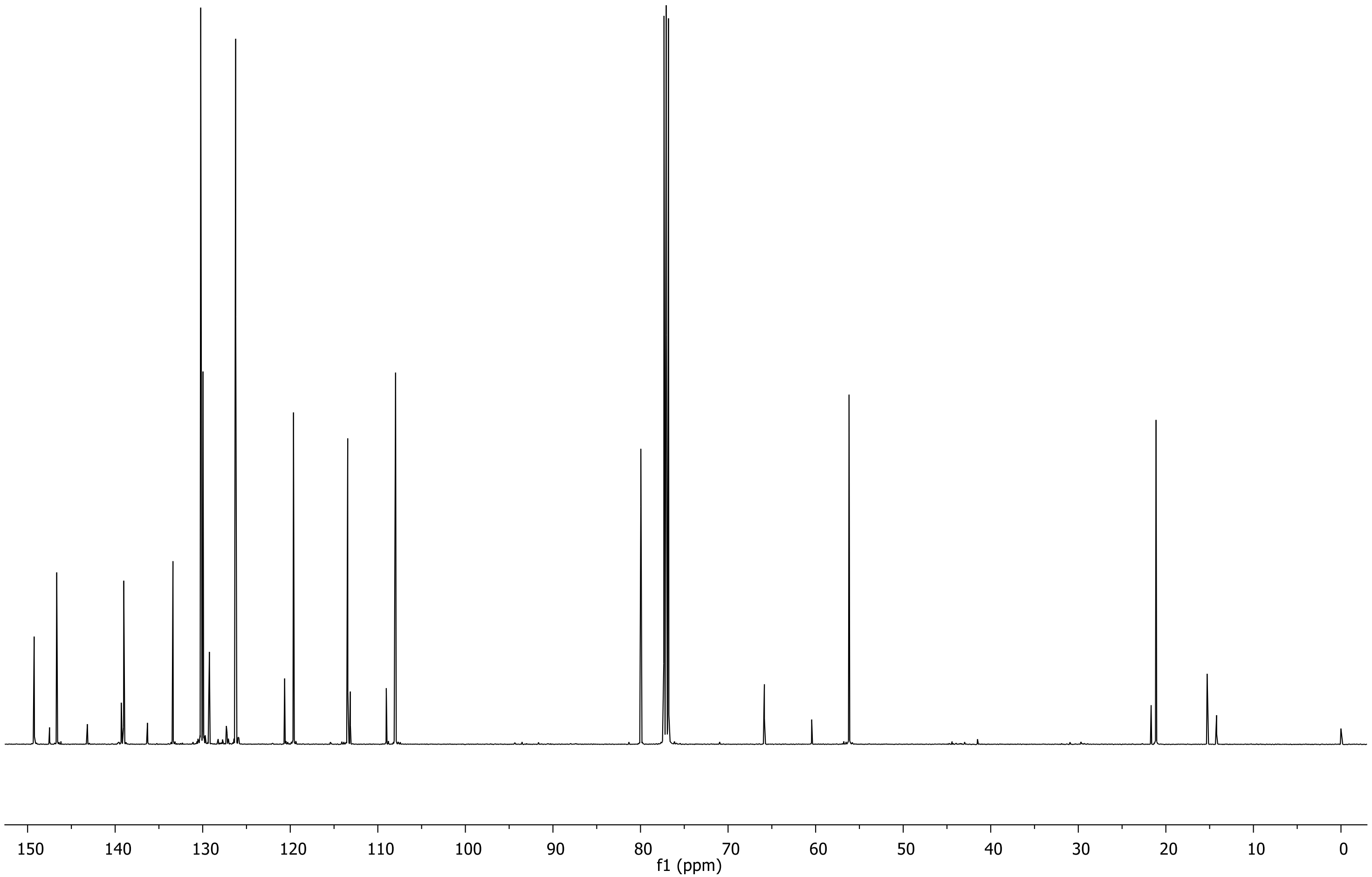

Figure 1.182: ^{13}C NMR spectrum of **4a** 3-nitro-*N*-(2-nitro-1-(*p*-tolyl)ethyl)aniline.

Table 1.25: MS data

Compound	Formula/Mass		Parent m/z	Cone Voltage	Daughters	Collision Energy	Ion Mode
**4a**	301	1	302.10	14	164.08	8	ES+
		2	302.10	14	118.10	16	ES+
		3	302.10	14	117.71	58	ES+
		4	302.10	14	91.05	58	ES+
		5	302.10	14	121.03	20	ES+

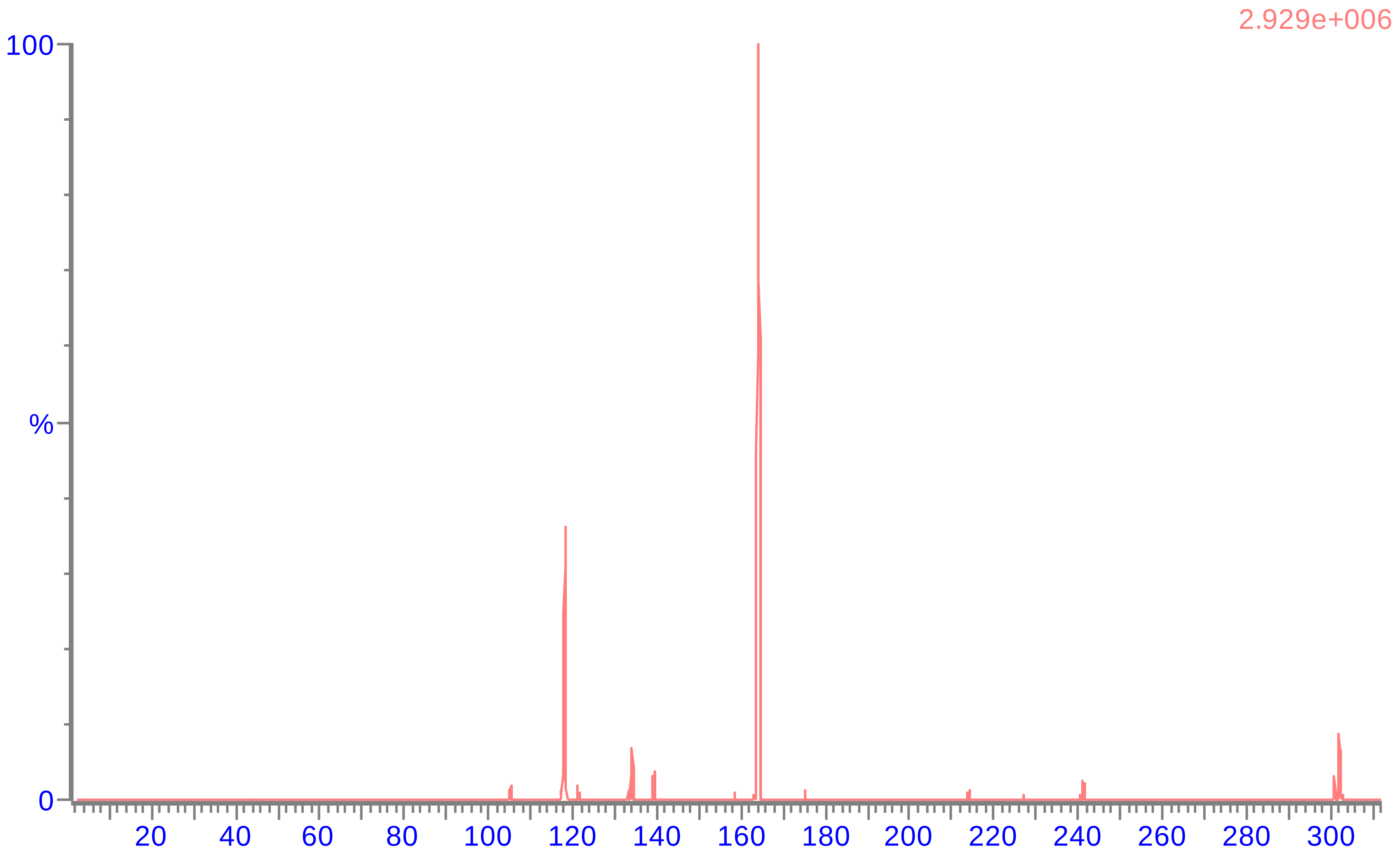

Figure 1.183: Mass spectrum for daughter fragment peak ES+, m/z 302.10 -> 164.08.

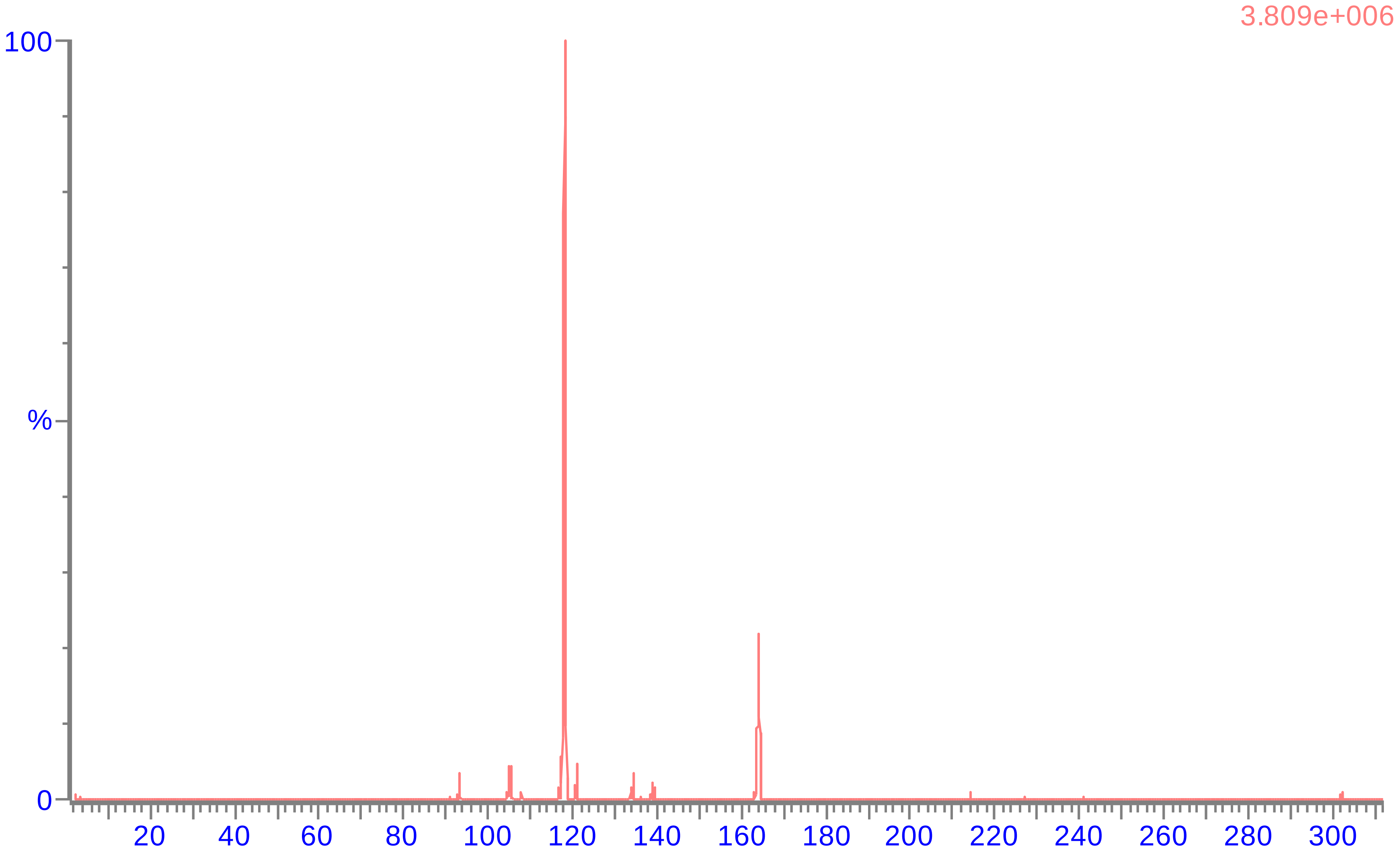

Figure 1.184: Mass spectrum for daughter fragment peak ES+, m/z 302.10 -> 118.10.

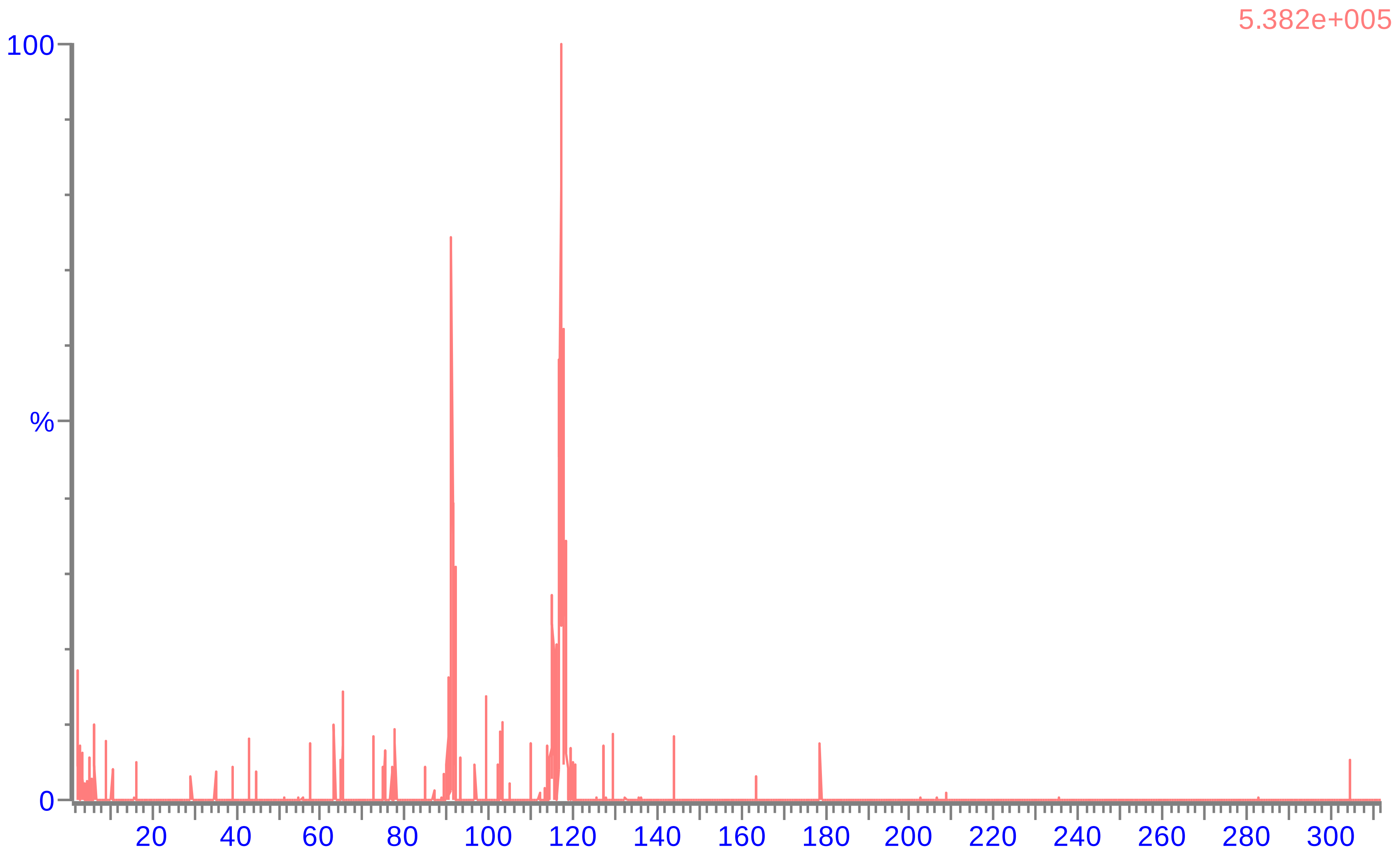

Figure 1.185: Mass spectrum for daughter fragment peak ES+, m/z 302.10 -> 117.71.

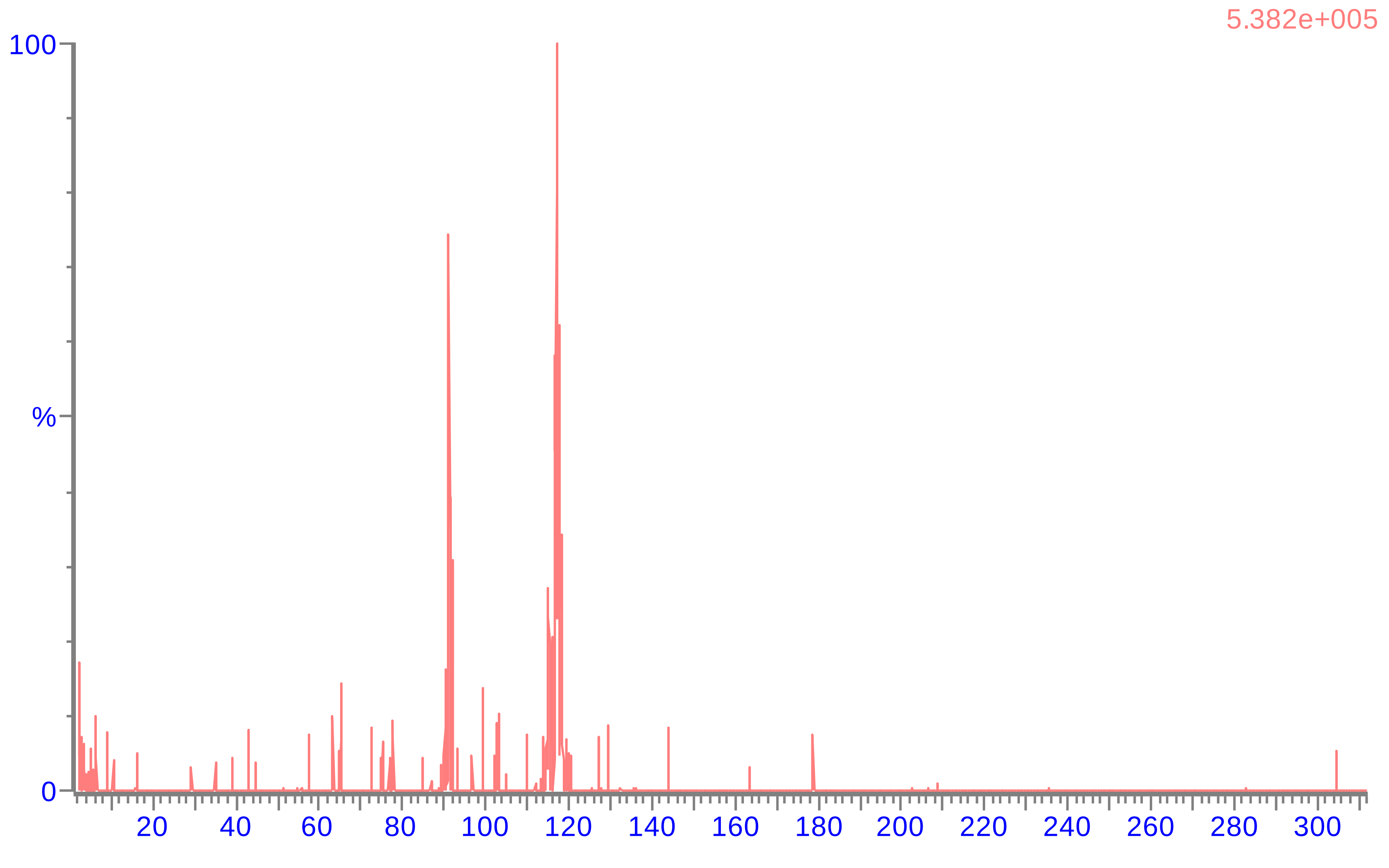

Figure 1.186: Mass spectrum for daughter fragment peak ES+, m/z 302.10 -> 91.05.

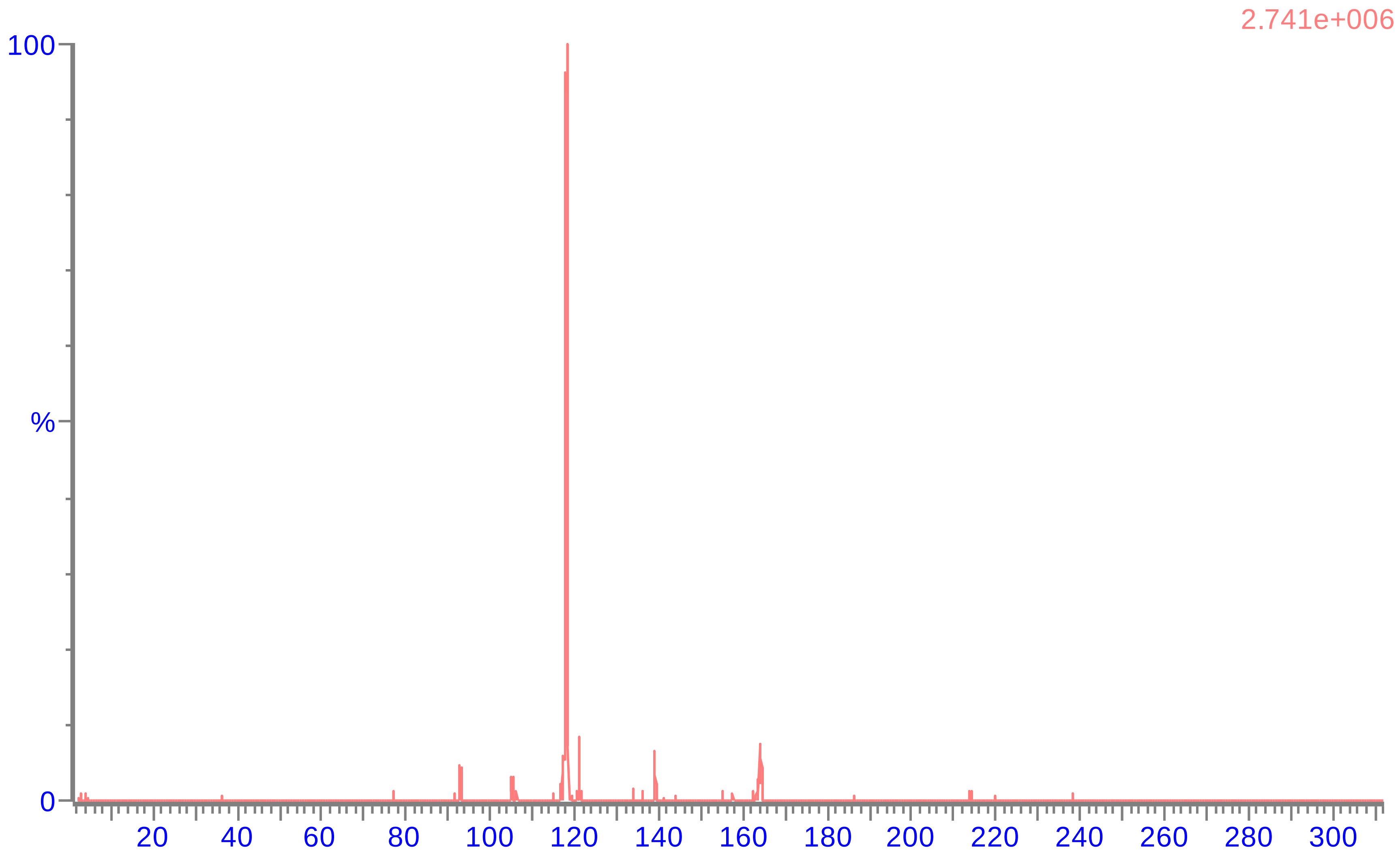

Figure 1.187: Mass spectrum for daughter fragment peak ES+, m/z 302.10 -> 121.03.

4b 4-chloro-*N*-(1-(4-methoxyphenyl)-2-nitroethyl)aniline

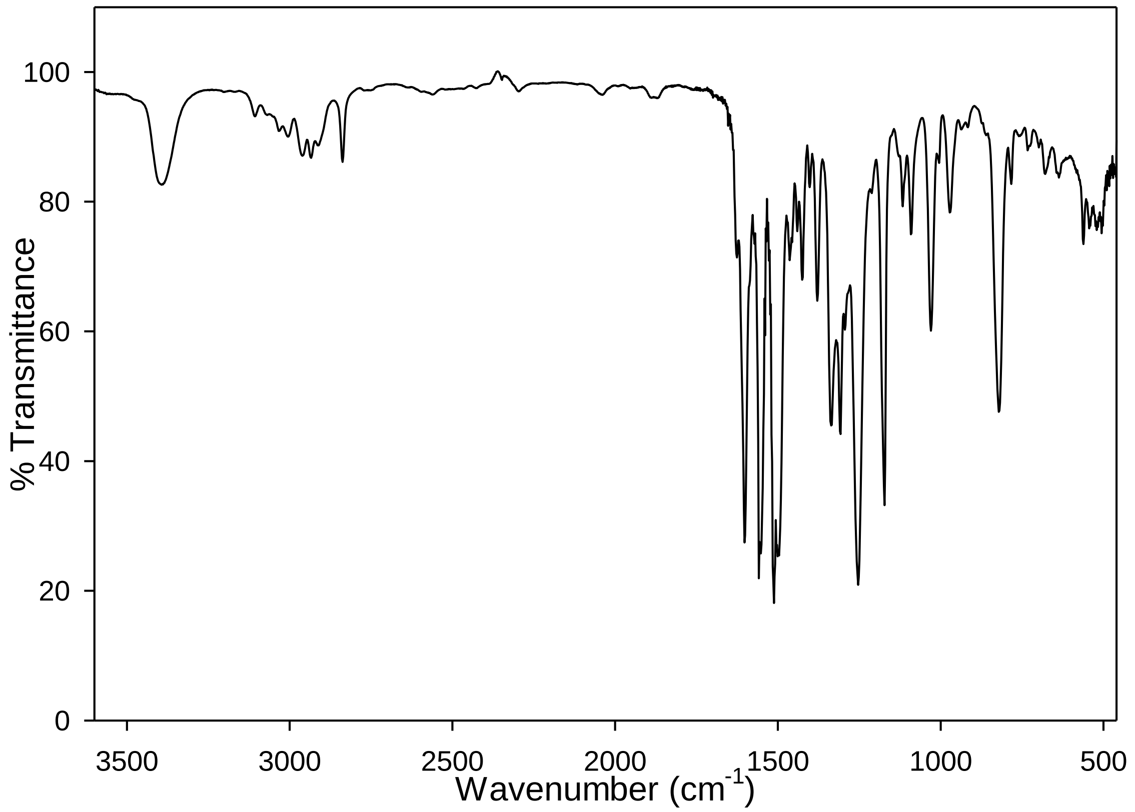

Figure 1.188: IR spectrum of **4b** 4-chloro-*N*-(1-(4-methoxyphenyl)-2-nitroethyl)aniline.

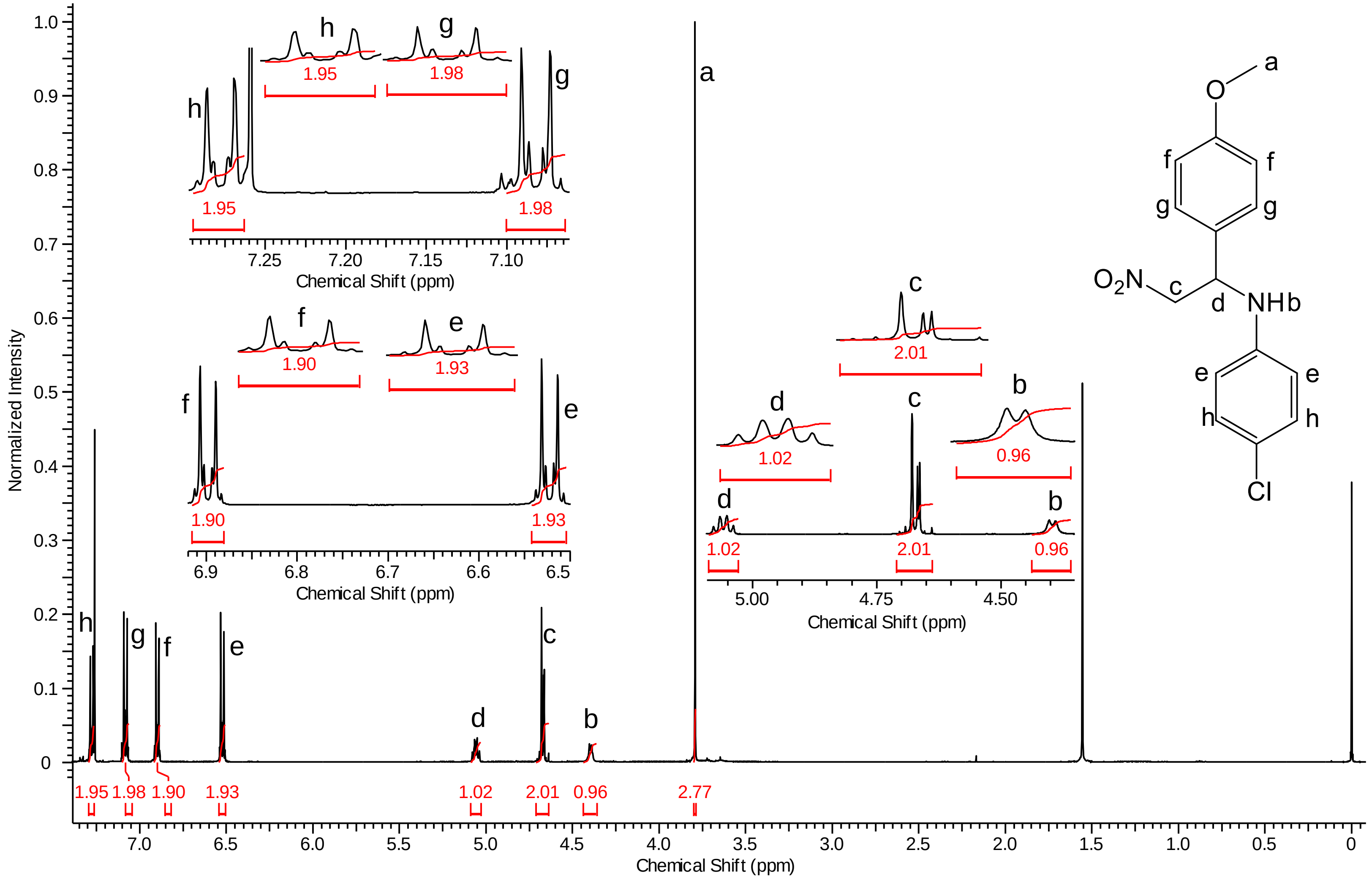

Figure 1.189: ^{1}H NMR spectrum of **4b** 4-chloro-*N*-(1-(4-methoxyphenyl)-2-nitroethyl)aniline.

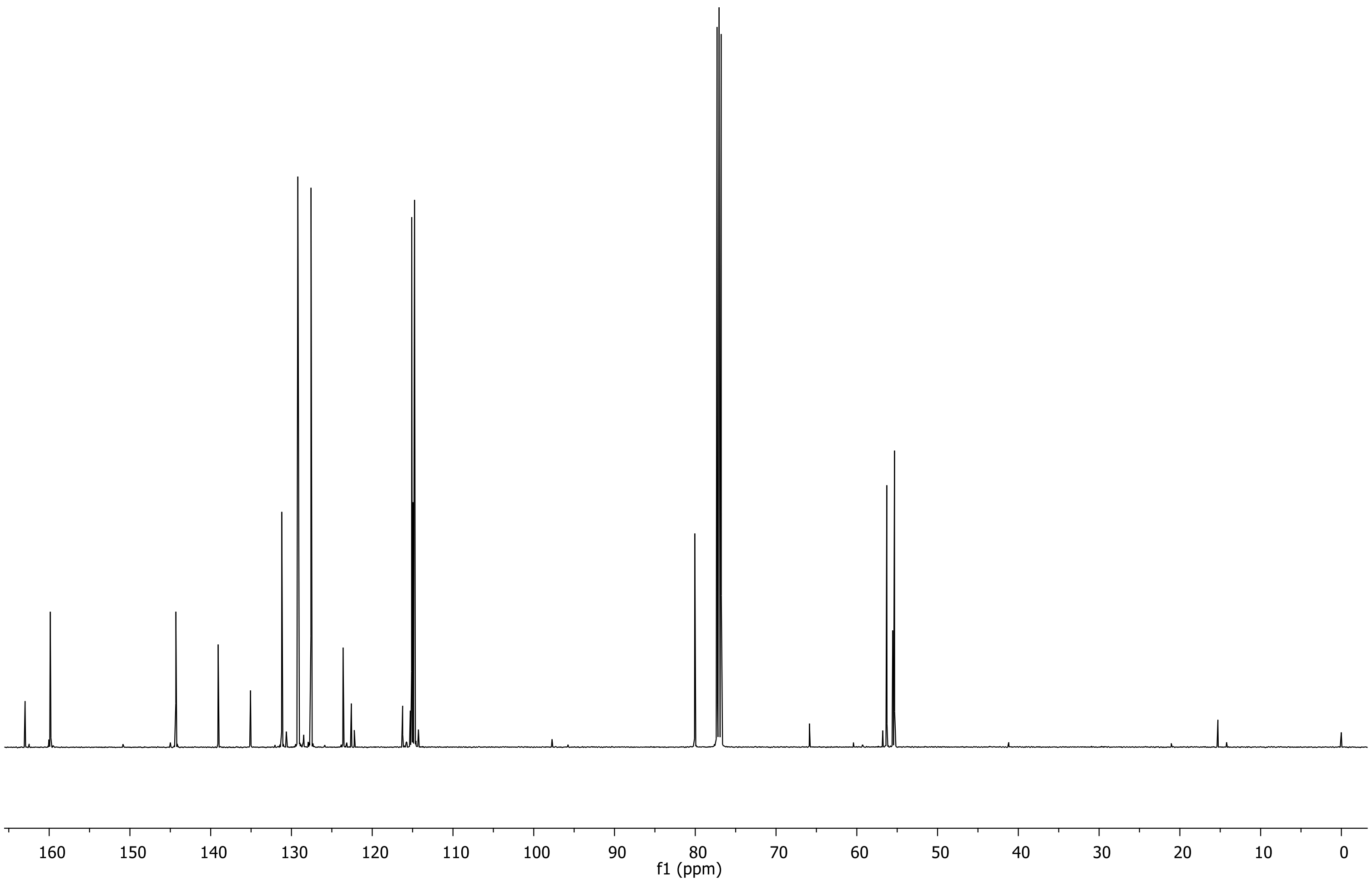

Figure 1.190: ^{13}C NMR spectrum of **4b** 4-chloro-*N*-(1-(4-methoxyphenyl)-2-nitroethyl)aniline.

Table 1.26: MS data

Compound	Formula/Mass		Parent m/z	Cone Voltage	Daughters	Collision Energy	Ion Mode
**4b**	305.8	1	307.09	12	180.05	6	ES+
		2	307.09	12	91.06	52	ES+
		3	307.09	12	119.04	40	ES+

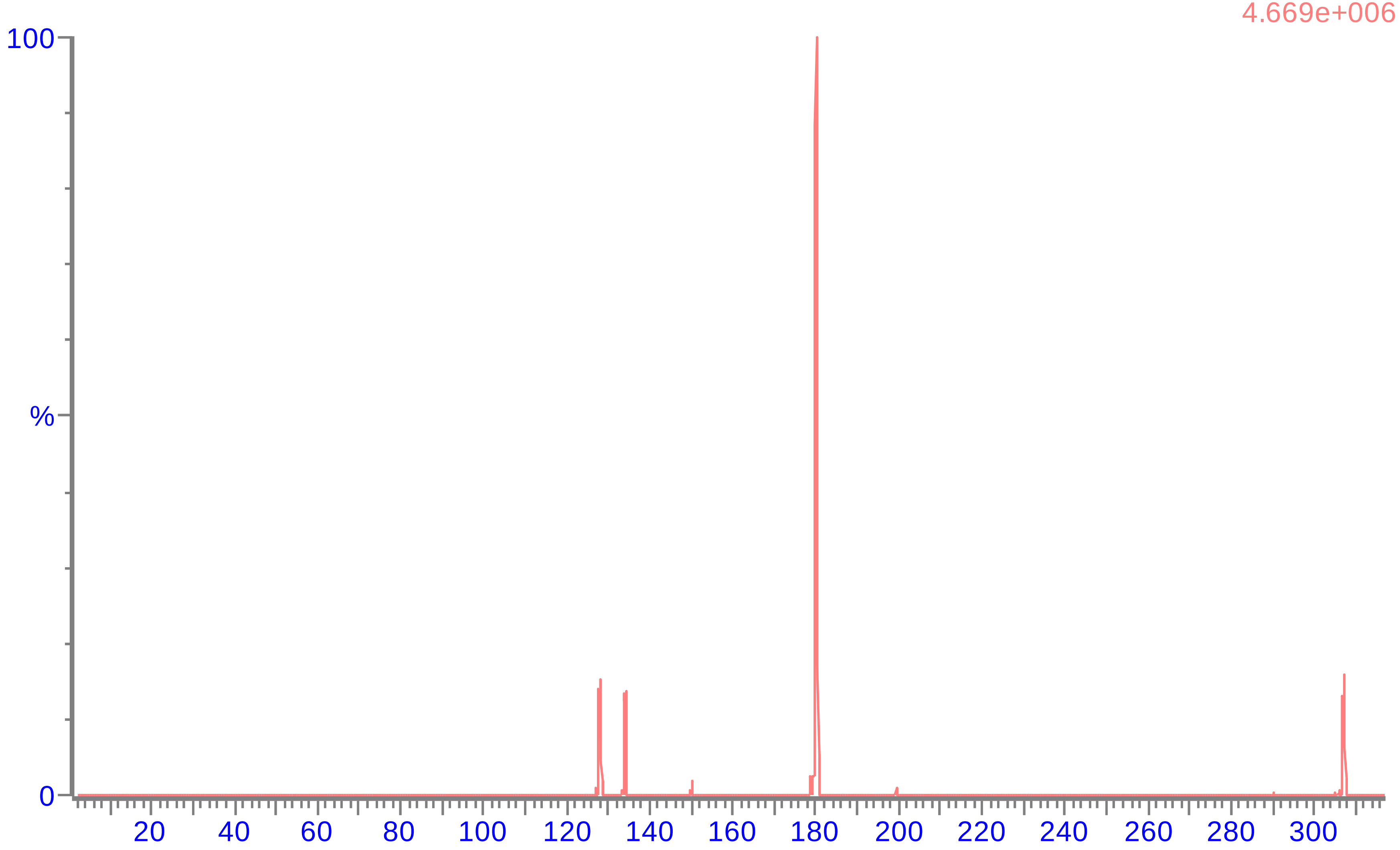

Figure 1.191: Mass spectrum for daughter fragment peak ES+, m/z 307.09 -> 180.05.

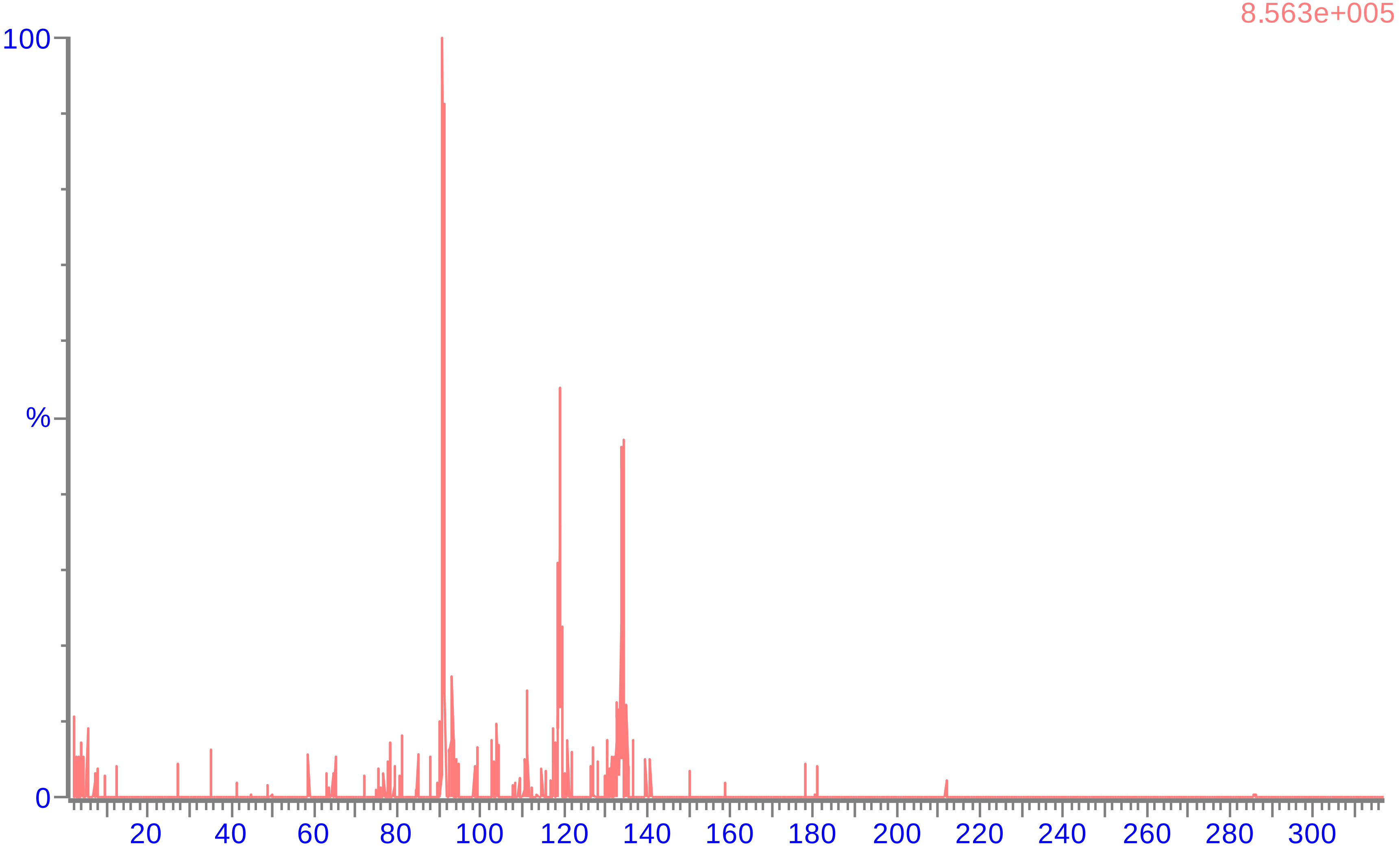

Figure 1.192: Mass spectrum for daughter fragment peak ES+, m/z 307.09 -> 91.06.

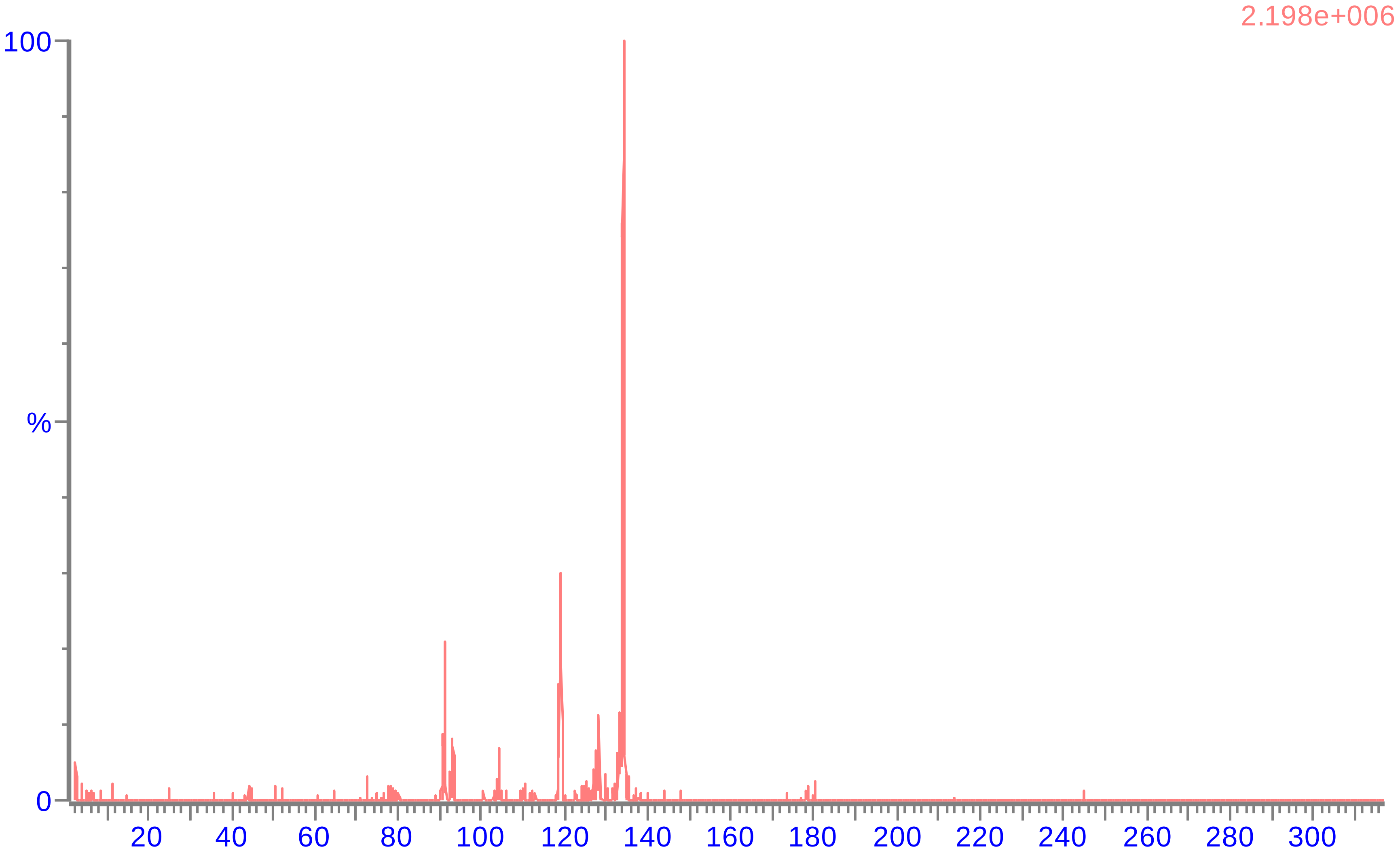

Figure 1.193: Mass spectrum for daughter fragment peak ES+, m/z 307.09 -> 119.04.

4c 3-(1-((4-methoxyphenyl)amino)-2-nitroethyl)phenol

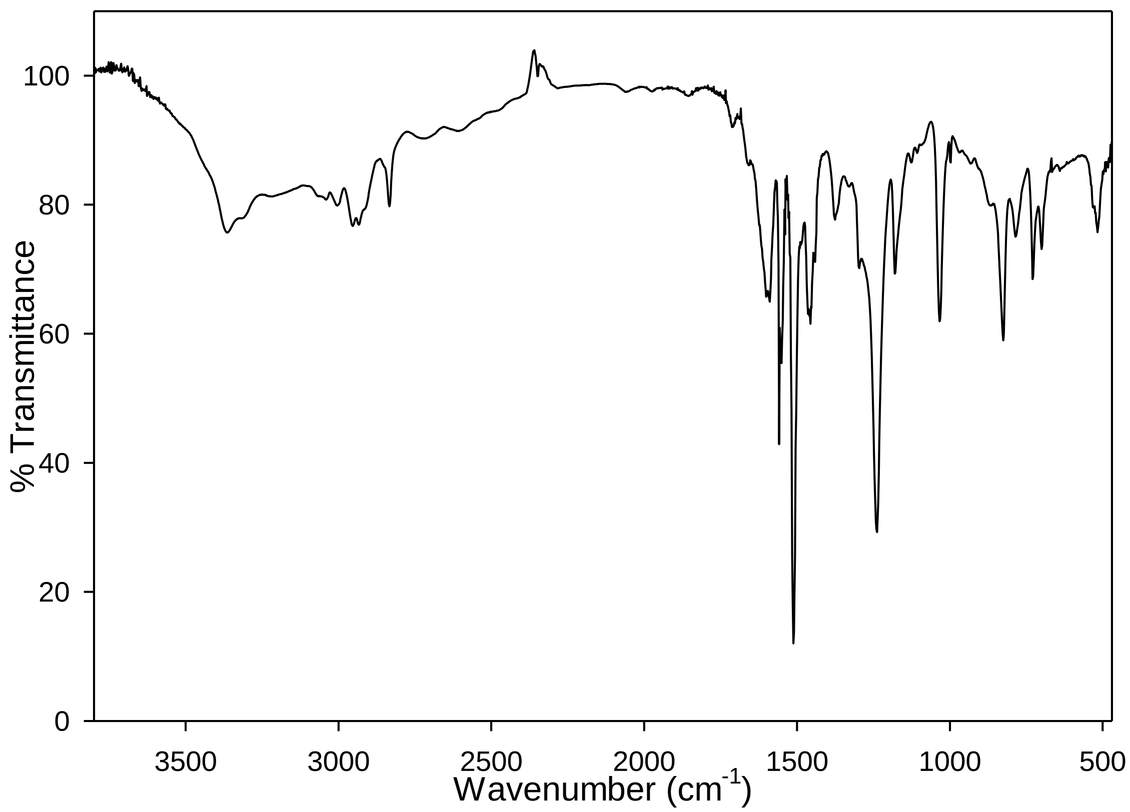

Figure 1.194: IR spectrum of **4c** 3-(1-((4-methoxyphenyl)amino)-2-nitroethyl)phenol.

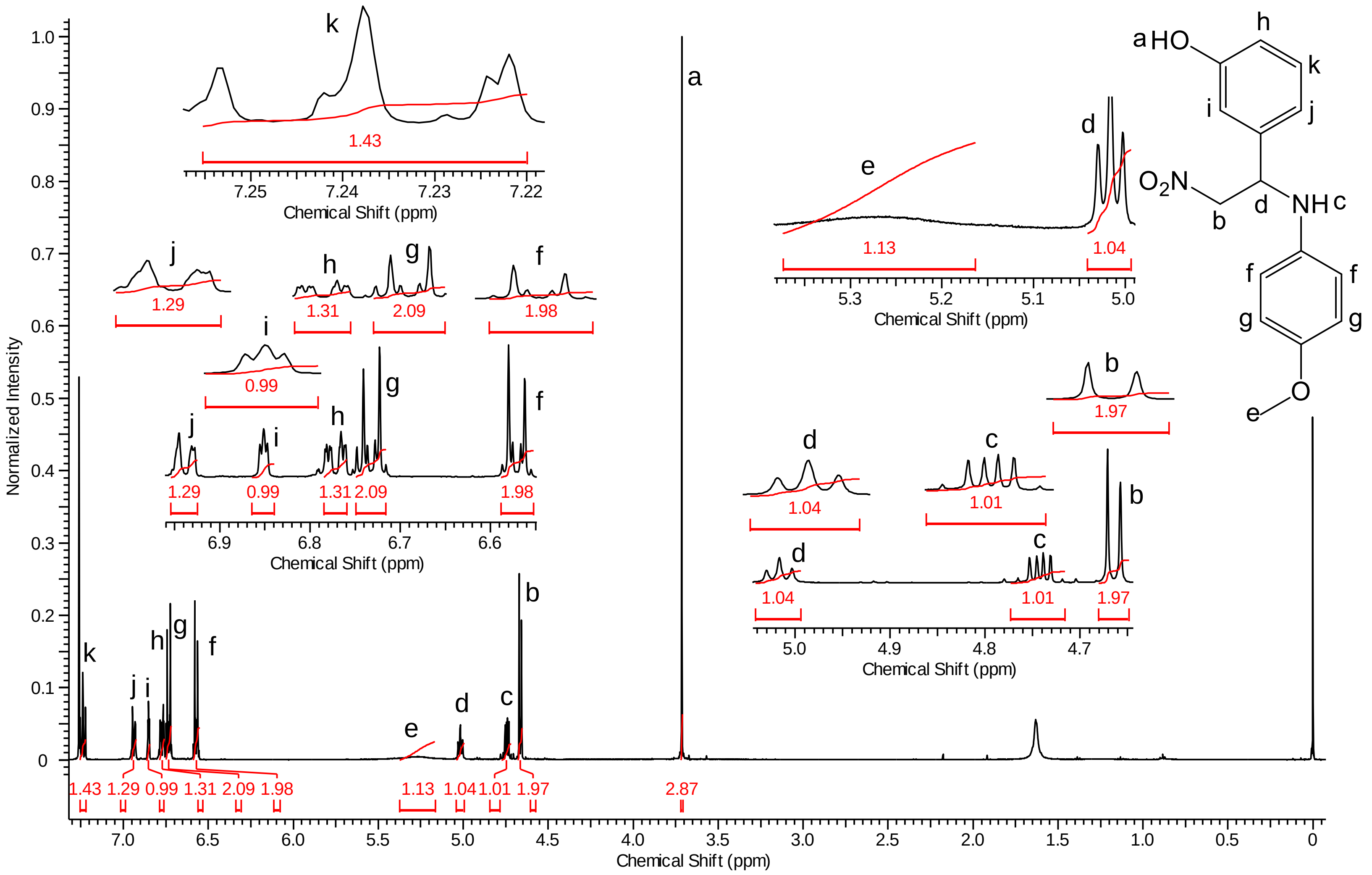

Figure 1.195: ^{1}H NMR spectrum of **4c** 3-(1-((4-methoxyphenyl)amino)-2-nitroethyl)phenol.

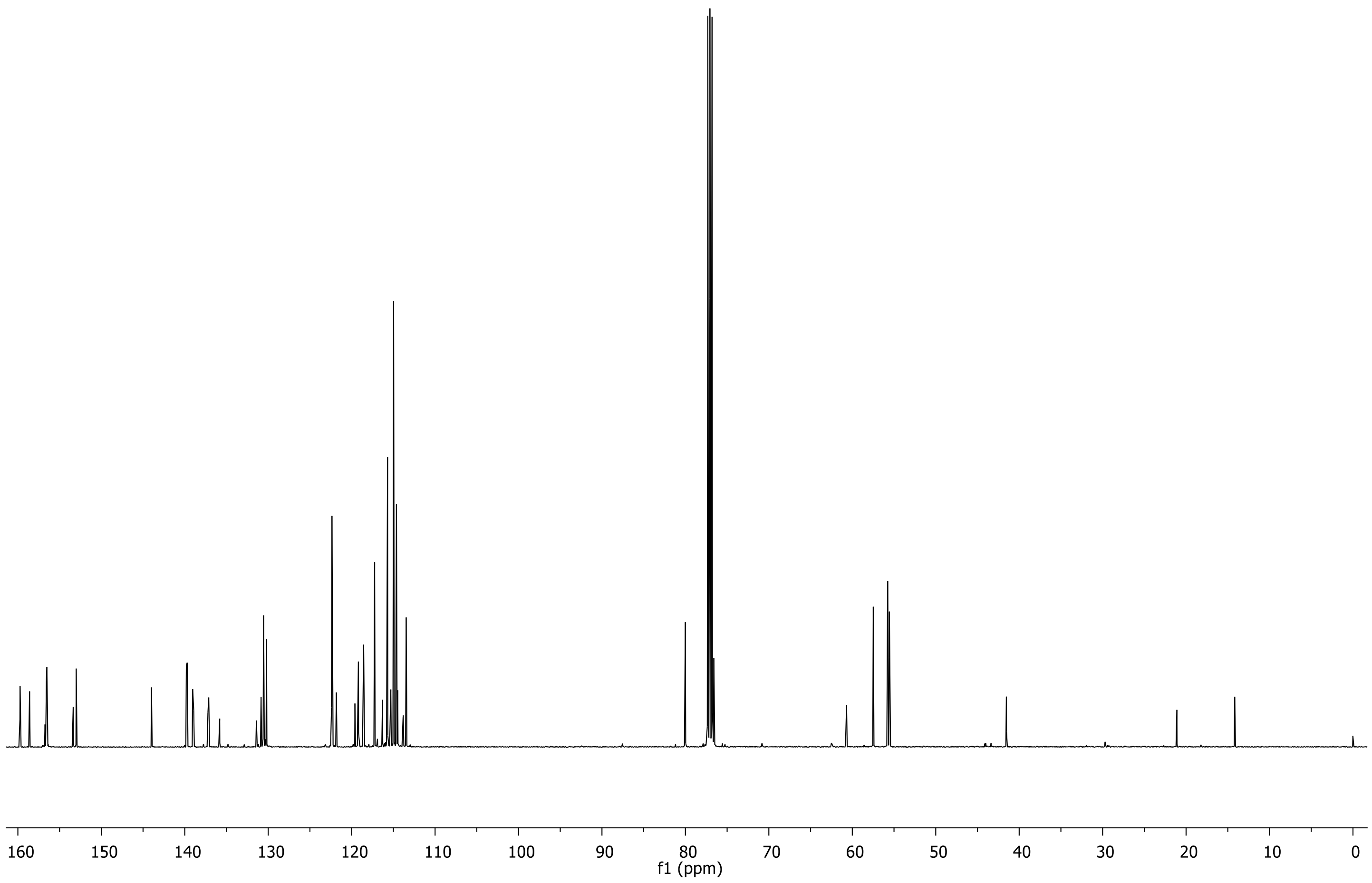

Figure 1.196: ^{13}C NMR spectrum of **4c** 3-(1-((4-methoxyphenyl)amino)-2-nitroethyl)phenol.

Table 1.27: MS data

Compound	Formula/Mass		Parent m/z	Cone Voltage	Daughters	Collision Energy	Ion Mode
**4c**	288	1	289.07	14	166.06	8	ES+
		2	289.07	14	123.92	14	ES+
		3	289.07	38	200.15	14	ES+

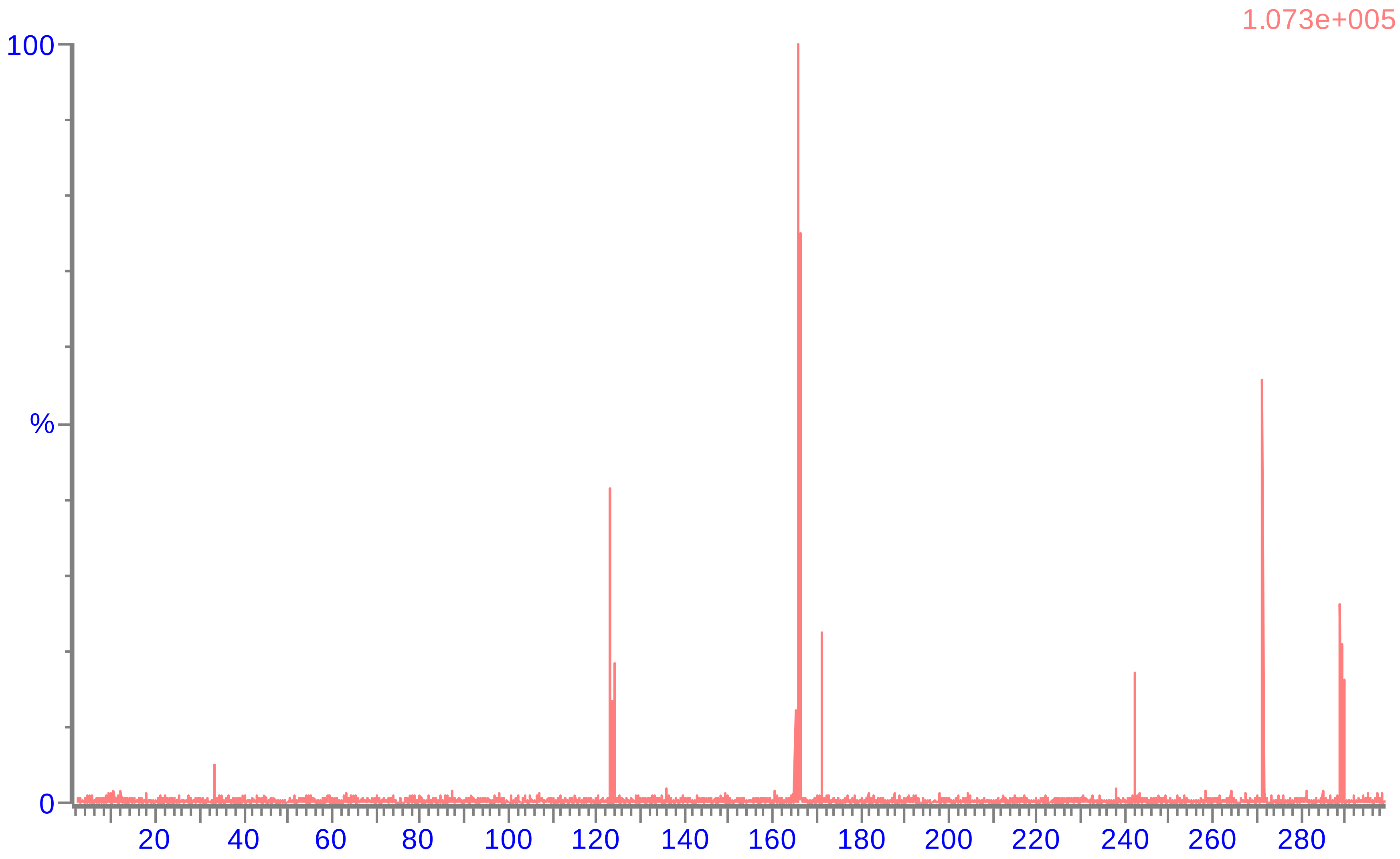

Figure 1.197: Mass spectrum for daughter fragment peak ES+, m/z 289.07 -> 166.06.

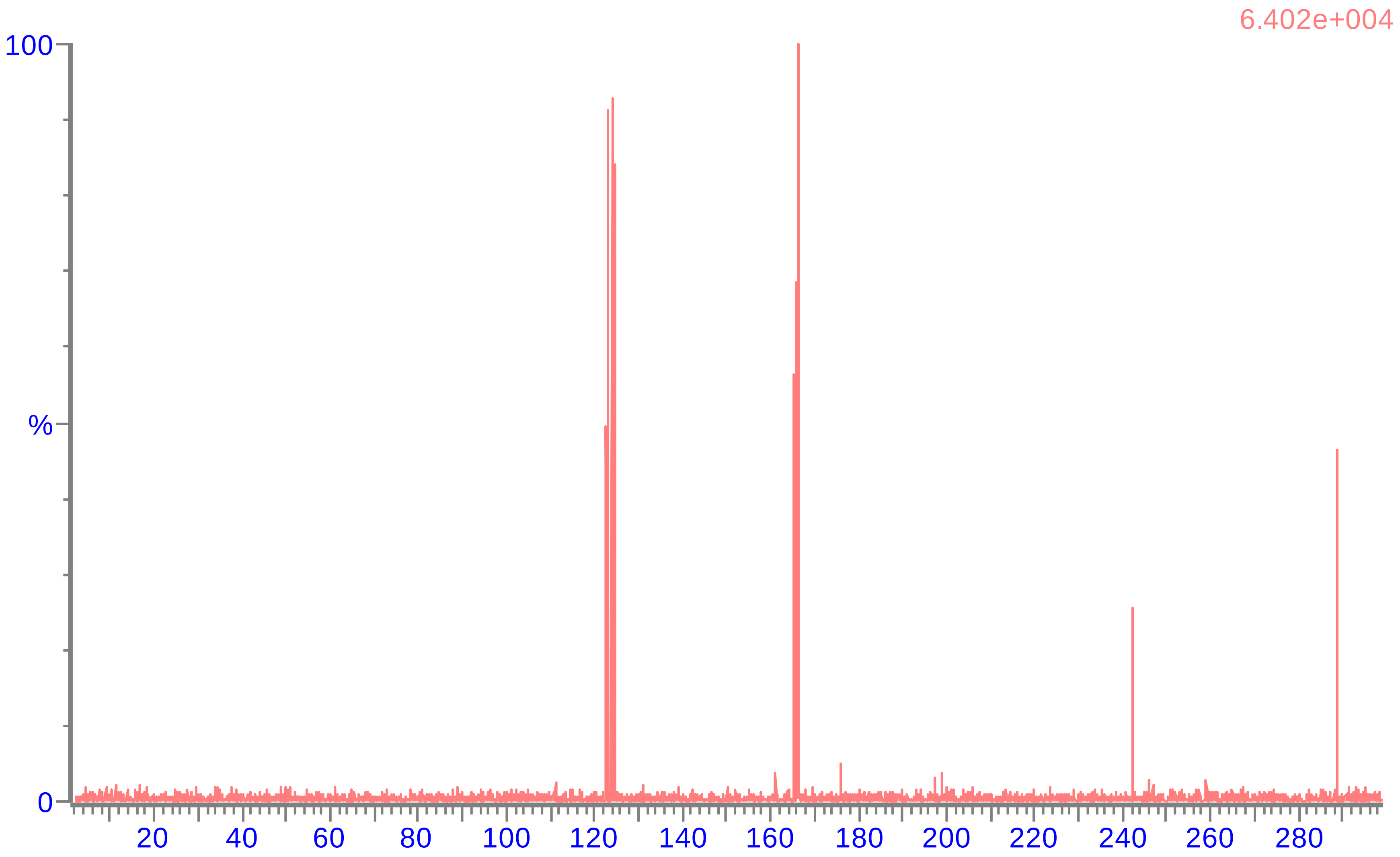

Figure 1.198: Mass spectrum for daughter fragment peak ES+, m/z 289.07 -> 123.92.

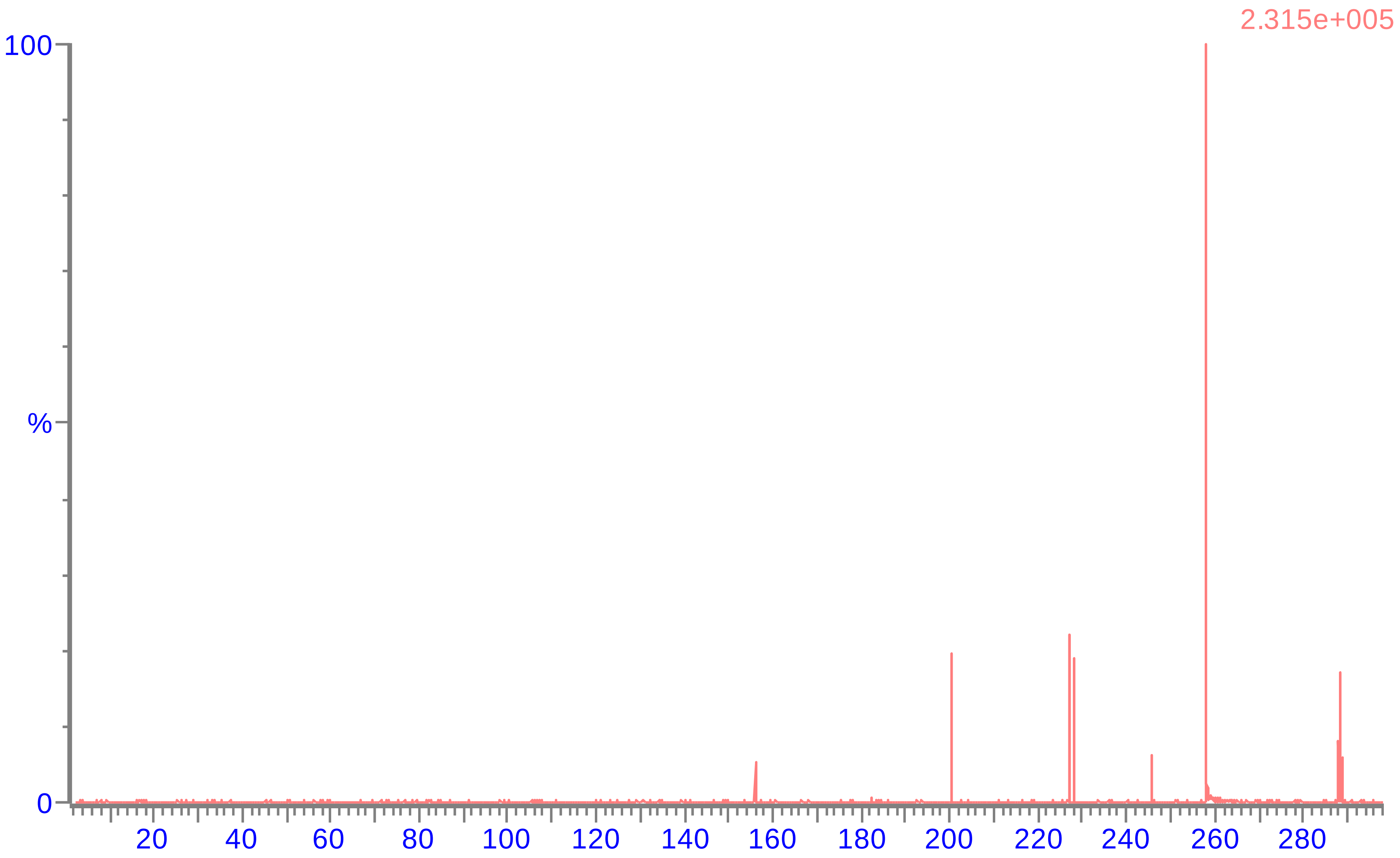

Figure 1.199: Mass spectrum for daughter fragment peak ES+, m/z 289.07 -> 200.15.

4d 4-bromo-*N*-(1-cyclohexyl-2-nitroethyl)aniline

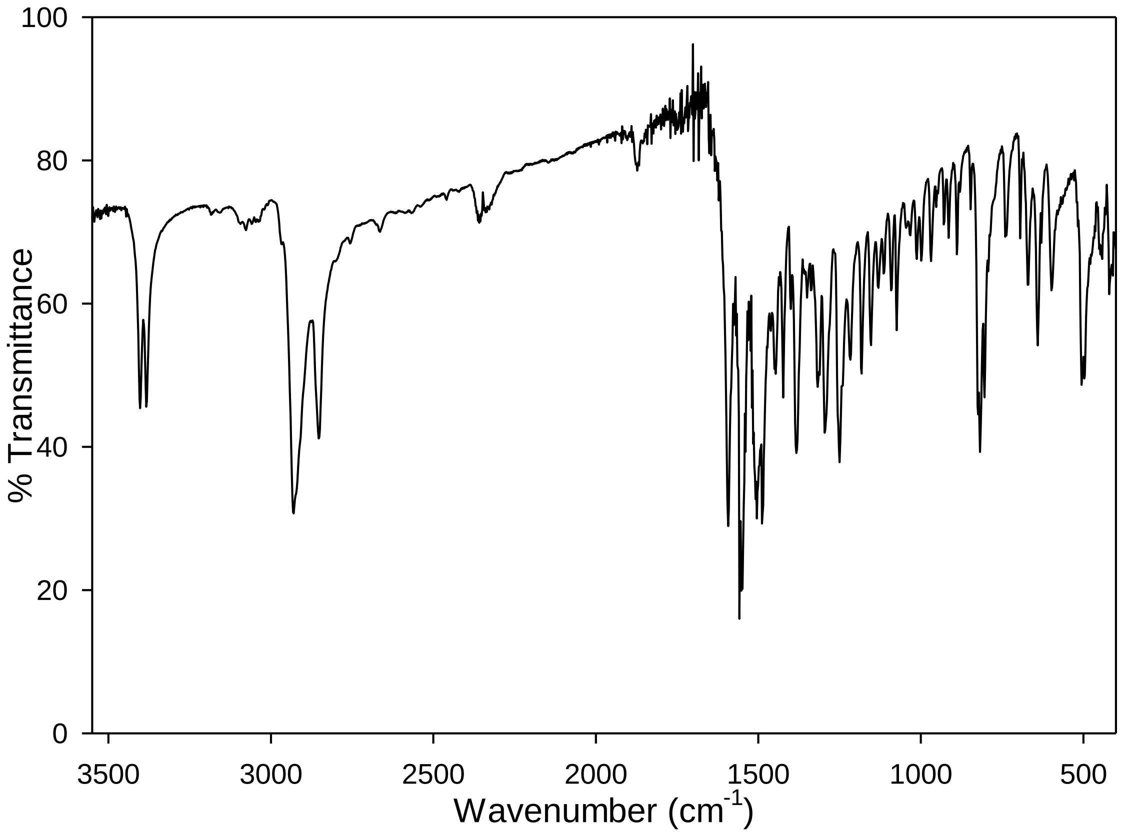

Figure 1.200: IR spectrum of **4d** 4-bromo-*N*-(1-cyclohexyl-2-nitroethyl)aniline.

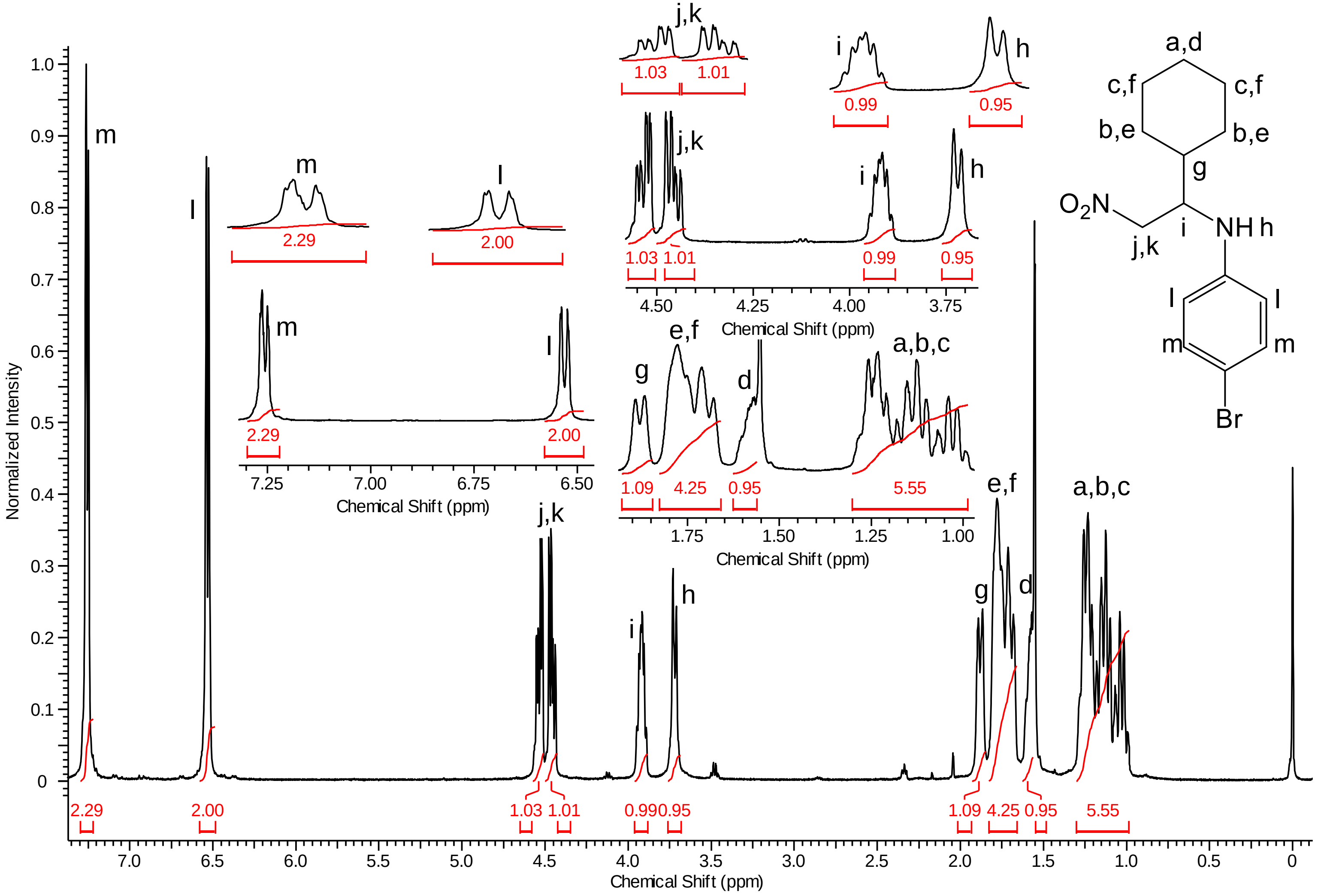

Figure 1.201: ^{1}H NMR spectrum of **4d** 4-bromo-*N*-(1-cyclohexyl-2-nitroethyl)aniline.

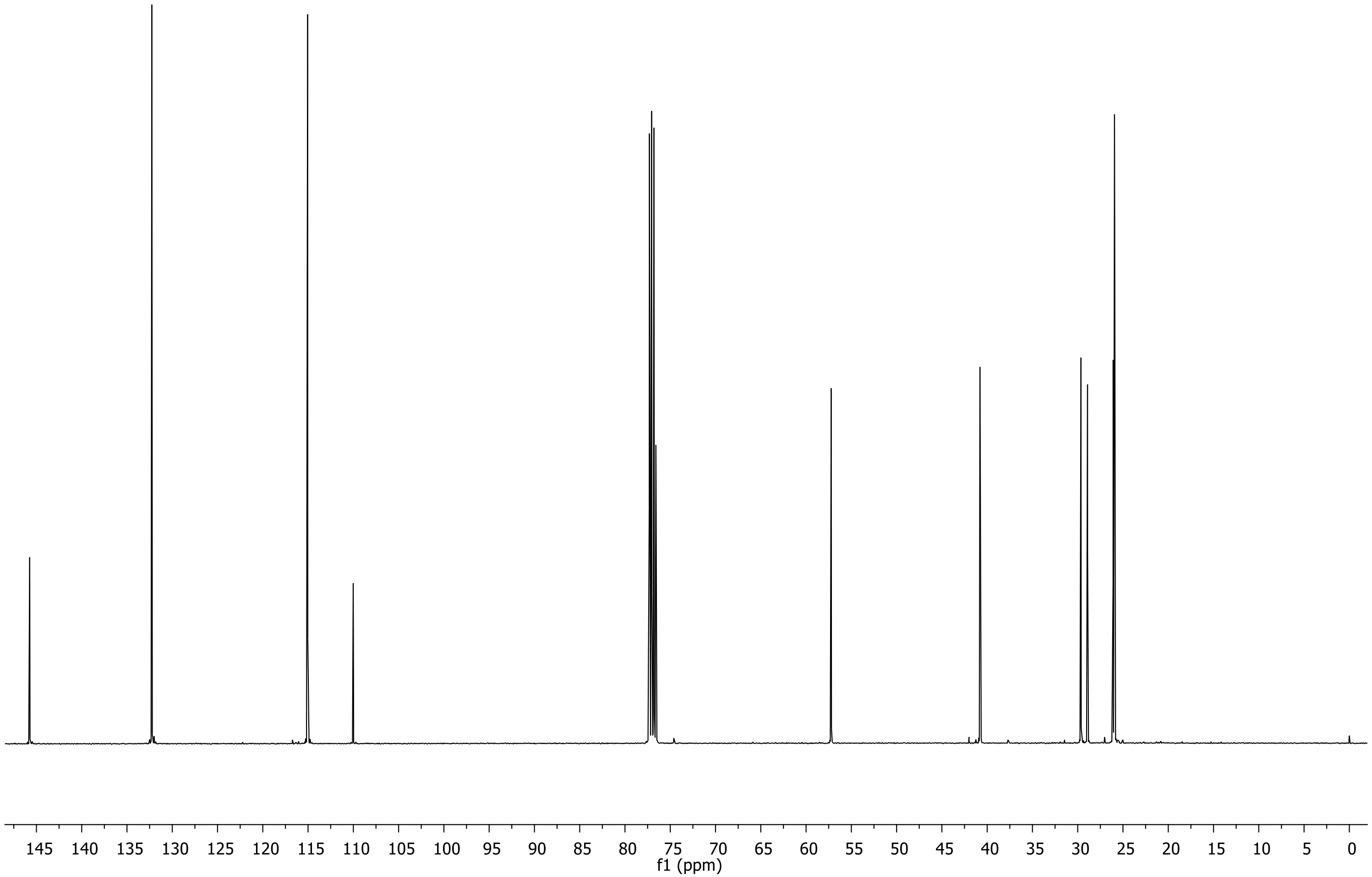

Figure 1.202: ^{13}C NMR spectrum of **4d** 4-bromo-*N*-(1-cyclohexyl-2-nitroethyl)aniline.

Table 1.28: MS data.

Compound	Formula/Mass		Parent m/z	Cone Voltage	Daughters	Collision Energy	Ion Mode
**4d**	326	1	325.03	18	154.07	6	ES-
		2	325.03	18	169.93	12	ES-
		3	325.03	18	78.89	28	ES-
		4	325.03	18	136.05	18	ES-
		5	325.03	18	60.02	12	ES-

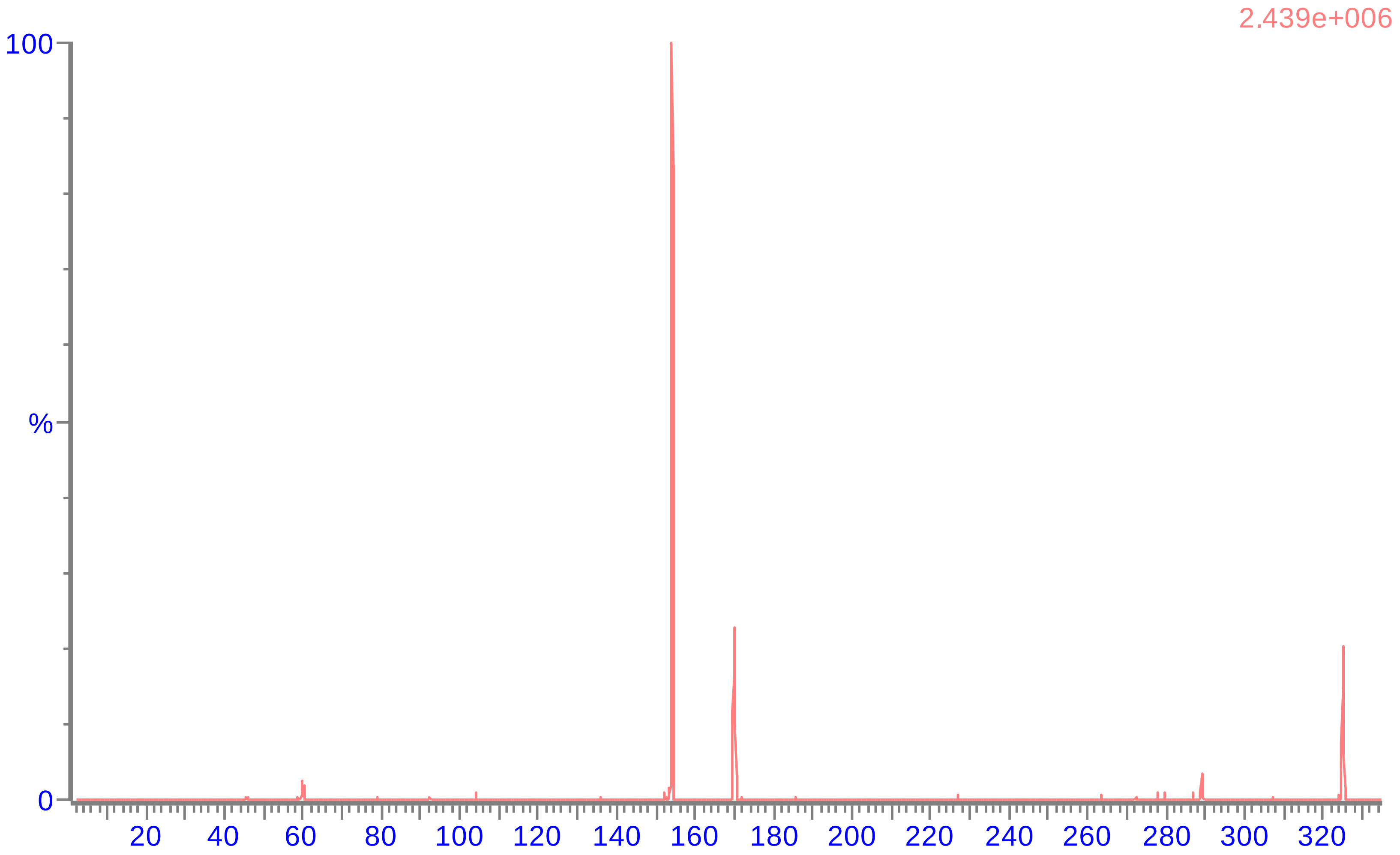

Figure 1.203: Mass spectrum for daughter fragment peak ES+, m/z 325.03 -> 154.07.

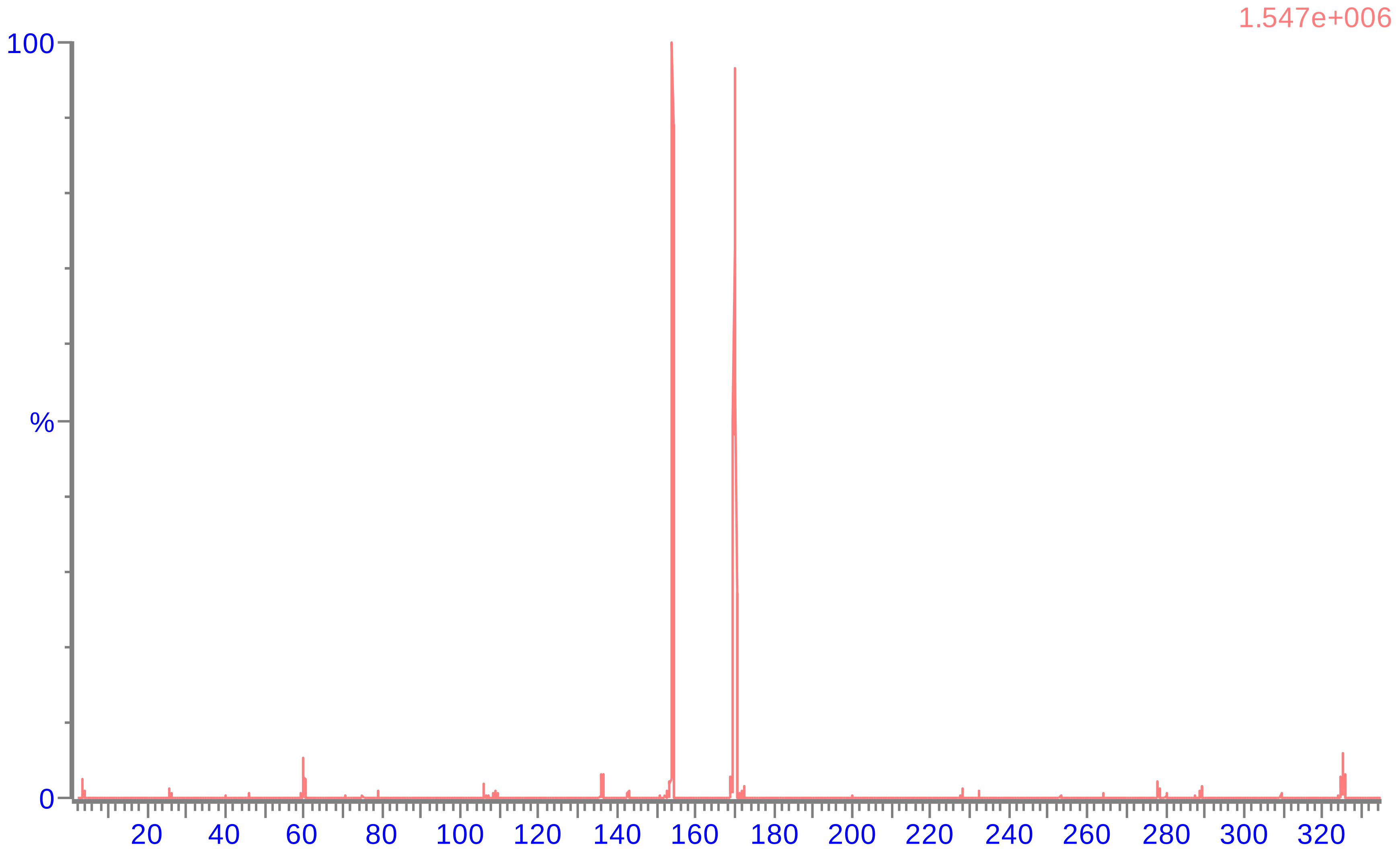

Figure 1.204: Mass spectrum for daughter fragment peak ES+, m/z 325.03 -> 169.93.

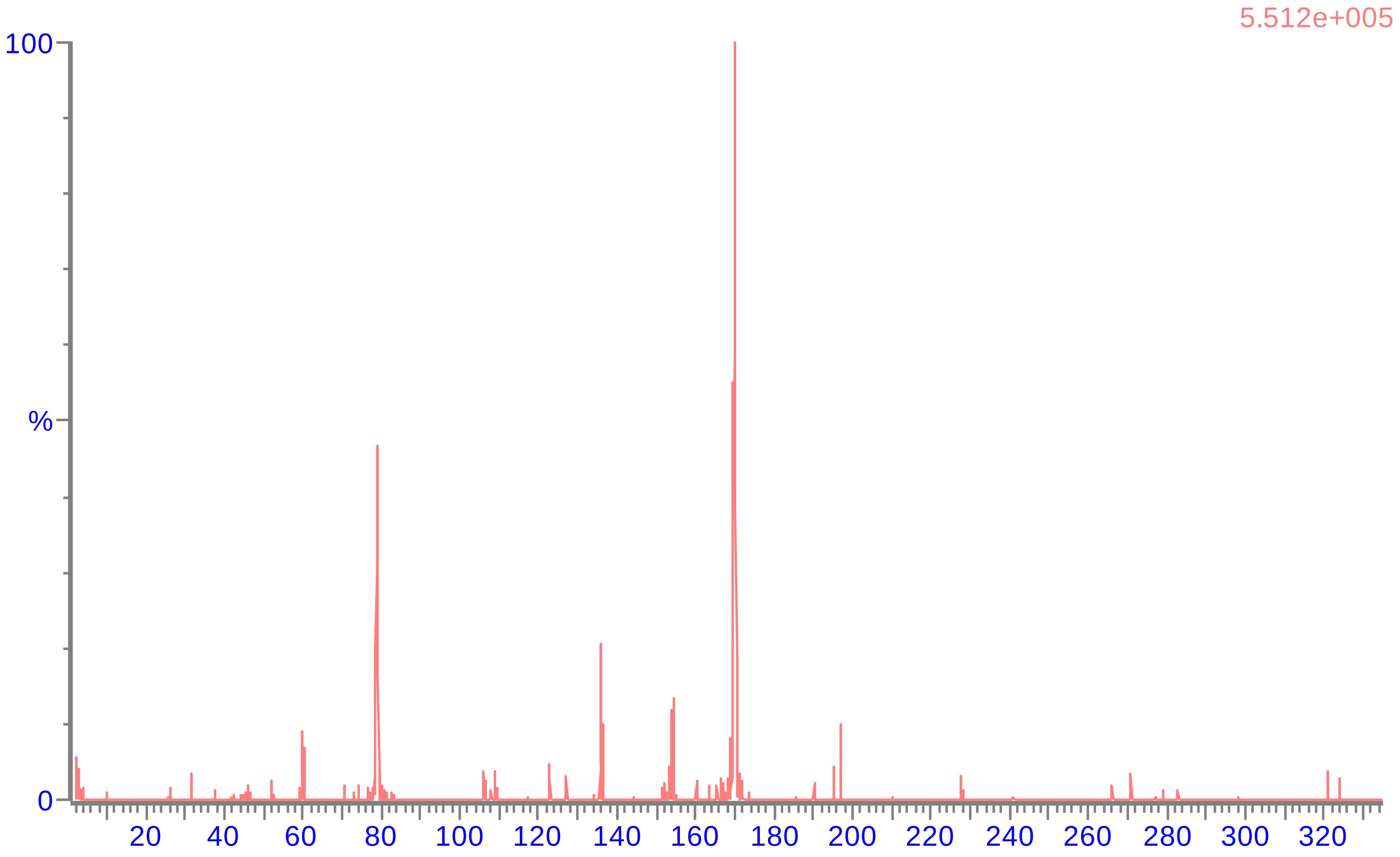

Figure 1.205: Mass spectrum for daughter fragment peak ES+, m/z 325.03 -> 78.89.

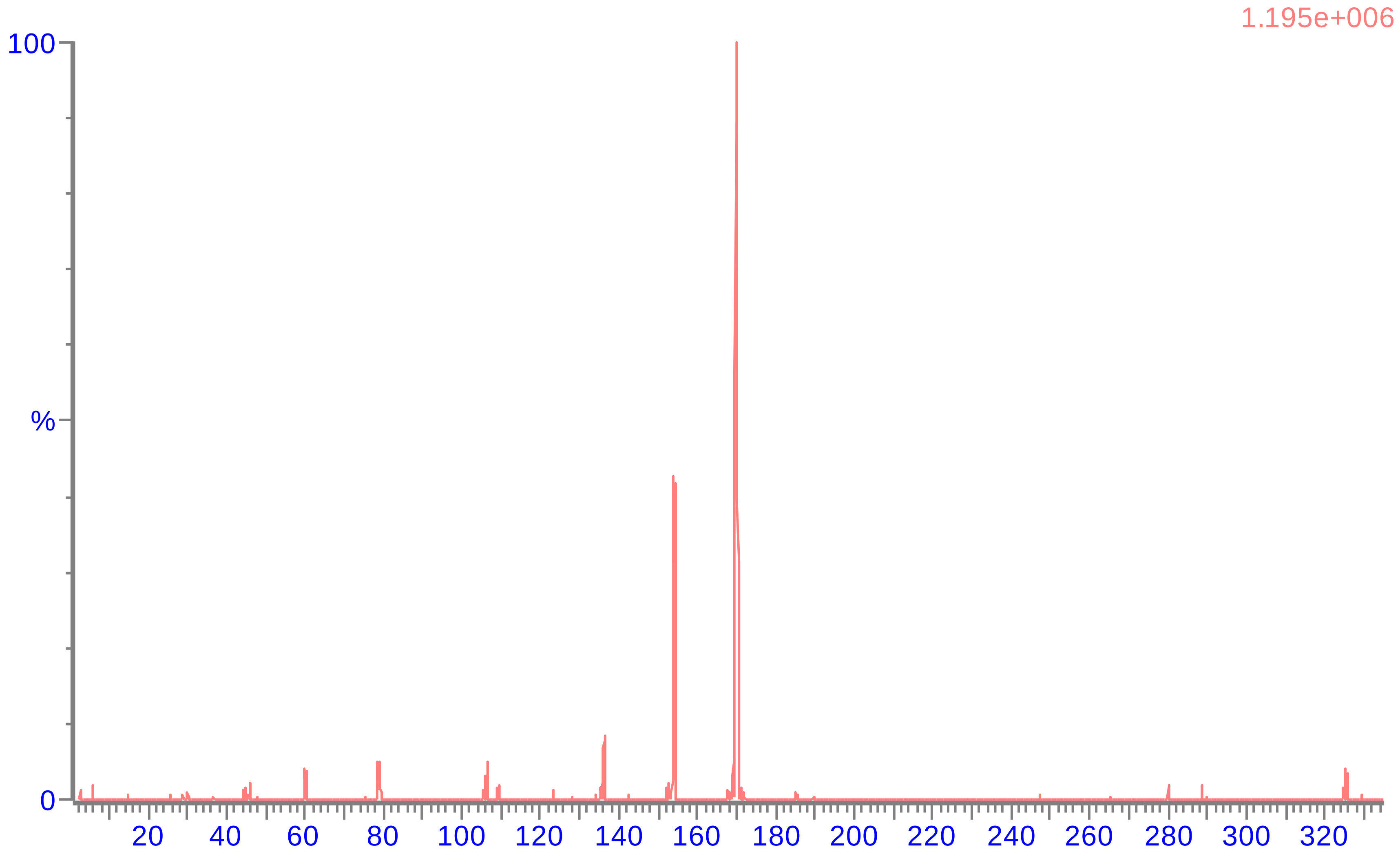

Figure 1.206: Mass spectrum for daughter fragment peak ES+, m/z 325.03 -> 136.05.

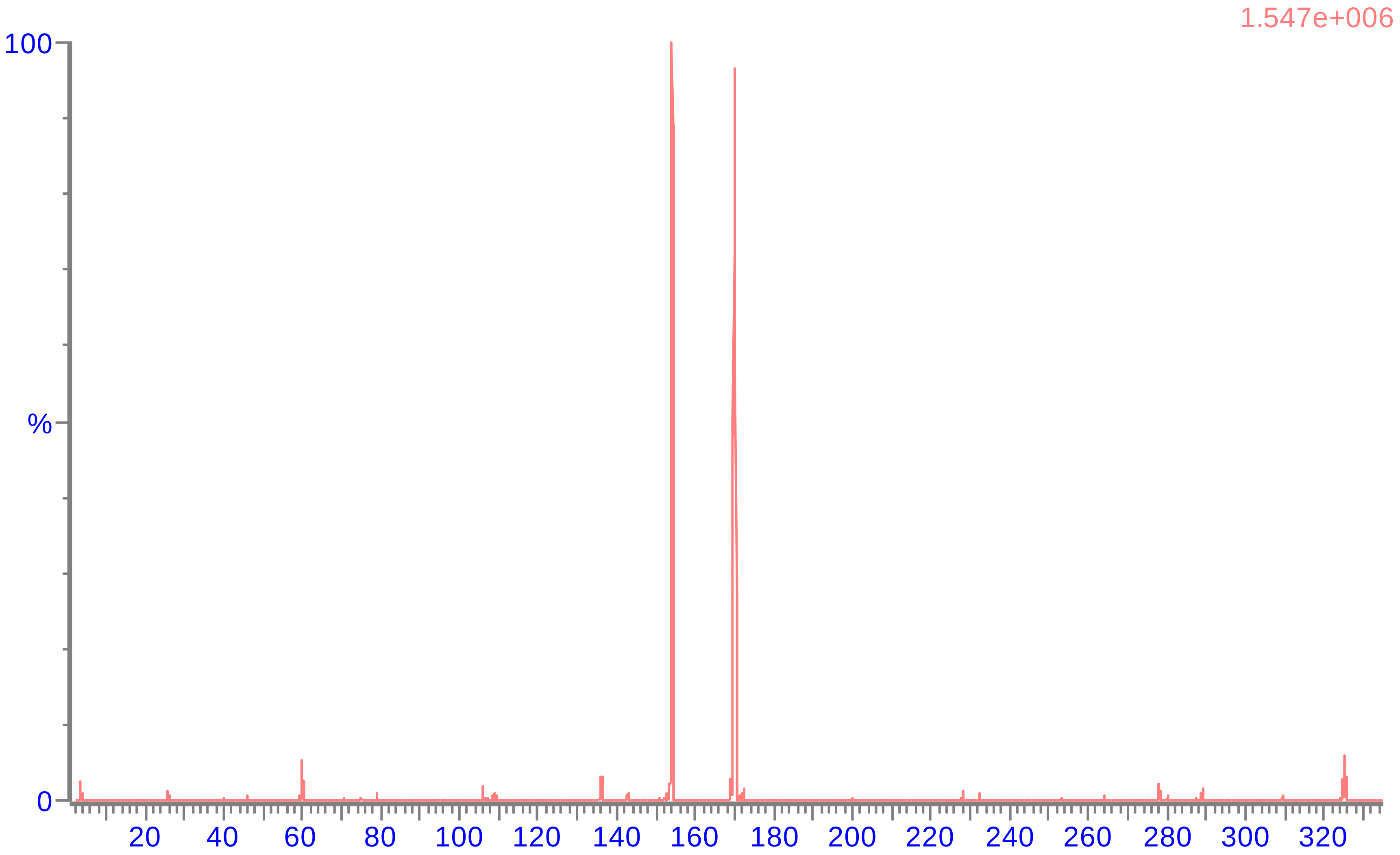

Figure 1.207: Mass spectrum for daughter fragment peak ES+, m/z 325.03 -> 60.02.

4e 4-methyl-*N*-(2-nitro-1-(2-nitrophenyl)ethyl)aniline

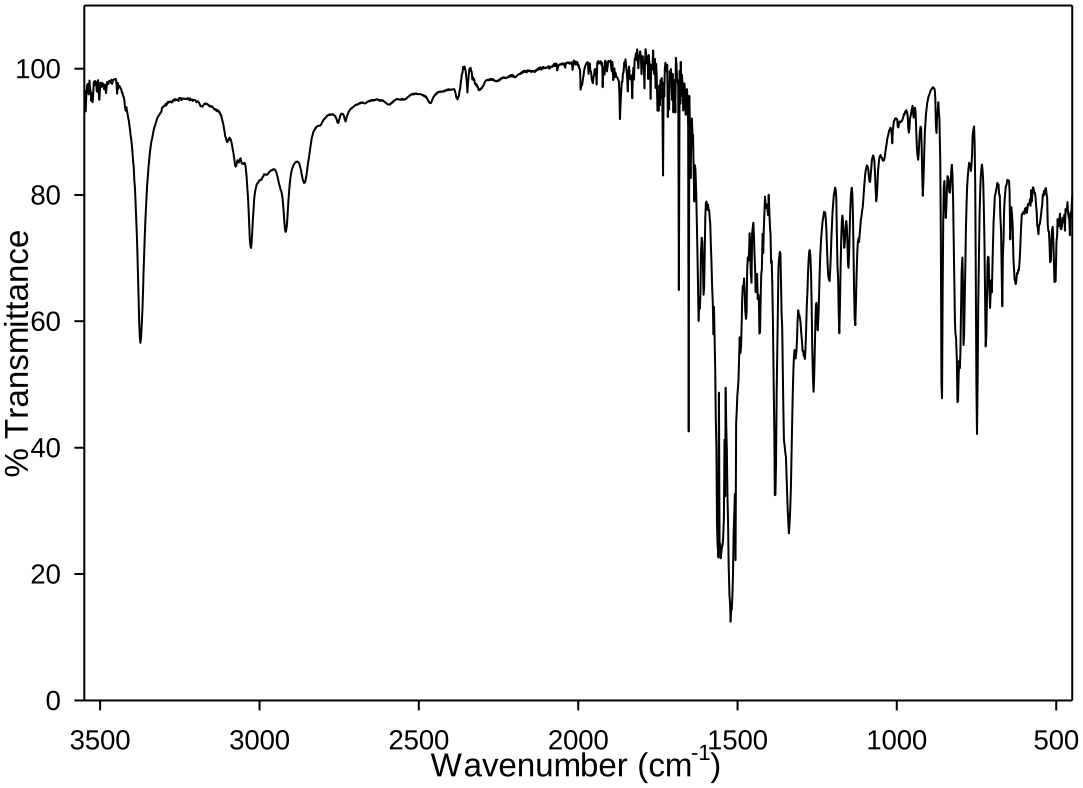

Figure 1.208: IR spectrum of **4e** 4-methyl-*N*-(2-nitro-1-(2-nitrophenyl)ethyl)aniline.

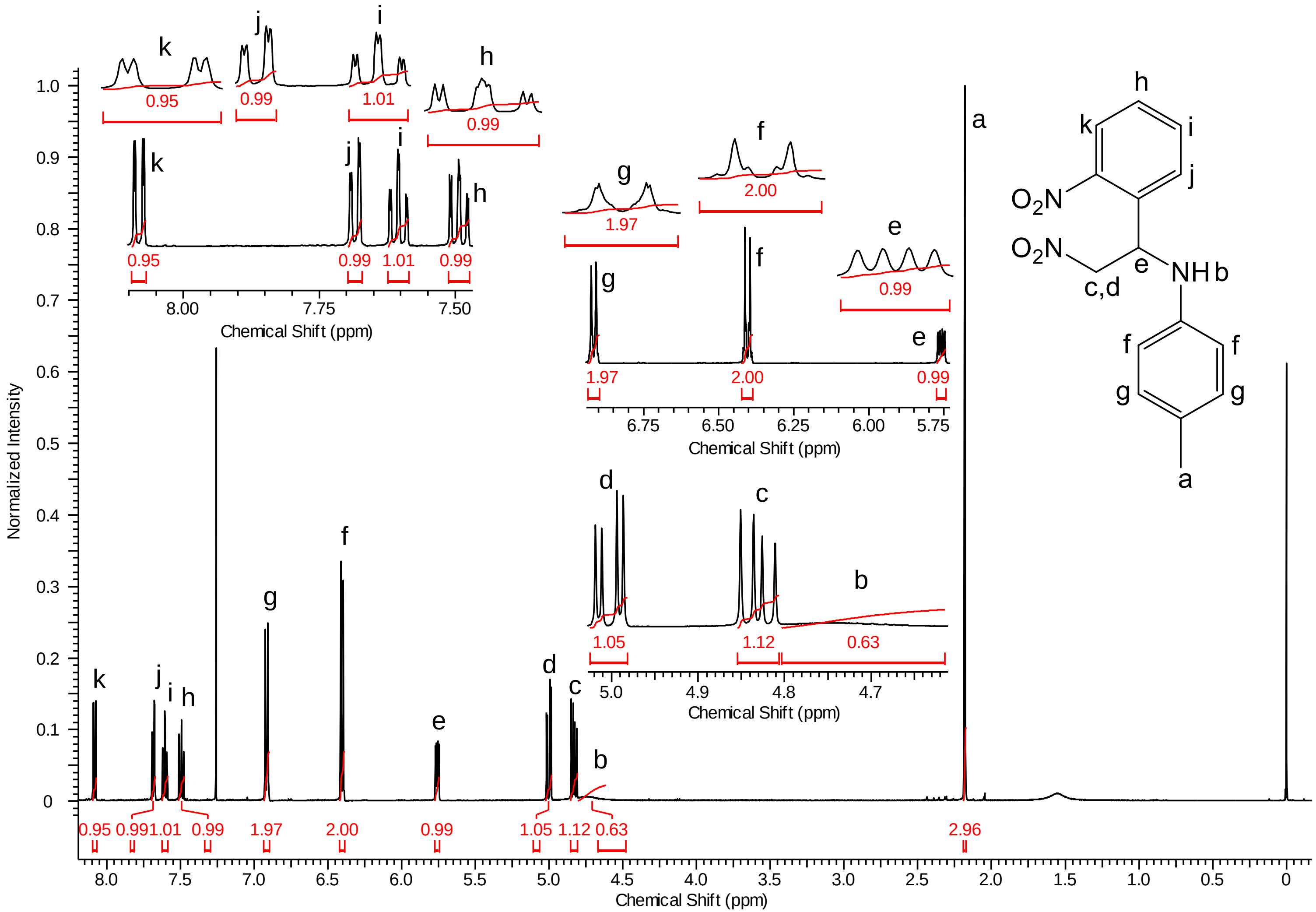

Figure 1.209: ^{1}H NMR spectrum of **4e** 4-methyl-*N*-(2-nitro-1-(2-nitrophenyl)ethyl)aniline.

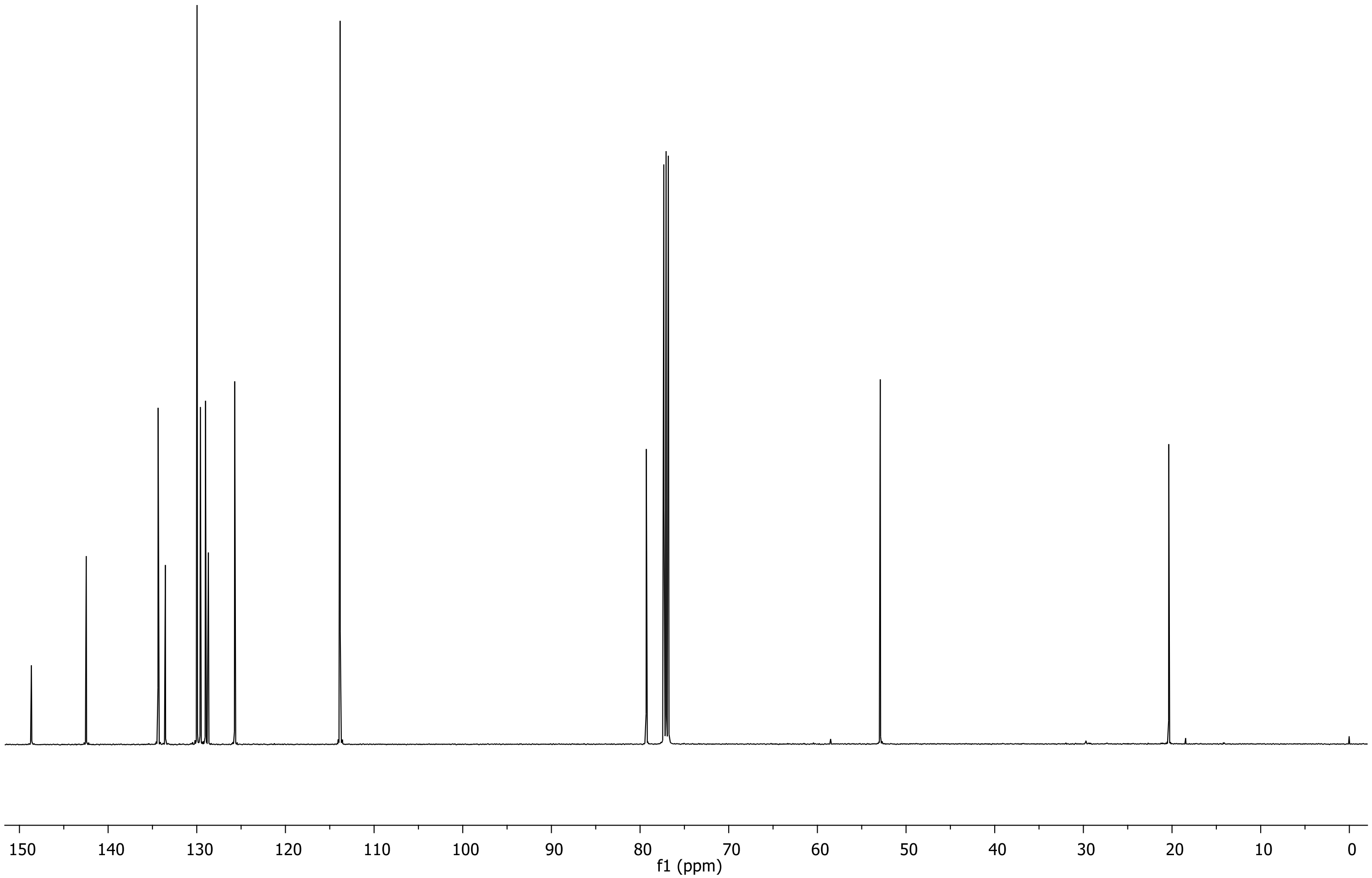

Figure 1.210: ^{13}C NMR spectrum of **4e** 4-methyl-*N*-(2-nitro-1-(2-nitrophenyl)ethyl)aniline.

Table 1.29: MS data.

Compound	Formula/Mass		Parent m/z	Cone Voltage	Daughters	Collision Energy	Ion Mode
**4e**	301	1	302.10	24	107.05	16	ES+
		2	302.10	24	241.15	14	ES+
		3	302.10	24	119.73	14	ES+
		4	302.10	24	120.05	12	ES+

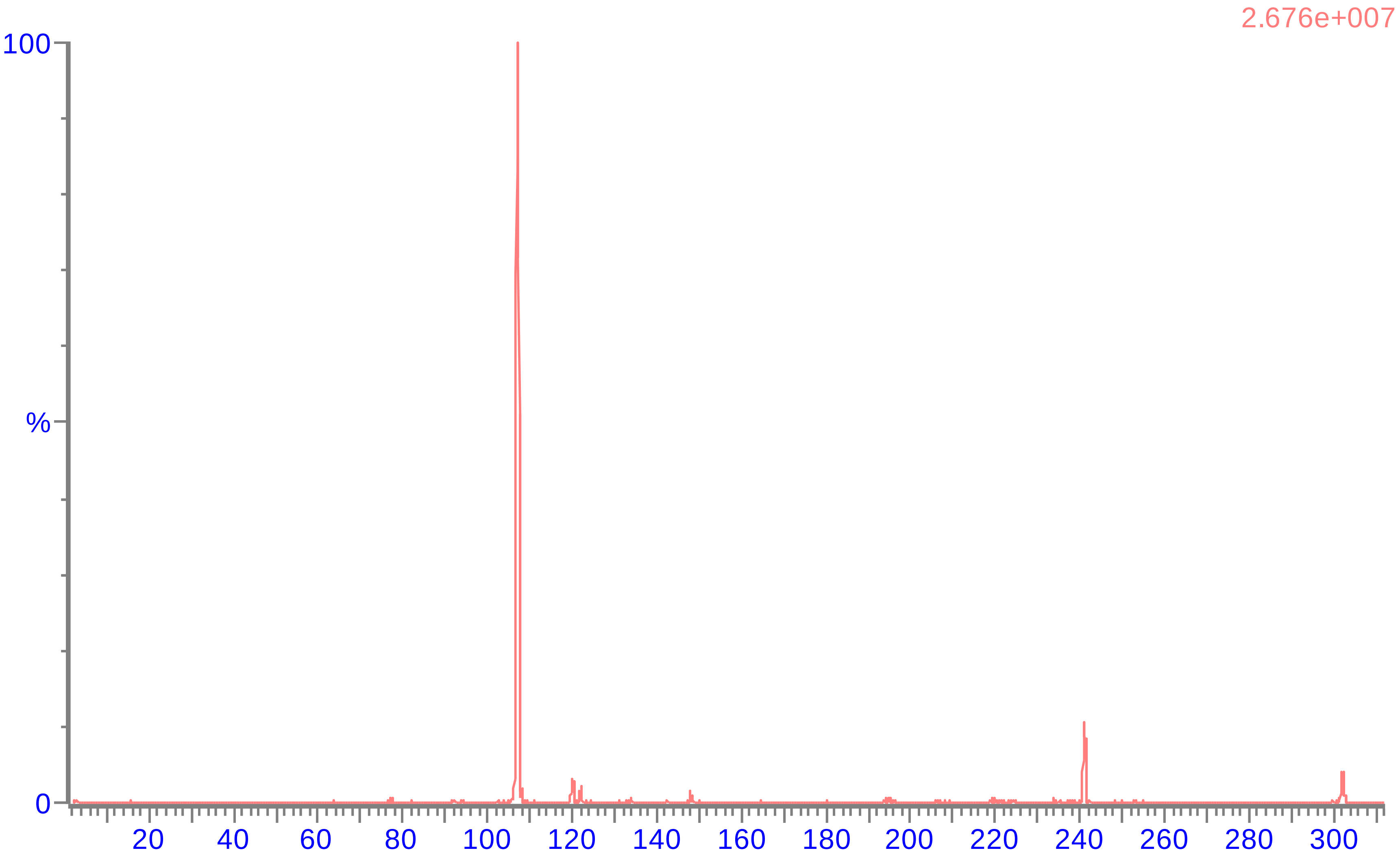

Figure 1.211: Mass spectrum for daughter fragment peak ES+, m/z 302.10 -> 107.05.

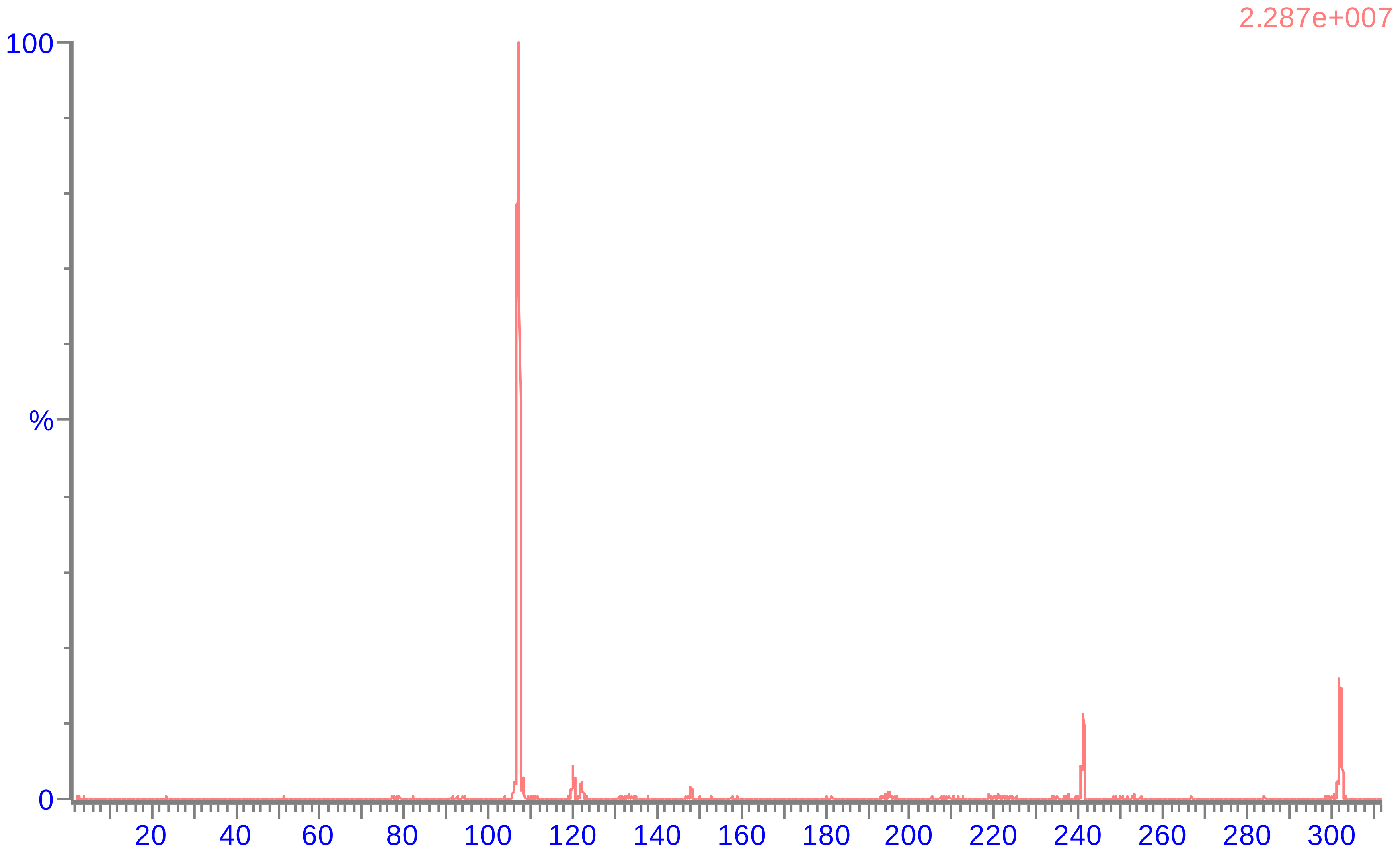

Figure 1.212: Mass spectrum for daughter fragment peak ES+, m/z 302.10 -> 241.15.

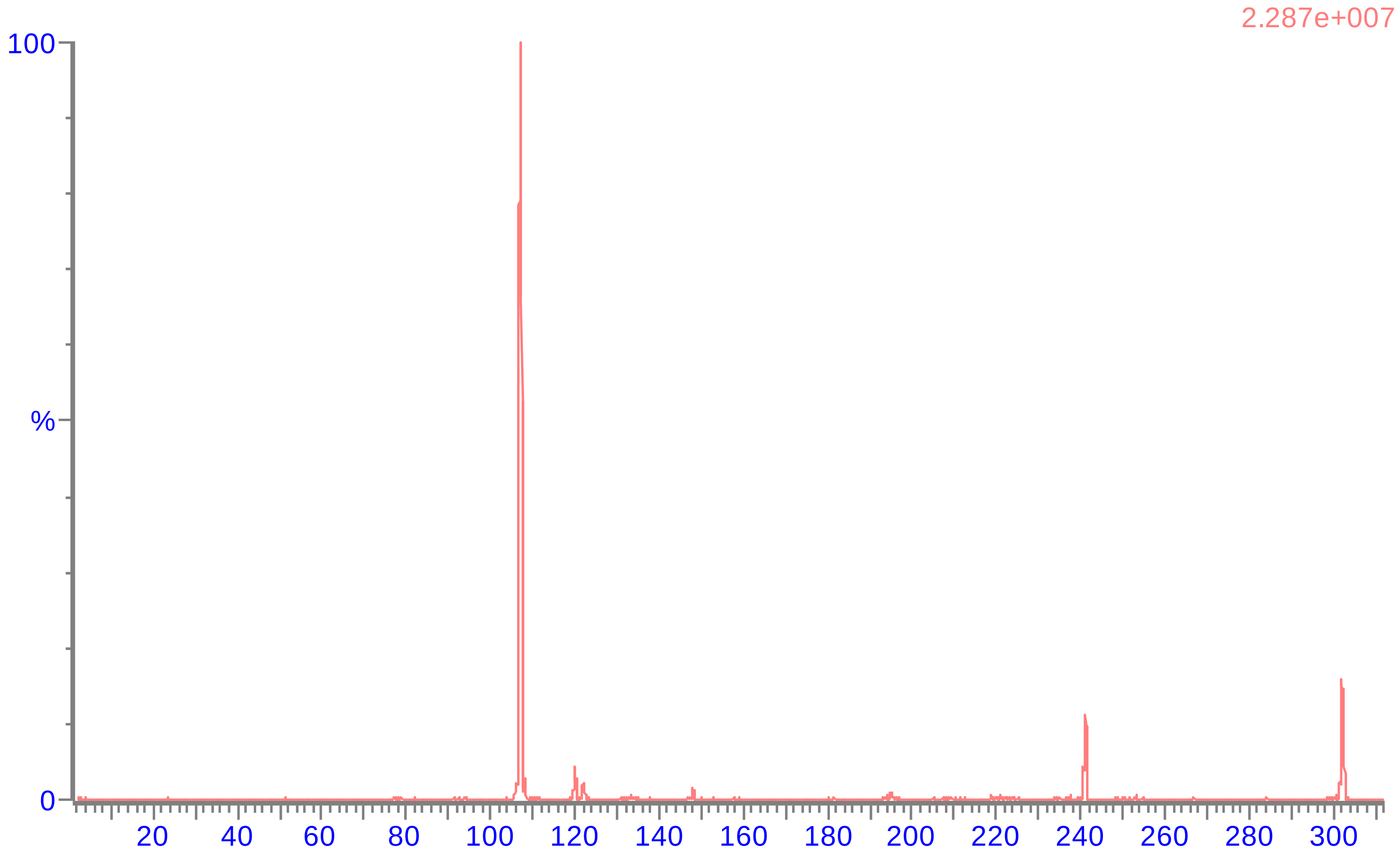

Figure 1.213: Mass spectrum for daughter fragment peak ES+, m/z 302.10 -> 119.73.

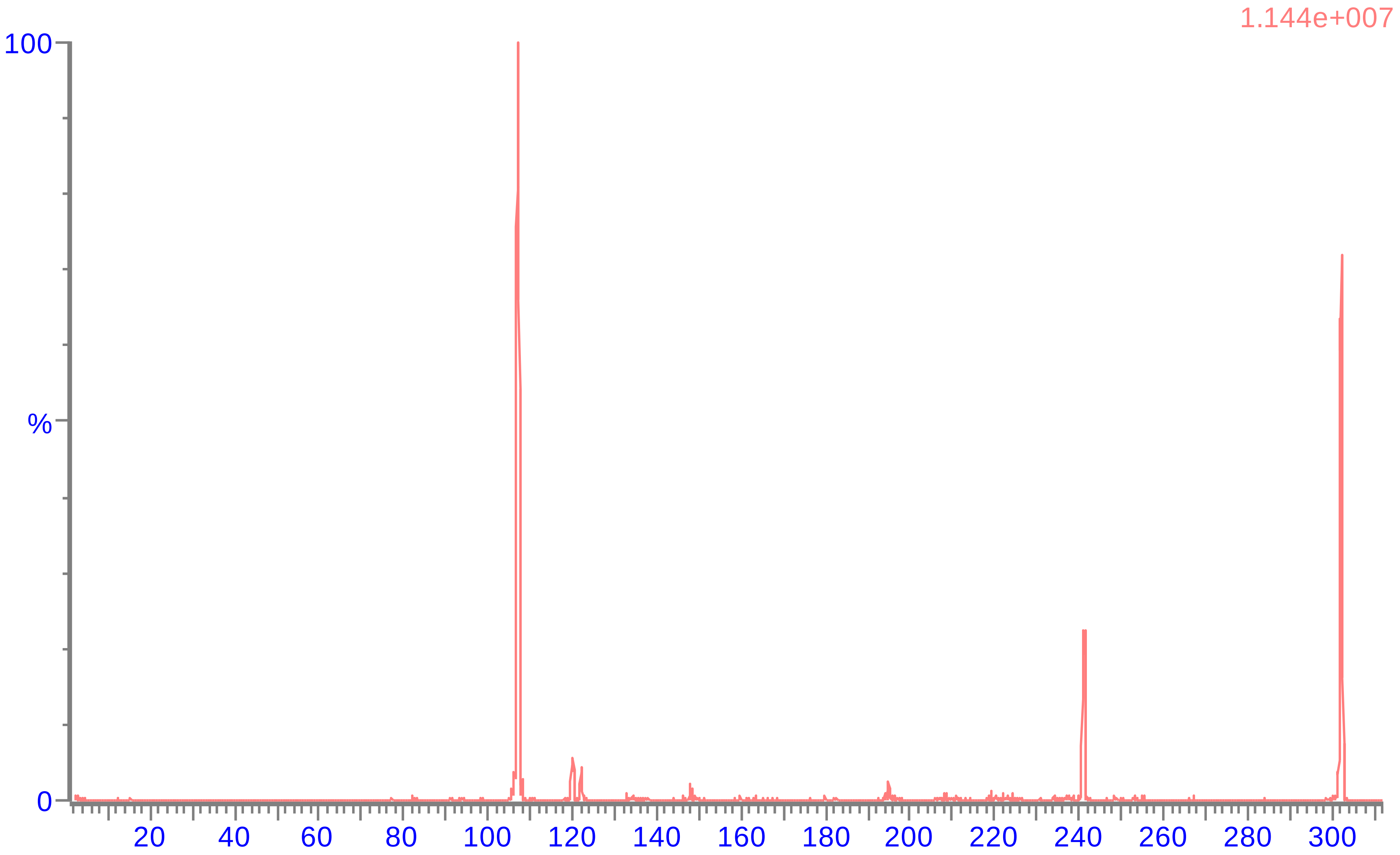

Figure 1.214: Mass spectrum for daughter fragment peak ES+, m/z 302.10 -> 120.05.

4g *N*-(1-(4-bromophenyl)-2-nitroethyl)-4-ethylaniline

Table 1.30: MS data.

Compound	Formula/Mass		Parent m/z	Cone Voltage	Daughters	Collision Energy	Ion Mode
**4g**	350	1	351.03	92	90.98	36	ES+
		2	351.03	92	64.97	66	ES+
		3	351.03	92	335.77	36	ES+
		4	351.03	92	180.26	46	ES+

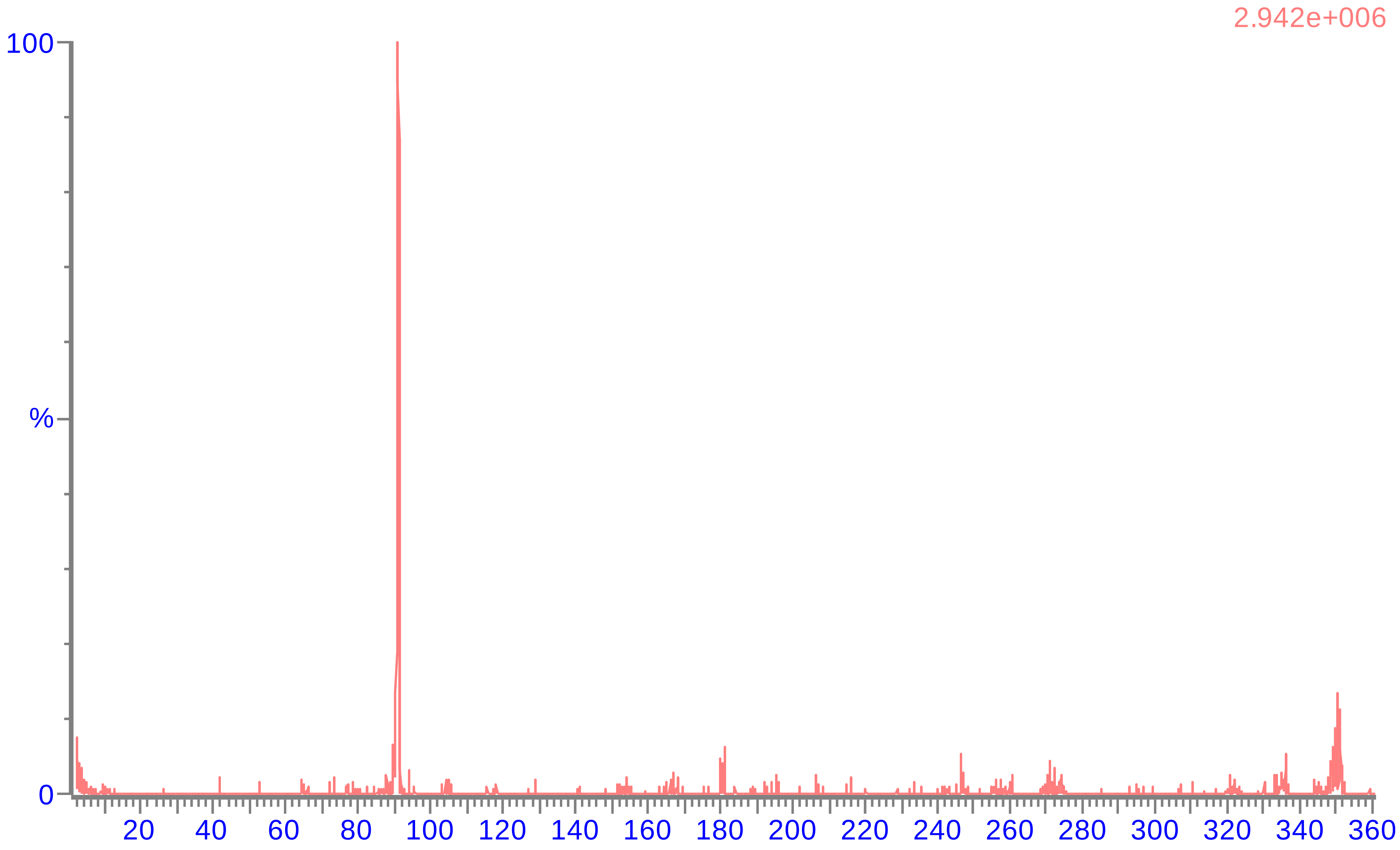

Figure 1.215: Mass spectrum for daughter fragment peak ES+, m/z 351.03 -> 90.98.

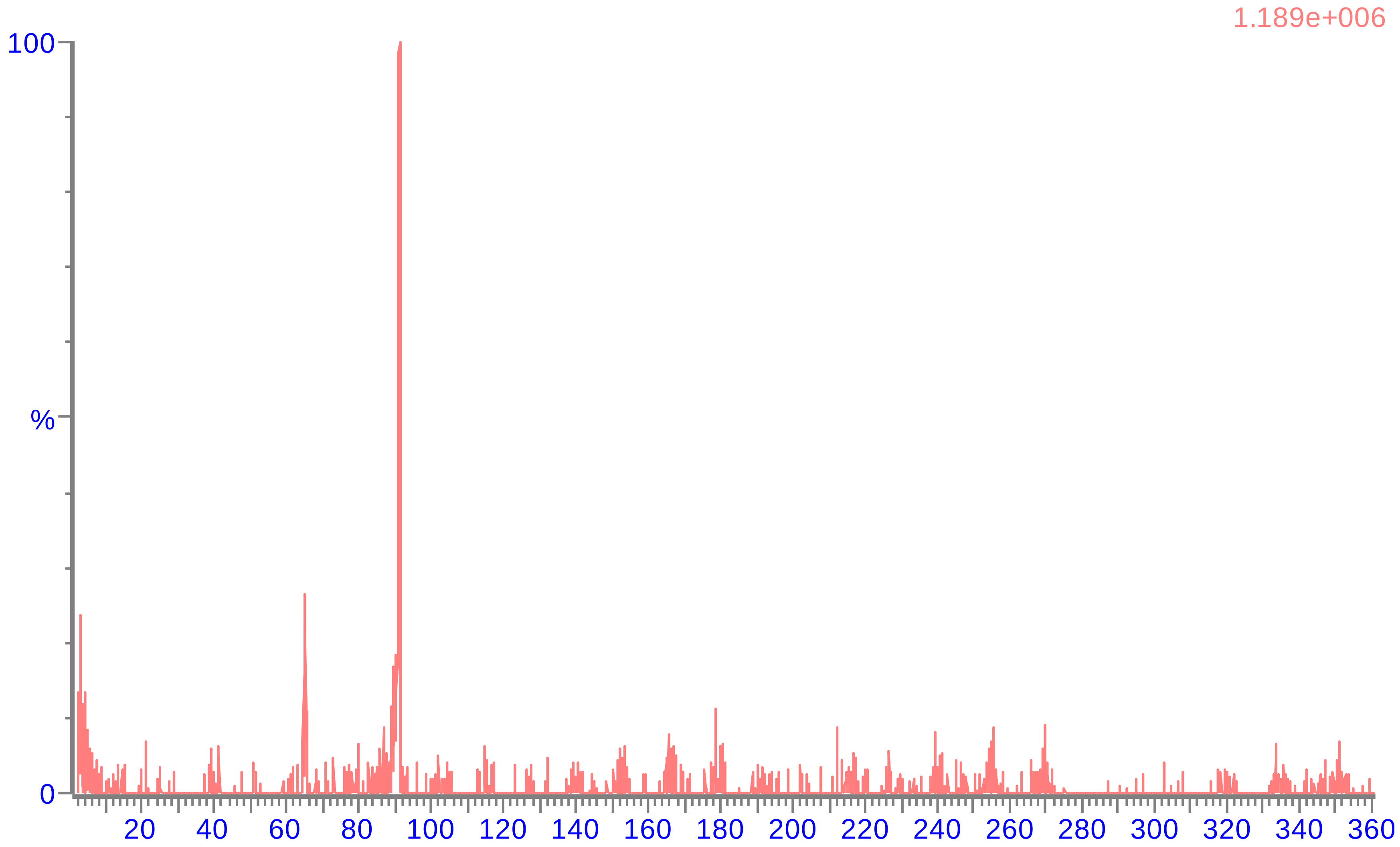

Figure 1.216: Mass spectrum for daughter fragment peak ES+, m/z 351.03 -> 64.97.

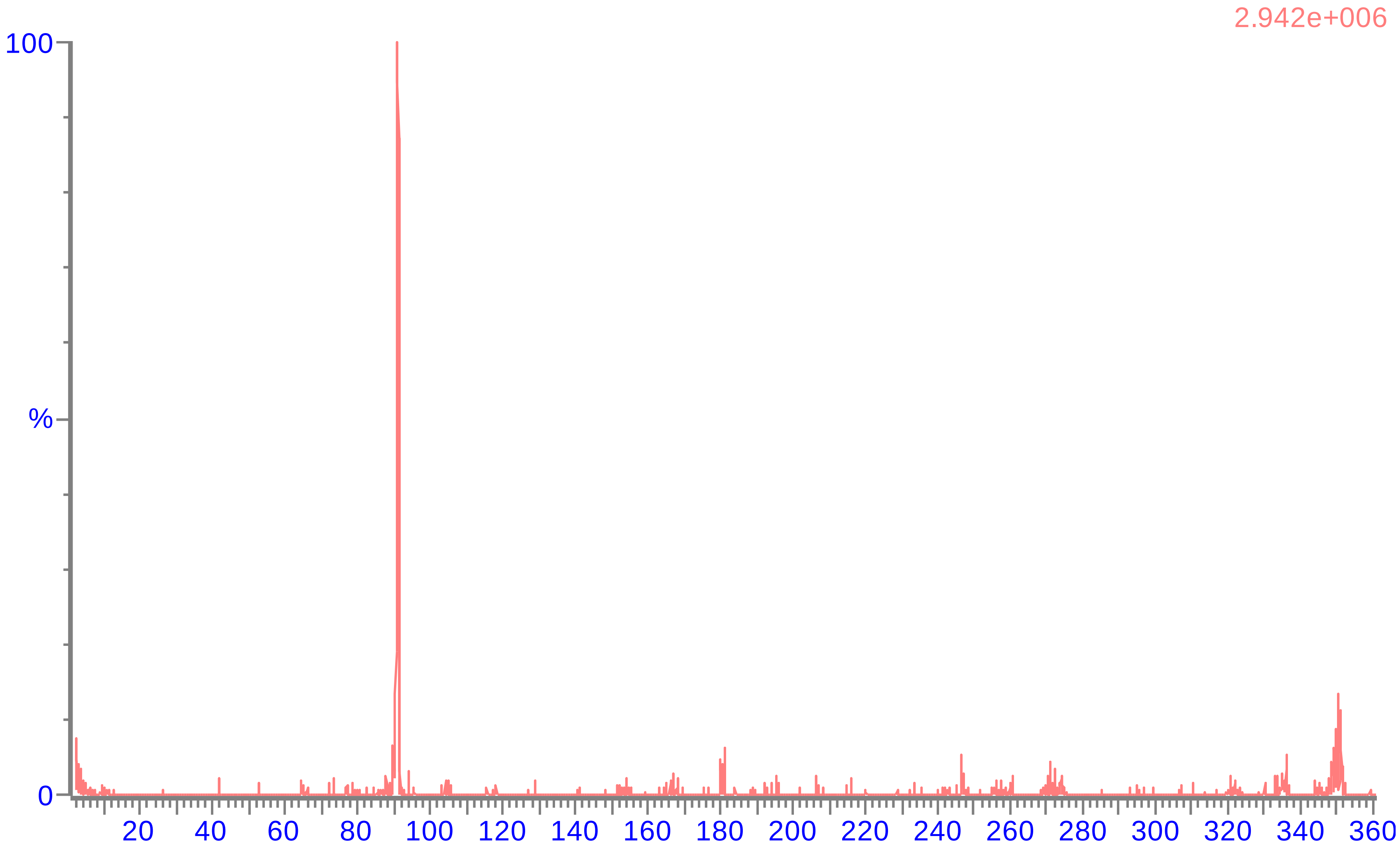

Figure 1.217: Mass spectrum for daughter fragment peak ES+, m/z 351.03 -> 335.77.

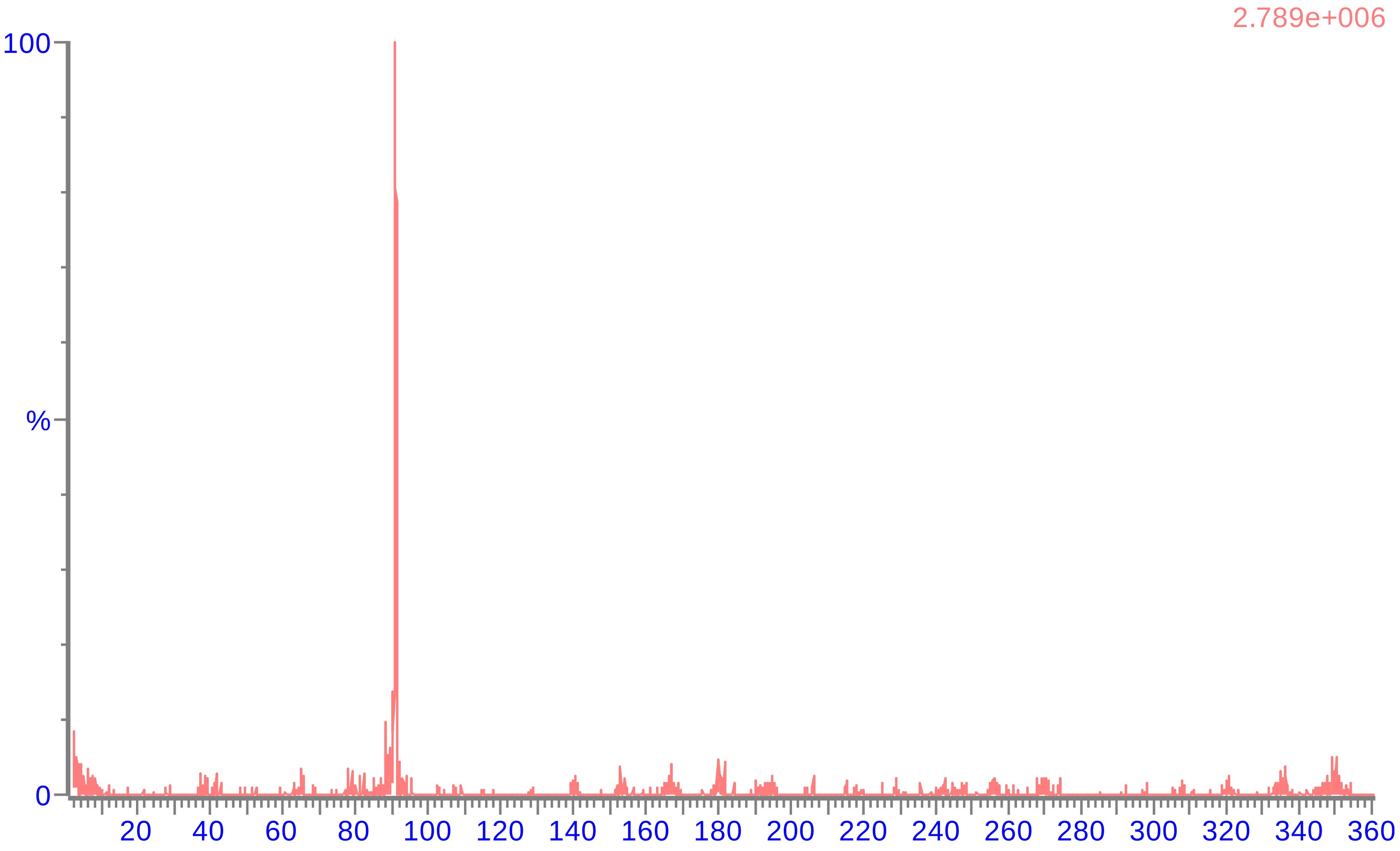

Figure 1.218: Mass spectrum for daughter fragment peak ES+, m/z 351.03 -> 180.26.

4h 4-methyl-N-(1-nitropentan-2-yl)aniline

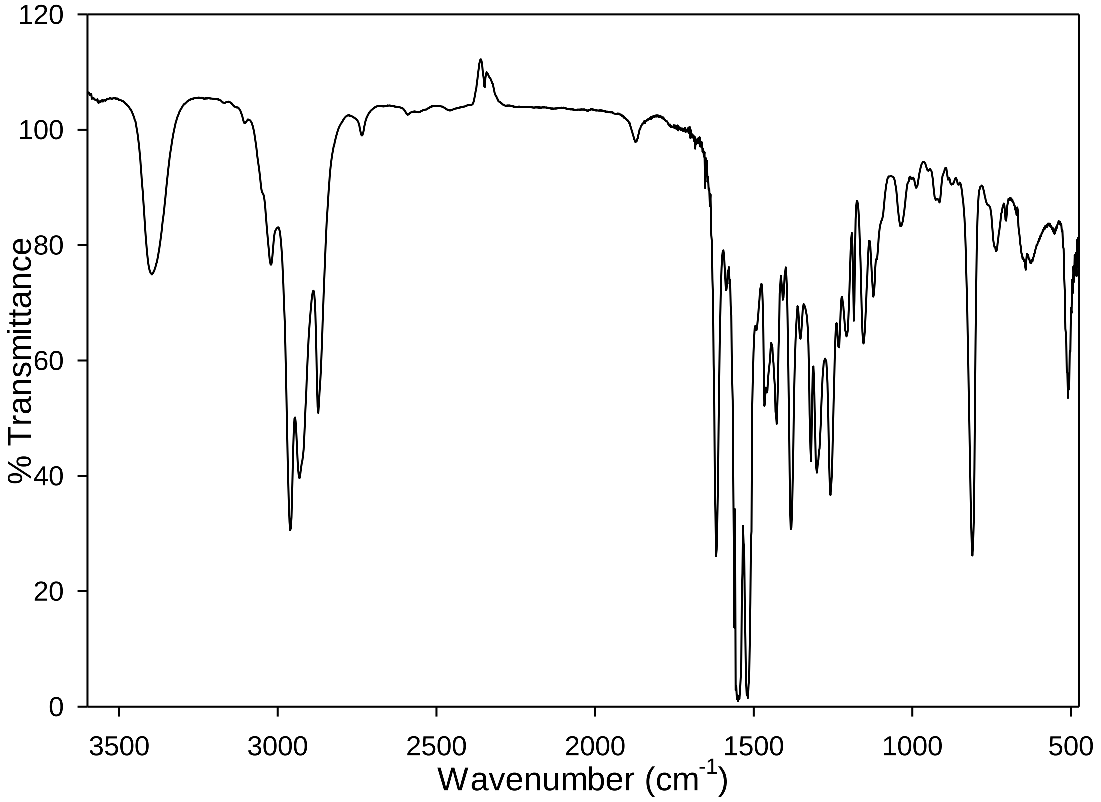

Figure 1.219: IR spectrum of **4h** 4-methyl-N-(1-nitropentan-2-yl)aniline.

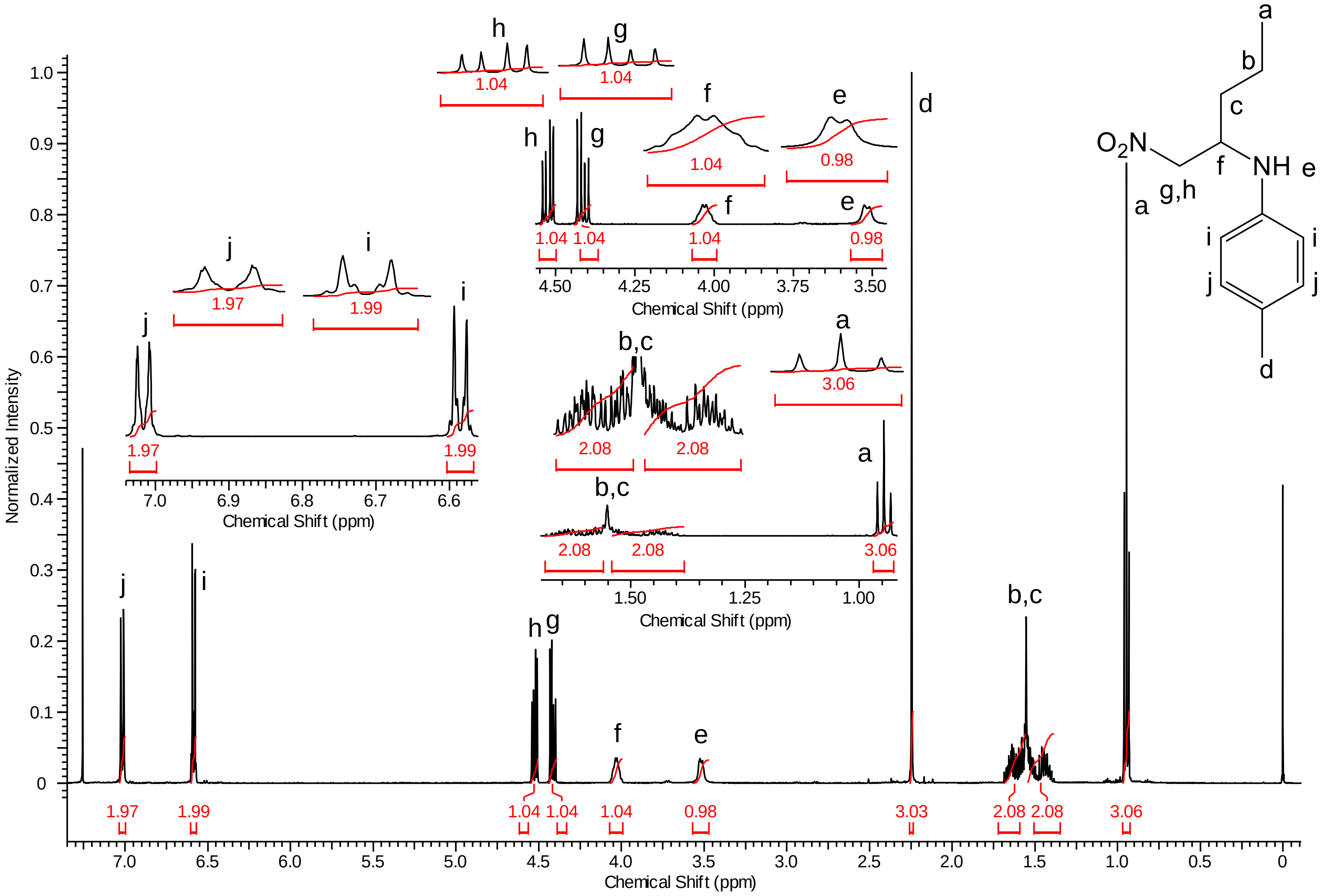

Figure 1.220: ^{1}H NMR spectrum of **4h** 4-methyl-N-(1-nitropentan-2-yl)aniline.

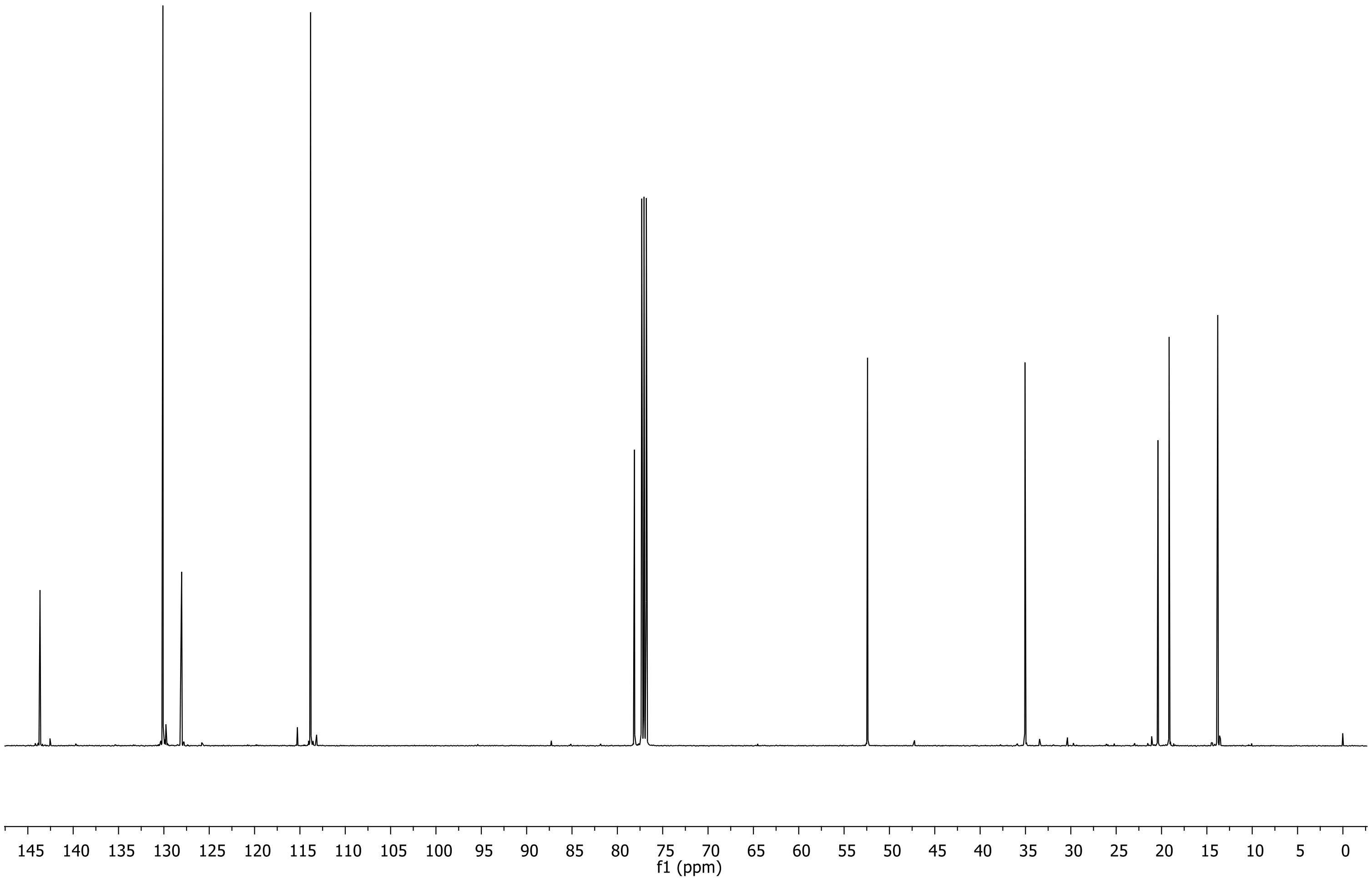

Figure 1.221: ^{13}C NMR spectrum of **4h** 4-methyl-N-(1-nitropentan-2-yl)aniline.

Table 1.31: MS data.

Compound	Formula/Mass		Parent m/z	Cone Voltage	Daughters	Collision Energy	Ion Mode
**4h**	229	1	229.07	58	199.24	26	ES+
		2	229.07	58	91.06	40	ES+
		3	229.07	58	118.00	28	ES+
		4	229.07	58	184.74	32	ES+

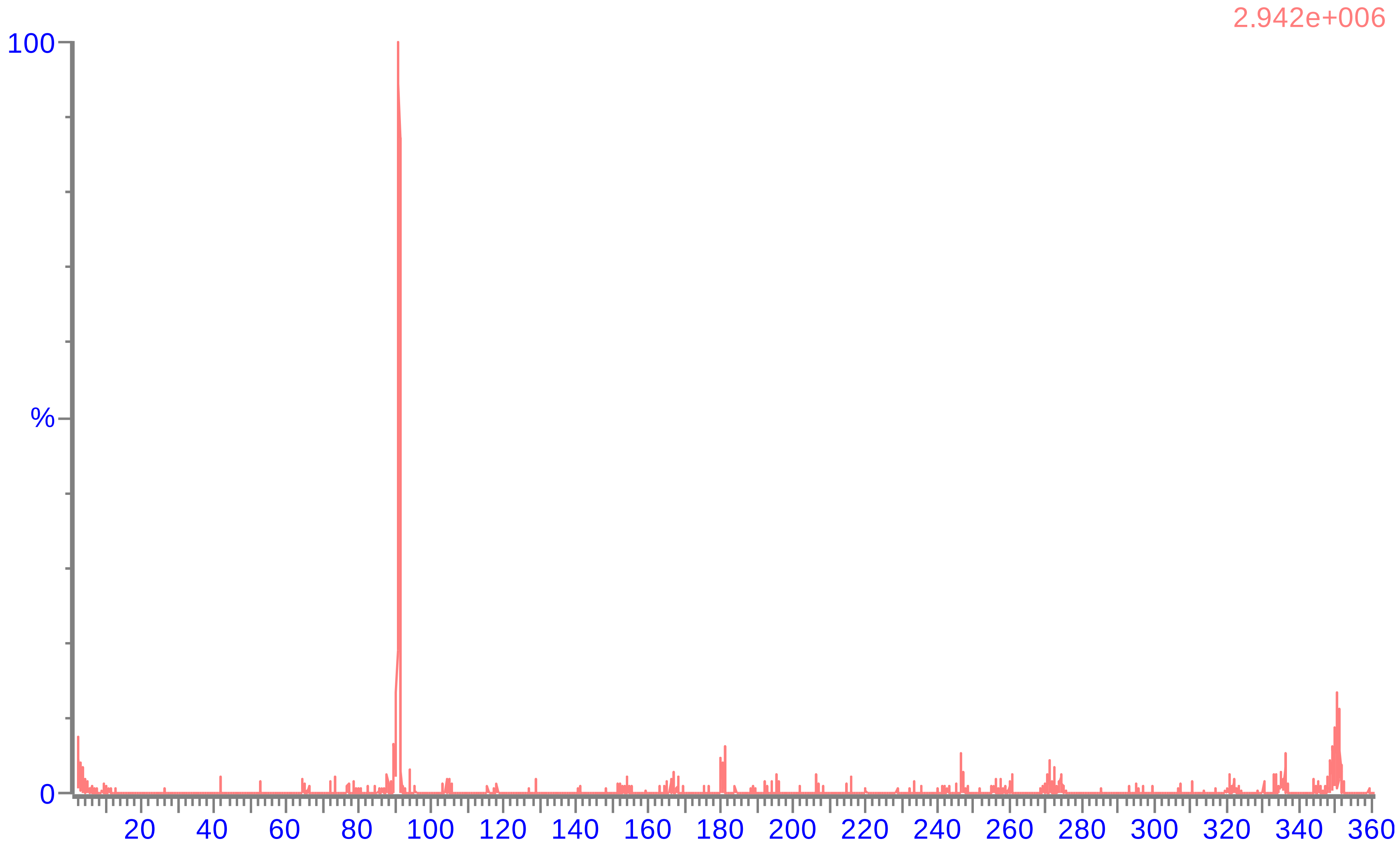

Figure 1.222: Mass spectrum for daughter fragment peak ES+, m/z 229.07 -> 199.24.

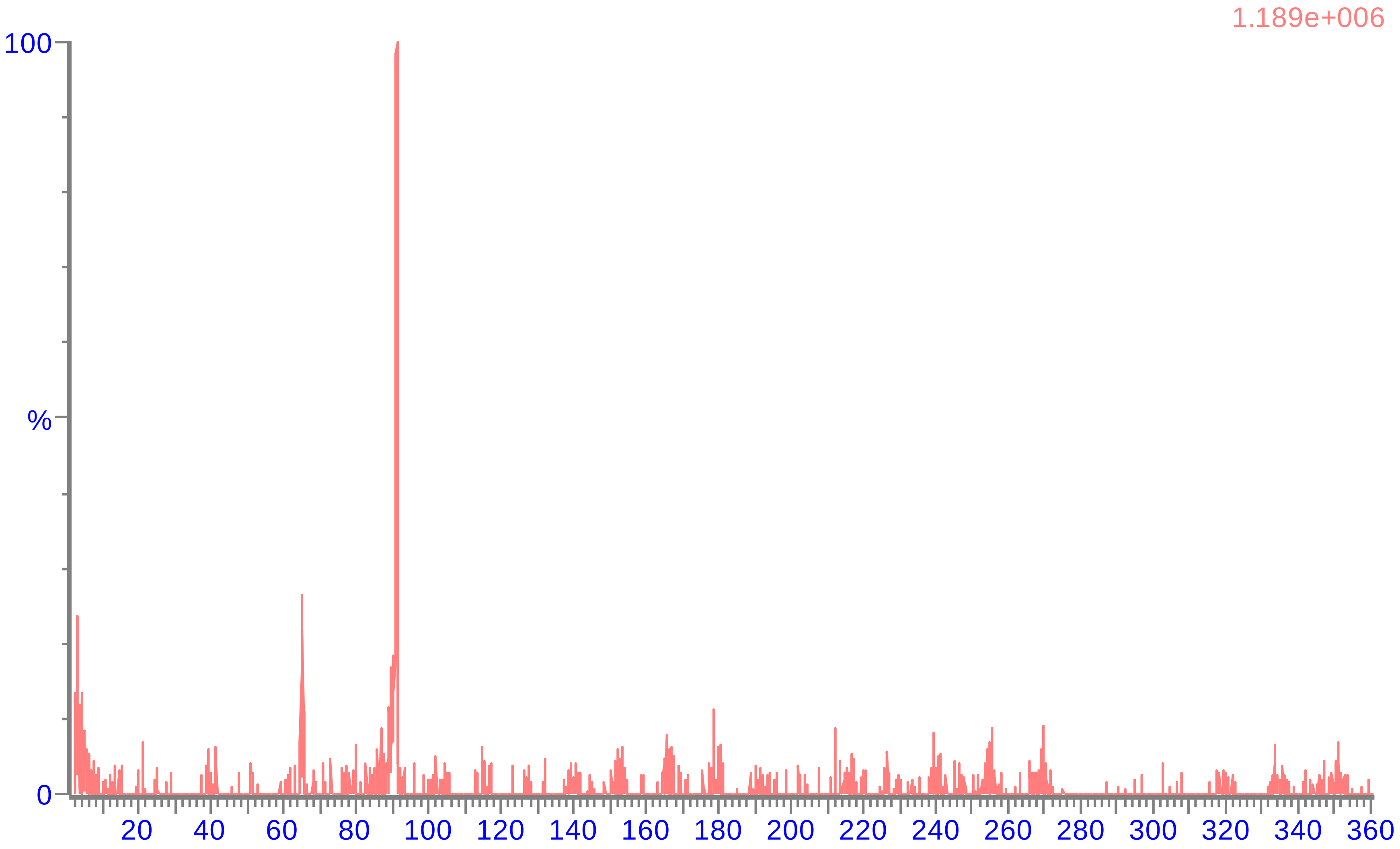

Figure 1.223: Mass spectrum for daughter fragment peak ES+, m/z 229.07 -> 64.97.

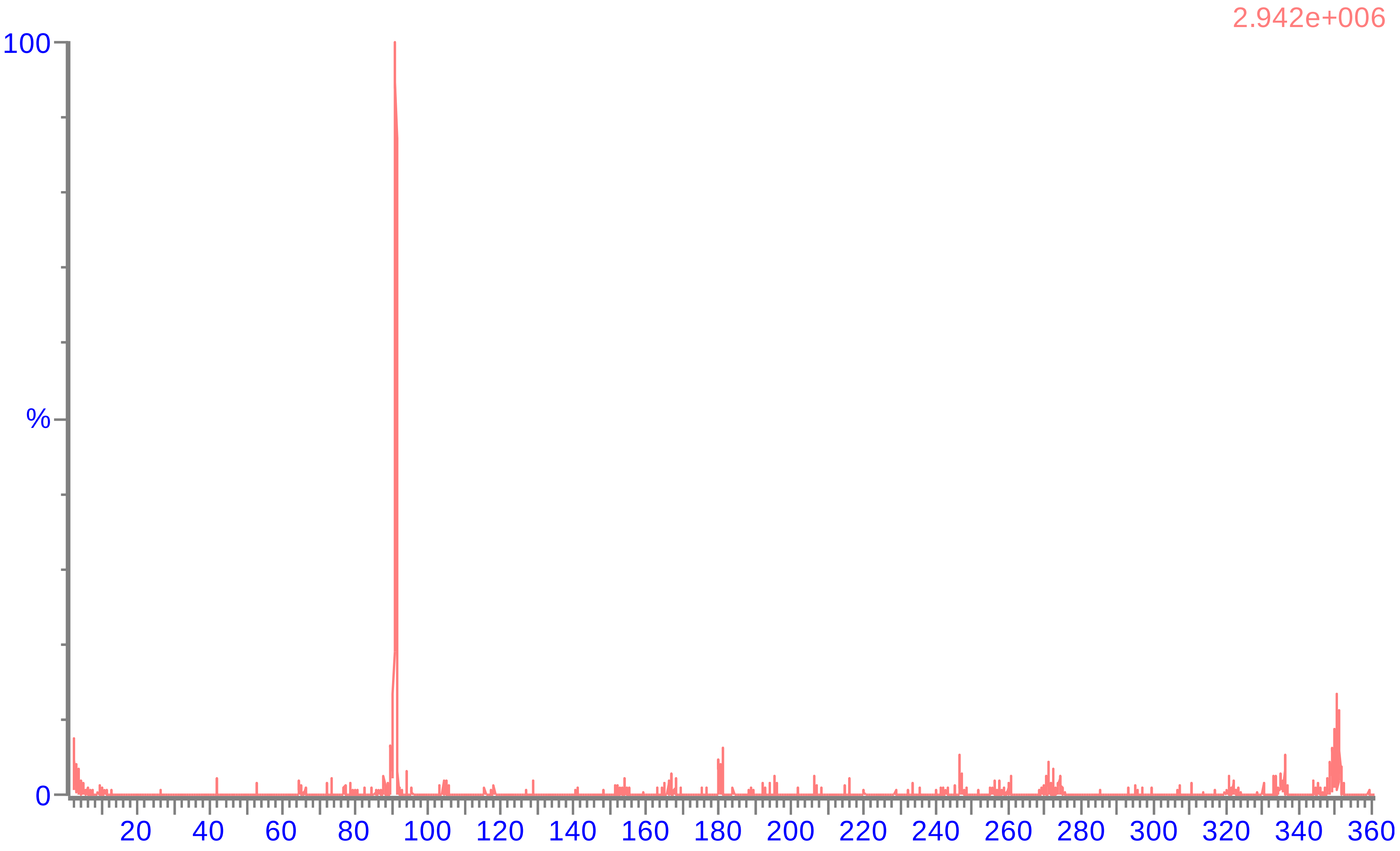

Figure 1.224: Mass spectrum for daughter fragment peak ES+, m/z 229.07 -> 335.77.

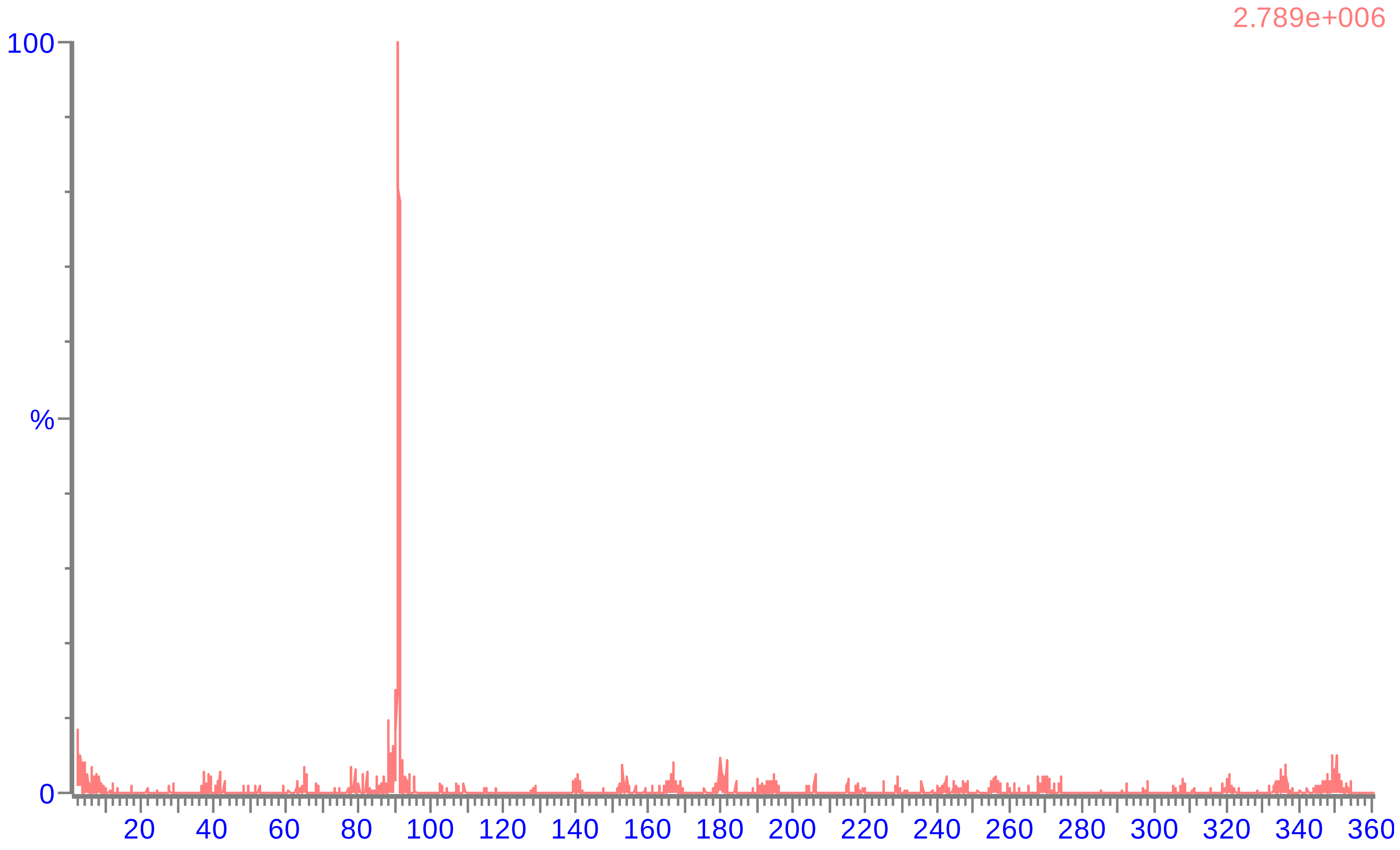

Figure 1.225: Mass spectrum for daughter fragment peak ES+, m/z 229.07 -> 180.26.

4i (*E*)-*N*-(2-nitro-1-(*p*-tolyl)ethyl)-4-(phenyldiazenyl)aniline

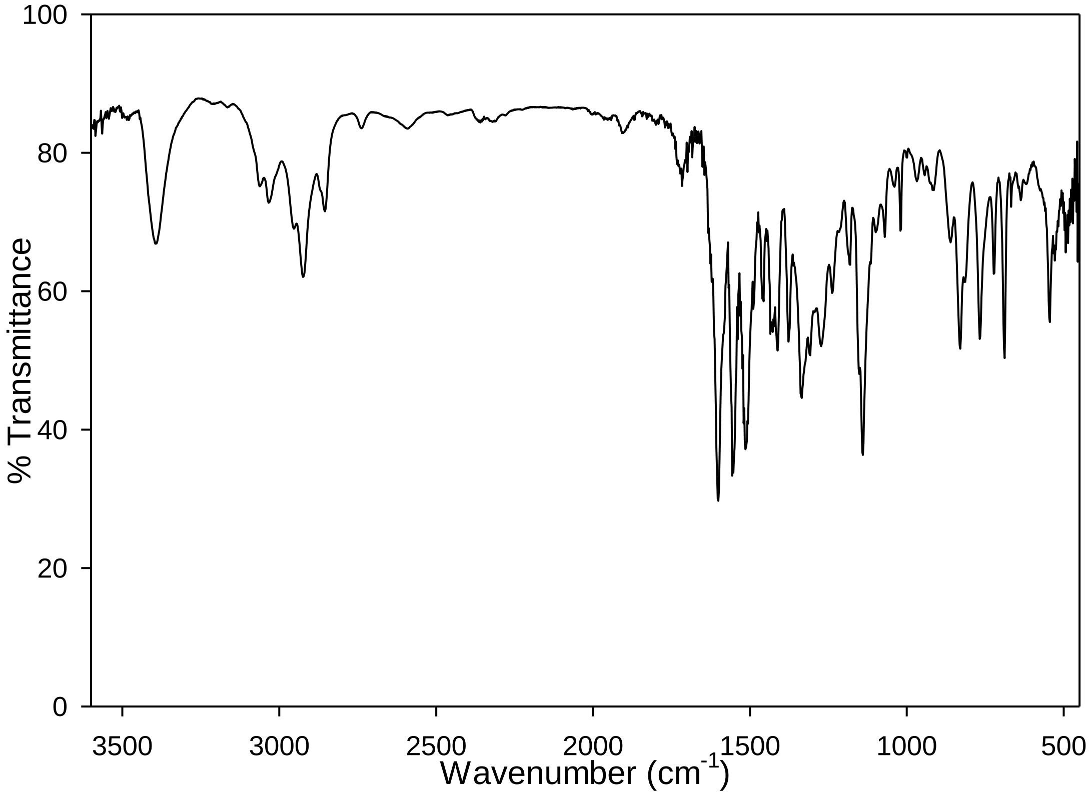

Figure 1.226: IR spectrum of **4i** (*E*)-*N*-(2-nitro-1-(*p*-tolyl)ethyl)-4-(phenyldiazenyl)aniline.

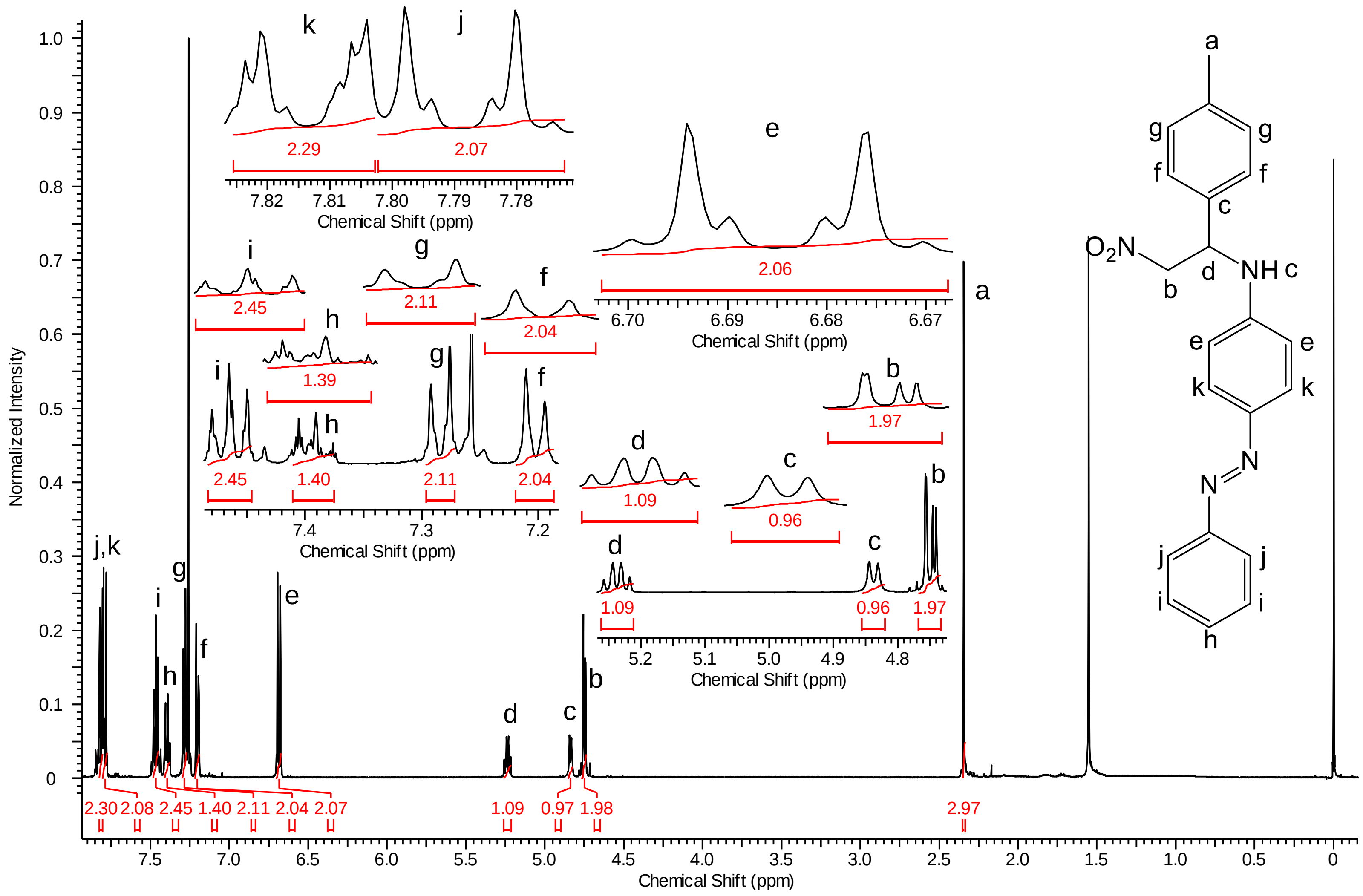

Figure 1.227: ^{1}H NMR spectrum of **4i** (*E*)-*N*-(2-nitro-1-(*p*-tolyl)ethyl)-4-(phenyldiazenyl)aniline.

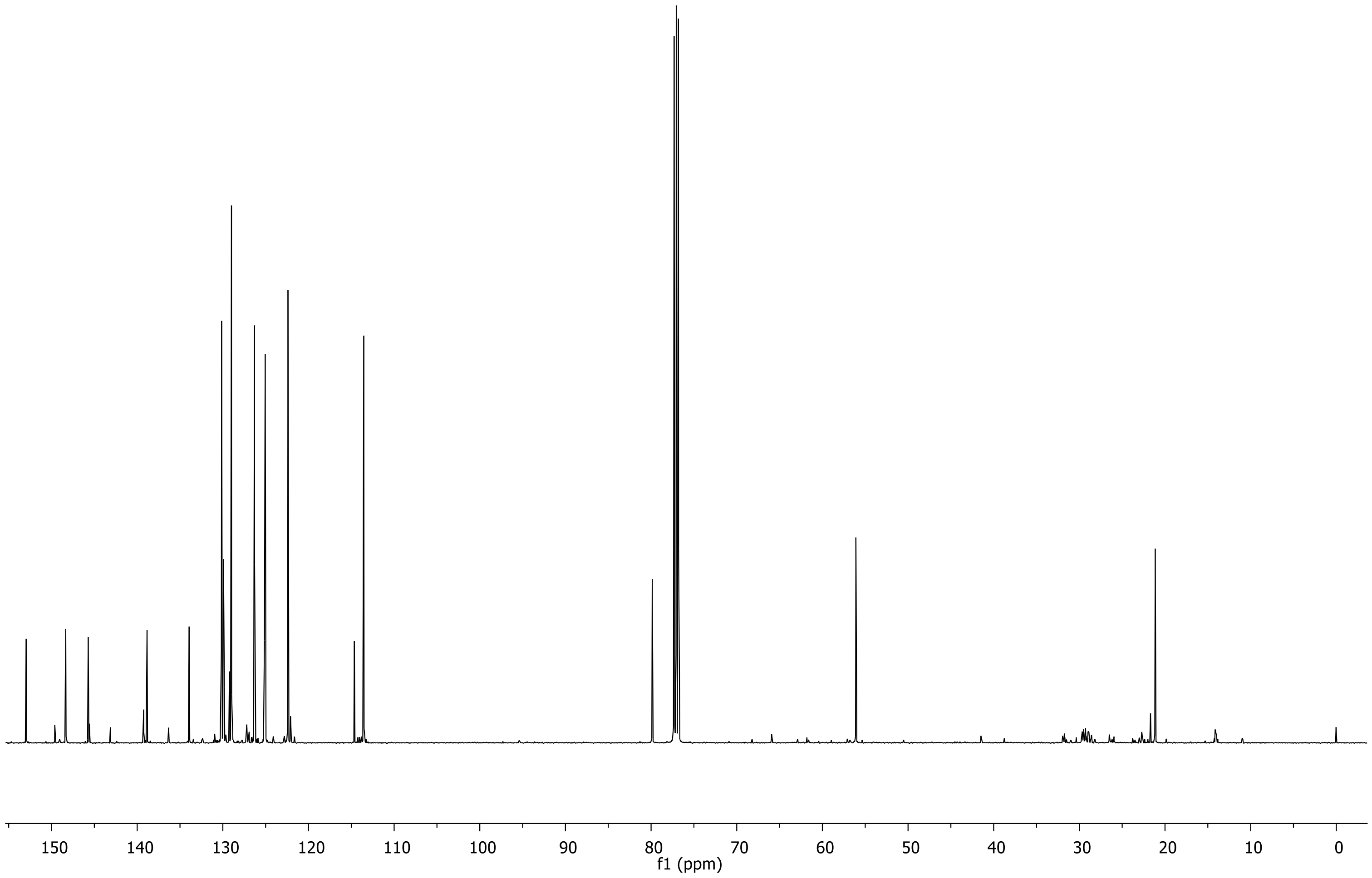

Figure 1.228: ^{13}C NMR spectrum of **4i** (*E*)-*N*-(2-nitro-1-(*p*-tolyl)ethyl)-4-(phenyldiazenyl)aniline.

Table 1.32: MS data.

Compound	Formula/Mass		Parent m/z	Cone Voltage	Daughters	Collision Energy	Ion Mode
**4i**	360.4	1	361.18	40	197.18	24	ES+
		2	361.18	40	118.04	34	ES+
		3	361.18	40	77.00	40	ES+
		4	361.18	40	91.90	50	ES+
		5	361.18	40	64.97	70	ES+

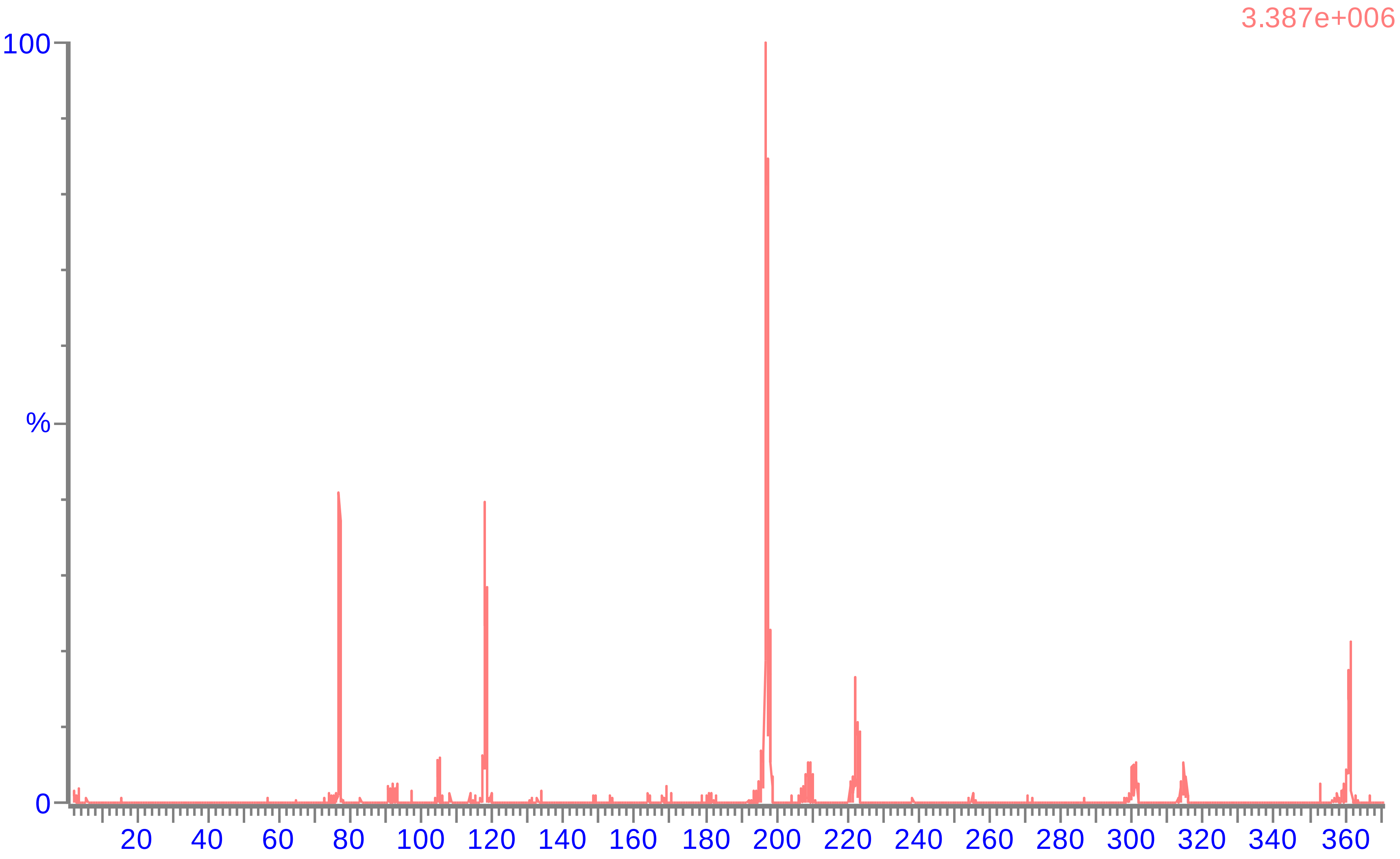

Figure 1.229: Mass spectrum for daughter fragment peak ES+, m/z 361.18 -> 197.18.

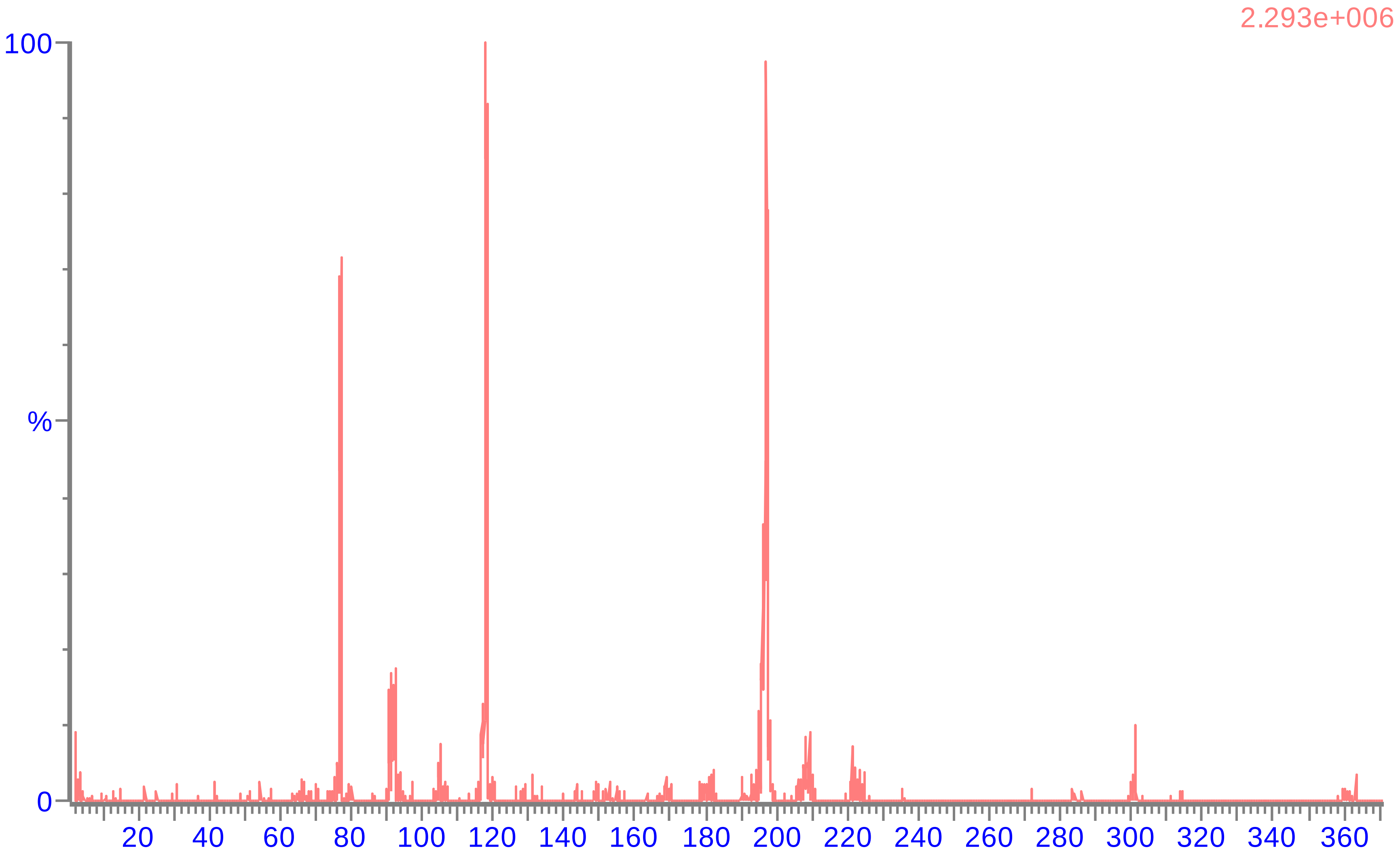

Figure 1.230: Mass spectrum for daughter fragment peak ES+, m/z 361.18 -> 118.04.

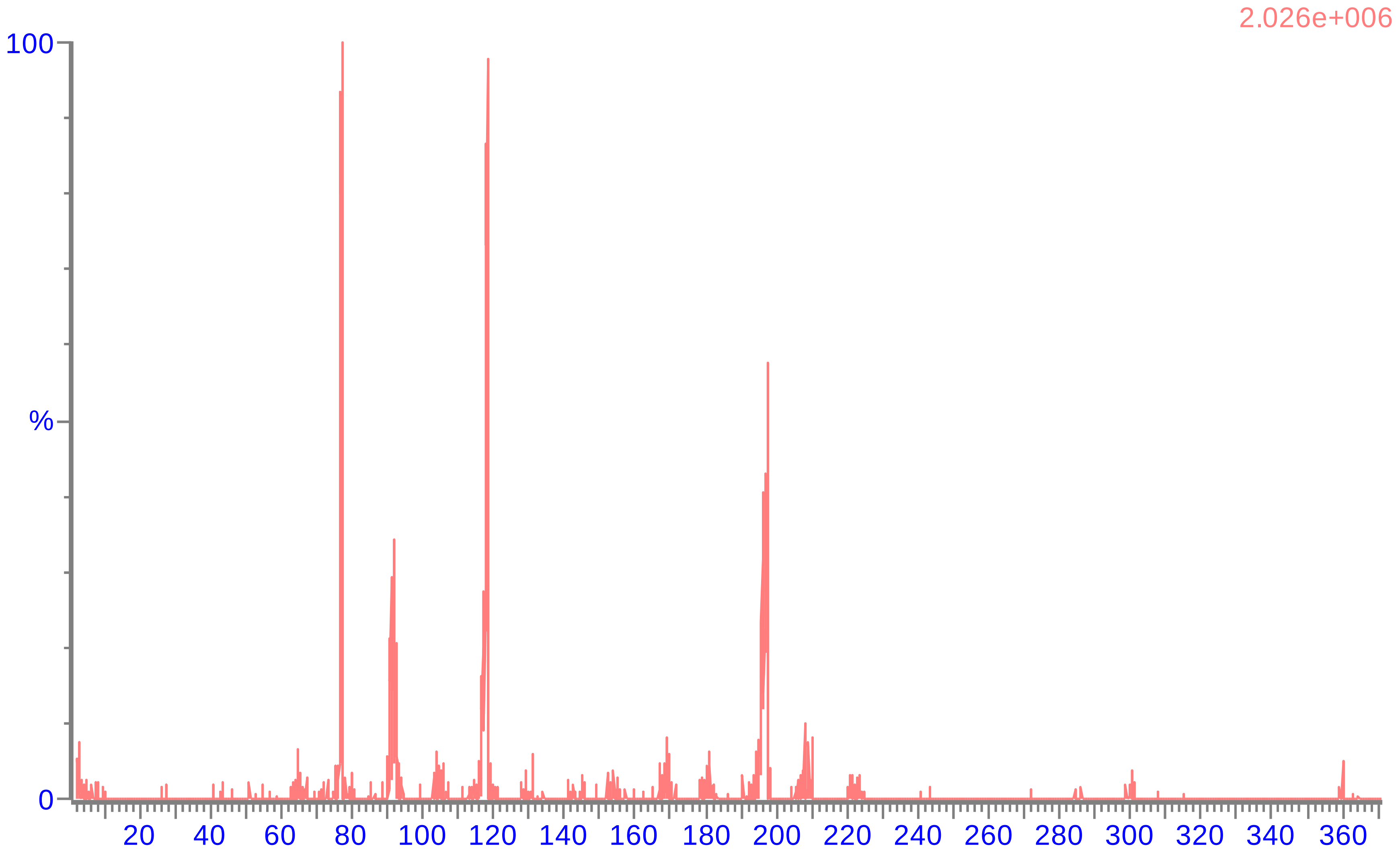

Figure 1.231: Mass spectrum for daughter fragment peak ES+, m/z 361.18 -> 77.00.

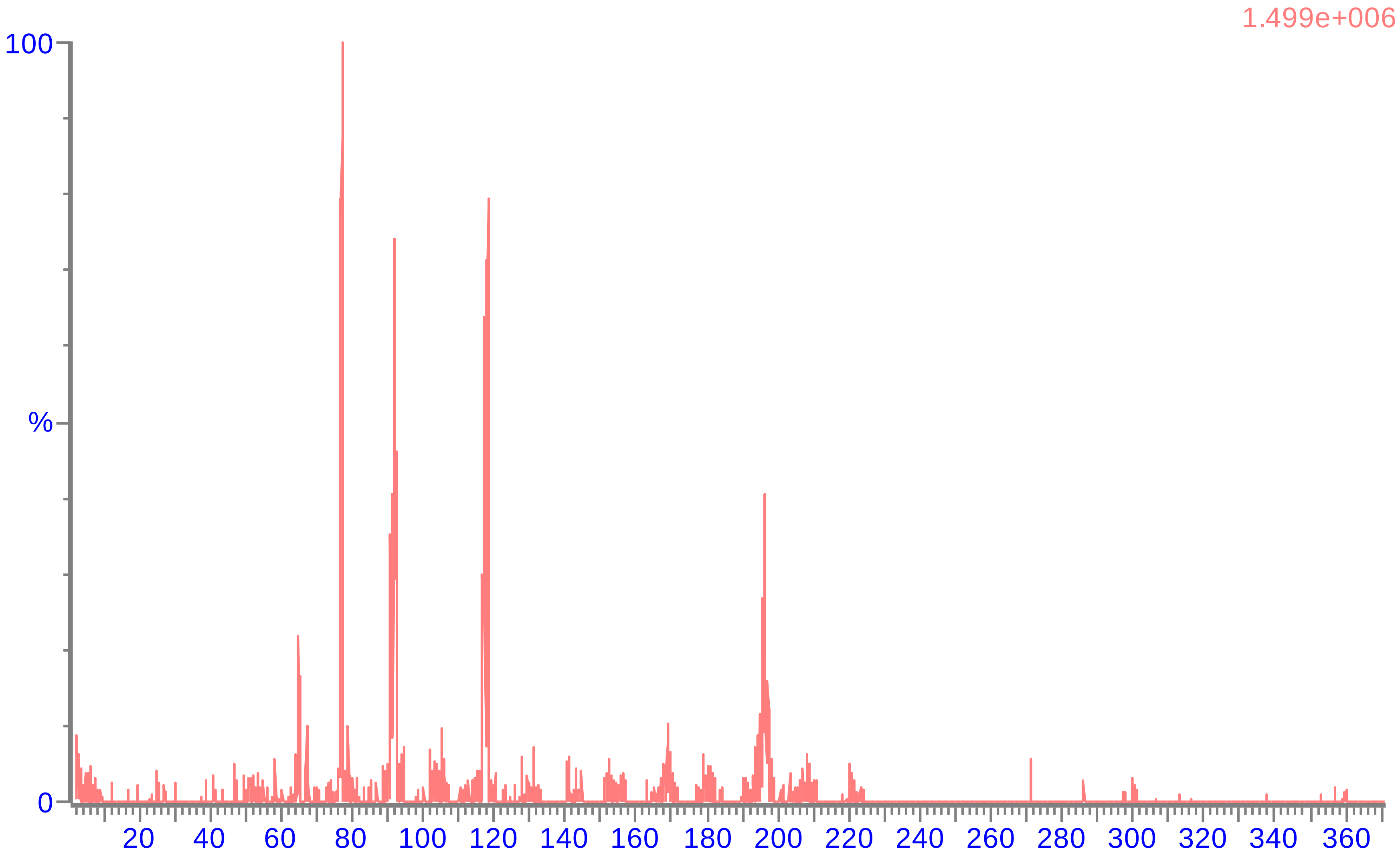

Figure 1.232: Mass spectrum for daughter fragment peak ES+, m/z 361.18 -> 91.90.

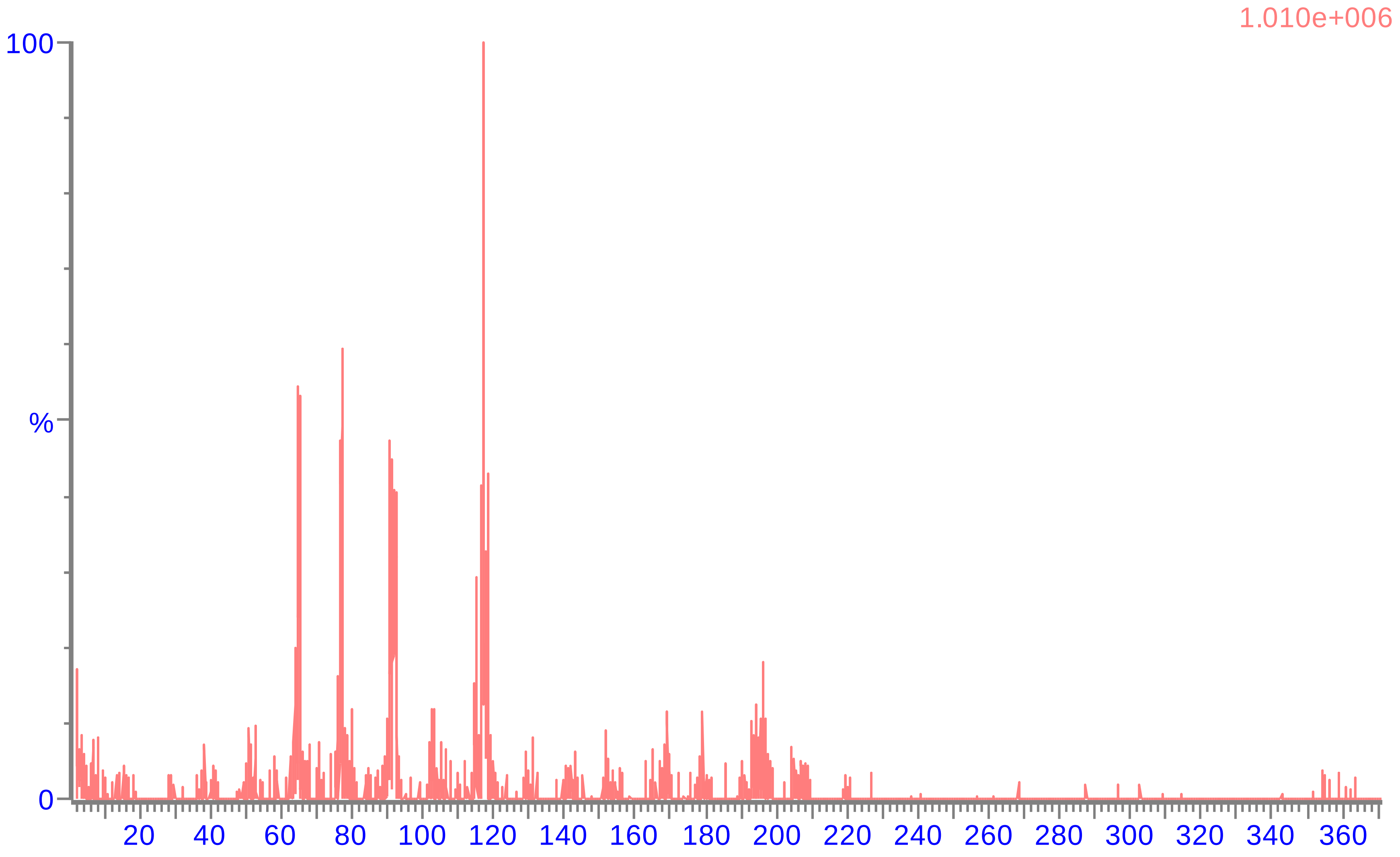

Figure 1.233: Mass spectrum for daughter fragment peak ES+, m/z 361.18 -> 64.97.

4j 1-(4-((1-(4-methoxyphenyl)-2-nitropropyl)amino)phenyl)ethanone

Table 1.33: MS data.

Compound	Formula/Mass		Parent m/z	Cone Voltage	Daughters	Collision Energy	Ion Mode
**4j**	328	1	329.10	34	148.03	24	ES+
		2	329.10	34	136.00	20	ES+
		3	329.10	34	254.11	18	ES+
		4	329.10	34	117.01	44	ES+
		5	329.10	34	105.04	56	ES+

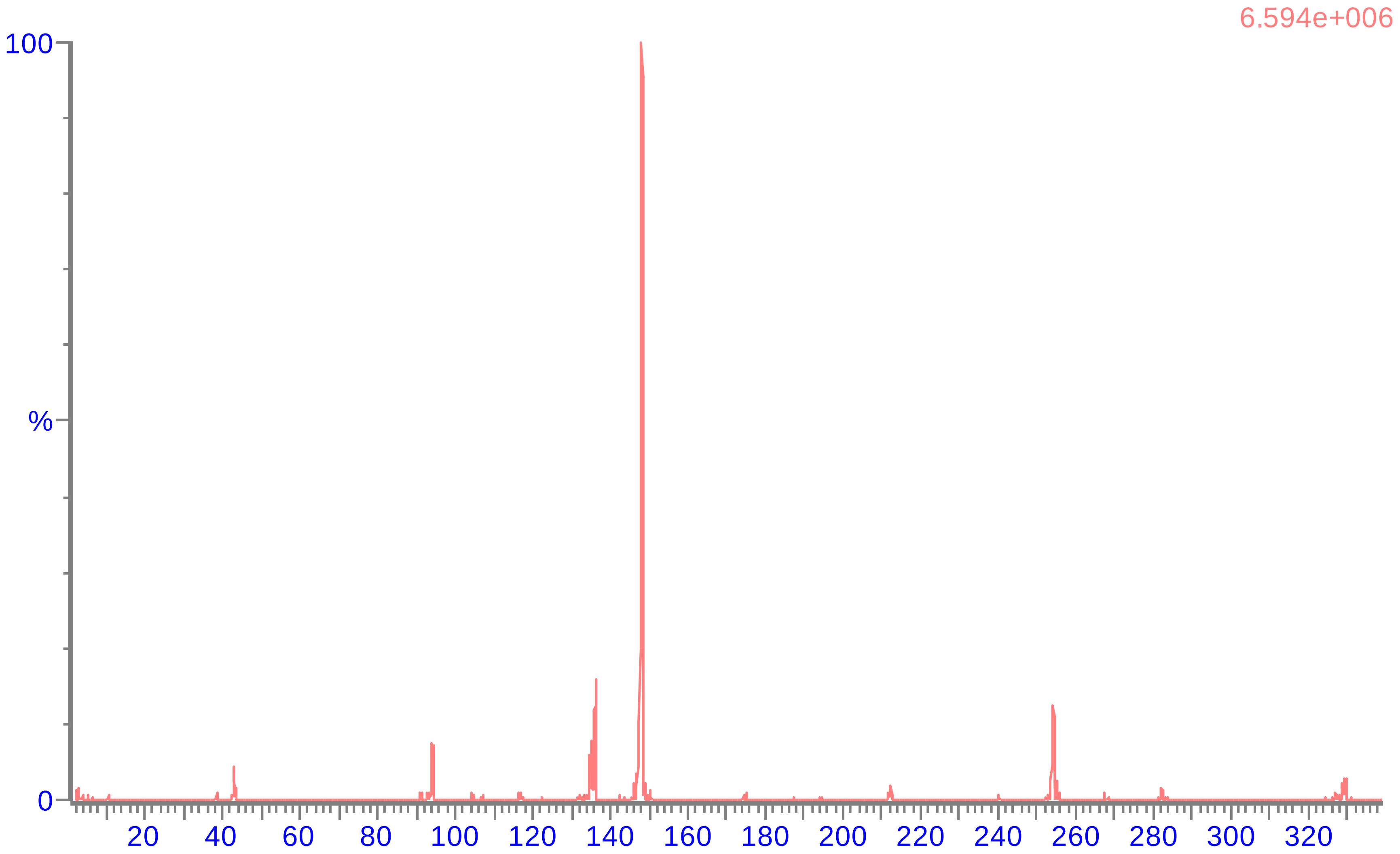

Figure 1.234: Mass spectrum for daughter fragment peak ES+, m/z 329.10 -> 148.03.

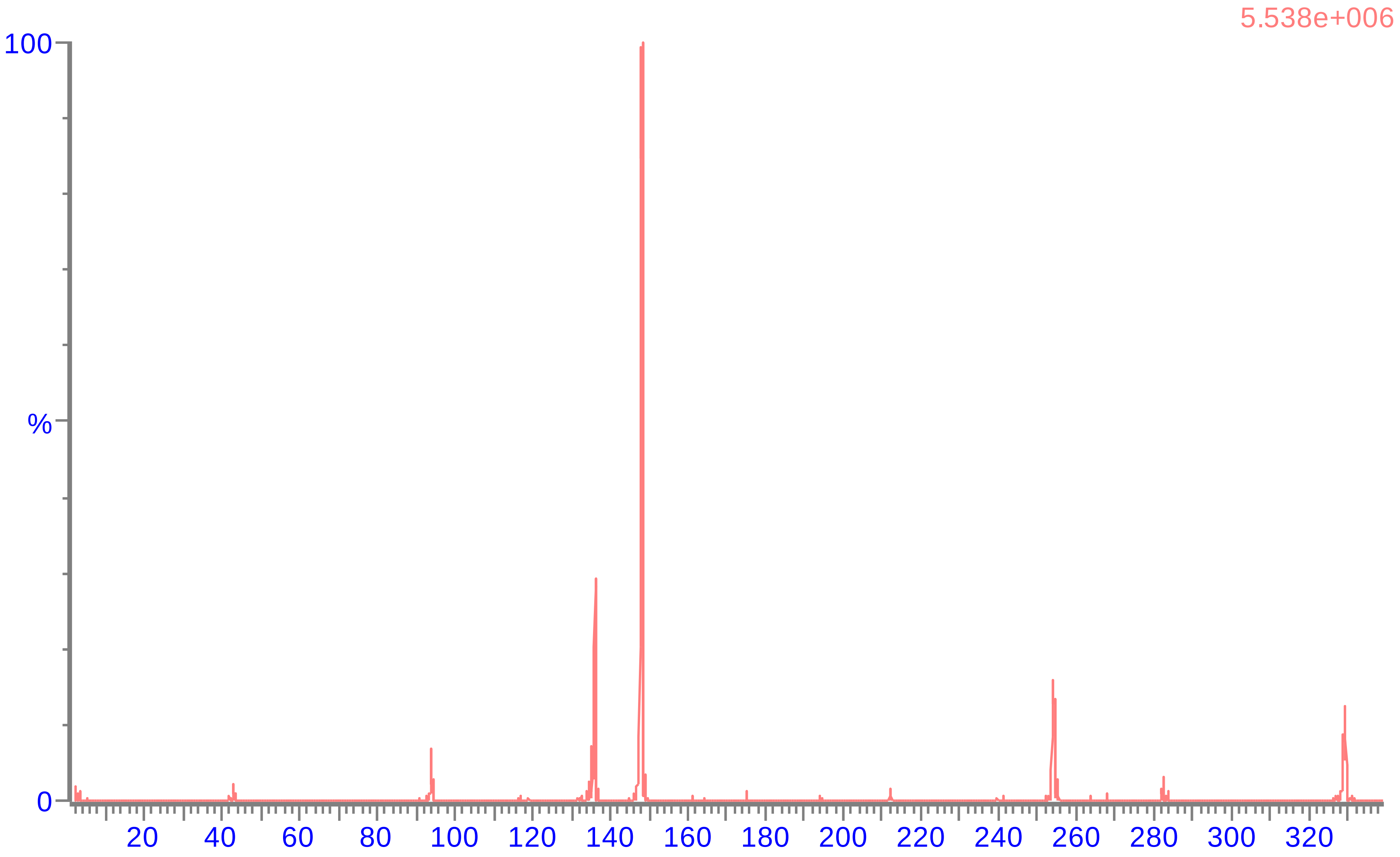

Figure 1.235: Mass spectrum for daughter fragment peak ES+, m/z 329.10 -> 136.00.

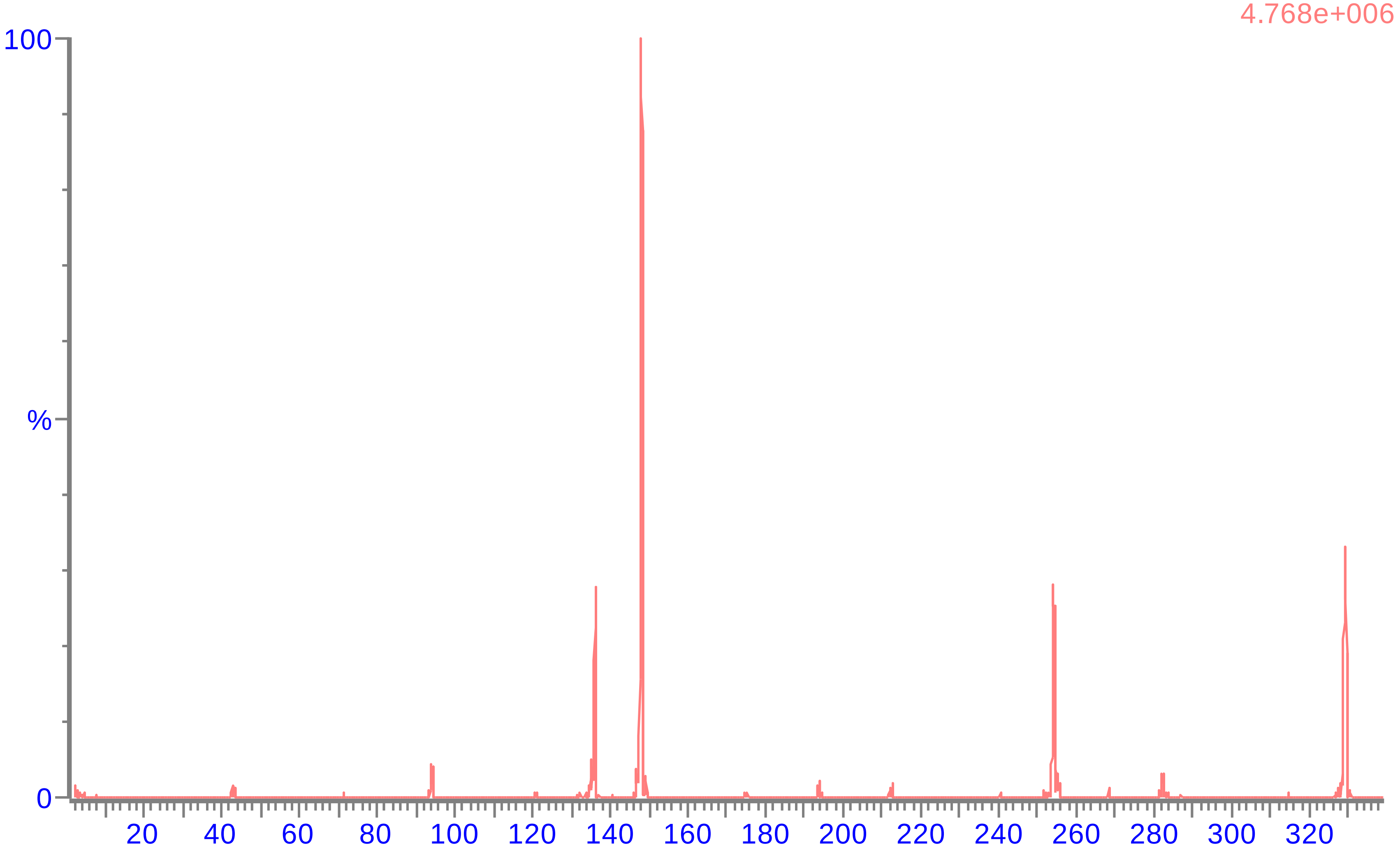

Figure 1.236: Mass spectrum for daughter fragment peak ES+, m/z 329.10 -> 254.11.

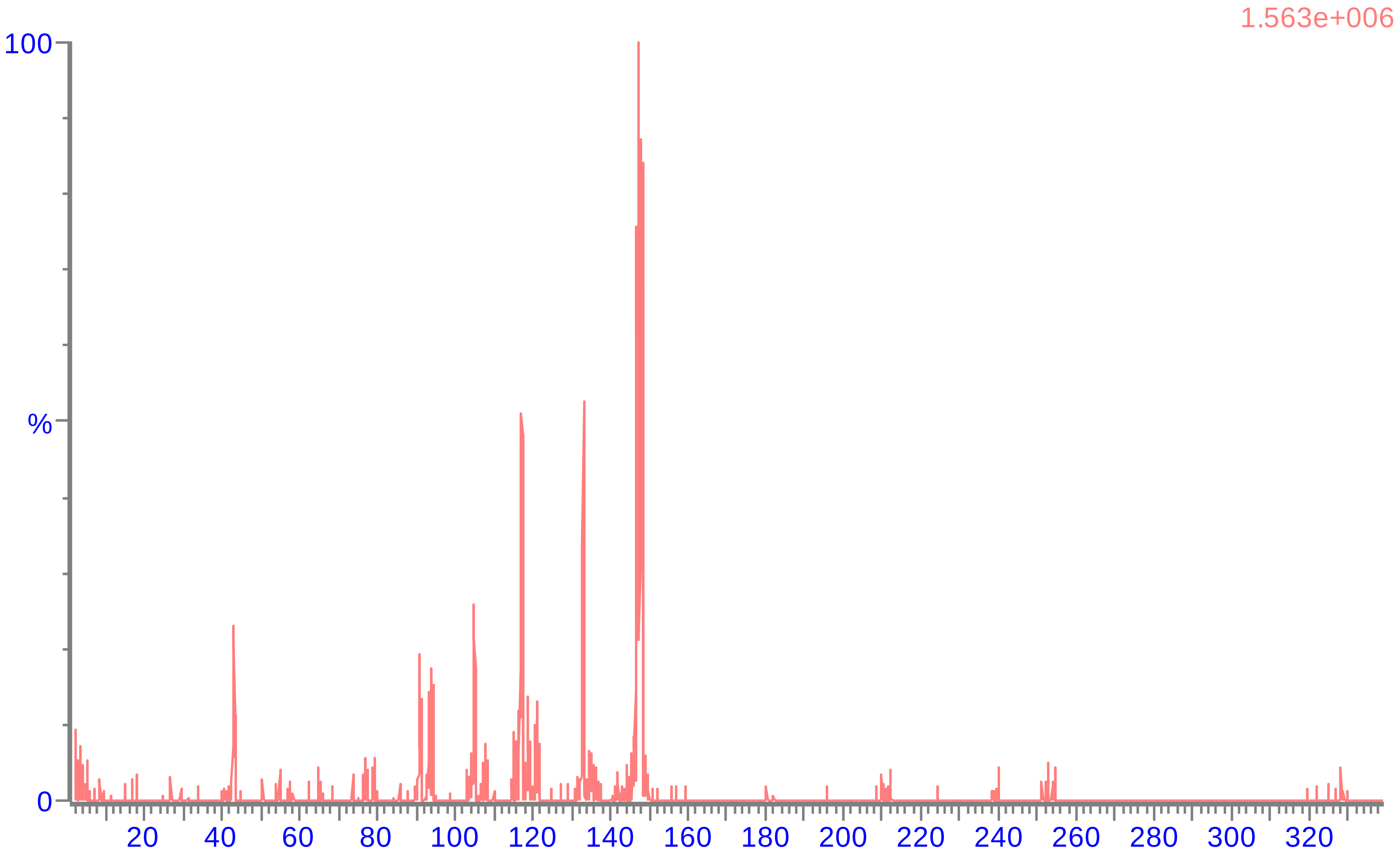

Figure 1.237: Mass spectrum for daughter fragment peak ES+, m/z 329.10 -> 117.01.

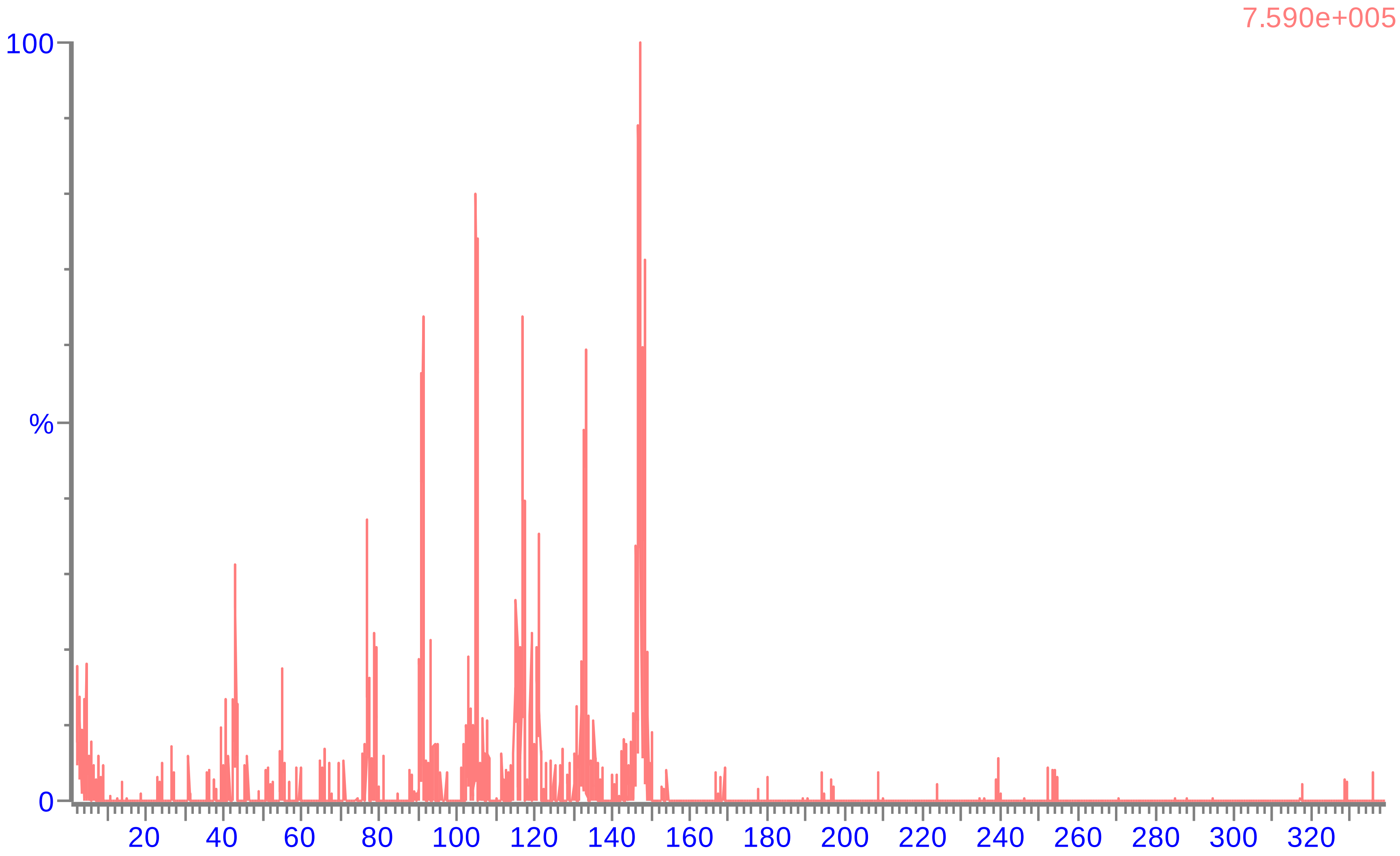

Figure 1.238: Mass spectrum for daughter fragment peak ES+, m/z 329.10 -> 105.04.

4k 4-methyl-*N*-(2-nitro-1-(2-nitrophenyl)butyl)aniline

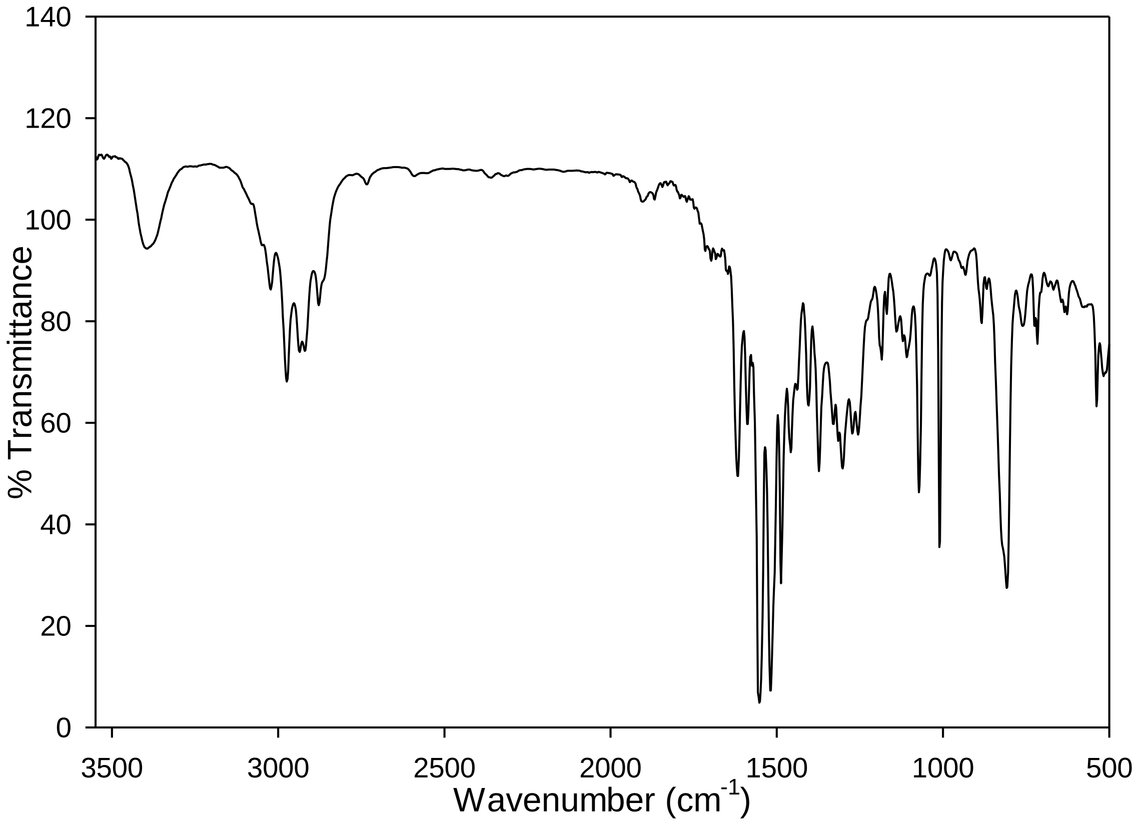

Figure 1.239: IR spectrum of **4k** 4-methyl-*N*-(2-nitro-1-(2-nitrophenyl)butyl)aniline.

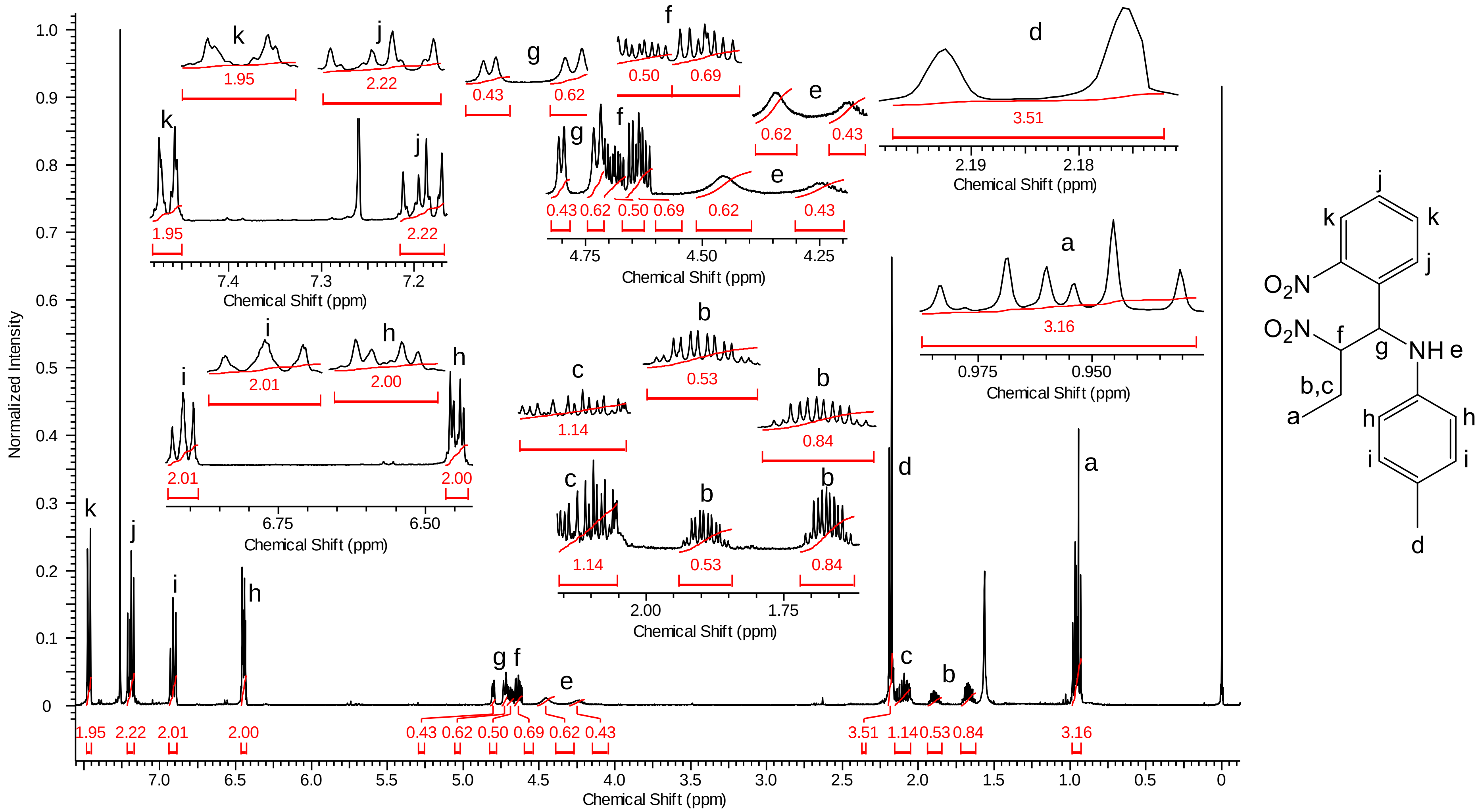

Figure 1.240: ^{1}H NMR spectrum of **4k** 4-methyl-*N*-(2-nitro-1-(2-nitrophenyl)butyl)aniline.

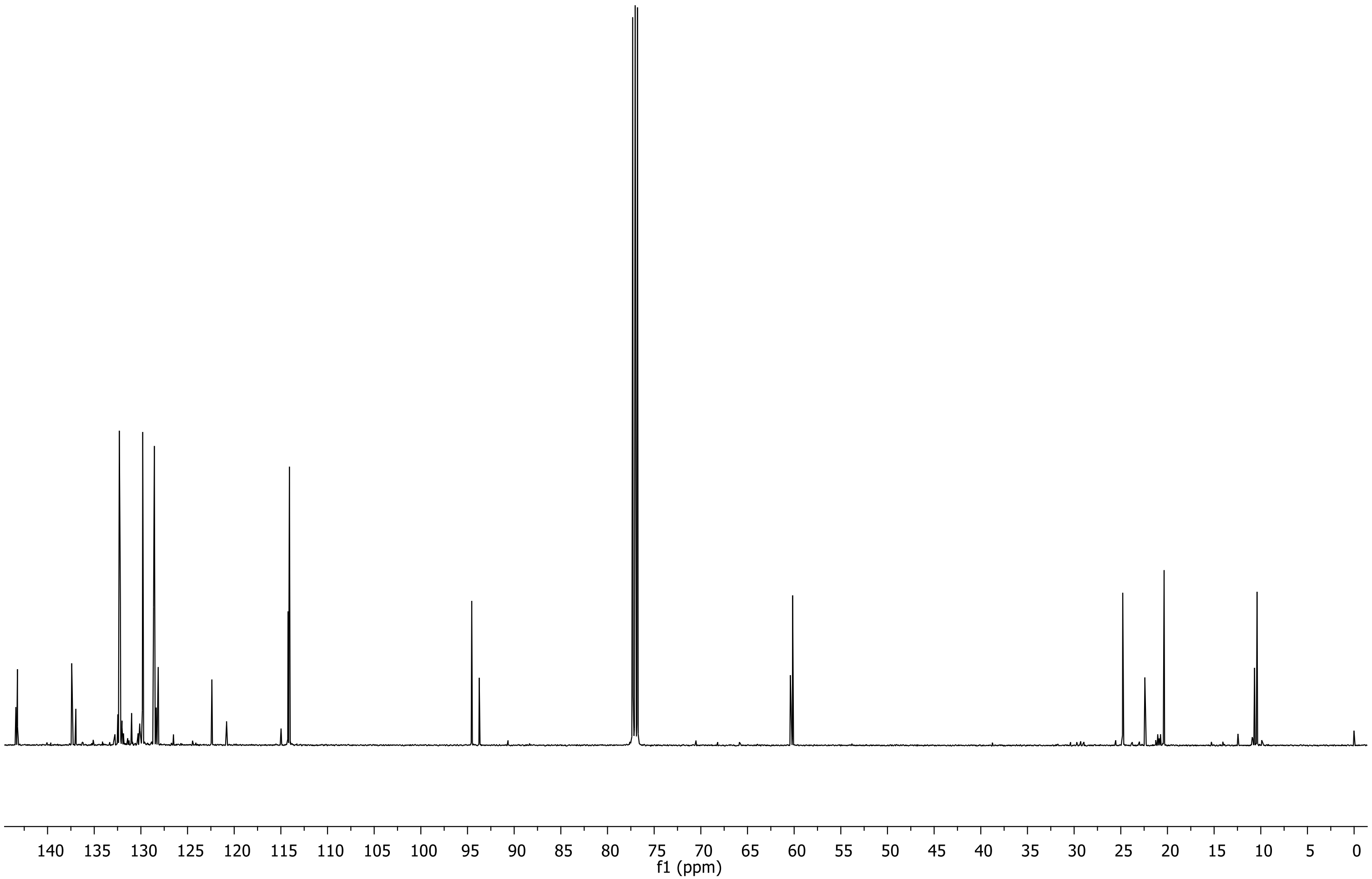

Figure 1.241: ^{13}C NMR spectrum of **4k** 4-methyl-*N*-(2-nitro-1-(2-nitrophenyl)butyl)aniline.

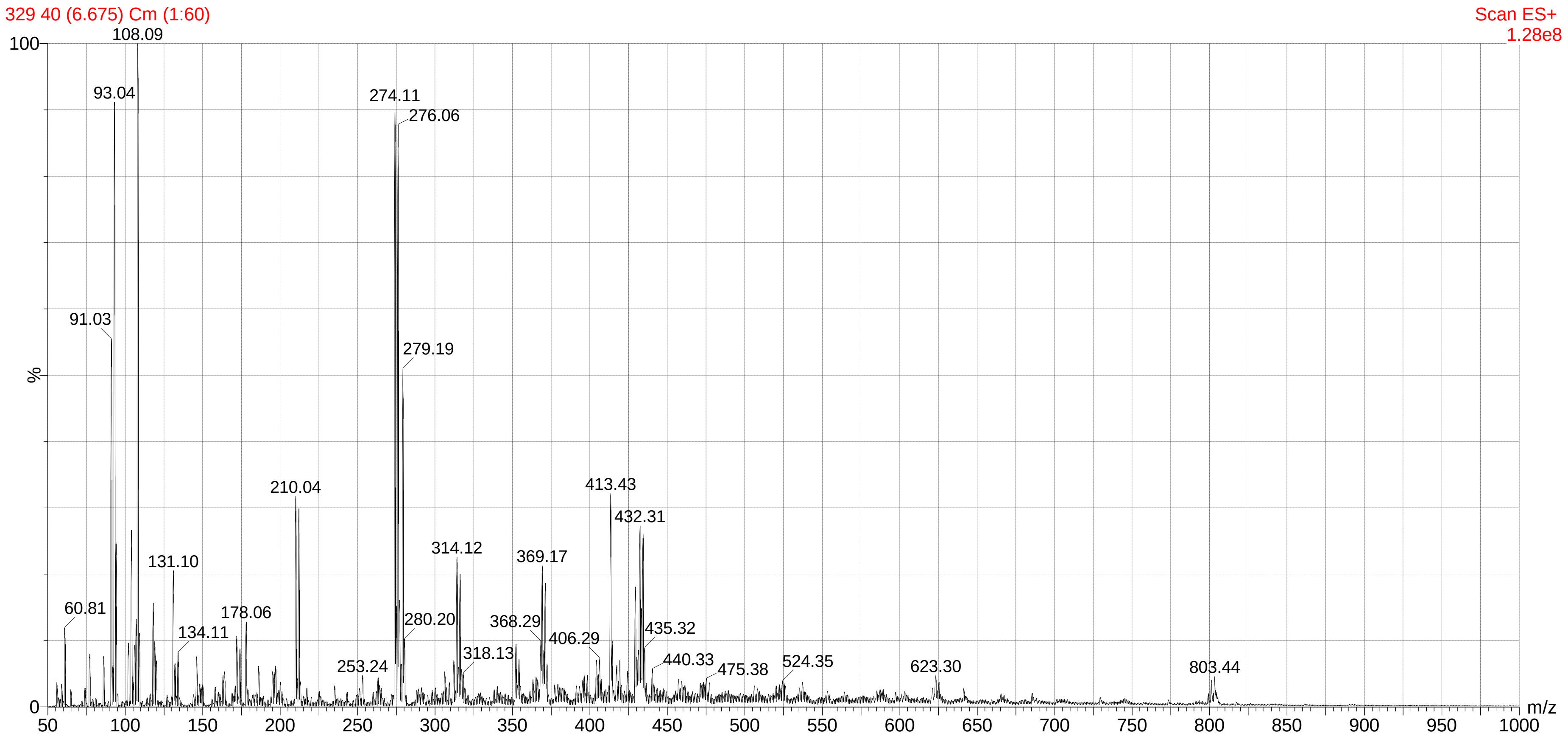

Figure 1.242: Mass spectrum of **4k** 4-methyl-*N*-(2-nitro-1-(2-nitrophenyl)butyl)aniline.
