# Supplementary material for: One-pot multicomponent nitro-Mannich reaction using a heterogeneous catalyst under solvent-free conditions
Source: PeerJ. 2018 Jun 27;6:e5065. doi: 10.7717/peerj.5065 (PMC6026460; doi:10.7717/peerj.5065)
Supplement: File S2 [file peerj-06-5065-s002.pdf]

Analytical Data for all products

6 *N*-(2-nitro-1-phenylethyl)aniline [54]. Brown solid. m.p. 73-75°C. IR (neat KBr, cm^{-1}): ν = 3354, 3078, 3058, 3027, 2970, 2916, 2894, 1604, 1546, 1514, 1508, 1489, 1458, 1425, 1381, 1314, 1264, 1213, 1181, 1127, 1083, 1067, 1032, 994, 940, 879, 851, 822, 756, 727, 701, 654, 628, 533, 511. ^{1}H NMR ($CDCl_3$, 500 MHz) δ (ppm): 4.35 (d, 1H, Ha, *J*=6.4 Hz), 4.70 (d, 2H, Hb, *J*=6.8 Hz), 5.15 (q, 1H, Hc, *J*=6.8 Hz), 6.60 (d, 2H, Hd, J_o=8.6 Hz, J_m=1Hz), 6.75 (t, 1H, He, *J*=7.4 Hz), 7.15 (t, 2H, Hf, *J*=8.0 Hz), 7.30 (m,1H Hg), 7.40 (m, 4H Hh, Hi). ^{13}C NMR ($CDCl_3$, 126 MHz) δ (ppm): 56.7, 80.0, 113.9, 119.0, 126.5, 128.7, 129.3, 129.4, 137.8, 145.7. MS (ES+) m/z = 242.07 [MH^+], 122.03, 94.06, 92.04, 86.98, 92.04.

7a *N*-(2-nitro-1-(*p*-tolyl)ethyl)aniline [55]. Dark-red thick oil. IR (neat NaCl, cm^{-1}): ν = 3400, 3091, 3053, 3027, 2954, 2921, 2863, 1633, 1603, 1554, 1506, 1458, 1436, 1423, 1378,1338, 1316, 1266, 1214, 1182, 1157, 1113, 1068, 1031, 1021, 994, 971, 914, 872, 814, 752, 693, 669, 510. ^{1}H NMR ($CDCl_3$, 500 MHz) δ (ppm): 2.33 (s, 3H, Ha), 4.35 (d, 1H, Hb, *J*=6.1 Hz), 4.70 (m, 2H, Hc), 5.15 (q, 1H, Hd, *J*=6.7 Hz), 6.61 (d, 2H, He, *J*=8.6 Hz), 6.75 (t, 1H, Hf, *J*=7.4 Hz), 7.14 (t, 2H, Hg, J_o=8.0 Hz, J_m=1.2 Hz), 7.18 (d, 2H, Hh, *J*=7.9 Hz), 7.25 (d, 2H, Hi, *J*=8.2 Hz). ^{13}C NMR ($CDCl_3$, 126 MHz) δ (ppm): 21.1, 56.5, 80.0, 113.9, 118.9, 126.4, 129.4, 130.0, 134.7, 138.5, 145.8. MS (ES+) m/z = 257.10 [MH^+], 196.12, 164.05, 118.06, 94.06, 91.07.

7b *N*-(1-(4-methoxyphenyl)-2-nitroethyl)aniline [36]. Dark-red thick oil. IR (neat NaCl, cm^{-1}): ν = 3382, 3054, 3030, 3011, 2961, 2935, 2912, 2937, 1604, 1552, 1514, 1472, 1440, 1424, 1379, 1335, 1308, 1251, 1216, 1177, 1156, 1110, 1070, 1030, 1001, 975, 917, 876, 860, 831, 754, 692, 668, 622, 563, 542. ^{1}H NMR ($CDCl_3$, 500 MHz) δ: 3.79 (s, 3H, Ha), 4.32 (d, 1H, Hb, *J*=7.6 Hz), 4.69 (m, 2H, Hc), 5.11 (q, 1H, Hd, *J*=6.4 Hz), 6.61 (d, 2H, He, *J*=8.6 Hz), 6.75 (t, 1H, Hf, *J*=7.4 Hz), 6.90 (d, 2H, Hg, *J*=8.8 Hz), 7.15 (t, 2H, Hh, *J*=7.8 Hz), 7.31 (d, 2H, Hi, *J*=8.6 Hz). ^{13}C NMR ($CDCl_3$, 126 MHz) δ (ppm): 55.7, 57.7, 80.1, 114.9, 115.7, 126.5, 128.6, 129.3, 138.0, 139.7, 153.1. MS (ES+) m/z = 273.10 [MH^+], 212.14, 123.05, 108.02, 104.06, 80.06.

7c *N*-(1-(4-bromophenyl)-2-nitroethyl)aniline. Dark-red thick oil. IR (neat NaCl, cm^{-1}): ν = 3404, 3087, 3053, 3026, 2963, 2918, 1603, 1553, 1508, 1423, 1404, 1377, 1335, 1314, 1263, 1215, 1180, 1155, 1128, 1099, 1072, 1011, 993, 902, 918, 876, 822, 752, 692, 648, 540, 509. ^{1}H NMR ($CDCl_3$, 500 MHz) δ (ppm): 4.40 (d, 1H, Ha, *J*=6.3 Hz), 4.69 (d, 2H, Hb, *J*=6.6 Hz), 5.12 (q, 1H, Hc, *J*=6.6 Hz), 6.58 (d, 2H, Hd, *J*=7.7 Hz), 6.75 (t, 1H, He, *J*=7.4 Hz), 7.15 (t, 2H, Hf *J*=7.4 Hz), 7.28 (d, 2H, Hg, *J*=8.5 Hz), 7.51 (d, 2H, Hh, *J*=8.5Hz). ^{13}C NMR ($CDCl_3$, 126 MHz) δ (ppm): 56.1, 79.7, 113.9, 119.2, 122.6, 128.2, 129.4, 132.5, 137.8, 145.3. MS (ES+) m/z = 323.04 [MH^+], 262.04, 183.99, 103.02, 93.98, 77.01.

7d *N*-(2-nitro-1-(2-nitrophenyl)ethyl)aniline [56]. Yellow-brown solid. m.p. 77-79°C. IR (neat, KBr, cm^{-1}): ν = 3375, 3104, 3078, 3030, 2920, 2862, 1622, 1580, 1560, 1522, 1474, 1435, 1386, 1340, 1289, 1263, 1250, 1215, 1182, 1154, 1131, 1086, 1066, 937, 921, 879, 860, 851, 811, 792, 750, 724, 708, 673, 647, 627, 560, 517, 505. ^{1}H NMR ($CDCl_3$, 500 MHz) δ

(ppm): 4.83 (m, 2H, Ha), 5.01 (dd, 1H, Hb J=3.7 Hz, 12.4 Hz), 5.80 (m, 1H, Hc), 6.48 (d, 2H, Hd, J_o=8.6 Hz, J_m=1.0 Hz), 6.75 (t, 1H, He, J_o=7.4 Hz, J_m=1.0 Hz), 7.10 (t, 2H, Hf, J=8.0 Hz), 7.50 (t, 1H, Hg, J_o=8.1 Hz, J_m=1.4 Hz), 7.60 (t, 1H, Hh, J_o=7.5 Hz, J_m=1.2 Hz), 7.69 (d, 1H, Hi, J_o=7.9 Hz, J_m=1.3 Hz), 8.09 (d, 1H, Hj, J_o=8.2 Hz, J_m=1.2 Hz).

7e 3-(2-Nitro-1-(phenylamino)ethyl)phenol. Dark-red oil. IR (neat NaCl, cm^{-1}): ν = 3407, 3097, 3072, 3036, 2923, 2856, 1613, 1580, 1560, 1509, 1451, 1422, 1380, 1344, 1325, 1289, 1273, 1257, 1215, 1186, 1154, 1138, 1083, 1066, 1028, 996, 970, 934, 918, 876, 860, 844, 792, 750, 718, 692, 669, 643, 611, 518. ^{1}H NMR ($CDCl_3$, 500 MHz) δ (ppm): 4.38 (m, 1H, Ha), 4.69 (d, 2H, Hb, J=6.7 Hz), 5.01 (broad, 1H, Hc), 5.11 (q, 1H, Hd, J=6.1 Hz), 6.60 (d, 2H, He, J=8.6 Hz), 6.75 (t, 1H, Hf, J=7.4 Hz), 6.76 (m, 1H, Hg), 6.85 (s, 1H, Hh), 6.95 (d, 1H, Hi, J=7.6 Hz), 7.15 (t, 2H, Hj, J_o=7.0 Hz, J_m=1.2 Hz), 7.25 (t, 1H, Hk, J=7.9 Hz). ^{13}C NMR ($CDCl_3$, 126 MHz) δ (ppm): 56.4, 79.9, 113.4, 113.9, 115.7, 118.6, 119.0, 129.4, 130.6, 139.6, 145.6, 156.4. MS (ES+) m/z = 259.10 [MH^+], 212.17, 198.06, 166.06, 120.07, 94.05.

7f *N*-(1-(naphthalene-1-yl)-2-nitroethyl)aniline [43]. Yellow-red solid. m.p. 93-96°C. IR (neat, KBr, cm^{-1}): ν = 3389, 3086, 3057, 3015, 2915, 2851, 1652, 1604, 1558, 1507, 1459, 1437, 1424, 1394, 1375, 1320, 1266, 1230, 1210, 1197, 1184, 1165, 1136, 1078, 1068, 1033, 997, 977, 965, 936, 926, 913, 871, 807, 794, 781, 758, 700, 674, 632, 616, 590, 558, 542, 507, 474, 422. ^{1}H NMR ($CDCl_3$, 500 MHz) δ (ppm): 4.55 (d, 1H, Ha, J=5.0 Hz), 4.70 (dd, 1H, Hb, J=9.1 Hz, 12.4 Hz), 4.95 (dd, 1H, Hc, J=4.2 Hz, 12.4 Hz), 6.00 (m, 1H, Hd), 6.57 (d, 2H, He, J_o=8.6 Hz, J_m=1.0 Hz), 6.73 (t, 1H, Hf, J_o=7.4 Hz, J_m=0.9 Hz), 7.10 (t, 2H, Hg, J_o=8.0 Hz, J_m=2.0 Hz), 7.43 (t, 1H, Hh, J=7.6 Hz), 7.58 (t, 1H, Hi, J=7.6 Hz), 7.66 (d, 1H, Hj, J=6.5 Hz), 7.66 (t, 1H, Hk, J=7.6 Hz), 7.85 (d, 1H, Hl, J=8.2 Hz), 7.95 (d, 1H, Hm, J=8.2 Hz), 8.20 (d, 1H, Hn, J=8.5 Hz). MS (ES+) m/z = 293.10 [MH^+], 200.07, 198.07, 154.02, 119.03, 93.99.

7g *N*-(1-([1,1-biphenyl]-4-yl)-2-nitroethyl)aniline. Yellow-red solid. m.p. 97-99°C. IR (neat, KBr, cm^{-1}): ν = 3398, 3055, 3030, 2960, 2918, 1603, 1549, 1511, 1486, 1476, 1460, 1441, 1422, 1381, 1314, 1266, 1257, 1213, 1180, 1155, 1133, 1076, 1008, 993, 920, 876, 837, 768, 755, 732, 695, 650, 621, 561, 507. ^{1}H NMR ($CDCl_3$, 500 MHz) δ (ppm): 4.42 (d, 1H, Ha, J=6.4 Hz), 4.75 (d, 2H, Hb, J=6.8 Hz), 5.22 (q, 1H, Hc, J=6.6 Hz), 6.65 (d, 2H, Hd, J_o=8.6 Hz, J_m=1.0 Hz), 6.75 (t, 1H, He, J_o=7.4 Hz, J_m=1.0 Hz), 7.16 (t, 2H, Hf, J_o=8.0 Hz, J_m=1.2 Hz), 7.35 (t, 1H, Hg, J_o=7.4 Hz, J_m=1.3 Hz), 7.43 (t, 2H, Hh, J=7.6 Hz), 7.47 (d, 2H, Hi, J_o=8.1 Hz, J_m=1.6 Hz), 7.55 (d, 2H, Hj, J_o=8.2 Hz, J_m=2.0 Hz), 7.60 (d, 2H, Hk, J_o=8.4 Hz, J_m=2.0 Hz). ^{13}C NMR ($CDCl_3$, 126 MHz) δ (ppm): 56.5, 79.9, 114.0, 119.0, 126.9, 127.1, 127.6, 128.0, 128.9, 129.4, 136.7, 140.3, 141.7, 145.7. MS (ES+) m/z = 327.03 [MH^+], 80.91.

7h *N*-(1-(3,4-dichlorophenyl)-2-nitroethyl)aniline. Dark-red thick oil. IR (neat NaCl, cm^{-1}): ν = 3403, 3093, 3054, 3025, 2960, 2922, 2857, 1604, 1556, 1508, 1472, 1423, 1378, 1313, 1262, 1214, 1194, 1181, 1136, 1075, 1033, 997, 929, 884, 826, 752, 697, 668, 619, 584. ^{1}H NMR ($CDCl_3$, 500 MHz) δ (ppm): 4.43 (broad, 1H, Ha), 4.68 (d, 2H, Hb, J=6.9 Hz), 5.11 (q, 1H, Hc, J=6.0 Hz), 6.57 (d, 2H, Hd, J_o=9.1 Hz, J_m=0.95 Hz), 6.78 (t, 1H, He, J_o=7.4 Hz, J_m=1.0 Hz), 7.15 (t, 2H, Hf, J=8.0 Hz), 7.25 (d, 1H, Hg, J=9.3 Hz), 7.45 (d, 1H, Hh, J=8.2

Hz), 7.51 (s, 1H, Hi). ^{13}C NMR (CDCl$_3$, 126 MHz) δ (ppm): 55.8, 79.6, 114.0, 119.5, 125.8, 128.6, 129.5, 131.6, 132.9, 133.6, 138.1, 145.1. MS (ES+) m/z = 312.97 [MH$^+$], 252.96, 252.02, 173.91, 101.73, 93.98.

7i *N*-(1-(2,6-dichlorophenyl)-2-nitroethyl)aniline. Red solid. m.p. 96-99°C. IR (neat, KBr, cm^{-1}): ν = 3388, 3061, 3049, 3033, 2982, 2928, 2855, 1603, 1581, 1555, 1508, 1495, 1457, 1441, 1381, 1339, 1311, 1289, 1266, 1250, 1216, 1200, 1181, 1152, 1123, 1089, 1056, 1028, 993, 923, 901, 885, 860, 834, 793, 774, 758, 726, 695, 640, 621, 558, 495. ^{1}H NMR (CDCl$_3$, 500 MHz) δ (ppm): 4.73 (dd, 1H, Ha, J=5.2 Hz, 12.3 Hz), 4.95 (d, 1H, Hb, 11.2 Hz), 5.07 (dd, 1H, Hc, 9.9 Hz, 12.3 Hz), 6.29 (ddd, 1H, Hd, 11.2 Hz, 10.0 Hz, 5.3 Hz), 6.74 (m, 3H, He, Hf), 7.13-7.19 (m, 3H, Hg, Hh), 7.27-7.38 (m, 2H, Hi). ^{13}C NMR (CDCl$_3$, 126 MHz) δ (ppm): 53.3, 76.5, 114.2, 119.4, 129.4, 130.2, 132.2, 145.3. MS (ES+) m/z = 310.97 [MH$^+$], 250.02, 136.98, 124.95, 93.33.

7j *N*-(1-(benzo[*d*][1,3]dioxol-5-yl)-2-nitroethyl)aniline. Red thick oil. IR (neat NaCl, cm^{-1}): ν = 3393, 3051, 3028, 2960, 2896, 1607, 1559, 1508, 1491, 1449, 1378, 1339, 1313, 1246, 1184, 1155, 1110, 1036, 997, 971, 929, 871, 816, 755, 733, 693, 668, 639. ^{1}H NMR (CDCl$_3$, 500 MHz) δ (ppm): 4.35 (broad, 1H, Ha), 4.66 (d, 2H, Hb, *J*=6.7 Hz), 5.07 (q, 1H, Hc, *J*=6.0 Hz), 5.96 (m, 2H, Hd), 6.61 (d, 2H, He, J_o=8.6 Hz, J_m=1.0 Hz), 6.75 (t, 1H, Hf, *J*=7.3 Hz), 6.79 (d, 1H, Hg, *J*=8.6 Hz), 6.87 (m, 2H, Hh, Hi), 7.15 (t, 2H, Hj, J_o=7.4 Hz, J_m=2.0 Hz). ^{13}C NMR (CDCl$_3$, 126 MHz) δ (ppm): 56.5, 80.2, 101.4, 106.7, 108.9, 113.9, 119.0, 120.0, 129.4, 131.6, 145.6, 147.9, 148.5. MS (ES+) m/z = 287.10 [MH$^+$], 194.02, 148.03, 94.05, 91.64, 90.99.

7k *N*-(1-nitropentan-2-yl)aniline. Red oil. IR (neat NaCl, cm^{-1}): ν = 3367, 3055, 3017, 2965, 2931, 2875, 1684, 1604, 1555, 1500, 1463, 1445, 1383, 1362, 1317, 1258, 1220, 1185, 1140, 1075, 1047, 994, 922, 839, 756, 692, 669. ^{1}H NMR (CDCl$_3$, 500 MHz) δ (ppm): 0.95 (t, 3H, Ha, *J*= 7.3 Hz), 1.40-1.70 (m, 4H, Hb, Hc), 3.65 (d, 1H, Hd, *J*=7.2 Hz), 4.10 (m, 1H, He), 4.40 (dd, 1H, Hf, *J*=6.2 Hz, 11.8 Hz), 4.50, (dd, 1H, Hg, *J*=5.1 Hz, 11.8 Hz), 6.65 (d, 2H, Hh, J_o=8.6 Hz, J_m=0.9 Hz), 6.75 (t, 1H, Hi, J_o=7.4 Hz, J_m=0.9 Hz), 7.20 (t, 2H, Hj, J_o=8.0 Hz, J_m=1.2 Hz). ^{13}C NMR (CDCl$_3$, 126 MHz) δ (ppm): 13.8, 19.1, 35.1, 52.0, 78.1, 113.5, 118.7, 129.6, 146.0. MS (ES+) m/z = 209.10 [MH$^+$], 148.11, 118.25, 106.02, 93.08, 40.98.

7l *N*-(1-cyclohexyl-2-nitroethyl)aniline. Red oil. (neat NaCl, cm^{-1}): ν = 3399, 3053, 3022, 2930, 2853, 1601, 1557, 1504, 1449, 1427, 1381, 1348, 1315, 1256, 1215, 1180, 1155, 1123, 1072, 1030, 993, 964, 914, 893, 874, 841, 752, 692, 667, 619, 509. ^{1}H NMR (CDCl3, 500 MHz) δ: 1.00-1.30 (m, 5H, Ha, Hb, Hc), 1.55-1.85 (m, 5H, Hd, He, Hf), 1.91 (d, 1H, Hg, *J*=12.5 Hz), 3.70 (d, 1H, Hh, *J*= 9.8 Hz), 3.94-4.00 (m, 1H, Hi), 4.45 (dd, 1H, Hj *J*=6.3 Hz, 12.1 Hz), 4.55 (dd, 1H, Hk, *J*=5.7 Hz, 12.1 Hz), 6.65 (d, 2H, Hl, *J*=7.7 Hz), 6.75 (t, 1H, Hm, *J*=7.3 Hz), 7.2 (t, 2H, Hn, J_o=7.9 Hz, J_m=1.0 Hz). ^{13}C NMR (CDCl$_3$, 126 MHz) δ (ppm): 26.0, 26.1, 28.3, 29.6, 40.6, 57.0, 76.5, 113.5, 118.4, 129.5, 146.6. MS (ES+) m/z = 249.14 [MH$^+$], 188.17, 109.08, 106.02, 94.71, 67.06.

7m Ethyl 3-nitro-2-(phenylamino)propanoate. Red oil. IR (neat NaCl, cm^{-1}): ν = 3388, 3054, 3028, 2983, 2936, 2905, 2870, 1742, 1605, 1558, 1507, 1447, 1421, 1380, 1313, 1281, 1256, 1215, 1158, 1100, 1078, 1053, 1018, 958, 922, 875, 856, 757, 693. ^{1}H NMR ($CDCl_3$, 500 MHz) δ (ppm): 1.29 (t, 3H, Ha, *J*=7.1 Hz), 4.29 (m, 2H, Hb), 4.51 (d, 1H, Hc, *J*=7.7 Hz), 4.64 (m, 1H, Hd), 4.79 (dd, 1H, He, *J*=5.0 Hz, 13.7 Hz), 4.87 (dd, 1H, Hf, *J*=4.5 Hz, 13.7 Hz), 6.68 (d, 2H, Hg, *J*=7.7 Hz), 6.84 (t, 1H, Hh, *J*=7.4 Hz), 7.22 (t, 2H, Hi, J_o= 8.0 Hz, J_m= 1.9 Hz). ^{13}C NMR ($CDCl_3$, 126 MHz) δ (ppm): 14.0, 54.9, 62.7, 75.6, 113.8, 119.7, 129.6, 145.2, 169.5. MS (ES+) m/z = 239.10 [MH^+], 178.11, 150.07, 104.02, 93.02, 77.02.

8a 4-Methoxy-*N*-(2-nitro-1-phenylethyl)aniline [54,57]. Dark red thick oil. IR (neat NaCl, cm^{-1}): ν = 3366, 3062, 3031, 3002, 2954, 2933, 2916, 2834, 1559, 1513, 1456, 1423, 1377, 1339, 1292, 1265, 1241, 1182, 1132, 1106, 1077, 1034, 1002, 973, 922, 824, 760, 701, 667, 616, 536, 515. ^{1}H NMR ($CDCl_3$, 500 MHz) δ (ppm): 3.70 (s, 1H, Ha), 4.70 (d, 2H, Hb, *J*= 6.8 Hz), 5.09 (t, 1H, Hc, *J*=6.7 Hz), 6.58 (d, 2H, Hd, *J*=9.0 Hz), 6.73 (d, 2H, He, *J*=9.0 Hz), 7.30-7.34 (m, 1H, Hf), 7.36-7.40 (m, 4H, Hg, Hh).

8b 4-Methyl-*N*-(2-nitro-1-phenylethyl)aniline [43]. Yellow solid. m.p. 78-80°C. IR (neat, KBr, cm^{-1}): ν = 3354, 3090, 3067, 3034, 3018, 2967, 2918, 2870, 1620, 1549, 1520, 1494, 1459, 1433, 1381, 1352, 1320, 1304, 1256, 1213, 1184, 1159, 1136, 1114, 1084, 1029, 1004, 939, 858, 813, 768, 723, 707, 649, 616, 571, 515, 478, 455. ^{1}H NMR ($CDCl_3$, 500 MHz) δ (ppm): 2.20 (s, 3H, Ha), 4.25 (d, 1H, Hb, *J*=5.5 Hz), 4.70 (d, 2H, Hc, *J*=6.7 Hz), 5.15 (q, 1H, Hd, *J*=6.6 Hz), 6.53 (d, 2H, He, *J*=8.5 Hz), 6.95 (d, 2H, Hf, J_o=8.5 Hz, J_m=0.6 Hz), 7.30-7.33 (m, 1H, Hg), 7.35-7.40 (m, 4H, Hh, Hi).

8c 3-Methyl-*N*-(2-nitro-1-phenylethyl)aniline. Red thick oil. IR (neat NaCl, cm^{-1}): ν = 3396, 3027, 2949, 2919, 2859, 1608, 1591, 1558, 1508, 1491, 1453, 1424, 1380, 1339, 1323, 1269, 1215, 1178, 1126, 1078, 1032, 998, 970, 926, 847, 768, 699, 668, 624, 592, 527. ^{1}H NMR ($CDCl_3$, 500 MHz) δ (ppm): 2.24 (s, 3H, Ha), 4.31 (d, 1H, Hb, *J*=6.4 Hz), 4.71 (d, 2H, Hc, *J*=6.8 Hz), 5.17 (q, 1H, Hd, *J*=6.7 Hz), 6.41 (d, 1H, He, J_o=8.0 Hz, J_m=2.2 Hz), 6.46 (s, 1H, Hf), 6.58 (d, 1H, Hg, *J*=7.5 Hz), 7.03 (t, 1H, Hh, *J*=7.8 Hz), 7.31-7.34 (m, 1H, Hi), 7.35-7.41 (m, 4H, Hj, Hk). ^{13}C NMR ($CDCl_3$, 126 MHz) δ (ppm): 21.5, 56.6, 79.9, 110.9, 114.8, 119.9, 126.4, 128.6, 129.2, 129.3, 137.8, 139.2, 145.7. MS (ES+) m/z = 257.08 [MH^+], 196.11, 149.99, 107.98, 104.07, 78.05.

8d 2-Methyl-*N*-(2-nitro-1-phenylethyl)aniline. Red thick oil. IR (neat NaCl, cm^{-1}): ν = 3417, 3058, 3024, 2967, 2915, 2853, 1636, 1608, 1588, 1554, 1508, 1481, 1455, 1423, 1377, 1345, 1313, 1263, 12161189, 1160, 1136, 1074, 1053, 1030, 1003, 987, 968, 923, 844, 751, 701, 653, 621, 592, 536. ^{1}H NMR ($CDCl_3$, 500 MHz) δ (ppm): 2.23 (s, 3H, Ha), 4.35 (d, 1H, Hb, *J*=5.5 Hz), 4.75 (d, 2H, Hc, *J*=6.7 Hz), 5.19 (q, 1H, Hd, *J*=6.4 Hz), 6.46 (d, 1H, He, *J*=8.0 Hz), 6.69 (t, 1H, Hf, J_o=7.3 Hz, J_m=0.8 Hz), 7.00 (t, 1H, Hg, J_o=7.5 Hz, J_m=1.0 Hz), 7.08 (d, 1H, Hh, *J*=7.4 Hz), 7.31-7.34 (m, 1H, Hi), 7.36-7.42 (m, 4H, Hj, Hk). ^{13}C NMR ($CDCl_3$, 126 MHz) δ (ppm): 17.5, 56.8, 80.3, 111.2, 118.5, 123.0, 126.4, 127.1, 128.7, 129.4, 130.4, 137.9, 143.7. MS (ES+) m/z = 257.08 [MH^+], 196.11, 149.99, 107.98, 104.07, 77.99.

8e 4-Ethyl-*N*-(2-nitro-1-phenylethyl)aniline. Red thick oil. IR (neat NaCl, cm^{-1}): ν = 3400, 3062, 3027, 2962, 2928, 2889, 2870, 1615, 1557, 1521, 1494, 1452, 1426, 1414, 1377, 1343, 1313, 1265, 1212, 1184, 1134, 1113, 1076, 1029, 1000, 968, 921, 826, 767, 700, 644, 628, 540. ^{1}H NMR ($CDCl_3$, 500 MHz) δ (ppm): 1.15 (t, 3H, Ha, *J*=7.6 Hz), 2.50 (q, 2H, Hb, *J*=7.6 Hz), 4.27 (broad, 1H, Hc), 4.70 (d, 2H, Hd, *J*=6.8 Hz), 5.14 (broad, 1H, He), 6.55 (d, 2H, Hf, *J*=8.5 Hz), 6.98 (d, 2H, Hg, *J*=8.5 Hz), 7.32 (m, 1H, Hh), 7.36-7.41 (m, 4H, Hi, Hj). ^{13}C NMR ($CDCl_3$, 126 MHz) δ (ppm): 15.8, 27.9, 57.0, 80.0, 114.1, 126.5, 128.6, 128.7, 129.3, 134.8, 138.0, 143.6. MS (ES+) m/z = 271.08 [MH^+], 210.11, 121.37, 104.01, 103.69, 78.06.

8f 4-Bromo-*N*-(2-nitro-1-phenylethyl)aniline [55]. Dark-red thick oil. IR (neat NaCl, cm^{-1}): ν = 3404, 3063, 3031, 3005, 2971, 2915, 1596, 1556, 1493, 1455, 1426, 1398, 1380, 1339, 1317, 1294, 1260, 1211, 1182, 1129, 1113, 1076, 1027, 1003, 970, 922, 816, 763, 701, 669, 636, 604, 534, 501. ^{1}H NMR ($CDCl_3$, 500 MHz) δ (ppm): 4.45 (d, 1H, Ha, *J*= 6.5 Hz), 4.70 (m, 2H, Hb), 5.11 (q, 1H, Hc, *J*= 6.9 Hz), 6.48 (d, 2H, Hd, *J*=8.9 Hz), 7.21 (d, 2H, He, *J*=8.9 Hz), 7.31-7.41 (m, Hf, Hg, Hh). MS (ES+) m/z = 321.03 [MH^+], 260.03, 171.96, 150.05, 104.06, 77.98.

8g 4-Chloro-*N*-(2-nitro-1-phenylethyl)aniline [56,58]. Dark-red thick oil. IR (neat NaCl, cm^{-1}): ν = 3401, 3066, 3032, 3005, 2968, 2919, 2843, 1601, 1555, 1501, 1457, 1423, 1404, 1380, 1340, 1313, 1294, 1260, 1212, 1177, 1131, 1092, 1003, 968, 920, 819, 766, 739, 700, 668, 640, 536, 501. ^{1}H NMR ($CDCl_3$, 500 MHz) δ (ppm): 4.45 (d, 1H, Ha, *J*= 6.5 Hz), 4.70 (m, 2H, Hb), 5.11 (q, 1H, Hc, *J*= 7.0 Hz), 6.52 (d, 2H, Hd, *J*=8.9 Hz), 7.09 (d, 2H, He, *J*=8.9 Hz), 7.31-7.41 (m, Hf, Hg, Hh). ^{13}C NMR ($CDCl_3$, 126 MHz) δ (ppm): 56.8, 80.0, 115.1, 123.7, 126.4, 128.8, 129.2, 129.4, 137.3, 144.3. MS (ES+) m/z = 277.07 [MH^+], 216.05, 128.02.

8h 1-(4-((2-Nitro-1-phenylethyl)amino)phenyl)ethanone. Dark-red thick oil. IR (neat NaCl, cm^{-1}): ν = 3319, 3156, 3087, 3068, 3027, 2963, 2919, 2864, 1651, 1594, 1562, 1533, 1496, 1457, 1425, 1378, 1362, 1356, 1283, 1216, 1181, 1136, 1117, 1073, 1025, 955, 926, 834, 812, 765, 724, 704, 638, 599, 577. ^{1}H NMR ($CDCl_3$, 500 MHz) δ (ppm): 2.47 (s, 3H, Ha), 4.75 (m, 2H, Hb), 4.99 (d, 1H, Hc, *J*=6.7 Hz), 5.26 (q, 1H, Hd, *J*=7.3 Hz), 6.60 (d, 2H, He, J_o=6.9 Hz, J_m=1.9 Hz), 7.33-7.42 (m, 5H, Hf, Hg, Hh), 7.79 (d, 2H, Hi, J_o=6.9 Hz, J_m=1.9 Hz). ^{13}C NMR ($CDCl_3$, 126 MHz) δ (ppm): 26.1, 56.0, 79.8, 112.7, 126.4, 128.2, 129.0, 129.5, 130.7, 136.8, 149.8, 196.5. MS (ES+) m/z = 285.10 [MH^+], 224.14, 135.09, 104.07, 93.08, 42.99.

8i 4-((2-Nitro-1-phenylethyl)amino)benzonitrile. Red thick oil. IR (neat NaCl, cm^{-1}): ν = 3337, 3059, 3027, 3005, 2965, 2915, 2843, 2215, 1605, 1558, 1519, 1494, 1458, 1417, 1377, 1343, 1311, 1278, 1217, 1179, 1139, 1102, 1074, 1028, 1001, 968, 920, 825, 761, 700, 668, 627, 606, 546. ^{1}H NMR ($CDCl_3$, 500 MHz) δ (ppm): 4.71 (dd, 1H, Ha, *J*= 8.5 Hz, 12.5 Hz), 4.76 (dd, 1H, Hb, *J*= 4.7 Hz, 12.5 Hz), 5.03 (broad d, 1H, Hc, *J*=6.5 Hz), 5.20 (m, 1H Hd), 6.60 (d, 2H, He, J_o=8.9 Hz, J_m=2.0 Hz), 7.34-7.38 (m, 3H, Hf, Hg), 7.38-7.42 (m, 4H, Hh, Hi). ^{13}C NMR ($CDCl_3$, 126 MHz) δ (ppm): 55.9, 79.7, 100.7, 113.5, 120.0, 126.3, 129.1, 129.6, 133.8, 136.9, 149.2. MS (ES+) m/z = 268.10 [MH^+], 221.12, 143.08, 118.10, 91.05.

8j 3-Nitro-*N*-(2-nitro-1-phenylethyl)aniline. Red thick oil. IR (neat NaCl, cm^{-1}): ν = 3392, 3085, 3066, 3030, 3004, 2971, 2921, 2862, 1623, 1561, 1526, 1507, 1459, 1424, 1379, 1348, 1276, 1220, 1129, 1099, 1081, 998, 964, 890, 853, 817, 793, 761, 737, 700, 670, 624, 529. ^{1}H NMR ($CDCl_3$, 500 MHz) δ (ppm): 4.70-4.75 (dd, 1H, Ha, *J*=8.7 Hz, 12.4 Hz), 4.75-4.80 (dd, 1H, Hb, *J*=4.5 Hz, 12.4 Hz), 4.88 (d, 1H, Hc, *J*=6.7 Hz), 5.23 (d, 1H, Hd, *J*=4.7, 6.6, 8.5 Hz), 6.89 (d, 1H, He, J_o=8.2 Hz, J_m=2.0 Hz), 7.25 (t, 1H, Hf, *J*=8.2 Hz), 7.34-7.36 (m, 1H, Hg), 7.38-7.42 (m, 5H, Hh, Hi, Hj), 7.55 (d, 1H, Hk J_o=8.2 Hz, J_m=1.4 Hz). ^{13}C NMR ($CDCl_3$, 126 MHz) δ (ppm): 56.4, 79.9, 108.0, 113.5, 119.6, 126.3, 129.1, 129.6, 130.0, 136.4, 146.6, 149.3. MS (ES+) m/z = 288.08 [MH^+], 150.05, 138.99, 104.06, 91.97, 77.99.

8k 2,5-Dichloro-*N*-(2-nitro-1-phenylethyl)aniline. Red thick oil. IR (neat NaCl, cm^{-1}): ν = 3483, 3396, 3090, 3067, 3031, 2963, 2921, 2853, 1594, 1559, 1508, 1485, 1459, 1417, 1378, 1343, 1294, 1211, 1136, 1094, 1046, 952, 910, 835, 794, 768, 700, 613, 581, 532. ^{1}H NMR ($CDCl_3$, 500 MHz) δ (ppm): 4.75 (m, 2H, Ha), 5.18 (q, 1H, Hb, *J*=6.4 Hz), 5.23 (d, 1H, Hc, *J*=6.9 Hz), 6.51 (s, 1H, Hd, J_m=2.3 Hz), 6.65 (d, 1H, He, J_o=8.4 Hz, J_m =2.3 Hz), 7.18 (d, 1H, Hf, *J*=8.4 Hz), 7.35-7.42 (m, 5H, Hg, Hh, Hi). ^{13}C NMR ($CDCl_3$, 126 MHz) δ (ppm): 56.3, 79.9, 112.4, 118.2, 118.7, 126.3, 129.1, 129.6, 130.0, 133.6, 136.4, 142.5. MS (ES+) m/z = 312.97 [MH^+], 252.02, 217.96, 173.91, 101.73, 93.98.

8l (*E*)-*N*-(2-nitro-1-phenylethyl)-4-(phenyldiazenyl)aniline. Red solid. m.p. 103-105°C. IR (neat, KBr, cm^{-1}): ν = 3483, 3396, 3090, 3067, 3031, 2963, 2921, 2853, 1594, 1559, 1508, 1485, 1459, 1417, 1378, 1343, 1294, 1211, 1136, 1094, 1046, 952, 910, 835, 794, 768, 700, 613, 581, 532. ^{1}H NMR ($CDCl_3$, 500 MHz) δ (ppm): 4.77 (m, 1H, Ha), 4.88 (broad, 1H, Hb), 5.28 (broad, 1H, Hc), 6.69 (d, 2H, Hd, J_o=6.8 Hz, J_m= 2.1 Hz), 7.32-7.42 (m, 6H, He, Hf, Hg, Hh), 7.46 (t, 2H, Hi, J_o=7.5 Hz, J_m=1.5 Hz), 7.79 (d, 2H, Hj, J_o=6.9 Hz, J_m =2.1 Hz), 7.82 (d, 2H, Hk, *J*=8.4 Hz). ^{13}C NMR ($CDCl_3$, 126 MHz) δ (ppm): 56.3, 79.8, 113.6, 122.4, 125.1, 126.4, 128.9, 129.0, 129.5, 130.0, 137.0, 145.8, 148.2, 152.9. MS (ES+) m/z = 347.16 [MH^+], 197.07, 104.13, 91.97, 77.07, 65.04.

9a *N*-(2-nitro-1-phenylpropyl)aniline [43,59]. Yellow-red thick oil. IR (neat NaCl, cm^{-1}): ν = 3400, 3086, 3058, 3031, 3005, 2941, 2903, 2876, 1607, 1559, 1508, 1455, 1436, 1391, 1361, 1320, 1297, 1284, 1255, 1201, 1181, 1155, 1142, 1081, 1033, 997, 913, 874, 839, 755, 693, 610, 561, 522. NMR data is for each pair of diastereomers: ^{1}H NMR ($CDCl_3$, 500 MHz) δ (ppm): 1.49 (d, 3H, Ha, *J*=6.6 Hz), 4.50 (d, 1H, Hb, *J*=8.0 Hz), 4.79 (t, 1H, Hc, *J*=8.2 Hz), 4.87 (m, 1H, Hd), 6.59 (d, 2H, He, J_o=8.7 Hz, J_m=1.0 Hz), 6.70 (t, 1H, Hf, J_o=7.3 Hz, J_m=1.0 Hz), 7.10 (t, 2H, Hg, J_o=7.9 Hz, J_m=1.2 Hz), 7.28-7.37 (m, 5H, Hh, Hi, Hj). ^{1}H NMR ($CDCl_3$, 500 MHz) δ (ppm): 1.55 (d, 3H, Ha, J_m=1.6 Hz), 4.44 (d, 1H, Hb, *J*=6.6 Hz), 4.94 (m, 1H, Hc), 5.01 (m, 1H, Hd), 6.57 (d, 2H, He, *J*=8.5 Hz), 6.72 (t, 1H, Hf, J_o=7.3 Hz, J_m=0.9 Hz), 7.11 (t, 2H, Hg, J_o=8.0 Hz, J_m=1.0 Hz), 7.30-7.37 (m, 5H, Hh, Hi, Hj).

9b *N*-(2-nitro-1-phenylbutyl)aniline [36]. Red solid. m.p. 114-116°C. IR (neat, KBr, cm^{-1}): ν = 3385, 3090, 3068, 3036, 2972, 2938, 2912, 2877, 1606, 1546, 1511, 1460, 1438, 1374, 1339, 1314, 1279, 1257, 1231, 1206, 1181, 1158, 1136, 1114, 1082, 1034, 1000, 917, 892, 872, 856, 834, 803, 771, 748, 707, 688, 628, 606, 513, 495. NMR data is for each pair of

diastereomers: ^{1}H NMR ($CDCl_3$, 500 MHz) δ (ppm): 0.94 (t, 3H, Ha, *J* =7.4 Hz), 1.65 (m, 1H, Hb), 2.09 (m, 1H, Hc), 4.57 (d, 1H, Hd, *J*=8.6 Hz), 4.68 (ddd, 1H, He, *J*=11.1 Hz, 7.9 Hz, 4.0 Hz), 4.80 (t, 1H, Hf, *J*=8.3 Hz), 6.57 (2H, Hg, *J*=8.8 Hz), 6.68 (t, 1H, Hh, J_o=7.3 Hz, J_m=1.0 Hz), 7.09 (t, 2H, Hi, J_o=8.0 Hz, J_m=2.0 Hz), 7.28-7.36 (m, 5H, Hj, Hk, Hl). ^{13}C NMR ($CDCl_3$, 126 MHz) δ (ppm): 10.4, 24.8, 60.5, 94.8, 113.9, 118.6, 126.9, 128.5, 129.1, 129.2, 138.1, 145.9. ^{1}H NMR ($CDCl_3$, 500 MHz) δ (ppm): 0.97 (t, 3H, Ha, *J*=7.3 Hz), 1.90 (m, 1H, Hb), 2.17 (m, 1H, Hc), 4.38 (d, 1H, Hd, *J* =6.5 Hz), 4.75 (ddd, 1H, He, *J* =11.0 Hz, 5.8 Hz, 3.1 Hz), 4.90 (t, 1H, Hf, *J* =6.1 Hz), 6.56 (d, 2H, Hg, J_o=8.7 Hz, J_m=1.0 Hz), 6.72 (t, 1H, Hh, J_o=7.3 Hz, J_m=1.0 Hz), 7.11 (t, 2H, Hi, J_o=8.0 Hz, J_m=2.0 Hz), 7.28-7.36 (m, 5H, Hj, Hk, Hl). ^{13}C NMR ($CDCl_3$, 126 MHz) δ (ppm): 10.7, 22.3, 60.6, 94.0, 114.0, 118.7, 126.9, 128.6, 129.0, 129.3, 137.7, 146.1. MS (ES+) m/z = 271.16 [MH^+], 148.03, 77.07, 56.19.

4a 3-Nitro-*N*-(2-nitro-1-(*p*-tolyl)ethyl)aniline. Red thick oil. IR (neat NaCl, cm^{-1}): ν = 3398, 3089, 3027, 2923, 2864, 1624, 1588, 1559, 1536, 1422, 1380, 1351, 1266, 1214, 1181, 1109, 1096, 1071, 1044, 1025, 996, 966, 918, 859, 820, 794, 758, 739, 673, 521. ^{1}H NMR ($CDCl_3$, 500 MHz) δ (ppm): 2.34 (s, 3a, Ha), 4.72 (m, 2H, Hb), 4.86 (d, 1H, Hc, *J*=6.7 Hz), 5.19 (ddd, 1H, Hd, *J*=8.4 Hz, 7.0 Hz, 4.7 Hz), 6.89 (d, 1H, He, J_o=8.2 Hz, J_m=0.7 Hz), 7.2 (d, 2H, Hf, *J*=8.9 Hz), 7.24-7.29 (m, 3H, Hg, Hh), 7.41 (s, 1H, Hi, J_m=2.3 Hz), 7.56 (d, 1H, Hj, J_o=8.1 Hz, J_m=0.8 Hz). ^{13}C NMR ($CDCl_3$, 126 MHz) δ (ppm): 21.1, 56.2, 80.0, 108.0, 113.4, 119.6, 126.2, 130.0, 130.2, 133.4, 139.0, 146.7, 149.3. MS (ES+) m/z = 302.10 [MH^+], 164.08, 118.10, 121.03, 117.71, 91.05.

4b 4-Chloro-*N*-(1-(4-methoxyphenyl)-2-nitroethyl)aniline. Red thick oil. IR (neat NaCl, cm^{-1}): ν = 3390, 3106, 3070, 3034, 3008, 2962, 2936, 2916, 2838, 1605, 1559, 1513, 1497, 1464, 1421, 1379, 1340, 1310, 1254, 1176, 1121, 1091, 1032, 970, 826, 783, 731, 682, 640, 564, 548, 525, 509. ^{1}H NMR ($CDCl_3$, 500 MHz) δ (ppm): 3.79 (s, 3H, Ha), 4.4 (d, 1H, Hb, *J*=6.4 Hz), 4.67 (d, 2H, Hc, *J*=6.7 Hz), 5.06 (q, 1H, Hd, *J*=6.7 Hz), 6.52 (d, 2H, He, *J*=8.9 Hz), 6.90 (d, 2H, Hf, *J*=8.8 Hz), 7.08 (d, 2H, Hg, *J*=8.9 Hz), 7.28 (d, 2H, Hh, *J*=8.6 Hz). ^{13}C NMR ($CDCl_3$, 126 MHz) δ (ppm): 55.3, 56.3, 80.1, 114.8, 115.1, 127.6, 129.2, 131.2, 139.1, 144.3, 159.9. MS (ES+) m/z = 307.09 [MH^+], 180.05, 119.04, 91.06.

4 c 3-(1-((4-methoxyphenyl)amino)-2-nitroethyl)phenol. Dark-red thick oil. IR (neat NaCl, cm^{-1}): ν = 3361, 3306, 3066, 3035, 3000, 2952, 2931, 2827, 1591, 1560, 1513, 1457, 1377, 1298, 1239, 1183, 1127, 1035, 997, 934, 827, 788, 730, 699, 517. ^{1}H NMR ($CDCl_3$, 500 MHz) δ (ppm): 3.71 (s, 3H, Ha), 4.66 (d, 2H, Hb, *J*=6.7 Hz), 4.74 (m, 1H, Hc), 5.02 (t, 1H, Hd, *J*=6.7 Hz), 5.16-5.38 (broad, 1H, He), 6.57 (d, 2H, Hf, J_o=8.9 Hz, J_m=2.3 Hz), 6.73 (d, 2H, Hg, J_o=8.9 Hz, J_m=2.2 Hz), 6.77 (d, 1H, Hh, J_o=9.9 Hz, J_m=0.6 Hz), 6.85 (s, 1H, Hi, J_m=2.0 Hz), 6.94 (d, 1H, Hj, *J*=8.6 Hz, J_m=1.5 Hz), 7.24 (t, 1H, Hk, *J*=7.9 Hz). ^{13}C NMR ($CDCl_3$, 126 MHz) δ (ppm): 55.7, 57.5, 80.0, 114.6, 115.0, 115.7, 117.2, 122.4, 130.2, 130.6, 139.7, 139.8, 144.0, 153.0, 156.5. MS (ES+) m/z = 289.07 [MH^+], 200.15, 166.06, 123.92.

4d 4-bromo-*N*-(1-cyclohexyl-2-nitroethyl)aniline. Red solid. IR m.p. 74-75°C. IR (neat, KBr, cm^{-1}): ν = 3405, 3386, 3100, 3077, 3064, 3038, 2929, 2851, 1598, 1558, 1509, 1489, 1446, 1424, 1387, 1354, 1318, 1299, 1250, 1217, 1184, 1155, 1135, 1115, 1096, 1079, 1030, 1014,

1000, 971, 931, 915, 892, 816, 803, 745, 699, 672, 639, 597, 508, 442, 423. ^{1}H NMR ($CDCl_3$, 500 MHz) δ (ppm): 1.00-1.30 (m, 5H, Ha, Hb, Hc), 1.60 (m, 1H, Hd), 1.65-1.85 (m, He, Hf), 1.90 (m, Hg), 3.72 (d, 1H, Hh, *J*=9.8 Hz), 3.92 (m, 1H, Hi), 4.46, (dd, 1H, Hj, *J*=6.8 Hz, 12.2 Hz), 4.53 (dd, 1H, Hk, *J*=5.2 Hz, 12.2 Hz), 6.53 (d, 2H, Hl, *J*=7.4 Hz), 7.26 (m, 2H, Hm). ^{13}C NMR ($CDCl_3$, 126 MHz) δ (ppm): 25.9, 26.1, 28.9, 29.6, 40.8, 57.2, 76.6, 110.0, 115.0, 132.3, 145.7. MS (ES+) m/z = 325.03 [MH$^+$], 169.93, 154.07, 136.05, 78.89, 60.02.

4e 4-methyl-*N*-(2-nitro-1-(2-nitrophenyl)ethyl)aniline. Yellow-red solid. m.p. 85-87°C. IR (neat, KBr, cm^{-1}): ν = 3375, 3104, 3078, 3030, 2920, 2862, 1622, 1580, 1560, 1522, 1474, 1435, 1386, 1340, 1289, 1263, 1250, 1215, 1182, 1154, 1131, 1086, 1066, 937, 921, 879, 860, 851, 811, 792, 750, 724, 708, 673, 647, 627, 560, 517, 505. ^{1}H NMR ($CDCl_3$, 500 MHz) δ (ppm): 2.18 (s, 3H, Ha), 4.64-4.86 (broad, 1H, Hb), 4.83 (dd, 1H, Hc, *J*=7.6 Hz, 12.5 Hz), 5.00 (dd, 1H, Hd, *J*=3.7 Hz, 12.5 Hz), 5.76 (dd, 1H, He, *J*=3.7 Hz, 7.6 Hz), 6.40 (d, 2H, Hf, *J*=8.5 Hz), 6.66 (d, 2H, Hg, *J*=8.5Hz), 7.49 (t, 1H, Hh, J_o=7.8 Hz, J_m=1.5 Hz), 7.60 (t, 1H, Hi, J_o=7.6 Hz, J_m=1.4 Hz), 7.68 (d, 1H, Hj, J_o=7.8 Hz, J_m=1.4 Hz), 8.08 (d, 1H, Hk, J_o=8.1 Hz, J_m=1.4 Hz). ^{13}C NMR ($CDCl_3$, 126 MHz) δ (ppm): 20.4, 52.9, 79.3, 113.8, 125.7, 128.7, 129.0, 129.6, 130.0, 133.5, 134.4, 142.5, 148.7. MS (ES+) m/z = 302.10 [MH$^+$], 241.15, 120.05, 119.73, 107.05.

4g *N*-(1-(4-bromophenyl)-2-nitroethyl)-4-ethylaniline. Red oil. IR (neat NaCl, cm^{-1}): ν = 3377, 3086, 3015, 2957, 2919, 2860, 1604, 1582, 1547, 1511, 1478, 1446, 1414, 1369, 1330, 1307, 1259, 1211, 1178, 1126, 1107, 1097, 1069, 1039, 1007, 962, 917, 820, 759, 726, 661, 648, 532. ^{1}H NMR ($CDCl_3$, 500 MHz) δ (ppm): 1.15 (t, 3H, Ha, *J*=7.6 Hz), 2.50 (q, 2H, Hb, *J*=7.6 Hz), 4.29 (d, 1H, Hc, *J*=6.1 Hz), 4.67 (d, 2H, Hd, *J*=6.7 Hz), 5.09 (q, 1H, He, *J*=6.5 Hz), 6.52 (d, 2H, Hf, J_o=8.5 Hz, J_m=2.0 Hz), 6.98 (d, 2H, Hg, *J*=8.5 Hz), 7.28 (d, 2H, Hh, J_o=8.4 Hz, J_m=1.7 Hz), 7.50 (d, 2H, Hi, J_o=8.5 Hz, J_m=1.9 Hz). ^{13}C NMR ($CDCl_3$, 126 MHz) δ (ppm): 15.8, 27.9, 56.5, 79.8, 114.1, 122.6, 128.2, 128.8, 132.5, 135.2, 137.0, 143.2. MS (ES+) m/z = 351.08 [MH$^+$], 335.77, 180.26, 90.98, 64.97.

4h 4-methyl-N-(1-nitropentan-2-yl)aniline. Red oil. IR (neat NaCl, cm^{-1}): ν = 3398, 3027, 2965, 2933, 2871, 1621, 1588, 1553, 1520, 1468, 1429, 1383, 1354, 1321, 1305, 1259, 1230, 1208, 1188, 1155, 1123, 1038, 993, 921, 813, 735, 644, 628, 511. ^{1}H NMR ($CDCl_3$, 500 MHz) δ (ppm): 0.95 (t, 3H, Ha, *J*=7.3 Hz), 1.38-1.68 (m, 4H, Hb, Hc), 2.24 (s, 3H, Hd), 3.52 (d, 1H, He, *J*=7.9 Hz), 4.03 (q, 1H, Hf, *J*=5.8 Hz), 4.41 (dd, 1H, Hg, *J*=6.1 Hz, 11.7 Hz), 4.52 (dd, 1H, Hh, *J*=5.0 Hz, 11.7 Hz), 6.58 (d, 2H, Hi, *J*=8.4 Hz), 7.01 (d, 2H, Hj, *J*=8.0 Hz). ^{13}C NMR ($CDCl_3$, 126 MHz) δ (ppm): 13.8, 19.2, 20.4, 35.1, 52.4, 78.1, 113.8, 128.0, 130.1, 143.7. MS (ES+) m/z = 229.07 [MH$^+$], 199.24, 184.74, 118.00, 91.06.

4i (*E*)-*N*-(2-nitro-1-(*p*-tolyl)ethyl)-4-(phenyldiazenyl)aniline. Dark-red thick oil. IR (neat NaCl, cm^{-1}): ν = 3397, 3064, 3035, 2956, 2924, 2860, 1605, 1558, 1516, 1460, 1434, 1411, 1377, 1339, 1313, 1278, 1240, 1186, 1142, 1100, 1075, 1024, 967, 919, 862, 834, 767, 722, 687, 668, 640, 548, 532. ^{1}H NMR ($CDCl_3$, 500 MHz) δ (ppm): 2.34 (s, 3H, Ha), 4.75 (d, Hb, 2H, *J*=6.6 Hz), 4.84 (d, Hc, 1H, *J*=6.7 Hz), 5.24 (m, Hd, 1H), 6.69 (d, 2H, He, *J*=8.9 Hz), 7.20 (d, 2H, Hf, *J*=7.9 Hz), 7.28 (d, 2H, Hg, *J*=8.1 Hz), 7.39 (t, 1H, Hh, J_o=7.3 Hz, J_m=1.3

Hz), 7.46 (t, 2H, Hi, J=7.9 Hz), 7.79 (d, 2H, Hj, J_o=8.9 Hz, J_m=2.0 Hz), 7.81 (d, 2H, Hk, J=8.3 Hz). ^{13}C NMR ($CDCl_3$, 126 MHz) δ (ppm): 21.1, 56.1, 79.9, 113.5, 122.4, 125.1, 126.3, 129.0, 129.9, 130.1, 133.9, 138.8, 145.7, 148.3, 153.0. MS (ES+) m/z = 361.18 [MH^+], 197.18, 118.04, 91.90, 77.00, 64.97.

4j 1-(4-((1-(4-methoxyphenyl)-2-nitropropyl)amino)phenyl)ethanone. Yellow-red thick oil. IR (neat NaCl, cm^{-1}): ν = 3348, 3059, 3004, 2962, 2939, 2906, 2839, 1656, 1602, 1556, 1514, 1458, 1439, 1426, 1388, 1365, 1307, 1281, 1254, 1184, 1147, 1122, 1083, 1034, 960, 876, 833, 759, 669, 636, 597, 532. ^{1}H NMR ($CDCl_3$, 500 MHz) δ (ppm): 1.56 (d, 3H, Ha, J=6.8 Hz), 2.46 (s, 3H, Hb), 3.79 (s, 3H, Hc,), 4.80 (t, 1H, Hd, J= 7.9 Hz), 4.99 (m, 1H, He), 5.05 (d, 1H, Hf, J=6.8 Hz), 6.56 (d, 2H, Hg, J= 8.9 Hz), 6.89 (d, 2H, Hh, J=8.8 Hz), 7.21 (d, 2H, Hi, J=8.7 Hz), 7.74 (d, 2H, Hj, J=8.8 Hz). ^{13}C NMR ($CDCl_3$, 126 MHz) δ (ppm): 14.1, 26.1, 55.3, 59.7, 86.3, 112.8, 114.6, 128.0, 130.5, 150.1, 159.8, 196.4. ^{1}H NMR (CDCl3, 500 MHz) δ (ppm): 1.52 (d, Ha, 3H, J= 6.5 Hz), 2.45 (s, Hb, 3H), 3.78 (s, Hc, 3H), 4.85 (t, 1H, Hd, J= 7.8 Hz), 4.94 (m, 1H, He), 5.05 (d, 1H, Hf, J= 6.8 Hz), 6.54 (d, 2H, Hg, J=8.9 Hz), 6.88 (d, 2H, Hh, J= 8.9 Hz), 7.23 (d, 2H, Hi, J=8.6 Hz), 7.76 (d, 2H, Hj, J=8.8 Hz). ^{13}C NMR ($CDCl_3$, 126 MHz) δ (ppm): 17.1, 26.0, 55.3, 60.3, 87.5, 112.7, 114.7, 128.0, 130.5, 150.1, 159.9, 196.4. MS (ES+) m/z = 329.10 [MH^+], 254.11, 148.03, 136.00, 117.01, 105.04.

4k 4-methyl-*N*-(2-nitro-1-(2-nitrophenyl)butyl)aniline. Red thick oil. IR (neat NaCl, cm^{-1}): ν = 3397, 3054, 3025, 2978, 2936, 2920, 2876, 1621, 1593, 1555, 1523, 1488, 1456, 1405, 1377, 1332, 1329, 1300, 1269, 1256, 1186, 1173, 1142, 1110, 1075, 1014, 935, 884, 808, 760, 716, 633, 541. ^{1}H NMR ($CDCl_3$, 500 MHz) δ (ppm): 0.94 (t, 3H, Ha, J=7.4 Hz), 1.66 (m, 1H, Hb), 2.08 (m, 1H, Hc), 2.2 (s, 3H, Hd), 4.46 (broad, 1H, He), 4.63 (m, 1H, Hf), 4.73 (m, 1H, Hg), 6.45 (d, 2H, Hh, J=8.5 Hz), 6.90 (d, 2H, Hi, J=8.7 Hz), 7.18 (m, 2H, Hj), 7.46 (m, 2H, Hk). ^{13}C NMR ($CDCl_3$, 126 MHz) δ (ppm): 10.4, 20.4, 24.8, 60.2, 94.6, 114.1, 122.4, 128.1, 128.6, 129.8, 132.3, 137.4, 143.2.^{1}H NMR (CDCl3, 500 MHz) δ (ppm): 0.97 (t, 3H, Ha, J=7.3 Hz),1.90 (m, 1H, Hb), 2.14 (m, 1H, Hc), 4.24 (broad, 1H, Hd), 4.68 (m, 1H, He), 4.80 (m, 1H, Hf), 6.44 (d, 2H, Hg, J=8.5 Hz), 6.92 (d, 2H, Hh, J=9.1 Hz), 7.20 (m, 2H, Hi), 7.46 (m, 2H, Hj). ^{13}C NMR ($CDCl_3$, 126 MHz) δ (ppm): 10.7, 22.4, 24.8, 60.4, 93.7, 114.2, 122.4, 128.4, 128.6, 129.8, 132.2, 137.0, 143.4. MS (ES+) m/z = 314.12, 274.11, 210.04, 178.06, 131.10, 108.09, 93.04, 91.03, 60.81.

References

1 . Kolb, H. C.; Finn, M. G.; Sharpless, K. B. 2001. Click Chemistry: Diverse Chemical Function from a few Good Reactions. *Angew. Chem. Int. Ed.*, 40:2004-2021.
2. Noble, A.; Anderson, J. C. 2013. Nitro-Mannich Reaction. *Chem. Rev.*, 113:2887-2939.
3. El-Sayed, M. T.; Mahmoud, K.; Hilgeroth, A. 2013. Synthesis of β-Nitroamines via Classical Mannich and Aza-Henry Reactions. *Curr. Org. Chem.*, 17:1200-1224.
4. Anderson, J. C.; Koovits, P. J. 2013. An Enantioselective Tandem Reduction/nitro-Mannich Reaction of Nitroalkenes using a Simple Thiourea Organocatalyst. *Chem. Sci.*, 4:2897-2901.
5. Marqués-López, E.; Merino, P.; Tejero, T.; Herrera, R. P. 2009. Catalytic Enantioselective Aza-Henry Reactions. *Eur. J. Org. Chem.*, 15:2401-2420.

6 . Westermann, B. 2003. Asymmetric Catalytic Aza- Henry Reactions Leading to 1,2-Diamines and 1,2- Diaminocarboxylic Acids. *Angew. Chem. Int. Ed.*, 42:151-153.

7. Ballini, R.; Petrini M. 2004. Recent Synthetic Developments in the Nitro to Carbonyl Conversion (Nef Reaction). *Tetrahedron*, 60:1017-1047.

8. Shen, B.; Makley, D. M.; Johnston, J. N. 2010. Umpolung Reactivity in Amide and Peptide Synthesis. *Nature*, *465*:1027-1032.

9. Ruano, J. L. G.; López-Cantarero, J.; de Haro, T.; Alemán, J.; Cid, M. B. 2006. Preparation of α-Amino Ketones, β-Amino Hydroxylamines using Asymmetric aza-Henry Reactions of N-p-Tolylsulfinylimines with Nitroethane. *Tetrahedron*, 62:12197-12203.

10. Czekelius, C.; Carreira, E. M. 2005. Convenient Transformation of Optically Active Nitroalkanes into Chiral Aldoximes and Nitriles. *Angew. Chem. Int. Ed.*, 44:612-615.

11. Tsuritani, N.; Yamada, K.; Yoshikawa, N.; Shibasaki, M. 2002. Catalytic Asymmetric Syntheses of ICI-199441 and CP-99994 using nitro-Mannich Reaction. *Chem. Lett.*, 276-277.

12. Kumaraswamy, G.; Pitchaiah, A. 2011. A Concise Enantioselective Synthesis of L-(−)-733,061 and (2S,3S)-Methyl 3-Aminopiperidine-2-Carboxylate using Catalytic Enantioselective aza-Henry Reaction as Key Step. *Tetrahedron*, 67:2536-2541.

13. Jakubec, P.; Hawkins, A.; Felzmann, W.; Dixon, D. J. 2012. Total Synthesis of Manzamine A and Related Alkaloids. *J. Am. Chem. Soc.*, 134:17482-17485.

14. Handa, S.; Gnanadesikan, V.; Matsunga, S.; Shibasaki, M. 2010. Heterobimetallic Transition Metal/Rare Earth Metal Bifunctional Catalysis: A Cu/Sm/Schiff Base Complex for Syn-Selective Catalytic Asymmetric Nitro-Mannich Reaction. *J. Am. Chem. Soc.*, 132:4925-4934.

15. Davis, T. A.; Danneman, M. W.; Johnston, J. N. 2012. Chiral Proton Catalysis of Secondary Nitroalkane Additions to Azomethine: Synthesis of a Potent GlyT1 Inhibitor. *Chem. Commun.*, 48:5578-5580.

16. Weng, J.; Li, Y.-B.; Wang, R.-B.; Li, F.-Q.; Liu, C.; Chan, A. S. C.; Lu, G. 2010. A Practical and Azide-Free Synthetic Approach to Oseltamivir from Diethyl D-Tartrate. *J. Org. Chem.*, 75:3125-3128.

17. Davis, T. A.; Johnston, J. N. 2011. Catalytic, Enantioselective Synthesis of Stilbene cis-Diamines: a Concise Preparation of (−)-Nutlin-3, a Potent p53/MDM2 Inhibitor. *Chem. Sci.*, 2:1076-1079.

18. Vara, B. A.; Mayasundari, A.; Tellis, J. C.; Danneman, M. W.; Arredondo, V.; Davis, T. A.; Min, J.; Finch, K.; Guy, R. K.; Johnston, J. N. 2014. Organocatalytic, Diastereo-and Enantioselective Synthesis of Nonsymmetric cis-Stilbene Diamines: A Platform for the Preparation of Single-Enantiomer cis-Imidazolines for Protein–Protein Inhibition. *J. Org. Chem.*, 79:6913-6938.

19. Xie, H.; Zhang, Y.; Zhang, S.; Chen, X.; Wang, W. 2011. Bifunctional Cinchona Alkaloid Thiourea Catalyzed Highly Efficient, Enantioselective Aza- Henry Reaction of Cyclic Trifluoromethyl Ketimines: Synthesis of Anti- HIV Drug DPC 083. *Angew. Chem. Int. Ed.*, 123:11977-11980.

20. Choudhary, M. K.; Das, A.; Kureshy, R. I.; Kumar, M.; Noorul, H. K.; Abdi, S. H.; Bajaj, H. C. 2014. Chiral Cu (II)-Amino Alcohol Based Complexes for Asymmetric aza-Henry Reaction of N-Ts Imines. *Catal. Sci. Technol.*, 4:548-555.

21. Hynes, P. S.; Stupple, P. A.; Dixon, D. J. 2008. Organocatalytic Asymmetric Total Synthesis of (R)-Rolipram and Formal Synthesis of (3S, 4R)-Paroxetine. *Org. Lett.*, 10:1389-1391.

22. Jakubec, P.; Cockfield, D. M.; Dixon, D. J. 2009. Total Synthesis of (−)-Nakadomarin A. *J. Am. Chem. Soc.*, 131:16632-16633.

23. Kumaraswamy, G.; Pitchaiah, A. 2011. Highly Enantioselective Synthesis of Orthogonally Protected (2S)- 2,3- Diaminopropanoates through Catalytic Phase- Transfer Aza- Henry Reaction. *Helv. Chim. Acta*, 94:1543-1550.
24. Walvoord, R. R.; Kozlowski, M. C. 2015. Cinchonidinium Acetate as a Convenient Catalyst for the Asymmetric Synthesis of cis-Stilbenediamines. *Tetrahedron Lett.*, 56:3070-3074.
25. Bernardi, L.; Bonini, B. F.; Dessole, G.; Fochi, M.; Comes-Franchini, M.; Gavioli, S.; Ricci, A.; Varchi, G. 2003. Concise and Stereocontrolled Synthesis of Pseudo-C2-symmetric Diamino Alcohols and Triamines for Use in HIV Protease Inhibitors. *J. Org. Chem.*, 68:1418-1425.
26. Anderson, J. C.; Blake, A. J.; Howell, G. P.; Wilson, C. 2005. Scope and Limitations of the nitro-Mannich Reaction for the Stereoselective Synthesis of 1, 2-Diamines *J. Org. Chem.*, 70:549-555.
27. Pelagalli, A.; Pellacani, L.; Scandozza, E.; Fioravanti, S. 2016. Aza-Henry Reactions on C-Alkyl Substituted Aldimines. *Molecules*, 21:723-823.
28. Adams, H.; Anderson, J. C.; Peace, S.; Pennel, A. M. K. 1998. The nitro-Mannich Reaction and its Application to the Stereoselective Synthesis of 1, 2-diamines. *J. Org. Chem.*, 63:9932-9934.
29. Rodríguez-Solla , H.; Concellon, C.; Alvaredo , N.; Soengas R. G. 2012. The Use of Samarium or Sodium Iodide Salts as an Alternative for the aza-Henry Reaction. *Tetrahedron*, 68:1736-1744.
30. Wang, Y.-H.; Liu, Y.-L.; Cao, Z.-Y.; Zhou, J. 2014. Aza-Henry Reaction of Ketimines Catalyzed by Guanidine and Phosphazene Bases. *Asian J. Org. Chem.*, 3:429-432.
31. Kutovaya, I. V.; Shmatova, O. I.; Tkachuk, V. M.; Melnichenko, N. V.; Vovk, M. V.; Nenajdenko, V. G. 2015. Aza- Henry Reaction with CF_3- Ketimines: An Efficient Approach to Trifluoromethylated β- Nitroamines, 1,2- Diamines, α- Aminooximes, and Imidazolidinones. *Eur. J. Org. Chem.*, 30:6749-6761.
32. Ballini, R.; Gabrielli, S.; Palmieri, A.; Petrini, M. 2014. Reaction of α-Amido Sulfones with Functionalized Nitrocompounds: a New Two-Step Synthesis of N-Alkoxycarbonyl-2,5-Disubstituted Pyrroles. *RSC Adv.*, 4:43258-43261.
33. Komura, K.; Taninaka, Y.; Ohtaki, Y.; Sugi, Y. 2010. H–Y Zeolite is a Versatile Heterogeneous Catalyst for the Synthesis of β-Nitroamines. *Appl. Catal., A*, 388:211-215.
34. Chakrapani, L.; Kantam, M. L. 2011. Synthesis of β-Nitroamines Using Nanocrystalline MgO. *Synth. Commun.*, 41:3442–3447.
35. Soengas, R. G.; Silva, A. M. S. 2013. One-Pot Three-Component Barbier-Type Reaction for the Synthesis of β-Nitroamines. *Synlett*, 24:1949-1952.
36. Piscopo, C. G.; Sartori, G.; Mayoral, J. A.; Lanari, D.; Vaccaro, L.; Maggi, R. 2013. Study on the Influence of a Sustainable Medium for the Design of Multistep Processes: Three-Component Synthesis of 2-Nitroamines. *Synlett*, 2596-2600.
37. Cruz-Acosta, F.; de Armas, P.; Garcia-Tellado, F. 2013. Water- Compatible Hydrogen-Bond Activation: A Scalable and Organocatalytic Model for the Stereoselective Multicomponent Aza- Henry Reaction. *Chem. Eur. J.*, 19:16550-16554.
38. Sharma, Y. C.; Singh, B. 2011. Advancements in Solid Acid Catalysts for Ecofriendly and Economically Viable Synthesis of Biodiesel. *Biofuels, Bioprod. Bioref.,* 5*:*69–92.
39. Bosica, G.; Abdilla, R. 2017. A regioselective one-pot aza-Friedel-Crafts reaction for primary, secondary and tertiary anilines using a heterogeneous catalyst. *Green Chem.* 19:5683-5690.
40. Wang, L.; Tan, C.; Liu, X.; Feng, X. 2008. Aza-Henry Reaction of Ketoimines Catalyzed by Na_2CO_3: An Efficient Way to β-Nitroamines. *Synlett*, 13:2075-2077.

41. Zhang, F.; Liu, Z.-L.; Liu, J.-T. 2011. Asymmetric aza-Henry Reaction of Chiral Fluoroalkyl α, β-Unsaturated N-*tert*-Butanesulfinyl Ketoimines: an Efficient Approach to Enantiopure Fluoroalkylated α,β-Diamines and α,β-Diamino Acids. *Org. Biomol. Chem.*, 9:3625-3628.
42. García-Muñoz, M. J.; Dema, H. K.; Foubelo, F.; Yus, M. 2014. Base-Promoted Diastereoselective Addition of Nitromethane and Nitroethane to N-*tert*-Butylsulfinyl Imines: Synthesis of N-Protected α-Amino Acids and Amino Ketones. *Tetrahedron: Asymmetry*, 25:362-372.
43. Mahasneh, A. S. *Z* . 2006. Synthesis of Some 2-Aminonitroethanes via Silica Gel-Catalyzed nitro-Mannich Reaction. *Naturforsch.*, 61b:1162-1165.
44. Baricordi, N.; Benetti, S.; Biondini, G.; De Risi, C.; Pollini, G. P. 2004. A New 'One-Pot' Synthesis of 2-Substituted 3-Nitro Pyrrolidines through a Multicomponent Domino Reaction. *Tetrahedron Lett.*, 45:1373-1375.
45. Harichandran, G.; Amalraj, S. D.; Shanmugam, P. 2016. Amberlite IRA-400 Cl Resin Catalyzed Synthesis of Secondary Amines and Transformation into N-((1H-indol-3-yl) (heteroaryl) methyl)-N-heteroaryl benzenamines and bis-Indoles via Multicomponent Reaction. *J. Saudi Chem. Soc.*, 1-10.
46. Arunkumar, R.; Subramani, K.; Ravichandran, S. 2010. Montmorillonite K-10 Clay–Catalyzed Microwave Synthesis of some Mannich Bases and their Characterization. *Int. J. ChemTech Res.*, 2:278-281.
47. Vekariya, R. H.; Prajapati, N. P.; Patel, H. D. 2016. Silica-Supported Polyphosphoric Acid (PPA-SiO_2): An Efficient and Reusable Heterogeneous Catalyst for Ecofriendly Organic Synthesis. *Synth. Commun.*, 46:197-219.
48. Ballini, R.; Bosica, G.; Forconi, P. 1996. Nitroaldol (Henry) Reaction Catalyzed by Amberlyst A-21 as a far Superior Heterogeneous Catalyst. *Tetrahedron*, 52:1677-1684.
49. Bosica, G.; Gabarretta, J. 2015. Unprecedented One-Pot Multicomponent Synthesis of Propargylamines using Amberlyst A-21 Supported CuI Under Solvent-Free Conditions. *RSC Adv.* 5:46074-46087.
50. Bosica, G.; Abdilla, R. 2017. The KA^2 coupling reaction under green, solventless, heterogeneous catalysis. *J. Mol. Catal. A: Chemical*, 426:542-549.
51. Das, A.; Choudhary, M. K.; Kureshy, R. I.; Roy, T.; Khan, N. U. H.; Abdi, S. H.; Bajaj, H. C. 2014. Enantioselective Henry and Aza- Henry Reaction in the Synthesis of (R)-Tembamide Using Efficient, Recyclable Polymeric CuII Complexes as Catalyst. *ChemPlusChem*, 79:1138-1146.
52. Girard, C.; Onen, E.; Aufort, M.; Beauviere, S.; Samson, E.; Hercovic, J. 2006. Reusable Polymer-Supported Catalyst for the [3+2] Huisgen Cycloaddition in Automation Protocols. *Org. Lett.*, 8:1689-1692.
53. Keshavarz, M.; Iravani, N.; Ghaedi, A.; Ahmady, A. Z.; Vafaei-Nezhad, M.; Karimi, S. 2013. Macroporous Polymer Supported Azide and Nanocopper (I): Efficient and Reusable Reagent and Catalyst for Multicomponent Click Synthesis of 1,4-Disubstituted-1H-1,2,3-Triazoles from Benzyl Halides. *SpringerPlus*, 2:64-71.
54. Choudhary, G.; Peddinti, R. K. 2011. An Expeditious, Highly Efficient, Catalyst-Free and Solvent-Free Synthesis of Nitroamines and Nitrosulfides by Michael Addition. *Green Chem.*, 13:276-282.
55. Yang, L.; Xia, C.; Huang, H. 2011. Chiral Phosphoric Acid Catalyzed Enantioselective aza-Michael Addition of Aromatic Amines to Nitroolefins. *Chin. J. Chem.*, 32:1573-1576.
56. Tajbakhsh, M.; Farhang, M.; Hosseini, A. A. 2014. MgO Nanoparticles as an Efficient and Reusable Catalyst for aza-Michael Reaction. *J. Iran. Chem. Soc.*, 11:665-672.

57. Soengas, R. G.; Silva, S.; Estévez,A. M.; Estévez, J. C.; Estévez, R. J.; Rodríguez-Solla, H. 2012. Indium-Mediated Aza-Henry Reaction of Imines: Access to 2-Nitroamines. *Eur. J. Org. Chem.*, 23:4339-4346.
58. Ziyaei-Halimehjani, A.; Saidi, M. R. 2008. Synthesis of aza-Henry Products and Enamines in Water by Michael Addition of Amines or Thiols to Activated Unsaturated Compounds. *Tetrahedron Lett.*, 49:1244-1248.
59. He, H.-X.; Yang, W.; Du, D.-M. 2013. Enantioselective Aza-Henry Reaction of Imines Bearing a Benzothiazole Moiety Catalyzed by a Cinchona-Based Squaramide *Adv. Synth. Catal.*, 355:1137-1148.
